# Bulky, electron-rich, renewable: analogues of Beller's phosphine for cross-couplings†

**DOI:** 10.1039/d3cy01375h

**Published:** 2023-10-30

**Authors:** Danielle van der Westhuizen, Abril C. Castro, Nilay Hazari, Ashot Gevorgyan

**Affiliations:** a Department of Chemistry, UiT The Arctic University of Norway 9037 Tromsø Norway gevorgyan.ashot@uit.no; b Hylleraas Centre for Quantum Molecular Sciences, Department of Chemistry, University of Oslo 0315 Oslo Norway; c Department of Chemistry, Yale University New Haven Connecticut 06520 USA

## Abstract

In recent years, considerable progress has been made in the conversion of biomass into renewable chemicals, yet the range of value-added products that can be formed from biomass remains relatively small. Herein, we demonstrate that molecules available from biomass serve as viable starting materials for the synthesis of phosphine ligands, which can be used in homogeneous catalysis. Specifically, we prepared renewable analogues of Beller's ligand (di(1-adamantyl)-*n*-butylphosphine, cataCXium® A), which is widely used in homogeneous catalysis. Our new renewable phosphine ligands facilitate Pd-catalysed Suzuki–Miyaura, Stille, and Buchwald–Hartwig coupling reactions with high yields, and our catalytic results can be rationalized based on the stereoelectronic properties of the ligands. The new phosphine ligands generate catalytic systems that can be applied for the late-stage functionalization of commercial drugs.

## Abstract 2

We have harnessed biomass-derived molecules to create sustainable phosphine ligands for catalysis. These renewable ligands exhibit high activity in Pd-catalyzed cross-couplings, opening new avenues for the late-stage modification of pharmaceuticals.

## Graphical abstract

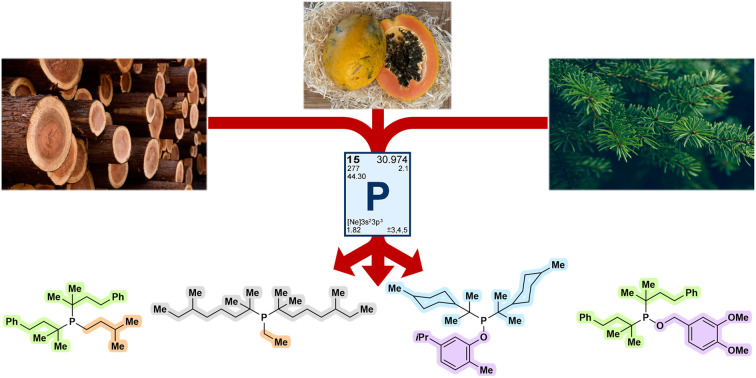

## Introduction

Organic synthesis is currently based predominantly on chemicals obtained from fossil deposits.^1^ These fossil reserves are used to synthesize pharmaceuticals, materials, and other value-added products that we use daily. Unfortunately, fossil resources are not part of the carbon cycle and are not renewable.^2^ Further, we will likely eventually completely deplete our fossil reserves, and currently, there are no renewable alternatives for most of the chemicals required for the normal functioning of modern societies.

Over the last four decades there has been significant interest in finding renewable alternatives for fossil fuel-derived chemicals.^3^ It has been demonstrated that biomass, and particularly lignocellulose, can be selectively converted into platform chemicals, including furanics, phenols, aliphatic carboxylic acids, and alcohols.^3^ Some of these renewable chemicals are potential alternatives for conventional fossil-derived solvents and fuels,^4,5^ but practical systems remain elusive and there is a need for a greater range of products to be produced from biomass.

**Scheme 1:**
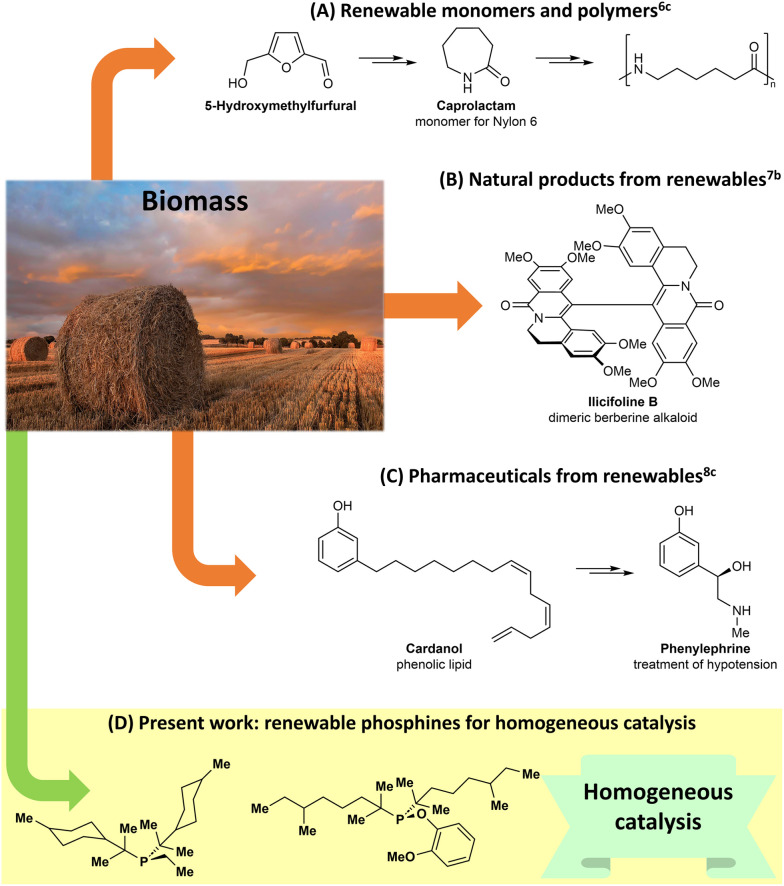
Previous work on the production of value-added products from renewable chemicals (A–C) and the present study showing the development of ligands from biomass-derived chemicals (D). The picture was adapted from the stock image library of Office 365.

More recent studies have started to bridge the gap between biomass conversion and the development of renewable value-added products for a range of applications. For instance, in a work related to the generation of renewable polymers, the groups of de Vries and Heeres designed an alternative sequence to the caprolactam monomer of Nylon 6 starting from 5-hydroxymethylfurfural, which is available from cellulose (Scheme 1A).^6^ In the area of natural product synthesis, Arduengo and Opatz developed elegant synthetic pathways for the preparation of dimeric berberine alkaloids using chemicals derived entirely from wood (Scheme 1B).^7^ Finally, the groups of Barta and Cole-Hamilton performed innovative works on the use of biomass-derived chemicals for the production of pharmaceuticals and bioactive compounds (Scheme 1C).^3*b*,8^ Inspired by these studies, we envisioned that renewable chemicals could be used to build new phosphine ligands for homogeneous catalysis (Scheme 1D). Targeting phosphines was not arbitrary, given that since 2001, the Nobel Prize in Chemistry has been awarded five times for ground-breaking discoveries in homogeneous catalysis.^9^ These include, but are not limited to, awards for the development of asymmetric hydrogenation reactions,^9*a*,*b*^ olefin metathesis,^9*c*^ and Pd-catalyzed cross-couplings,^9*d*,*e*^ which are largely enabled by phosphine ligands.

**Scheme 2:**
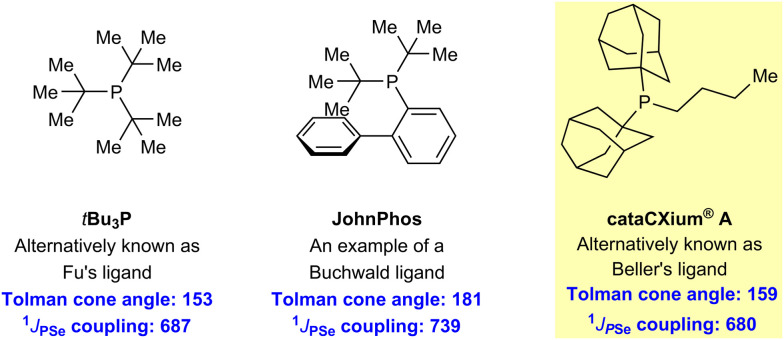
Examples of prominent non-renewable phosphine ligands used in homogeneous catalysis.^19*e*^

Our aim was not the indiscriminate production of renewable phosphine ligands, but rather to reproduce the stereoelectronic properties of fossil-derived phosphine ligands, which have previously exhibited exceptional activity in homogeneous catalysis (Scheme 2).^10,11^ Therefore, we specifically targeted Beller's ligand (cataCXium® A or Ad_2_P*n*Bu, Scheme 2), which is among the most electron-rich phosphines commercially available. Further, various studies have indicated that cataCXium® A-supported complexes exhibit the best balance between steric hindrance and activity in certain catalytic reactions.^10*c*,12^

To achieve activity similar to cataCXium® A, our phosphines likely had to possess the following structural characteristics: (i) at least two alkyl substituents to provide suitable σ-donor properties of the ligand, (ii) two of the substituents on the phosphine should be tertiary alkyl groups to ensure the ligand has adequate steric bulk, and (iii) the third substituent should be a primary alkyl group or a related substituent to avoid excessive steric hindrance preventing ligand binding.

Herein, we describe the synthesis of renewable phosphine ligands containing tertiary alkyl substituents that are obtained from terpenes bearing a tertiary alcohol subunit, which are common by-products in the paper and food industries (Scheme 1D).^13^ Further, some of our ligands feature renewable primary alkyl substituents that are obtained from lignocellulose-derived primary alcohols. Finally, we have extended the diversity of renewable analogues of cataCXium® A by incorporating lignin-derived phenols instead of primary alkyl substituents into phosphine ligands (Scheme 1D).^14^ Many of our new ligands support highly active Pd catalysts for Suzuki–Miyaura, Stille, and Buchwald–Hartwig coupling reactions.

## Results and discussion

**Scheme 3:**
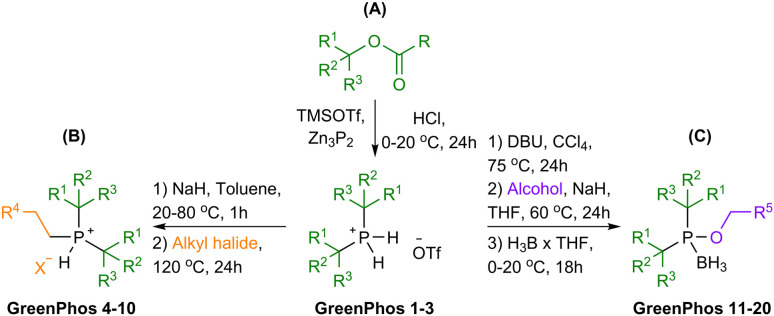
Synthesis of renewable phosphines and phosphinites.

**Scheme 4:**
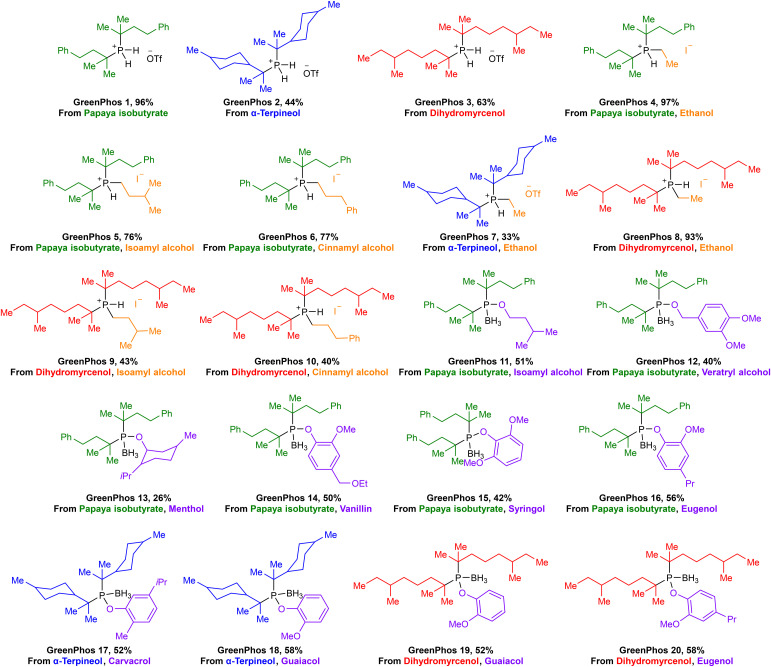
Structures of prepared ligands. Colour codes are used to indicate the origins of functional groups at the ligands.

Renewable analogues of cataCXium® A were prepared according to the sequence of transformations depicted in Scheme 3. Terpenes possessing a tertiary alcohol moiety were transformed into the corresponding esters, which were subsequently phosphorylated *via* an S_N_1 reaction pathway by *ex situ* generated PH_3_ (Scheme 3A).^11*e*,*h*,*i*^ Phosphorylation of the tertiary esters proceeds with high selectivity, affording the corresponding disubstituted phosphines (**GreenPhos 1**–**3**), which are protected by triflic acid (Schemes 3 and 4). We successfully synthesized three dialkylated phosphines with backbones coming from papaya isobutyrate (**GreenPhos 1**, indicated in green), α-terpineol (**GreenPhos 2**, indicated in blue), and dihydromyrcenol (**GreenPhos 3**, indicated in red). These compounds are the first renewable analogues of Beller's phosphine (Scheme 4). They are stable under ambient conditions and serve as pivotal intermediates for further diversification.

The deprotection of dialkylated phosphines (**GreenPhos 1**–**3**) followed by the addition of primary alkyl halides, can facilitate the formation of phosphines possessing two tertiary substituents and a primary alkyl group (**GreenPhos 4**–**10**, Scheme 3B).^15^ S_N_2 alkylation was performed using alkyl halides derived from ethanol, isoamyl alcohol, and cinnamyl alcohol (Scheme 4, indicated in orange). The use of 1.1 equivalents of NaH in the deprotection step results in the formation of protected trisubstituted phosphines, which can be isolated in the form of the corresponding phosphonium salts.

For further diversification of the renewable analogues of cataCXium® A, we proposed and successfully executed the synthesis of phosphinites (**GreenPhos 11**–**20**, Scheme 3C). These compounds were prepared by *in situ* generation of dialkylphosphine chlorides *via* an interrupted Appel reaction.^16^ The dialkylphosphine chlorides can then be alkoxylated with the corresponding alcohols and phenols. More specifically, for alkoxylations we employed a range of renewable substrates, including isoamyl alcohol, veratryl alcohol, menthol, protected vanillyl alcohol, syringol, dihydroeugenol, carvacrol, and guaiacol (Scheme 4, indicated in purple). The phosphinites are susceptible to oxidation upon exposure to air and should be stored as borane adducts.

In total, we synthesized 20 renewable ligands which differ in their structural and electronic properties, with yields ranging from 26% to 97% (Scheme 4). Our next objective was to assess the performance of the new ligands in homogeneous catalysis. To that end, we focused on using the new ligands to facilitate Suzuki–Miyaura, Stille, and Buchwald–Hartwig coupling reactions, given the prevalence of these transformations in medicinal chemistry and the patent literature.^17^

Our preliminary experiments were aimed at establishing appropriate conditions for utilizing the new phosphine ligands, and these studies were performed using **GreenPhos 4** (for further details see ESI,† Tables S2–S28). Optimization of various reaction parameters indicates that for Suzuki–Miyaura coupling **GreenPhos 4** works best in combination with di-μ-mesylbis[2′-(amino-*N*)[1,1′-biphenyl]-2-yl-*C*]dipalladium(ii) (henceforth Pd G3) and KOH in dimethoxyethane (DME) at 20 °C (ESI,† Tables S2–S5). The best results for Stille coupling were achieved utilizing **GreenPhos 4** with Pd G3 and CsF in 2-methyltetrahydrofuran (2MeTHF) at 80 °C (ESI,† Tables S11–S14). The optimal conditions for Buchwald–Hartwig amination were found to be similar to those mentioned above, with the most favourable outcomes for **GreenPhos 4** obtained when using Cs_2_CO_3_ in 1,4-dioxane at 80 °C (ESI,† Tables S20–S23).

The optimized conditions are compatible with unreactive substrates like aryl chlorides and sulfonates, as well as aryl bromides (ESI,† Tables S9, S18 and S27, see also Fig. S16–S18). In relation to aryl chlorides, we observed reactivity patterns similar to those previously observed in the literature.^11,12^ Aryl chlorides are quite reactive even at room temperature when the reactions are conducted in the presence of strong bases. This however significantly restricts the potential utility of the developed methodologies as the use of strong bases leads to limited functional group tolerance (ESI,† Table S10, Fig. S11). In contrast, couplings involving aryl chlorides can be successfully conducted in the presence of mild bases, albeit at elevated temperatures.

**Chart 1:**
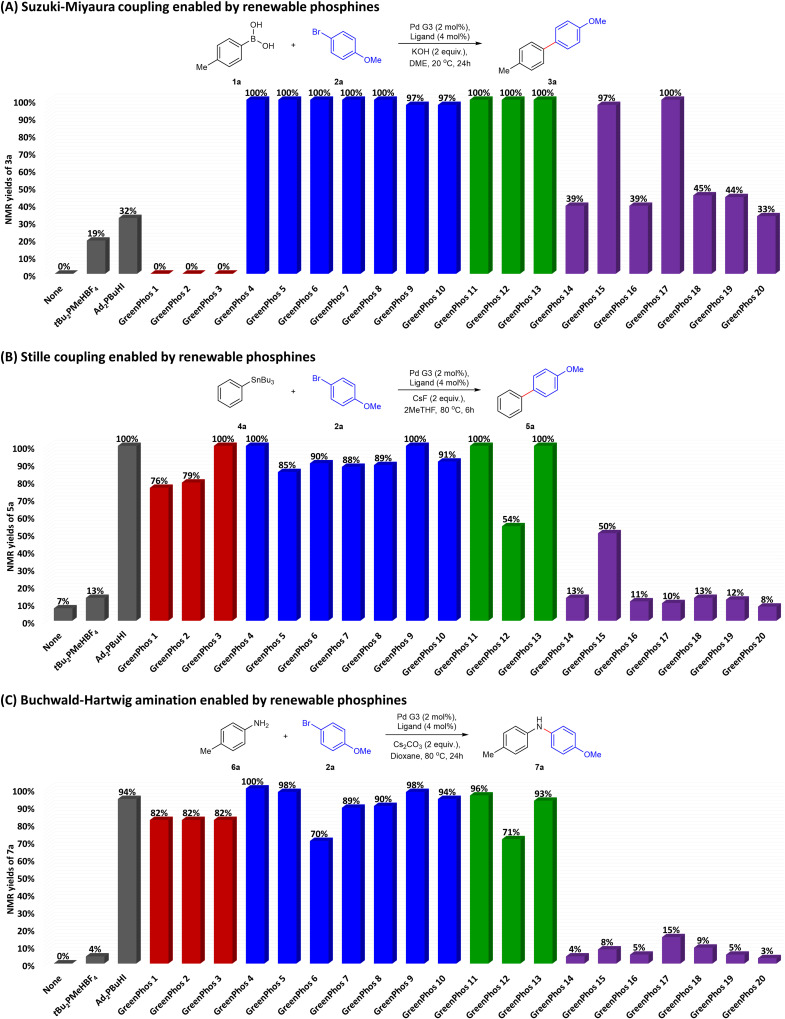
Analysis of the activity of prepared ligands for Suzuki–Miyaura coupling (A), Stille coupling (B) and Buchwald–Hartwig amination (C). Yields determined by ^1^H NMR spectroscopy using 1,3,5-trimethoxybenzene as an internal standard in CDCl_3_.

The positive results obtained from the preliminary optimization using **GreenPhos 4** inspired us to evaluate the performance of other renewable analogues of Beller's phosphine (Chart 1). Our ligand screening studies revealed notable differences in the efficiency of phosphines and phosphinites.

Generally, ligands possessing three alkyl substituents (**GreenPhos 4**–**10**) performed well for all cross-coupling reactions. For Suzuki–Miyaura coupling, **GreenPhos 1**–**3** were not effective, a phenomenon not observed in other cross-couplings (Chart 1A). On the other hand, it is worth noting that the lack of activity using **GreenPhos 1**–**3** was expected, given that these ligands have a proton instead of a primary alkyl group, making them susceptible to arylation at phosphorus and other related reactions.^18^ In contrast, phosphinites based on renewable phenols (**GreenPhos 14**–**20**) were not effective in most cases (Chart 1). However, there were few exceptions as phosphinites based on renewable alcohols (**GreenPhos 11**–**13**) showed reliable effectiveness across all couplings, while **GreenPhos 15** and **GreenPhos 17** demonstrated high activity for Suzuki–Miyaura couplings (Chart 1A). It is worth highlighting that the optimal conditions found for our ligands exhibit general compatibility with cataCXium® A but are not productive for analogous di-*tert*-butylmethylphosphine (Chart 1).

**Scheme 5:**
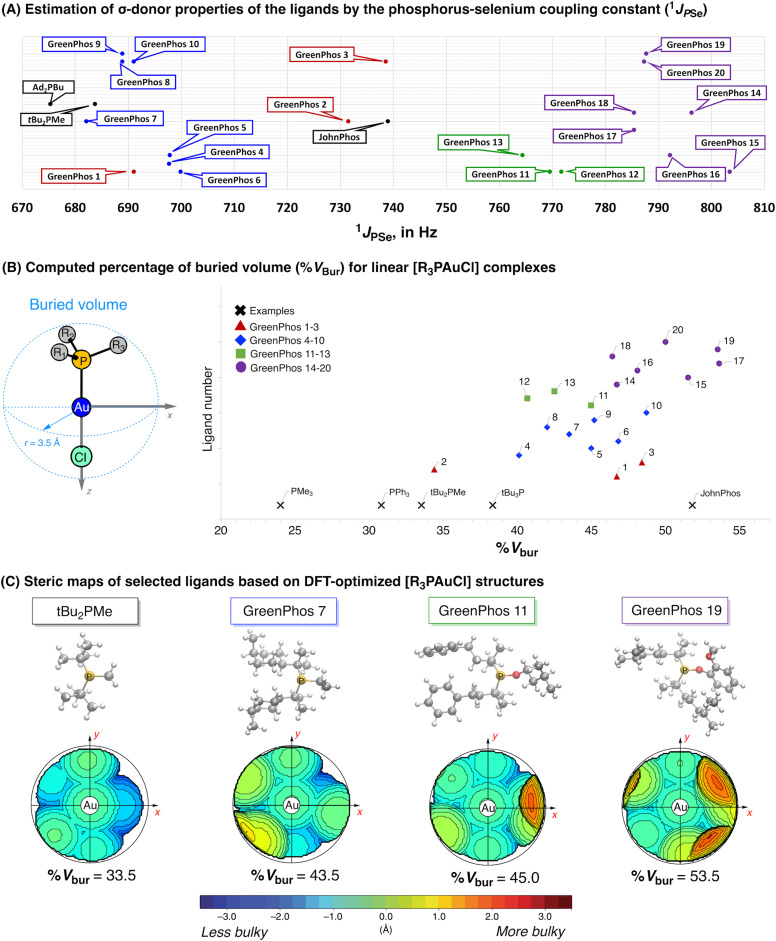
Stereoelectronic properties of prepared ligands (A and B) and steric maps representing catalytic pockets for selected ligands (C).

The poor performance of phosphinites derived from renewable phenols (**GreenPhos 14**–**20**) prompted us to undertake further studies to estimate the stereoelectronic properties of our ligands, as such an analysis could provide valuable insights into the unusual behavior observed in certain reactions (Scheme 5). To evaluate the σ-donor properties of our ligands, we prepared the corresponding selenides of renewable phosphines and phosphinites and measured the ^31^P NMR chemical shifts and associated ^1^*J*_PSe_ couplings of the phosphine selenides (for further details see ESI,† section 4, Table S1).^19^ These measurements revealed that the ligands containing three alkyl substituents (**GreenPhos 4**–**10**) exhibit a σ-donor ability that is comparable to that of Beller's phosphine (Scheme 5A). **GreenPhos 1** also proved to be a strong σ-donor, while related **GreenPhos 2** and **GreenPhos 3** are somewhat less nucleophilic and fall within the range characteristic for Buchwald's phosphines (*e.g.*, JohnPhos). Meanwhile, phosphinites (**GreenPhos 11**–**20**), and in particular, those derived from renewable phenols (**GreenPhos 14**–**20**), are notably less nucleophilic. Although this may partially account for the decreased activity of **GreenPhos 14**–**20** in catalysis, it does not fully explain it, as phosphinites based on renewable alcohols (**GreenPhos 11**–**13**) demonstrate comparable σ-donor abilities despite their high catalytic activity (Chart 1, Scheme 5A). The P–Se coupling constant for **GreenPhos 12** suggests that this ligand is situated on the threshold between highly active and inactive ligands. It is possible that the σ-donor abilities of **GreenPhos 12** are sufficient to meet the minimal requirements for effective performance in cross-coupling reactions.

The steric hindrance in **GreenPhos 1**–**20** ligands was analysed using computational methods (Scheme 5B, for further details see ESI,† section 9).^20^ Topographic steric maps and corresponding percent buried volumes (% *V*_bur_)^21^ were calculated for linear [R_3_PAuCl] and tetrahedral [R_3_PNi(CO)_3_] complexes (Scheme 5B and C, see also ESI,† Fig. S19). Evaluating both linear and tetrahedral coordination environments allowed us to quantify the steric effects introduced by the ligands in different geometries, which can have significant implications in the study of organometallic reactions and catalytic processes. It is worth noting that phosphines are flexible ligands with rich structural dynamics. Hence, performing a conformational search^22^ for each phosphine was crucial, resulting in the identification of several low-energy conformations. Subsequently, density functional theory (DFT)^23^ was used to optimize the main conformers, and we selected the most stable conformation to calculate the steric maps and % *V*_bur_ (for further details see ESI,† Tables S30–S33, Fig. S19 and S20).

The analysis of % *V*_bur_ highlights that among our ligands the least hindered phosphine is **GreenPhos 2** (Scheme 5B). In contrast, the ligands that include three alkyl substituents (**GreenPhos 4**–**10**) are quite bulky, resembling Fu's ligand (*t*Bu_3_P) and related systems. Interestingly, **GreenPhos 1** and **GreenPhos 3**, as well as phosphinites derived from renewable alcohols (**GreenPhos 11**–**13**), exhibit steric hindrance comparable to that of observed for phosphines featuring three alkyl substituents (**GreenPhos 4**–**10**). Analysis of phosphinites derived from renewable phenols (**GreenPhos 14**–**20**) indicates that their estimated percent buried volumes are quite significant, falling within the range typical for sterically demanding phosphines, such as Buchwald's ligands (*e.g.*, JohnPhos, Scheme 5B).^10*f*^ A complete analysis of % *V*_bur_ values in both linear [R_3_PAuCl] and tetrahedral [R_3_PNi(CO)_3_] complexes can be found in the ESI,† section 9. Examination of catalytic pockets with topographic steric maps revealed that phosphinites derived from renewable phenols (**GreenPhos 14**–**20**) frame a heavily congested environment around the catalytic centre (Scheme 5C). Altogether, our findings indicate that the reduced catalytic efficiency of phosphinites derived from renewable phenols (**GreenPhos 14**–**20**) can be primarily attributed to a distinctive interplay between enhanced steric hindrance and diminished σ-donor abilities. We also do not exclude the possibility of cyclometallation involving phosphinites derived from renewable phenols, which could potentially lead to reduced catalyst efficiency.

**Chart 2:**
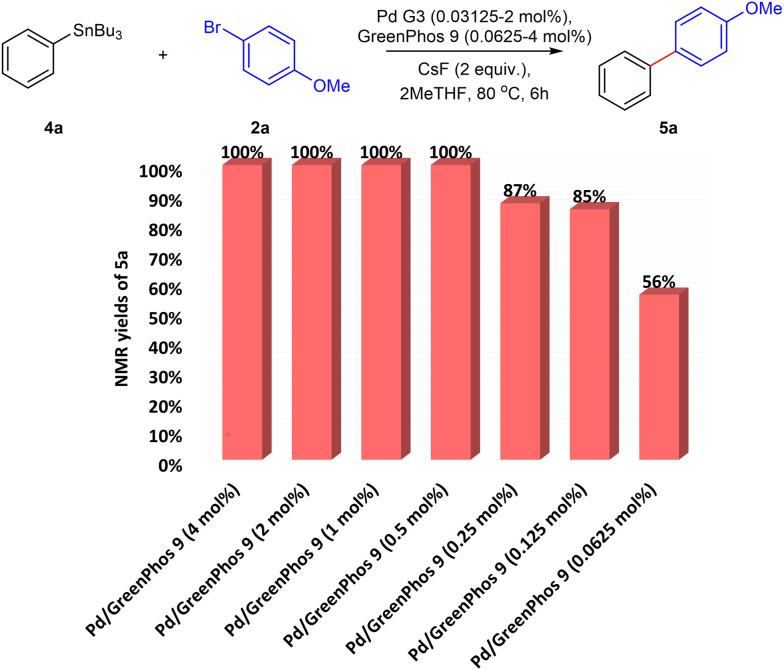
The effect of low catalyst loading on the efficiency of Stille coupling. Yields determined by ^1^H NMR spectroscopy using 1,3,5-trimethoxybenzene as an internal standard in CDCl_3_.

The high activity of catalysts ligated with our renewable ligands prompted us to explore the performance of our systems at lower catalyst loadings (Chart 2). To that end, we performed a series of studies where the catalyst loading was gradually decreased from 2 mol% to 0.0625 mol%. For our model Stille cross-coupling reaction, respectable yields can be achieved even with catalyst concentrations approaching the level of parts per million (ppm) (see also ESI,† Table S29).^24^ We believe that further improvements can be achieved by the development of preformed catalysts based on our ligands.^25^ However, this topic is beyond the scope of the present study.

The suitability of a new synthetic method for real-world applications is determined in part by its functional group tolerance. We demonstrated that our new systems have excellent functional group tolerance and are compatible with the synthesis and modification of multifunctionalized and complex systems (ESI,† Tables S10, S19 and S28, see also Fig. S11, S13 and S15).^26^ Our original approach for the Suzuki–Miyaura coupling entailed the use of a strong base and was incompatible with functional groups that are susceptible to hydrolysis. Nevertheless, we could eliminate this obstacle by replacing KOH with tetrabutylammonium fluoride (TBAF). The Stille coupling exhibited moderate compatibility with free amino groups and carboxylic acids, concurrently demonstrating pronounced tolerance towards terminal olefins, acetylenes, phenols *etc.* While the Buchwald–Hartwig amination was found to be somewhat incompatible with aldehydes and ketones, as these functional groups form corresponding Schiff bases with the amine. In contrast, secondary amines, water and terminal olefins have little to no effect on the efficiency of the reaction (ESI,† Tables S10, S19 and S28, Fig. S11, S13 and S15†). To the best of our knowledge, this type of robustness screening was not previously conducted for cross-coupling reactions. The screening included highly reactive functional groups, which are typically avoided in most published works in the field.

**Scheme 6:**
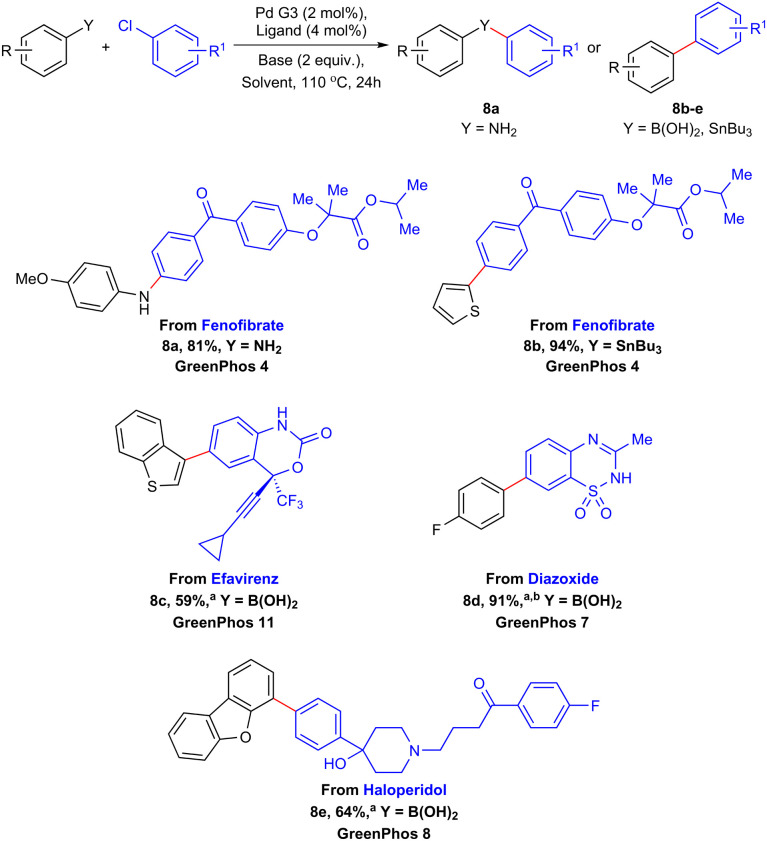
Late-stage functionalization of commercial drugs enabled by renewable ligands. Unless otherwise mentioned the yields refer to isolated products. ^a^The limiting reagent was aryl chloride. ^b^Yield determined by ^1^H NMR spectroscopy using 1,3,5-trimethoxybenzene as an internal standard in DMSO-d_6_.

The versatility and efficiency of our established protocols allowed us to undertake several late-stage functionalizations on commercial pharmaceuticals featuring halogen atoms (Scheme 6).^27^ Fenofibrate, a lipid-lowering drug, can be selectively aminated (8a, 81%) and arylated (8b, 94%) by the means of Buchwald–Hartwig amination and Stille coupling respectively. Using a similar approach, we managed to functionalize efavirenz (an antiretroviral medication, 8c, 59%), diazoxide (used to treat hypoglycemia, 8d, 91%) and haloperidol (a first-generation antipsychotic, 8e, 64%) utilizing our methodology for the Suzuki–Miyaura coupling (Scheme 6).

## Conclusions

We show that one of the most prominent ligands in homogeneous catalysis, Beller's phosphine or cataCXium® A, can be substituted with structurally analogous phosphines generated from renewable building blocks. Our new phosphine and phosphinite ligands exhibit different activity in Pd-catalysed cross-coupling reactions. Renewable phosphines possessing three alkyl substituents and phosphinites based on renewable aliphatic alcohols are particularly active ligands for the Suzuki–Miyaura coupling, Stille coupling, and Buchwald–Hartwig amination. In contrast, phosphinites based on renewable phenols are far less active, likely due to the distinctive combination of enhanced steric hindrance and reduced σ-donor ability that characterize these ligands. The methodologies we have developed exhibit exceptional versatility, enabling the implementation of renewable ligands in the late-stage functionalization of commercial pharmaceuticals. Our findings highlight the potential of using renewable building blocks in the design of value-added products, such as phosphines. We suggest that further studies to extend the range of renewable ligand families holds significant potential for unlocking unprecedented levels of catalytic activity.

## Author contributions

D. v. d. W. carried out the experiments and analysed the data. A. C. C. performed the computational study of steric hindrance in the developed ligands. N. H. provided advice to the research and manuscript. A. G. conceptualized the research, supervised the project, and wrote the main manuscript text. All authors discussed the results and reviewed the manuscript.

## Conflicts of interest

There are no conflicts to declare.

## Supplementary Material

CY-013-D3CY01375H-s001
