# Supplementary material for: Bulky, electron-rich, renewable: analogues of Beller's phosphine for cross-couplings
Source: Catal Sci Technol. 2023 Oct 30;13(23):6733–42. doi: 10.1039/d3cy01375h (PMC10680433; doi:10.1039/d3cy01375h)

Electronic Supplementary Material (ESI) for Catalysis Science & Technology.
This journal is © The Royal Society of Chemistry 2023

Supplementary Information

Bulky, Electron-Rich, Renewable: Analogues of Beller's Phosphine for Cross-Couplings

Danielle van der Westhuizen, [a] Abril C. Castro, [b] Nilay Hazari, [c] and Ashot Gevorgyan *[a]

[a] Department of Chemistry, UiT The Arctic University of Norway, 9037 Tromsø, Norway.
[b] Hylleraas Centre for Quantum Molecular Sciences, Department of Chemistry, University of Oslo, 0315 Oslo, Norway.
[c] Department of Chemistry, Yale University, New Haven, Connecticut 06520, United States.

***E-mail: gevorgyan.ashot@uit.no**

Contents

1. EXPERIMENTAL PART: GENERAL CONSIDERATIONS 3

2. PREPARATION OF STARTING MATERIALS 4

2.1. HYDROGENATION OF UNSATURATED TERPENES 4
2.2. SYNTHESIS OF IODOALKANES 5
2.3. SYNTHESIS OF *TERT*-ALKYL ESTERS 5

3. PREPARATION OF RENEWABLE LIGANDS 7

3.1. SYNTHESIS OF DI-*TERT*-ALKYLPHOSPHONIUM SALTS (GREENPHOS 1-3) 7
3.2. SYNTHESIS OF TRIALKYLATED PHOSPHONIUM SALTS (GREENPHOS 4-10) 9
3.3. SYNTHESIS OF RENEWABLE PHOSPHINITES (GREENPHOS 11-20) 13

4. ESTIMATION OF THE ELECTRON-DONATING ABILITIES OF PREPARED LIGANDS 18

5. THE SCREENING OF CATALYTIC ACTIVITY OF PREPARED LIGANDS 20

5.1. SUZUKI-MIYAURA CROSS-COUPLING REACTION 20
5.1.1. Optimization of the reaction conditions 20
5.1.2. Screening of phosphonium salts for Suzuki-Miyaura coupling 32
5.1.3. Screening of phosphinites for Suzuki-Miyaura coupling 34
5.1.4. Optimization of reaction conditions for chlorobenzene 35
5.1.5. Estimation of the functional group tolerance of Suzuki-Miyaura coupling 37
5.2. STILLE CROSS-COUPLING REACTION 40
5.2.1. Optimization of the reaction conditions 40
5.2.2. Screening of phosphonium salts for Stille coupling 44
5.2.3. Screening of phosphinites for Stille coupling 46
5.2.4. Optimization of Stille coupling for 4-chlorotoluene 48
5.2.5. Estimation of the functional group tolerance of Stille coupling 50
5.3. BUCHWALD-HARTWIG AMINATION 53
5.3.1. Optimization of the reaction conditions 53

5.3.2. Screening of phosphonium salts for Buchwald-Hartwig amination ... 57
5.3.3. Screening of phosphinites for Buchwald-Hartwig amination ... 59
5.3.4. Optimization of Buchwald-Hartwig amination for 4-chlorotoluene ... 61
5.3.5. Estimation of the functional group tolerance of Buchwald-Hartwig amination ... 63

6. STILLE COUPLING WITH LOW CATALYST LOADING ... 66

7. SELECTED EXAMPLES OF ISOLATED PRODUCTS ... 68

7.1.1. Isolated products for Suzuki-Miyaura coupling ... 68
7.1.2. Isolated products for Stille coupling ... 70
7.1.3. Isolated products for Buchwald-Hartwig amination ... 72

8. LATE-STAGE FUNCTIONALIZATION OF PHARMACEUTICALS ... 74

8.1. FUNCTIONALIZATION OF FENOFIBRATE BY BUCHWALD-HARTWIG AMINATION ... 74
8.2. ARYLATION OF FENOFIBRATE BY STILLE COUPLING ... 75
8.3. ARYLATION OF EFAVIRENZ BY SUZUKI-MIYAURA COUPLING ... 76
8.4. ARYLATION OF DIAZOXIDE BY SUZUKI-MIYAURA COUPLING ... 77
8.5. ARYLATION OF HALOPERIDOL BY SUZUKI-MIYAURA COUPLING ... 78

9. COMPUTATIONAL METHODS ... 79

9.1. CONFORMATIONAL SEARCH OF LOWEST-ENERGY CONFORMERS ... 79
9.2. STRUCTURE OPTIMIZATIONS ... 79
9.3. CALCULATION OF STERIC MAPS ... 80

10. COPIES OF SPECTRA ... 91

1. Experimental part: general considerations

Materials. All synthetic procedures sensitive to air and moisture were conducted under inert atmosphere (either under nitrogen or argon gas) using standard Schlenk line or vacuum line techniques. Air sensitive solids were stored and handled in an argon-filled glovebox. Solvents were degassed and stored over activated 3 Å or 4 Å molecular sieves, depending on the solvent. All other commercially available starting materials were used without further purification. Normal flash column chromatography was performed with Merck silica gel 60 (230-400 mesh). Thin layer chromatography was carried out using Merck TLC Silica gel 60 F_{254} and visualized by short-wavelength ultraviolet light or by treatment with potassium permanganate ($KMnO_4$) stain. Automated flash chromatography was carried out on Teledyne ISCO CombiFlash® EZ Prep using Teledyne ISCO RediSep Gold® high performance silica chromatography columns (4-24g).

Instrumentation. Nuclear magnetic resonance (NMR) spectra were recorded at 20 °C on a Bruker Avance Spectrometer operating at 400.18 MHz for ^{1}H, 100.64 MHz for ^{13}C, 162 MHz for ^{31}P, 376.51 MHz for ^{19}F and 128.39 MHz for ^{11}B. All ^{1}H NMR spectra are reported in parts per million (δ, ppm) downfield of TMS and were measured relative to the signal for residual $CHCl_3$ (7.26 ppm). Proton coupling constants (*J*) are given in Hertz (Hz) and the spectral coupling patterns are abbreviated as follows: s-singlet; d-doublet; t-triplet; q-quartet; m-multiplet; br s-broad signal. All ^{13}C NMR spectra are reported in ppm (δ) relative to residual $CDCl_3$ (77.20 ppm) and were obtained with ^{1}H decoupling. High-resolution mass spectra (HRMS) were recorded from methanol solutions on a Thermo Scientific Orbitrap Exploris 120 either in negative or in positive electrospray ionization (ESI) mode by direct injection at a flow rate for 10 µL/min. Melting points were measured using a Stuart SMP50 automatic melting point apparatus. Infrared (IR) measurements were carried out using an Agilent Technologies Cary 630 FTIR instrument, and the signals are reported as follows: s-strong intensity; m-medium intensity; w-weak intensity.

2. Preparation of starting materials

2.1. Hydrogenation of unsaturated terpenes

General procedure for the hydrogenation of terpenes[1]

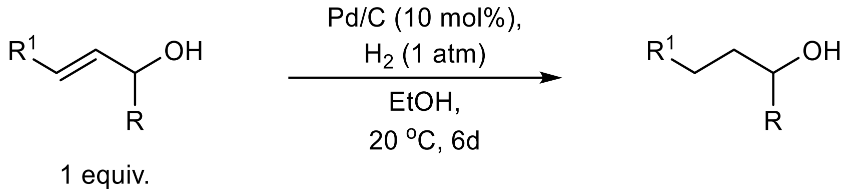

Dry Pd/C (10 mol%) was added to a one-neck oven-dried round bottom flask inside of an argon-filled glovebox. The flask was closed with a rubber septum and removed from the glovebox. The flask was evacuated and then fitted with a H_2 balloon. Anhydrous, degassed ethanol (100 mL) was added followed by the corresponding olefin (1 equiv., 0.130 mol). In cases where the olefin was a solid, the round bottom flask was charged with the solid prior to the introduction of the flask to the glovebox. The resulting reaction mixture was left to stir for 6 days at 20 °C. The H_2 balloon was changed regularly over the course of the reaction to ensure a constant pressure of H_2. Afterwards, the reaction mixture was filtered through celite and the celite was washed with several portions of methanol (400 mL in total). The mother liquor was evaporated to afford the pure product.

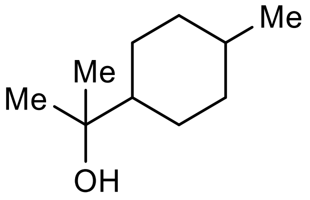

2-(4-Methylcyclohexyl)propan-2-ol.[2] 2-(4-Methylcyclohexyl)propan-2-ol was obtained from (-)-α-terpineol (2.00 g, 12.97 mmol) as a colorless liquid (2.02 g, 99%). **^{1}H NMR (400 MHz, CDCl$_3$):** δ = 0.90 (d, $^3J_{HH}$ = 6.5 Hz, 2H), 0.92-0.95 (m, 1H), 0.97 (d, $^3J_{HH}$ = 7.2 Hz, 1H), 1.11-1.01 (m, 2H), 1.19-1.18 (m, 6H), 1.24-1.36 (m, 3H), 1.52-1.59 (m, 2H), 1.76-1.84 (m, 3H). **^{13}C NMR (101 MHz, CDCl$_3$):** δ = 17.6, 21.6, 22.8, 27.1, 27.2, 27.6, 32.2, 32.9, 35.5, 49.0, 49.7, 73.1.

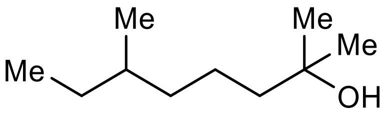

2,6-Dimethyloctan-2-ol (tetrahydromyrcenol).[3] 2,6-Dimethyloctan-2-ol was obtained from dihydromyrcenol (20.0 g, 0.130 mol) as a colorless liquid (15.8 g, 77%). **^{1}H NMR (400 MHz, CDCl$_3$):** δ = 0.83-0.87 (m, 6H, 2xMe), 1.06-1.17 (m, 2H, CH$_2$), 1.21 (s, 6H, 2xMe), 1.23-1.48 (m, 8H, CH$_2$, CH). **^{13}C NMR (101 MHz, CDCl$_3$):** δ = 11.5, 19.3, 22.0, 29.3, 29.4, 29.6, 34.5, 37.3, 44.4, 71.2.

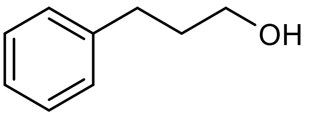

3-Phenylpropan-1-ol.[4] 3-Phenylpropan-1-ol was obtained from cinnamyl alcohol (15.0 g, 0.112 mol) as a colorless liquid (14.3 g, 94%). **^{1}H NMR (400 MHz, CDCl$_3$):** δ = 1.44-1.45 (m, 1H, OH), 1.87-1.94 (m, 2H, CH$_2$), 2.72 (dd, *J* = 8.7, 6.8 Hz, 2H, CH$_2$), 3.68 (t, *J* = 6.4 Hz, 2H, CH$_2$), 7.18-7.22 (m, 3H, Ar), 7.28-7.32 (m, 2H, Ar). **^{13}C NMR (101 MHz, CDCl$_3$):** δ = 32.2, 34.4, 62.4, 126.0, 128.5, 128.6, 142.0.

---

[1] P. Müller and J.-C. Rossier, *J. Chem. Soc., Perkin Trans. 2*, 2000, 2232-2237.
[2] W. Zhu, X. Liu, Y. Wang, Y. Tong and Y. Hu, *Eur. J. Med. Chem.*, 2018, **143**, 419-425.
[3] D. P. Lubov, M. V. Shashkov, A. A. Nefedov and K. P. Bryliakov, *Org. Lett.*, 2023, **25**, 1359-1363.
[4] V. Goyal, T. Bhatt, C. Dewangan, A. Narani, G. Naik, E. Balaraman, K. Natte and R. V. Jagadeesh, *J. Org. Chem.*, 2023, **88**, 2245-2259.

2.2. Synthesis of iodoalkanes

General procedure for the iodination of 3-phenyl-1-propanol[5]

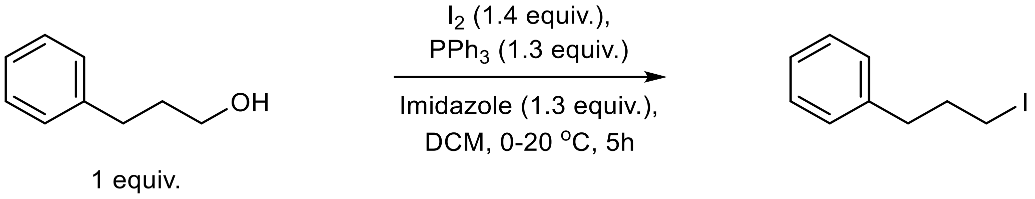

Iodine (1.4 equiv.), PPh_3 (1.3 equiv.) and imidazole (1.3 equiv.) were sequentially added to an oven dried 250 mL round bottom flask filled with DCM (300 mL). The mixture was stirred for 15 minutes at 0 °C, whereupon 3-phenyl-1-propanol (1 equiv., 36.7 mmol) was added. The reaction was stirred at 20 °C for 5 hours and filtered through a short pad of silica gel with several portions of pentane (600 mL in total). The filtrate was concentrated under reduced pressure to yield (3-Iodopropyl)benzene.

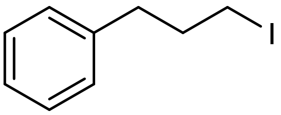

(3-Iodopropyl)benzene.[5] (3-Iodopropyl)benzene was obtained from 3-phenylpropan-1-ol (5.0 g, 0.0367 mol) as a yellowish liquid (8.2 g, 91%). **^{1}H NMR (400 MHz, $CDCl_3$):** δ = 2.14 (p, *J* = 6.9 Hz, 2H, CH_2), 2.74 (t, *J* = 7.3 Hz, 2H, CH_2), 3.18 (t, *J* = 6.8 Hz, 2H, CH_2), 7.19-7.24 (m, 3H, Ar), 7.28-7.32 (m, 2H, Ar). **^{13}C NMR (101 MHz, $CDCl_3$):** δ = 6.5, 35.1, 36.4, 126.4, 128.6, 128.7, 140.6.

2.3. Synthesis of *tert*-alkyl esters

General procedure for the acylation of tertiary alcohols[6]

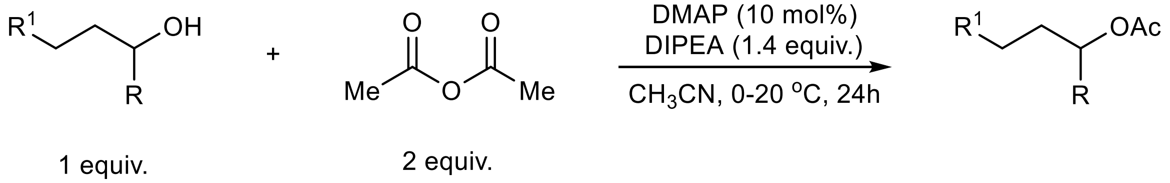

DMAP (10 mol%) was added to an oven-dried one-neck round bottom flask and the flask was sealed with a rubber septum, evacuated, and equipped with an argon-filled balloon. Subsequently, anhydrous acetonitrile (50 mL), the corresponding alcohol (1 equiv., 64 mmol) and DIPEA (1.4 equiv.) were added. The reaction mixture was cooled to 0 °C and acetic anhydride (2 equiv.) was added dropwise. After the addition was complete, the reaction flask was removed from the ice bath and continued to stir at 20 °C for 24 hours. Upon completion, the reaction mixture was poured into distilled water (500 mL) and stirred overnight. Thereafter, the organic phase of the reaction mixture was extracted with diethyl ether (3 x 150 mL). The diethyl ether fractions were combined, then 100 mL 6 M aqueous HCl was added, and the crude reaction mixture was stirred overnight. After the organic phase was separated from the acidic aqueous phase, the organic phase was subsequently washed with 6 M aqueous HCl (2 x 100 mL), followed by washing with a saturated aqueous solution of $NaHCO_3$ (2 x 100 mL) and distilled

[5] A. Horn and P. H. Dussault, *RSC Adv.*, 2020, **10**, 44408-44429.
[6] J. M. Alvarez-Calero, Z. D. Jorge and G. M. Massanet, *Org. Lett.*, 2016, **18**, 6344-6347.

water (1 x 100 mL). The resulting organic phase was dried with anhydrous Na_2SO_4, filtered and the solvent was evaporated to yield the pure product.

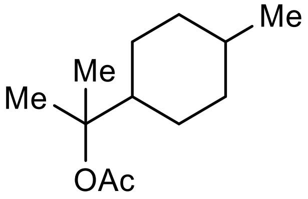

2-(4-Methylcyclohexyl)propan-2-yl acetate.[7] 2-(4-Methylcyclohexyl)propan-2-yl acetate was obtained from 2-(4-methylcyclohexyl)propan-2-ol (10.0 g, 63.9 mmol), as a yellow liquid (10.7 g, 84%). **^{1}H NMR (400 MHz, $CDCl_3$):** δ = 0.86-1.11 (m, 6H), 1.22-1.32 (m, 2H), 1.39-1.54 (m, 7H), 1.67-1.73 (m, 3H), 1.81-1.88 (m, 1H), 1.96 (s, 3H). **^{13}C NMR (101 MHz, $CDCl_3$):** δ = 21.2, 22.7, 23.6, 27.3, 32.0, 35.3, 46.0, 85.5, 170.6. **IR (ATR, cm^{-1}):** *ν* = 2938 (m), 2868 (w), 1731 (s), 1451 (m), 1367 (s), 1256 (s), 1167 (m), 1140 (m), 1116 (s), 1018 (m), 940 (w), 788 (w), 760 (w).

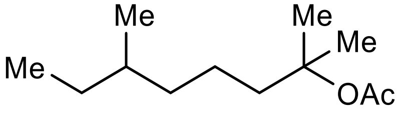

2,6-Dimethyloctan-2-yl acetate.[8] 2,6-Dimethyloctan-2-yl acetate was obtained from 2,6-dimethyloctan-2-ol (10.0 g, 63.2 mmol) as a yellow liquid (10.1 g, 80%). **^{1}H NMR (400 MHz, $CDCl_3$):** δ = 0.84-0.87 (m, 6H), 1.07-1.14 (m, 2H), 1.24-1.36 (m, 5H), 1.42 (s, 6H), 1.67-1.72 (m, 2H), 1.96 (s, 3H). **^{13}C NMR (101 MHz, $CDCl_3$):** δ = 11.5, 19.3, 21.5, 22.6, 26.2, 29.7, 34.4, 37.0, 41.1, 82.7, 170.7. **IR (ATR, cm^{-1}):** *ν* = 2958 (w), 2934 (w), 2874 (w), 1731 (s), 1460 (m), 1367 (s), 1254 (s), 1205 (s), 1154 (m), 1115 (m), 1087 (w), 1018 (m), 945 (w), 837 (w), 775 (w), 762 (w).

[7] C. Colonge, *Bull. Soc. Chim. Fr.*, 1959, 1505-1508.
[8] G. Ohloff, J. Seibl and E. Kovats, *Justus Liebigs Ann. Chem.*, 1964, **675**, 83-101.

3. Preparation of renewable ligands

3.1. Synthesis of di-*tert*-alkylphosphonium salts (GreenPhos 1-3)

General procedure for the synthesis of di-*tert*-alkylphosphonium salts.[9]

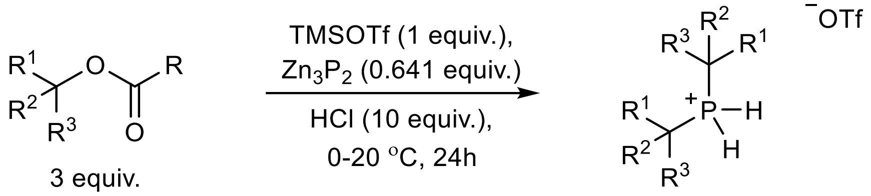

The synthesis of di-*tert*-alkylphosphonium salts were conducted in two-chamber CO-ware reactors (100 mL total volume).[10] Each chamber was sealed with a compatible screwcap, PTFE stabilizing disc and single-use silico/PTFE septum. Inside of an argon-filled glovebox, zinc phosphide (0.641 equiv., to produce 1 equiv. PH_3) was added to chamber **A** and the chamber was closed. To chamber **B**, the appropriate degassed *tert*-alkyl ester (3 equiv., 25 mmol) was added, and the chamber was closed. The reactor was removed from the glovebox and cooled down to 0 °C. Then, trimethylsilyl trifluoromethanesulfonate (1 equiv.) was added to the *tert*-alkyl ester in chamber **B**. Degassed hydrochloric acid (11.65 M, 10 equiv.) was added to chamber **A** in one portion using a Luer lock syringe. **CAUTION:** gas pressure within the reactor increases immediately. The plunger of the syringe used to add hydrochloric acid should be held down after the acid has been added and while the syringe is removed from the reactor. The reaction vessel was removed from the ice bath and stirred at 20 °C for 24h. Afterwards, under a well operating fume hood chamber **B** was opened to air and a small amount of diethyl ether (≤ 10 mL) was added to the chamber **B** and all the contents of the chamber **B** was transferred to a separate flask containing heptane (100 mL). The resulting precipitate was filtered, washed with pentane, and dried in vacuo. Precipitation was not always immediate; in such cases the reaction mixture was stirred in heptane overnight.

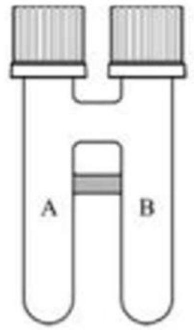

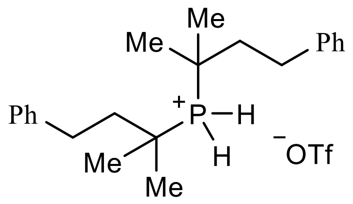

Di-(2-methyl-4-phenylbutan-2-yl)phosphonium trifluoromethanesulfonate, GreenPhos 1. Di-(2-methyl-4-phenylbutan-2-yl)phosphonium trifluoromethanesulfonate was obtained from papaya isobutyrate (6.00 g, 25.60 mmol) as a white solid (3.89 g, 96%). **m.p.** = 74-77 °C. **^{1}H NMR (400 MHz, CDCl$_3$):** δ = 1.58 (d, $^3J_{PH}$ = 18.4 Hz, 12H, Me), 2.08 (ddd, $^3J_{PH}$ = 17.2 Hz, $^3J_{HH}$ = 8.5, 4.8 Hz, 4H, CH_2), 2.71 (dd, $^3J_{HH}$ = 8.1, 4.1 Hz, 2H, CH_2), 2.72 (dd, $^3J_{HH}$ = 8.0, 4.5 Hz, 2H, CH_2), 6.54 (d, $^1J_{PH}$ = 476.0 Hz, 2H, PH_2), 7.16-7.19 (m, 4H, Ar), 7.20-7.23 (m, 2H, Ar), 7.26-7.30 (m, 4H, Ar). **^{13}C NMR (101 MHz, CDCl$_3$):** δ = 25.0 (Me), 29.7 (d, $^3J_{PC}$ = 8.1 Hz, CH_2), 35.3 (d, $^1J_{PC}$ = 33.2 Hz, C_q), 41.6 (CH_2), 120.7 (q, $^1J_{CF}$ = 320.1 Hz, CF_3), 125.5 (Ar), 126.7 (Ar), 128.5 (Ar), 128.9 (Ar), 139.8 (Ar-C_q). **^{31}P NMR (162 MHz, CDCl$_3$):** δ = 13.67 (t, $^1J_{PH}$ = 475.7 Hz). **^{19}F NMR (377 MHz, CDCl$_3$):** δ =

[9] T. Barber, S. P. Argent and L. T. Ball, *ACS Catal.*, 2020, **10**, 5454-5461.

[10] COware gas reactor reaction scale of 1.0-5.0 mmol (total volume 100 mL) | Sigma-Aldrich (sigmaaldrich.com), accessed at 09.06.2023.

-78.26. **HRMS:** [M-OTf]$^+$ calcd for $C_{22}H_{32}P$: 327.2236; found: 327.2237. **IR (ATR, cm^{-1}):** v = 2964 (w), 2425 (w), 2396 (w), 1604 (w), 1499 (w), 1458 (m), 1374 (w), 1274 (s), 1249 (s), 1221 (s), 1153 (s), 1027 (s), 979 (w), 935 (m), 746 (s), 702 (s).

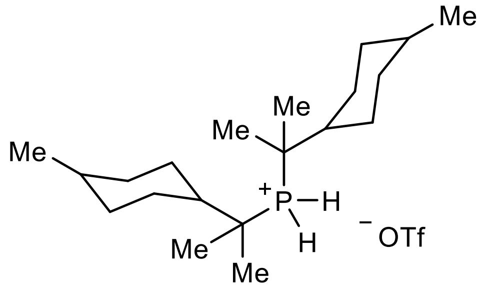

Di-(2-methyl-2-(4-methylcyclohexyl)propan-2-yl)phosphonium trifluoromethanesulfonate, GreenPhos 2. Di-(2-methyl-2-(4-methylcyclohexyl)propan-2-yl)phosphonium trifluoromethanesulfonate was obtained from 2-(4-methylcyclohexyl)propan-2-yl acetate (5.00 g, 25.21 mmol) as a white solid (1.68 g, 44%). **m.p.** = 155-161 °C. **^{1}H NMR (400 MHz, CDCl$_3$):** δ = 0.90 (d, $^3J_{HH}$ = 6.6 Hz, 6H, Me), 0.93-1.00 (m, 4H, CH$_2$), 1.23-1.41 (m, 6H, CH$_2$), 1.53 (d, $^3J_{PH}$ = 19.1 Hz, 12H, Me), 1.61-1.70 (m, 2H, CH$_2$), 1.78-1.87 (m, 8H, CH$_2$), 6.41 (d, $^1J_{PH}$ = 470.8 Hz, 2H, PH$_2$). **^{13}C NMR (101 MHz, CDCl$_3$):** δ = 22.3 (Me), 23.6 (Me), 27.3 (d, J_{CP} = 6.25 Hz, CH$_2$), 32.2 (CH$_2$), 34.8 (CH$_2$), 40.3 (d, $^1J_{CP}$ = 30.9 Hz, C$_q$), 45.1 (CH$_2$). **^{31}P NMR (162 MHz, CDCl$_3$):** δ = 9.24 (t, $^1J_{PH}$ = 478.0 Hz). **^{19}F NMR (377 MHz, CDCl$_3$):** δ = -78.36. **HRMS:** [M-OTf]$^+$ calcd for $C_{20}H_{40}P$: 311.2862; found: 311.2863. **IR (ATR, cm^{-1}):** v = 2930 (w), 2871 (w), 2851 (w), 2418 (w), 1478 (w), 1453 (w), 1403 (w), 1381 (w), 1256 (s), 1225 (m), 1159 (s), 1029 (s), 987 (w), 969 (w), 945 (w), 926 (w), 760 (w), 692 (w).

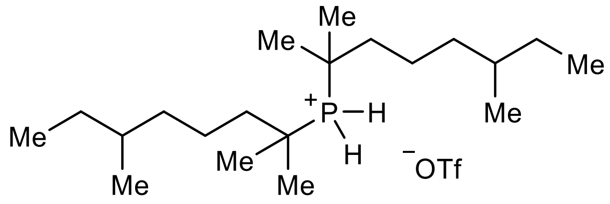

Di-(2,6-dimethyloctan-2-yl)phosphonium trifluoromethanesulfonate, GreenPhos 3. Di-(2,6-dimethyloctan-2-yl)phosphonium trifluoromethanesulfonate was obtained from 2,6-dimethyloctan-2-yl acetate (5.00 g, 24.96 mmol) as a white solid (2.43 g, 63%). **m.p.** = 84-87 °C. **^{1}H NMR (400 MHz, CDCl$_3$):** δ = 0.85-0.88 (m, 12H, Me), 1.11-1.19 (m, 4H, CH$_2$), 1.28-1.48 (m, 10H), 1.56 (d, $^3J_{PH}$ = 18.6 Hz, 12H, Me), 1.72-1.81 (m, 4H, CH$_2$), 6.38 (d, $^1J_{HH}$ = 471.7 Hz, 2H, PH$_2$). **^{13}C NMR (101 MHz, CDCl$_3$):** δ = 11.5 (Me), 19.2 (Me), 21.3 (d, J_{CP} = 7.9 Hz), 25.3 (Me), 29.5, 34.3, 35.6 (d, $^1J_{CP}$ = 33.0 Hz, C$_q$), 36.7, 40.2. **^{31}P NMR (162 MHz, CDCl$_3$):** δ = 16.53 (t, $^1J_{PH}$ = 488.1 Hz). **^{19}F NMR (377 MHz, CDCl$_3$):** δ = -78.41. **HRMS:** [M-OTf]$^+$ calcd for $C_{20}H_{44}P$: 315.3175; found: 315.3176. **IR (ATR, cm^{-1}):** v = 2963 (w), 2935 (w), 2878 (w), 2408 (w), 1466 (w), 1405 (w), 1382 (w), 1260 (s), 1227 (m), 1155 (m), 1031 (s), 924 (w), 759 (w).

3.2. Synthesis of trialkylated phosphonium salts (GreenPhos 4-10)

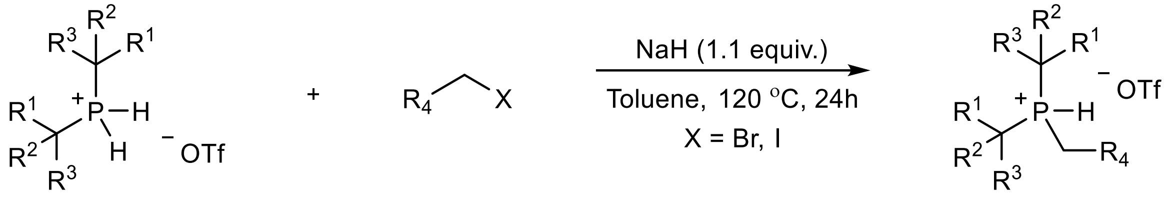

1 equiv. 6 equiv.

Method A.[11] Inside of an argon-filled glovebox an oven dried 50 mL pressure tube was charged with starting phosphonium salt (4 mmol, 1 equiv.), degassed dry toluene (16 mL) and NaH (1.1 equiv.). The resulting mixture was allowed to stirre in the glovebox at 20 °C for 1h. This was followed by addition of the degassed alkyl iodide (6 equiv.). The pressure tube was sealed with the corresponding stopper and removed from the glovebox. The resulting mixture was stirred at 120 °C for 24h. Thereafter, the reaction vessel was cooled down to room temperature, fitted with an argon balloon and degassed heptane (at least twice the volume of toluene) was added. The reaction mixture was stirred for at least 30 minutes, after which the vessel was opened to air and the stirring ceased. Once the reaction mixture had settled (after 10-30 minutes) the solvent was removed by pipette as the product precipitated as sticky oil droplets against the glass wall of the reaction flask. After the precipitate was washed once more with heptane, the precipitate was dissolved in DCM, transferred into a 250 mL separating funnel, diluted with DCM (80 mL) and was washed with water (twice 15 mL). The resulting DCM solution was evaporated to dryness to give the product.

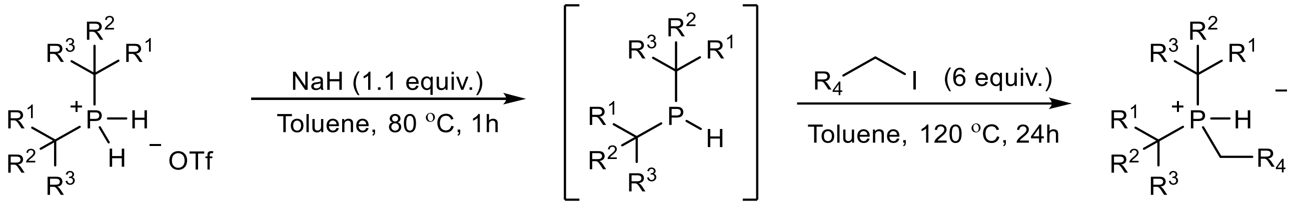

1 equiv.

Method B.[11] Inside of an argon-filled glovebox the appropriate di-*tert*-alkylphosphonium salt (1 equiv., 4 mmol) and sodium hydride (1.1 equiv.) were added to an oven-dried 100 mL Schlenk flask. The Schlenk flask was closed with a septum fitted with a cannula filter (connected on the other side to an oven-dried 100 mL round bottom collecting flask) and the reaction setup was removed from the glovebox. The Schlenk flask was fitted with an argon balloon and dry, degassed toluene (16 mL) was added to the flask. Afterwards, the reaction was heated to 80 °C for 1 hour and then cooled down to room temperature while fitted to an argon balloon. Once at room temperature, the balloon was removed, and the cannula filter was lowered into the reaction mixture. The mother liquor was transferred *via* the cannula to the collecting flask (fitted with a degas needle) under the flow of argon. Once the transfer was complete, the mother liquor was transferred *via* a syringe to a 50 mL pressure tube containing the corresponding alkyl iodide (6 equiv.) and sealed with the compatible screwcap, septum, and PTFE stabilizer. After the addition was complete, the reaction vial was heated to 120 °C

[11] A. Tewari, M. Hein, A. Zapf, M. Beller, *Synthesis*, 2004, **6**, 935-941.

for 24 hours. Thereafter, the reaction work-up followed the same procedure as outlined in the Method A.

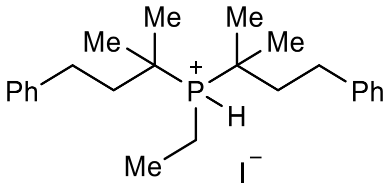

Ethylbis(2-methyl-4-phenylbutan-2-yl)phosphonium iodide, GreenPhos 4. Ethylbis(2-methyl-4-phenylbutan-2-yl)phosphonium iodide was obtained from di-(2-methyl-4-phenylbutan-2-yl)phosphonium trifluoromethanesulfonate (GreenPhos 1, 2.00 g, 4.197 mmol) using the method B. The product was isolated as a white solid (1.96 g, 97%). **m.p.** = 119-122 °C. **^{1}H NMR (400 MHz, CDCl$_3$):** δ = 1.60-1.71 (m, 15H, 5xMe), 2.09-2.16 (m, 4H, CH$_2$), 2.27-2.37 (m, 2H, CH$_2$), 2.77-2.82 (m, 4H, 2xCH$_2$), 7.17-7.24 (m, 6H, Ar), 7.27-7.31 (m, 4H, Ar), 8.88 (dt, $^3J_{HP}$ = 479.04 Hz, $^3J_{HH}$ = 3.99 Hz, 1H, PH). **^{13}C NMR (101 MHz, CDCl$_3$):** δ = 9.4 (d, $^1J_{CP}$ = 40.4 Hz, CH$_2$), 11.0 (d, $^2J_{CP}$ = 6.12 Hz, Me), 24.7 (Me), 25.0 (Me), 29.5 (d, $^4J_{CP}$ = 8.20 Hz), 37.6 (d, $^1J_{CP}$ = 31.6 Hz, C$_q$), 41.0 (CH$_2$), 126.8 (Ar), 128.5 (Ar), 129.0 (Ar), 139.8 (Ar-C$_q$). **^{31}P NMR (162 MHz, CDCl$_3$):** δ = 31.32 (d, $^1J_{PH}$ = 480.0 Hz). **HRMS:** [M-I]$^+$ calcd for $C_{24}H_{36}P$: 355.2549; found: 355.2549. **IR (ATR, cm^{-1}):** v = 2950 (w), 2883 (w), 2287 (w), 1602 (w), 1493 (m), 1475 (m), 1459 (m), 1380 (w), 1282 (w), 1216 (w), 1143 (w), 1056 (w), 1032 (m), 922 (w), 913 (w), 903 (w), 769 (m), 755 (s), 719 (m), 705 (s).

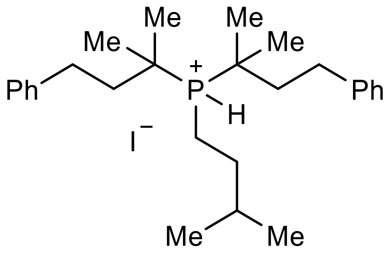

Isopentylbis(2-methyl)-4-phenylbutan-2-yl)phosphonium iodide, GreenPhos 5. Isopentylbis(2-methyl)-4-phenylbutan-2-yl)phosphonium iodide was obtained from di-(2-methyl-4-phenylbutan-2-yl)phosphonium trifluoromethanesulfonate (GreenPhos 1, 0.25 g, 0.525 mmol) using the method B. The product was isolated as a viscous white oil (0.21 g, 76%). **^{1}H NMR (400 MHz, CDCl$_3$):** δ = 0.96 (d, $^3J_{HH}$ = 6.6 Hz, 6H, Me), 1.67 (dd, $^3J_{HP}$ = 16.8 Hz, $^4J_{HH}$ = 8.9 Hz, 12H, Me), 1.60-1.78 (m, 1H, CH), 1.81-1.89 (m, 2H, CH$_2$), 2.06-2.22 (m, 6H, CH$_2$), 2.72-2.86 (m, 4H, CH$_2$), 7.17-7.24 (m, 6H, Ar), 7.26-7.30 (m, 4H, Ar), 8.54 (dt, $^1J_{HP}$ = 477.9 Hz, $^3J_{HH}$ = 4.0 Hz, PH). **^{13}C NMR (101 MHz, CDCl$_3$):** δ = 13.3 (d, $^1J_{CP}$ = 38.9, CH$_2$), 21.8 (Me), 24.6 (Me), 24.9 (Me), 29.4 (d, $^4J_{CP}$ = 8.1 Hz, CH$_2$), 29.7 (d, $^4J_{CP}$ = 12.7 Hz, CH), 35.0 (d, $^3J_{CP}$ = 5.7 Hz, CH$_2$), 37.6 (d, $^1J_{CP}$ = 31.7, C$_q$), 40.9 (CH$_2$), 126.7 (Ar), 128.4 (Ar), 128.9 (Ar), 139.9 (Ar-C$_q$). **^{31}P NMR (162 MHz, CDCl$_3$):** δ = 29.34 (d, $^1J_{PH}$ = 479.1 Hz). **HRMS:** [M-I]$^+$ calcd for $C_{27}H_{42}P$: 397.3019; found: 397.3019. **IR (ATR, cm^{-1}):** v = 3027 (w), 2953 (m), 2934 (m), 2868 (m), 2267 (m), 1602 (w), 1498 (w), 1467 (m), 1454 (m), 1397 (w), 1376 (w), 1263 (w), 1223 (w), 1146 (w), 1074 (w), 1032 (m), 912 (w), 748 (s), 700 (s).

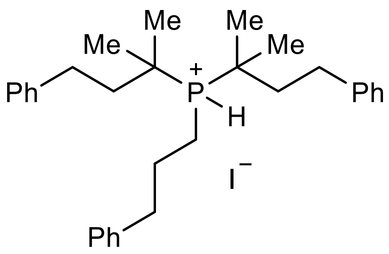

(3-Phenylpropyl)bis(2-methyl-4-phenylbutan-2-yl)phosphonium iodide, GreenPhos 6. (3-Phenylpropyl)bis(2-methyl-4-phenylbutan-2-yl)phosphonium iodide was obtained from di-(2-methyl-4-phenylbutan-2-yl)phosphonium trifluoromethanesulfonate (GreenPhos 1, 0.25 g, 0.525 mmol) using the method B. The product was isolated as a white solid (0.23 g, 77%). **m.p.** = 122-126 °C. **^{1}H NMR (400 MHz, CDCl$_3$):** δ = 1.57 (dd, $^3J_{HP}$ = 16.9 Hz, $^4J_{HH}$ = 7.8 Hz, 12H, Me), 1.97-2.04 (m, 4H, CH$_2$), 2.06-2.11

(m, 2H, CH_2), 2.31-2.40 (m, 2H, CH_2), 2.66-2.76 (m, 4H, CH_2), 2.82 (t, $^3J_{HH}$ = 6.7 Hz, 2H, CH_2), 7.13-7.25 (m, 12H, Ar), 7.27-7.30 (m, 3H, Ar), 8.94 (d, $^1J_{HP}$ = 479.0 Hz, 1H, PH). **^{13}C NMR (101 MHz, CDCl$_3$):** δ = 13.7 (d, $^1J_{CP}$ = 38.9 Hz, CH_2), 24.3 (Me), 24.6 (Me), 28.0 (d, J = 5.1 Hz, CH_2), 29.3 (d, J = 8.2 Hz, CH_2), 36.3 (d, J = 13.2 Hz, CH_2), 37.4 (d, $^1J_{CP}$ = 31.6 Hz, C_q), 40.6 (CH_2), 126.6 (Ar), 126.8 (Ar), 128.3 (Ar), 128.7 (Ar), 128.8 (Ar), 128.9 (Ar), 138.9 (Ar-C_q), 139.8 (Ar-C_q). **^{31}P NMR (162 MHz, CDCl$_3$):** δ = 26.6 (t, $^1J_{PH}$ = 477.9 Hz). **HRMS**: [M-I]$^+$ calcd for $C_{31}H_{42}P$: 445.3019; found: 445.3018. **IR (ATR, cm^{-1}):** v = 3068 (w), 3032 (w), 3037 (w), 2965 (w), 2931 (w), 2284 (w), 1604 (w), 1498 (w), 1457 (m), 1397 (w), 1377 (w), 1073 (w), 1031 (w), 911 (w), 898 (w), 879 (w), 750 (s), 744 (s), 704 (s), 698 (s).

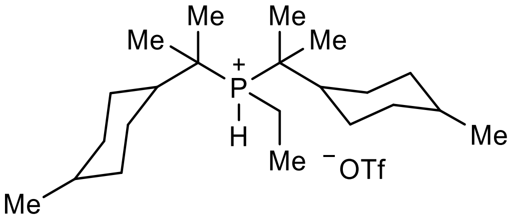

Ethylbis(2-methyl-2-(4-methylcyclohexyl)propan-2-yl)phosphonium trifluoromethanesulfonate, GreenPhos 7. Ethylbis(2-methyl-2-(4-methylcyclohexyl)propan-2-yl)phosphonium trifluoromethanesulfonate was obtained from di-(2-methyl-2-(4-methylcyclohexyl)propan-2-yl)phosphonium trifluoromethanesulfonate (GreenPhos 2, 0.50 g, 1.086 mmol) using the method A. The product was isolated as a white solid (0.17 g, 33%) after purification with flash chromatography (eluent 15% MeOH in EtOAc). **m.p.** = 133-136 °C. **^{1}H NMR (400 MHz, CDCl$_3$):** δ = 0.86-0.98 (m, 10H), 1.11-1.39 (m, 7H), 1.40-1.57 (m, 14H), 1.60-1.93 (m, 10H), 2.24-2.33 (m, 2H), 6.43 (dt, $^1J_{PH}$ = 465.3 Hz, $^3J_{HH}$ = 4.1 Hz, 1H, PH). **^{13}C NMR (101 MHz, CDCl$_3$):** δ = 10.6 (d, J = 40.5 Hz, CH_2), 12.6 (d, J = 6.6 Hz), 21.7 (Me), 22.3 (Me), 23.1 (Me), 27.1 (d, J = 6.1 Hz), 27.9 (d, J = 5.8 Hz), 32.4, 34.9 (d, J = 13.1 Hz), 42.5 (d, J = 29.9 Hz, C_q), 43.9, 120.9 (part of the q, OTf). **^{31}P NMR (162 MHz, CDCl$_3$):** δ = 38.60 (t, $^1J_{PH}$ = 465.4 Hz). **^{19}F NMR (377 MHz, CDCl$_3$):** δ = -78.28. **HRMS:** [M-OTf]$^+$ calcd for $C_{22}H_{44}P$: 339.3175; found: 339.3174. **IR (ATR, cm^{-1}):** v = 2976 (w), 2947 (m), 2917 (m), 2840 (w), 2410 (w), 1446 (m), 1460 (m), 1385 (w), 1272 (s), 1249 (s), 1222 (s), 1167 (s), 1154 (s), 1077 (w), 1055 (w), 1026 (s), 984 (s), 916 (w), 888 (w), 758 (w), 737 (w), 713 (w).

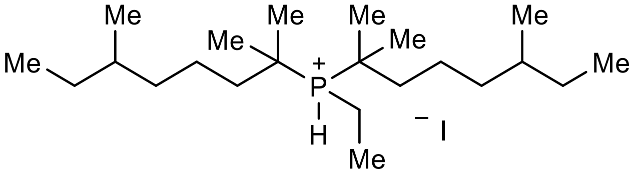

Ethylbis(2,6-dimethyloctan-2-yl)phosphonium iodide, GreenPhos 8. Ethylbis(2,6-dimethyloctan-2-yl)phosphonium iodide was obtained from di-(2,6-dimethyloctan-2-yl)phosphonium trifluoromethanesulfonate (GreenPhos 3, 0.24 g, 0.523 mmol) using the method B. The product was isolated as a light-yellow oil (0.23 g, 93%) after purification with flash chromatography (40% DCM in CH_3CN). **^{1}H NMR (400 MHz, CDCl$_3$):** δ = 0.80-0.83 (m, 12H, Me), 1.04-1.14 (m, 4H), 1.23-1.48 (m, 10H), 1.54 (dd, $^3J_{PH}$ = 16.9 Hz, $^4J_{HH}$ = 5.38 Hz, 12H), 1.58-1.59 (m, 3H, Me), 1.65-1.79 (m, 4H), 2.26-2.34 (m, 2H, 4H, CH_2), 7.61 (br d, $^1J_{PH}$ = 470.9 Hz, 1H, PH). **^{13}C NMR (101 MHz, CDCl$_3$):** δ = 9.2 (d, $^1J_{PC}$ = 40.5 Hz, CH_2), 11.4, 19.2, 20.5 (d, J = 8.15 Hz), 20.5 (d, J = 8.2), 24.3. 24.7, 29.4, 34.3, 36.7, 37.3 (d, $^1J_{PC}$ = 32.41 Hz, C_q), 39.0. **^{31}P NMR (162 MHz, CDCl$_3$):** δ = 37.30 (d, $^1J_{PH}$ = 464.5 Hz). **HRMS:** [M-I]$^+$ calcd for $C_{22}H_{48}P$: 343.3488; found: 343.3487. **IR (ATR, cm^{-1}):** v = 2958 (m), 2927 (m), 2874 (m), 2245 (w), 1465 (m), 1397 (w), 1378 (m), 1261 (s), 1224 (m), 1153 (m), 1052 (w), 1032 (s), 921 (m), 732 (s).

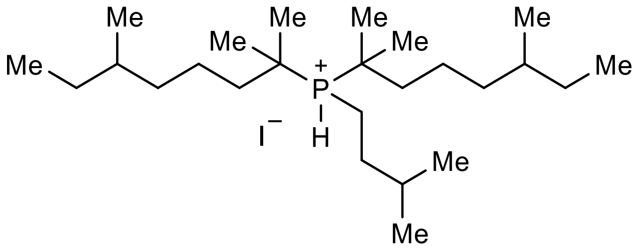

Isopentylbis(2,6-dimethyloctan-2-yl)phosphonium iodide, GreenPhos 9. Isopentylbis(2,6-dimethyloctan-2-yl)phosphonium iodide was obtained from di-(2,6-dimethyloctan-2-yl)phosphonium trifluoromethanesulfonate (GreenPhos 3, 0.24 g, 0.523 mmol) using the method B. The product was isolated as a colorless oil (0.11 g, 43%) after purification with flash chromatography (eluent: 30% CH_3CN in EtOAc). **^{1}H NMR (400 MHz, $CDCl_3$):** δ = 0.82-0.86 (m, 12H, Me), 0.96 (d, $^3J_{HH}$ = 6.4 Hz, 6H, Me), 1.07-1.19 (m, 4H, CH_2), 1.23-1.44 (m, 10H), 1.53 (dd, $^3J_{PH}$ = 16.9, $^4J_{HH}$ = 6.20 Hz, 12H, Me), 1.65-1.83 (m, 7H), 2.06-2.15 (m, 2H, CH_2), 7.91 (dt, $^1J_{PH}$ = 475.4, $^3J_{HH}$ = 3.4 Hz, 1H, PH). **^{13}C NMR (101 MHz, $CDCl_3$):** δ = 11.4 (Me), 13.1 (d, $^1J_{CP}$ = 39.2 Hz, CH_2), 19.2 (d, $^4J_{CP}$ = 4.4 Hz, Me), 20.6 (d, J_{CP} = 8.1 Hz), 21.8 (Me), 24.4 (Me), 24.7 (d, $^3J_{CP}$ = 1.9 Hz, Me), 29.5 (d, J_{CP} = 5.12 Hz), 29.6 (d, J_{CP} = 12.8 Hz), 34.3 (d, J_{CP} = 1.71 Hz), 35.3 (d, J_{CP} = 5.8 Hz), 36.8, 37.4 (d, $^1J_{CP}$ = 32.3 Hz, C_q), 39.1. **^{31}P NMR (162 MHz, $CDCl_3$):** δ = 33.22 (t, $^1J_{PH}$ = 478.12 Hz). **HRMS:** $[M-I]^+$ calcd for $C_{25}H_{54}P$: 385.3958; found: 385.3957. **IR (ATR, cm^{-1}):** ν = 2955 (s), 2930 (s), 2901 (s), 2870 (s), 2265 (m), 2254 (m), 1459 (s), 1379 (m), 1488 (m), 1224 (w), 1156 (m), 1118 (w), 1079 (w), 1032 (s), 953 (m), 768 (m), 735 (m), 724 (m).

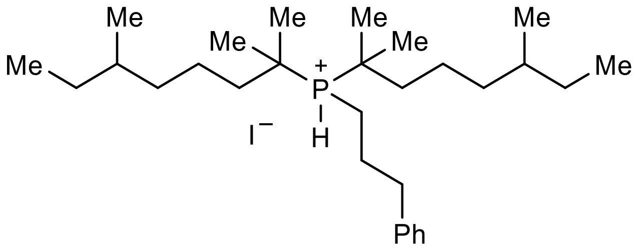

(3-Phenylpropyl)bis(2,6-dimethyloctan-2-yl)phosphonium iodide, GreenPhos 10. (3-Phenylpropyl)bis(2,6-dimethyloctan-2-yl)phosphonium iodide was obtained from di-(2,6-dimethyloctan-2-yl)phosphonium trifluoromethanesulfonate (GreenPhos 3, 0.25 g, 0.528 mmol) using the method B. The product was isolated as a yellowish oil (0.13 g, 40%) after purification with flash chromatography (eluent: 30% CH_3CN in EtOAc). **^{1}H NMR (400 MHz, $CDCl_3$):** δ = 0.84-0.87 (m, 12H, Me), 1.01-1.11 (m, 4H, CH_2), 1.21-1.37 (m, 10H), 1.41-1.48 (m, 12H, Me), 1.53-1.68 (m, 4H), 2.01-2.09 (m, 2H, CH_2), 2.24-2.34 (m, 2H, CH_2), 2.8 (t, $^3J_{HH}$ = 6.7 Hz, 2H, CH_2), 7.20-7.25 (m, 3H, Ar), 7.30-7.33 (m, 2.5H, Ar + 0.5PH), 8.49 (0.5H, PH). *Unable to determine the multiplet peak and calculate $^1J_{PH}$ due to the overlap of PH signal with the aromatic region.* **^{13}C NMR (101 MHz, $CDCl_3$):** δ = 11.5 (Me), 13.8 (d, $^1J_{CP}$ = 39.3 Hz, CH_2), 19.2 (Me), 20.6 (d, J_{CP} = 8.1 Hz), 21.8 (Me), 24.3 (Me), 24.6 (d, $^3J_{CP}$ = 2.7 Hz, Me), 28.1 (d, J_{CP} = 5.19 Hz) 29.5 (d, J_{CP} = 1.52 Hz), 34.4, 36.5 (d, J_{CP} = 13.4 Hz), 36.8, 37.4 (d, $^1J_{CP}$ = 32.3 Hz, C_q), 39.1 (d, J_{CP} = 1.8 Hz), 126.9 (Ar), 128.9 (Ar), 128.9 (Ar), 139.2 (Ar). **^{31}P NMR (162 MHz, $CDCl_3$):** δ = 32.7 (t, $^1J_{PH}$ = 480.2 Hz). **HRMS:** $[M-I]^+$ calcd for $C_{29}H_{54}P$: 433.3958; found: 433.3958. **IR (ATR, cm^{-1}):** ν = 2956 (m), 2929 (m), 2908 (m), 2873 (m), 2294 (w), 1498 (w), 1459 (m), 1401 (w), 1379 (m), 1260 (s), 1223 (m), 1154 (s), 1077 (w), 1032 (s), 896 (w), 800 (w), 756 (w), 735 (m), 714 (w), 698 (m).

3.3. Synthesis of renewable phosphinites (GreenPhos 11-20)

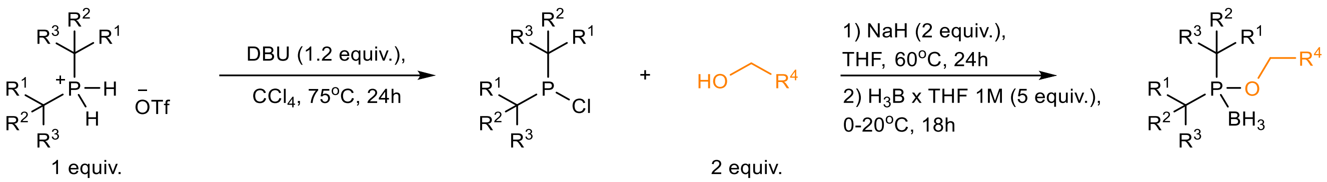

Inside of an argon-filled glovebox an oven dried 10 mL vial was sequentially charged with corresponding di-*tert*-alkylphosphonium salt (1 equiv., 0.839 mmol), DBU (1.2 equiv.) and anhydrous degassed tetrachloromethane (3 mL). The vial was sealed with a stopper equipped with a septum, removed from the glovebox and stirred at 75 °C for 24h. Next, the vial containing the reaction mixture was transferred back to the glovebox. The solution of the *in situ* generated chloro-di-*tert*-alkylphosphine was transferred into an oven dried 25 mL Schlenk flask using a syringe. The separated layer of byproducts (in the vial) was washed with anhydrous degassed tetrachloromethane (2 mL, once), which was added to the content of the Schlenk flask. Afterwards, the Schlenk flask was sealed with a rubber septum and removed from the glovebox. The volatiles were carefully removed under vacuum to give the crude chloro-di-*tert*-alkylphosphine, which was used for the following alkoxylation without further purification.

Inside of the glovebox, an oven-dried 25 mL round bottom flask was sequentially charged with the corresponding alcohol/phenol (2 equiv.), anhydrous degassed THF (4 mL) and NaH (2 equiv., addition of NaH can be accompanied with intense H_2 elimination). The flask was sealed with a rubber septum, removed from the glovebox and equipped with an Ar balloon. This was followed by stirring of the reaction mixture at 50 °C for 1h. The resulting solution was left to cool to room temperature. Next, the solution of corresponding alkoxide/phenolate was transferred to the previously prepared solution of the crude chloro-di-*tert*-alkylphosphine in anhydrous degassed THF (2 mL). The resulting mixture was stirred at 60 °C for 24h. Afterwards, the Schlenk flask containing the reaction mixture was transferred into an ice bath, which was followed by dropwise addition of borane tetrahydrofuran complex solution (1.0 M in THF, 5 equiv.). The resulting mixture was allowed to reach room temperature and was stirred at 20 °C for 18h. Next, the reaction mixture was opened to air, and transferred into a 250 mL separating funnel. The mixture was diluted with ethyl acetate (100 mL), which was followed by careful addition of 6M HCl solution (30 mL). The two phases were separated, and the organic phase was sequentially washed with water (30 mL, once). The two phases were separated, and the resulting ethyl acetate solution was evaporated to dryness. Target borane adducts of renewable phosphinites were obtained following purification by column chromatography using mixtures of heptane/ethyl acetate as eluent.

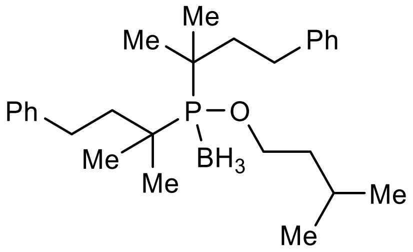

(Isopentyloxy)bis(2-methyl-4-phenylbutan-2-yl)phosphane borane adduct, GreenPhos 11. Starting from 0.839 mmol of corresponding di-*tert*-alkylphosphonium salt the product was obtained as a colorless oil (0.182 g, 51%). **^{1}H NMR (400 MHz, CDCl$_3$):** δ = 0.49-0.85 (m, 3H, BH_3), 0.97 (d, J = 6.7 Hz, 6H, 2xMe), 1.33-1.49 (m, 12H, 4xMe), 1.56-1.61 (m, 2H, CH_2), 1.75-1.82 (m, 1H, CH), 2.06 (q, J = 8.1 Hz, 4H, 2xCH_2), 2.69-2.76 (m, 4H, 2xCH_2), 4.09 (q, J = 6.4 Hz, 2H, CH_2O), 7.20-7.35 (m, 10H, 2xPh). **^{13}C**

NMR (101 MHz, CDCl₃): δ = 22.7, 23.3, 23.4, 24.8, 29.8 (d, *J* = 7.8 Hz), 39.6, 39.9 (dd, *J* = 4.2, 2.0 Hz), 40.0, 68.8 (d, *J* = 5.6 Hz), 126.0, 128.4, 128.6, 142.3. **³¹P NMR (162 MHz, CDCl₃):** δ = 143.3 (d, *J* = 86.1 Hz). **¹¹B NMR (128 MHz, CDCl₃):** δ = -43.5. **HRMS:** $[M+H]^+$ calcd. for $C_{27}H_{45}BOP$: 427.3296; found: 427.3295. **IR (ATR, cm⁻¹):** *v* = 3028 (w), 2958 (w), 2931 (w), 2869 (w), 2381 (m), 2355 (w), 1603 (w), 1498 (w), 1466 (w), 1454 (m), 1386 (w), 1139 (w), 1074 (s), 1052 (s), 984 (s), 973 (s), 893 (m), 804 (w), 784 (w), 742 (s), 700 (s).

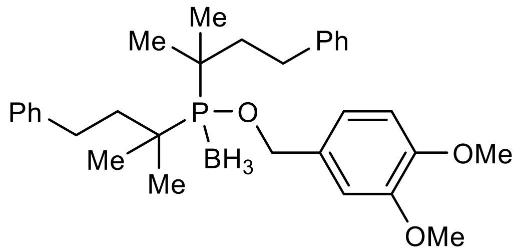

((3,4-Dimethoxybenzyl)oxy)bis(2-methyl-4-phenylbutan-2-yl)phosphane borane adduct, GreenPhos 12. Starting from 0.839 mmol of corresponding di-*tert*-alkylphosphonium salt the product was obtained as a colorless oil (0.170 g, 40%). **¹H NMR (400 MHz, CDCl₃):** δ = 0.58-0.88 (m, 3H, BH_3), 1.35-1.41 (m, 12H, 4xMe), 1.98-2.09 (m, 4H, $2xCH_2$), 2.61-2.69 (m, 4H, $2xCH_2$), 3.81 (s, 3H, OMe), 3.90 (s, 3H, OMe), 5.01 (d, *J* = 6.7 Hz, 2H, OCH_2), 6.82-6.84 (m, 1H, Ar), 6.91-6.95 (m, 2H, Ar), 7.13-7.23 (m, 7H, Ph), 7.27-7.31 (m, 3H, Ph). **¹³C NMR (101 MHz, CDCl₃):** δ = 23.2 (d, *J* = 2.2 Hz), 23.3 (d, *J* = 1.7 Hz), 29.7 (d, *J* = 7.9 Hz), 39.6, 39.8 (d, *J* = 2.1 Hz), 39.9, 55.9 (d, *J* = 9.0 Hz), 71.7 (d, *J* = 5.1 Hz), 111.2 (d, *J* = 51.6 Hz), 120.9, 126.0, 128.4, 128.5, 128.6, 130.0 (d, *J* = 5.5 Hz), 142.2, 149.0 (d, *J* = 2.1 Hz). **³¹P NMR (162 MHz, CDCl₃):** δ = 145.9 (d, *J* = 75.9 Hz). **¹¹B NMR (128 MHz, CDCl₃):** δ = -43.3. **HRMS:** $[M+H]^+$ calcd. for $C_{31}H_{45}BO_3P$: 507.3194; found: 507.3194. **IR (ATR, cm⁻¹):** *v* = 2951 (w), 2936 (w), 2385 (w), 1604 (w), 1595 (w), 1517 (s), 1498 (w), 1465 (m), 1454 (m), 1421 (w), 1369 (w), 1265 (s), 1239 (m), 1159 (m), 1140 (s), 1072 (w), 1029 (s), 1001 (s), 972 (s), 913 (w), 799 (m), 747 (s), 734 (s), 699 (s).

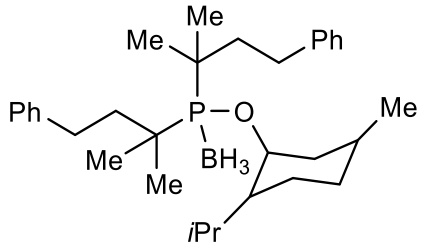

((2-Isopropyl-5-methylcyclohexyl)oxy)bis(2-methyl-4-phenylbutan-2-yl)phosphane borane adduct, GreenPhos 13. Starting from 0.839 mmol of corresponding di-*tert*-alkylphosphonium salt the product was obtained as a colorless oil (0.106 g, 26%). **¹H NMR (400 MHz, CDCl₃):** δ = 0.50-1.08 (m, 15H, BH_3, CH_2, Me), 1.26-1.45 (m, 14H, CH_2, Me), 1.62-1.70 (m, 2H, CH_2), 1.90-2.22 (m, 4H, $2xCH_2$), 2.39 (pd, *J* = 6.9, 2.1 Hz, 1H, CH), 2.57-2.71 (m, 5H, $2xCH_2$, CH), 4.05 (tt, *J* = 10.4, 4.0 Hz, 1H, CH), 7.71-7.37 (m, 10H, 2xPh). **¹³C NMR (101 MHz, CDCl₃):** δ = 16.6, 21.7, 22.4, 23.0 (d, *J* = 14.3 Hz), 23.4 (d, *J* = 2.5 Hz), 24.1, 24.3 (d, *J* = 3.9 Hz), 25.2, 29.6 (d, *J* = 8.7 Hz), 30.0 (d, *J* = 7.4 Hz), 31.6, 34.1, 39.1, 39.2 (d, *J* = 1.7 Hz), 39.4, 40.1 (d, *J* = 1.5 Hz), 40.3, 40.6, 44.2, 50.2 (d, *J* = 5.5 Hz), 81.3 (d, *J* = 7.2 Hz), 126.0 (d, *J* = 4.9 Hz), 128.4, 128.5, 128.6 (d, *J* = 3.3 Hz), 142.4 (d, *J* = 9.0 Hz). **³¹P NMR (162 MHz, CDCl₃):** δ = 138.9 (d, *J* = 78.3 Hz). **¹¹B NMR (128 MHz, CDCl₃):** δ = -42.3. **HRMS:** $[M+H]^+$ calcd. for $C_{32}H_{53}BOP$: 495.3922; found: 495.3921. **IR (ATR, cm⁻¹):** *v* = 2955 (m), 2925 (m), 2868 (w), 2386 (w), 1604 (w), 1498 (w), 1454 (m), 1386 (w), 1369 (w), 1276 (w), 1144 (w), 1073 (m), 975 (s), 962 (s), 800 (m), 868 (m), 746 (s), 699 (s).

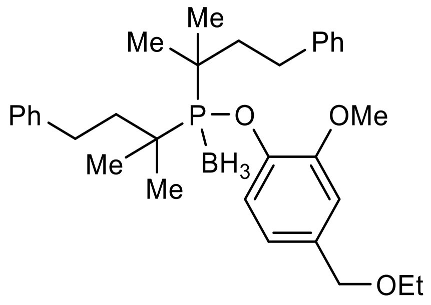

(4-(Ethoxymethyl)-2-methoxyphenoxy)bis(2-methyl-4-phenylbutan-2-yl)phosphane borane adduct*, *GreenPhos 14. Starting from 0.839 mmol of corresponding di-*tert*-alkylphosphonium salt the product was obtained as a colorless oil (0.220 g, 50%). **^{1}H NMR (400 MHz, $CDCl_3$):** δ = 0.62-0.98 (m, 3H, BH_3), 1.28 (t, *J* = 7.0 Hz, 3H, Me), 1.37-1.52 (m, 12H, 4xMe), 2.16-2.23 (m, 4H, 2xCH_2), 2.68-2.72 (m, 4H, 2xCH_2), 3.56 (q, *J* = 7.0 Hz, 2H, OCH_2), 3.86 (s, 3H, OMe), 4.47 (s, 2H, OCH_2), 6.85 (dd, *J* = 8.3, 2.0 Hz, 1H, Ar), 6.95 (s, 1H, Ar), 7.17-7.32 (m, 10H, 2xPh), 7.44 (d, *J* = 8.3 Hz, 1H, Ar). **^{13}C NMR (101 MHz, $CDCl_3$):** δ = 15.4, 23.3 (d, *J* = 2.6 Hz), 23.5 (d, *J* = 1.7 Hz), 29.8 (d, *J* = 7.7 Hz), 39.9 (d, *J* = 2.0 Hz), 40.8 (d, *J* = 27.5 Hz), 55.6, 65.8, 72.5, 111.5, 119.8, 121.2 (d, *J* = 2.4 Hz), 126.0, 128.5, 128.6, 134.8, 142.4, 143.0 (d, *J* = 7.2 Hz), 150.5 (d, *J* = 3.8 Hz). **^{31}P NMR (162 MHz, $CDCl_3$):** δ = 152.5 (d, *J* = 53.1 Hz). **^{11}B NMR (128 MHz, $CDCl_3$):** δ = -41.7. **HRMS:** $[M+H]^+$ calcd. for $C_{32}H_{47}BO_3P$: 521.3350; found: 521.3350. **IR (ATR, cm^{-1}):** *v* = 2965 (w), 2935 (w), 2865 (w), 2390 (w), 2348 (w), 1603 (w), 1509 (s), 1499 (m), 1466 (m), 1454 (m), 1421 (w), 1387 (w), 1370 (w), 1270 (s), 1211 (s), 1156 (s), 1124 (s), 1036 (w), 889 (s), 814 (w), 808 (m), 747 (s), 699 (s).

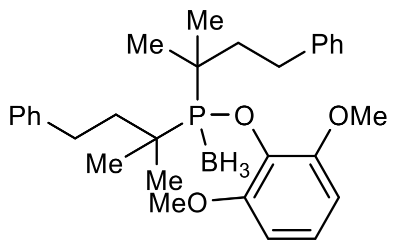

(2,6-Dimethoxyphenoxy)bis(2-methyl-4-phenylbutan-2-yl)phosphane borane adduct*, *GreenPhos 15. Starting from 0.839 mmol of corresponding di-*tert*-alkylphosphonium salt the product was obtained as a colorless oil (0.173 g, 42%). **^{1}H NMR (400 MHz, $CDCl_3$):** δ = 0.14-0.68 (m, 3H, BH_3), 1.54 (dd, *J* = 22.0, 14.2 Hz, 12H, 4xMe), 2.16-2.25 (m, 2H, CH_2), 2.34-2.43 (m, 2H, CH_2), 2.71-2.75 (m, 4H, 2xCH_2), 3.76 (s, 6H, 2xOMe), 6.55 (d, *J* = 8.4 Hz, 2H, Ar), 7.04 (t, *J* = 8.4 Hz, 1H, Ar), 7.19-7.32 (m, 10H, 2xPh). **^{13}C NMR (101 MHz, $CDCl_3$):** δ = 23.4 (d, *J* = 1.6 Hz), 23.6 (d, *J* = 3.2 Hz), 29.8 (d, *J* = 8.0 Hz), 39.4 (d, *J* = 1.7 Hz), 41.2 (d, *J* = 25.6 Hz), 55.1, 104.8 (d, *J* = 1.3 Hz), 124.5 (d, *J* = 1.5 Hz), 125.9, 128.4, 128.5, 132.1 (d, *J* = 8.1 Hz), 142.6, 152.5 (d, *J* = 2.1 Hz). **^{31}P NMR (162 MHz, $CDCl_3$):** δ = 156.6 (d, *J* = 57.6 Hz). **^{11}B NMR (128 MHz, $CDCl_3$):** δ = -41.8. **HRMS:** $[M+H]^+$ calcd. for $C_{30}H_{43}BO_3P$: 493.3037; found: 493.3036. **IR (ATR, cm^{-1}):** *v* = 3001 (w), 2966 (w), 2939 (w), 2840 (w), 2445 (w), 2397 (w), 2331 (w), 1599 (m), 1493 (s), 1479 (s), 1466 (s), 1459 (s), 1453 (s), 1387 (w), 1369 (w), 1303 (m), 1259 (s), 1204 (s), 1187 (m), 1114 (s), 1068 (s), 896 (s), 751 (s), 708 (s), 704 (s).

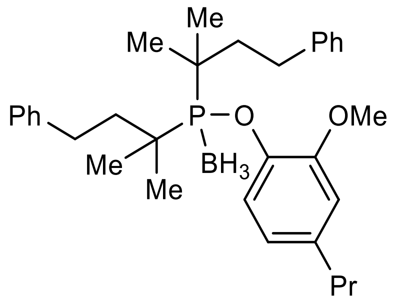

(2-methoxy-4-propylphenoxy)bis(2-methyl-4-phenylbutan-2-yl)phosphane borane adduct*, *GreenPhos 16. Starting from 0.839 mmol of corresponding di-*tert*-alkylphosphonium salt the product was obtained as a colorless oil (0.236 g, 56%). **^{1}H NMR (400 MHz, $CDCl_3$):** δ = 0.12-0.77 (m, 3H, BH_3), 0.97 (t, *J* = 7.3 Hz, 3H, Me), 1.40-1.56 (m, 12H, 4xMe), 1.65 (h, *J* = 7.5 Hz, 2H, CH_2), 2.14-2.26 (m, 4H, 2xCH_2), 2.56 (t, *J* = 7.7 Hz, 2H, CH_2), 2.68-2.72 (m, 4H, 2xCH_2), 3.83 (s, 3H, OMe), 6.69-6.74 (m, 2H, Ar), 7.16-7.23 (m, 6H, Ph), 7.28-7.31 (m, 4H, Ph), 7.36 (d, *J* = 8.1 Hz, 1H, Ar). **^{13}C NMR (101 MHz, $CDCl_3$):** δ = 14.0, 23.4 (d, *J* = 2.6 Hz), 23.6 (d, *J* = 1.6 Hz), 24.7, 29.8 (d, *J* = 7.7

Hz), 37.9, 39.9 (d, *J* = 2.0 Hz), 40.7 (d, *J* = 27.6 Hz), 55.6, 112.4, 120.2, 121.3 (d, *J* = 2.4 Hz), 126.0, 128.5, 128.6, 139.0, 141.6 (d, *J* = 7.1 Hz), 142.5, 150.1 (d, *J* = 3.7 Hz). **^{31}P NMR (162 MHz, CDCl$_3$):** δ = 151.8 (d, *J* = 54.4 Hz). **^{11}B NMR (128 MHz, CDCl$_3$):** δ = -41.7. **HRMS:** $[M+H]^+$ calcd. for $C_{32}H_{47}BO_2P$: 505.3401; found: 505.3402. **IR (ATR, cm^{-1}):** *v* = 3028 (w), 2957 (w), 2933 (w), 2871 (w), 2408 (m), 2390 (w), 2347 (w), 1602 (w), 1593 (w), 1509 (s), 1454 (s), 1418 (w), 1390 (w), 1369 (w), 1290 (w), 1270 (s), 1213 (s), 1153 (s), 1071 (m), 1030 (m), 910 (s), 887 (m), 849 (m), 810 (s), 802 (m), 735 (s), 699 (s).

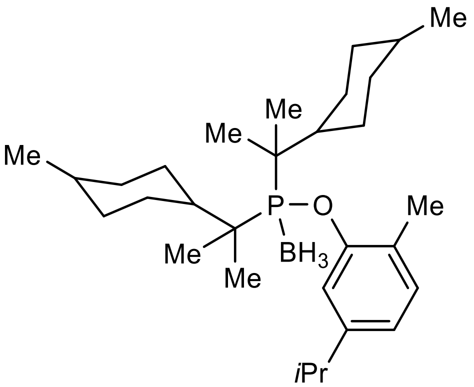

(5-Isopropyl-2-methylphenoxy)bis(2-(4-methylcyclohexyl)propan-2-yl)phosphane borane adduct, GreenPhos 17. Starting from 0.651 mmol of corresponding di-*tert*-alkylphosphonium salt the product was obtained as a colorless oil (0.159 g, 52%). **^{1}H NMR (400 MHz, CDCl$_3$):** δ = 0.67-1.16 (m, 16H, BH$_3$, CH$_2$, Me), 1.21-1.46 (m, 21H, Me, CH, CH$_2$), 1.59-1.64 (m, 2H, CH$_2$), 1.71-1.77 (m, 4H, 2xCH$_2$), 2.00-2.09 (m, 4H, 2xCH$_2$), 2.24 (s, 3H, Me), 2.86 (p, *J* = 6.9 Hz, 1H, CH), 6.79 (d, *J* = 7.6 Hz, 1H, Ar), 7.01 (d, *J* = 7.6 Hz, 1H, Ar), 7.73 (s, 1H, Ar). **^{13}C NMR (101 MHz, CDCl$_3$):** δ = 17.3, 20.7 (d, *J* = 2.2 Hz), 21.5 (d, *J* = 4.0 Hz), 22.6, 24.1, 27.7 (d, *J* = 5.6 Hz), 28.2 (d, *J* = 3.9 Hz), 32.8, 33.9, 35.5 (d, *J* = 2.6 Hz), 43.3, 45.1 (d, *J* = 25.1 Hz), 117.2 (d, *J* = 3.2 Hz), 120.8, 124.7 (d, *J* = 5.1 Hz), 130.6, 148.0, 152.9 (d, *J* = 8.2 Hz). **^{31}P NMR (162 MHz, CDCl$_3$):** δ = 154.4 (d, *J* = 54.2 Hz). **^{11}B NMR (128 MHz, CDCl$_3$):** δ = -38.1. **HRMS:** $[M+H]^+$ calcd. for $C_{30}H_{55}BOP$: 473.4078; found: 473.4078. **IR (ATR, cm^{-1}):** *v* = 2946 (s), 2918 (s), 2868 (m), 2851 (m), 2391 (s), 2380 (s), 2357 (w), 1616 (w), 1572 (w), 1561 (w), 1502 (w), 1457 (s), 1450 (s), 1410 (m), 1387 (m), 1369 (m), 1277 (w), 1240 (s), 1176 (m), 1138 (m), 1118 (s), 1001 (w), 962 (s), 955 (s), 886 (s), 875 (s), 819 (s), 768 (m), 752 (m), 738 (m), 690 (s).

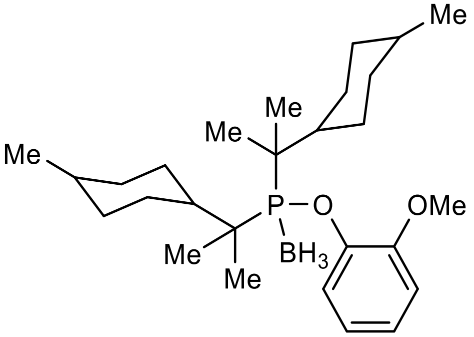

(2-Methoxyphenoxy)bis(2-(4-methylcyclohexyl)propan-2-yl)phosphane borane adduct, GreenPhos 18. Starting from 0.651 mmol of corresponding di-*tert*-alkylphosphonium salt the product was obtained as a colorless oil (0.168 g, 58%). **^{1}H NMR (400 MHz, CDCl$_3$):** δ = 0.68-0.99 (m, 12H, BH$_3$, CH$_2$, Me), 1.05-1.16 (m, 4H, 2xCH$_2$), 1.23-1.44 (m, 15H, CH$_2$, CH, Me), 1.62-1.66 (m, 2H, CH$_2$), 1.72-1.83 (m, 4H, 2xCH$_2$), 1.93-2.02 (m, 2H, CH$_2$), 2.06-2.12 (m, 2H, CH$_2$), 3.80 (s, 3H, OMe), 6.83-6.91 (m, 2H, Ar), 6.97-7.02 (m, 1H, Ar), 7.54 (dd, *J* = 8.1, 1.5 Hz, 1H, Ar). **^{13}C NMR (101 MHz, CDCl$_3$):** δ = 20.9 (d, *J* = 4.0 Hz), 21.3 (d, *J* = 2.3 Hz), 22.7, 27.8 (d, *J* = 5.4 Hz), 28.2 (d, *J* = 3.9 Hz), 32.8, 35.7 (d, *J* = 4.7 Hz), 43.2, 44.9 (d, *J* = 24.1 Hz), 55.7, 112.1, 120.4, 121.1 (d, *J* = 2.2 Hz), 123.6, 143.3 (d, *J* = 7.9 Hz), 150.6 (d, *J* = 4.1 Hz). **^{31}P NMR (162 MHz, CDCl$_3$):** δ = 159.4 (d, *J* = 64.3 Hz). **^{11}B NMR (128 MHz, CDCl$_3$):** δ = -39.3. **HRMS:** $[M+H]^+$ calcd. for $C_{27}H_{49}BO_2P$: 447.3558; found: 447.3557. **IR (ATR, cm^{-1}):** *v* = 2944 (m), 2910 (s), 2839 (m), 2402 (w), 2383 (w), 2349 (w), 1588 (w), 1502 (s), 1457 (s), 1450 (s), 1388 (w), 1370 (w), 1322

(w), 1260 (s), 1209 (s), 1174 (m), 1159 (m), 1118 (s), 1072 (m), 1027 (s), 928 (s), 793 (m), 741 (s), 712 (w).

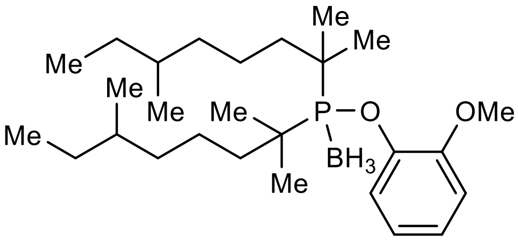

Bis(2,6-dimethyloctan-2-yl)(2-methoxyphenoxy)phosphane borane adduct**, *****GreenPhos 19.*** Starting from 0.645 mmol of corresponding di-*tert*-alkylphosphonium salt the product was obtained as a colorless oil (0.151 g, 52%). **^{1}H NMR (400 MHz, CDCl$_3$):** δ = 0.48-0.89 (m, 15H, BH_3, Me), 1.06-1.15 (m, 4H, 2xCH_2), 1.24-1.44 (m, 22H, CH_2, CH, Me), 1.73-1.81 (m, 4H, 2xCH_2), 3.82 (s, 3H, OMe), 6.85-6.89 (m, 2H, Ar), 7.01-7.05 (m, 1H, Ar), 7.43 (d, *J* = 8.1 Hz, 1H, Ar). **^{13}C NMR (101 MHz, CDCl$_3$):** δ = 11.6 (d, *J* = 2.1 Hz), 19.4 (d, *J* = 1.8 Hz), 20.6 (dd, *J* = 8.1, 2.2 Hz), 23.2 (dd, *J* = 4.8, 2.2 Hz), 23.3 (d, *J* = 3.8 Hz), 29.6 (d, *J* = 1.8 Hz), 34.6 (d, *J* = 1.7 Hz), 37.4 (d, *J* = 1.2 Hz), 38.0 (d, *J* = 2.4 Hz), 40.7 (d, *J* = 27.9 Hz), 55.5, 112.1 (d, *J* = 3.2 Hz), 120.5, 121.8 (d, *J* = 2.4 Hz), 124.1, 143.9 (d, *J* = 7.2 Hz), 150.6 (d, *J* = 3.7 Hz). **^{31}P NMR (162 MHz, CDCl$_3$):** δ = 152.6 (d, *J* = 68.7 Hz). **^{11}B NMR (128 MHz, CDCl$_3$):** δ = -41.8. **HRMS:** $[M+H]^+$ calcd. for $C_{27}H_{53}BO_2P$: 451.3871; found: 451.3868. **IR (ATR, cm^{-1}):** *ν* = 2954 (m), 2927 (m), 2873 (m), 2395 (w), 2349 (w), 1599 (w), 1589 (w), 1503 (s), 1458 (s), 1379 (w), 1259 (s), 1209 (s), 1176 (s), 1116 (s), 1069 (w), 1049 (w), 1031 (m), 908 (s), 790 (s), 712 (s).

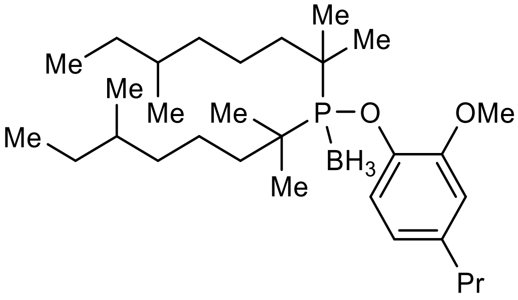

Bis(2,6-dimethyloctan-2-yl)(2-methoxy-4-propylphenoxy)phosphane borane adduct**, *****GreenPhos 20.*** Starting from 0.645 mmol of corresponding di-*tert*-alkylphosphonium salt the product was obtained as a colorless oil (0.183 g, 58%). **^{1}H NMR (400 MHz, CDCl$_3$):** δ = 0.50-0.91 (m, 14H, BH_3, CH, Me), 0.94 (t, *J* = 7.3 Hz, 3H, Me), 1.06-1.17 (m, 4H, 2xCH_2), 1.23-1.44 (m, 23H, CH_2, Ch, Me), 1.63 (h, *J* = 7.4 Hz, 2H, CH_2), 1.73-1.81 (m, 4H, 2xCH_2), 2.51-2.55 (m, 2H, CH_2), 3.81 (s, 3H, OMe), 6.66-6.69 (m, 2H, Ar), 7.30 (d, *J* = 8.2 Hz, 1H, Ar). **^{13}C NMR (101 MHz, CDCl$_3$):** δ = 11.5 (d, *J* = 2.6 Hz), 14.0, 19.4 (d, *J* = 1.6 Hz), 20.6 (dd, *J* = 8.0, 1.6 Hz), 23.2 (dd, *J* = 4.9, 2.2 Hz), 23.3 (d, *J* = 4.3 Hz), 24.7, 26.3, 29.6 (d, *J* = 1.7 Hz), 34.6 (d, *J* = 2.2 Hz), 37.4, 37.9, 38.0, 40.6 (d, *J* = 28.1 Hz), 55.5, 112.3, 120.1, 121.3 (d, *J* = 2.4 Hz), 138.7, 141.8 (d, *J* = 7.0 Hz), 150.2 (d, *J* = 3.7 Hz). **^{31}P NMR (162 MHz, CDCl$_3$):** δ = 151.9 (d, *J* = 57.4 Hz). **^{11}B NMR (128 MHz, CDCl$_3$):** δ = -42.0. **HRMS:** $[M+H]^+$ calcd. for $C_{30}H_{59}BO_2P$: 493.4340; found: 493.4341. **IR (ATR, cm^{-1}):** *ν* = 2957 (s), 2929 (s), 2871 (m), 2390 (w), 2350 (w), 1592 (w), 1511 (s), 1465 (s), 1418 (w), 1379 (w), 1269 (s), 1212 (s), 1155 (s), 1130 (s), 1069 (m), 1039 (m), 906 (s), 814 (s), 788 (w), 734 (s).

4. Estimation of the electron-donating abilities of prepared ligands

General procedure for the selenation of phosphonium salts[12]

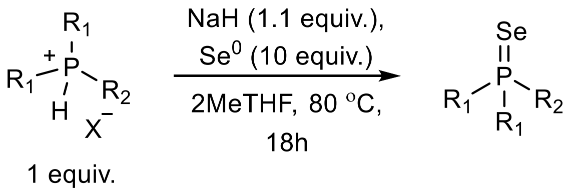

Inside of an argon-filled glovebox the appropriate phosphonium salt (0.02 mmol, 1 equiv.) and selenium powder (10 equiv.) were weighed into a 10 mL reaction vial. A 2 mL aliquot of a stock solution of sodium hydride in 2MeTHF was added to the reaction flask in such a way that the final equivalents of NaH in the reaction flask equals 1.1 equivalent. The reaction vial was capped with a septum and removed from the glovebox. The reaction proceeded at 80 °C for 24h. Afterwards, the reaction was removed from the heating source and left to reach room temperature. The solvent was evaporated with a rotary evaporator. Thereafter 0.5 mL $CDCl_3$ was added to the reaction residue and analyzed with ^{31}P NMR spectroscopy.

General procedure for the selenation of borane protected phosphinites

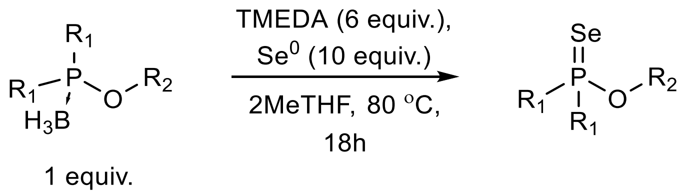

Inside of an argon-filled glovebox the appropriate phosphinite (0.02 mmol, 1 equiv.), TMEDA (6 equiv.) and selenium powder (10 equiv.) were weighed into a 10 mL reaction vial. Then, 2 mL 2MeTHF (degassed and anhydrous) was added to the reaction vial. The reaction vial was capped with a septum and removed from the glovebox. The reaction proceeded at 80 °C for 24h. Afterwards, the reaction was removed from the heating source and left to reach room temperature. The solvent was evaporated with a rotary evaporator. Thereafter 0.5 mL $CDCl_3$ was added to the reaction residue and analyzed with ^{31}P NMR spectroscopy.

[12] Z. L. Niemeyer, A. Milo, D. P. Hickey and M. S. Sigman, *Nat. Chem.*, 2016, **8**, 610-617.

Table S1. The ^{31}P NMR resonance (δ_P, in ppm) and the corresponding phosphorous selenium coupling constants ($^1J_{PSe}$, in Hz) for the selenides of GreenPhos 1-20 in $CDCl_3$.

Entry	Ligand name	δ_P (ppm)[a]	$^1J_{PSe}$ (Hz)
1	GreenPhos 1	119.18	691.1
2	GreenPhos 2	115.91	731.5
3	GreenPhos 3	121.49	738.6
4	GreenPhos 4	81.90	697.8
5	GreenPhos 5	84.45	697.9
6	GreenPhos 6	81.42	699.9
7	GreenPhos 7	85.91	682.2
8	GreenPhos 8	85.03	688.9
9	GreenPhos 9	82.52	688.9
10	GreenPhos 10	82.11	691.1
11	GreenPhos 11	137.18	769.6
12	GreenPhos 12	139.40	771.8
13	GreenPhos 13	131.22	764.4
14	GreenPhos 14	140.80	796.3
15	GreenPhos 15	142.72	803.5
16	GreenPhos 16	142.34	792.2
17	GreenPhos 17	138.81	785.4
18	GreenPhos 18	146.00	785.5
19	GreenPhos 19	142.73	787.7
20	GreenPhos 20	142.32	787.4
21	*t*BuPMeHBF$_4$	67.45	683.8
22	Ad$_2$PBuHI	69.81	675.4

[a] Appeared as a sharp singlet in the ^{31}P NMR spectra flanked by a doublet of ^{77}Se satellite peaks from which the chemical shift and coupling constant is determined.[12] For further details see Spectra S97 and S98.

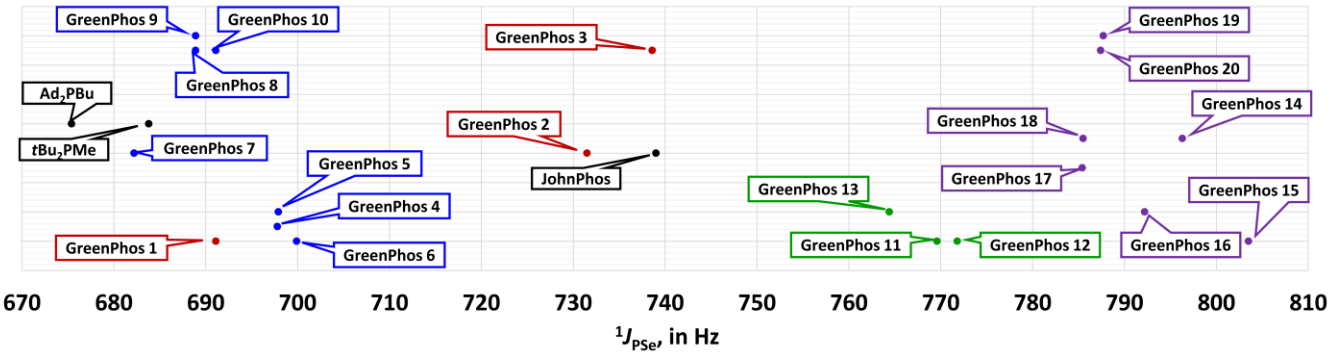

Figure S1. Graphical representation of the phosphorous selenium coupling constants ($^1J_{PSe}$, in Hz) of the selenides of GreenPhos 1-20.

5. The screening of catalytic activity of prepared ligands

5.1. Suzuki-Miyaura cross-coupling reaction

5.1.1. Optimization of the reaction conditions

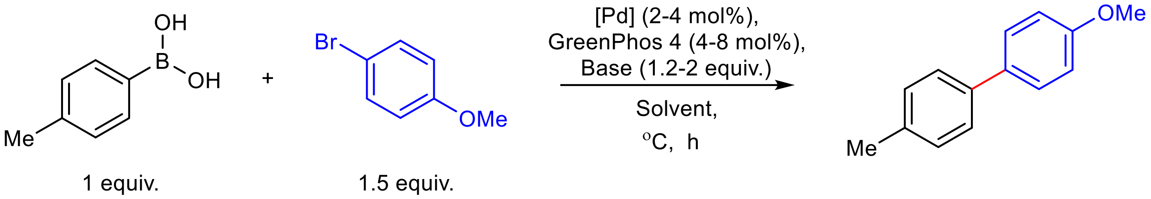

All screening reactions were conducted inside of 10 mL test tubes with standard ground joints. Inside of an argon-filled glovebox *p*-tolylboronic acid (0.368 mmol, 1 equiv.), the corresponding palladium source (2-4 mol%), the base (1.2-2 equiv.) and GreenPhos 4 (4-8 mol%) were weighed into an oven-dried reaction vial. The reaction vial was sealed with a rubber septum, removed from the glovebox, and equipped with an argon-filled balloon. Next, the reaction vial was charged with the corresponding dry and degassed solvent (2 mL) and 4-bromoanisole (0.552 mmol, 1.5 equiv.). The balloon was removed, and the reaction proceeded at 20-80 °C for 2-24h. Afterwards, the reaction mixture was cooled down to room temperature, and the solvent was removed by rotary evaporator. The internal standard, 1,3,5-trimethoxybenzene (1 equiv.), was added to the reaction vial and the resulting mixture was treated with $CDCl_3$ (2 mL). The reaction vial was thoroughly shaken for 2 minutes, and then centrifuged (Figure S2-S9). This was followed by ^{1}H NMR analysis of an aliquot taken from the mixture (Figure S10).

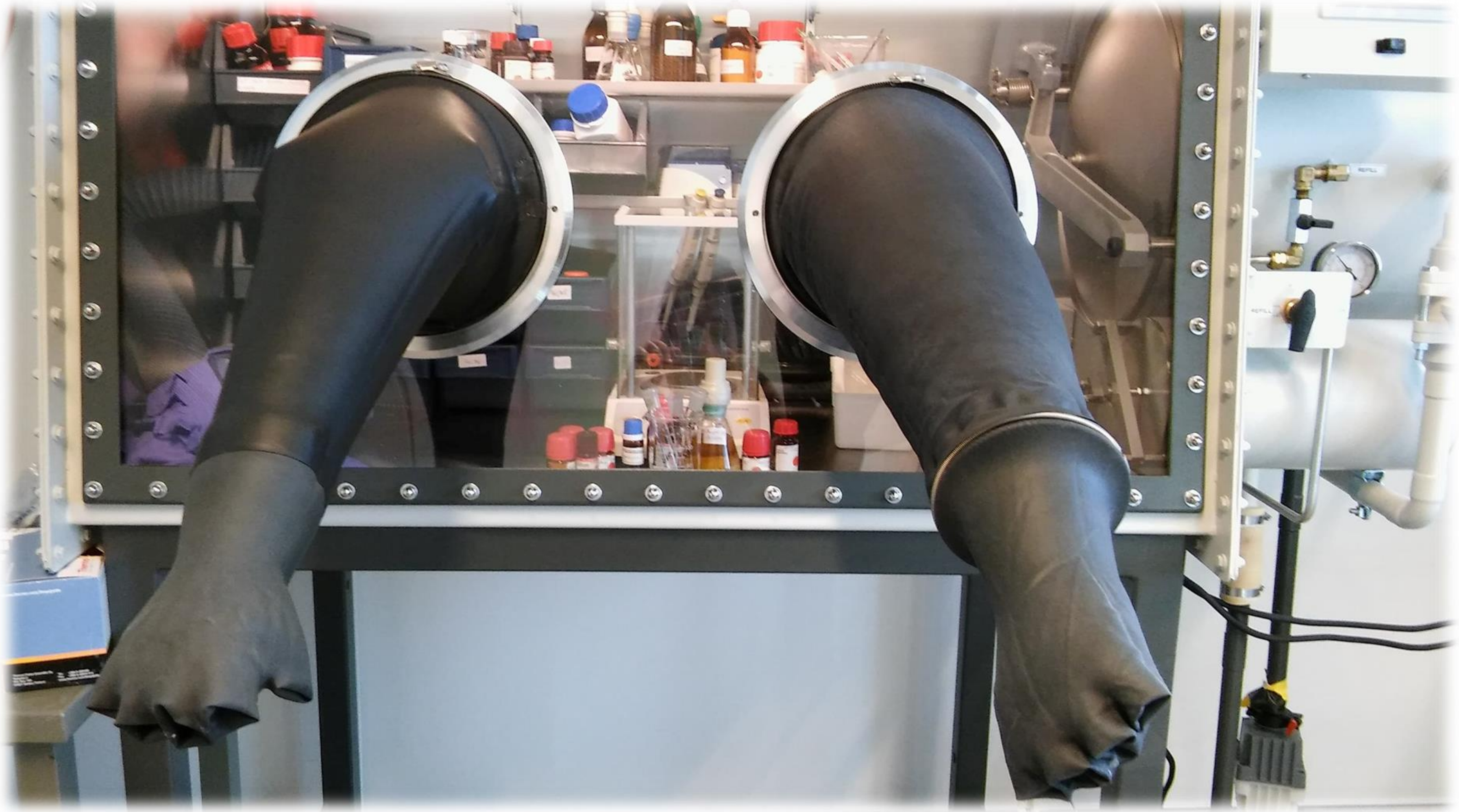

Figure S2. Charging of reaction vials with solid reactants and catalyst in the glovebox.

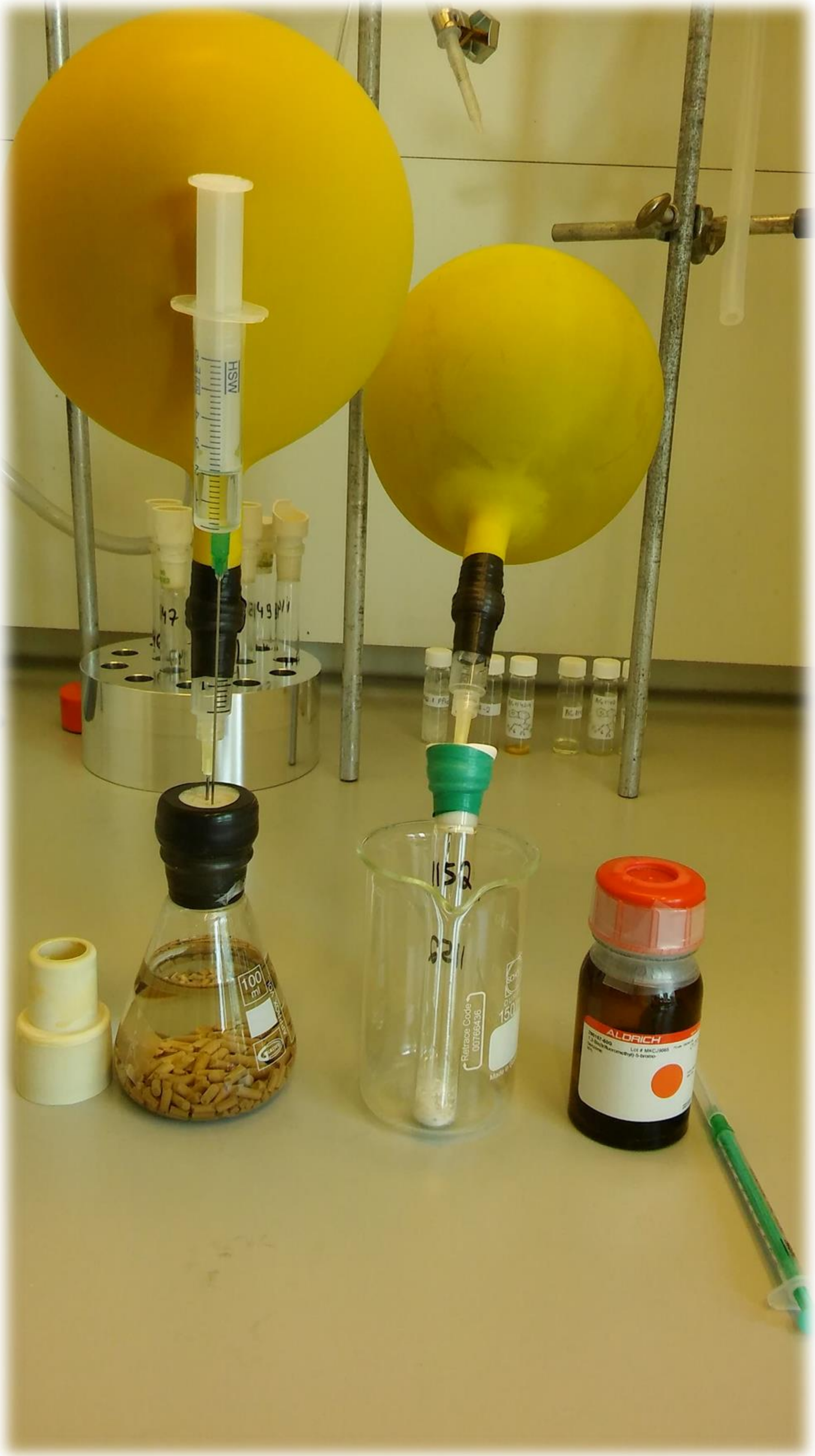

Figure S3. Addition of the solvent and liquid reaction components.

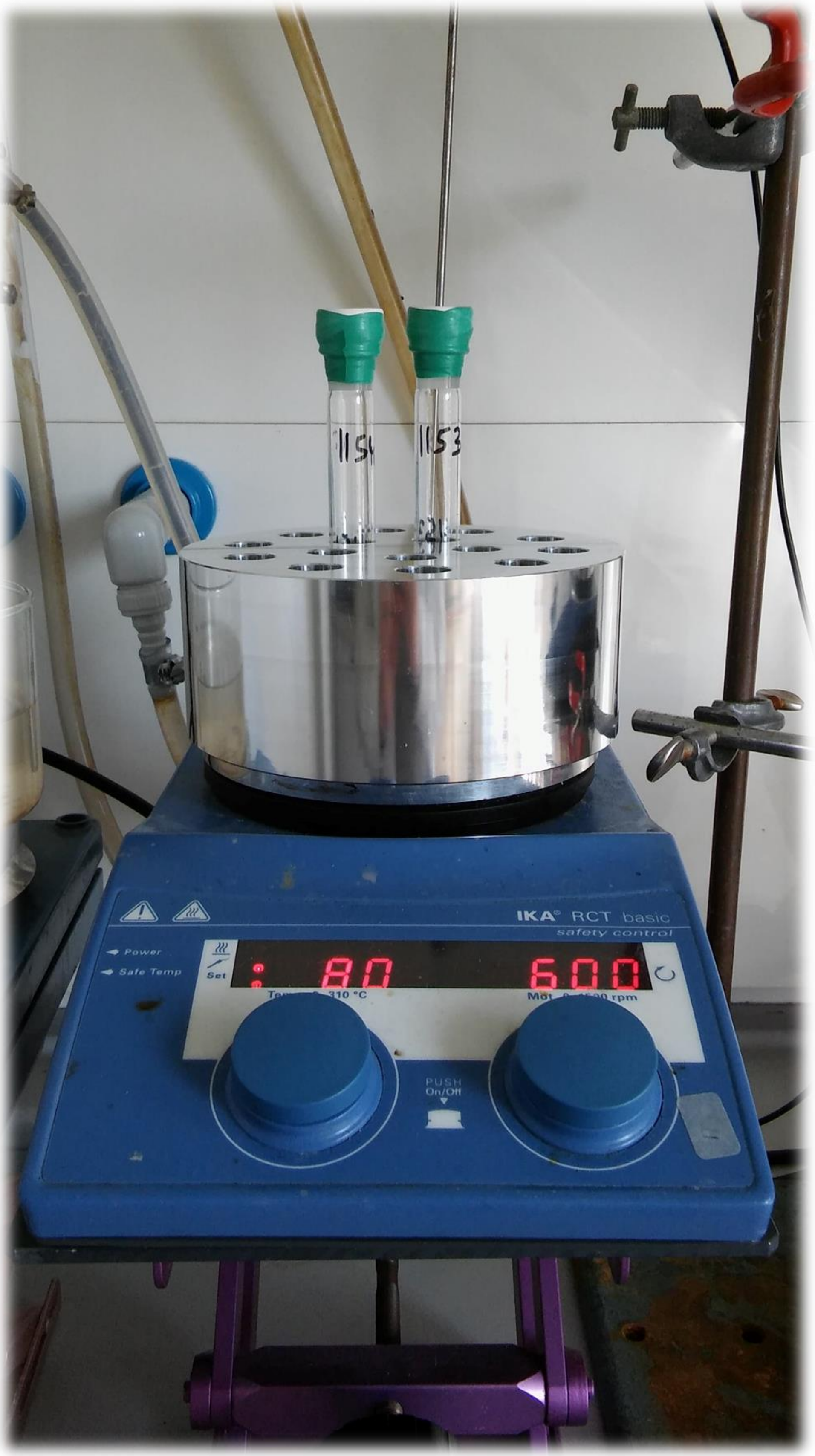

Figure S4. Stirring of the reaction mixture at appropriate temperature.

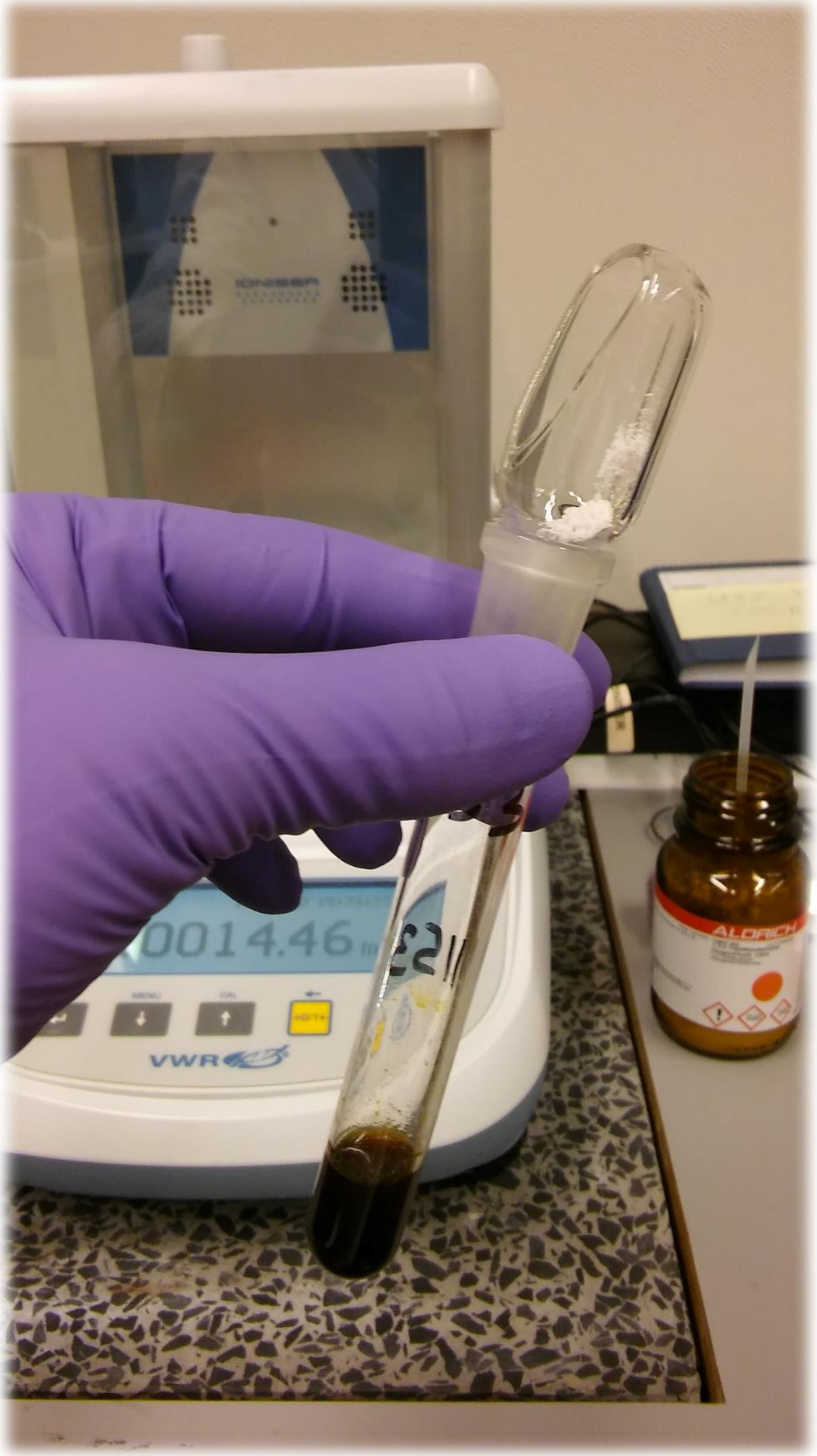

Figure S5. Addition of an internal standard to the reaction mixture at room temperature.

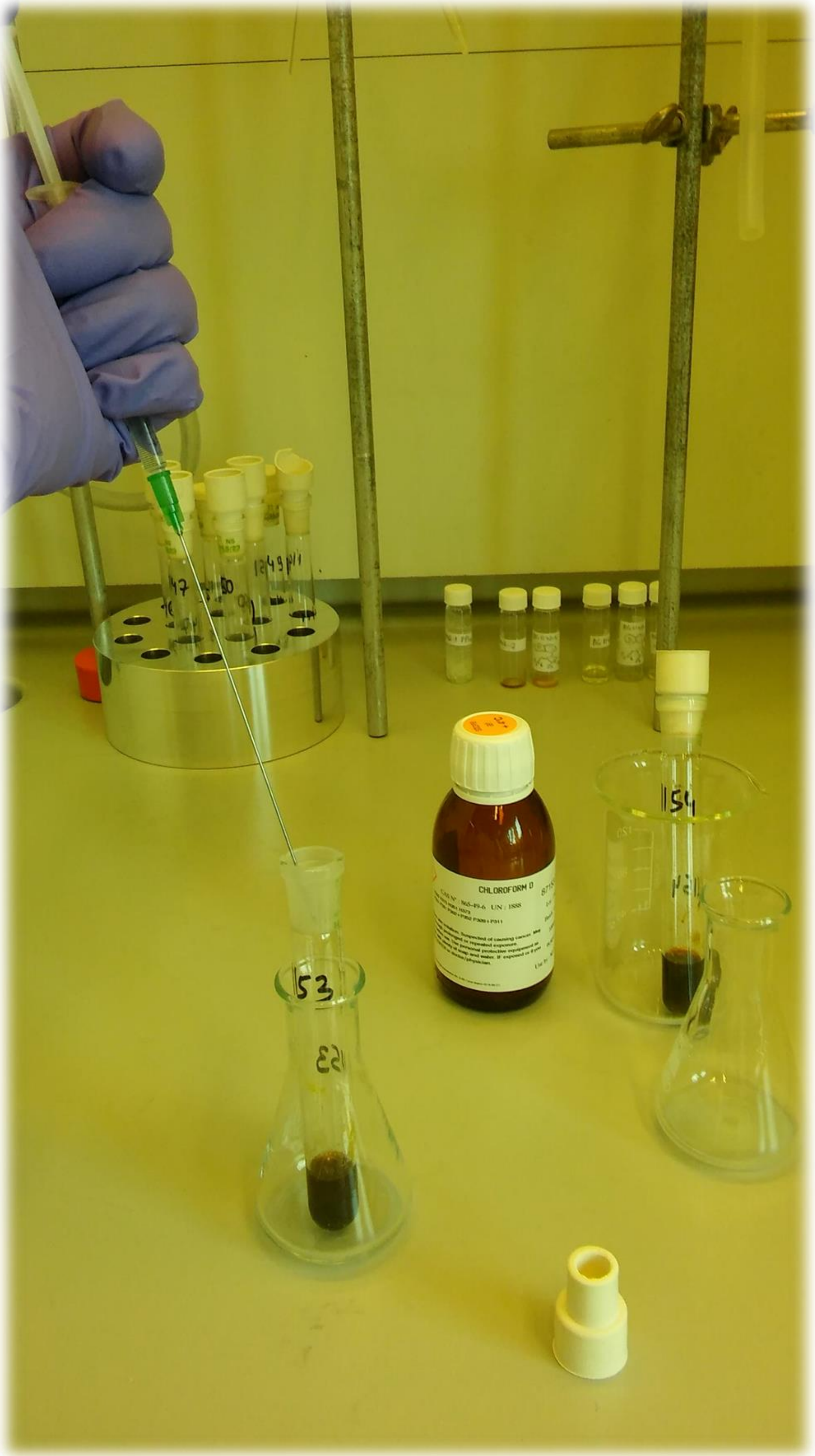

Figure S6. Addition of $CDCl_3$.

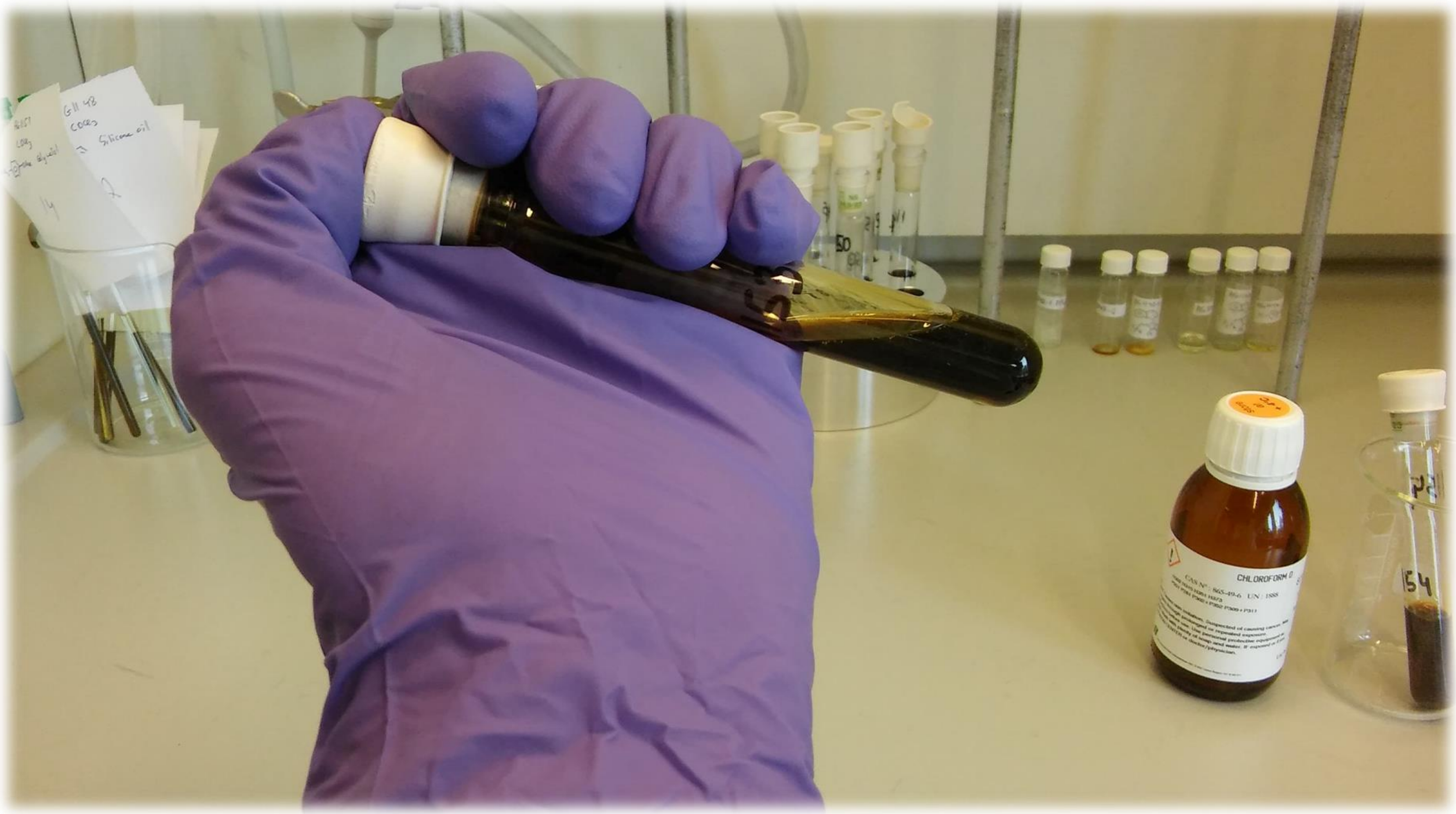

Figure S7. Shaking of the reaction mixture to dissolve all organic matter.

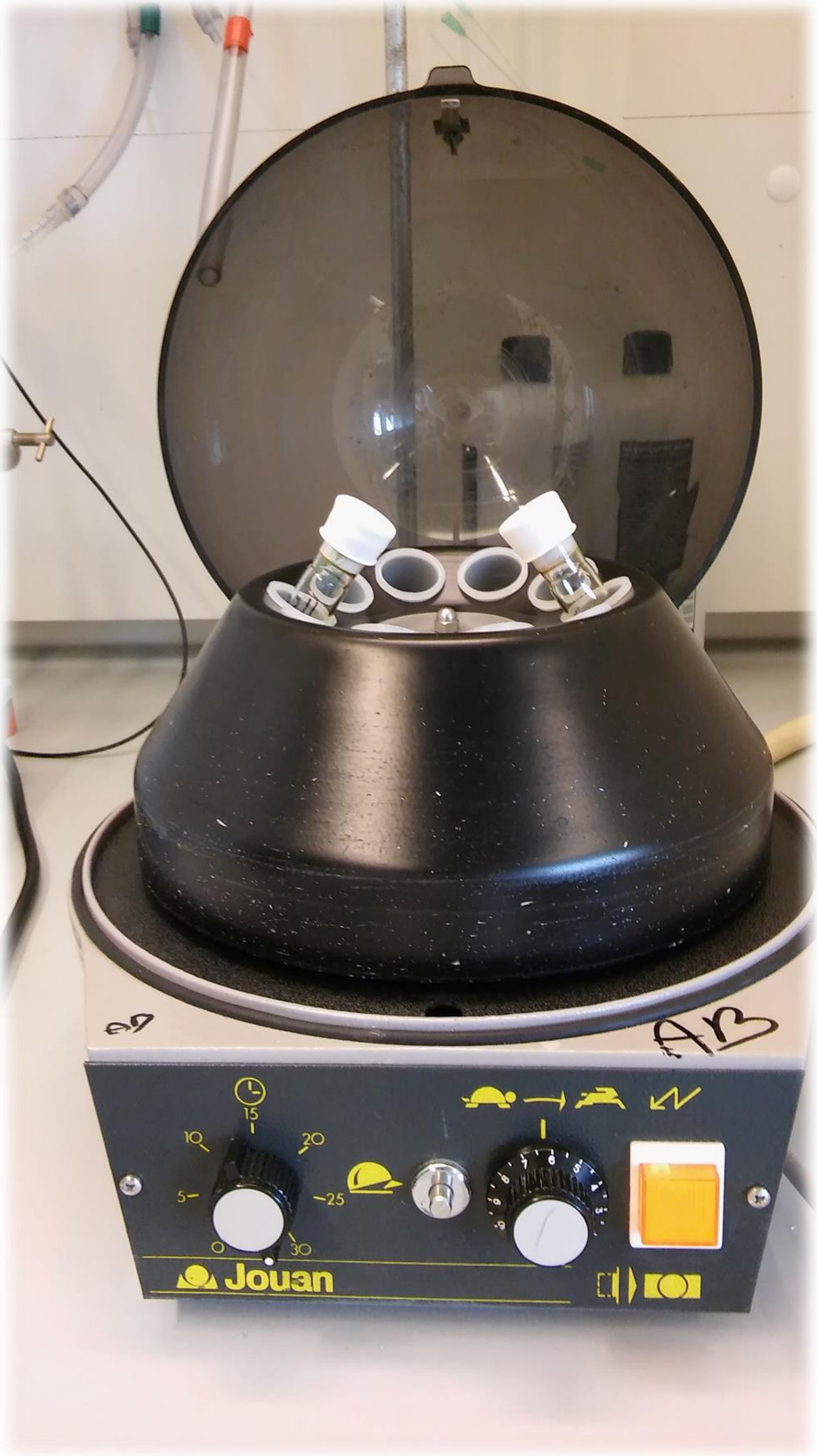

Figure S8. Centrifugation to precipitate the inorganic matter.

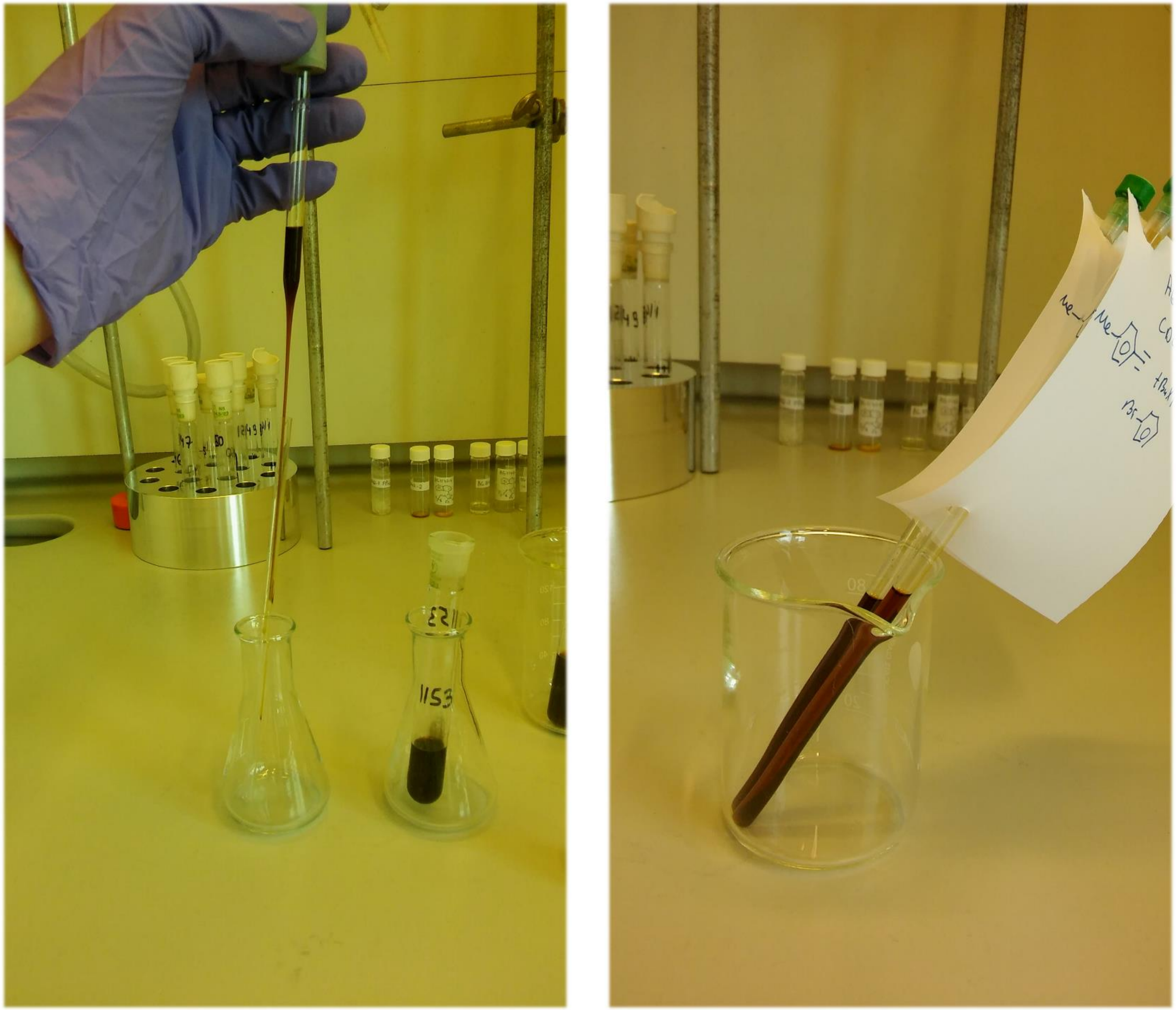

Figure S9. Taking an aliquot for a crude ^{1}H NMR analysis.

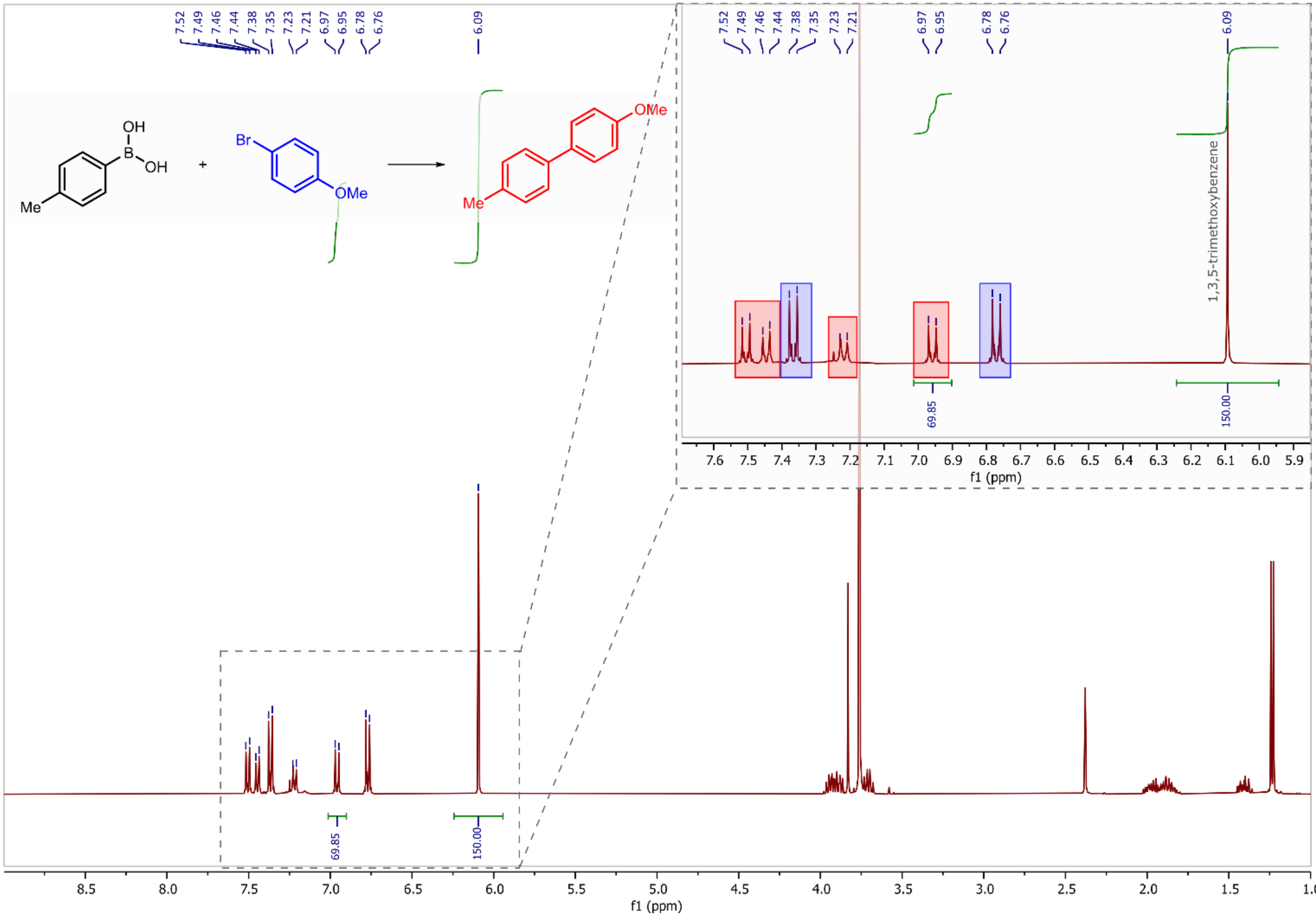

Figure S10. Selected example of a crude ^{1}H NMR for the reaction mixture of a Suzuki-Miyaura coupling between 4-bromoanisole and *p*-tolylboronic acid. The resonance (d, δ_H: 6.97-6.94 ppm, integral range: 7.013-6.902 ppm) of the product (4-methoxy-4'-methyl-1-1'-biphenyl) is indicated in red. The singlet resonance of the internal standard, 1,3,5-trimethoxybenzene was referenced to δ_H: 6.0929 ppm and integrated to 150 protons (integral range: 6.243-5.943 ppm).

Table S2. Screening of Pd precatalysts for Suzuki-Miyaura coupling.

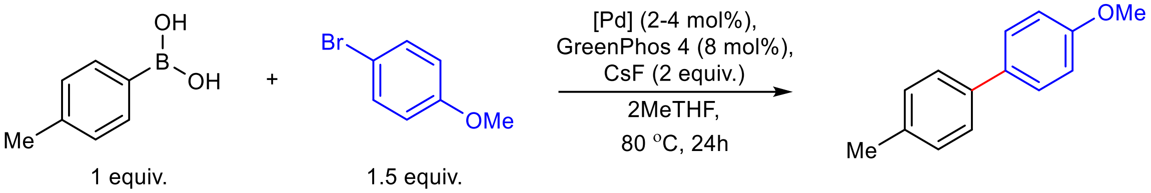

Entry	[Pd] (mol%)	Yield (%)[a]
1	$Pd(OAc)_2$ (4)	100
2	Pd_2dba_3 (2)	100
3	$PdCl_2(MeCN)_2$ (4)	95
4	$[PdCl(C_3H_5)]_2$ (2)	100
5	[(η^3-1-*tert*-Butylindenyl)(μ-Cl)Pd]$_2$ (2)	98
6	[(Cinnamyl)PdCl]$_2$ (2)	100
7	Di-μ-chlorobis[2-[(dimethylamino)methyl]phenyl-C,N]dipalladium(II) (2)	100
8	Di-μ-mesylbis[2′-(amino-*N*)[1,1′-biphenyl]-2-yl-C]dipalladium(II) (Pd G3) (2)	100

[a] Yield determined by ^{1}H NMR analysis in $CDCl_3$ using 1,3,5-trimethoxybenzene as an internal standard.

Table S3. Screening of temperature and duration of Suzuki-Miyaura coupling.

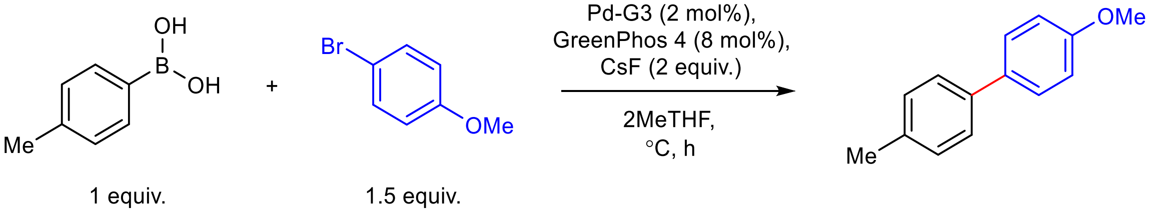

Entry	Temperature (°C)	Time (h)	Yield (%)[a]
1	80	24	100
2	80	6	100
3	80	2	94
4	20	24	70
5	20	6	56
6	40	24	79
7	40	6	62

[a] Yield determined by ^{1}H NMR analysis in $CDCl_3$ using 1,3,5-trimethoxybenzene as an internal standard.

Table S4. Screening of solvents for Suzuki-Miyaura coupling.

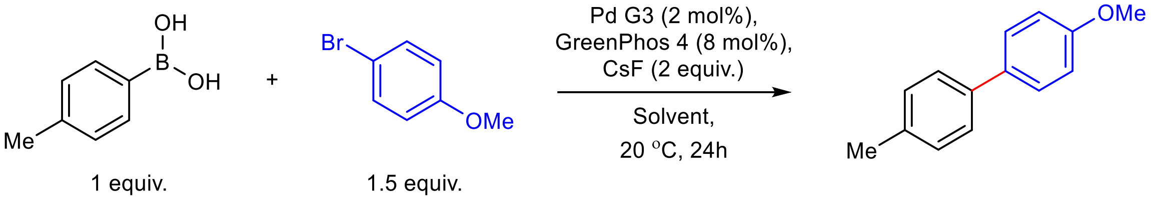

Entry	Solvent (2 mL)	Yield (%)[a]
1	2MeTHF	70
2	Acetal (1,1-diethoxyethane)	30
3	Dioxane	41
4	THF	42
5	DME	78
6	EtOH	60
7	Toluene	31
8	MeCN	45
9	DMF	41
10	Rapeseed oil (Askim)	0
11	Water (2 mL)/TBAB (0.5 equiv.)	0

[a] Yield determined by ^{1}H NMR analysis in $CDCl_3$ using 1,3,5-trimethoxybenzene as an internal standard.

Table S5. Screening of bases for Suzuki-Miyaura coupling.

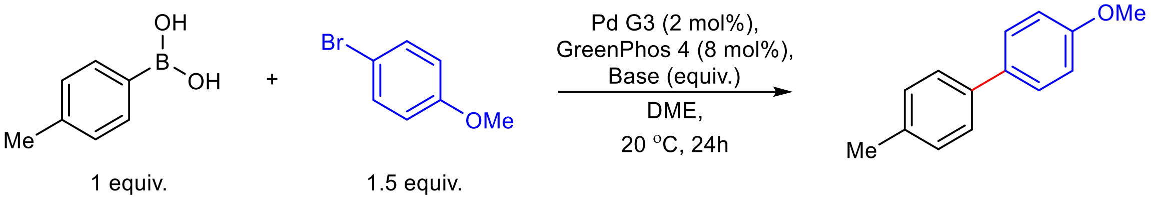

Entry	Base (equiv.)	Yield (%)[a]
1	CsF (2)	78
2	KF (2)	15
3	Cs_2CO_3 (2)	55
4	K_2CO_3 (2)	39
5	K_3PO_4 (2)	43
6	KO*t*Bu (2)	30
7	KOMe (2)	99
8	KOH (2)	100
9	KOH (1.2)	71

[a] Yield determined by ^{1}H NMR analysis in $CDCl_3$ using 1,3,5-trimethoxybenzene as an internal standard.

5.1.2. Screening of phosphonium salts for Suzuki-Miyaura coupling

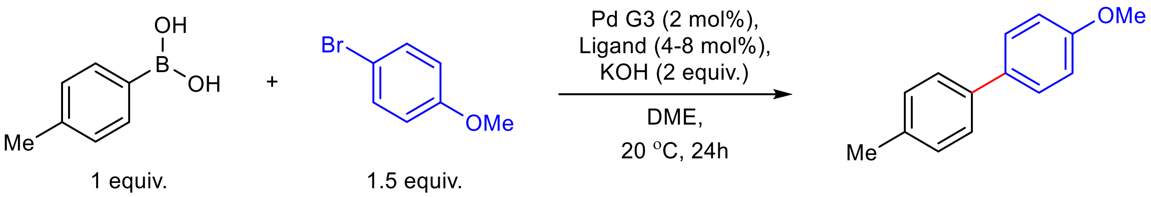

All screening reactions were conducted inside of 10 mL test tubes with standard ground joints. Inside of an argon-filled glovebox *p*-tolylboronic acid (0.368 mmol, 1 equiv.), Pd G3 (2 mol%), KOH (2 equiv.) and the corresponding ligand (4-8 mol%) were weighed into an oven-dried reaction vial. The reaction vial was sealed with a rubber septum, removed from the glovebox, and equipped with an argon-filled balloon. Next, the reaction vial was charged with dry and degassed DME (2 mL) and 4-bromoanisole (1.5 equiv.). The balloon was removed, and the reaction proceeded at 20 °C for 24h. Afterwards, the solvent was removed by rotary evaporator. The internal standard, 1,3,5-trimethoxybenzene (1 equiv.), was added to the reaction vial and the resulting mixture was treated with $CDCl_3$ (2 mL). The reaction vial was thoroughly shaken for 2 minutes, and then centrifuged (Figure S2-S9). This was followed by ^{1}H NMR analysis of an aliquot taken from the mixture (Figure S10).

Table S6. Analysis of the effect of the ligand loading on the performance of the catalytic system.

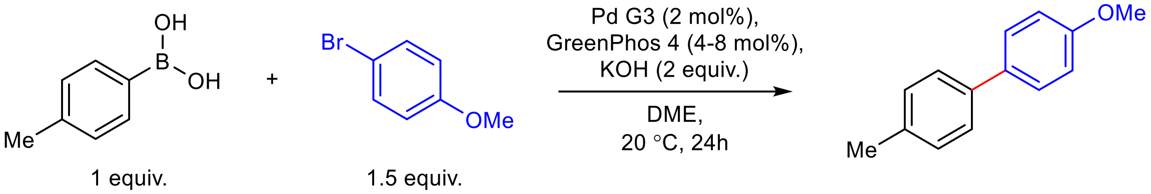

Entry	Ligand (mol%)	Yield (%)[a]
1	GreenPhos 4 (8)	100
2	GreenPhos 4 (4)	100

[a] Yield determined by ^{1}H NMR analysis in $CDCl_3$ using 1,3,5-trimethoxybenzene as an internal standard.

Table S7. Screening of phosphonium salts for Suzuki-Miyaura coupling.

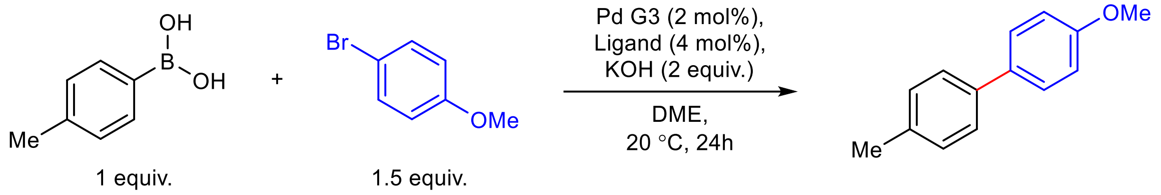

Entry	Ligand (4 mol%)	Yield (%)[a]
1	none	0
2	*t*$Bu_2PMeHBF_4$	19
3	Ad_2PBuHI	32
4	*t*Bu_3PHBF_4	99
5	GreenPhos 1	0
6	GreenPhos 2	0
7	GreenPhos 3	0
8	GreenPhos 4	100
9	GreenPhos 5	100
10	GreenPhos 6	100
11	GreenPhos 7	100
12	GreenPhos 8	100
13	GreenPhos 9	97
14	GreenPhos 10	97

[a] Yield determined by ^1H NMR analysis in $CDCl_3$ using 1,3,5-trimethoxybenzene as an internal standard.

5.1.3. Screening of phosphinites for Suzuki-Miyaura coupling

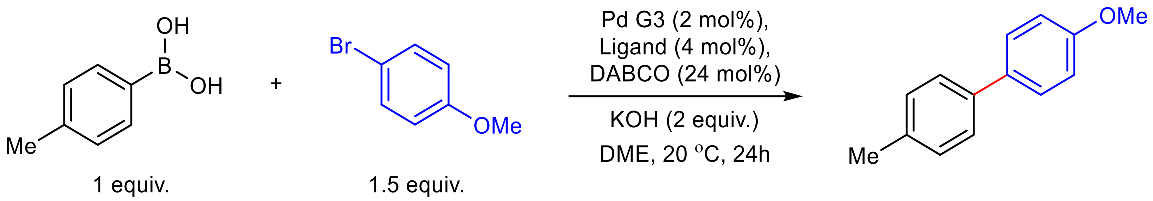

Inside of an argon-filled glovebox an oven dried 10 mL vial was sequentially charged with corresponding phosphinite (4 mol%), DABCO (24 mol%) and anhydrous degassed DME (2 mL). The vial was sealed with a stopper equipped with a septum, removed from the glovebox and stirred at 85 °C for 24h.

The next day, inside of an argon-filled glovebox an oven dried 10 mL flask was charged with Pd G3 (2 mol%), which was followed by addition of the deprotected ligand solution in DME. The vial containing the ligand solution was washed with anhydrous degassed DME (1 mL), which was added to the 10 mL flask. The resulting mixture was allowed to stir at 20 °C for 1 min, which lead to the formation of clear yellowish solution. Subsequently, the flask was sequentially charged with *p*-tolylboronic acid (1 equiv., 0.368 mmol), KOH (2 equiv.) and degassed 4-bromoanisole (1.5 equiv.). The flask was sealed with a rubber septum, removed from the glovebox and the resulting mixture was stirred at 20 °C for 24h. Afterwards, the volatiles were removed by rotary evaporator, which was followed by addition of 1,3,5-trimethoxybenzene (1 equiv., internal standard). The resulting mixture was treated with $CDCl_3$ (2 mL), thoroughly shaken for 2 minutes, and then centrifuged (Figure S2-S9). This was followed by ^{1}H NMR analysis of an aliquot taken from the mixture (Figure S10).

Table S8. Screening of phosphinites for Suzuki-Miyaura coupling.

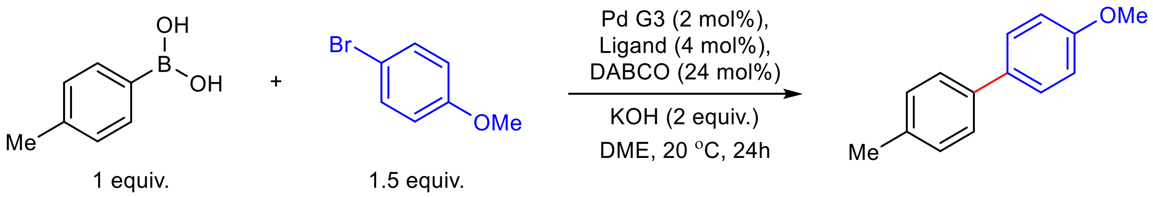

**Entry**	**Ligand (4 mol%)**	**Yield (%)[a]**
1	GreenPhos 11	100
2	GreenPhos 12	100
3	GreenPhos 13	100
4	GreenPhos 14	39
5	GreenPhos 15	97
6	GreenPhos 16	39
7	GreenPhos 17	100
8	GreenPhos 18	45
9	GreenPhos 19	44
10	GreenPhos 20	33

[a] Yield determined by ^{1}H NMR analysis in $CDCl_3$ using 1,3,5-trimethoxybenzene as an internal standard.

5.1.4. Optimization of reaction conditions for chlorobenzene

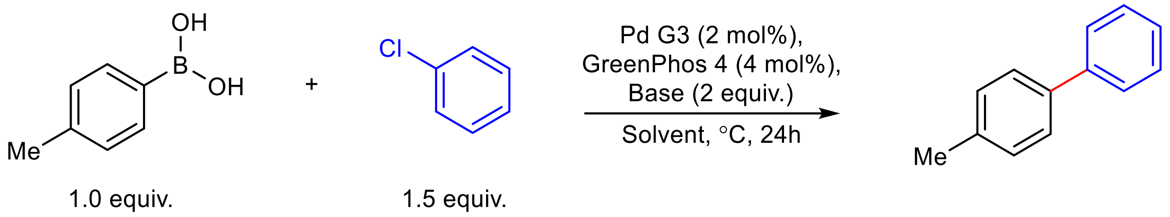

Optimizations of the conditions for chlorobenzene at 80 °C and lower temperatures were conducted inside of 10 mL test tubes with standard ground joints. Screening reactions at 110 °C were conducted inside of 10 mL pressure tubes equipped with corresponding septum, PTFE stabilizer and screw cap. Inside of an argon-filled glovebox *p*-tolylboronic acid (0.368 mmol, 1 equiv.), Pd G3 (2 mol%), corresponding base (2 equiv.) and GreenPhos 4 (4 mol%) were weighed into an oven-dried reaction vial. The reaction vial was sealed with a rubber septum, removed from the glovebox, and equipped with an argon-filled balloon. Next, the reaction vial was charged with the corresponding dry and degassed solvent (2 mL) and chlorobenzene (1.5 equiv.). The balloon was removed, and the reaction proceeded at 20-110 °C for 24h. Afterwards, the reaction mixture was cooled down to room temperature, and the solvent was removed by rotary evaporator. The internal standard, 1,3,5-trimethoxybenzene (1 equiv.), was added to the reaction vial and the resulting mixture was treated with $CDCl_3$ (2 mL). The reaction vial was thoroughly shaken for 2 minutes, and then centrifuged (Figure S2-S9). This was followed by ^{1}H NMR analysis of an aliquot taken from the mixture (Figure S10).

Table S9. Optimization of reaction conditions for chlorobenzene.

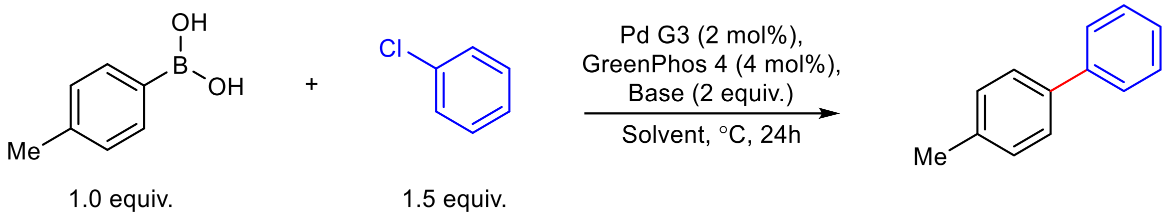

Entry	Base (2 equiv.)	Solvent (2 mL)	°C	Yield (%)[a]
1	KOH	Dioxane	80	60
2	KOMe	Dioxane	80	100
3	KOMe	Dioxane	40	86
4	KOMe	Dioxane	20	69
5	CsF	DME	80	30
6	CsF	Dioxane	80	59
7	CsF	2MeTHF	80	30
8	CsF	EtOH	80	5
9	CsF	Toluene/H_2O 4/1	80	0
10	Cs_2CO_3	Dioxane	80	28
11	CsOAc	Dioxane	80	7
12	K_2CO_3	Dioxane	80	6
13	K_3PO_4	Dioxane	80	5
14	TBAF x $3H_2O$	Dioxane	80	65
15	TBAF x $3H_2O$	Dioxane	110	100

[a] Yield determined by ^{1}H NMR analysis in $CDCl_3$ using 1,3,5-trimethoxybenzene as an internal standard.

5.1.5. Estimation of the functional group tolerance of Suzuki-Miyaura coupling

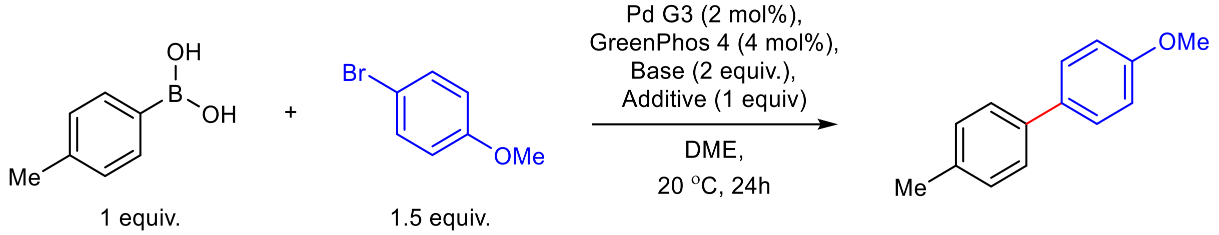

All screening reactions were conducted inside of 10 mL test tubes with standard ground joints. Inside of an argon-filled glovebox *p*-tolylboronic acid (0.368 mmol, 1 equiv.), Pd G3 (2 mol%), the corresponding base (2 equiv.) and GreenPhos 4 (4 mol%) were weighed into an oven-dried reaction vial. In the case of solid additives (1 equiv.), these were weighed into the reaction vials prior to the introduction of the reaction vail to the glovebox. Liquid additives (1 equiv.) were added to the reaction vials after the addition of DME and 4-bromoanisole. The reaction vial was sealed with a rubber septum, removed from the glovebox, and equipped with an argon-filled balloon. Next, the reaction vial was charged with dry and degassed DME (2 mL) and 4-bromoanisole (1.5 equiv.). The balloon was removed, and the reaction proceeded at 20 °C for 24h. Afterwards, the solvent was removed by rotary evaporator. The internal standard, 1,3,5-trimethoxybenzene (1 equiv.), was added to the reaction vial and the resulting mixture was treated with $CDCl_3$ (2 mL). The reaction vial was thoroughly shaken for 2 minutes, and then centrifuged (Figure S2-S9). This was followed by ^{1}H NMR analysis of an aliquot taken from the mixture (Figure S10).

Table S10. Robustness assessment for Suzuki-Miyaura coupling.

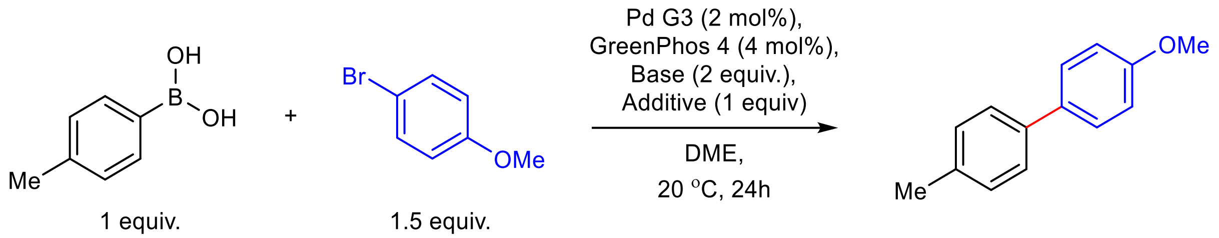

Entry	Additive (1 equiv.)	Base (2 equiv.)	Yield (%)[a]	Additive remaining (%)[a]
1	none	KOH	100	/
2	Air	KOH	32	/
3	H_2O (4.5 equiv.)	KOH	15	/
4	Glycine anhydride	KOH	35	/
5	Dicyclohexylamine	KOH	77	100
6	1,4-Dicyanobenzene	KOH	7	70
7	1,4-Dinitrobenzene	KOH	17	99
8	Imidazole	KOH	24	64
9	2,6-Lutidine	KOH	50	38
10	Menthol	KOH	100	91
11	2,6-Dimethoxyphenol[b]	KOH	50	100
12	Palmitic acid[b]	KOH	45	100
13	Methyl 3,5-dinitrobenzoate	KOH	41	10
14	2-Cyclohexen-1-one	KOH	48	/
15	2-Cyclohexen-1-one	KOH	15[c]	/
16	*cis*-Cyclooctene	KOH	17	/
17	1-Nonyne	KOH	16	54
18	1-Nonyne	KOH	7[c]	56[c]
19	Ethyl 3-chloropropionate	KOH	53	/
20	Cyclohexanecarboxaldehyde	KOH	100	11
21	Cyclohexyl methyl ketone	KOH	45	30
22	Dimethyl sulfone	KOH	38	59
23	(1*S*)-(−)-Verbenone	KOH	71	56
24	Camphene	KOH	100	/
25	none	TBAF x $3H_2O$	98	/
26	1,4-Dicyanobenzene	TBAF x $3H_2O$	83	98
27	1,4-Dinitrobenzene	TBAF x $3H_2O$	75	92
28	Methyl 3,5-dinitrobenzene	TBAF x $3H_2O$	67	95
29	Cyclohexyl methyl ketone	TBAF x $3H_2O$	81	89
30	Ethyl 3-chloropropionate	TBAF x $3H_2O$	72	87

[a] Yield determined by ^1H NMR analysis in $CDCl_3$ using 1,3,5-trimethoxybenzene as an internal standard. [b] The remaining of phenol and carboxylic acid were determined following acidification of the reaction mixture. [c] The reaction was performed with cataCXium® A instead of GreenPhos 4.

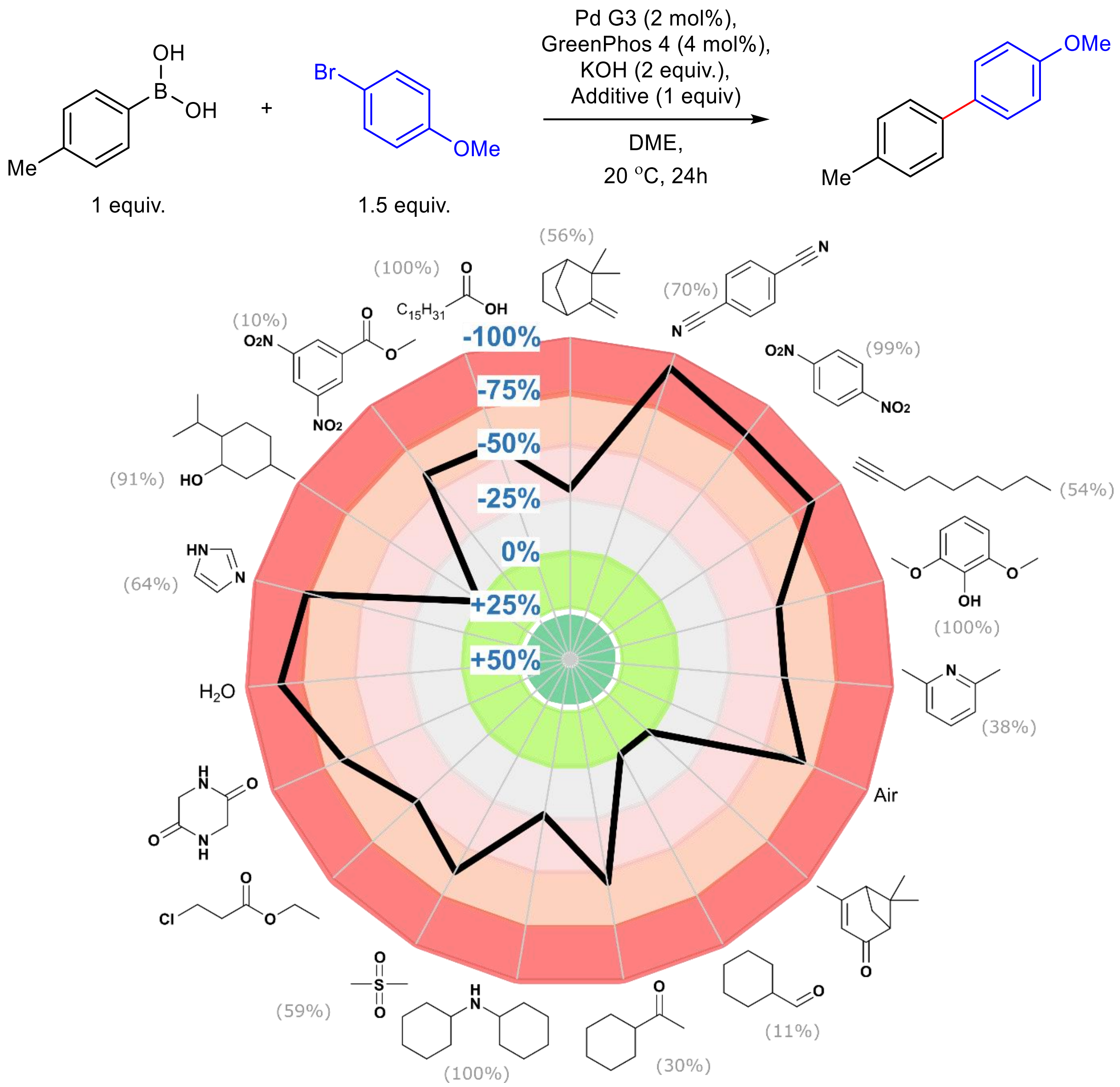

Figure S11. Robustness assessment radar diagram showing the effect of various additives on the yield of Suzuki-Miyaura coupling performed in the presence of KOH (2 equiv.) used as a base. Yields are determined by ^1H NMR analysis in $CDCl_3$ using 1,3,5-trimethoxybenzene as an internal standard. Numbers in the brackets indicate the percentages of recovered additives.

5.2. Stille cross-coupling reaction

5.2.1. Optimization of the reaction conditions

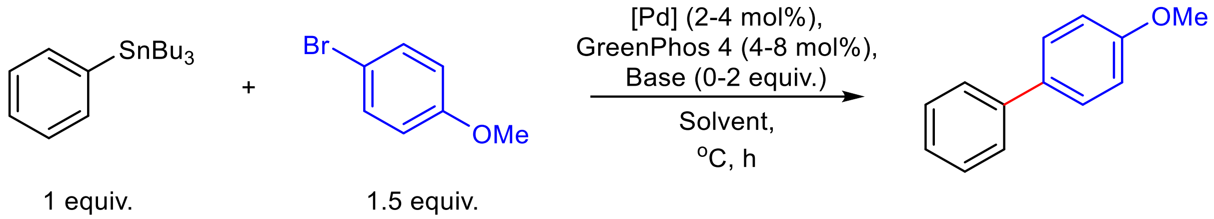

All screening reactions were conducted inside of 10 mL test tubes with standard ground joints. Inside of an argon-filled glovebox the corresponding palladium source (2-4 mol%), base (0-2 equiv.) and GreenPhos 4 (4-8 mol%) were weighed into an oven-dried reaction vial. The reaction vial was sealed with a rubber septum, removed from the glovebox, and equipped with an argon-filled balloon. This was followed by addition of the corresponding dry and degassed solvent (2 mL), tributylphenylstannane (0.272 mmol, 1.0 equiv.) and 4-bromoanisole (1.5 equiv.). The balloon was removed, and the reaction proceeded at 20-80 °C for 2-24h. Afterwards, the reaction mixture was cooled down to room temperature, and the solvent was removed by rotary evaporator. The internal standard, 1,3,5-trimethoxybenzene (1 equiv.), was added to the reaction vial and the resulting mixture was treated with $CDCl_3$ (2 mL). The reaction vial was thoroughly shaken for 2 minutes, and then centrifuged (Figure S2-S9). This was followed by ^{1}H NMR analysis of an aliquot taken from the mixture (Figure S12).

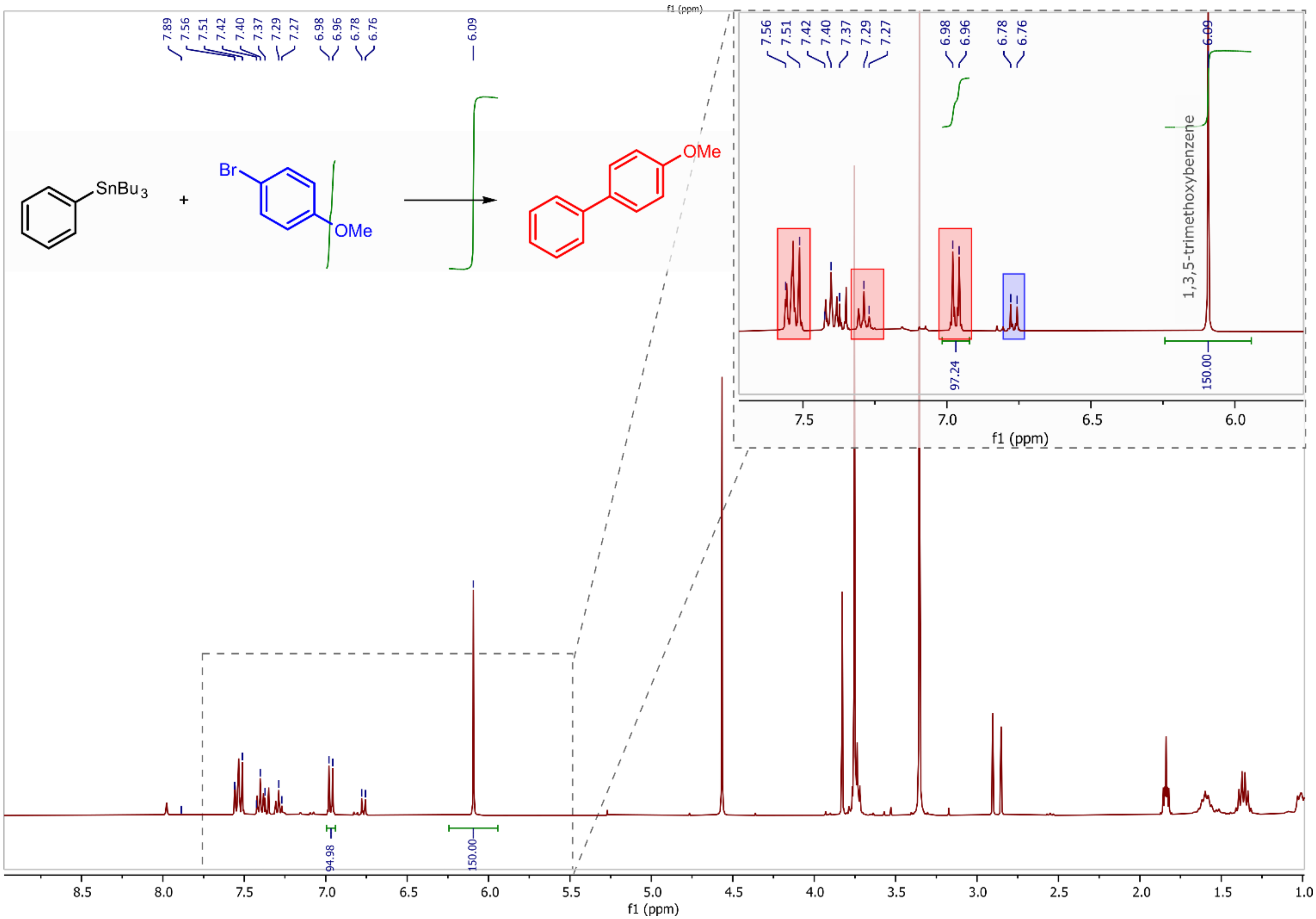

Figure S12. Selected example of a crude ^{1}H NMR for the reaction mixture of a Stille coupling between 4-bromoanisole (blue) and tributylphenylstannane. The resonance (d, δ_H: 6.98-6.96 ppm, integral range: 6.995-6.940 ppm) of the product (4-methoxy-1-1'-biphenyl) is indicated in red. The singlet resonance of the internal standard, 1,3,5-trimethoxybenzene, was referenced to δ_H: 6.0929 ppm and integrated to 150 protons (integral range: 6.243-5.943 ppm).

Table S11. Screening of Pd precatalysts for Stille coupling.

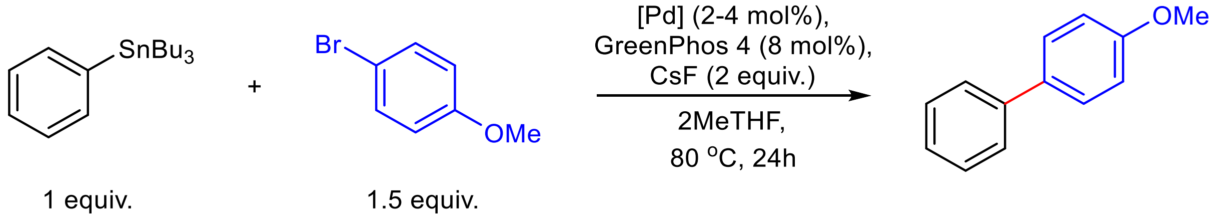

Entry	[Pd] (mol%)	Yield (%)[a]
1	$Pd(OAc)_2$ (4)	97
2	Pd_2dba_3 (2)	95
3	$PdCl_2(MeCN)_2$ (4)	98
4	$[PdCl(C_3H_5)]_2$ (2)	80
5	$[(\eta^3$-1-*tert*-Butylindenyl)(μ-Cl)Pd$]_2$ (2)	96
6	[(Cinnamyl)PdCl$]_2$ (2)	100
7	Di-μ-chlorobis[2-[(dimethylamino)methyl]phenyl-C,N]dipalladium(II) (2)	95
8	Di-μ-mesylbis[2′-(amino-*N*)[1,1′-biphenyl]-2-yl-C]dipalladium(II) (Pd G3) (2)	98

[a] Yield determined by ^{1}H NMR analysis in $CDCl_3$ using 1,3,5-trimethoxybenzene as an internal standard.

Table S12. Analysis of the temperature and duration on the performance of Stille coupling.

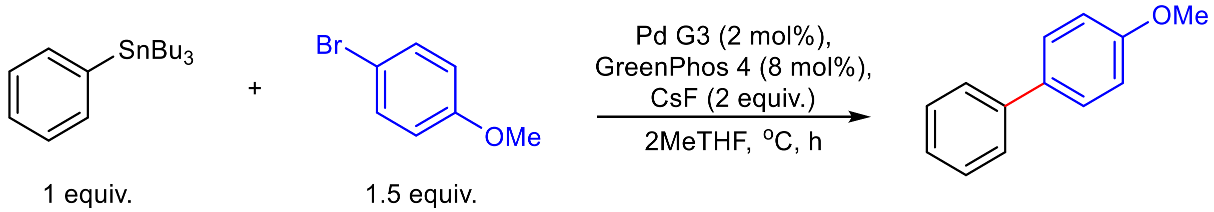

Entry	Temperature (°C)	Time (h)	Yield (%)[a]
1	80	24	98
2	80	6	100
3	80	2	0
4	20	24	18
5	20	6	14
6	40	24	32
7	40	6	23

[a] Yield determined by ^{1}H NMR analysis in $CDCl_3$ using 1,3,5-trimethoxybenzene as an internal standard.

Table S13. Screening of solvents for Stille coupling.

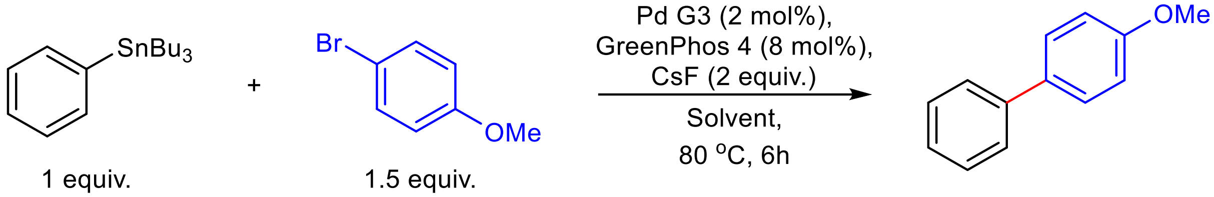

Entry	Solvent (2 mL)	Yield (%)[a]
1	2MeTHF	100
2	Acetal (1,1-diethoxyethane)	5
3	Dioxane	100
4	THF	97
5	DME	95
6	EtOH	68
7	Toluene	50
8	MeCN	100
9	DMF	62
10	Rapeseed oil (Askim)	7

[a] Yield determined by ^1H NMR analysis in $CDCl_3$ using 1,3,5-trimethoxybenzene as an internal standard.

Table S14. Screening of bases for Stille coupling.

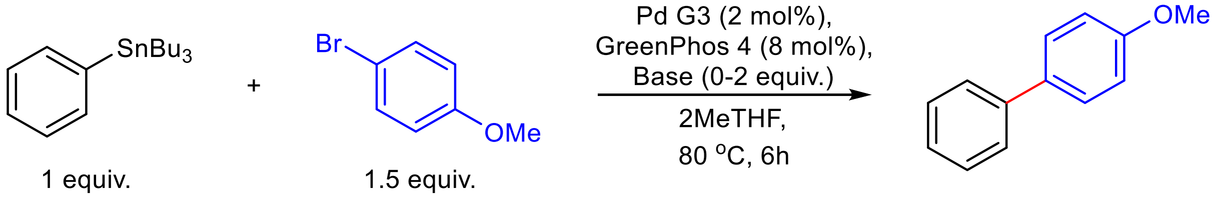

Entry	Base (equiv.)	Yield (%)[a]
1	None	0
2	CsF (2)	100
3	KF (2)	0
4	TBAF x $3H_2O$ (2)	100
5	Cs_2CO_3 (2)	29
6	K_2CO_3 (2)	36
7	KO*t*Bu (2)	20
8	KOMe (2)	0
9	CsF (1.2)	95
10	CsF (0.08)	0

[a] Yield determined by ^1H NMR analysis in $CDCl_3$ using 1,3,5-trimethoxybenzene as an internal standard.

5.2.2. Screening of phosphonium salts for Stille coupling

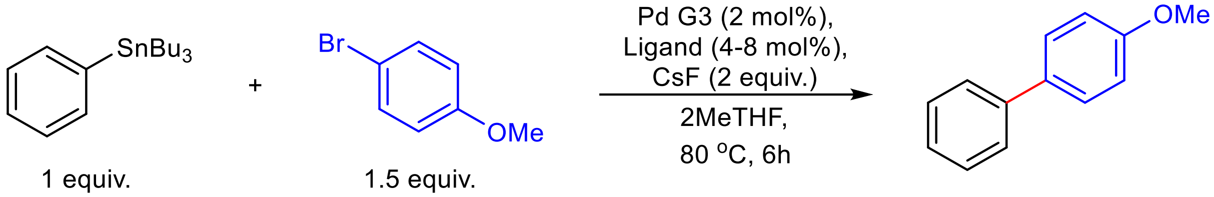

All screening reactions were conducted inside of 10 mL test tubes with standard ground joints. Inside of an argon-filled glovebox Pd G3 (2 mol%), CsF (2 equiv.) and the corresponding ligand (4-8 mol%) were weighed into an oven-dried reaction vial. The reaction vial was sealed with a rubber septum, removed from the glovebox, and equipped with an argon-filled balloon. This was followed by the sequential addition of dry and degassed 2MeTHF (2 mL), 4-bromoanisole (1.5 equiv.) and tributylphenylstannane (0.272 mmol, 1.0 equiv.). The balloon was removed, and the reaction proceeded at 80 °C for 6h. Afterwards, the reaction mixture was cooled down to room temperature, and the solvent was removed by rotary evaporator. The internal standard, 1,3,5-trimethoxybenzene (1 equiv.), was added to the reaction vial and the resulting mixture was treated with $CDCl_3$ (2 mL). The reaction vial was thoroughly shaken for 2 minutes, and then centrifuged (Figure S2-S9). This was followed by ^1H NMR analysis of an aliquot taken from the mixture (Figure S12).

Table S15. Analysis of the effect of the ligand loading on the performance of the catalytic system.

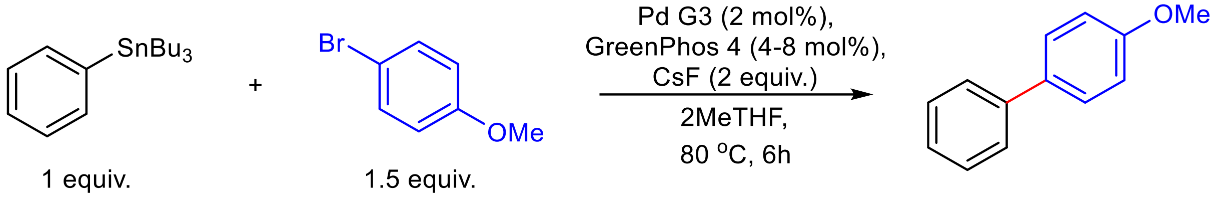

Entry	Ligand (mol%)	Yield (%)[a]
1	GreenPhos 4 (8)	100
2	GreenPhos 4 (4)	100

[a] Yield determined by ^1H NMR analysis in $CDCl_3$ using 1,3,5-trimethoxybenzene as an internal standard.

Table S16. Screening of phosphonium salts for Stille coupling.

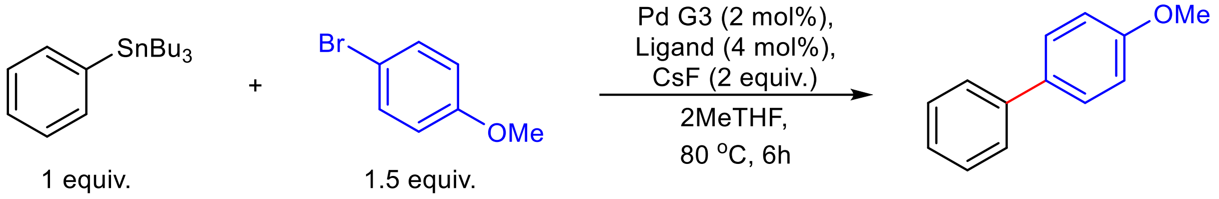

Entry	Ligand (4 mol%)	Yield (%)[a]
1	none	7
2	$t\mathrm{Bu_2PMeHBF_4}$	13
3	$\mathrm{Ad_2PBuHI}$	100
4	$t\mathrm{Bu_3PHBF_4}$	14
5	GreenPhos 1	76
6	GreenPhos 2	79
7	GreenPhos 3	100
8	GreenPhos 4	100
9	GreenPhos 5	85
10	GreenPhos 6	90
11	GreenPhos 7	88
12	GreenPhos 8	89
13	GreenPhos 9	100
14	GreenPhos 10	91

[a] Yield determined by ^{1}H NMR analysis in $CDCl_3$ using 1,3,5-trimethoxybenzene as an internal standard.

5.2.3. Screening of phosphinites for Stille coupling

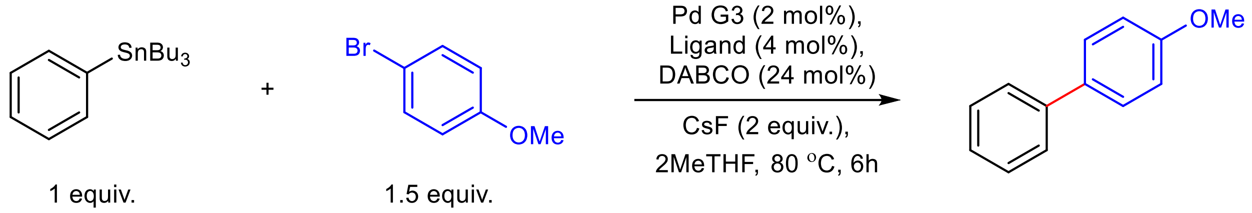

Inside of an argon-filled glovebox an oven dried 10 mL vial was sequentially charged with suitable ligand (4 mol%), DABCO (24 mol%) and anhydrous degassed 2MeTHF (2 mL). The vial was sealed with a stopper equipped with a septum, removed from the glovebox and stirred at 85 °C for 24h.

The next day, inside of an argon-filled glovebox an oven dried 10 mL flask was charged with Pd G3 (2 mol%), which was followed by addition of deprotected ligand solution in 2MeTHF. The vial containing the ligand solution was washed with anhydrous degassed 2MeTHF (1 mL), which was added to the 10 mL flask. The resulting mixture was allowed to stir at 20 °C for 1 min, which lead to the formation of clear yellowish solution. Subsequently, the flask was sequentially charged with degassed tributylphenylstannane (1 equiv., 0.354 mmol), CsF (2 equiv.) and degassed 4-bromoanisole (1.5 equiv.). The flask was sealed with a rubber septum, removed from the glovebox and the resulting mixture was stirred at 80 °C for 6h. Afterwards, the reaction mixture was cooled down to room temperature, which was followed by the removal of volatiles by rotary evaporator and addition of 1,3,5-trimethoxybenzene (1 equiv., internal standard). The resulting mixture was treated with $CDCl_3$ (2 mL), thoroughly shaken for 2 minutes, and then centrifuged (Figure S2-S9). This was followed by ^1H NMR analysis of an aliquot taken from the mixture (Figure S12).

Table S17. Screening of phosphinites for Stille coupling.

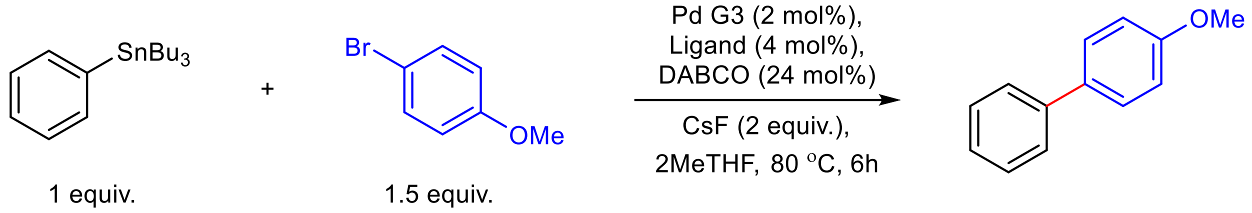

Entry	Ligand (4 mol%)	Yield (%)[a]
1	GreenPhos 11	100
2	GreenPhos 12	54
3	GreenPhos 13	100
4	GreenPhos 14	13
5	GreenPhos 15	50
6	GreenPhos 16	11
7	GreenPhos 17	10
8	GreenPhos 18	13
9	GreenPhos 19	12
10	GreenPhos 20	8

[a] Yield determined by ^1H NMR analysis in $CDCl_3$ using 1,3,5-trimethoxybenzene as an internal standard.

5.2.4. Optimization of Stille coupling for 4-chlorotoluene

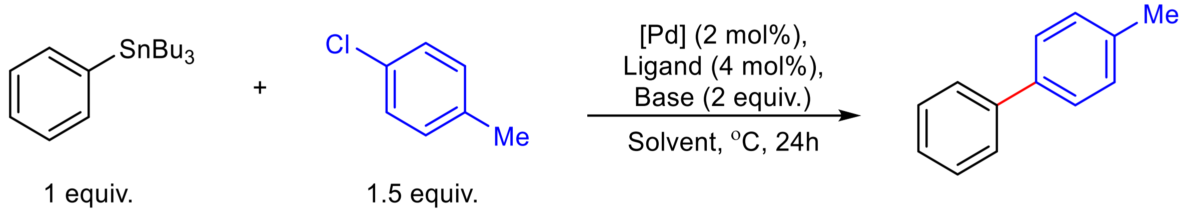

Screening reactions for 4-chlorotoluene were conducted inside of 10 mL pressure tubes equipped with corresponding septum, PTFE stabilizer and screw cap. Inside of an argon-filled glovebox the appropriate palladium source (2 mol%), suitable base (2 equiv.) and the corresponding ligand (4 mol%) were weighed into an oven-dried pressure tube. The pressure tube was sealed with a screw cap, removed from the glovebox, and equipped with an argon-filled balloon. This was followed by addition of the corresponding dry and degassed solvent (2 mL) 4-chlorotoluene (1.5 equiv.) and tributylphenylstannane (0.272 mmol, 1.0 equiv.). The balloon was removed, and the reaction proceeded at 80-110 °C for 24h. Afterwards, the reaction mixture was cooled down to room temperature, and the solvent was removed by rotary evaporator. The internal standard, 1,3,5-trimethoxybenzene (1 equiv.), was added to the reaction vial and the resulting mixture was treated with $CDCl_3$ (2 mL). The reaction vial was thoroughly shaken for 2 minutes, and then centrifuged (Figure S2-S9). This was followed by ^{1}H NMR analysis of an aliquot taken from the mixture (Figure S12).

Table S18. Optimization of Stille coupling for 4-chlorotoluene.

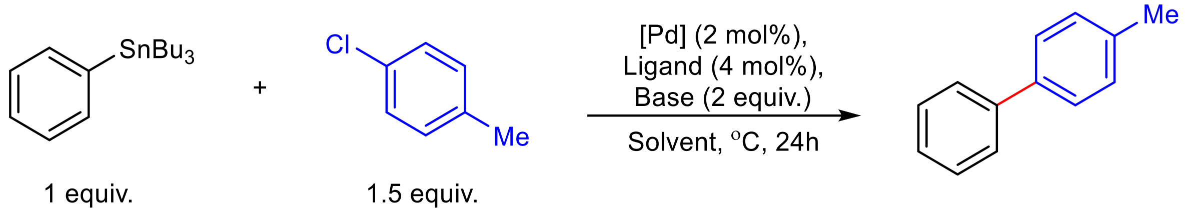

Entry	[Pd] (2 mol%)	Ligand (4 mol%)	Base (2 equiv.)	Solvent	°C	Yield (%)[a]
1	Pd G3	GreenPhos 4	CsF	2MeTHF	80	23
2	$[(Cinnamyl)PdCl]_2$	GreenPhos 4	CsF	2MeTHF	80	5
3	Pd G3	GreenPhos 4	CsF	DME	80	74
4	Pd G3	GreenPhos 4	CsF	MeCN	80	13
5	Pd G3	GreenPhos 4	TBAF x $3H_2O$	2MeTHF	80	41
6	Pd G3	GreenPhos 7	CsF	2MeTHF	80	50
7	Pd G3	GreenPhos 10	CsF	2MeTHF	80	45
8	Pd G3	GreenPhos 3	CsF	2MeTHF	80	24
9	Pd G3	GreenPhos 4	CsF	DME	110	95
10	Pd G3	GreenPhos 4	CsF	DME	110	66[b]
11	Pd G3	GreenPhos 7	CsF	DME	110	38

[a] Yield determined by ^1H NMR analysis in $CDCl_3$ using 1,3,5-trimethoxybenzene as an internal standard.
[b] The reaction proceeded for 6h.

5.2.5. Estimation of the functional group tolerance of Stille coupling

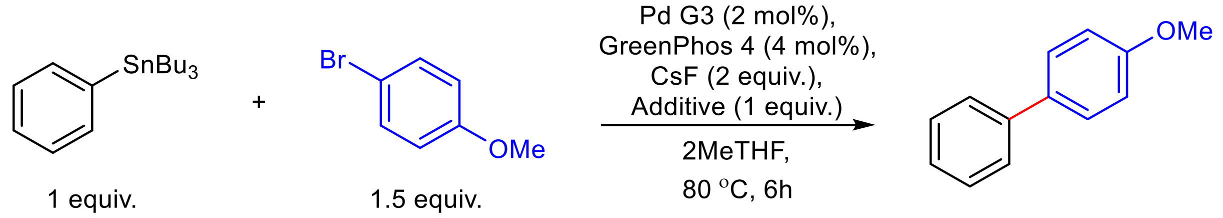

All reactions were conducted inside of 10 mL test tubes with standard ground joints. Inside of an argon-filled glovebox Pd G3 (2 mol%), CsF (2 equiv.) and GreenPhos 4 (4 mol%) were weighed into an oven-dried reaction vial. In the case of solid additives (1 equiv.), these were weighed into the reaction vials prior to the introduction of the reaction vails to the glovebox. Liquid additives (1 equiv.) were added to the reaction vials after the addition of solvent, tributylphenylstannane and 4-bromoanisole. The reaction vial was sealed with a rubber septum, removed from the glovebox, and equipped with an argon-filled balloon. This was followed by addition of dry and degassed 2MeTHF (2 mL), 4-bromoanisole (1.5 equiv.) and tributylphenylstannane (0.272 mmol, 1.0 equiv.). The balloon was removed, and the reaction proceeded at 80 °C for 6h. Afterwards, the reaction mixture was allowed to reach room temperature and the solvent was removed by rotary evaporator. The internal standard, 1,3,5-trimethoxybenzene (1 equiv.), was added to the reaction vial and the resulting mixture was treated with $CDCl_3$ (2 mL). The reaction vial was thoroughly shaken for 2 minutes, and then centrifuged (Figure S2-S9). This was followed by ^1H NMR analysis of an aliquot taken from the mixture (Figure S12).

Table S19. Robustness assessment for Stille coupling.

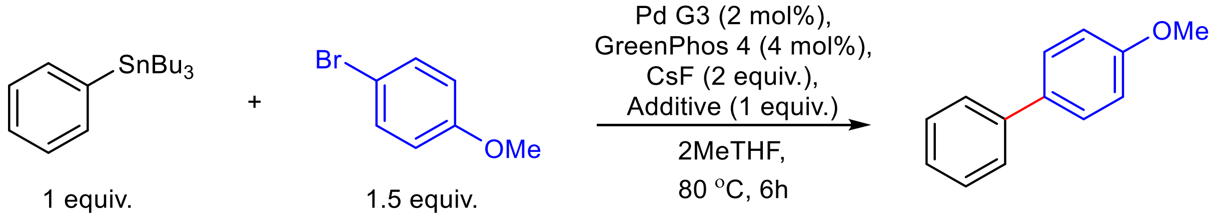

Entry	Additive (1 equiv.)	Yield (%)[a]	Additive remaining (%)[a]
1	none	100	/
2	Air	0	/
3	H_2O	56	/
4	Glycine anhydride	8	/
5	Dicyclohexylamine	21	70
6	1,4-Dicyanobenzene	99	46
7	1,4-Dicyanobenzene	100[b]	49[b]
8	1,4-Dinitrobenzene	5	73
9	Imidazole	0	63
10	2,6-Lutidine	100	30
11	Menthol	100	68
12	2,6-Dimethoxyphenol[c]	80	80
13	Palmitic acid[c]	24	100
14	Methyl 3,5-dinitrobenzoate	2	84
15	(1*S*)-(−)-Verbenone	96	100
16	Camphene	100	19
17	1-Nonyne	90	/
18	1-Nonyne	88[b]	/
19	Ethyl 3-chloropropionate	100	41
20	Cyclohexanecarboxaldehyde	100	20
21	Cyclohexyl methyl ketone	94	100
22	Dimethyl sulfone	67	76
23	Phenylboronic acid MIDA ester[d]	0	/

[a] Yield determined by ^{1}H NMR analysis in $CDCl_3$ using 1,3,5-trimethoxybenzene as an internal standard. [b] The reaction was performed with cataCXium® A instead of GreenPhos 4. [c] The remaining of phenol and carboxylic acid was determined following acidification of the reaction mixture. [d] Phenylboronic acid MIDA ester is only partially soluble in $CDCl_3$.

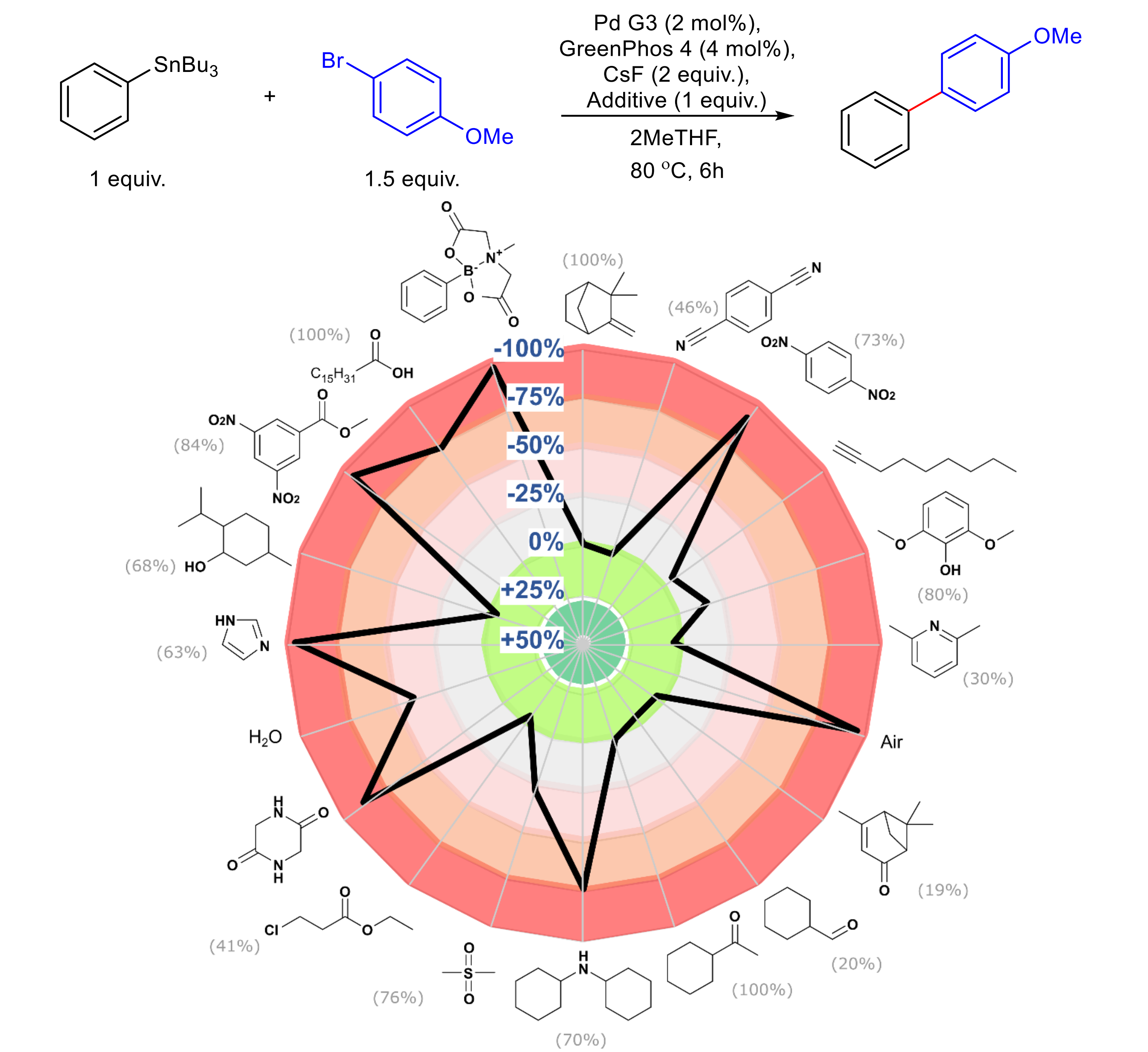

Figure S13. Robustness assessment radar diagram showing the effect of various additives on the yield of Stille coupling. Yields are determined by ^{1}H NMR analysis in $CDCl_3$ using 1,3,5-trimethoxybenzene as an internal standard. Numbers in the brackets indicate the percentages of recovered additives.

5.3. Buchwald-Hartwig amination

5.3.1. Optimization of the reaction conditions

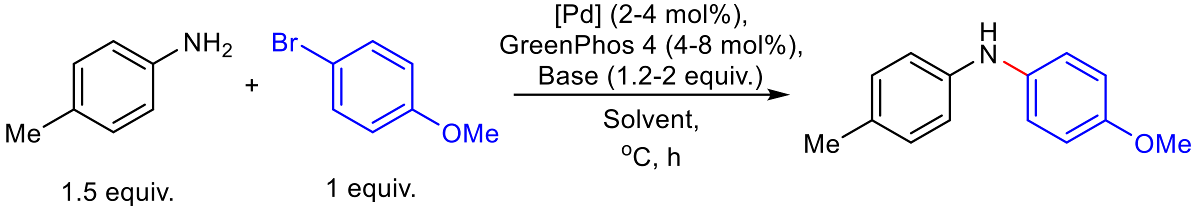

All reactions were conducted inside of 10 mL test tubes with standard ground joints. Inside of an argon-filled glovebox *p*-toluidine (1.5 equiv.), the corresponding palladium source (2-4 mol%), appropriate base (1.2-2 equiv.) and GreenPhos 4 (4-8 mol%) were weighed into an oven-dried reaction vial. The reaction vial was sealed with a rubber septum, removed from the glovebox, and equipped with an argon-filled balloon. This was followed by addition of the corresponding dry and degassed solvent (2 mL) and 4-bromoanisole (0.350 mmol, 1.0 equiv.). The balloon was removed, and the reaction proceeded at 20-80 °C for 2-24h. Afterwards, the reaction mixture was cooled down to room temperature, and the solvent was removed by rotary evaporator. The internal standard, 1,3,5-trimethoxybenzene (1 equiv.), was added to the reaction vial and the resulting mixture was treated with $CDCl_3$ (2 mL). The reaction vial was thoroughly shaken for 2 minutes, and then centrifuged (Figure S2-S9). This was followed by ^{1}H NMR analysis of an aliquot taken from the mixture (Figure S14).

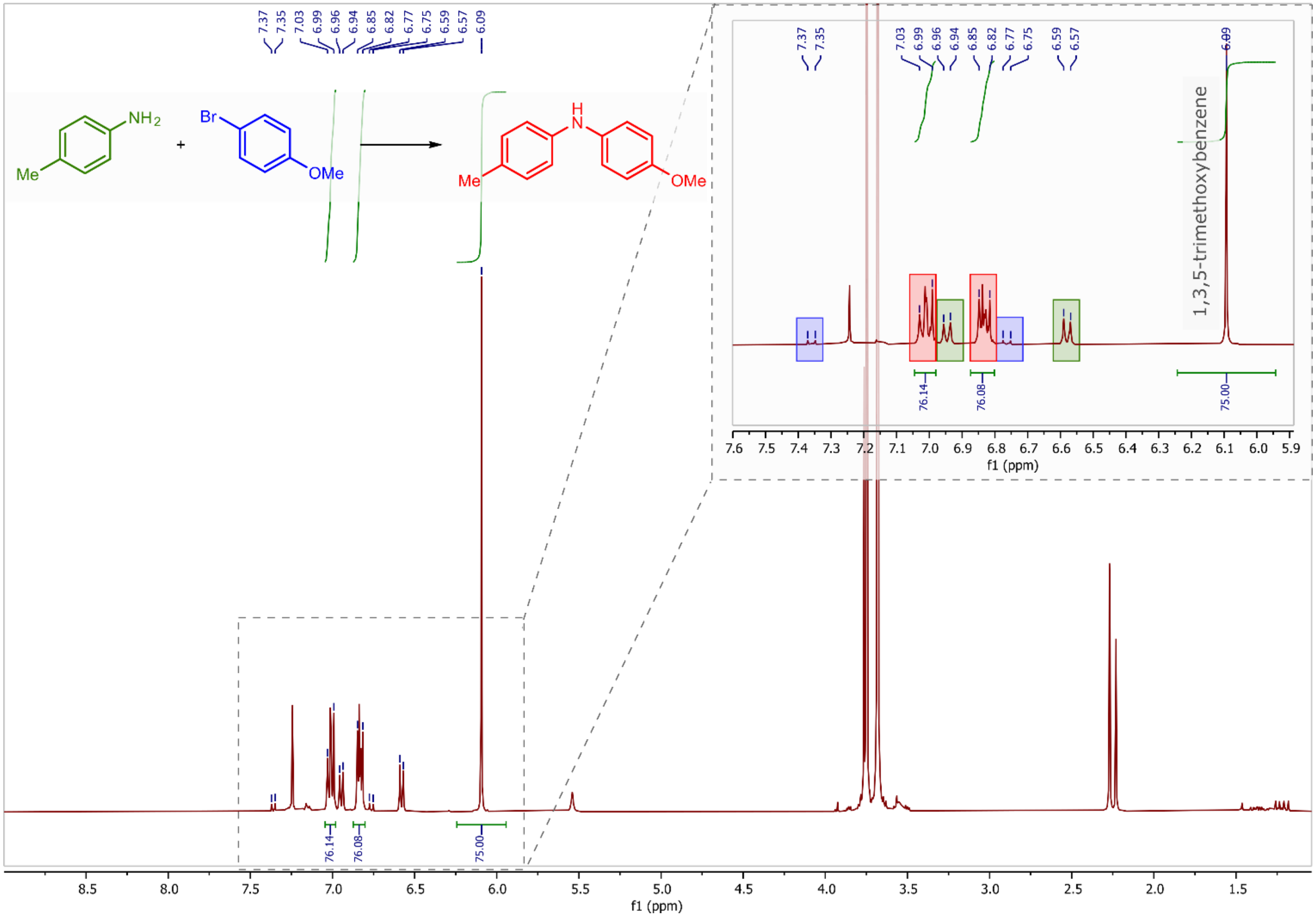

Figure S14. Selected example of a crude ^{1}H NMR for a Buchwald-Hartwig amination including 4-bromoanisole (blue) and *p*-toluidine (green). The resonance (d, δ_H: 6.82-6.85 ppm, integral range: 6.872-6.804 ppm) of the product (4-methoxy-*N*-*p*-tolylaniline) is indicated in red. The singlet resonance of the internal standard, 1,3,5-trimethoxybenzene, was referenced to δ_H: 6.0929 ppm and integrated to 75 protons (integral range: 6.243-5.943 ppm).

Table S20. Screening of bases for Buchwald-Hartwig amination.

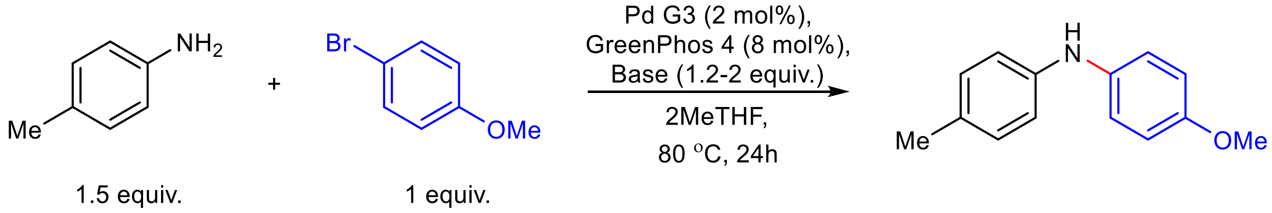

Entry	Base (equiv.)	Yield (%)[a]
1	CsF (2)	21
2	Cs_2CO_3 (2)	93
3	K_2CO_3 (2)	4
4	KO*t*Bu (2)	100
5	KOMe (2)	98
6	NaO*t*Bu (2)	96
7	NaOTMS (2)	73
8	LiO*t*Bu (2)	15
9	Cs_2CO_3 (1.2)	60

[a] Yield determined by ^{1}H NMR analysis in $CDCl_3$ using 1,3,5-trimethoxybenzene as an internal standard.

Table S21. Screening of temperature and duration for Buchwald-Hartwig amination.

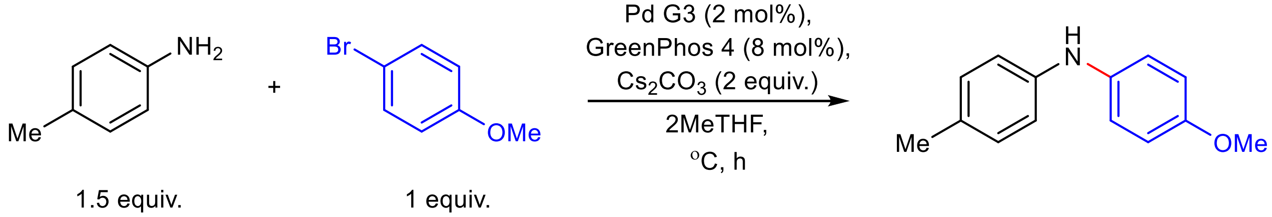

Entry	Temperature (°C)	Time (h)	Yield (%)[a]
1	80	24	93
2	80	6	46
3	80	2	13
4	20	24	0
5	20	6	0
6	40	24	0
7	40	6	0

[a] Yield determined by ^{1}H NMR analysis in $CDCl_3$ using 1,3,5-trimethoxybenzene as an internal standard.

Table S22. Screening of solvents for Buchwald-Hartwig amination.

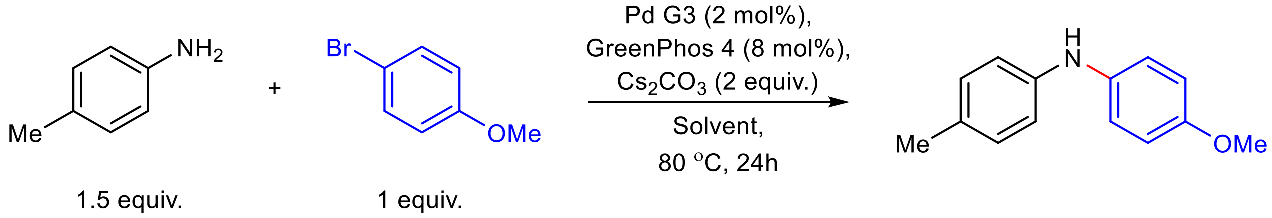

Entry	Solvent (2 mL)	Yield (%)[a]
1	2MeTHF	93
2	Acetal (1,1-diethoxyethane)	33
3	Dioxane	100
4	THF	89
5	DME	65
6	EtOH	0
7	Toluene	76
8	MeCN	10
9	DMF	49
10	Rapeseed oil (Askim)	22

[a] Yield determined by ^{1}H NMR analysis in $CDCl_3$ using 1,3,5-trimethoxybenzene as an internal standard.

Table S23. Screening of Pd precatalysts for Buchwald-Hartwig amination.

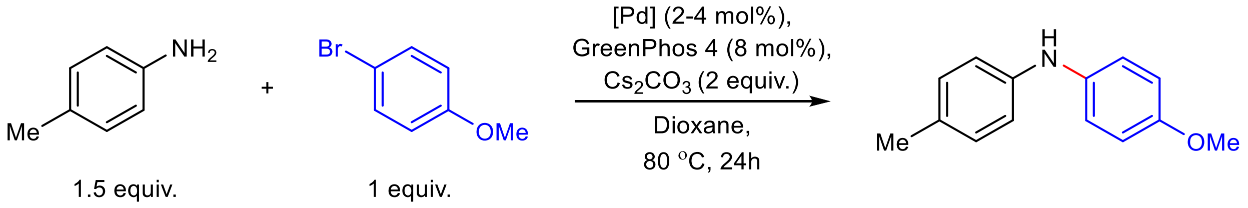

Entry	[Pd] (mol%)	Yield (%)[a]
1	$Pd(OAc)_2$ (4)	4
2	Pd_2dba_3 (2)	66
3	$PdCl_2(MeCN)_2$ (4)	13
4	$[PdCl(C_3H_5)]_2$ (2)	82
5	[(η³-1-*tert*-Butylindenyl)(μ-Cl)Pd]₂ (2)	76
6	$[(Cinnamyl)PdCl]_2$ (2)	83
7	Di-μ-chlorobis[2-[(dimethylamino)methyl]phenyl-C,N]dipalladium(II) (2)	79
8	Di-μ-mesylbis[2′-(amino-*N*)[1,1′-biphenyl]-2-yl-C]dipalladium(II) (Pd G3) (2)	100

[a] Yield determined by ^{1}H NMR analysis in $CDCl_3$ using 1,3,5-trimethoxybenzene as an internal standard.

5.3.2. Screening of phosphonium salts for Buchwald-Hartwig amination

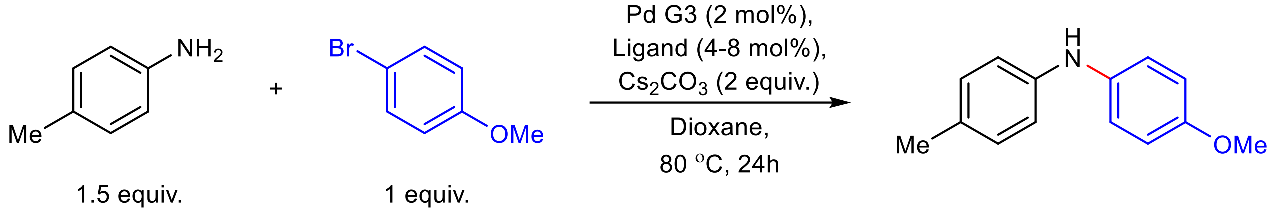

All reactions were conducted inside of 10 mL test tubes with standard ground joints. Inside of an argon-filled glovebox *p*-toluidine (1.5 equiv.), Pd G3 (2 mol%), Cs_2CO_3 (2 equiv.) and the appropriate ligand (4-8 mol%) were weighed into an oven-dried reaction vial. The reaction vial was sealed with a rubber septum, removed from the glovebox, and equipped with an argon-filled balloon. This was followed by addition of dry and degassed dioxane (2 mL) and 4-bromoanisole (0.350 mmol, 1.0 equiv.). The balloon was removed, and the reaction proceeded at 80 °C for 24h. Afterwards, the reaction mixture was cooled down to room temperature, and the solvent was removed by rotary evaporator. The internal standard, 1,3,5-trimethoxybenzene (1 equiv.), was added to the reaction vial and the resulting mixture was treated with $CDCl_3$ (2 mL). The reaction vial was thoroughly shaken for 2 minutes, and then centrifuged (Figure S2-S9). This was followed by ^1H NMR analysis of an aliquot taken from the mixture (Figure S14).

Table S24. Analysis of the effect of the ligand loading on the performance of the catalytic system.

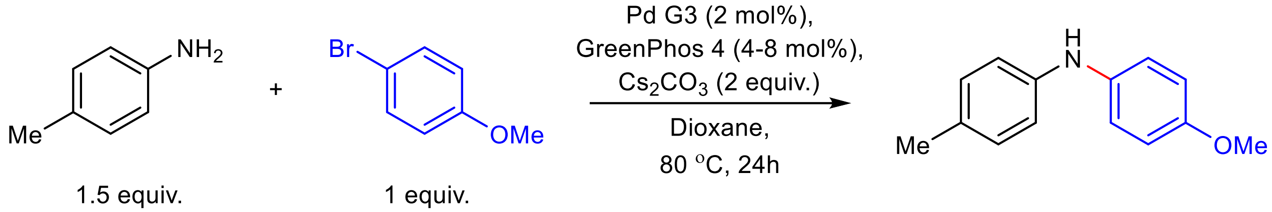

Entry	Ligand (mol%)	Yield (%)[a]
1	GreenPhos 4 (8)	100
2	GreenPhos 4 (4)	100

[a] Yield determined by ^1H NMR analysis in $CDCl_3$ using 1,3,5-trimethoxybenzene as an internal standard.

Table S25. Screening of phosphonium salts for Buchwald-Hartwig amination.

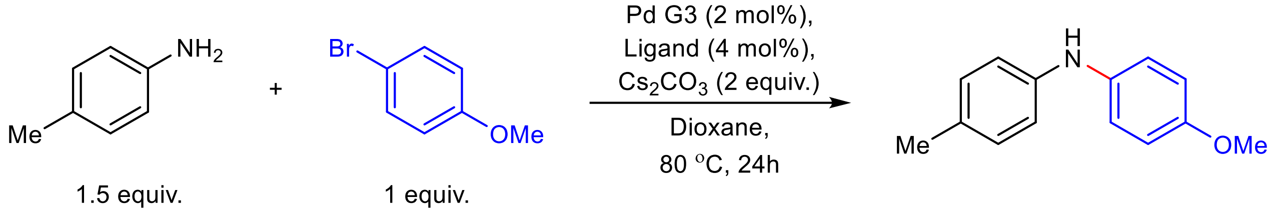

Entry	Ligand (4 mol%)	Yield (%)[a]
1	none	0
2	tBu$_2$PMeHBF$_4$	4
3	Ad$_2$PBuHI	94
4	tBu$_3$PHBF$_4$	97
5	GreenPhos 1	82
6	GreenPhos 2	82
7	GreenPhos 3	82
8	GreenPhos 4	100
9	GreenPhos 5	98
10	GreenPhos 6	70
11	GreenPhos 7	89
12	GreenPhos 8	90
13	GreenPhos 9	98
14	GreenPhos 10	94

[a] Yield determined by ^{1}H NMR analysis in $CDCl_3$ using 1,3,5-trimethoxybenzene as an internal standard.

5.3.3. Screening of phosphinites for Buchwald-Hartwig amination

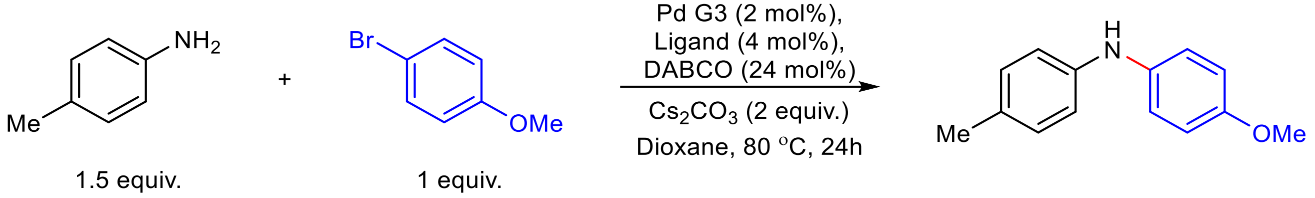

Inside of an argon-filled glovebox an oven dried 10 mL vial was sequentially charged with suitable ligand (4 mol%), DABCO (24 mol%) and anhydrous degassed dioxane (2 mL). The vial was sealed with a stopper equipped with a septum, removed from the glovebox and stirred at 85 °C for 24h.

The next day, inside of an argon-filled glovebox an oven dried 10 mL flask was charged with Pd G3 (2 mol%), which was followed by addition of deprotected ligand solution in dioxane. The vial containing the ligand solution was washed with anhydrous degassed dioxane (1 mL), which was added to the 10 mL flask. The resulting mixture was allowed to stir at 20 °C for 1 min, which lead to the formation of clear yellowish solution. Subsequently, the flask was sequentially charged with *p*-toluidine (1.5 equiv.), Cs_2CO_3 (2 equiv.) and degassed 4-bromoanisole (1 equiv., 0.374 mmol). The flask was sealed with a rubber septum, removed from the glovebox and the resulting mixture was stirred at 80 °C for 24h. Afterwards, the reaction mixture was cooled down to room temperature, which was followed by the removal of volatiles by rotary evaporator and addition of 1,3,5-trimethoxybenzene (1 equiv., internal standard). The resulting mixture was treated with $CDCl_3$ (2 mL), thoroughly shaken for 2 minutes, and then centrifuged (Figure S2-S9). This was followed by ^{1}H NMR analysis of an aliquot taken from the mixture (Figure S14).

Table S26. Screening of phosphinites for Buchwald-Hartwig amination.

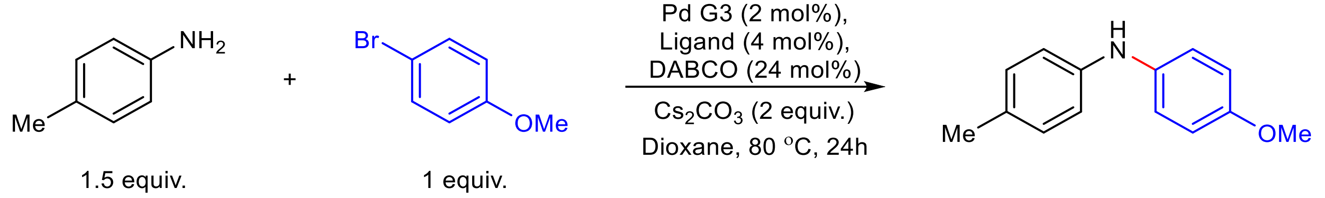

Entry	Ligand (4 mol%)	Yield (%)[a]
1	GreenPhos 11	96
2	GreenPhos 12	71
3	GreenPhos 13	93
4	GreenPhos 14	4
5	GreenPhos 15	8
6	GreenPhos 16	5
7	GreenPhos 17	15
8	GreenPhos 18	9
9	GreenPhos 19	5
10	GreenPhos 20	3

[a] Yield determined by ^{1}H NMR analysis in $CDCl_3$ using 1,3,5-trimethoxybenzene as an internal standard.

5.3.4. Optimization of Buchwald-Hartwig amination for 4-chlorotoluene

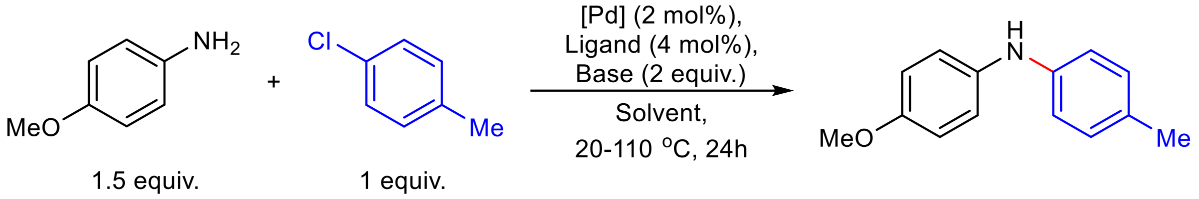

All reactions were conducted inside of 10 mL pressure tubes equipped with corresponding septum, PTFE stabilizer and a screw cap. Inside of an argon-filled glovebox 4-methoxyaniline (1.5 equiv.), the appropriate palladium source (2 mol%), the corresponding base (2 equiv.) and ligand (4 mol%) were weighed into an oven-dried pressure tube. The pressure tube was sealed with a screw cap, removed from the glovebox, and equipped with an argon-filled balloon. This was followed by addition of the corresponding dry and degassed solvent (2 mL) and 4-chlorotoluene (0.350 mmol, 1.0 equiv.). The balloon was removed, and the reaction proceeded at 20-110 °C for 24h. Afterwards, the reaction mixture was cooled down to room temperature, and the solvent was removed by rotary evaporator. The internal standard, 1,3,5-trimethoxybenzene (1 equiv.), was added to the reaction vial and the resulting mixture was treated with $CDCl_3$ (2 mL). The reaction vial was thoroughly shaken for 2 minutes, and then centrifuged (Figure S2-S9). This was followed by ^{1}H NMR analysis of an aliquot taken from the mixture (Figure S14).

Table S27. Optimization of Buchwald-Hartwig amination for 4-chlorotoluene.

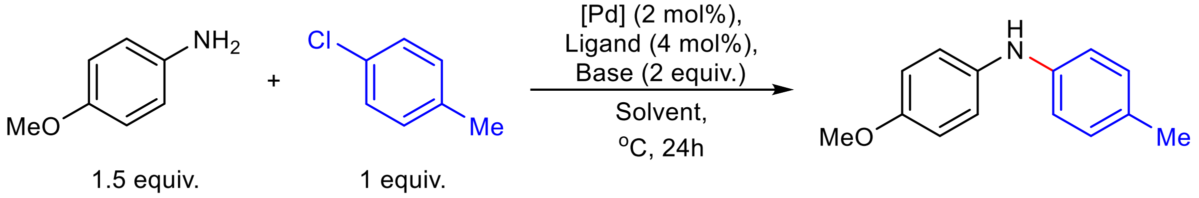

Entry	[Pd] (2 mol%)	Ligand (4 mol%)	Base (2 equiv.)	Solvent	°C	Yield (%)[a]
1	Pd G3	GreenPhos 4	Cs_2CO_3	2MeTHF	110	91
2	Pd G3	GreenPhos 4	KO*t*Bu	2MeTHF	110	100
3	Pd G3	GreenPhos 4	KO*t*Bu	2MeTHF	80	100
4	Pd G3	GreenPhos 4	KO*t*Bu	2MeTHF	40	88
5	Pd G3	GreenPhos 4	KO*t*Bu	2MeTHF	20	82
6	Pd G3	GreenPhos 4	Cs_2CO_3	Dioxane	110	100
7	Pd G3	GreenPhos 4	Cs_2CO_3	THF	110	84
8	[(Cinnamyl)PdCl]$_2$	GreenPhos 4	Cs_2CO_3	Dioxane	110	95
9	Pd G3	GreenPhos 7	Cs_2CO_3	Dioxane	110	94
10	Pd G3	GreenPhos 9	Cs_2CO_3	Dioxane	110	100
11	Pd G3	GreenPhos 5	Cs_2CO_3	Dioxane	110	55
12	[(Cinnamyl)PdCl]$_2$	GreenPhos 4	Cs_2CO_3	2MeTHF	110	40
13	[(Cinnamyl)PdCl]$_2$	GreenPhos 5	Cs_2CO_3	Dioxane	110	100
14	[(Cinnamyl)PdCl]$_2$	GreenPhos 5	Cs_2CO_3	Dioxane	20	0
15	Pd G3	GreenPhos 4	Cs_2CO_3	Dioxane	80	67
16	Pd G3	GreenPhos 4	Cs_2CO_3	Dioxane	20	9

[a] Yield determined by ^1H NMR analysis in $CDCl_3$ using 1,3,5-trimethoxybenzene as an internal standard.

5.3.5. Estimation of the functional group tolerance of Buchwald-Hartwig amination

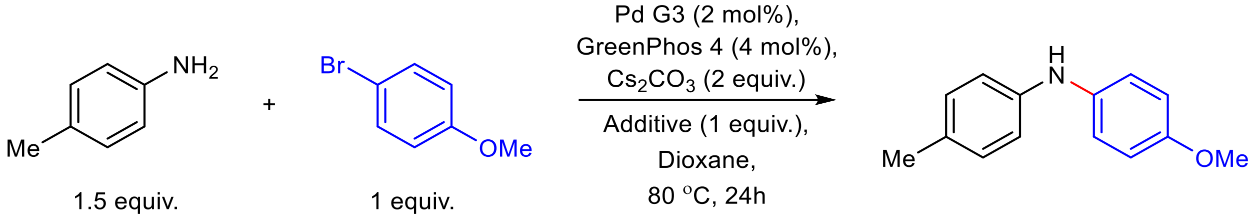

All reactions were conducted inside of 10 mL test tubes with standard ground joints. Inside of an argon-filled glovebox *p*-toluidine (1.5 equiv.), Pd G3 (2 mol%), Cs_2CO_3 (2 equiv.) and GreenPhos 4 (4 mol%) were weighed into an oven-dried reaction vial. In the case of solid additives (1 equiv.), these were weighed into the reaction vials prior to the introduction of the reaction vails to the glovebox. Liquid additives (1 equiv.) were added to the reaction vials after the addition of solvent and 4-bromoanisole. The reaction vial was sealed with a rubber septum, removed from the glovebox, and equipped with an argon-filled balloon. This was followed by addition of dry and degassed dioxane (2 mL) and 4-bromoanisole (0.350 mmol, 1.0 equiv.). The balloon was removed, and the reaction proceeded at 80 °C for 24h. Afterwards, the reaction was allowed to reach room temperature and the solvent was removed by rotary evaporator. The internal standard, 1,3,5-trimethoxybenzene (1 equiv.), was added to the reaction vial and the resulting mixture was treated with $CDCl_3$ (2 mL). The reaction vial was thoroughly shaken for 2 minutes, and then centrifuged (Figure S2-S9). This was followed by ^{1}H NMR analysis of an aliquot taken from the mixture (Figure S14).

Table S28. Robustness assessment for Buchwald-Hartwig amination.

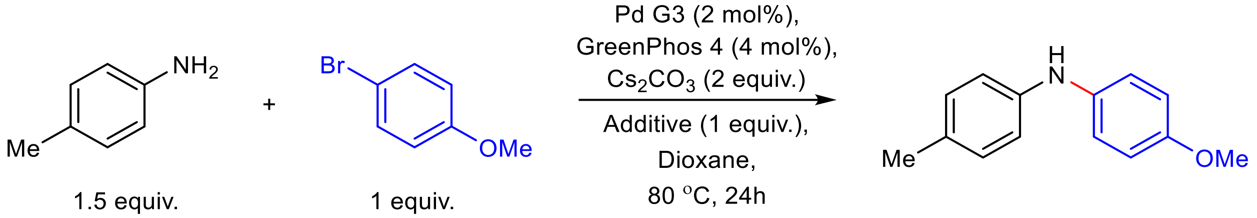

Entry	Additive (1 equiv.)	Yield (%)[a]	Additive remaining (%)[a]
1	none	100	/
2	Air	6	/
3	H_2O	89	/
4	Glycine anhydride	3	0
5	Dicyclohexylamine	98	96
6	1,4-Dicyanobenzene	0	50
7	1,4-Dicyanobenzene	0[b]	47[b]
8	1,4-Dinitrobenzene	32	71
9	Imidazole	0	30
10	2,6-Lutidine	91	40
11	Menthol	26	0
12	2,6-Dimethoxyphenol[c]	52	100
13	Palmitic acid[c]	20	100
14	Methyl 3,5-dinitrobenzoate	18	26
15	1-Nonyne	59	100
16	1-Nonyne	60[b]	100[b]
17	Ethyl 3-chloropropionate	36	0
18	Cyclohexanecarboxaldehyde	29	0
19	Cyclohexyl methyl ketone	71	40
20	Dimethyl sulfone	98	81
21	Phenylboronic acid MIDA ester[d]	2	/
22	(1*S*)-(−)-Verbenone	59	45
23	Camphene	91	/

[a] Yield determined by ^1H NMR analysis in $CDCl_3$ using 1,3,5-trimethoxybenzene as an internal standard. [b] The reaction was performed with cataCXium® A instead of GreenPhos 4. [c] The remaining of phenol and carboxylic acid were determined following acidification of the reaction mixture. [d] Phenylboronic acid MIDA ester is only partially soluble in $CDCl_3$.

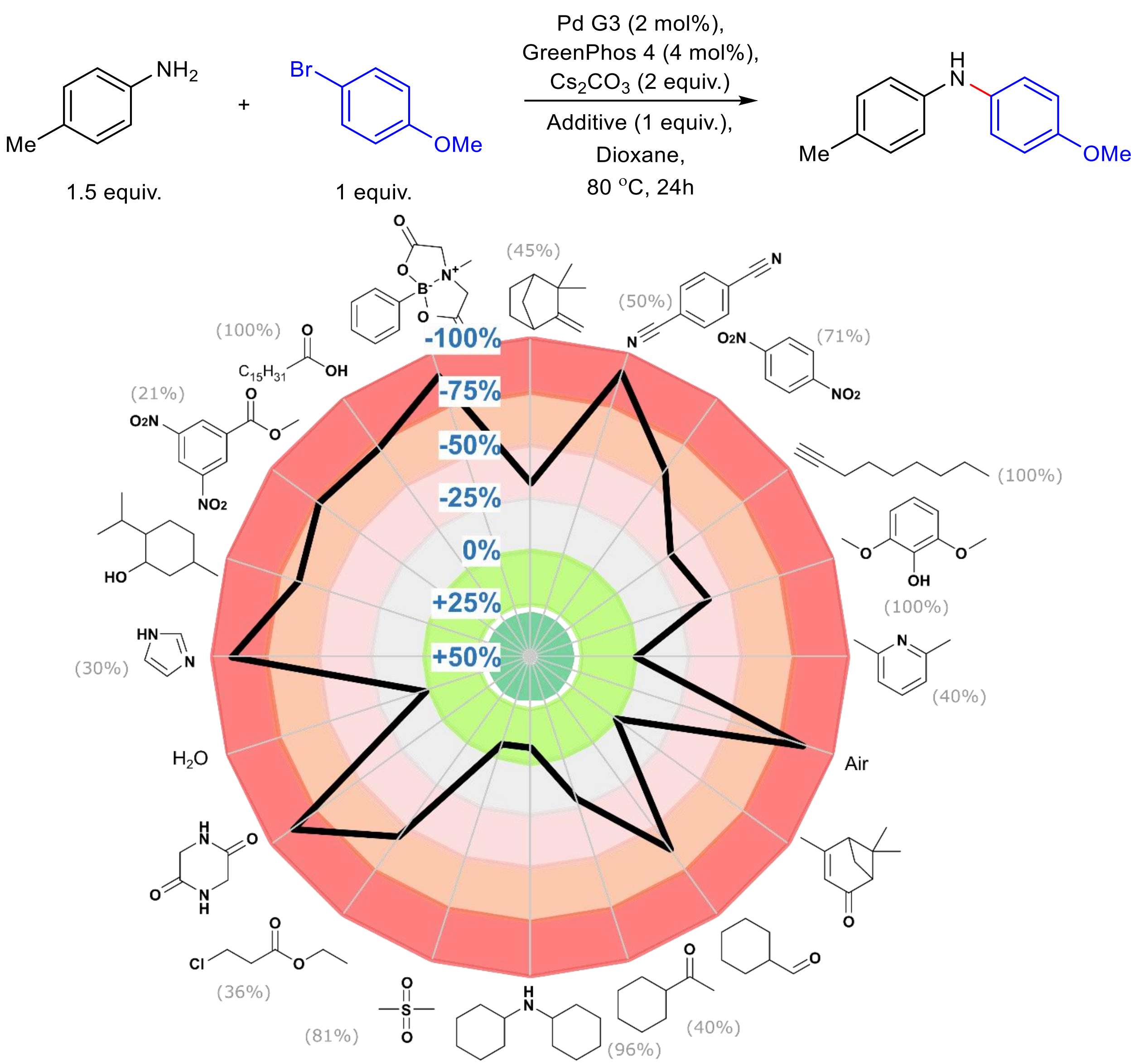

Figure S15. Robustness assessment radar diagram showing the effect of various additives on the yield of Buchwald-Hartwig amination. Yields are determined by ^{1}H NMR analysis in $CDCl_3$ using 1,3,5-trimethoxybenzene as an internal standard. Numbers in the brackets indicate the percentages of recovered additives.

6. Stille coupling with low catalyst loading

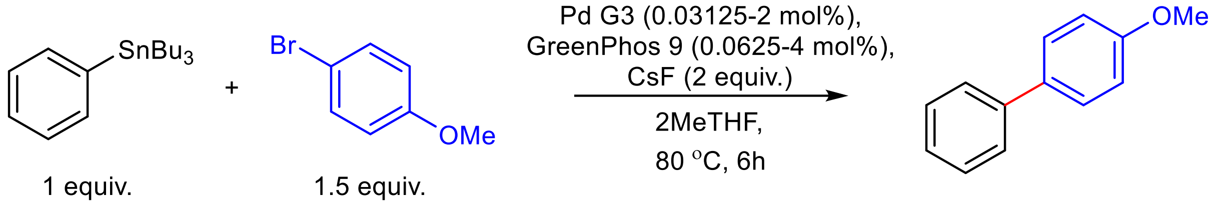

Inside of an argon-filled glovebox an oven dried 10 mL flask was sequentially charged with degassed tributylphenylstannane (1 equiv., 0.354 mmol), CsF (2 equiv.), degassed 4-bromoanisole (1.5 equiv.) and the corresponding solution of Pd G3 (0.03125-2 mol%) and GreenPhos 9 (0.0625-8 mol%) in anhydrous degassed 2MeTHF (2 mL, for details see below). The flask was sealed with a rubber septum, removed from the glovebox and the resulting mixture was stirred at 80 °C for 6h. Afterwards, the reaction mixture was cooled down to room temperature, which was followed by the removal of volatiles using rotary evaporator and addition of 1,3,5-trimethoxybenzene (1 equiv., internal standard). The resulting mixture was treated with $CDCl_3$ (2 mL), thoroughly shaken for 2 minutes, and then centrifuged (Figure S2-S9). This was followed by ^{1}H NMR analysis of an aliquot taken from the mixture (Figure S12).

To get a 4 mol% catalyst loading Pd G3 (2 mol%) and GreenPhos 9 (4 mol%) were dissolved in 2 mL anhydrous degassed 2MeTHF and transferred into the reaction flask.
To get a 2 mol% catalyst loading Pd G3 (1 mol%) and GreenPhos 9 (2 mol%) were dissolved in 2 mL anhydrous degassed 2MeTHF and transferred into the reaction flask.
To get a 1 mol% catalyst loading Pd G3 (1 mol%) and GreenPhos 9 (2 mol%) were dissolved in 4 mL anhydrous degassed 2MeTHF. 2 mL out of the prepared solution was transferred into the reaction flask.
To get a 0.5 mol% catalyst loading Pd G3 (1 mol%) and GreenPhos 9 (2 mol%) were dissolved in 8 mL anhydrous degassed 2MeTHF. 2 mL out of the prepared solution was transferred into the reaction flask.
To get a 0.25 mol% catalyst loading Pd G3 (1 mol%) and GreenPhos 9 (2 mol%) were dissolved in 16 mL anhydrous degassed 2MeTHF. 2 mL out of the prepared solution was transferred into the reaction flask.
To get a 0.125 mol% catalyst loading Pd G3 (1 mol%) and GreenPhos 9 (2 mol%) were dissolved in 32 mL anhydrous degassed 2MeTHF. 2 mL out of the prepared solution was transferred into the reaction flask.
To get a 0.0625 mol% catalyst loading Pd G3 (1 mol%) and GreenPhos 9 (2 mol%) were dissolved in 64 mL anhydrous degassed 2MeTHF. 2 mL out of the prepared solution was transferred into the reaction flask.

Table S29. The effect of low catalyst loading on the efficiency of Stille coupling.

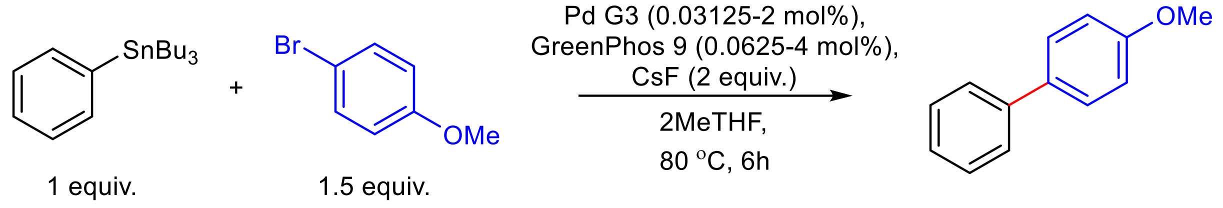

Entry	Pd-source (mol%)	Ligand (mol%)	Yield (%)[a]
1	Pd G3 (2)	-	6
2	Pd G3 (2)	GreenPhos 9 (8)	100
3	Pd G3 (2)	GreenPhos 9 (4)	100
4	Pd G3 (1)	GreenPhos 9 (2)	100
5	Pd G3 (0.5)	GreenPhos 9 (1)	100
6	Pd G3 (0.25)	GreenPhos 9 (0.5)	100
7	Pd G3 (0.125)	GreenPhos 9 (0.25)	87
8	Pd G3 (0.0625)	GreenPhos 9 (0.125)	85
9	Pd G3 (0.03125)	GreenPhos 9 (0.0625)	56

[a] Yield determined by ^{1}H NMR analysis in $CDCl_3$ using 1,3,5-trimethoxybenzene as an internal standard.

7. Selected examples of isolated products

7.1.1. Isolated products for Suzuki-Miyaura coupling

General procedure for the preparative Suzuki-Miyaura coupling (Figure S16)

All reactions were conducted in oven-dried 50 mL pressure tubes equipped with corresponding septum, PTFE stabilizer and screw cap. Inside of an argon-filled glovebox the corresponding boronic acid (0.736 mmol, 1 equiv.), Pd G3 (2 mol%), KOH (2 equiv.) and GreenPhos 4 (4 mol%) were weighed into the appropriate reaction flask. The pressure tube was sealed with appropriate screw cap, removed from the glovebox and equipped with an argon-filled balloon. Next, the flask was charged with anhydrous and degassed DME (4 mL) and appropriate aryl halide (1.5 equiv.). The balloon was removed, and the reaction proceeded at 20-110 °C for 24h. Afterwards, the reaction mixture was cooled down to room temperature, and the solvent was removed by rotary evaporator. The products were isolated by flash chromatography using mixtures of heptane and EtOAc as eluent.

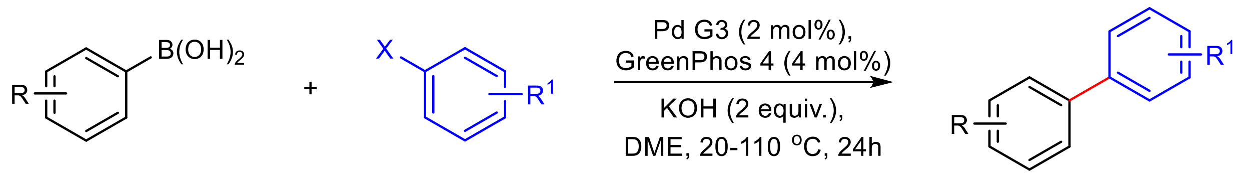

1 1 equiv. **2** 1.5 equiv. **3a-d**

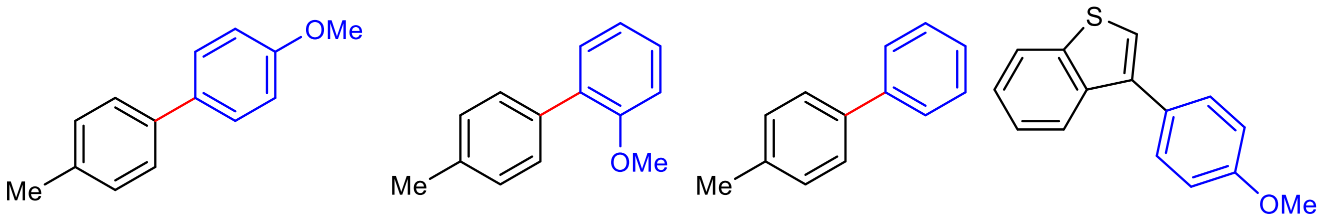

**3a**	**3b**	**3c**	**3d**
**X = Br, 89%[a]**	**X = Br, 99%[b]**	**X = Cl, 91%[b]**	**X = Br, 80%[b]**

Figure S16. The scope of isolated products for Suzuki-Miyaura coupling. [a] The reaction was performed at 20 °C. [b] The reaction was performed at 110 °C.

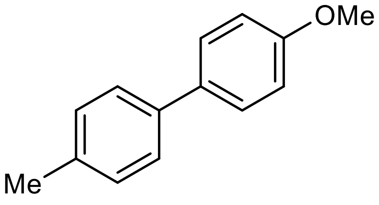

4-Methoxy-4'-methyl-1,1'-biphenyl, 3a.[13] Starting from *p*-tolylboronic acid (0.10 g, 0.736 mmol) the product was obtained as white crystalline powder (0.12 g, 89%, X = Br). **^{1}H NMR (400 MHz, $CDCl_3$):** δ = 2.39 (s, 3H, Me), 3.85 (s, 3H, OMe), 6.97 (d, *J* = 8.8 Hz, 2H, Ar), 7.23 (d, *J* = 7.8 Hz, 2H, Ar), 7.46 (d, *J* = 8.2 Hz; 2H, Ar), 7.52 (d, *J* = 8.9 Hz, 2H, Ar). **^{13}C NMR (101 MHz, $CDCl_3$):** δ = 21.2, 55.5, 114.3, 126.7, 128.1, 129.6, 133.9, 136.5, 138.1, 159.1.

[13] H. Zhao, Y. Wang, J. Sha, S. Sheng and M. Cai, *Tetrahedron*, 2008, **64**, 7517-7523.

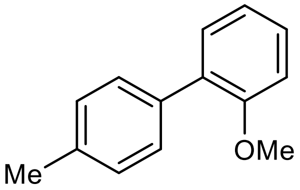

2-Methoxy-4'-methyl-1,1'-biphenyl, 3b.[14] Starting from *p*-tolylboronic acid (0.11 g, 0.772 mmol) the product was obtained as a yellow crystalline solid (0.15 g, 99%, X = Br). **^{1}H NMR (400 MHz, $CDCl_3$):** δ = 2.46 (s, 3H, Me), 3.86 (s, 3H, OMe), 7.03-7.10 (m, 2H, Ar), 7.29 (d, J = 8.4 Hz, 2H, Ar), 7.35-7.39 (m, 2H, Ar), 7.50-7.51(m, 2H, Ar). **^{13}C NMR (101 MHz, $CDCl_3$):** δ = 21.3, 55.6, 111.3, 120.9, 128.5, 128.9, 129.5, 130.8, 130.9, 135.7, 136.7, 156.6.

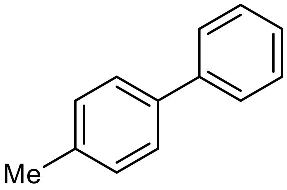

4-Methyl-1,1'biphenyl, 3c.[15] Starting from *p*-tolylboronic acid (0.10 g, 0.736 mmol) the product was obtained as a colorless oil (0.11 g, 91%, X = Cl). **^{1}H NMR (400 MHz, $CDCl_3$):** δ = 2.44 (s, 3H, Me), 7.29 (d, *J* = 7.8 Hz, 2H, Ar), 7.34-7.39 (m, 1H, Ar), 7.45-7.49 (m, 2H, Ar), 7.53-7.55 (m, 2H, Ar), 7.61-7.64 (m, 2H, Ar). **^{13}C NMR (101 MHz, $CDCl_3$):** δ = 21.2, 127.1, 127.2, 128.9, 129.6, 137.1, 138.5, 141.3.

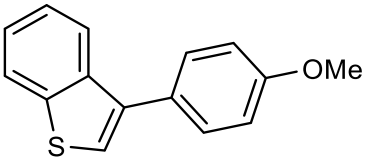

3-(4-Methoxyphenyl)benzo[b]thiophene, 3d.[16] Starting from benzo[*b*]thien-3-ylboronic acid (0.10 g, 0.562 mmol) the product was obtained as a colorless oil (0.11 g, 80%, X = Br). **^{1}H NMR (400 MHz, $CDCl_3$):** δ = 3.89 (s, 3H, OMe), 7.03 (d, *J* = 8.8 Hz, 2H, Ar), 7.34 (s, 1H, Ar), 7.34-7.42 (m, 2H, Ar), 7.53 (d, *J* = 8.8 Hz, 2H, Ar), 7.89-7.94 (m, 2H, Ar). **^{13}C NMR (101 MHz, $CDCl_3$):** δ = 55.4, 114.2, 122.6, 122.9, 124.3, 124.3, 128.6, 129.8, 137.7, 138.1, 140.7, 159.2.

[14] M. S. C. Rao and G. S. K. Rao, *Synthesis*, 1987, 231-233.
[15] D. W. Old, J. P. Wolfe and S. L. Buchwald, *J. Am. Chem. Soc.*, 1998, **120**, 9722-9723.
[16] K. Funaki, T. Sato and S. Oi, *Org. Lett.*, 2012, **14**, 6186-6189.

7.1.2. Isolated products for Stille coupling

General procedure for the preparative Stille coupling (Figure S17)

All reactions were conducted in oven-dried 50 mL pressure tubes equipped with suitable septum, PTFE stabilizer and screw cap. Inside of an argon-filled glovebox Pd G3 (2 mol%), CsF (2 equiv.) and GreenPhos 4 (4 mol%) were weighed into the appropriate reaction flask. The pressure tube was sealed with suitable screw cap, removed from the glovebox and equipped with an argon-filled balloon. This was followed by addition of anhydrous, and degassed DME (7 mL) appropriate aryl halide or aryl triflate (1.5 equiv.) and organotin reagent (0.953 mmol, 1.0 equiv.). The balloon was removed, and the reaction proceeded at 80-110 °C for 6-24h. Afterwards, the reaction mixture was cooled down to room temperature, and the solvent was removed by rotary evaporator. The products were isolated by flash chromatography using mixtures of heptane and EtOAc as eluent.

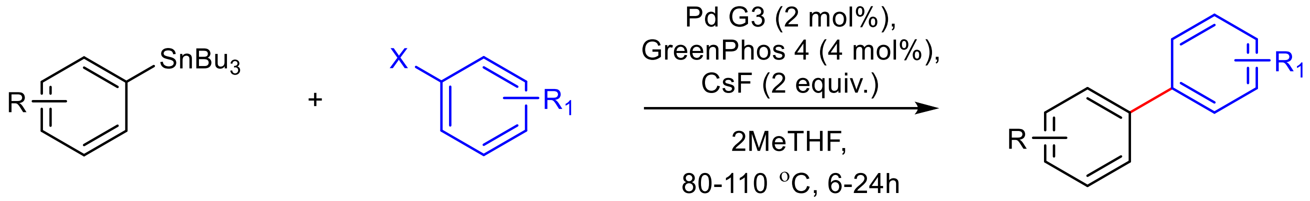

4 1 equiv. **2** 1.5 equiv. **5a-d**

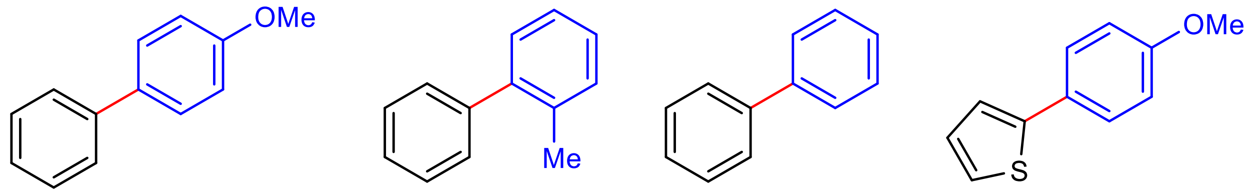

5a X = Br, 99%[a] **5b** X = Br, 85%[b] **5c** X = OTf, 95%[a] **5d** X = Br, 88%[b]

Figure S17. The scope of isolated products for Stille coupling. [a] The reaction was performed at 80 °C for 6h. [b] The reaction was performed at 110 °C for 24h.

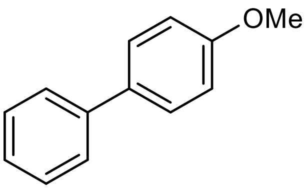

4-Methoxy-1,1'-biphenyl, 5a.[17] Starting from tributylphenylstannane (0.35 g, 0.953 mmol) the product was obtained as a white crystalline solid (0.17 g, 99%, X = Br). **^{1}H NMR (400 MHz, CDCl$_3$):** δ = 3.88 (s, 3H, OMe), 7.02 (d, $^3J_{HH}$ = 8.8 Hz, 2H, Ar), 7.32-7.36 (m, 1H, Ar), 7.46 (t, $^3J_{HH}$ = 7.7 Hz, 2H, Ar), 7.55-7.61 (m, 4H, Ar). **^{13}C NMR (101 MHz, CDCl$_3$):** δ = 55.4, 114.3, 126.8, 126.9, 128.3, 128.8, 133.9, 140.9, 159.3.

[17] I. D. Inaloo, S. Majnooni, H. Eslahi and M. Esmaeilpour, *ACS Omega*, 2020, **5**, 7406-7417.

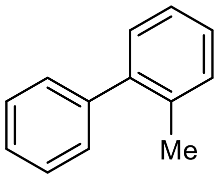

2-Methyl-1-1'-biphenyl, 5b.[18] Starting from tributylphenylstannane (0.35 g, 0.953 mmol) the product was obtained as a colorless oil (0.14 g, 85%, X = Br). **^{1}H NMR (400 MHz, $CDCl_3$):** δ = 2.28 (s, 3H, Me), 7.23-7.29 (m, 4H, Ar), 7.31-7.36 (m, 3H, Ar), 7.39-7.44 (m, 2H, Ar). **^{13}C NMR (101 MHz, $CDCl_3$):** δ = 20.6, 125.9, 126.9, 127.4, 128.4, 129.3, 129.9, 130.4, 135.5, 142.1, 142.2.

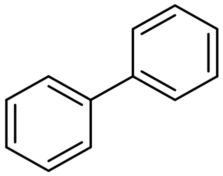

1,1'-Biphenyl, 5c.[19] Starting from tributylphenylstannane (0.35 g, 0.953 mmol) the product was obtained as a colorless oil (0.14 g, 95%, X = OTf). **^{1}H NMR (400 MHz, $CDCl_3$):** δ = 7.42-7.46 (m, 2H, Ar), 7.51-7.56 (m, 4H, Ar), 7.68-7.71 (m, 4H, Ar). **^{13}C NMR (101 MHz, $CDCl_3$):** δ = 127.3, 127.4, 128.9, 141.4.

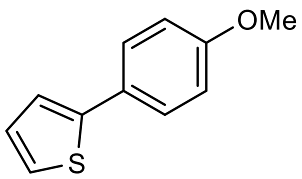

2-(4-Methoxyphenyl)thiophene, 5d.[20] Starting from tributyl(thiophen-2-yl)stannane (0.35 g, 0.938 mmol) the product was obtained as a white crystalline solid (0.16 g, 88%, X = Br). **^{1}H NMR (400 MHz, $CDCl_3$):** δ = 3.85 (s, 3H, OMe), 6.94 (d, $^3J_{HH}$ = 8.8 Hz, 2H, Ar), 7.06-7.08 (m, 1H, Ar), 7.21-7.24 (m, 2H, Ar), 7.56 (d, $^3J_{HH}$ = 8.8 Hz, 2H, Ar). **^{13}C NMR (101 MHz, $CDCl_3$):** δ = 55.1, 114.1, 121.9, 123.6, 127.0, 127.1, 127.7, 144.1, 159.0.

[18] F. Vallee, J. J. Mousseau and A. B. Charette, *J. Am. Chem. Soc.*, 2010, **132**, 1514-1516.
[19] Q. Lin, L. Xue, J. Sun, Y. Wang and H. Cheng, *J. Am. Soc. Mass Spectrom.*, 2022, **33**, 1921-1935.
[20] Y. Sudo, E. Yamaguchi and A. Itoh, *Org. Lett.*, 2017, **19**, 1610-1613.

7.1.3. Isolated products for Buchwald-Hartwig amination

General procedure for the preparative Buchwald-Hartwig amination (Figure S18)

All reactions were conducted in oven-dried 50 mL pressure tubes equipped with corresponding septum, PTFE stabilizer and screw cap. Inside of an argon-filled glovebox the corresponding amine (1.5 equiv.), Pd G3 (2 mol%), Cs_2CO_3 (2 equiv.) and GreenPhos 4 (4 mol%) were weighed into the pressure tube. The pressure tube was sealed with appropriate screw cap, removed from the glovebox and equipped with an argon-filled balloon. This was followed by addition of anhydrous and degassed dioxane (4 mL) and appropriate aryl halide (0.700 mmol, 1 equiv.). The balloon was removed, and the reaction proceeded at 80-110 °C for 24h. Afterwards, the reaction mixture was cooled down to room temperature, and the solvent was removed by rotary evaporator. The products were isolated by flash chromatography using mixtures of heptane and EtOAc as eluent.

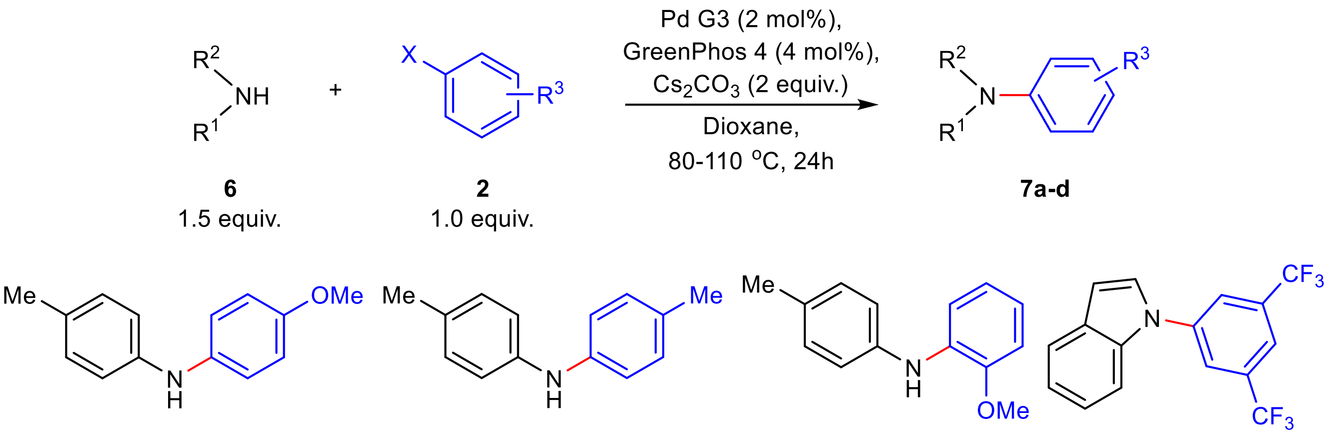

Figure S18. The scope of isolated products for Buchwald-Hartwig amination. [a] The reaction was performed at 80 °C. [b] The reaction was performed at 110 °C.

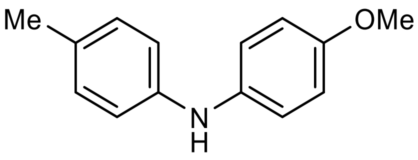

4-Methoxy-N-p-tolylaniline, 7a.[21] 4-Methoxy-*N*-*p*-tolylaniline was obtained from 4-bromoanisole (0.13 g, 0.700 mmol) as a light brown crystalline solid (0.13 g, 87%, X = Br). **^{1}H NMR (400 MHz, $CDCl_3$):** δ = 2.30 (s, 3H, Me), 3.81 (s, 3H, OMe), 5.40 (br s, 1H, NH), 6.85-6.88 (m, 4H, Ar), 7.03-7.07 (m, 4H, Ar). **^{13}C NMR (101 MHz, $CDCl_3$):** δ = 20.7, 55.7, 114.8, 116.7, 121.2, 129.4, 129.9, 136.8, 142.5, 154.9.

[21] Q. Shen and J. F. Hartwig, *Org. Lett.*, 2008, **10**, 4109-4112.

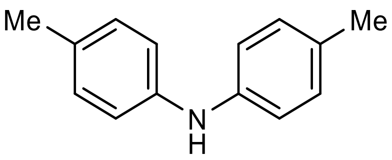

Di-p-tolylamine, 7b.[22] Di-*p*-tolylamine was obtained from 4-chlorotoluene (0.089 g, 0.700 mmol) as an orange solid (0.12 g, 85%, X = Cl). **^{1}H NMR (400 MHz, CDCl$_3$):** δ = 2.35 (s, 6H, 2xMe), 5.53 (br s, 1H, NH), 6.98-7.00 (m, 4H, Ar), 7.10-7.12 (m, 4H, Ar). **^{13}C NMR (101 MHz, CDCl$_3$):** δ = 20.7, 118.0, 129.9, 130.2, 141.3.

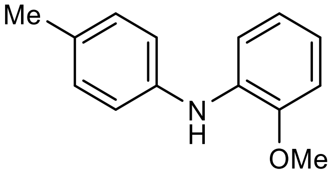

2-Methoxy-N-p-tolylaniline, 7c.[23] 2-Methoxy-*N*-*p*-tolylaniline was obtained from 2-bromoanisole (0.13 g, 0.700 mmol) as a yellow oil (0.11 g, 70%, X = Br). **^{1}H NMR (400 MHz, CDCl$_3$):** δ = 2.19 (s, 3H, Me), 3.74 (s, 3H, OMe), 5.96 (br s, 1H, NH), 6.67-6.76 (m, 3H, Ar), 6.93-6.99 (m, 4H, Ar), 7.10-7.12 (m, 1H, Ar). **^{13}C NMR (101 MHz, CDCl$_3$):** δ = 20.8, 55.6, 110.5, 113.8, 119.3, 119.7, 120.9, 129.9, 131.0, 133.9, 140.0, 147.9.

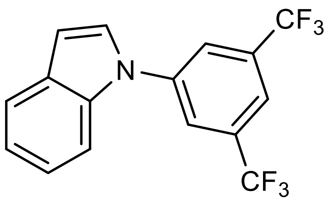

1-(3,5-Bis(trifluoromethyl)phenyl)-1H-indole, 7d.[24] 1-(3,5-Bis(trifluoromethyl)phenyl)-1*H*-indole was obtained from 1,3-bis(trifluoromethyl)-5-bromobenzene (0.205 g, 0.700 mmol) as a colorless oil (0.202 g, 88%, X = Br). **^{1}H NMR (400 MHz, CDCl$_3$):** δ = 6.80 (d, *J* = 3.4 Hz, 1H, indole), 7.25-7.29 (m, 1H, indole), 7.31-7.35 (m, 1H, indole), 7.38 (d, *J* = 3.4 Hz, 1H, indole), 7.57 (d, *J* = 8.3 Hz, 1H, indole), 7.74 (d, *J* = 7.8 Hz, 1H, indole), 7.90 (s, 1H, Ar), 8.02 (s, 2H, Ar). **^{13}C NMR (101 MHz, CDCl$_3$):** δ = 106.1, 109.9, 119.8 (p, *J* = 3.8 Hz), 121.7, 121.9, 123.1 (q, *J* = 271 Hz), 123.7, 124.0 (q, *J* = 3.7 Hz), 127.3, 130.0, 133.5 (q, *J* = 33.8 Hz), 135.6, 141.5. **^{19}F NMR (377 MHz, CDCl$_3$):** δ = -62.99.

[22] W. Chen, K. Chen, W. Chen, M. Liu and H. Wu, *ACS Catal.*, 2019, **9**, 8110-8115.
[23] D. S. Raghuvanshi, A. K. Gupta and K. N. Singh, *Org. Lett.*, 2012, **14**, 4326-4329.
[24] V. Soni, U. N. Patel and B. Punji, *RSC Adv.*, 2015, **5**, 57472-57481.

8. Late-stage functionalization of pharmaceuticals

8.1. Functionalization of fenofibrate by Buchwald-Hartwig amination

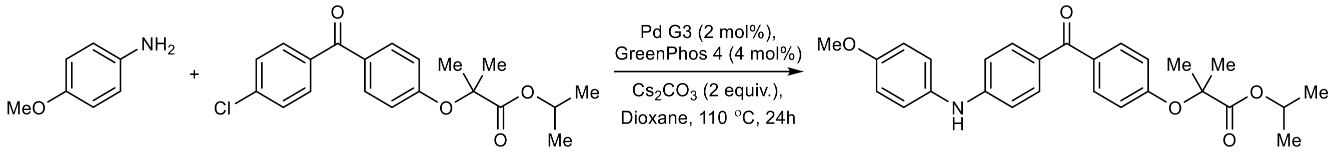

Inside of an argon-filled glovebox an oven dried 50 mL pressure tube was sequentially charged with fenofibrate (1 equiv., 0.831 mmol), *p*-anisidine (1.5 equiv.), Cs_2CO_3 (2 equiv.), Pd G3 (2 mol%) and GreenPhos 4 (4 mol%). This was followed by addition of anhydrous degassed dioxane (4.5 mL). The pressure tube was sealed with corresponding screw cup equipped with a septum and stabilizer and was removed from the glovebox. The resulting mixture was stirred at 110 °C for 24h. Afterwards, the reaction mixture was cooled down to room temperature, which was followed by the removal of volatiles using rotary evaporator. The product was isolated by flash chromatography using mixtures of heptane and EtOAc as eluent.

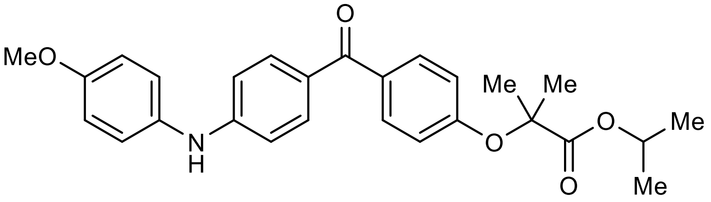

Isopropyl 2-(4-(4-((4-methoxyphenyl)amino)benzoyl)phenoxy)-2-methylpropanoate, 8a. Isopropyl 2-(4-(4-((4-methoxyphenyl)amino)benzoyl)phenoxy)-2-methylpropanoate was obtained from fenofibrate (0.300 g, 0.831 mmol) as a green solid (0.302 g, 81%). **m.p.** = 132-134 °C. **^{1}H NMR (400 MHz, $CDCl_3$):** δ = 1.20 (d, *J* = 6.3 Hz, 6H, 2xMe), 1.64 (s, 6H, 2xMe), 3.80 (s, 3H, OMe), 5.08 (hept, *J* = 6.3 Hz, 1H, O*CH*Me_2), 6.05 (br s, 1H, NH), 6.82-6.91 (m, 6H, Ar), 7.11-7.15 (m, 2H, Ar), 7.68-7.71 (m, 4H, Ar). **^{13}C NMR (101 MHz, $CDCl_3$):** δ = 21.7, 25.5, 55.7, 69.4, 79.4, 113.2, 114.9, 117.3, 124.5, 128.2, 131.6, 132.0, 132.7, 133.5, 149.8, 156.7, 158.8, 173.5, 194.2. **HRMS:** $[M+H]^+$ calcd for $C_{27}H_{30}NO_5$: 448.2118; found: 448.2118. **IR (ATR, cm^{-1}):** *v* = 3315 (s), 2980 (w), 2838 (w), 1729 (s), 1636 (w), 1586 (s), 1556 (s), 1531 (s), 1503 (s), 1384 (w), 1351 (s), 1290 (s), 1260 (m), 1238 (s), 1178 (s), 1173 (s), 1159 (s), 1148 (m), 1036 (w), 978 (m), 930 (s), 854 (s), 831 (s), 818 (s), 770 (s), 689 (m).

8.2. Arylation of fenofibrate by Stille coupling

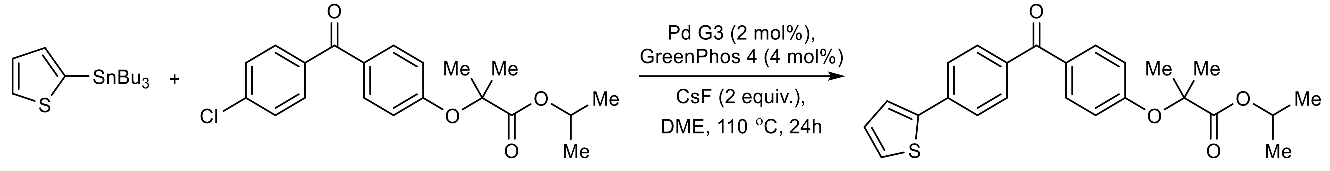

Inside of an argon-filled glovebox an oven dried 50 mL pressure tube was sequentially charged with fenofibrate (1.5 equiv.), 2-(tributylstannyl)thiophene (0.670 mmol, 1 equiv.), CsF (2 equiv.), Pd G3 (2 mol%) and GreenPhos 4 (4 mol%). This was followed by addition of anhydrous degassed DME (4 mL). The pressure tube was sealed with corresponding screw cup equipped with a septum and stabilizer and was removed from the glovebox. The resulting mixture was stirred at 110 °C for 24h. Afterwards, the reaction mixture was cooled down to room temperature, which was followed by the removal of volatiles using rotary evaporator. The product was isolated by flash chromatography using mixtures of heptane and EtOAc as eluent.

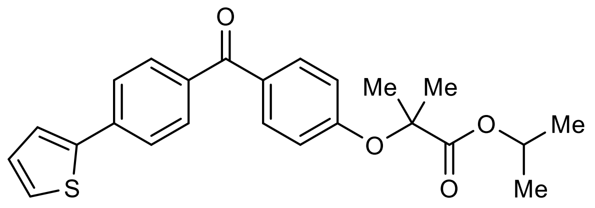

Isopropyl 2-methyl-2-(4-(4-(thiophen-2-yl)benzoyl)phenoxy)propanoate, 8b. Isopropyl 2-methyl-2-(4-(4-(thiophen-2-yl)benzoyl)phenoxy)propanoate was obtained from 2-(tributylstannyl)thiophene (0.250 g, 0.670 mmol) as a colorless oil (0.258 g, 94%). **^{1}H NMR (400 MHz, CDCl$_3$):** δ = 1.21 (d, *J* = 6.3 Hz, 6H, 2xMe), 1.67 (s, 6H, 2xMe), 5.09 (hept, *J* = 6.3 Hz, 1H, O*CH*Me$_2$), 6.86-6.90 (m, 2H, Ar), 7.11 (dd, *J* = 5.1, 3.6 Hz, 1H, thiophene), 7.35 (dd, *J* = 5.1, 1.1 Hz, 1H, thiophene), 7.42 (dd, *J* = 3.7, 1.1 Hz, 1H, thiophene), 7.68-7.71 (m, 2H, Ar), 7.75-7.80 (m, 4H, Ar). **^{13}C NMR (101 MHz, CDCl$_3$):** δ = 21.7, 25.5, 69.4, 79.5, 117.3, 124.5, 125.5, 126.4, 128.5, 130.7, 130.8, 132.1, 136.8, 138.0, 143.2, 159.6, 173.3, 194.8. **HRMS:** [M+H]$^+$ calcd for $C_{24}H_{25}O_4S$: 409.1468; found: 409.1470. **IR (ATR, cm^{-1}):** *v* = 2982 (w), 2933 (w), 1727 (s), 1649 (s), 1598 (s), 1578 (m), 1507 (w), 1466 (w), 1429 (w), 1385 (w), 1376 (w), 1316 (m), 1287 (s), 1277 (s), 1249 (s), 1176 (s), 1145 (s), 1100 (s), 972 (w), 929 (s), 853 (s), 826 (m), 732 (s), 699 (s).

8.3. Arylation of efavirenz by Suzuki-Miyaura coupling

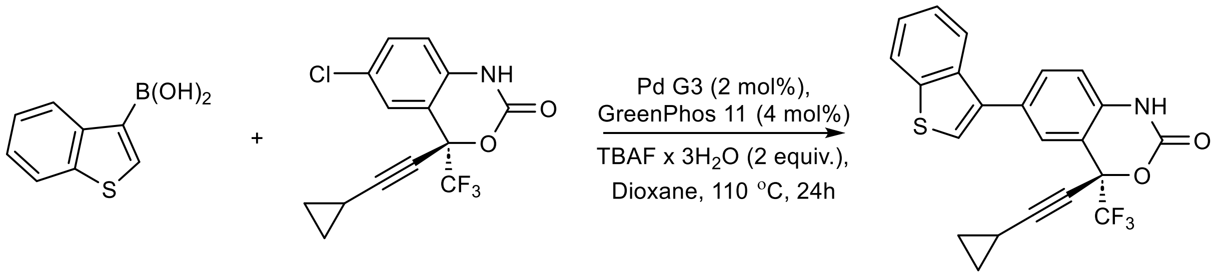

8c, 59%

Inside of an argon-filled glovebox an oven dried 50 mL pressure tube was sequentially charged with efavirenz (0.792 mmol, 1 equiv.), benzo[*b*]thien-3-ylboronic acid (1.5 equiv.), TBAF x $3H_2O$ (2 equiv.), Pd G3 (2 mol%) and GreenPhos 11 (4 mol%). This was followed by addition of anhydrous degassed dioxane (5 mL). The pressure tube was sealed with corresponding screw cup equipped with a septum and stabilizer and was removed from the glovebox. The resulting mixture was stirred at 110 °C for 24h. Afterwards, the reaction mixture was cooled down to room temperature, which was followed by the removal of volatiles using rotary evaporator. The product was isolated by flash chromatography using mixtures of heptane and EtOAc as eluent.

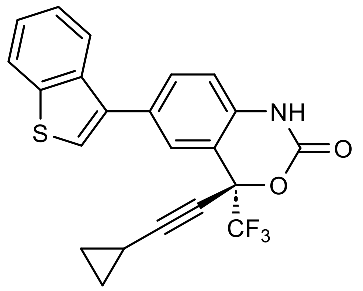

6-(Benzo[b]thiophen-3-yl)-4-(cyclopropylethynyl)-4-(trifluoromethyl)-1,4-dihydro-2H-benzo[d][1,3]oxazin-2-one, 8c. 6-(Benzo[*b*]thiophen-3-yl)-4-(cyclopropylethynyl)-4-(trifluoromethyl)-1,4-dihydro-2*H*-benzo[*d*][1,3]oxazin-2-one was obtained from efavirenz (0.250 g, 0.792 mmol) as a colorless solid (0.192 g, 59%). **m.p.** = 104-107 °C. **^{1}H NMR (400 MHz, CDCl$_3$):** δ = 1.17-1.23 (m, 2H, cyclopropyl), 1.25-1.32 (m, 2H, cyclopropyl), 2.32 (tt, *J* = 8.2, 4.8 Hz, 1H, cyclopropyl), 7.41-7.48 (m, 2H, Ar), 7.58 (d, *J* = 6.6 Hz, 2H, Ar), 7.96-7.99 (m, 3H, Ar), 8.17 (d, *J* = 8.7 Hz, 1H, Ar), 8.28-8.30 (m, 1H, NH). **^{13}C NMR (101 MHz, CDCl$_3$):** δ = 11.2, 18.4, 117.8 (q, *J* = 5.2 Hz), 121.7, 122.8, 123.2, 123.3 (q, *J* = 2.2 Hz), 123.8 (q, *J* = 274.6 Hz), 124.8, 124.9, 130.2, 131.2, 134.0 (q, *J* = 32 Hz), 134.5, 137.3, 137.8, 141.0, 143.5, 163.2. **^{19}F NMR (377 MHz, CDCl$_3$):** δ = -61.48. **HRMS:** $[M+H]^+$ calcd for $C_{22}H_{15}F_3NO_2S$: 414.0770; found: 414.0770. **IR (ATR, cm^{-1}):** ν = 3108 (w), 3010 (w), 2065 (w), 1613 (w), 1561 (w), 1498 (w), 1459 (w), 1405 (m), 1317 (m), 1311 (m), 1281 (m), 1269 (m), 1257 (s), 1208 (w), 1150 (s), 1147 (s), 1124 (s), 1099 (s), 1088 (s), 1022 (s), 916 (s), 843 (s), 785 (s), 759 (s), 714 (s).

8.4. Arylation of diazoxide by Suzuki-Miyaura coupling

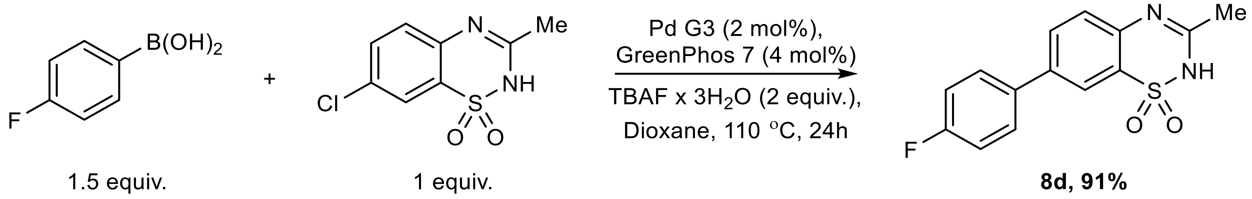

Inside of an argon-filled glovebox an oven dried 10 mL pressure tube was sequentially charged with diazoxide (0.390 mmol, 1 equiv.), 4-fluorophenylboronic acid (1.5 equiv.), TBAF x $3H_2O$ (2 equiv.), Pd G3 (2 mol%) and GreenPhos 7 (4 mol%). This was followed by addition of anhydrous degassed dioxane (2 mL). The pressure tube was sealed with corresponding screw cup equipped with a septum and stabilizer and was removed from the glovebox. The resulting mixture was stirred at 110 °C for 24h. Afterwards, the reaction mixture was cooled down to room temperature, which was followed by the removal of volatiles using rotary evaporator and addition of 1,3,5-trimethoxybenzene (1 equiv., internal standard). The resulting mixture was treated with DMSO-d6 (2 mL), thoroughly shaken for 2 minutes, and then centrifuged (Figure S2-S9). This was followed by ^{1}H NMR analysis of an aliquot taken from the mixture (see Spectra S131-S135).

8.5. Arylation of haloperidol by Suzuki-Miyaura coupling

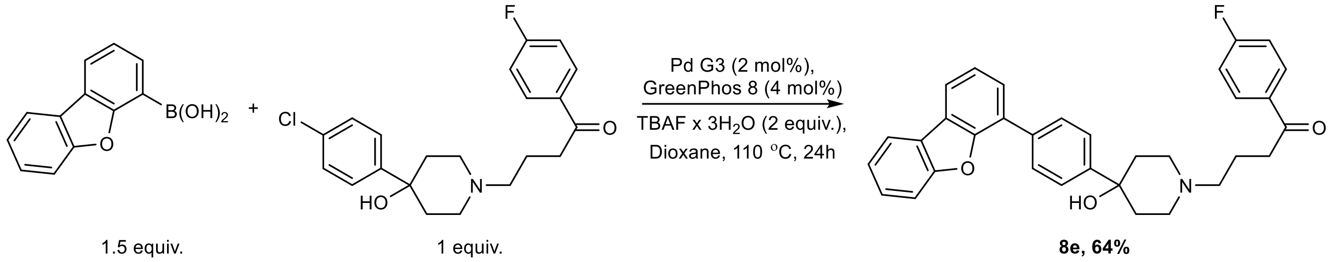

Inside of an argon-filled glovebox an oven dried 50 mL pressure tube was sequentially charged with haloperidol (0.798 mmol, 1 equiv.), 4-(dibenzofuranyl)boronic acid (1.5 equiv.), TBAF x $3H_2O$ (2 equiv.), Pd G3 (2 mol%) and GreenPhos 8 (4 mol%). This was followed by addition of anhydrous degassed dioxane (5 mL). The pressure tube was sealed with corresponding screw cap equipped with a septum and stabilizer and was removed from the glovebox. The resulting mixture was stirred at 110 °C for 24h. Afterwards, the reaction mixture was cooled down to room temperature, which was followed by the removal of volatiles using rotary evaporator. The product was isolated by flash chromatography using mixtures of EtOAc and Et_3N as eluent.

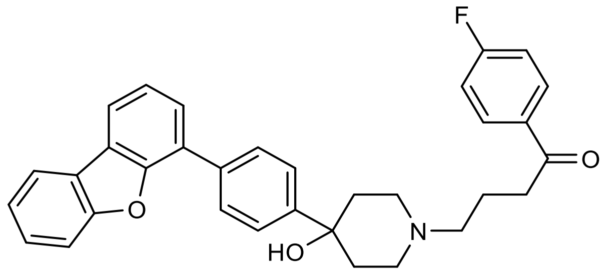

4-(4-(4-(Dibenzo[b,d]furan-4-yl)phenyl)-4-hydroxypiperidin-1-yl)-1-(4-fluorophenyl)butan-1-one, 8e. 4-(4-(4-(Dibenzo[*b*,*d*]furan-4-yl)phenyl)-4-hydroxypiperidin-1-yl)-1-(4-fluorophenyl)butan-1-one was obtained from haloperidol (0.300 g, 0.798 mmol) as a yellow solid (0.258 g, 64%). The product was obtained as a mixture of rotamers. **m.p.** = 162-164 °C. **^{1}H NMR (400 MHz, DMSO-d6):** δ = 1.43-1.69 (m, 3H, CH_2), 1.77-1.90 (m, 3H, CH_2), 2.28-2.42 (m, 4H, CH_2), 2.55-2.65 (m, 2H, CH_2), 2.95-3.01 (m, 2H, CH_2), 4.82-4.84 (m, 1H, OH), 7.13-7.56 (m, 9H, Ar), 7.67-7.75 (m, 1H, Ar), 7.82-7.84 (m, 1H, Ar), 8.05-8.20 (m, 4H, Ar). **^{13}C NMR (101 MHz, DMSO-d6):** δ = 22.2, 35.8, 35.9, 37.9, 38.0, 38.1, 49.0, 49.1, 49.2, 55.1, 57.4, 57.5, 69.7, 69.8, 69.9, 112.0, 115.8 (dd, *J* = 21.9, 2.4 Hz), 120.3, 121.4, 123.4, 123.7, 123.9, 124.5, 124.9, 125.2, 125.4, 126.2, 126.9, 127.0, 127.8, 127.9, 128.2, 131.0 (dd, *J* = 9.5, 2.9 Hz), 133.7, 134.1 (d, *J* = 2.9 Hz), 149.4, 150.1, 150.4, 152.8, 155.6, 163.7, 166.2, 198.4, 198.5. **^{19}F NMR (377 MHz, DMSO-d6):** δ = -106.86, -106.89. **HRMS:** $[M+H]^+$ calcd for $C_{33}H_{31}FNO_3$: 508.2282; found: 508.2282. **IR (ATR, cm^{-1}):** *v* = 2922 (w), 2894 (w), 2827 (w), 1685 (s), 1598 (s), 1507 (w), 1450 (m), 1412 (m), 1314 (w), 1362 (w), 1226 (s), 1188 (s), 1158 (s), 1138 (s), 1114 (s), 1048 (s), 997 (s), 964 (m), 921 (w), 828 (s), 748 (s), 744 (s), 735 (s).

9. Computational methods

9.1. Conformational search of lowest-energy conformers

For both linear [R_3PAuCl] and tetrahedral [$R_3PNi(CO)_3$] complexes, we carried out an exhaustive conformational search using the Conformer-Rotamer Ensemble Sampling Tool (CREST)[25] and a subsequent conformer ensemble reranking with CENSO program.[26] The CREST conformational search was performed at the GFN2-xTB level,[27] including the ALPB model[28] for solvation in THF and using an energy window of 6.0 kcal mol^{-1}. Further refinement on the electronic energy description was performed with CENSO at the DFT B97-3c/def2-SV(P)[29,30,31] and using the ORCA code.[32] This protocol has shown efficiency when exploring conformational energies of organometallic complexes.[33,34]

The conformational search generated a large number of stable conformers due to the high fluxionality of the GreenPhos ligands. Therefore, the refinement of the structures with CENSO was crucial to capture most of the lowest-energy structures. Among the best structures, the ligands adapt different conformations depending on the orientation of the alkyl substituents, which may be pointing away (open conformation) or towards (close conformation) the metal center. Furthermore, it should be noted that the most stable conformation of a single GreenPhos ligand can have a different arrangement when the AuCl fragment is replaced by $Ni(CO)_3$. For instance, in the less strained linear gold complex, GreenPhos 3 ligand adopts a close arrangement, whereas in the tetrahedral nickel complex, the most favored conformer exhibits an open conformation with the alkyl groups pointing away from the metal.

9.2. Structure optimizations

The lowest-energy conformers from the CREST/CENSO sampling were reoptimized and classified as local minima at the density-functional theory (DFT) level using the BP86-D3 functional[35,36,37] in conjunction with the Slater-type orbitals (STO) TZ2P basis sets.[38] Solvation effects were considered through the implicit COSMO model[39,40] for simulation bulk solvation in THF. The calculations were performed including scalar relativistic effects using the zeroth-order regular approximation (ZORA)

---

[25] P. Pracht, F. Bohle and S. Grimme, *Phys. Chem. Chem. Phys.*, 2020, **22**, 7169-7192.
[26] S. Grimme, F. Bohle, A. Hansen, P. Pracht, S. Spicher and M. Stahn, *J. Phys. Chem. A*, 2021, **125**, 4039-4054.
[27] C. Bannwarth, S. Ehlert and S. Grimme, *J. Chem. Theory and Comput.*, 2019, **15**, 1652-1671.
[28] S. Ehlert, M. Stahn, S. Spicher and S. Grimme, *J. Chem. Theory and Comput.*, 2021, **17**, 4250-4261.
[29] J. G. Brandenburg, C. Bannwarth, A. Hansen and S. Grimme, *J. Chem. Phys.*, 2018, **148**, 064104.
[30] F. Weigend and R. Ahlrichs, *Phys. Chem. Chem. Phys.*, 2005, **7**, 3297-3305.
[31] F. Weigend, *Phys. Chem. Chem. Phys.*, 2006, **8**, 1057-1065.
[32] F. Neese, F. Wennmohs, U. Becker and C. Riplinger, *J. Chem. Phys.*, 2020, **152**, 224108.
[33] M. Bursch, A. Hansen, P. Pracht, J. T. Kohn and S. Grimme, *Phys. Chem. Chem. Phys.*, 2021, **23**, 287-299.
[34] I. Iribarren and C. Trujillo, *J. Chem. Inf. Model*, 2022, **62**, 5568-5580.
[35] A. D. Becke, *Phys. Rev. A*, 1988, **38**, 3098-3100.
[36] J. P. Perdew, *Phys. Rev. B*, 1986, **33**, 8822-8824.
[37] S. Grimme, J. Antony, S. Ehrlich and H. Krieg, *J. Chem. Phys.*, 2010, **132**, 154104.
[38] E. van Lenthe and E. J. Baerends, *J. Comput. Chem.*, 2003, **24**, 1142-1156.
[39] A. Klamt and G. Schüürmann, *J. Chem. Soc., Perkin Trans. 2*, 1993, 799-805.
[40] C. C. Pye and T. Ziegler, *Theor. Chem. Acc.*, 1999, **101**, 396-408.

Hamiltonian,[41,42] as implemented in the ADF software package.[43] A dataset collection of the optimized structures is available in the ioChem-BD repository[44] and can be accessed *via* https://doi.org/10.19061/iochem-bd-6-260.

9.3. Calculation of steric maps

The steric maps were calculated using structures fully optimized at the BP86-D3/TZ2P level of theory. To build the steric maps, the overall complex was oriented in a Cartesian frame, selecting the metal at the origin, the M–P bond along the *z*-axis, and the R_3 functional group in the *xz*-plane (Figure S19). The R_3 functional group corresponds to the least bulky substituents, which can be either a primary alkyl group or an alkoxy/aryloxy group (Scheme 4). In case of phosphinites the R_3 functional group corresponds to two tertiary alkyl groups and an alkoxy/aryloxy group (Scheme 4). The percent buried volume ($\%V_{bur}$) and the topographic steric maps were calculated using the Samb*V*ca software.[45,46] In this work, the value for the sphere radius was set to 3.5 Å, the mesh of 0.1 Å was used to scan the sphere for buried voxels, and the Bondi raddi was scaled by 1.17. Only the phosphine (PR_3) ligands have been considered in the definition of the catalytic pocket, hydrogen atoms were omitted.

We first analyzed some representative phosphine ligands as reference systems (see Table S30). These ligands showed a clear difference in $\%V_{bur}$, where the PMe_3 and the JohnPhos ligands are the least and most bulky, respectively. Our computed $\%V_{bur}$ values for PMe_3 (24%), PPh_3 (31%), *t*Bu_3P (38%), and JohnPhos (52%) ligands are in good agreement to previously reported values of 22.2%, 29.9%, 38.1%, and 50.9%, respectively.[47] Note that the protocol used in this work do not consider any geometry restriction, *i.e.*, we do not keep the M–P distance fixed at 2.28 Å. In fact, this restriction was shown to have a certain impact on the $\%V_{bur}$ computed with a shorter M–P bond distance of 2.00.[47]

For GreenPhos 1-20 ligands, we observed variety of shapes in the computed catalytic pockets (see Tables S31-S33). As expected, the computed $\%V_{bur}$ and steric maps are influenced by the coordination environment of the phosphine ligand. In general, the $\%V_{bur}$ decreases when moving from the linear gold complex to the more hindered tetrahedral nickel complex. Furthermore, the steric bulkiness is also influenced by the ligand conformational changes. This behavior has been reported previously for other types of flexible phosphine ligands.[48]

---

[41] C. Chang, M. Pelissier and P. Durand, *Phys. Scr.*, 1986, **34**, 394-404.
[42] E. v. Lenthe, E. J. Baerends and J. G. Snijders, *J. Chem. Phys.*, 1993, **99**, 4597-4610.
[43] E. J. Baerends and co-workers, ADF 2023, SCM, Theoretical Chemistry, Vrije Universiteit, Amsterdam, The Netherlands, http://www.scm.com.
[44] M. Álvarez-Moreno, C. de Graaf, N. López, F. Maseras, J. M. Poblet and C. Bo, *J. Chem. Inf. Model*, 2015, **55**, 95-103.
[45] L. Falivene, Z. Cao, A. Petta, L. Serra, A. Poater, R. Oliva, V. Scarano and L. Cavallo, *Nature Chem.*, 2019, **11**, 872-879.
[46] L. Falivene, R. Credendino, A. Poater, A. Petta, L. Serra, R. Oliva, V. Scarano and L. Cavallo, *Organometallics*, 2016, **35**, 2286-2293.
[47] H. Clavier and S. P. Nolan, *Chem. Commun*., 2010, **46**, 841-861.
[48] J. Jover and J. Cirera, *Dalton Trans*., 2019, **48**, 15036-15048.

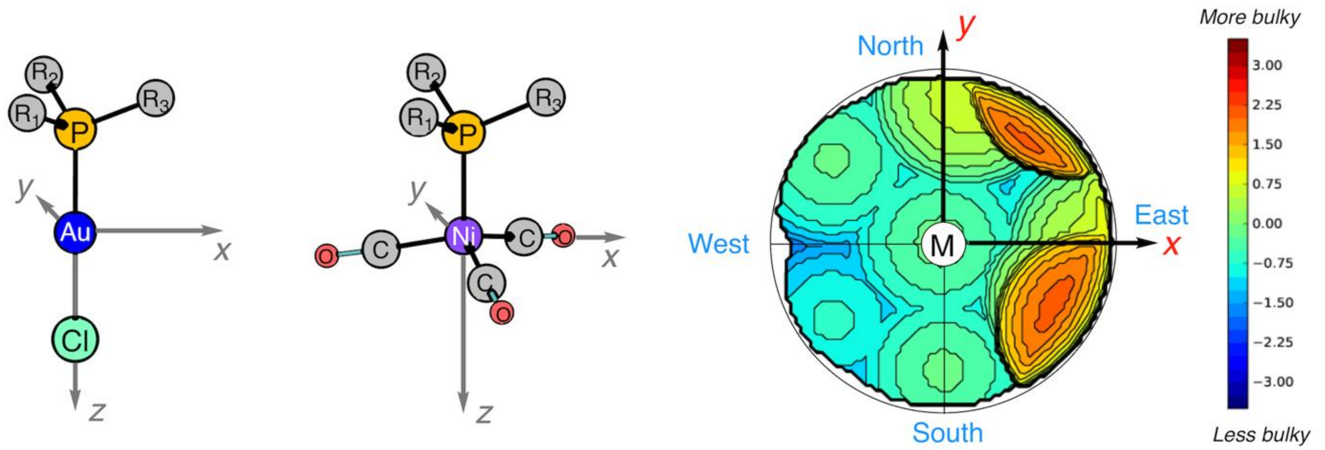

Figure S19. The linear [R_3PAuCl] and tetrahedral [$R_3PNi(CO)_3$] complexes studied in this work are oriented according to the diagrams on the left. The steric map is reported on the right, and it is viewed down the *z*-axis, using the labeling of the quadrants as North, South, East, and West. The isocontour scheme, in Å, indicate in red and blue the more- and less-hindered zones in the catalytic pocket, respectively.

Table S30. Steric maps of selected reference phosphine (PR_3) ligands comparing linear [R_3PAuCl] and tetrahedral [$R_3PNi(CO)_3$] coordination environments.

PR_3 ligand[a]	Complexes[b]	
PMe_3	Me_3PAuCl	$Me_3PNi(CO)_3$
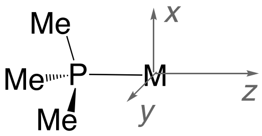	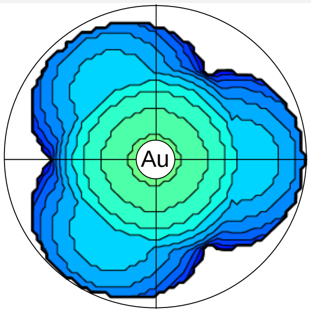 %V_{bur} = 24.0	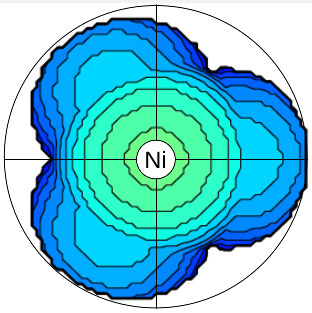 %V_{bur} = 23.9
PPh_3	Ph_3PAuCl	$Ph_3PNi(CO)_3$
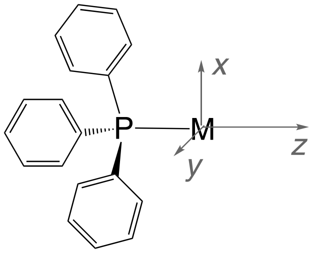	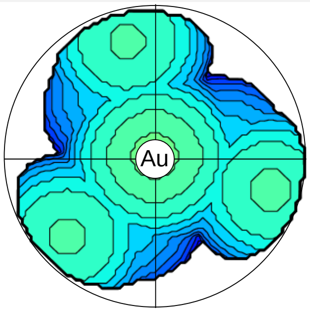 %V_{bur} = 30.8	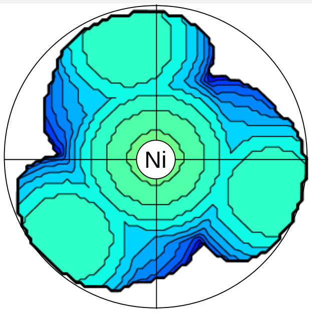 %V_{bur} = 30.3
tBu$_2$PMe	tBu$_2$MePAuCl	tBu$_2$MePNi(CO)$_3$
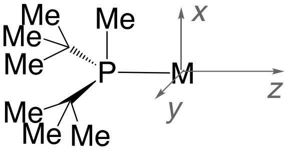	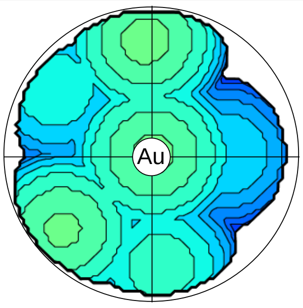 %V_{bur} = 33.5	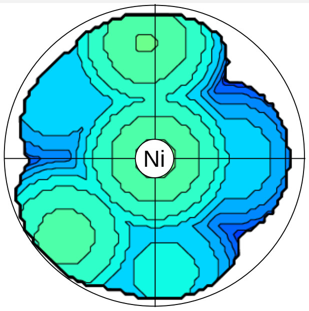 %V_{bur} = 31.8
tBu$_3$P	tBu$_3$PAuCl	tBu$_3$PNi(CO)$_3$
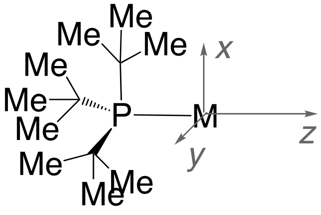	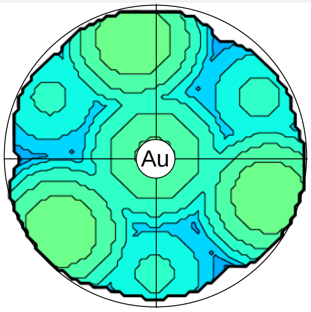 %V_{bur} = 38.3	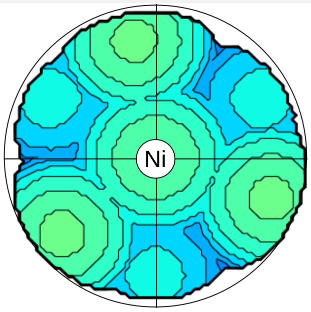 %V_{bur} = 36.1

JohnPhos	JohnPhos-AuCl	JohnPhos-$Ni(CO)_3$
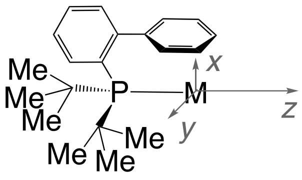	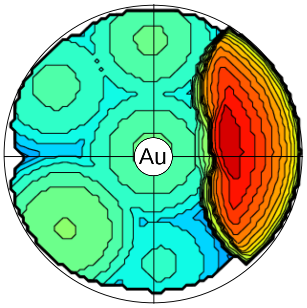	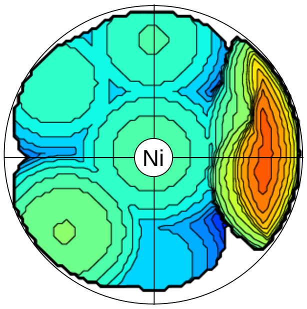
	**%V_{bur} = 51.8**	**%V_{bur} = 42.6**

[a] The orientation of the ligands is consistent with the orientation in Figure S19. [b] The labeling of the quadrants as North, South, East, and West is consistent with the labeling in Figure S19, and it applies to all the steric maps in this work.

Table S31. Steric maps of GreenPhos 1-3 ligands comparing linear [R_3PAuCl] and tetrahedral [$R_3PNi(CO)_3$] coordination environments.

PR_3 ligand[a]	Complexes[b]	
**GreenPhos 1**	R_3PAuCl	$R_3PNi(CO)_3$
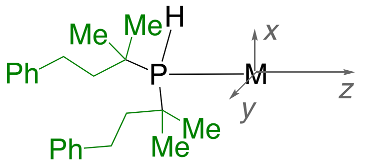	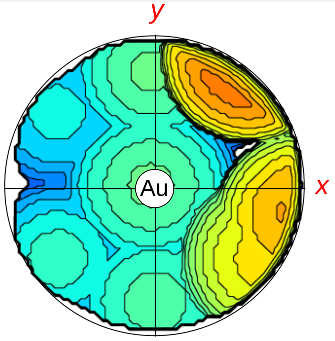 **%V_{bur} = 46.7**	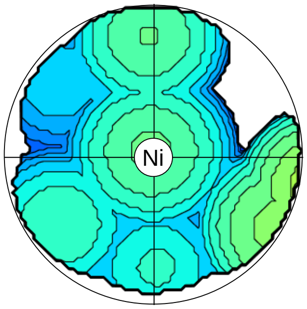 **%V_{bur} = 33.9**
**GreenPhos 2**	R_3PAuCl	$R_3PNi(CO)_3$
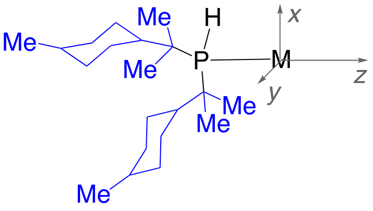	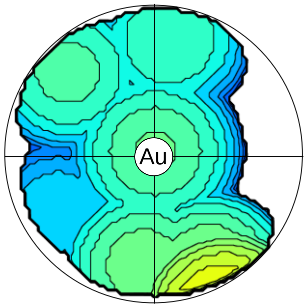 **%V_{bur} = 34.4**	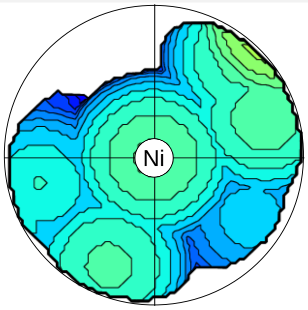 **%V_{bur} = 31.2**
**GreenPhos 3**	R_3PAuCl	$R_3PNi(CO)_3$
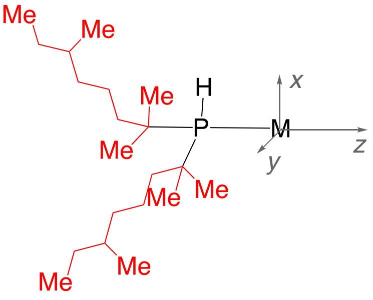	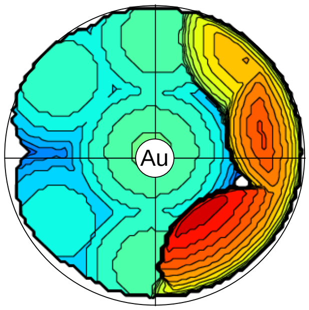 **%V_{bur} = 48.4**	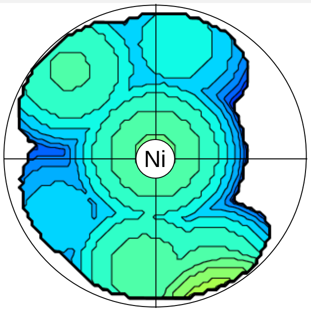 **%V_{bur} = 31.2**

[a] The orientation of the ligands is consistent with the orientation in Figure S19. [b] The labeling of the quadrants as North, South, East, and West is consistent with the labeling in Figure S19, and it applies to all the steric maps in this work.

Table S32. Steric maps of GreenPhos 4-10 ligands comparing linear [R_3PAuCl] and tetrahedral [$R_3PNi(CO)_3$] coordination environments.

PR_3 ligand[a]	Complexes[b]	
**GreenPhos 4**	R_3PAuCl	$R_3PNi(CO)_3$
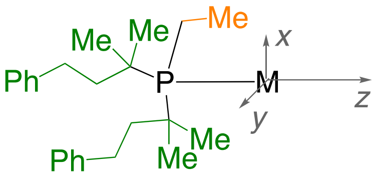	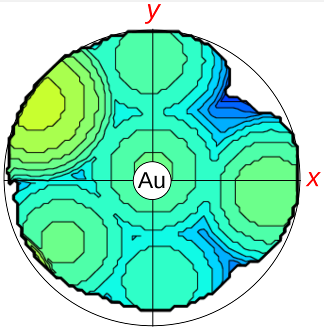 **%V_{bur} = 40.1**	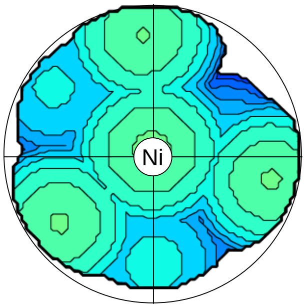 **%V_{bur} = 34.7**
**GreenPhos 5**	R_3PAuCl	$R_3PNi(CO)_3$
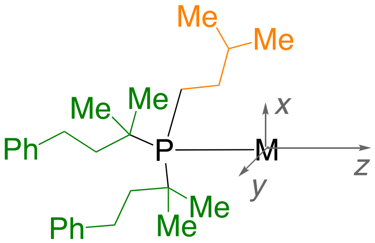	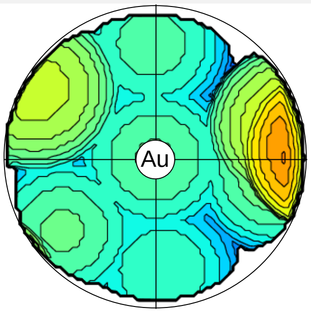 **%V_{bur} = 45.0**	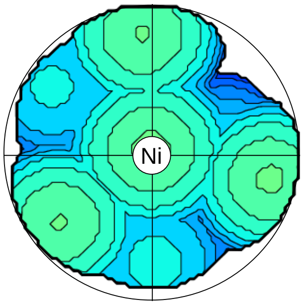 **%V_{bur} = 34.7**
**GreenPhos 6**	R_3PAuCl	$R_3PNi(CO)_3$
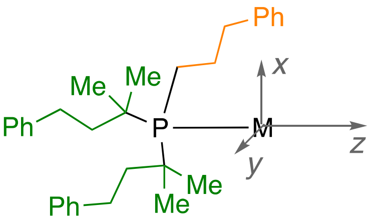	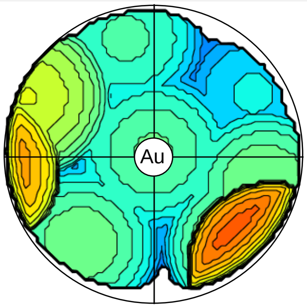 **%V_{bur} = 46.8**	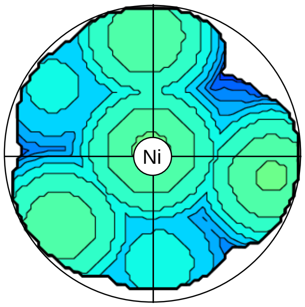 **%V_{bur} = 34.7**
**GreenPhos 7**	R_3PAuCl	$R_3PNi(CO)_3$
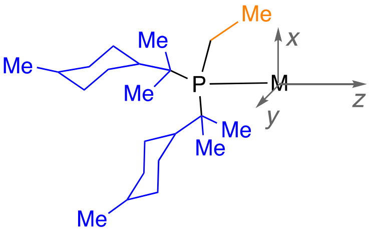	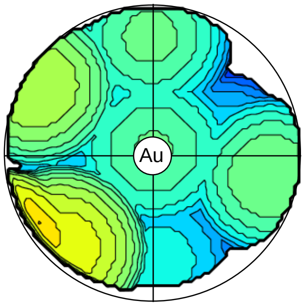 **%V_{bur} =43.5**	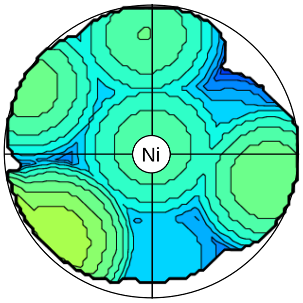 **%V_{bur} = 38.2**

GreenPhos 8	R_3PAuCl	$R_3PNi(CO)_3$
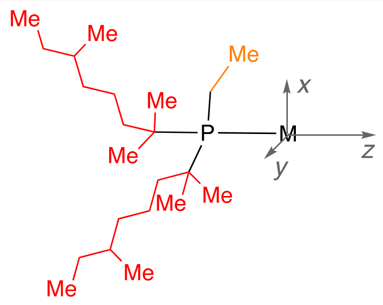	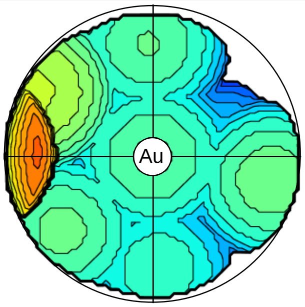	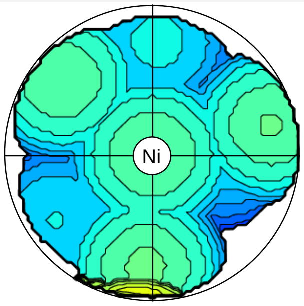
	**%V_{bur} = 42.0**	**%V_{bur} = 35.0**
**GreenPhos 9**	R_3PAuCl	$R_3PNi(CO)_3$
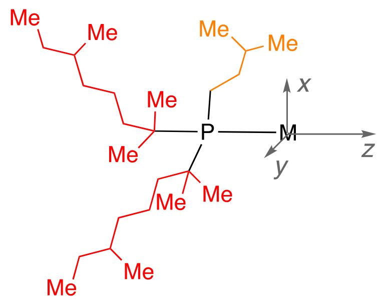	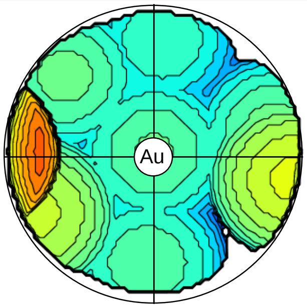	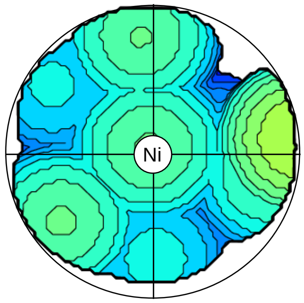
	**%V_{bur} = 45.2**	**%V_{bur} = 36.3**
**GreenPhos 10**	R_3PAuCl	$R_3PNi(CO)_3$
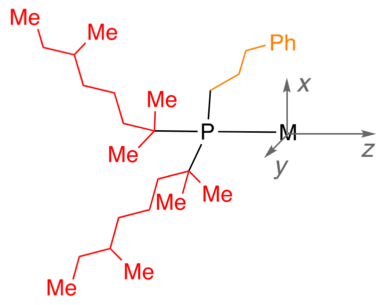	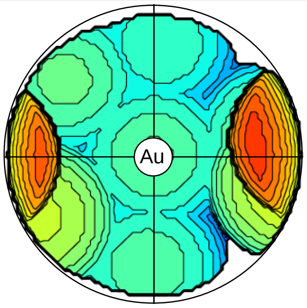	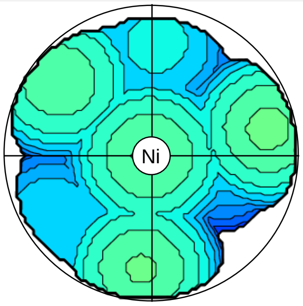
	**%V_{bur} = 48.7**	**%V_{bur} = 34.7**

[a] The orientation of the ligands is consistent with the orientation in Figure S19. [b] The labeling of the quadrants as North, South, East, and West is consistent with the labeling in Figure S19, and it applies to all the steric maps in this work.

Table S33. Steric maps of GreenPhos 11-20 ligands comparing linear [R_3PAuCl] and tetrahedral [$R_3PNi(CO)_3$] coordination environments.

PR_3 ligand[a]	Complexes[b]	
**GreenPhos 11**	R_3PAuCl	$R_3PNi(CO)_3$
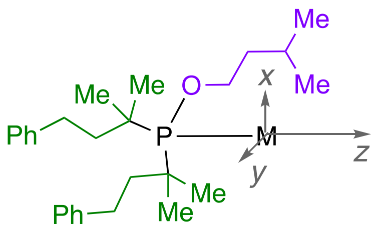	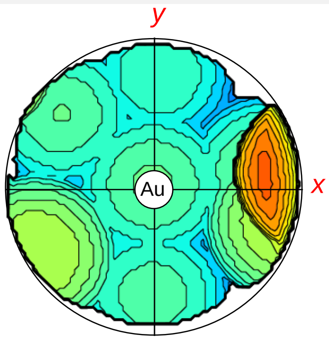 **%V_{bur} = 45.0**	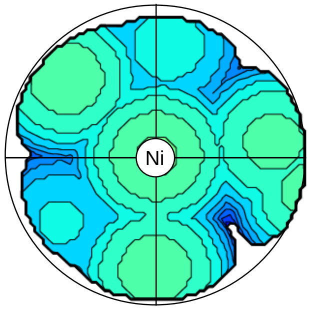 **%V_{bur} = 34.9**
**GreenPhos 12**	R_3PAuCl	$R_3PNi(CO)_3$
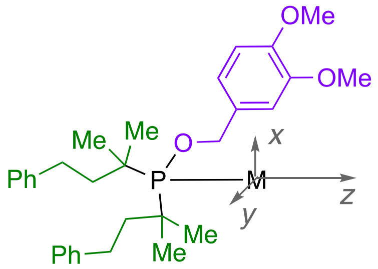	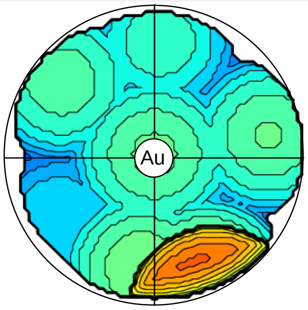 **%V_{bur} = 40.7**	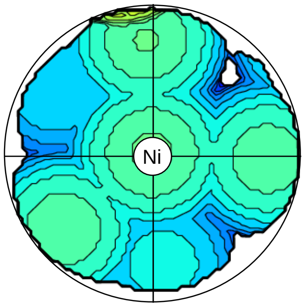 **%V_{bur} = 35.0**
**GreenPhos 13**	R_3PAuCl	$R_3PNi(CO)_3$
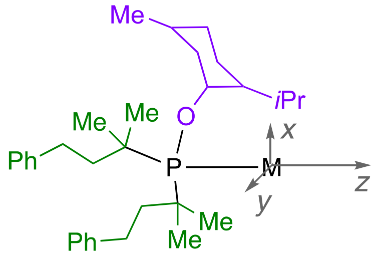	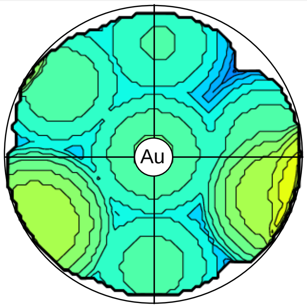 **%V_{bur} = 42.5**	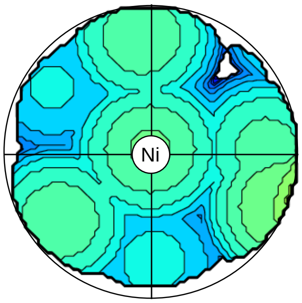 **%V_{bur} =36.1**
**GreenPhos 14**	R_3PAuCl	$R_3PNi(CO)_3$
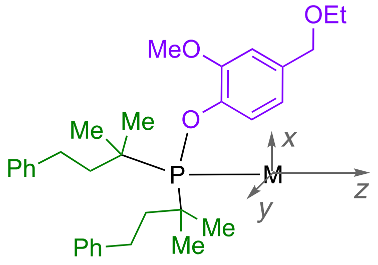	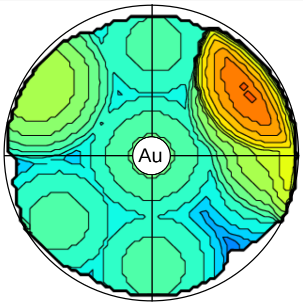 **%V_{bur} = 46.7**	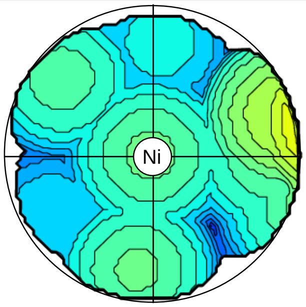 **%V_{bur} = 37.8**

<table>
<tr><th>GreenPhos 15</th><th>R_3PAuCl</th><th>$R_3PNi(CO)_3$</th></tr>
<tr><td>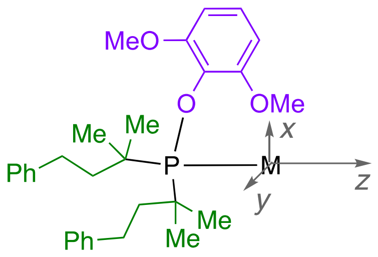
</td><td>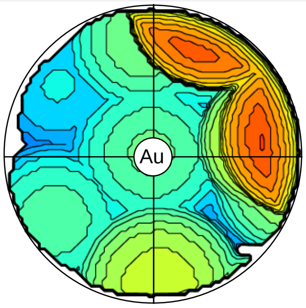

%V_{bur} = 51.5</td><td>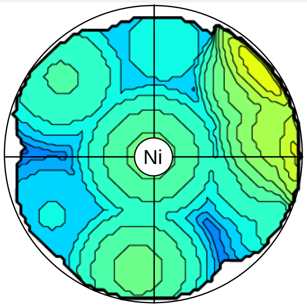

%V_{bur} = 38.8</td></tr>
<tr><th>GreenPhos 16</th><th>R_3PAuCl</th><th>$R_3PNi(CO)_3$</th></tr>
<tr><td>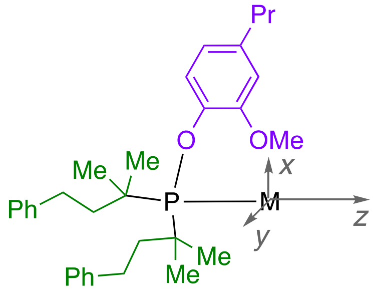
</td><td>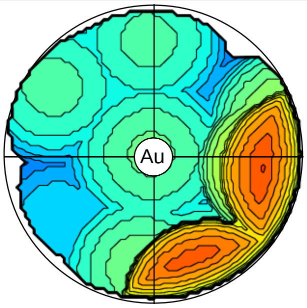

%V_{bur} = 48.1</td><td>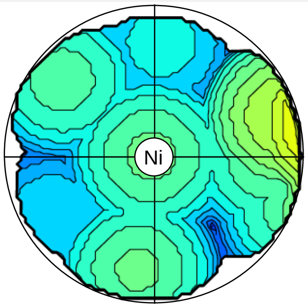

%V_{bur} = 37.9</td></tr>
<tr><th>GreenPhos 17</th><th>R_3PAuCl</th><th>$R_3PNi(CO)_3$</th></tr>
<tr><td>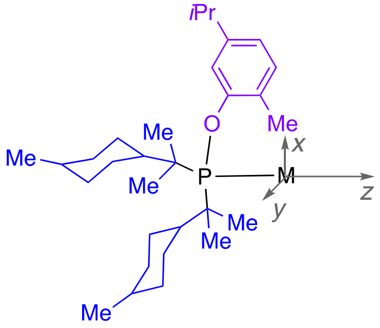
</td><td>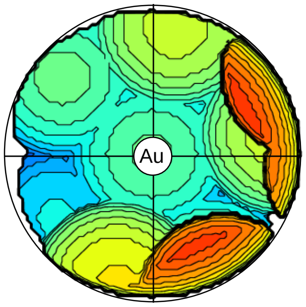

%V_{bur} = 53.6</td><td>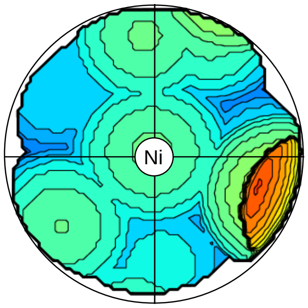

%V_{bur} = 39.6</td></tr>
<tr><th>GreenPhos 18</th><th>R_3PAuCl</th><th>$R_3PNi(CO)_3$</th></tr>
<tr><td>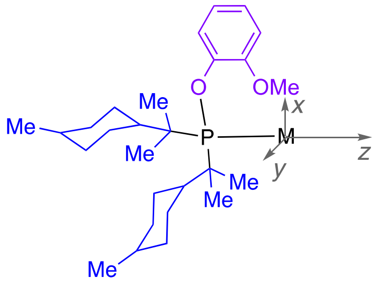
</td><td>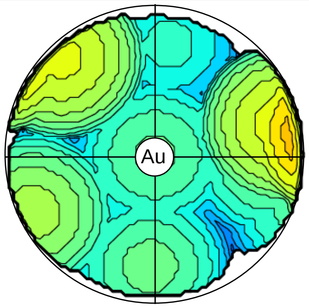

%V_{bur} = 46.4</td><td>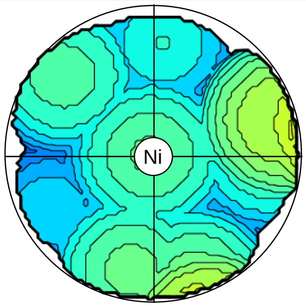

%V_{bur} = 38.8</td></tr>
</table>

<table>
<tr><th>GreenPhos 19</th><th>R_3PAuCl</th><th>$R_3PNi(CO)_3$</th></tr>
<tr><td>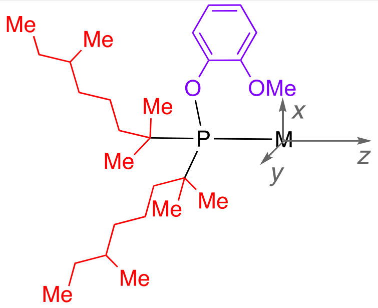</td><td>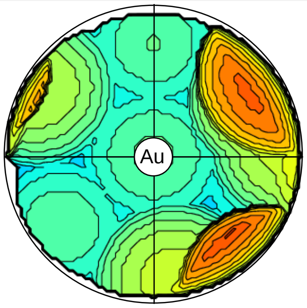
%V_{bur} = 53.5</td><td>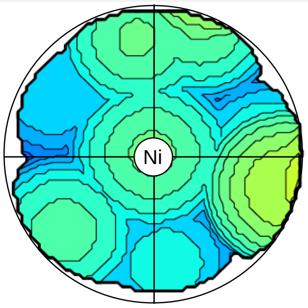
%V_{bur} = 38.0</td></tr>
<tr><th>GreenPhos 20</th><th>R_3PAuCl</th><th>$R_3PNi(CO)_3$</th></tr>
<tr><td>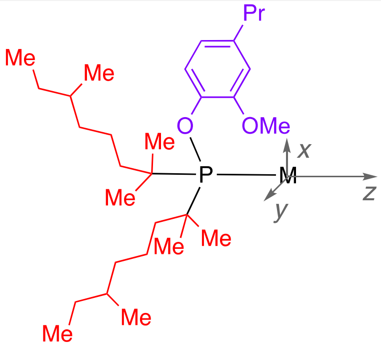</td><td>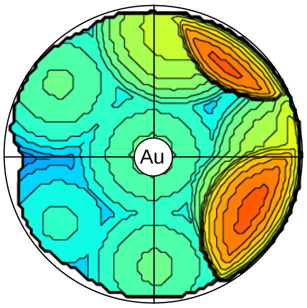
%V_{bur} = 50.0</td><td>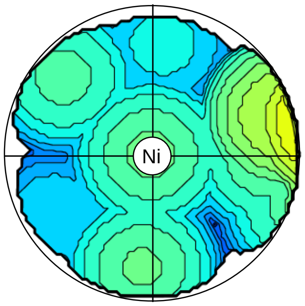
%V_{bur} = 37.8</td></tr>
</table>

[a] The orientation of the ligands is consistent with the orientation in Figure S19. [b] The labeling of the quadrants as North, South, East, and West is consistent with the labeling in Figure S19, and it applies to all the steric maps in this work.

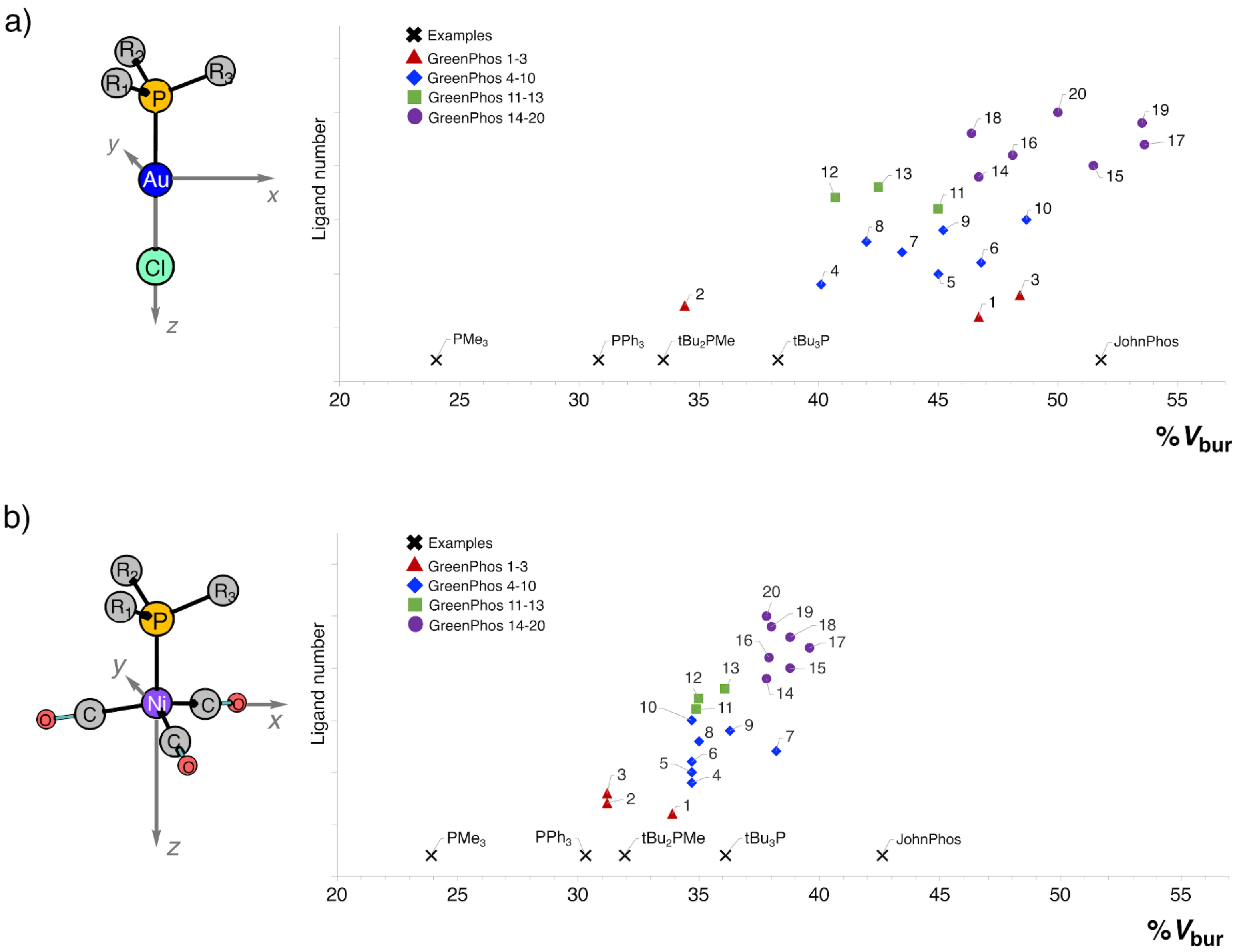

Figure S20. Graphical summary of the computed percentage buried volumes (%V_{bur}) of GreenPhos 1-20 ligands for a) linear [R_3PAuCl] and b) tetrahedral [$R_3PNi(CO)_3$] complexes. Color codes are used to differentiate between the different ligand classes.

10. Copies of spectra

Spectrum S1. The ^{1}H NMR spectrum of *2-(4-methylcyclohexyl)propan-2-ol* in $CDCl_3$.

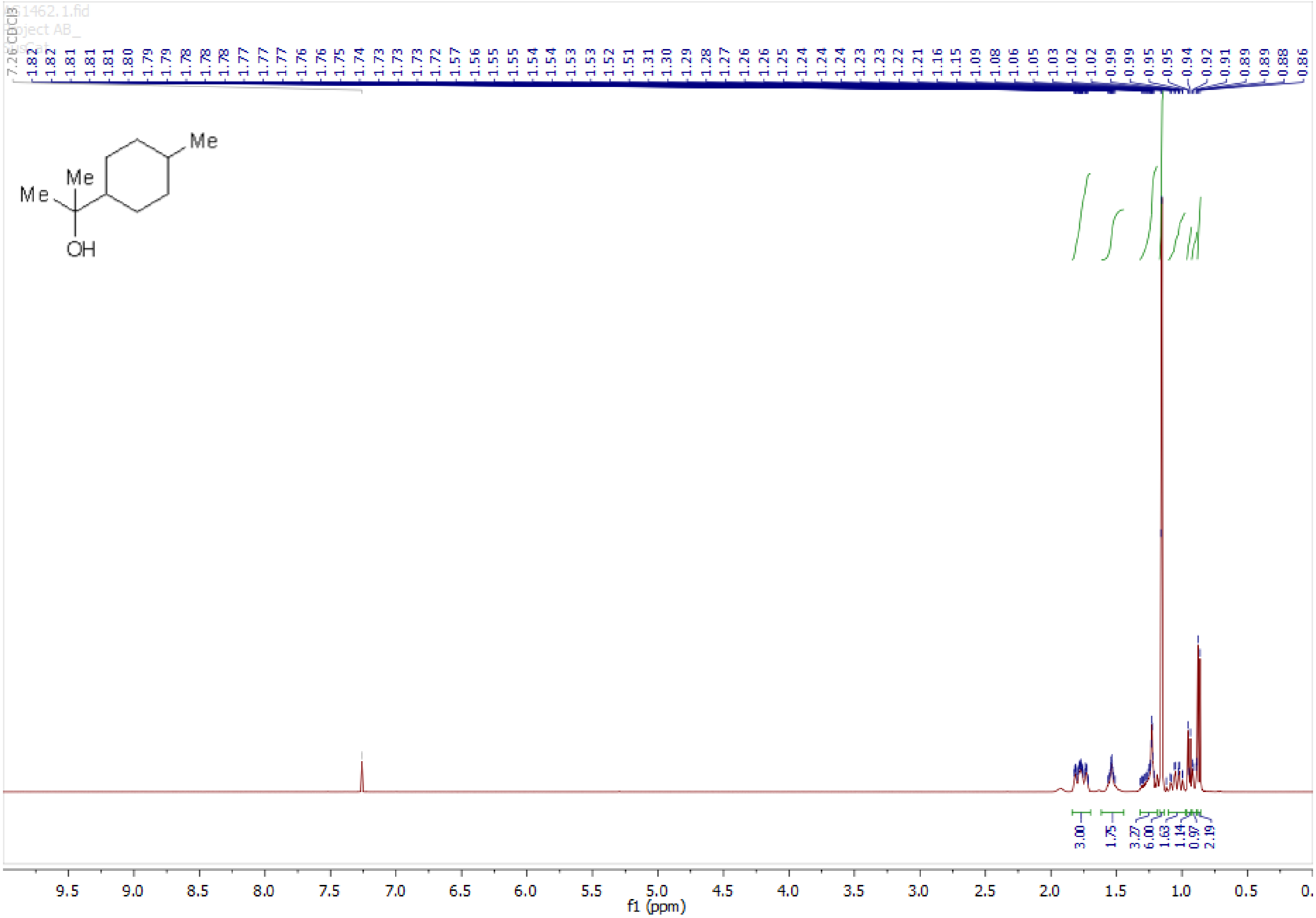

Spectrum S2. The ^{13}C NMR spectrum of *2-(4-methylcyclohexyl)propan-2-ol* in $CDCl_3$.

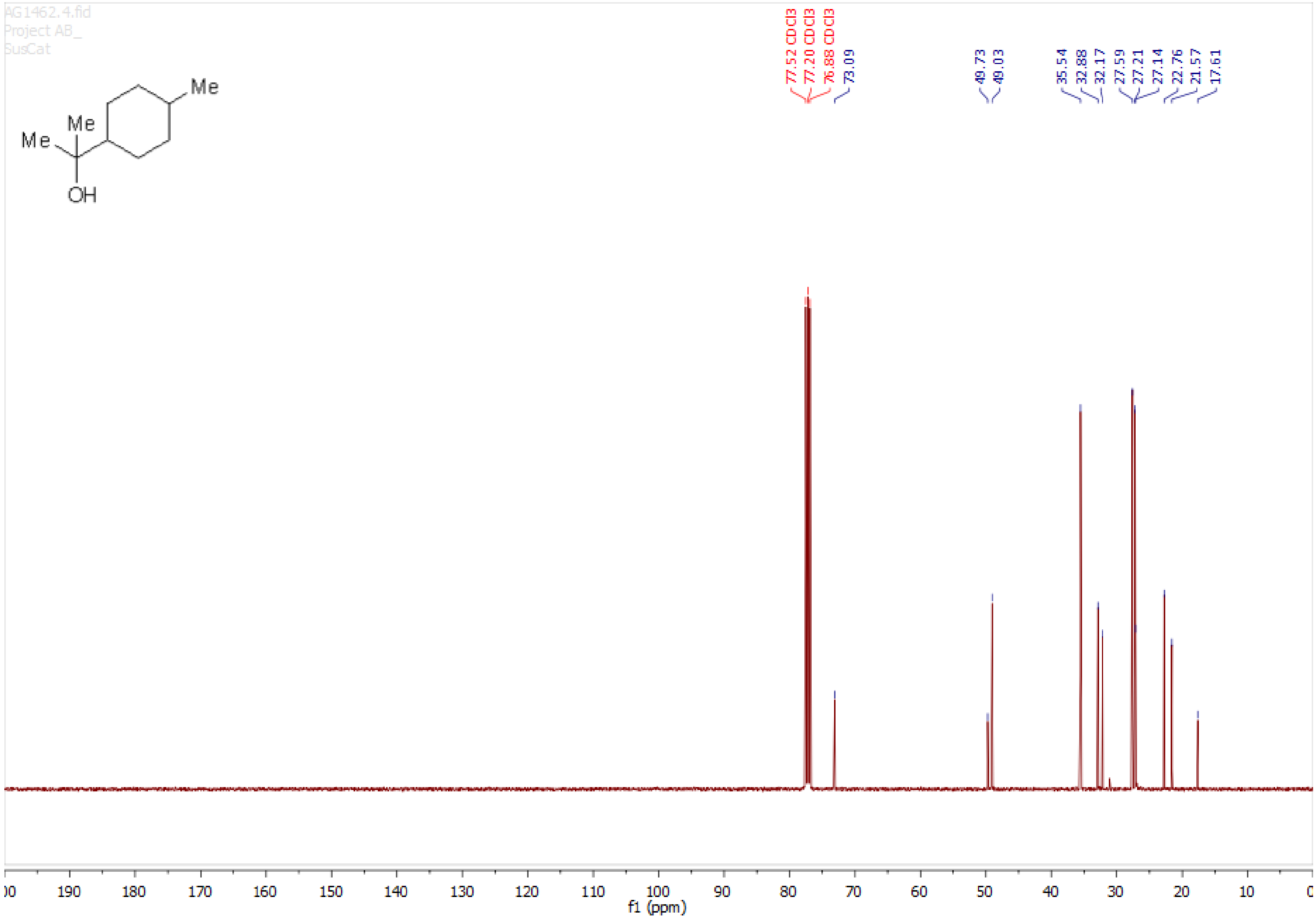

Spectrum S3. The ^{1}H NMR spectrum of *2,6-Dimethyloctan-2-ol (tetrahydromyrcenol)* in $CDCl_3$.

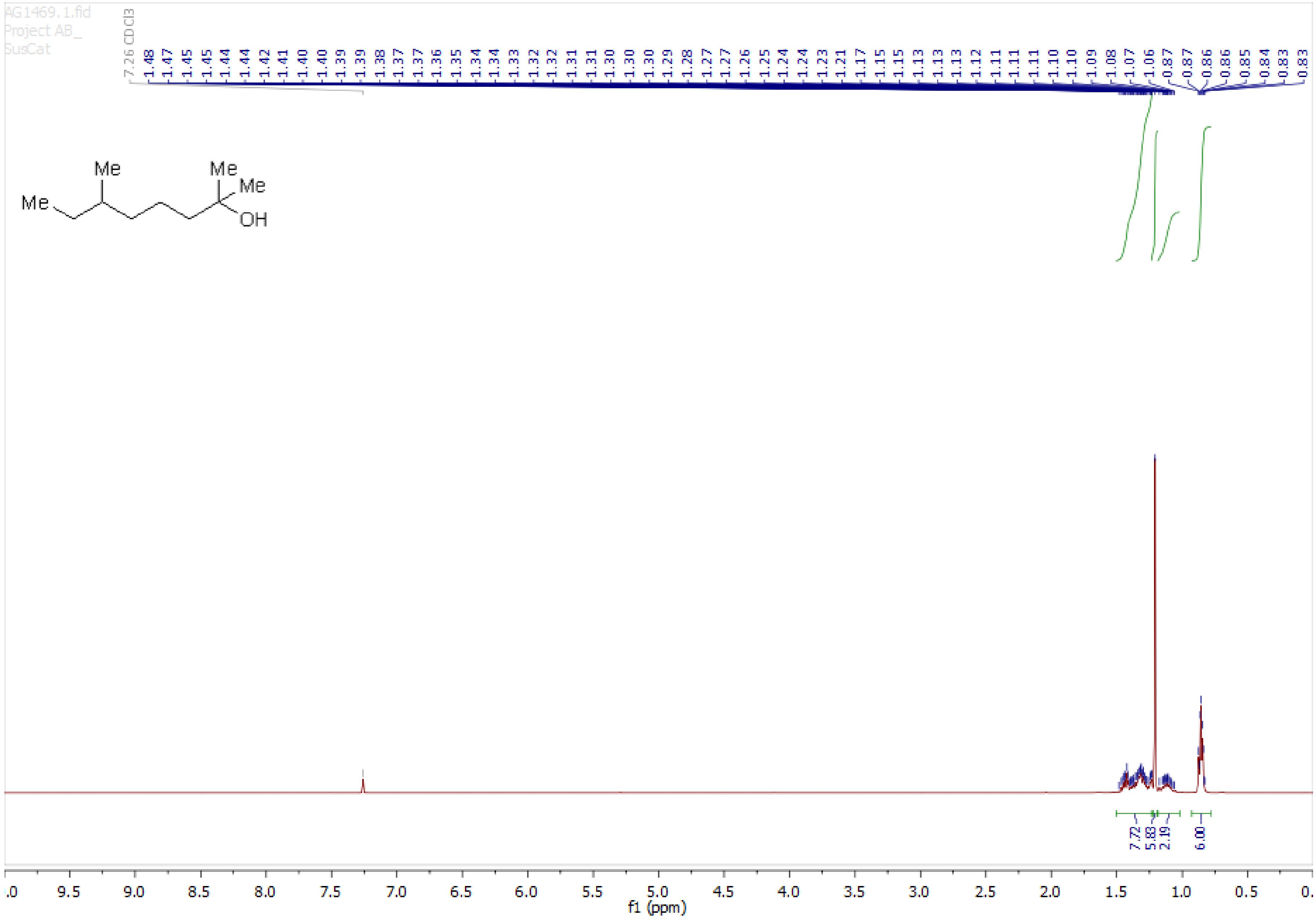

Spectrum S4. The ^{13}C NMR spectrum of *2,6-Dimethyloctan-2-ol (tetrahydromyrcenol)* in $CDCl_3$.

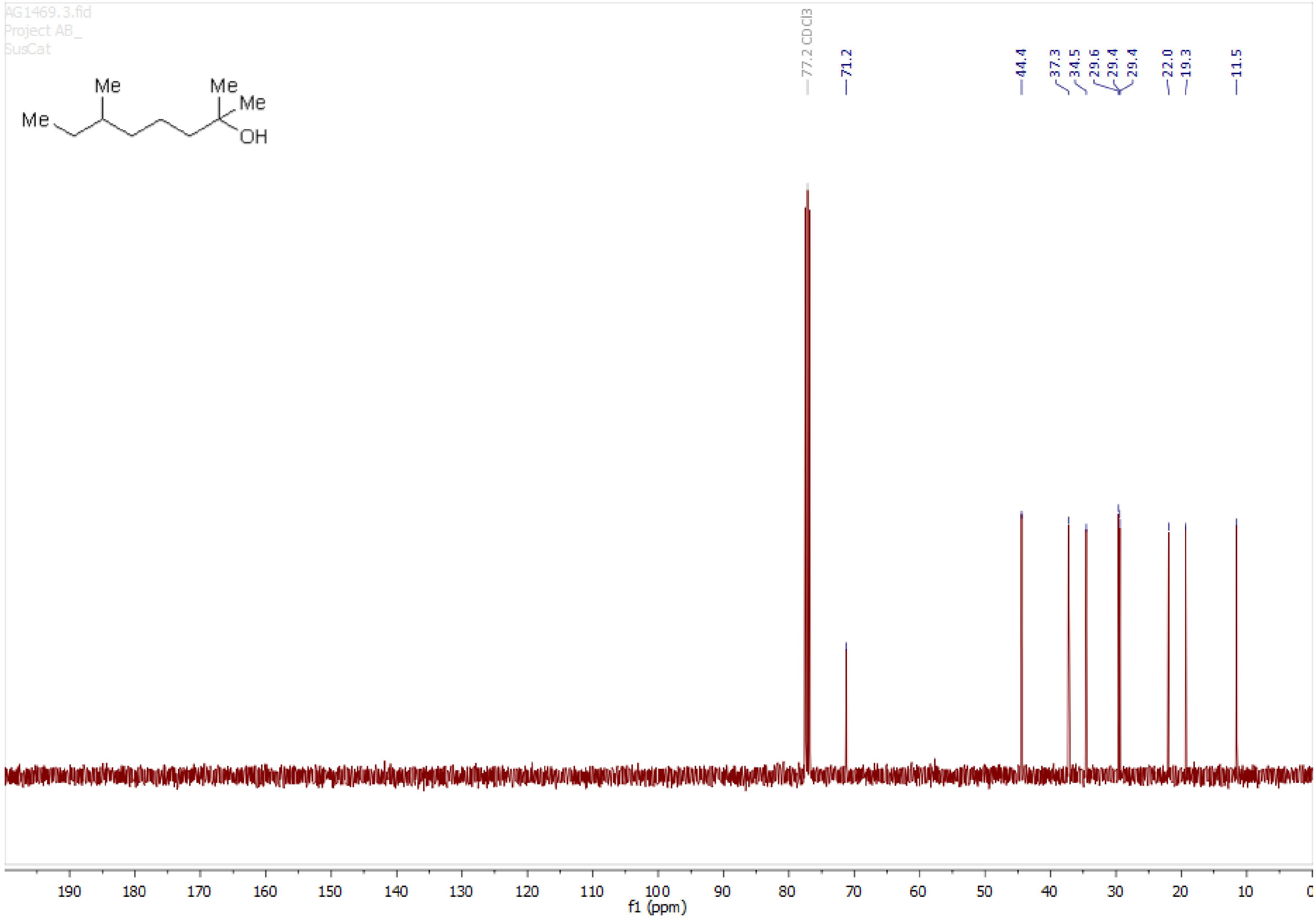

Spectrum S5. The ^{1}H NMR spectrum of *3-phenylpropan-1-ol* in $CDCl_3$.

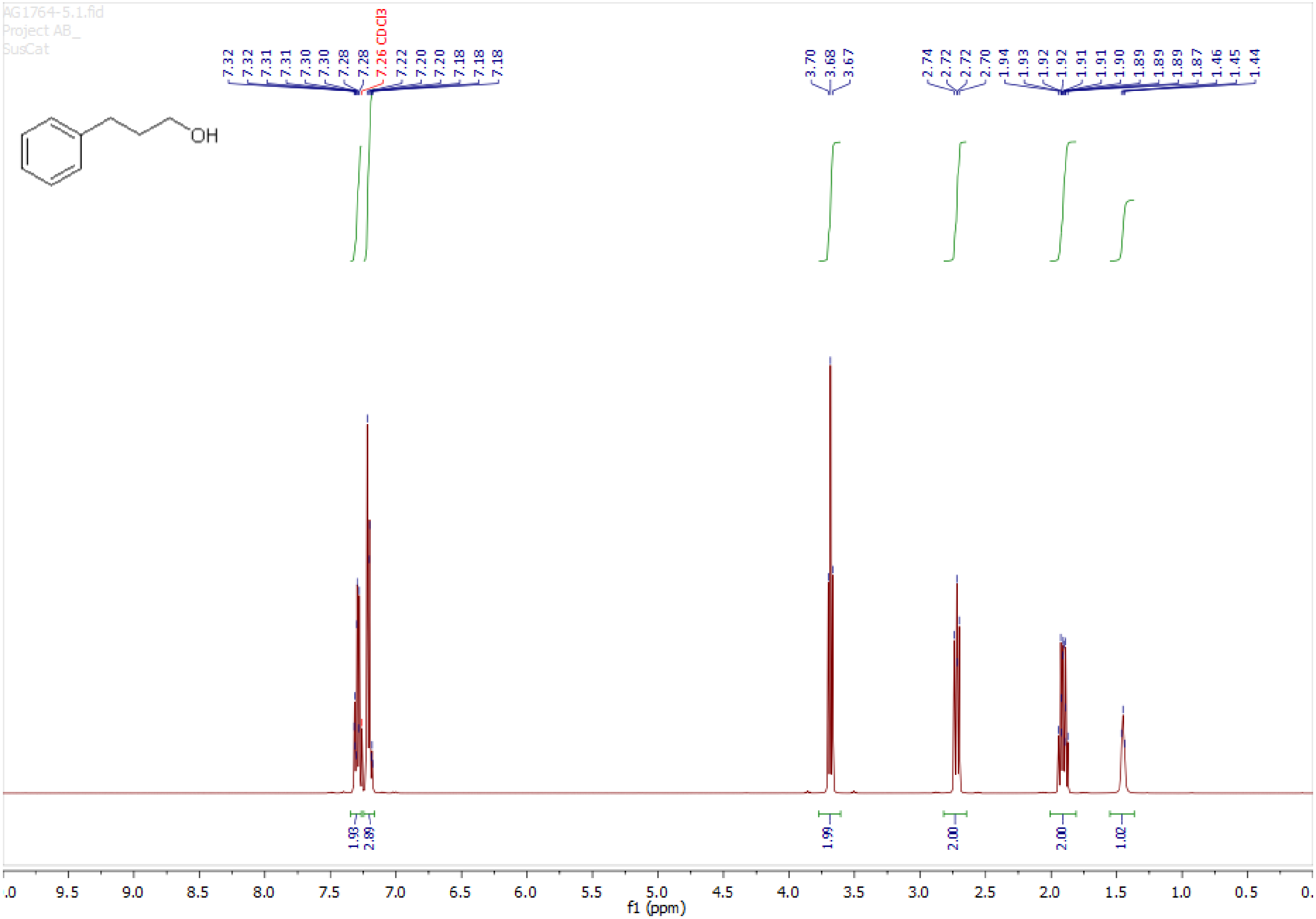

Spectrum S6. The ^{13}C NMR spectrum of *3-phenylpropan-1-ol* in $CDCl_3$.

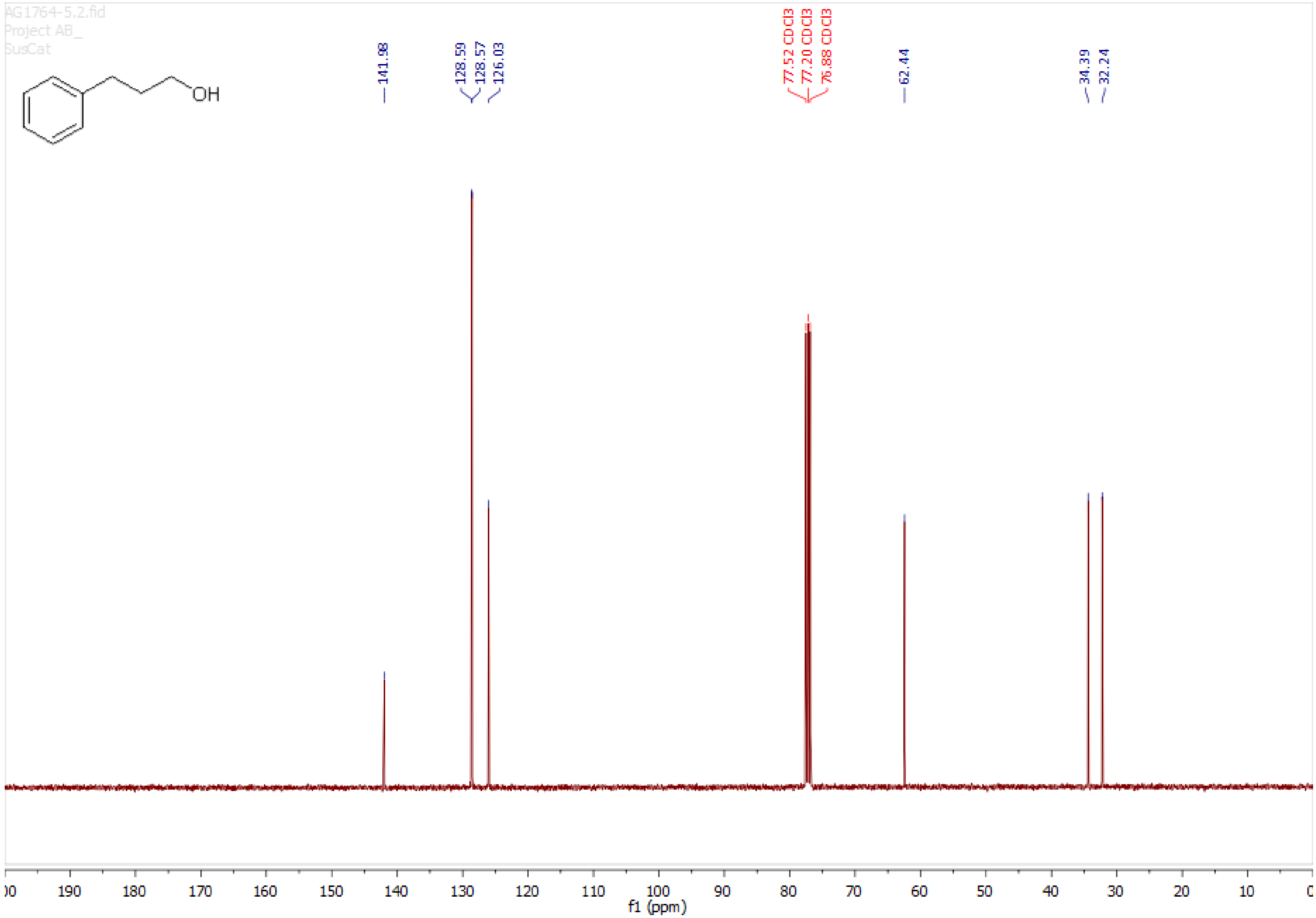

Spectrum S7. The ^{1}H NMR spectrum of *(3-iodopropyl)benzene* in $CDCl_3$.

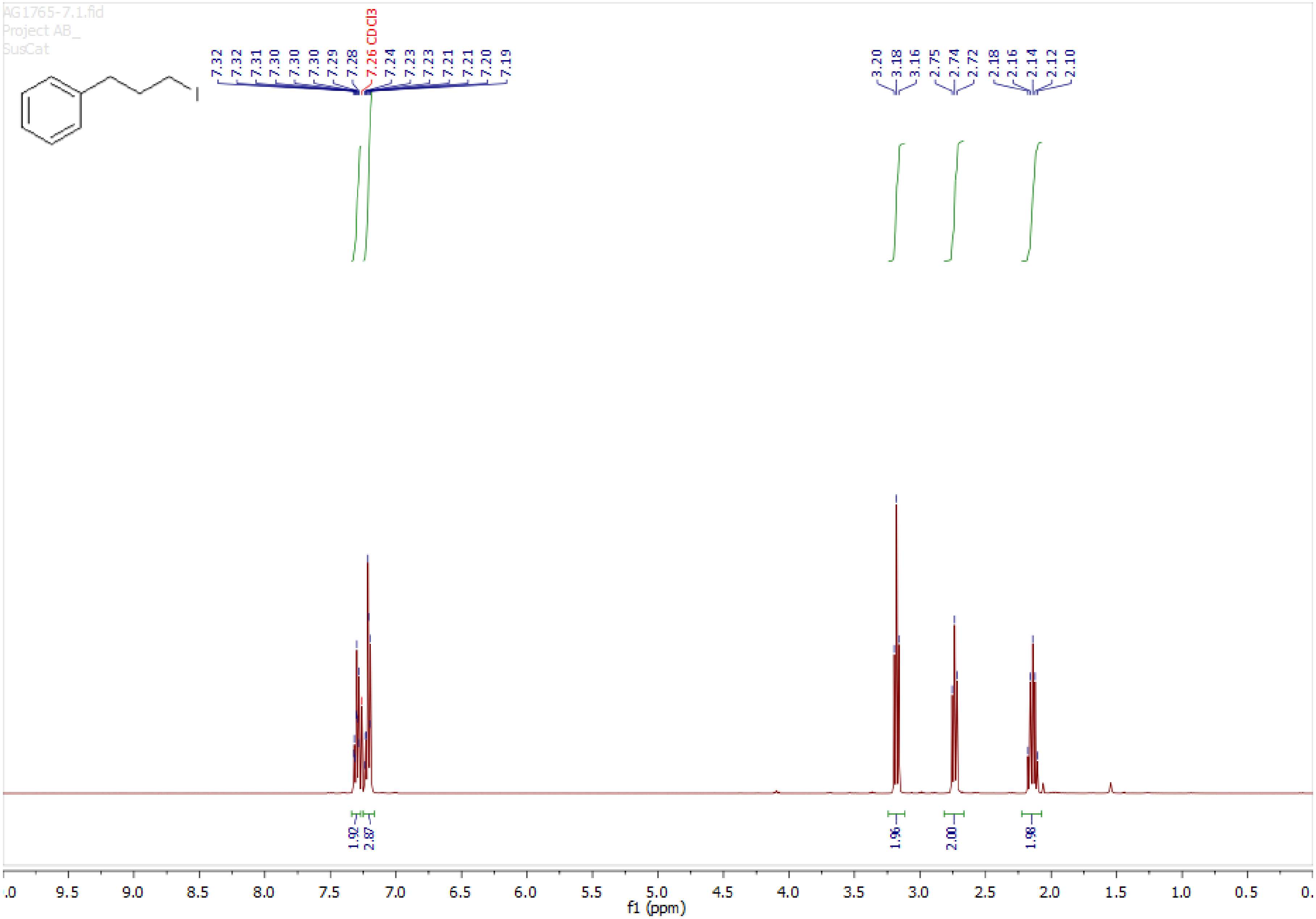

Spectrum S8. The ^{13}C NMR spectrum of *(3-iodopropyl)benzene* in $CDCl_3$.

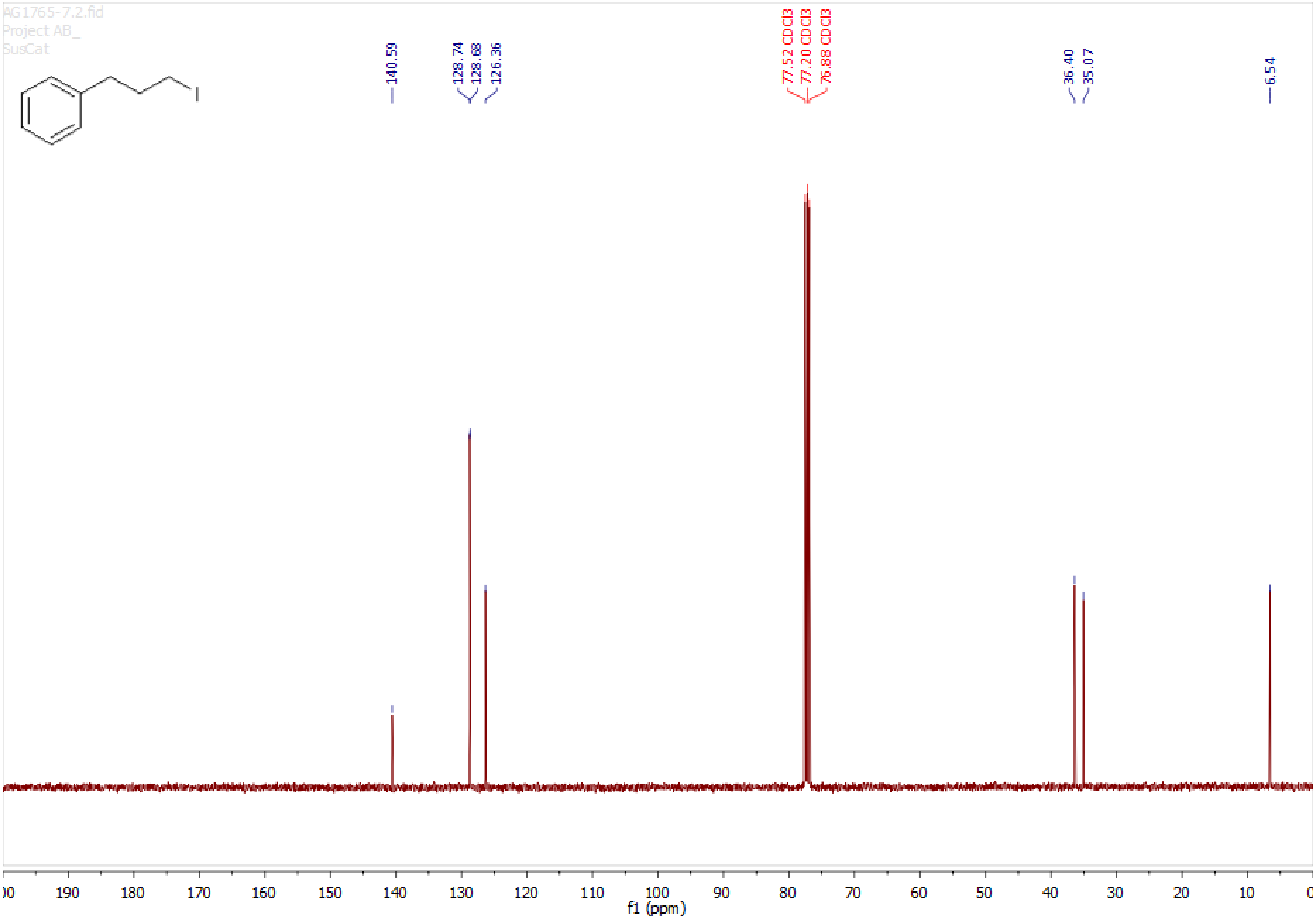

Spectrum S9. The ^{1}H NMR spectrum of *2-(4-methylcyclohexyl)propan-2-yl acetate* in $CDCl_3$.

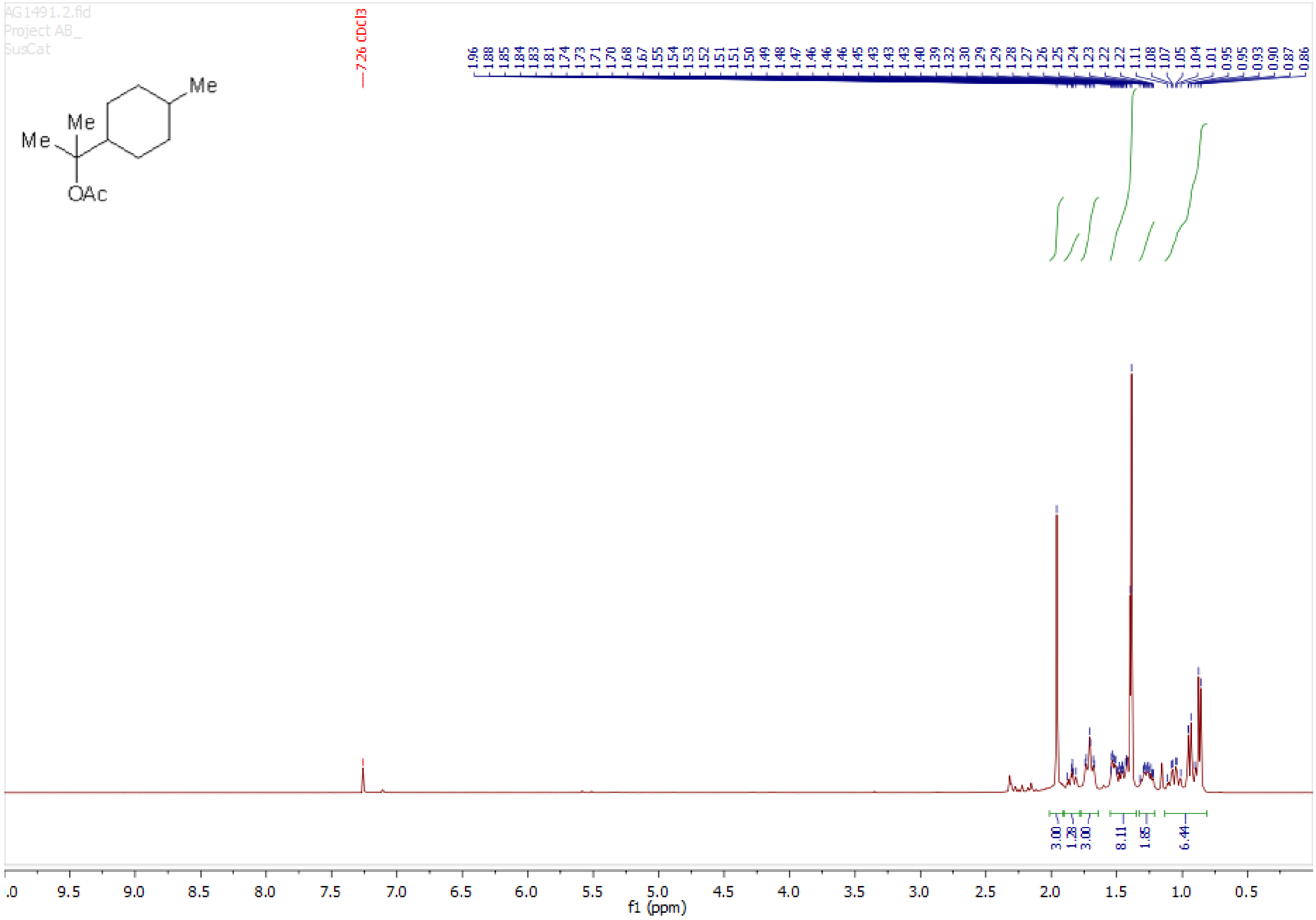

Spectrum S10. The ^{13}C NMR spectrum of *2-(4-methylcyclohexyl)propan-2-yl acetate* in $CDCl_3$.

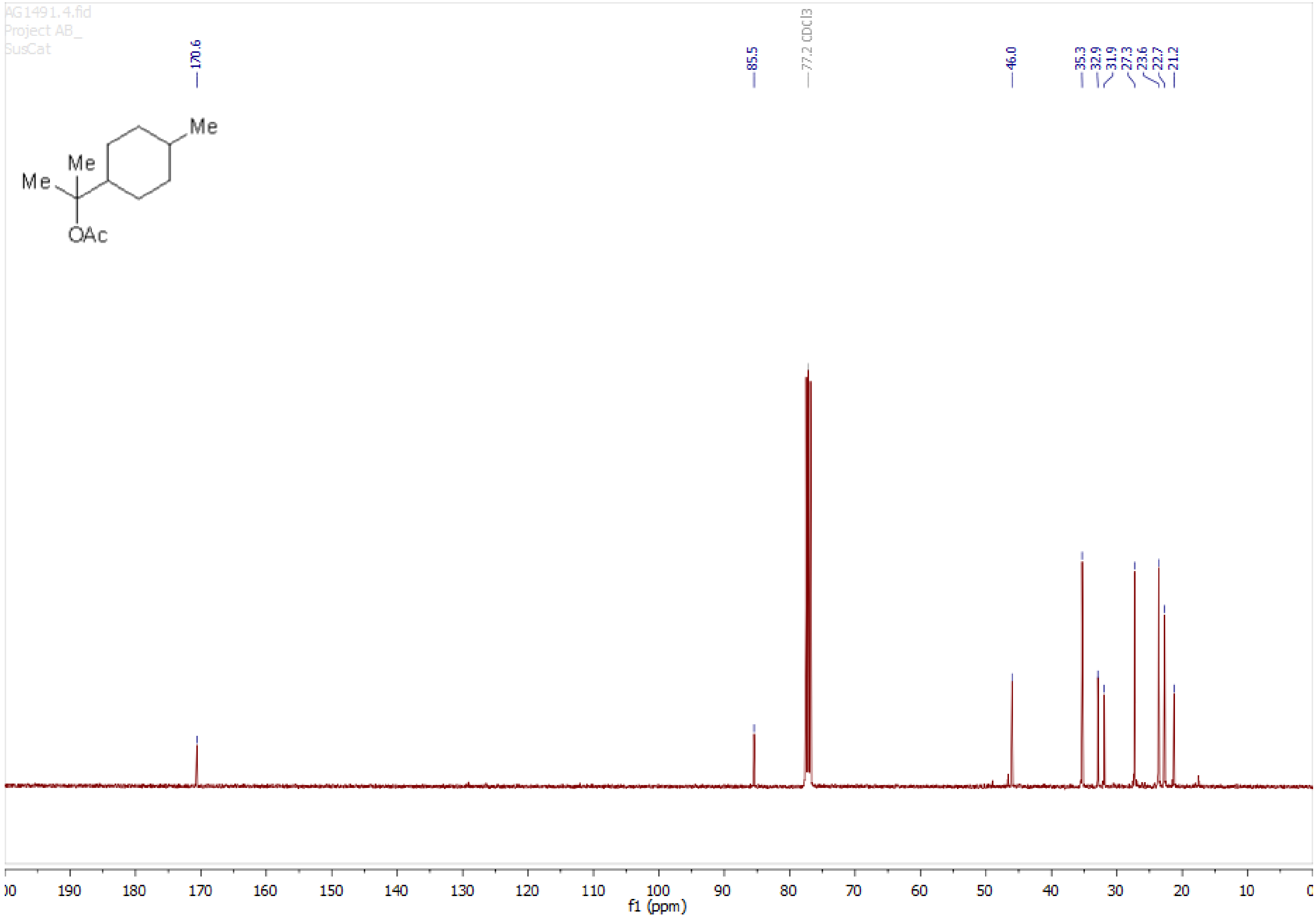

Spectrum S11. The ^{1}H NMR spectrum of *2,6-dimethyloctan-2-yl acetate* in $CDCl_3$.

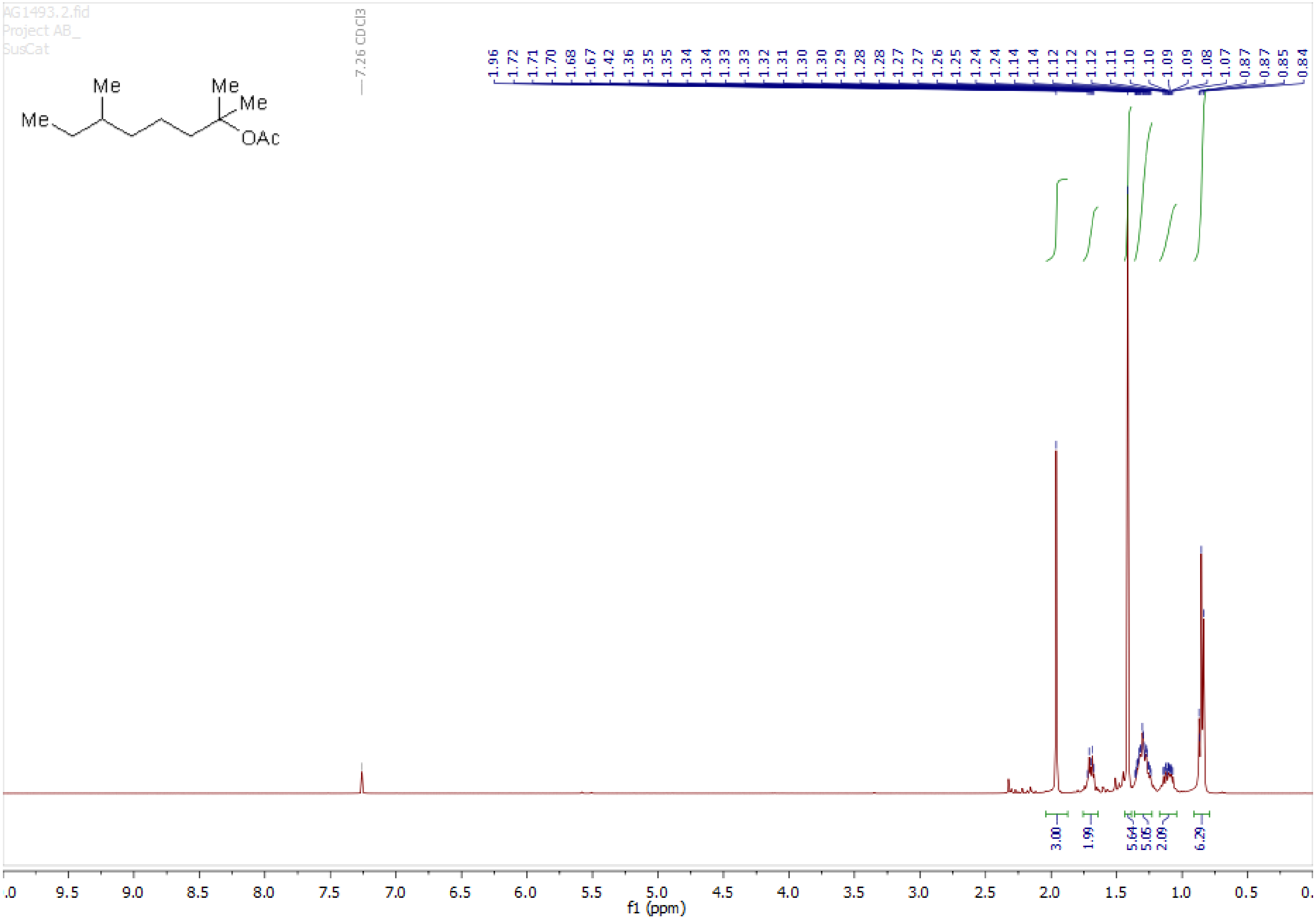

Spectrum S12. The ^{13}C NMR spectrum of *2,6-dimethyloctan-2-yl acetate* in $CDCl_3$.

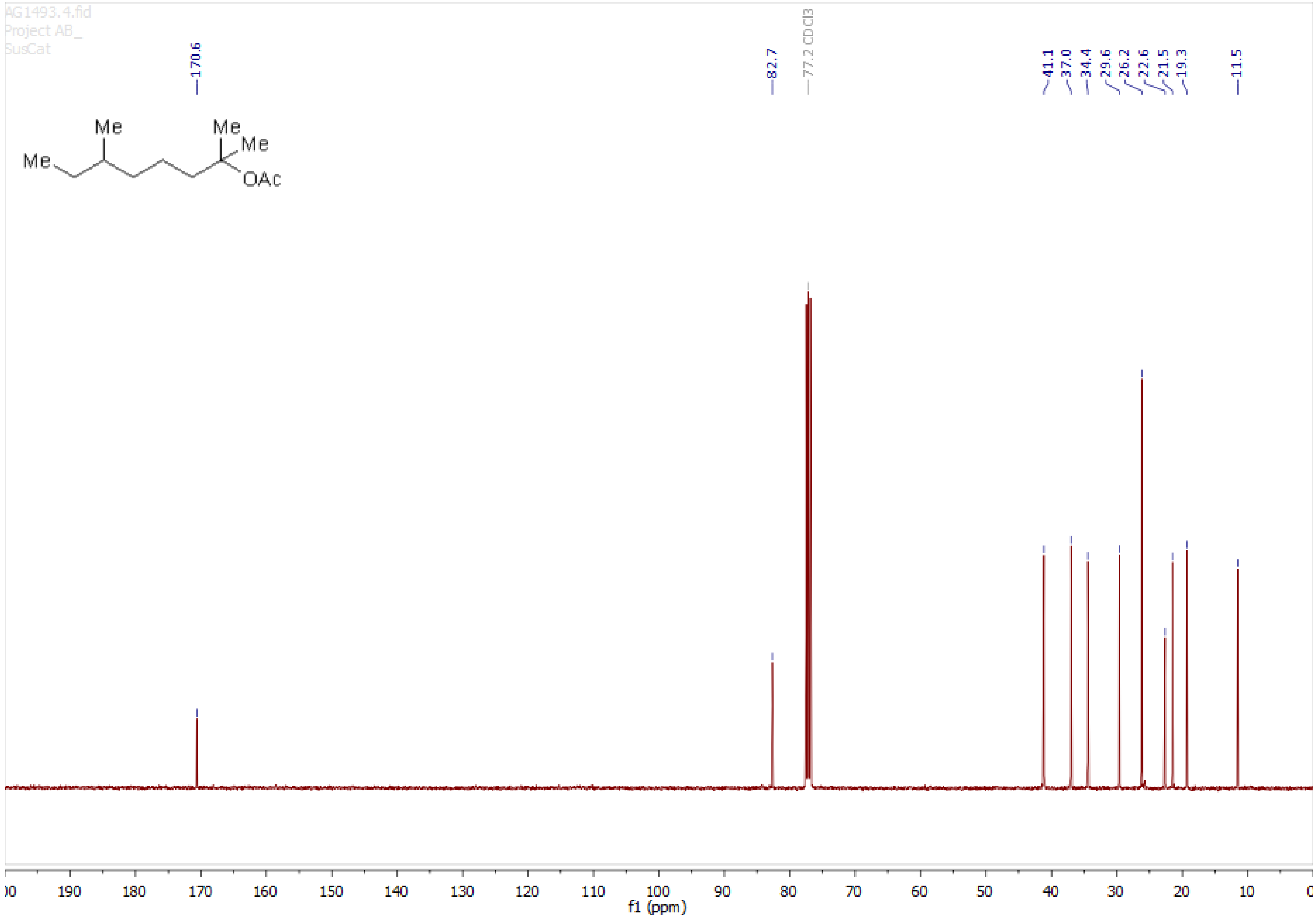

Spectrum S13. The ^1H NMR spectrum of *GreenPhos 1* in $CDCl_3$.

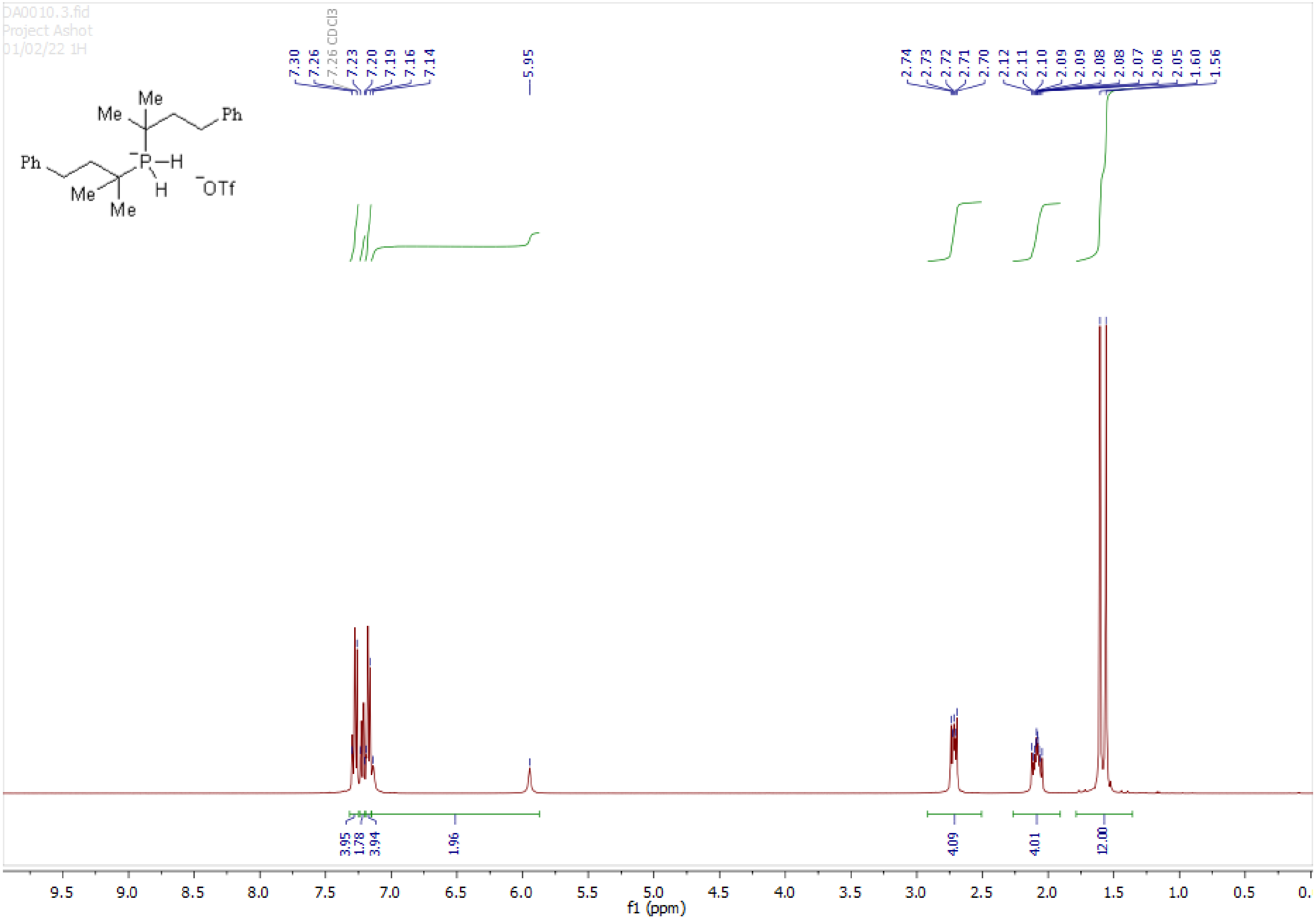

Spectrum S14. The ^{13}C NMR spectrum of *GreenPhos 1* in $CDCl_3$.

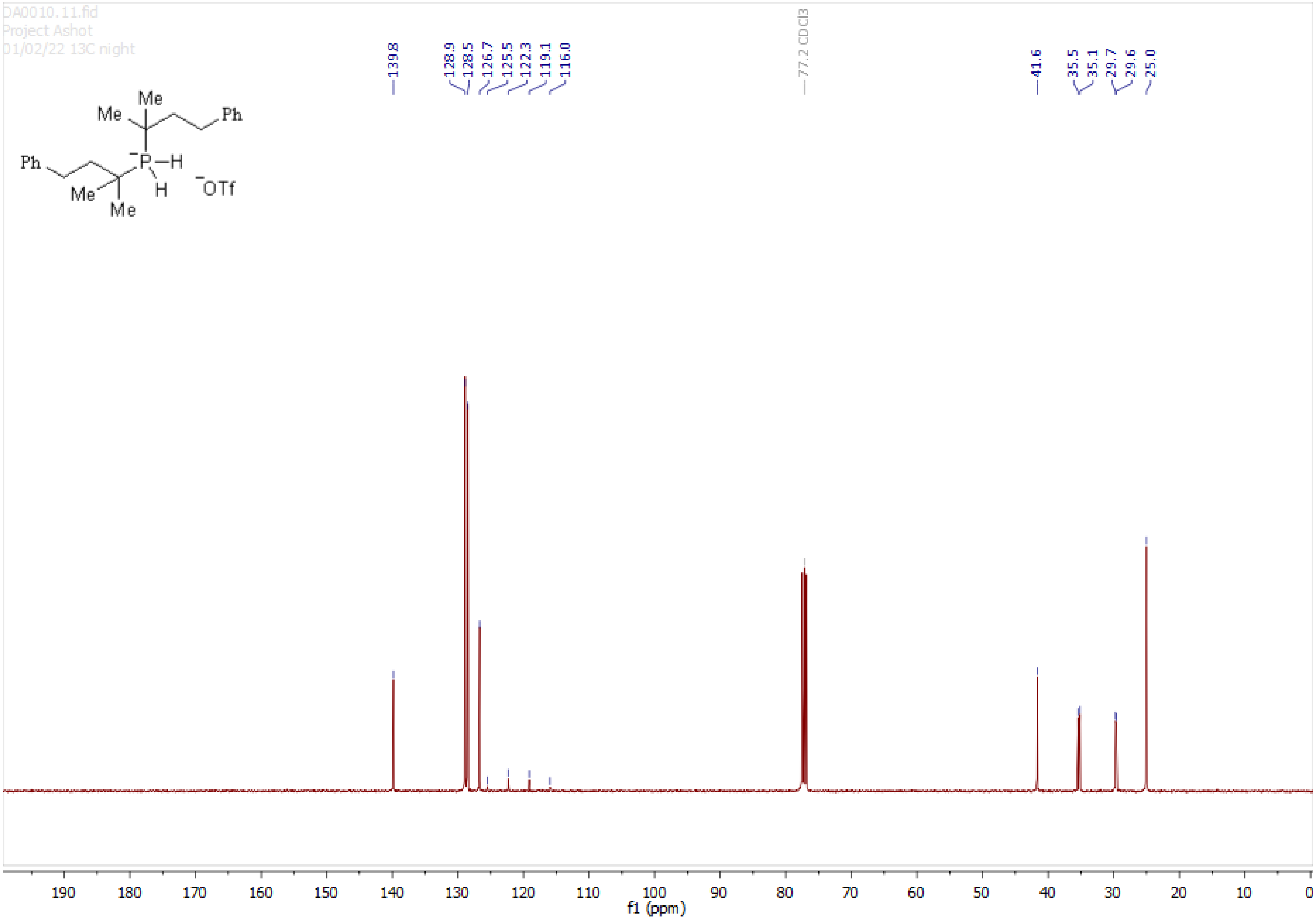

Spectrum S15. The ^{31}P NMR spectrum of *GreenPhos 1* with composite pulse decoupling in $CDCl_3$.

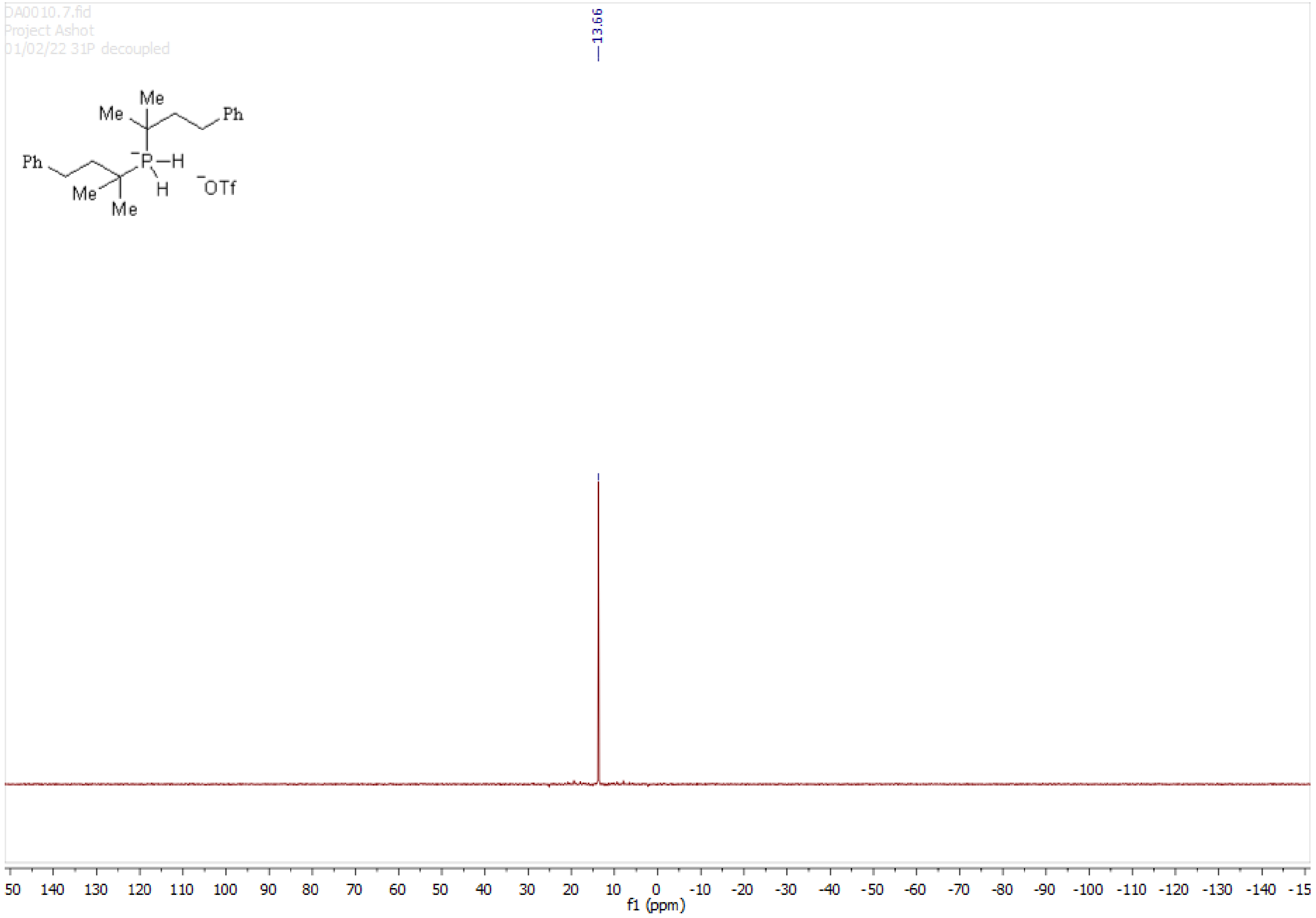

Spectrum S16. The ^{31}P NMR spectrum of *GreenPhos 1* with no decoupling in $CDCl_3$.

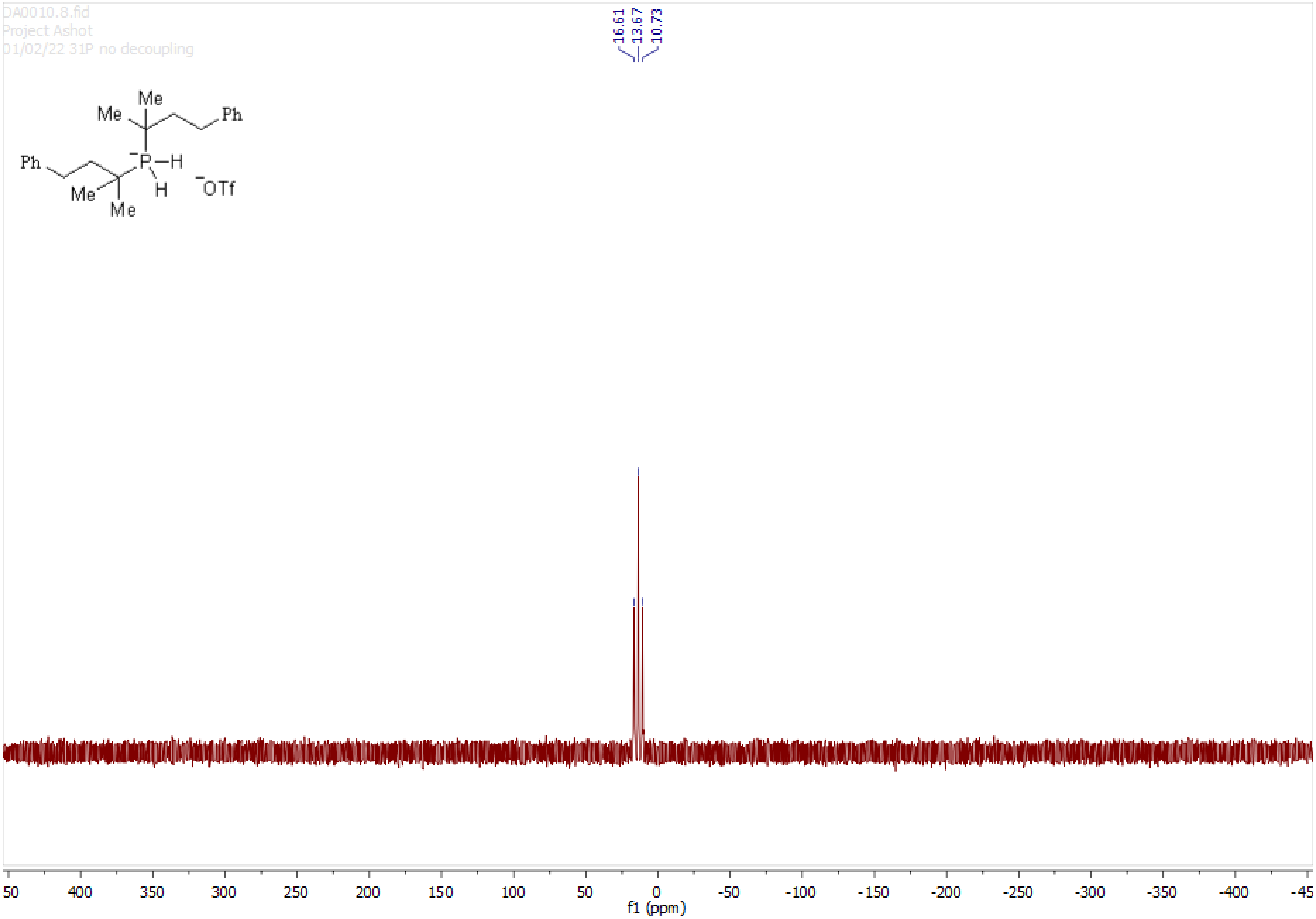

Spectrum S17. The ^{19}F NMR spectrum of *GreenPhos 1* in $CDCl_3$.

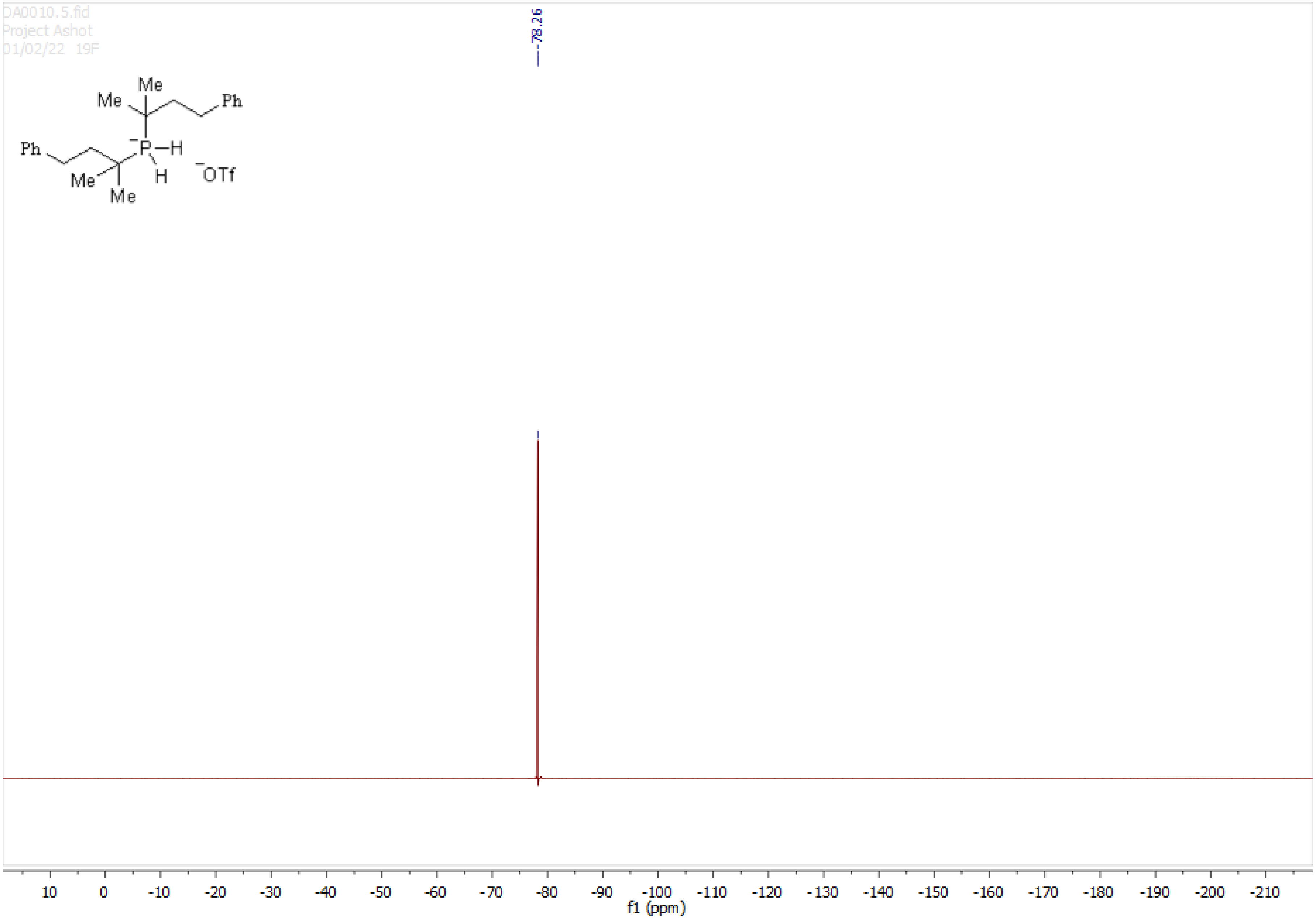

Spectrum S18. The ¹H NMR spectrum of *GreenPhos 2* in $CDCl_3$.

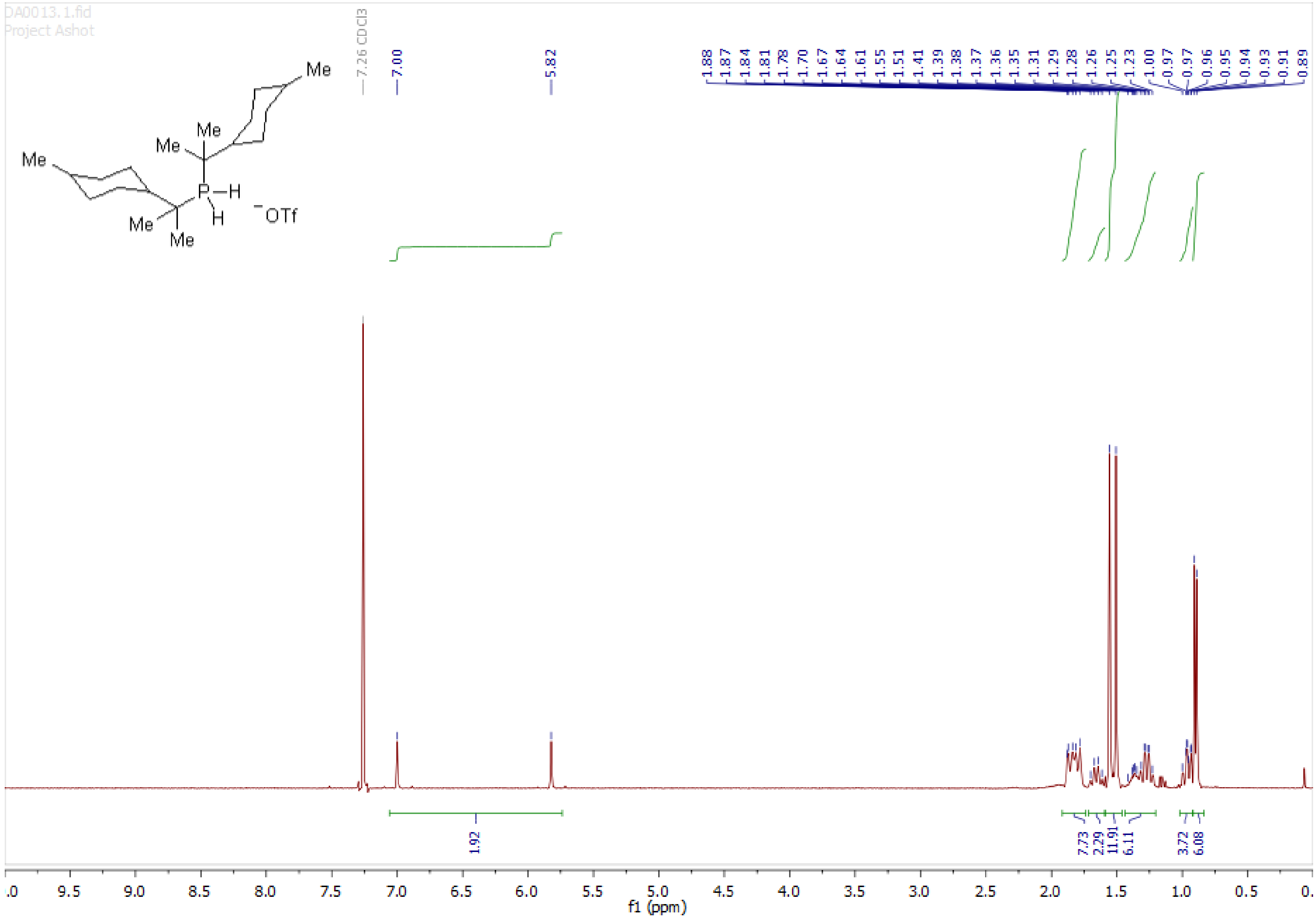

Spectrum S19. The ^{13}C NMR spectrum of *GreenPhos 2* in $CDCl_3$.

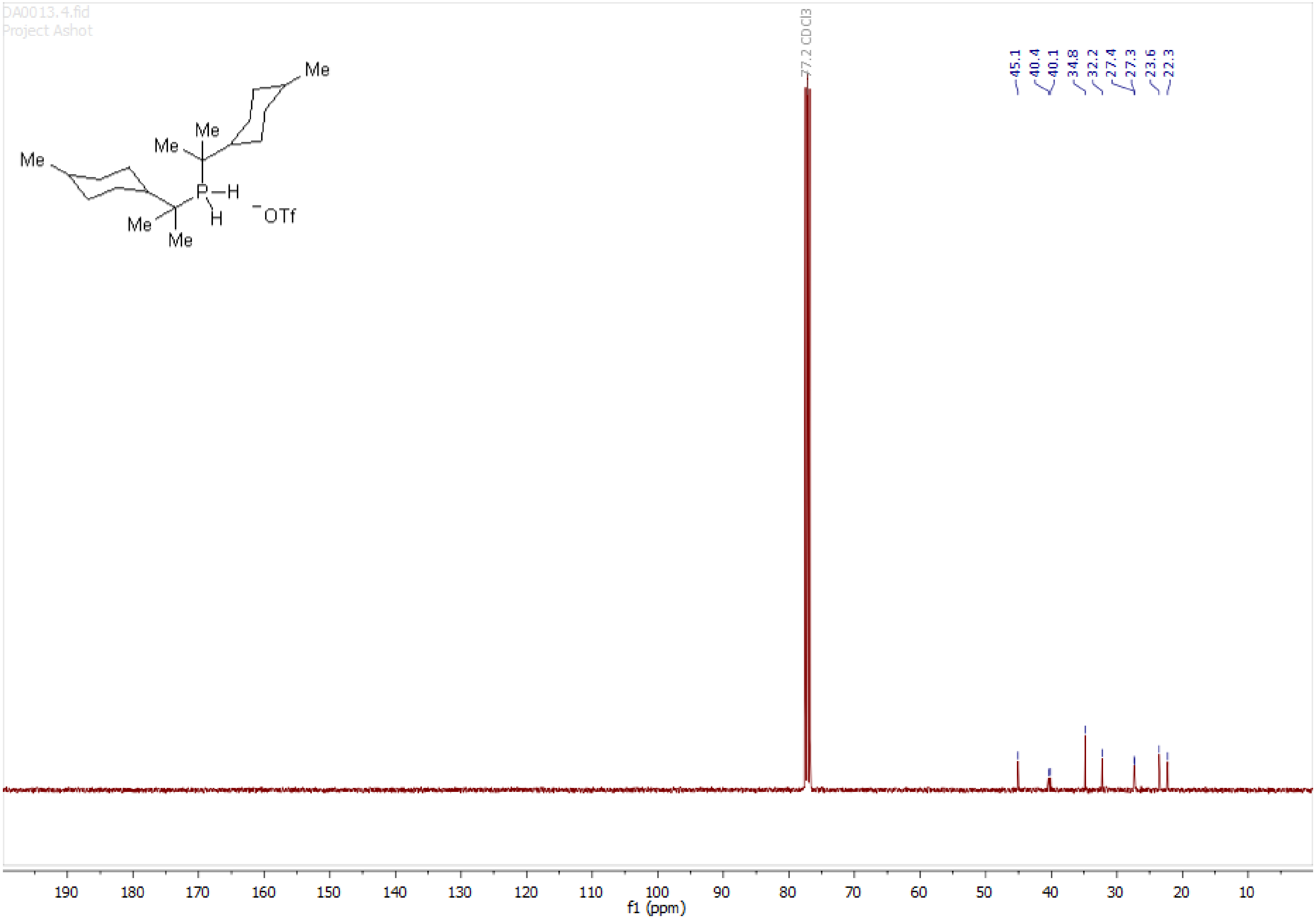

Spectrum S20. The ^{31}P NMR spectrum of *GreenPhos 2* with composite pulse decoupling in $CDCl_3$.

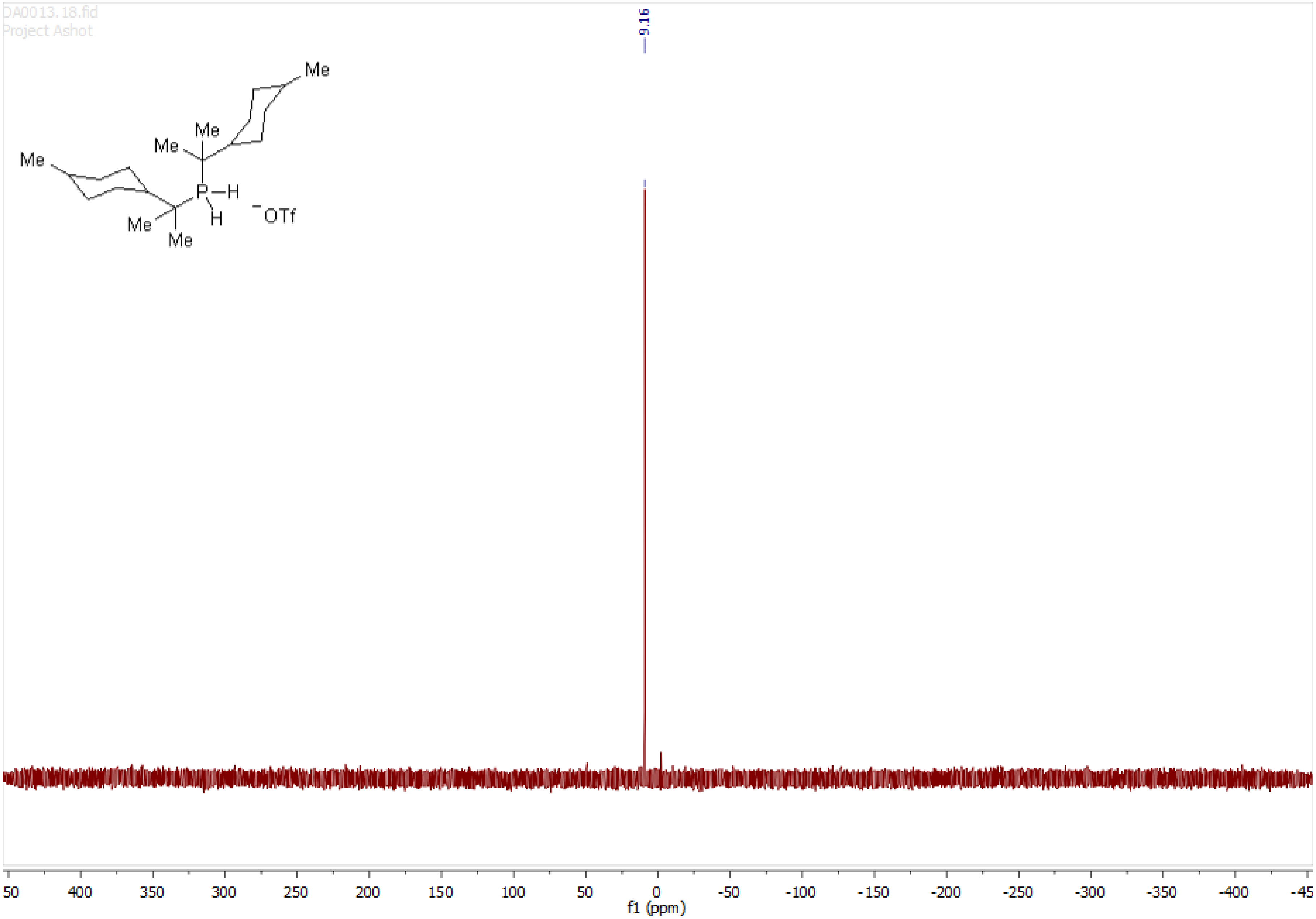

Spectrum S21. The ^{31}P NMR spectrum of *GreenPhos 2* with no decoupling in $CDCl_3$.

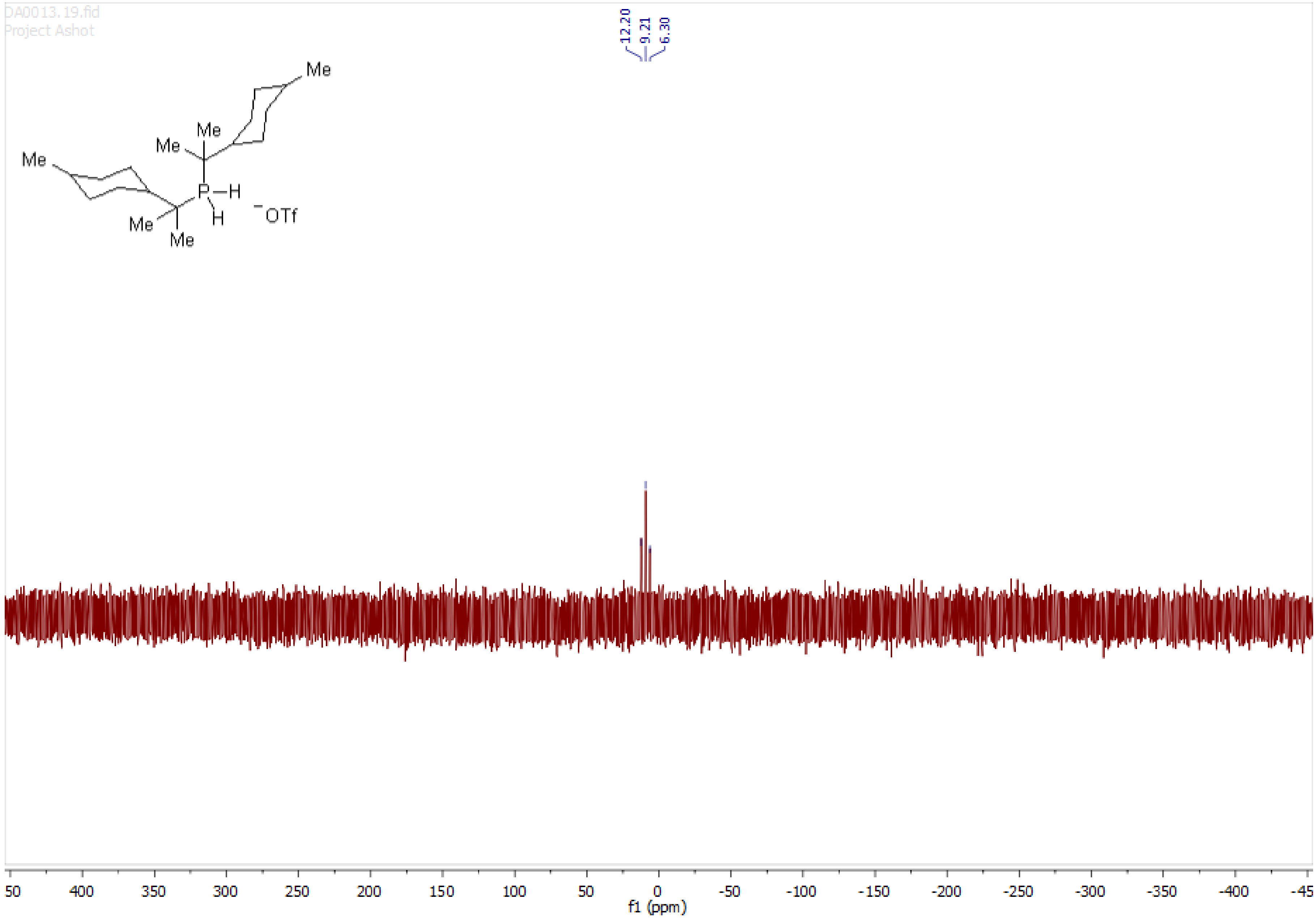

Spectrum S22. The ^{19}F NMR spectrum of *GreenPhos 2* in $CDCl_3$.

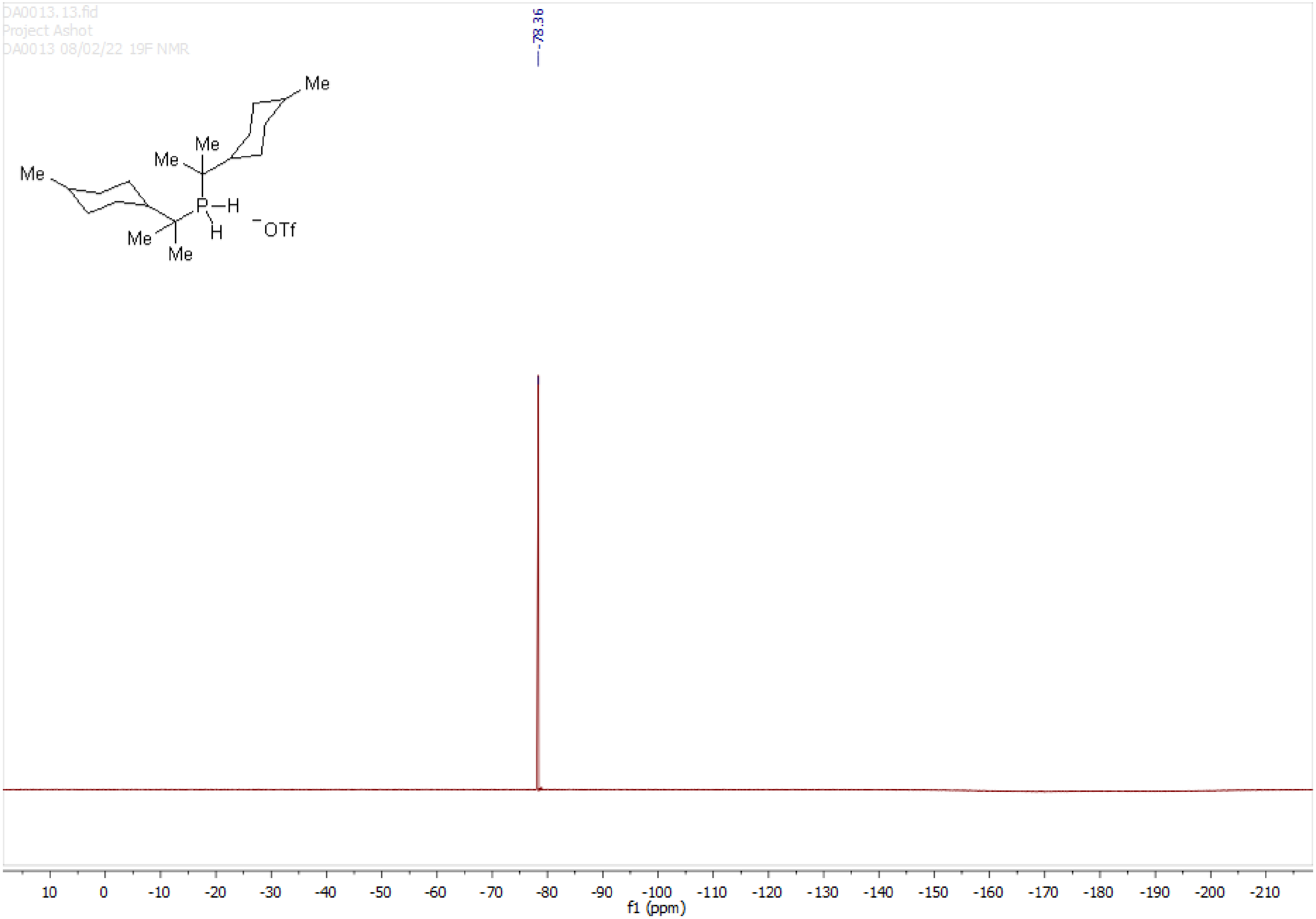

Spectrum S23. The ^{1}H NMR spectrum of *GreenPhos 3* in $CDCl_3$.

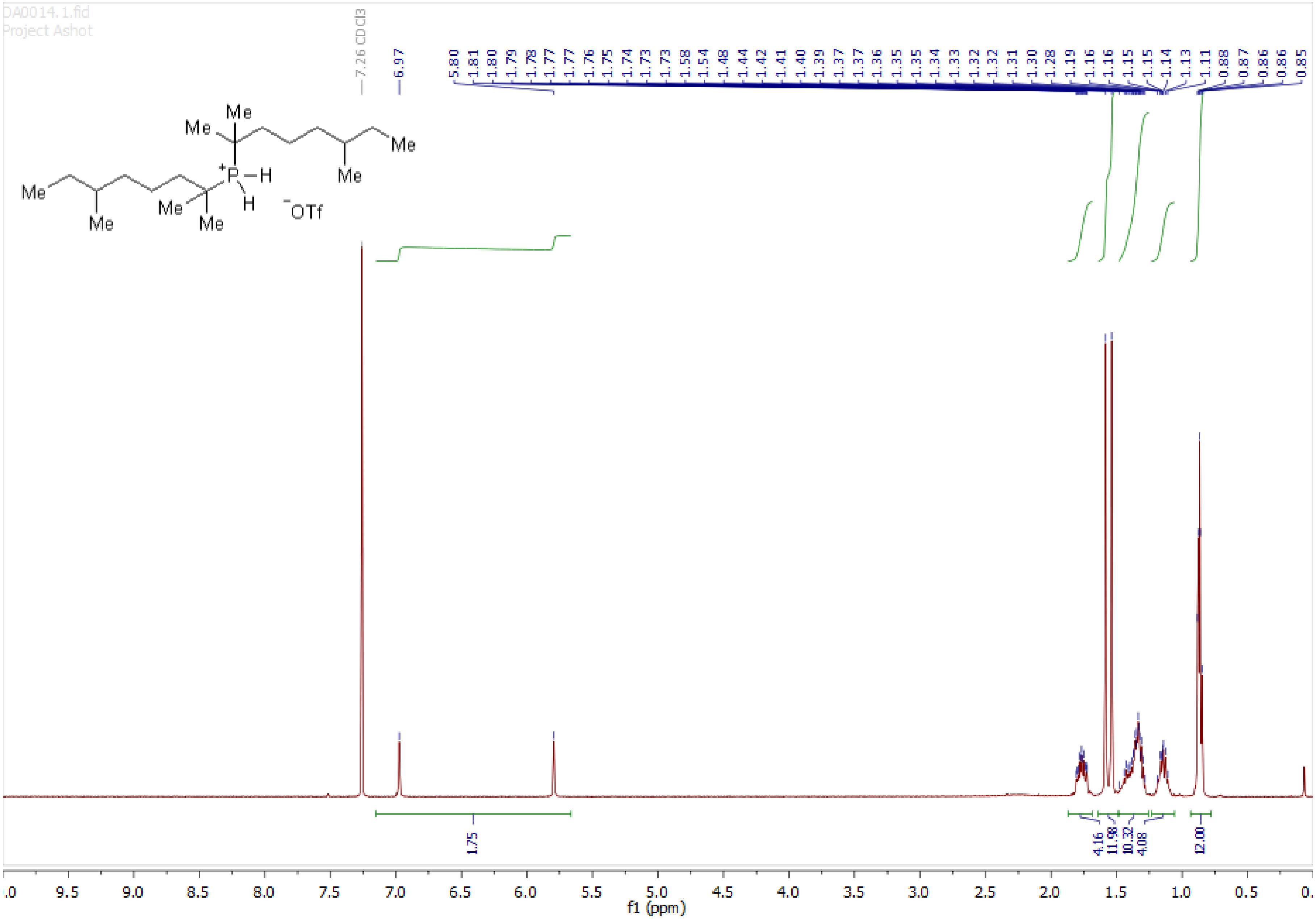

Spectrum S24. The ^{13}C NMR spectrum of *GreenPhos 3* in $CDCl_3$.

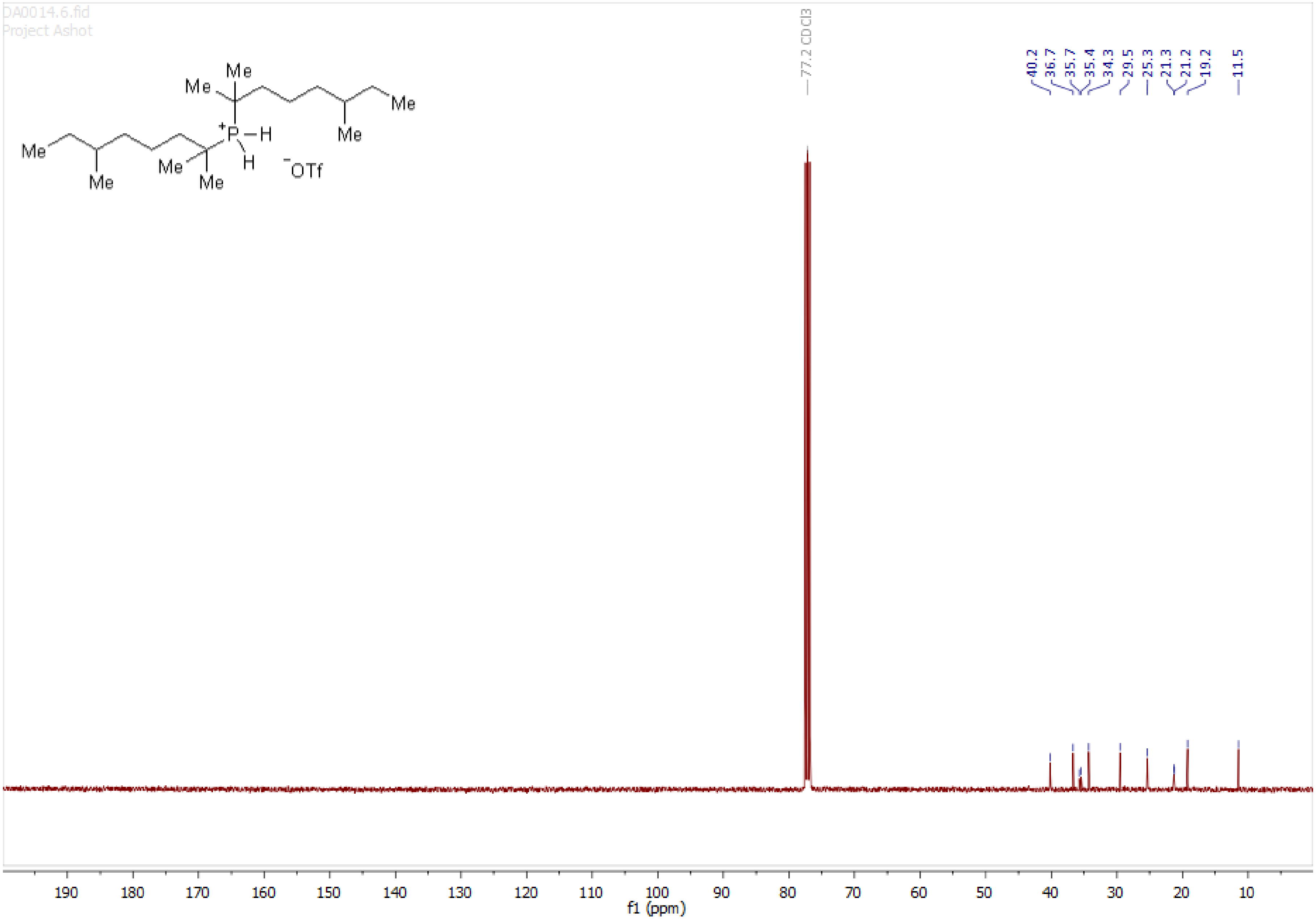

Spectrum S25. The ^{31}P NMR spectrum of *GreenPhos 3* with composite pulse decoupling in $CDCl_3$.

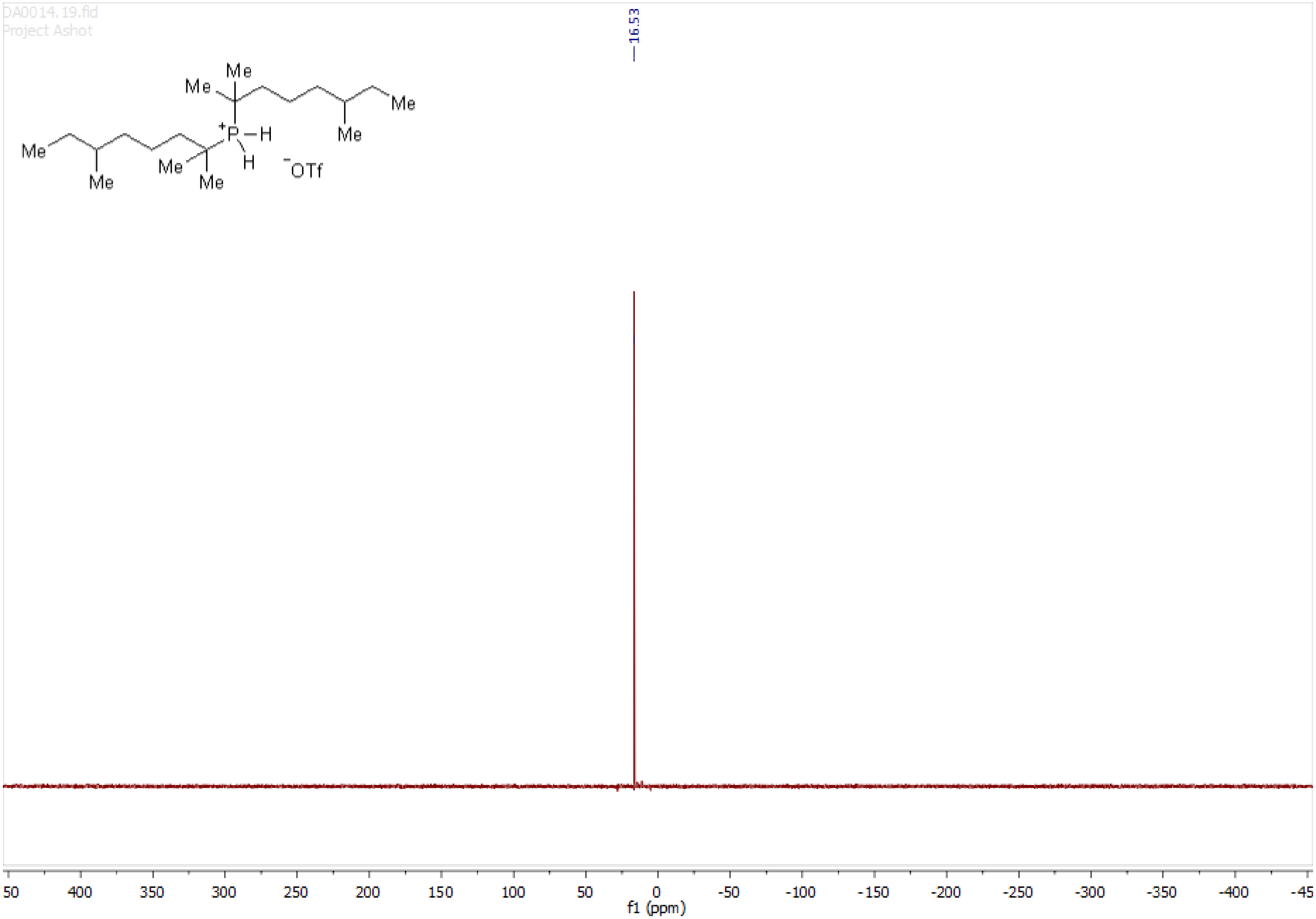

Spectrum S26. The ^{31}P NMR spectrum of *GreenPhos 3* with no decoupling in $CDCl_3$.

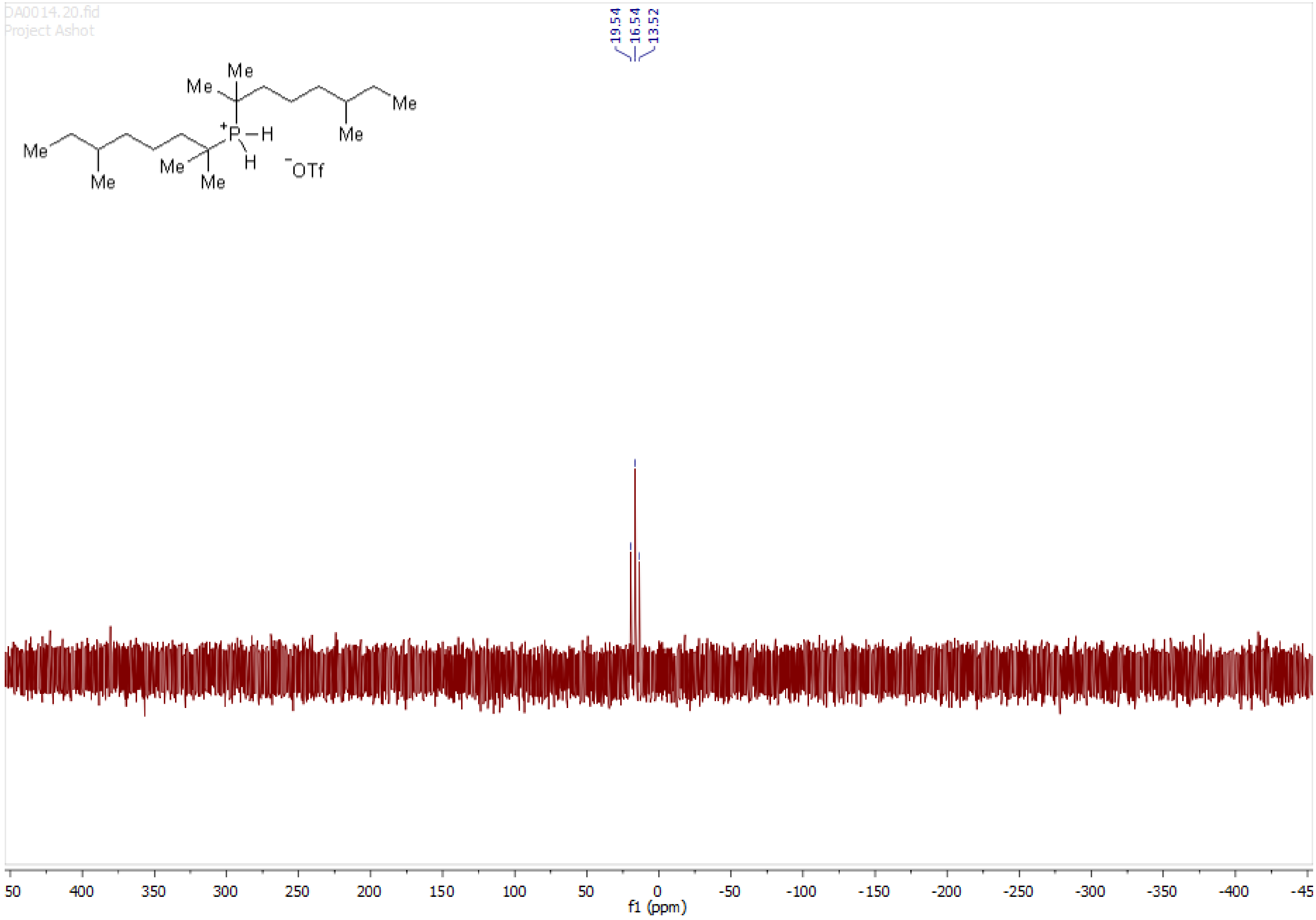

Spectrum S27. The ^{19}F NMR spectrum of *GreenPhos 3* in $CDCl_3$.

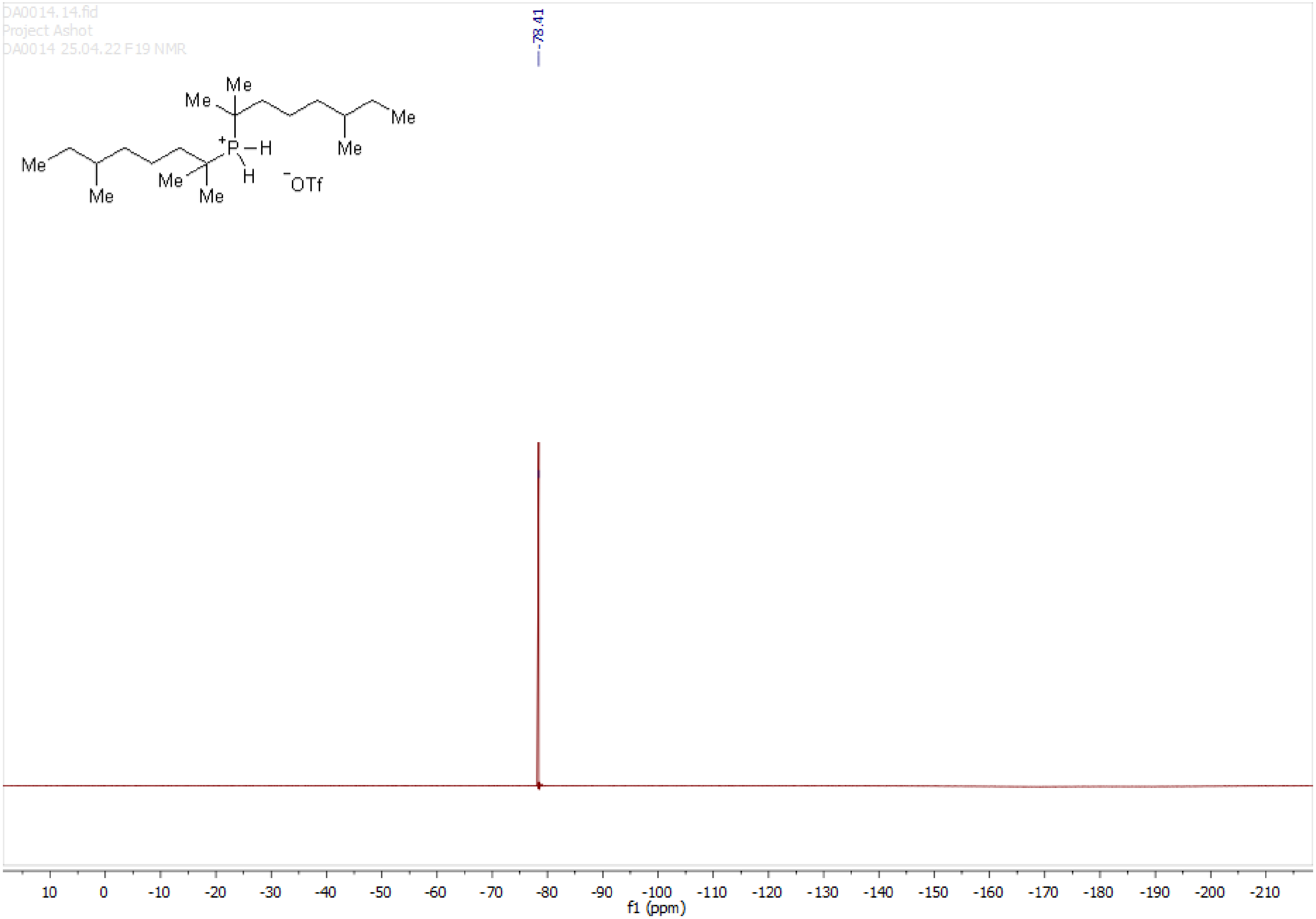

Spectrum S28. The ^{1}H NMR spectrum of *GreenPhos 4* in $CDCl_3$.

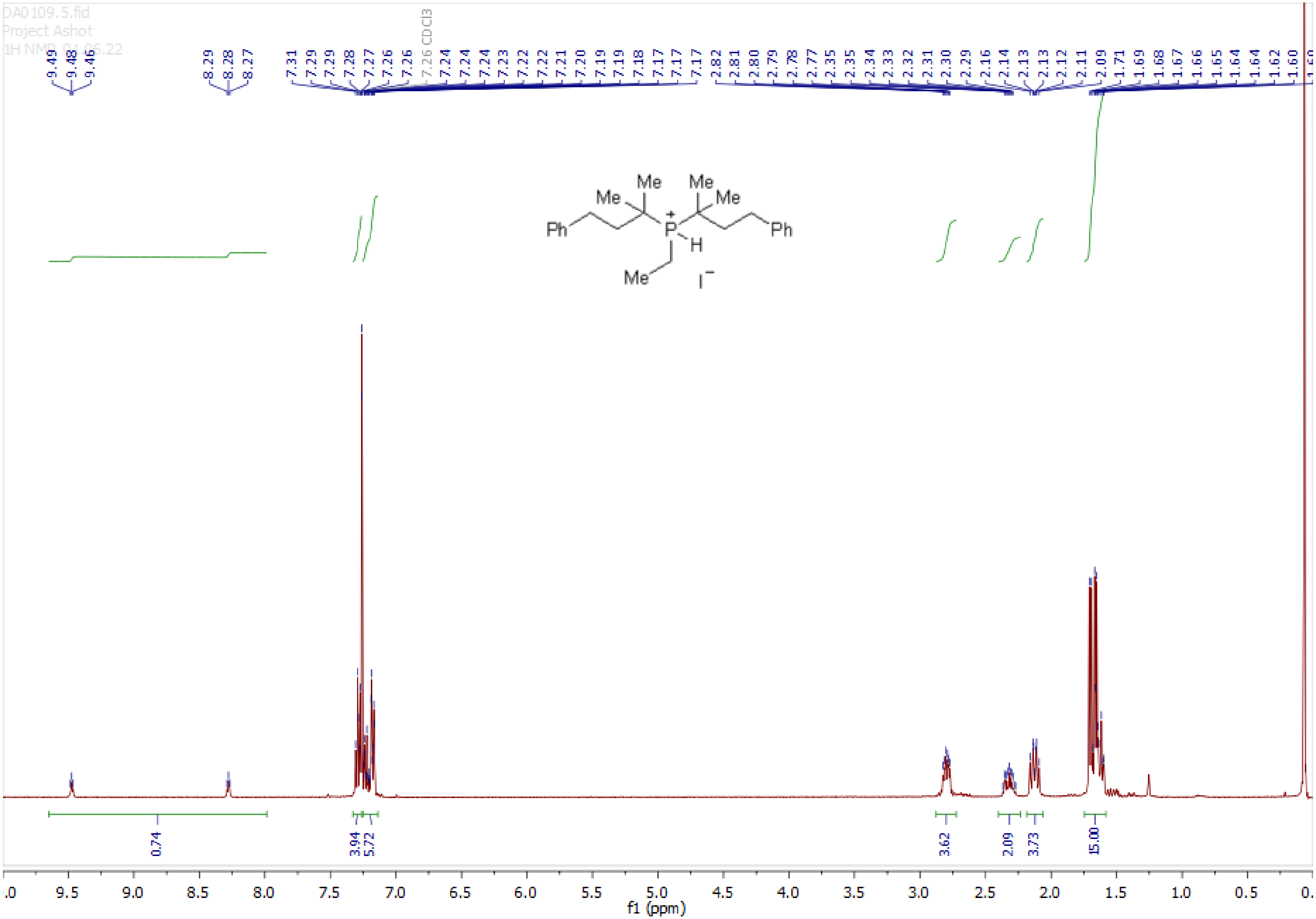

Spectrum S29. The ^{13}C NMR spectrum of *GreenPhos 4* in $CDCl_3$.

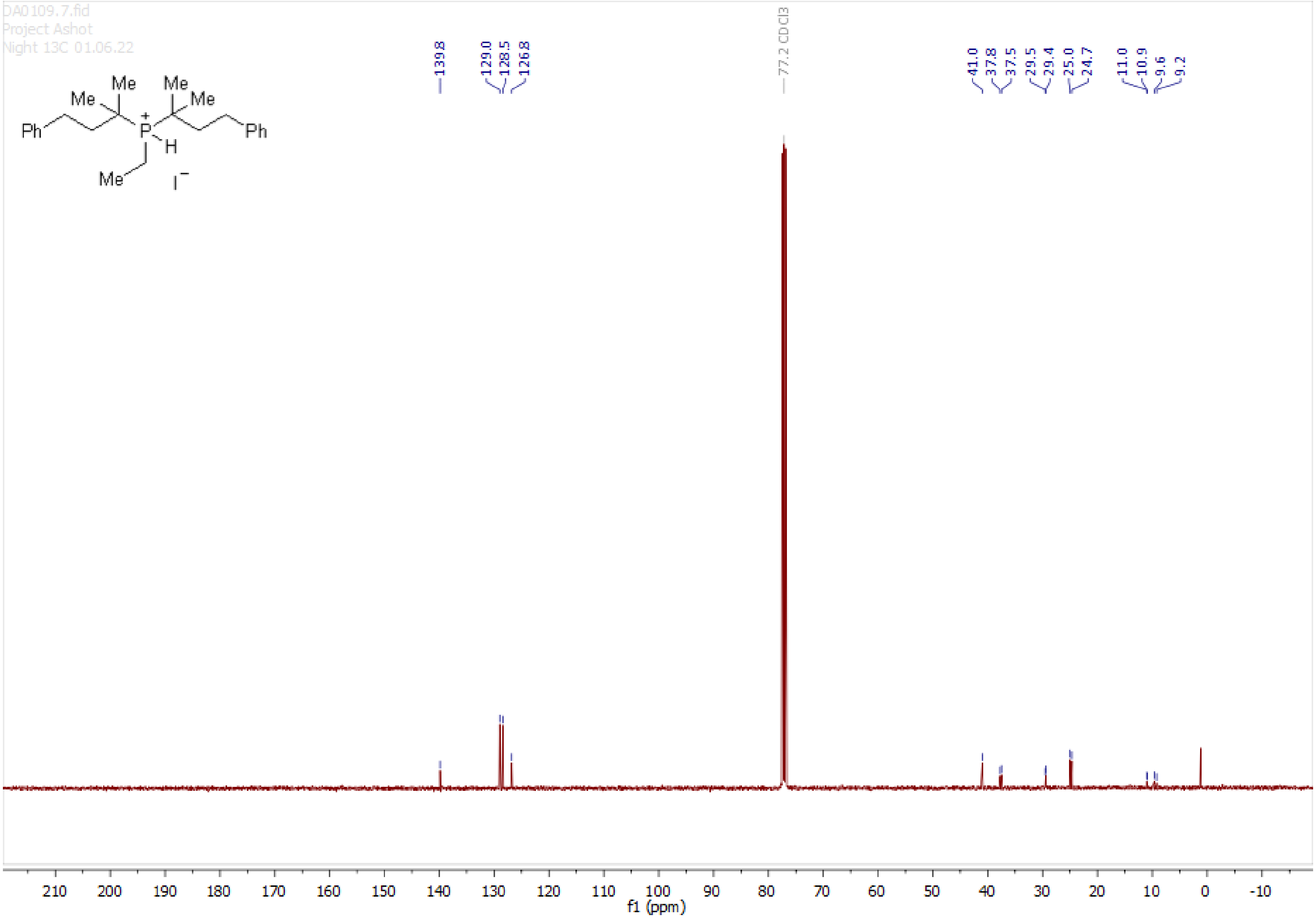

Spectrum S30. The ^{31}P NMR spectrum of *GreenPhos 4* with composite pulse decoupling in $CDCl_3$.

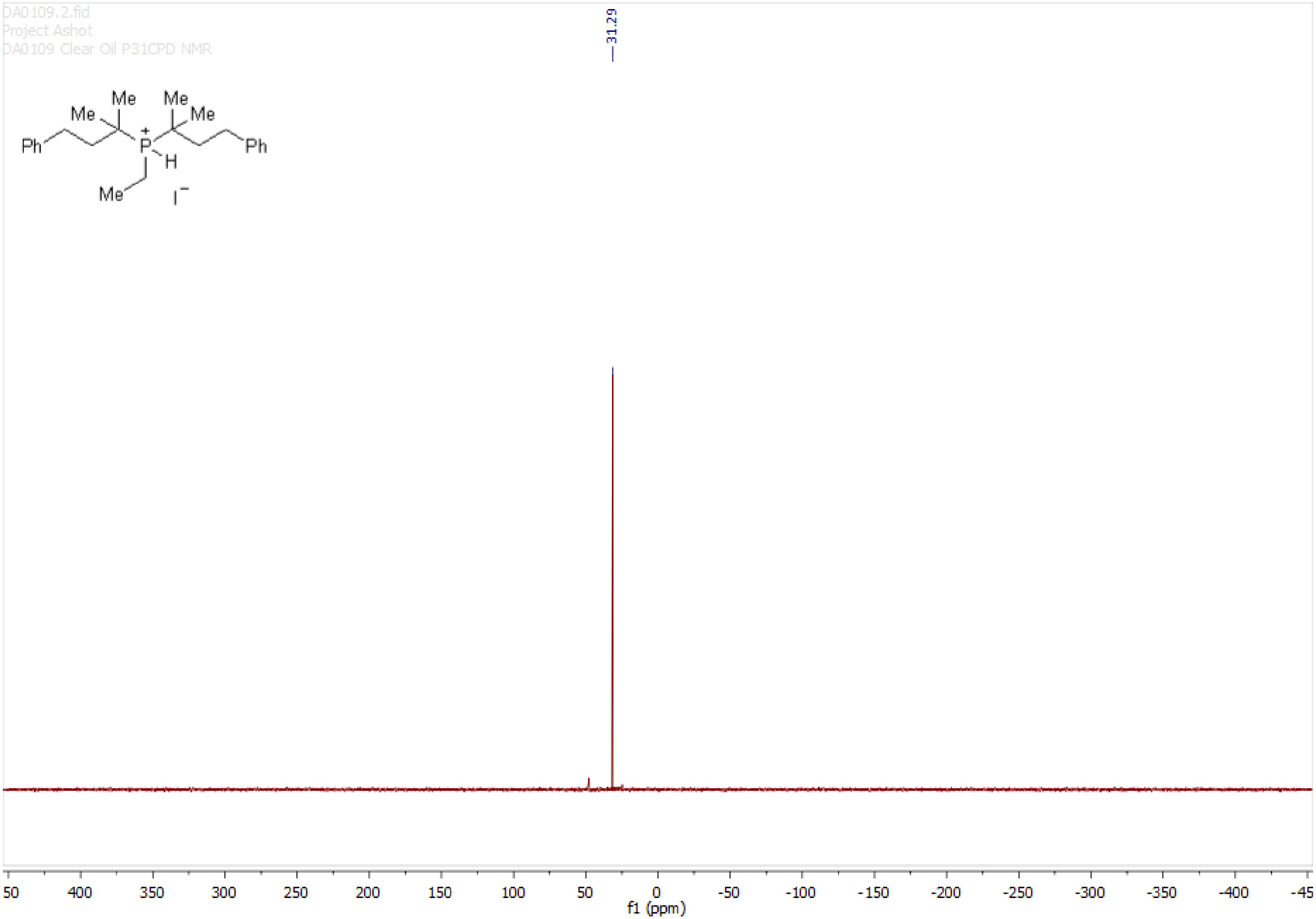

Spectrum S31. The ^{31}P NMR spectrum of *GreenPhos 4* with no decoupling in $CDCl_3$.

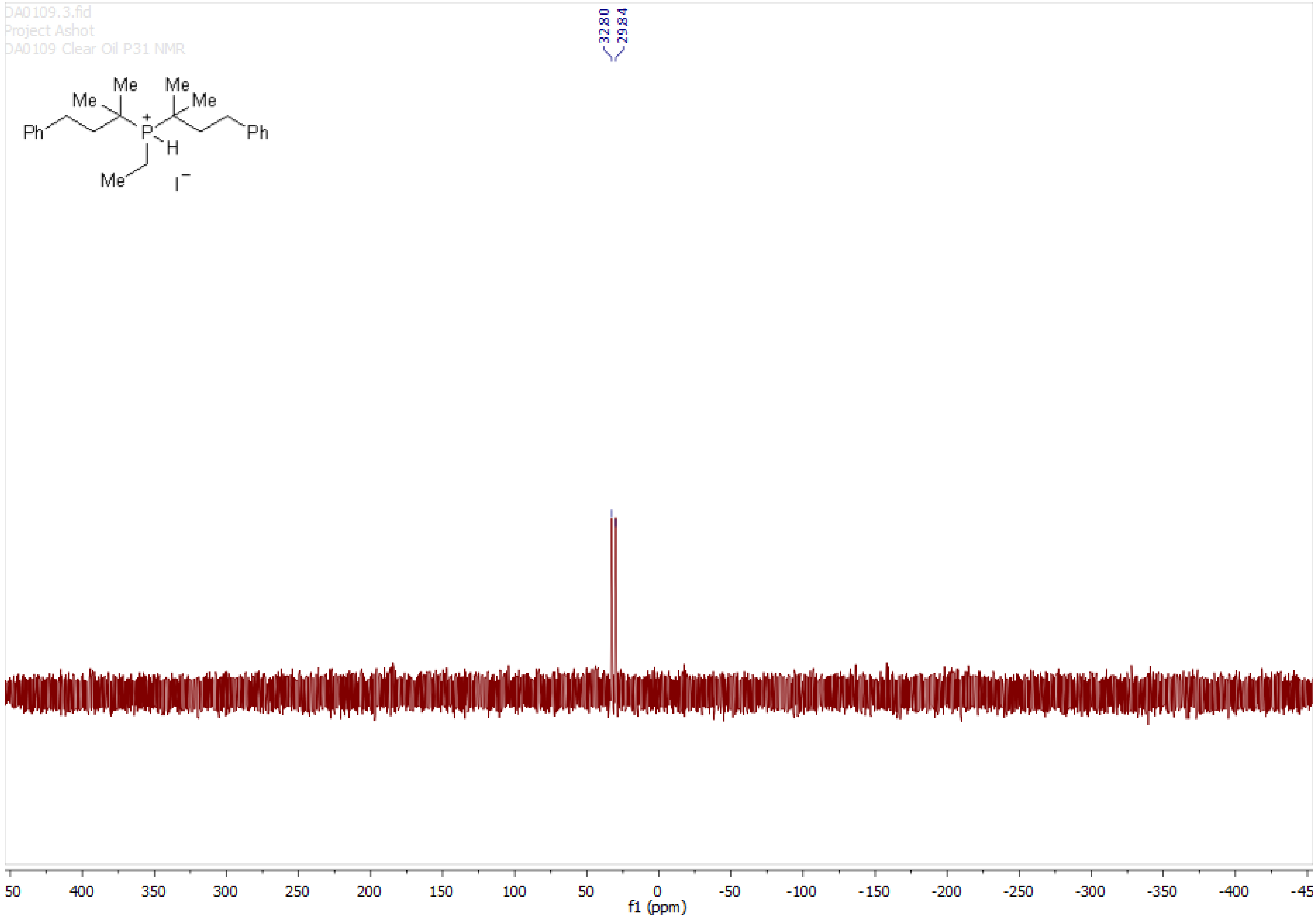

Spectrum S32. The ^{1}H NMR spectrum of *GreenPhos 5* in $CDCl_3$.

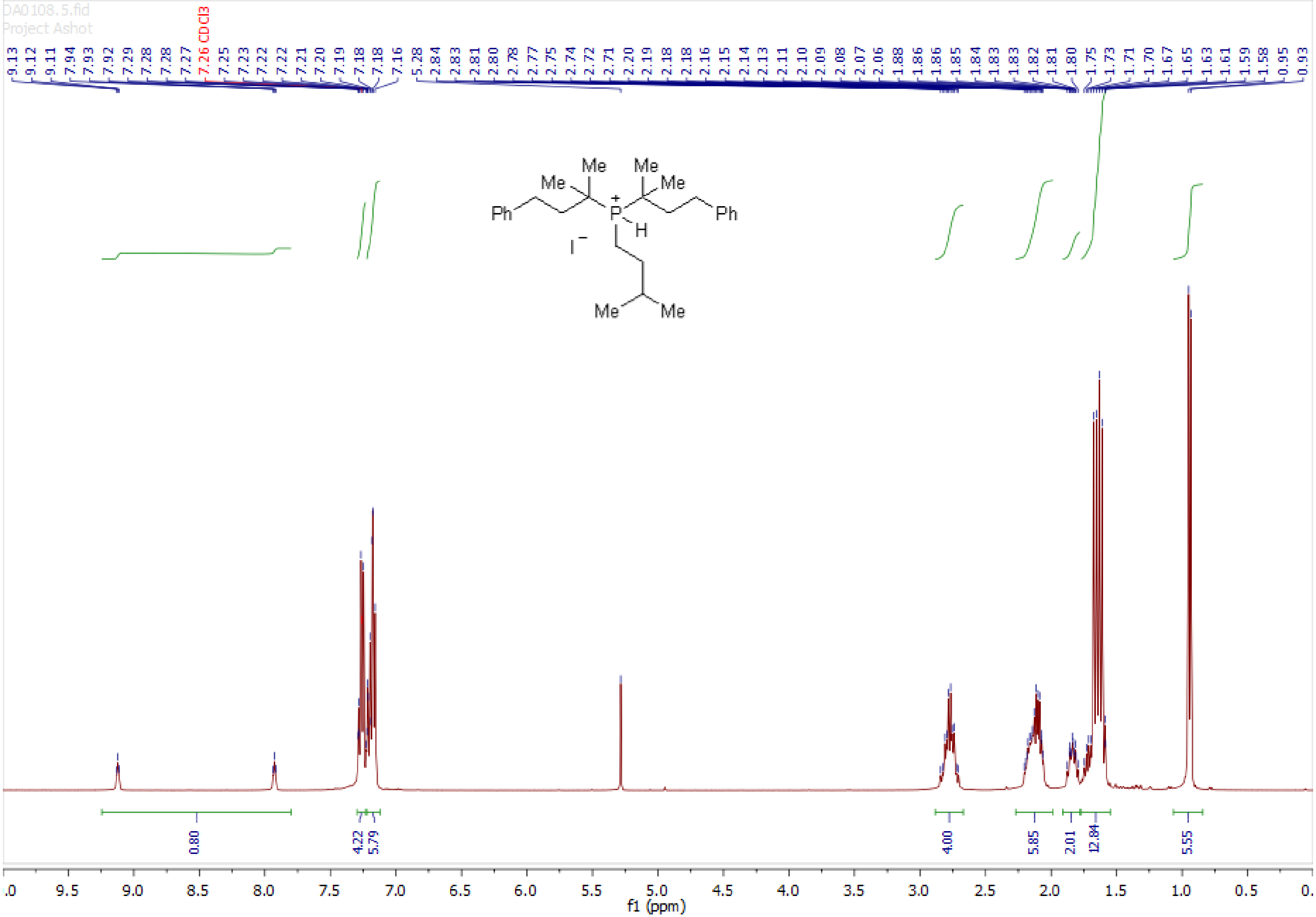

Spectrum S33. The ^{13}C NMR spectrum of *GreenPhos 5* in $CDCl_3$.

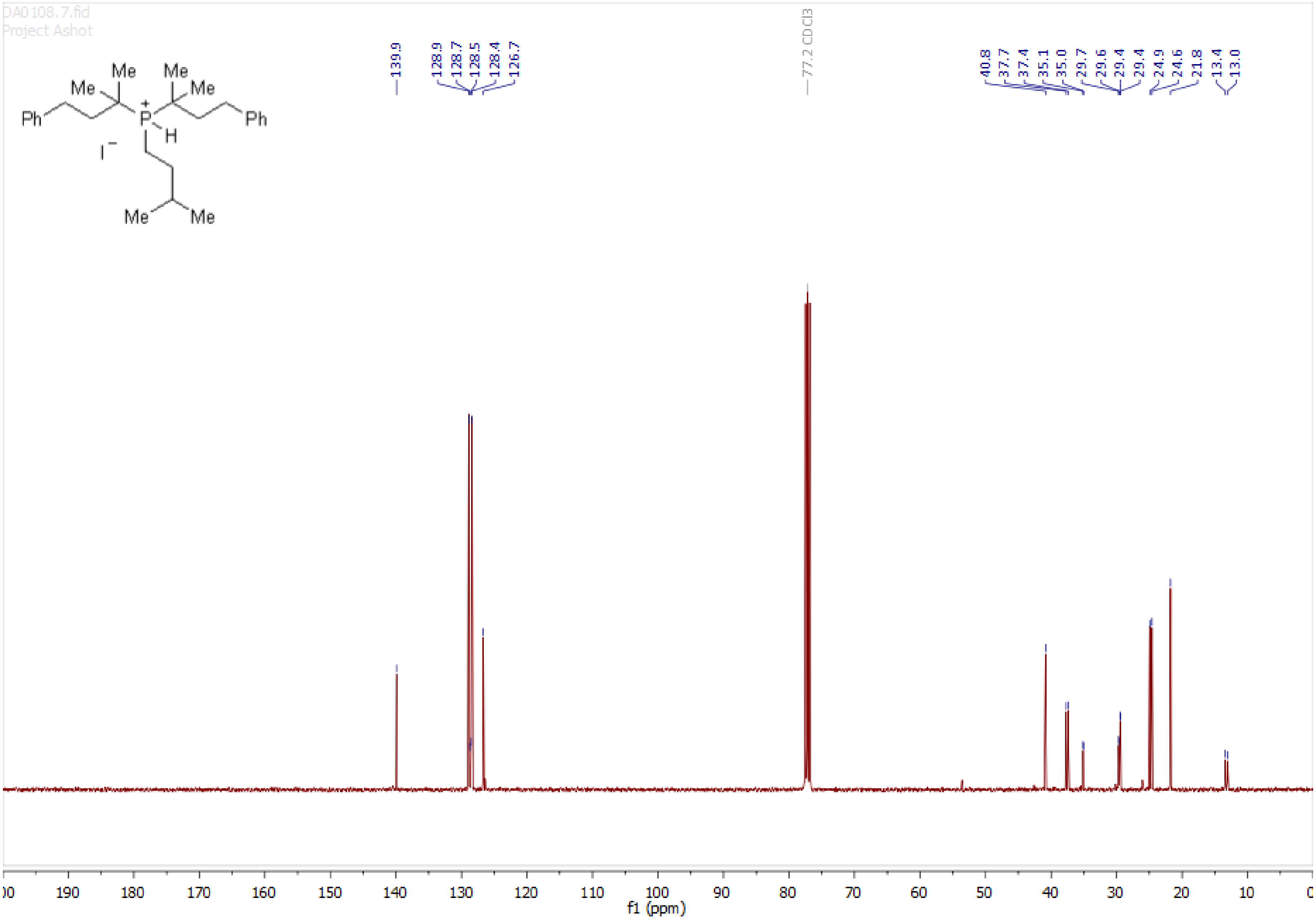

Spectrum S34. The ^{31}P NMR spectrum of *GreenPhos 5* with composite pulse decoupling in $CDCl_3$.

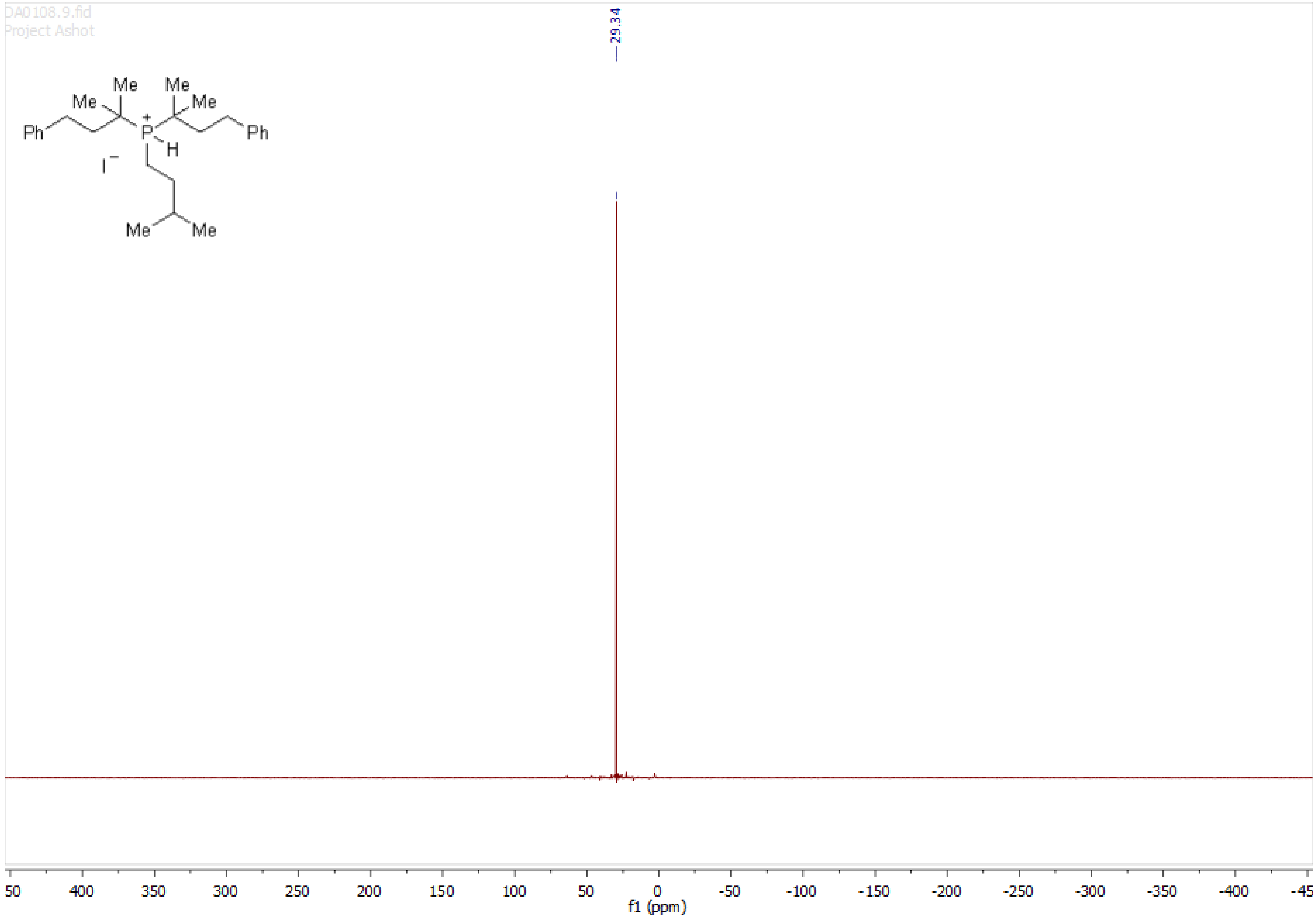

Spectrum S35. The ^{31}P NMR spectrum of *GreenPhos 5* with no decoupling in $CDCl_3$.

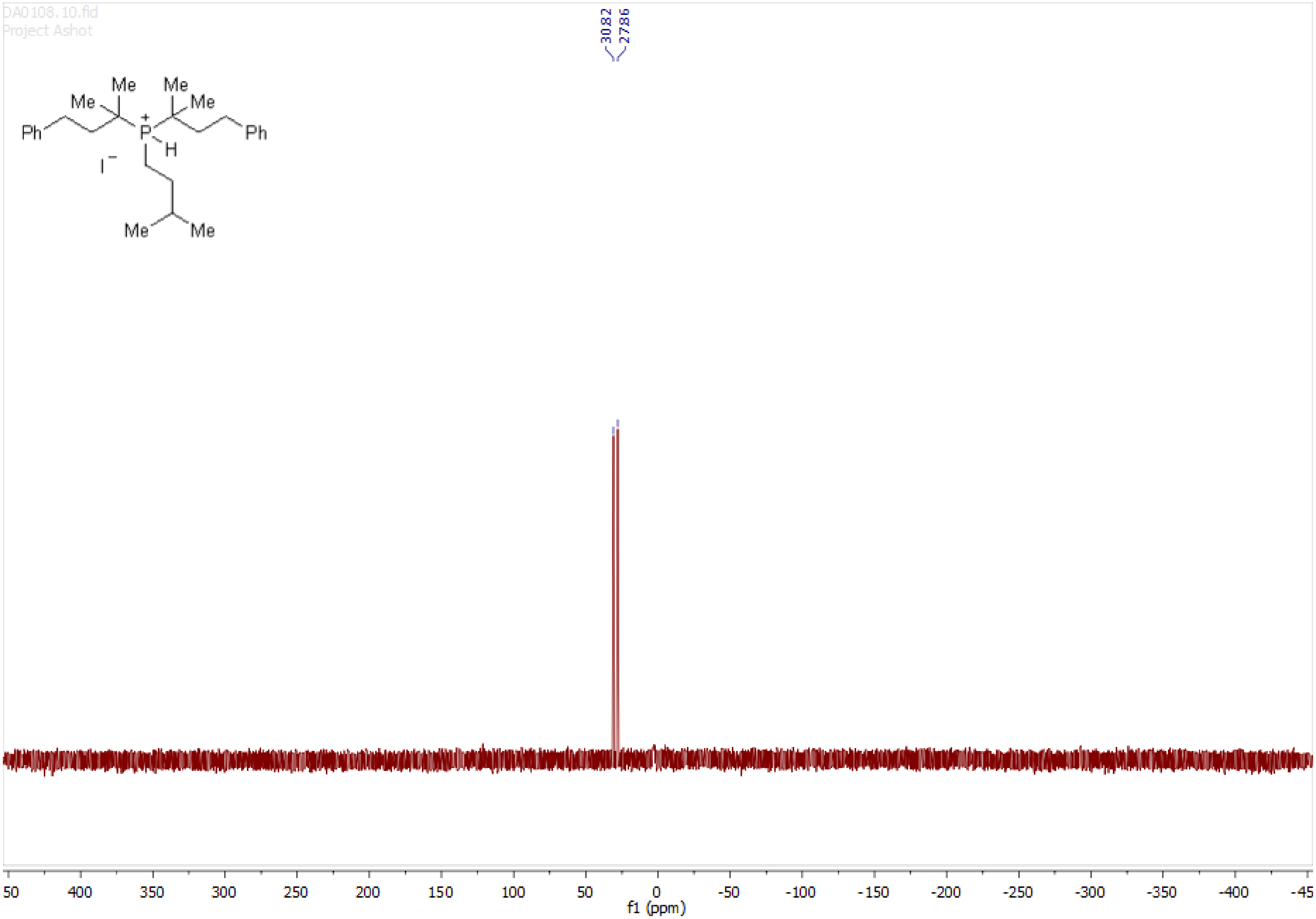

Spectrum S36. The ^{1}H NMR spectrum of *GreenPhos 6* in $CDCl_3$.

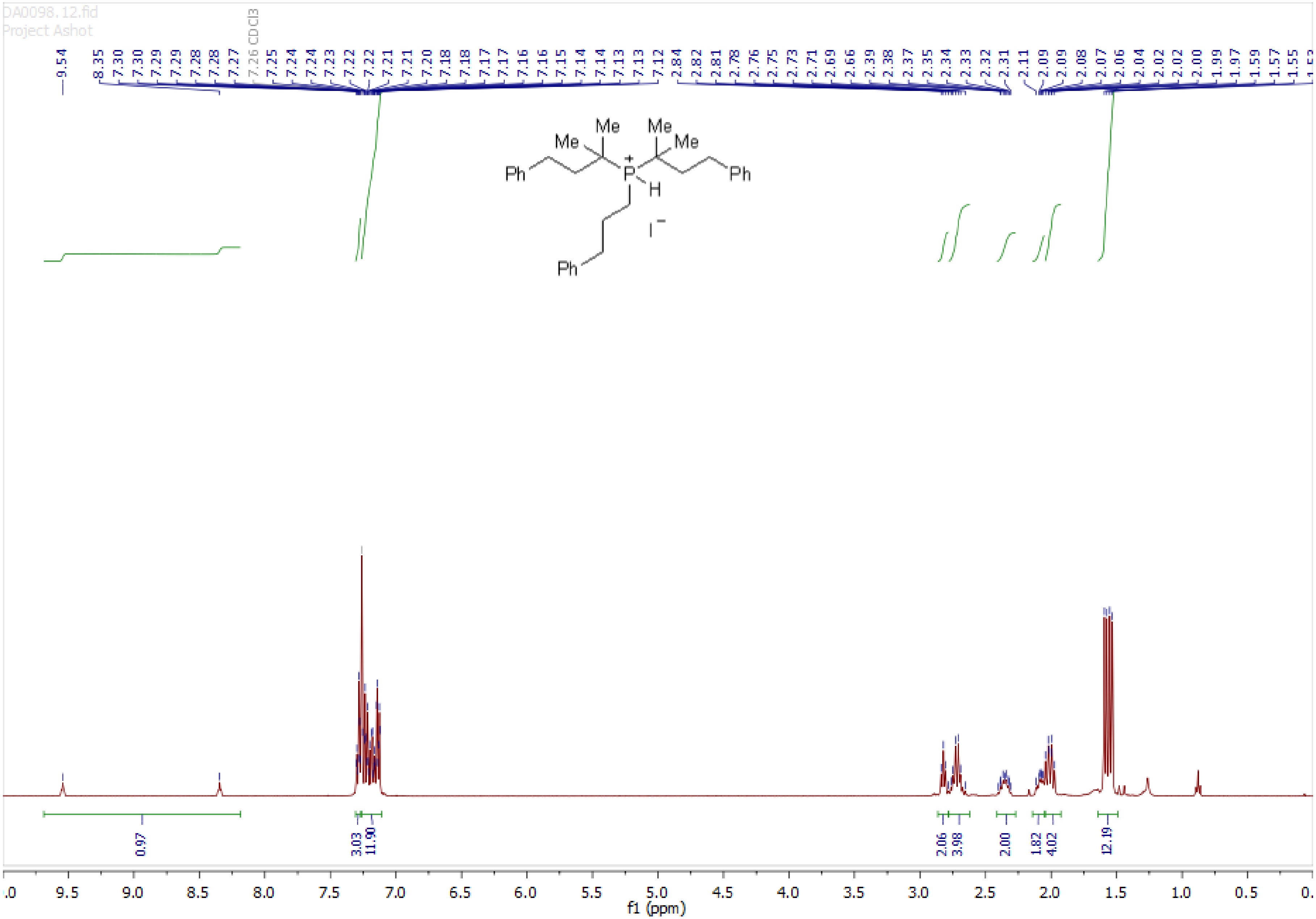

Spectrum S37. The ^{13}C NMR spectrum of *GreenPhos 6* in $CDCl_3$.

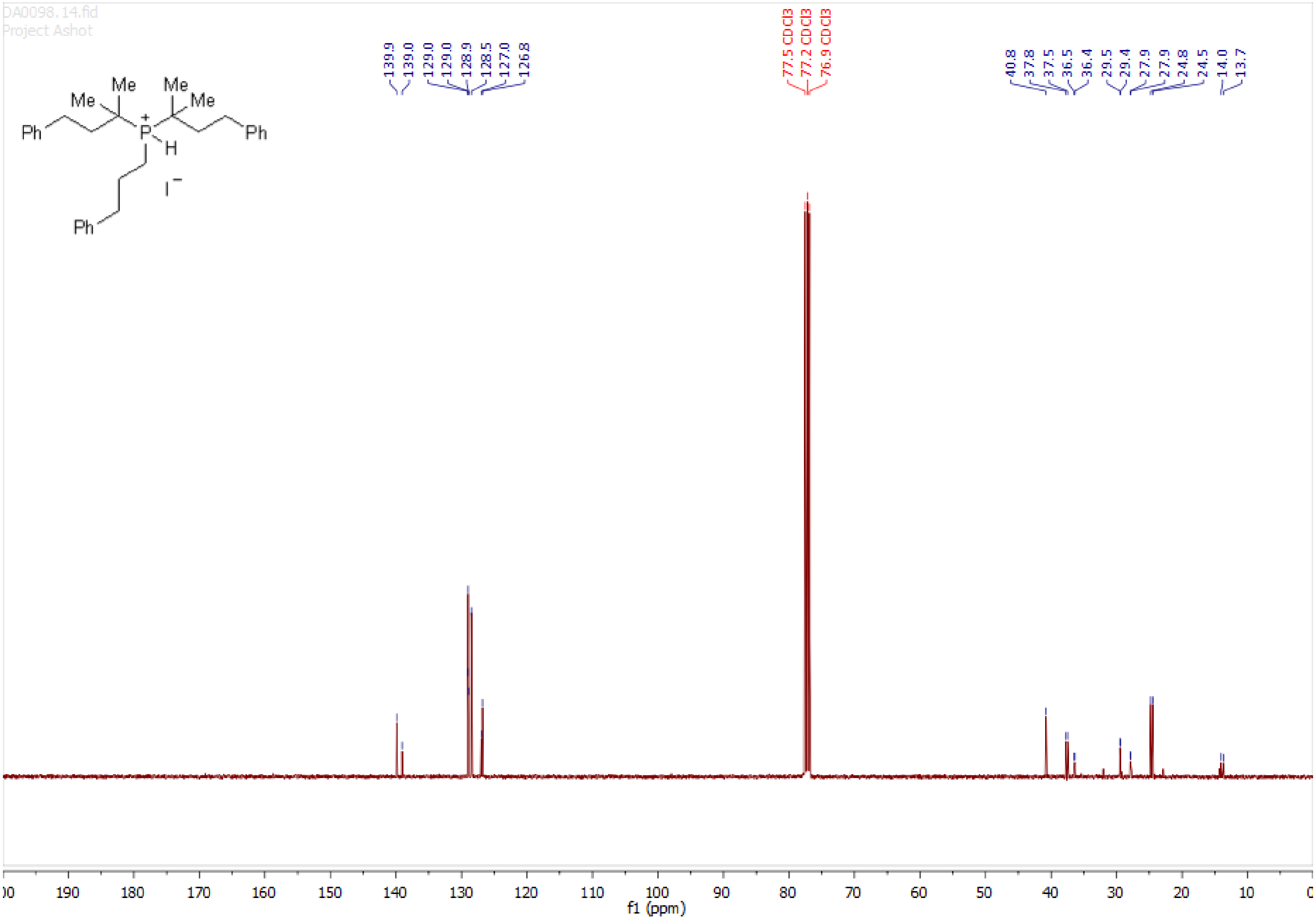

Spectrum S38. The ^{31}P NMR spectrum of *GreenPhos 6* with composite pulse decoupling in $CDCl_3$.

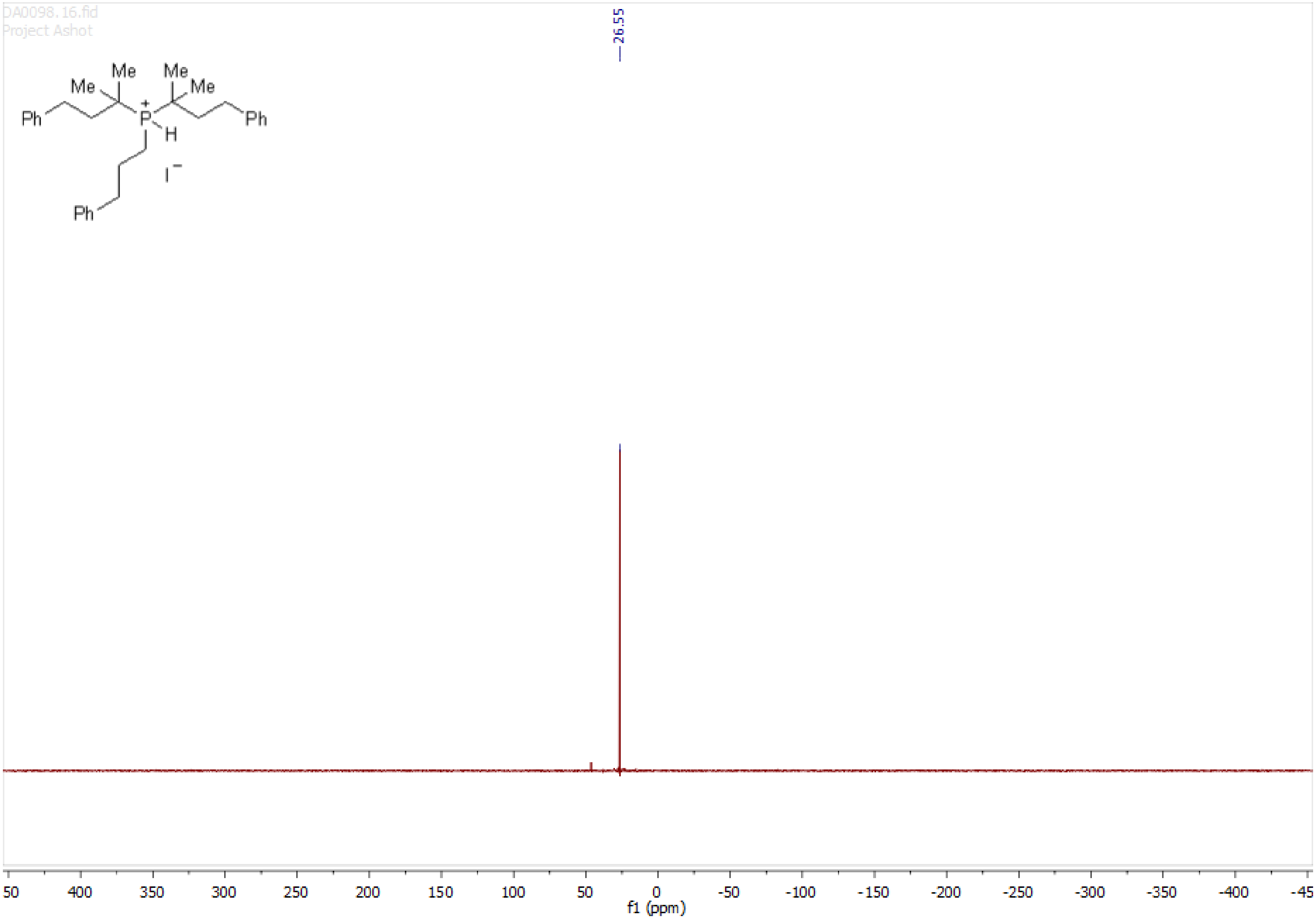

Spectrum S39. The ^{31}P NMR spectrum of *GreenPhos 6* with no decoupling in $CDCl_3$.

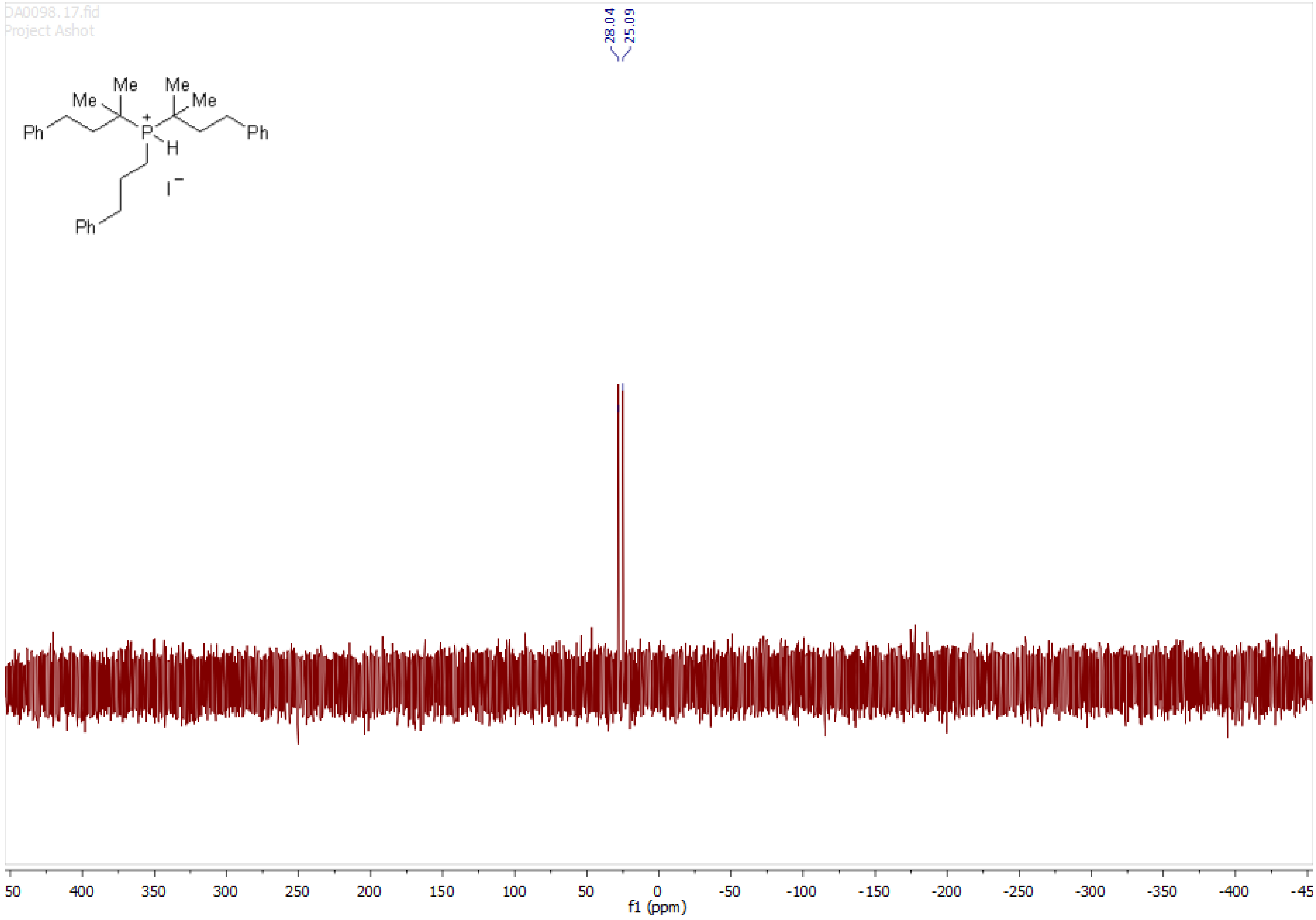

Spectrum S40. The ^1H NMR spectrum of *GreenPhos 7* in $CDCl_3$.

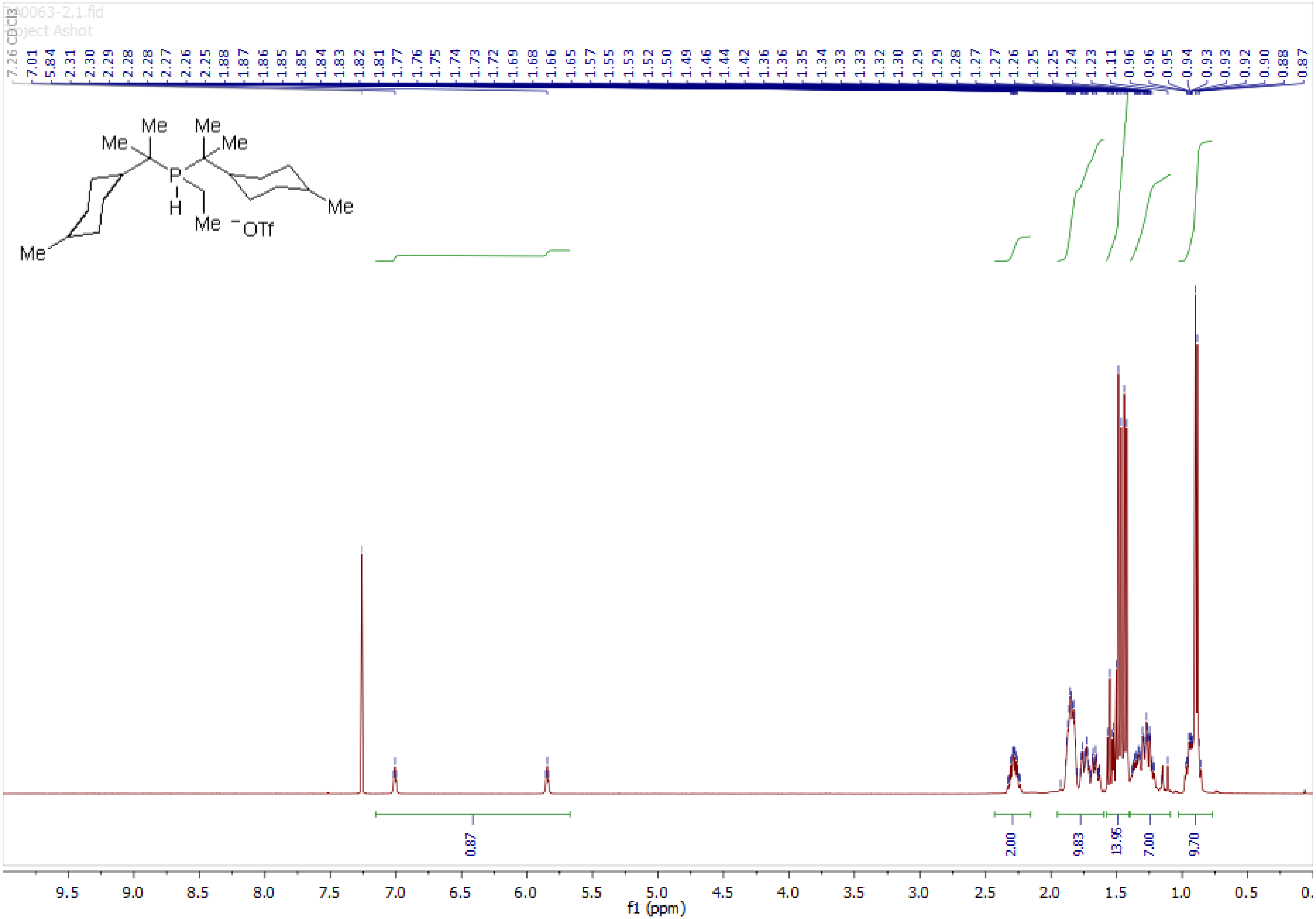

Spectrum S41. The ^{13}C NMR spectrum of *GreenPhos 7* in $CDCl_3$.

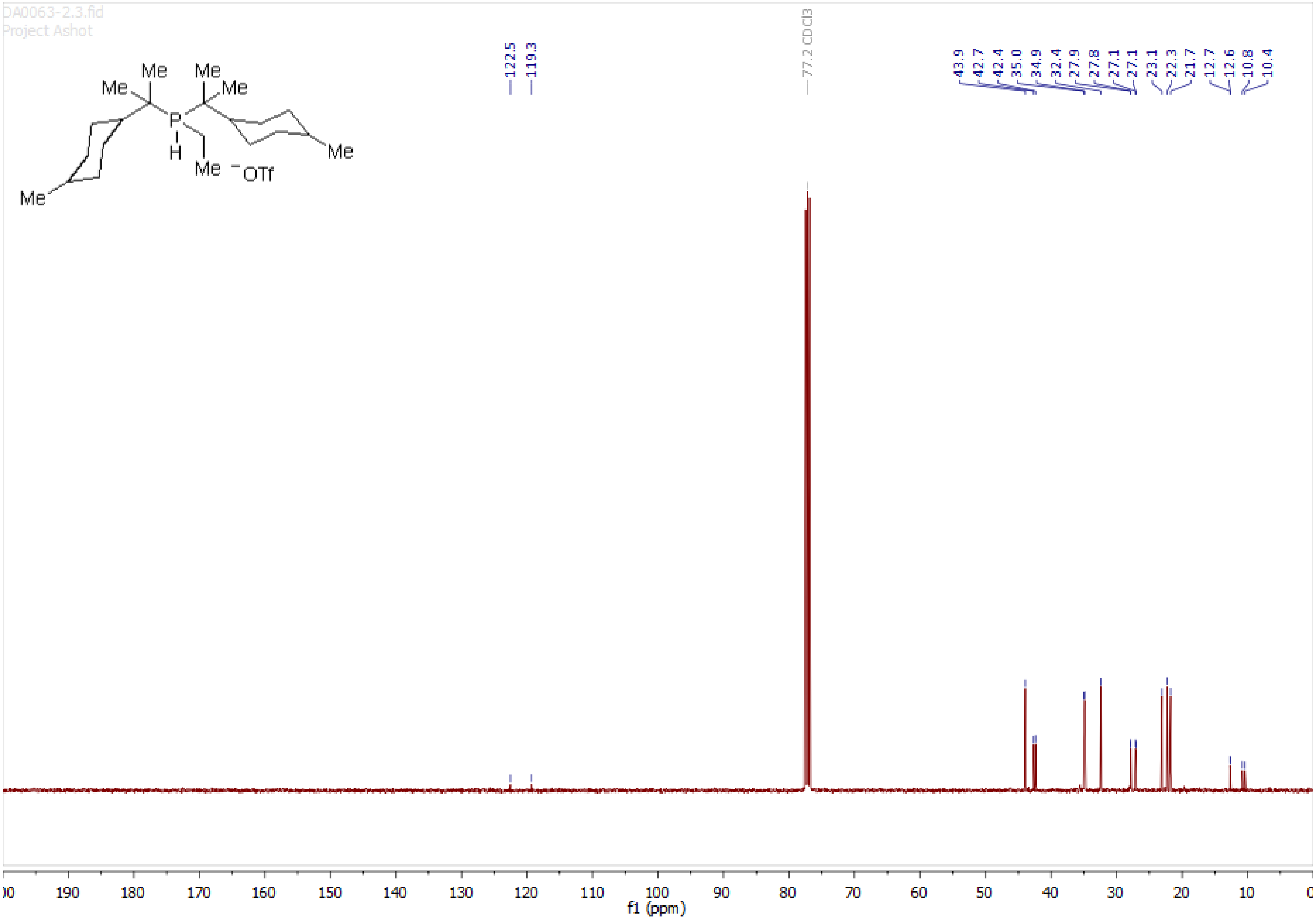

Spectrum S42. The ^{31}P NMR spectrum of *GreenPhos 7* with composite pulse decoupling in $CDCl_3$.

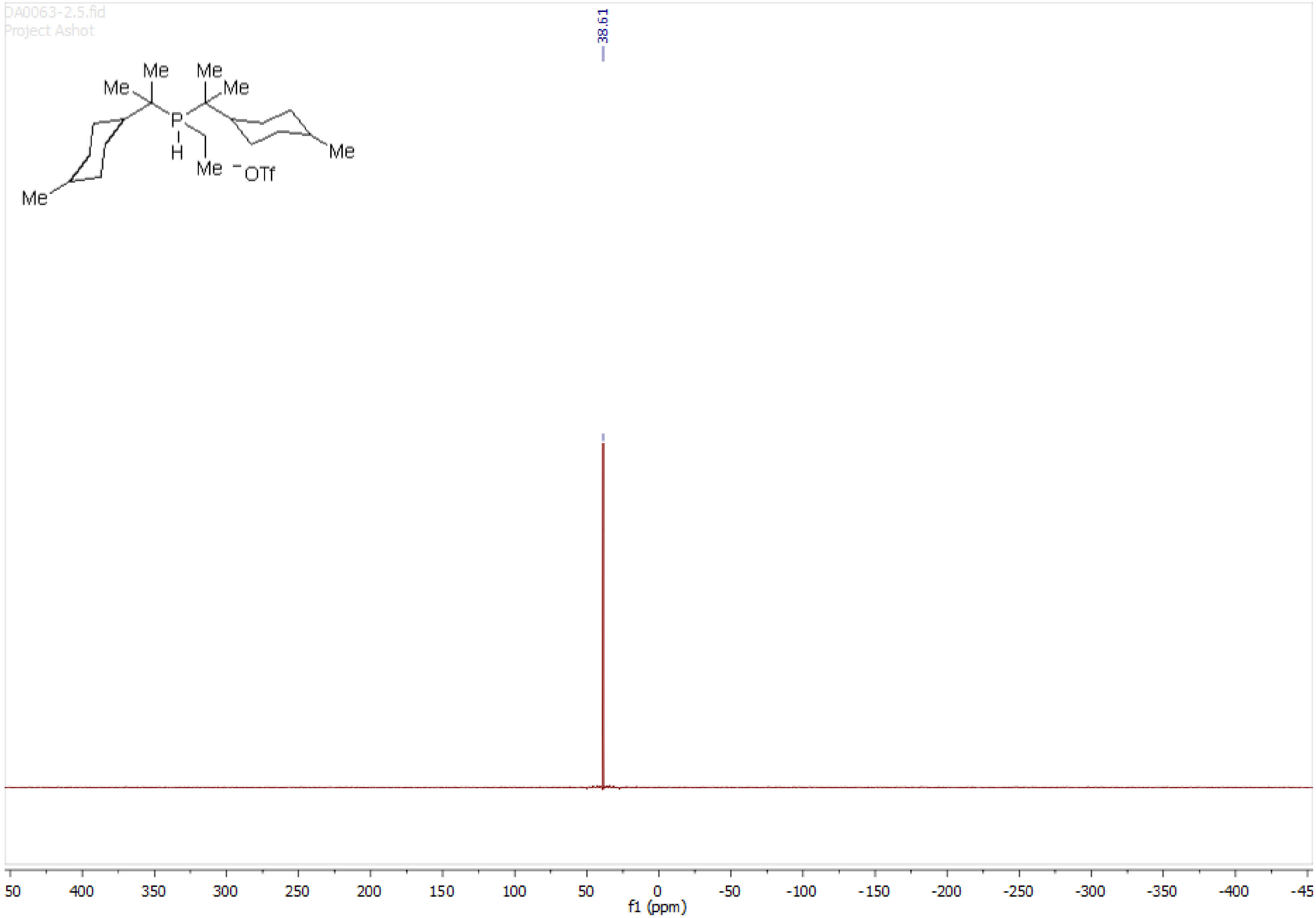

Spectrum S43. The ^{31}P NMR spectrum of *GreenPhos 7* with no decoupling in $CDCl_3$.

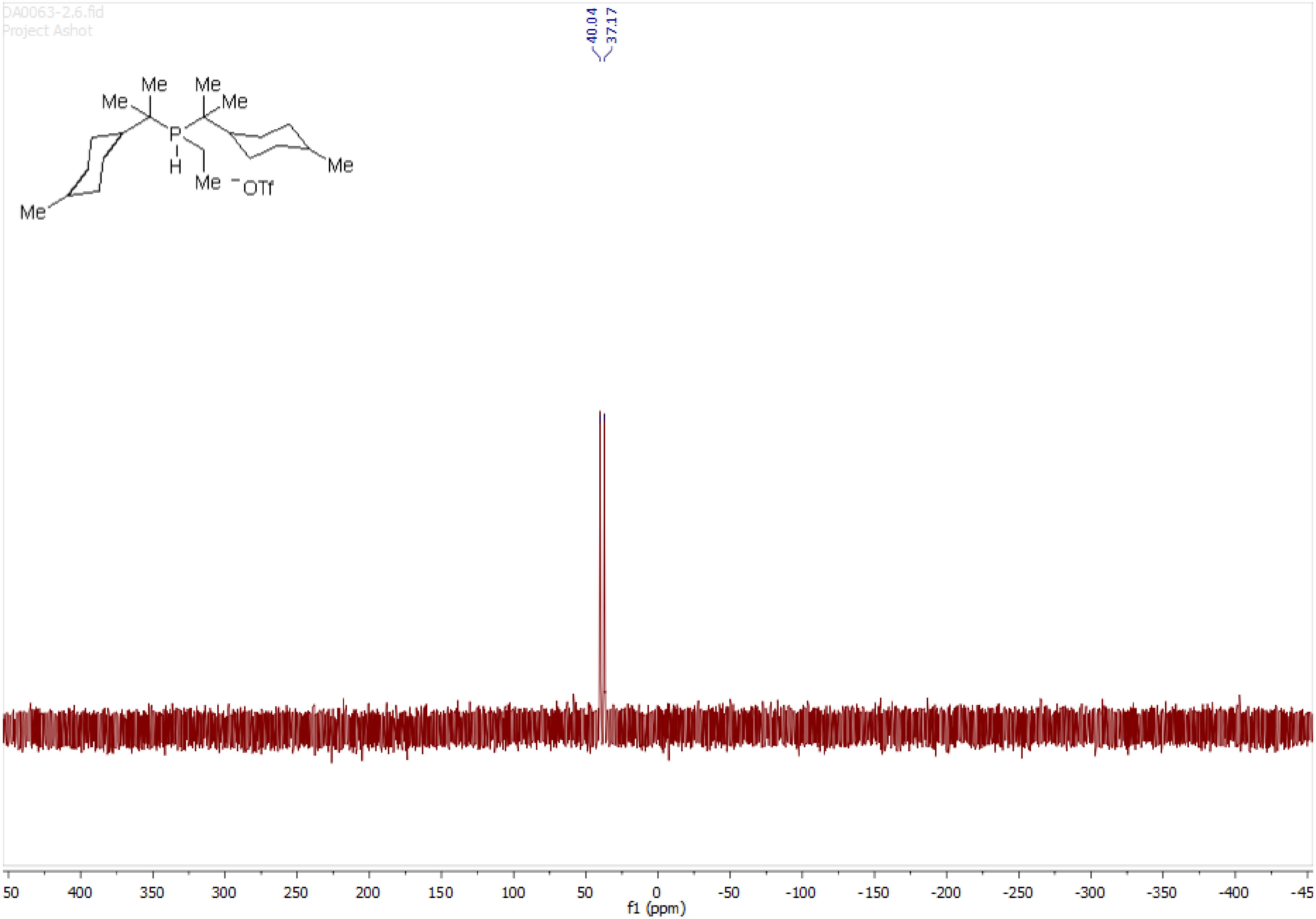

Spectrum S44. The ^{19}F NMR spectrum of *GreenPhos 7* in $CDCl_3$.

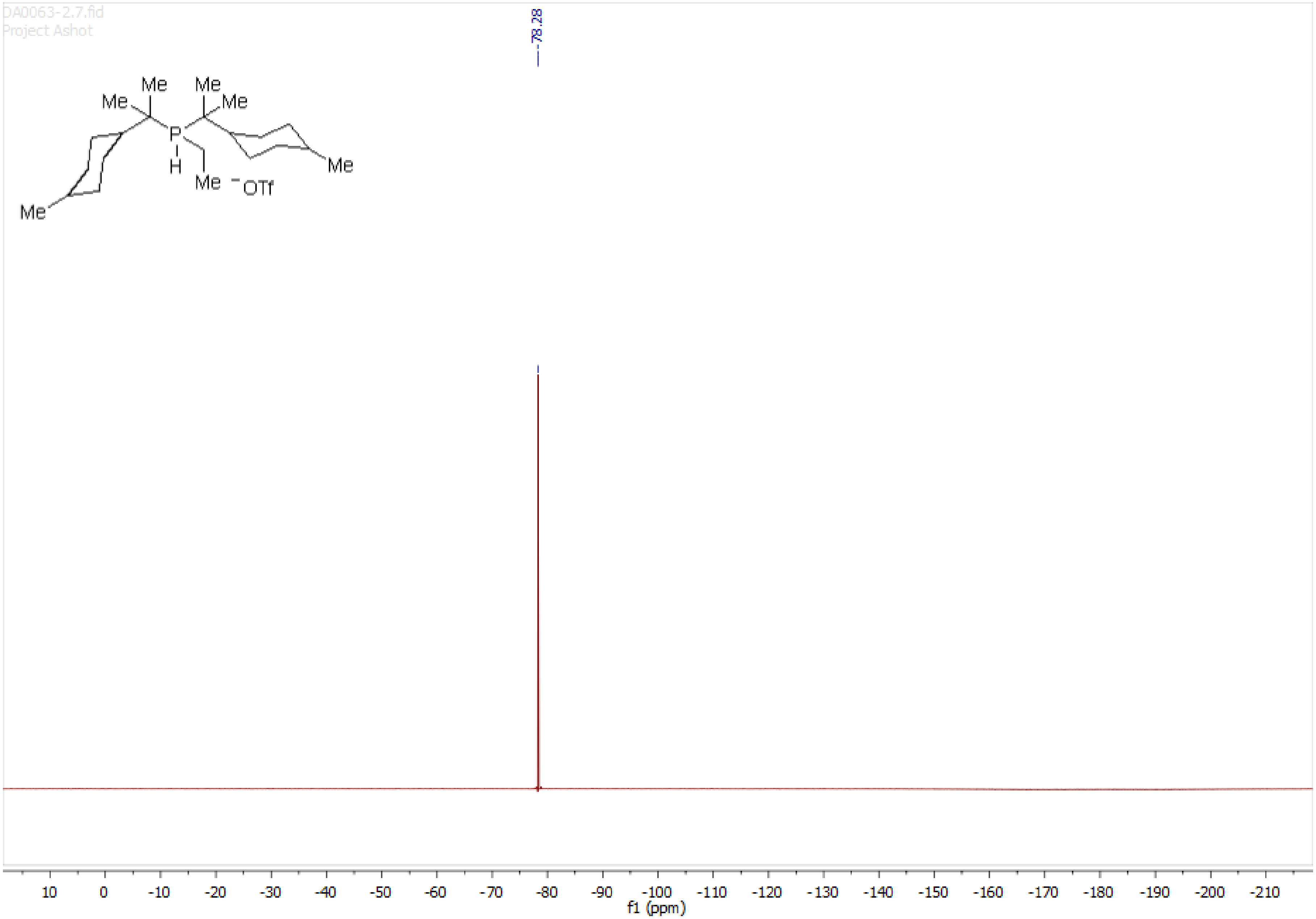

Spectrum S45. The ^{1}H NMR spectrum of *GreenPhos 8* in $CDCl_3$.

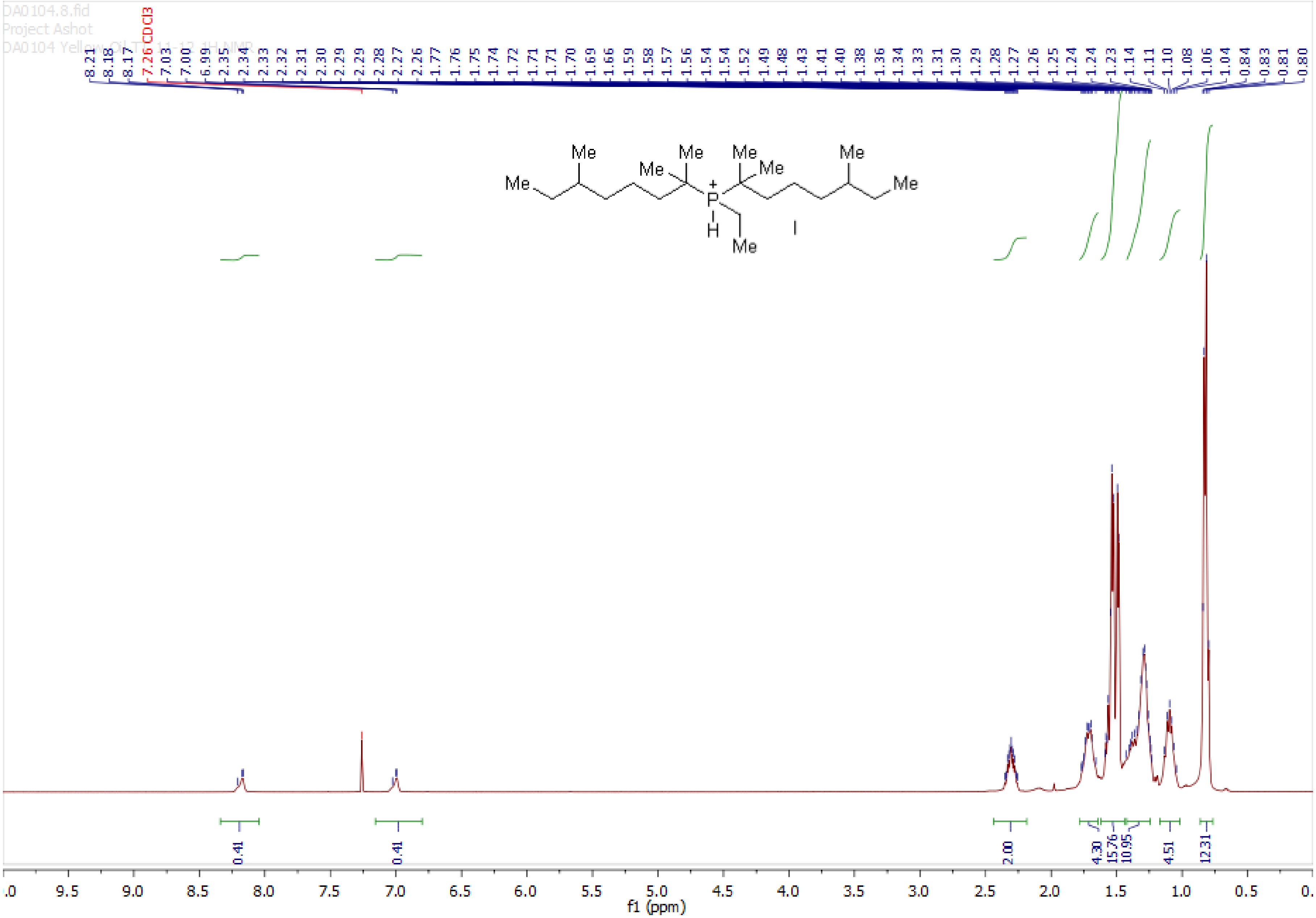

Spectrum S46. The ^{13}C NMR spectrum of *GreenPhos 8* in $CDCl_3$.

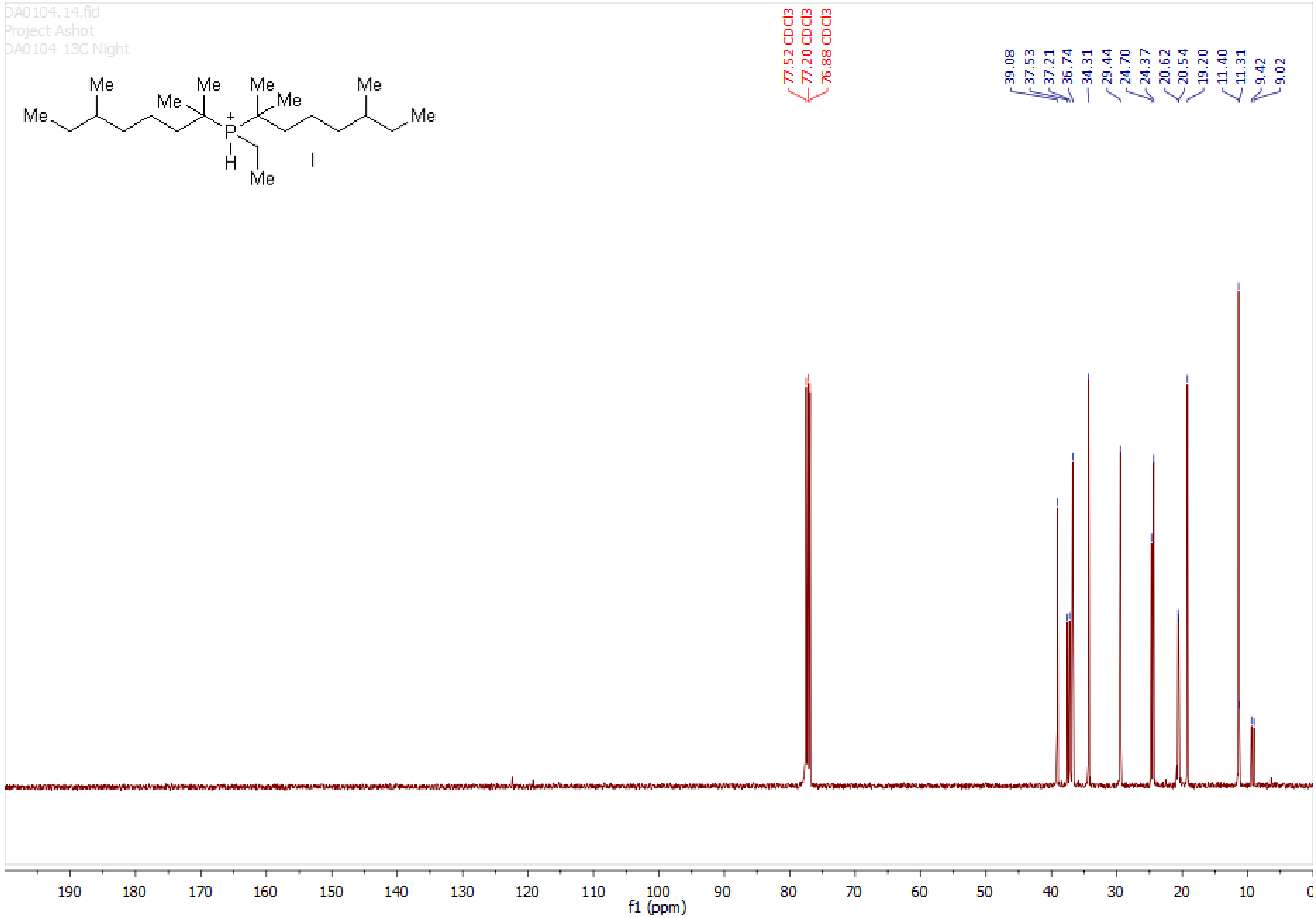

Spectrum S47. The ^{31}P NMR spectrum of *GreenPhos 8* with composite pulse decoupling in $CDCl_3$.

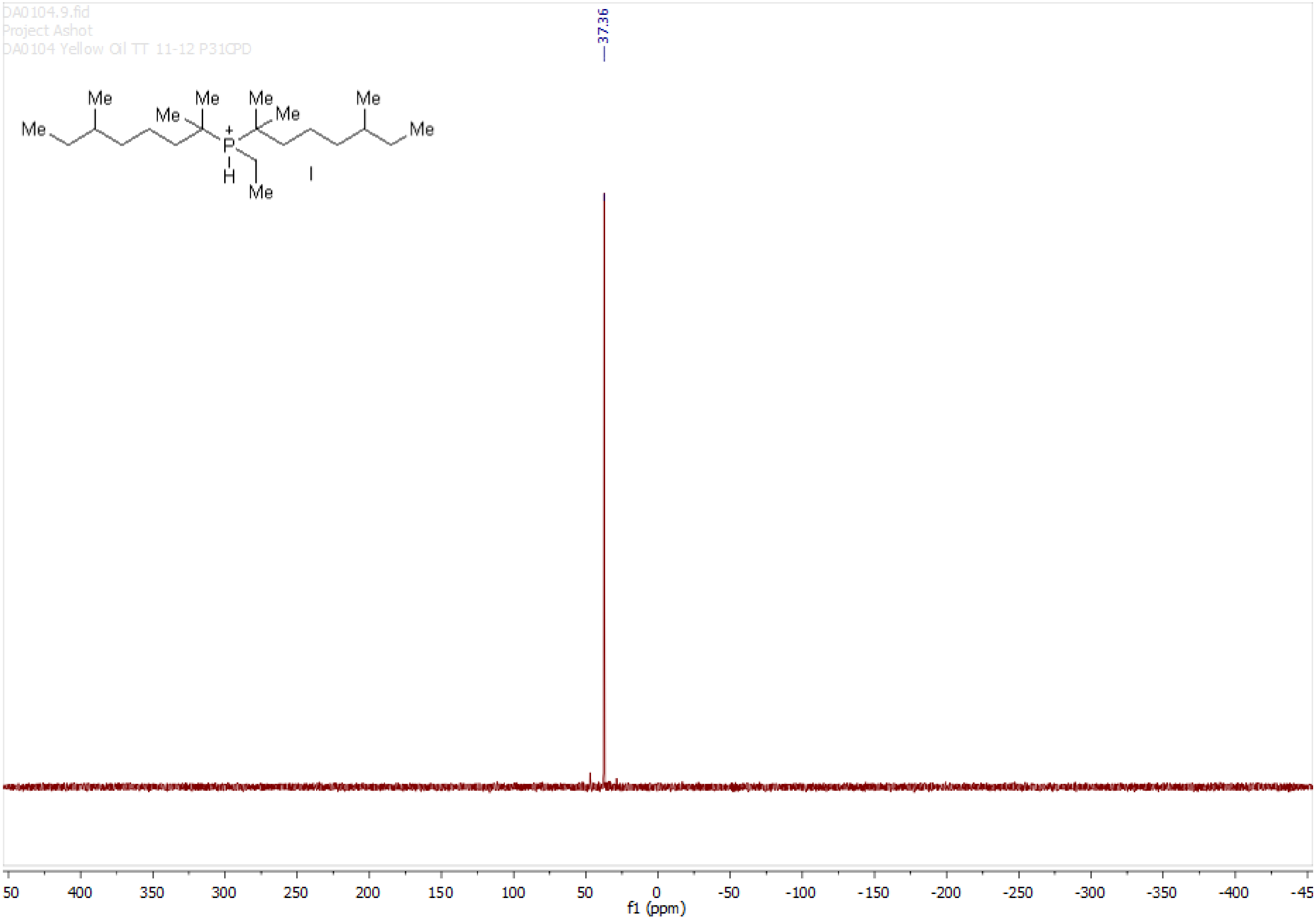

Spectrum S48. The ^{31}P NMR spectrum of *GreenPhos 8* with no decoupling in $CDCl_3$.

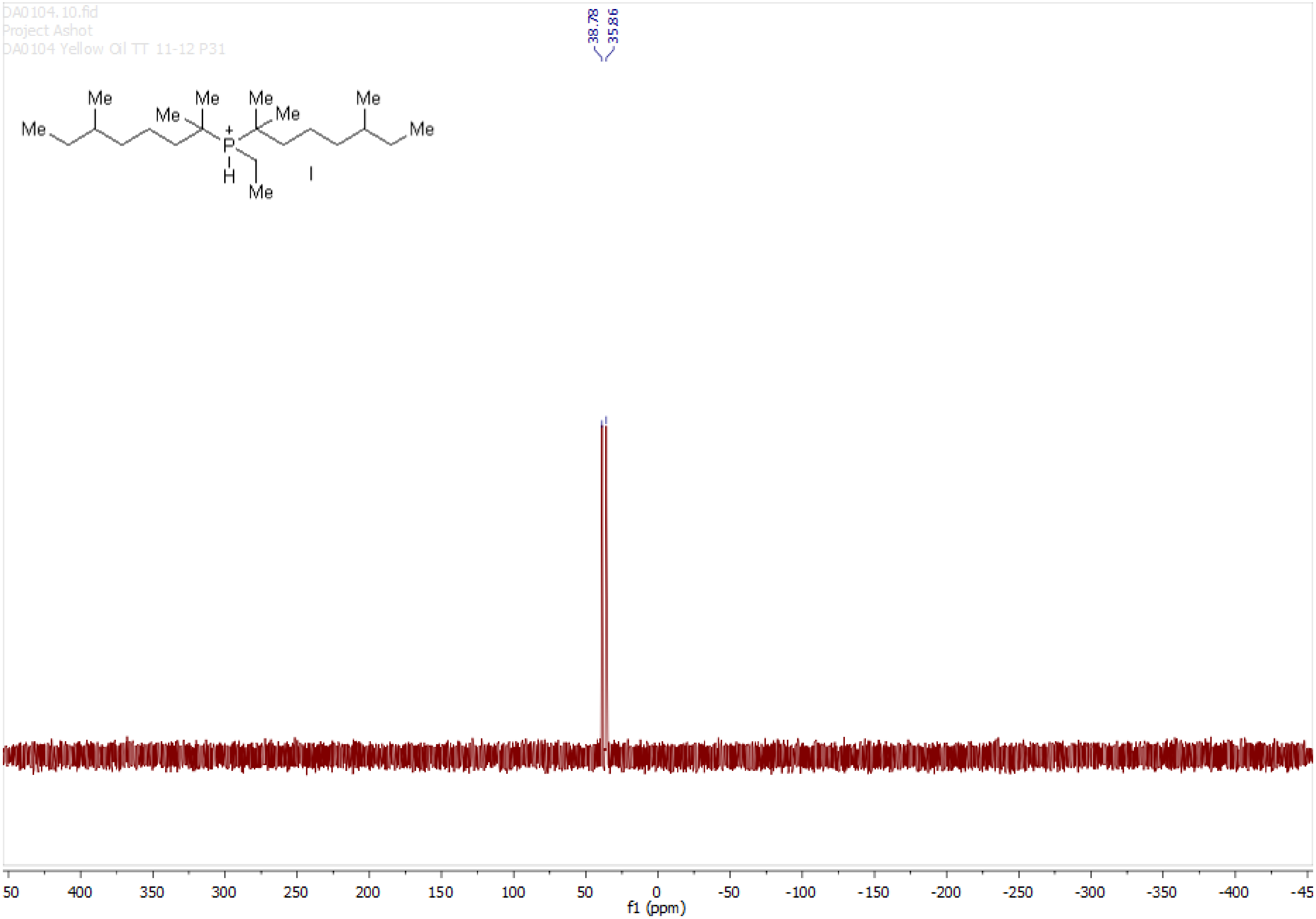

Spectrum S49. The ^{1}H NMR spectrum of *GreenPhos 9* in $CDCl_3$.

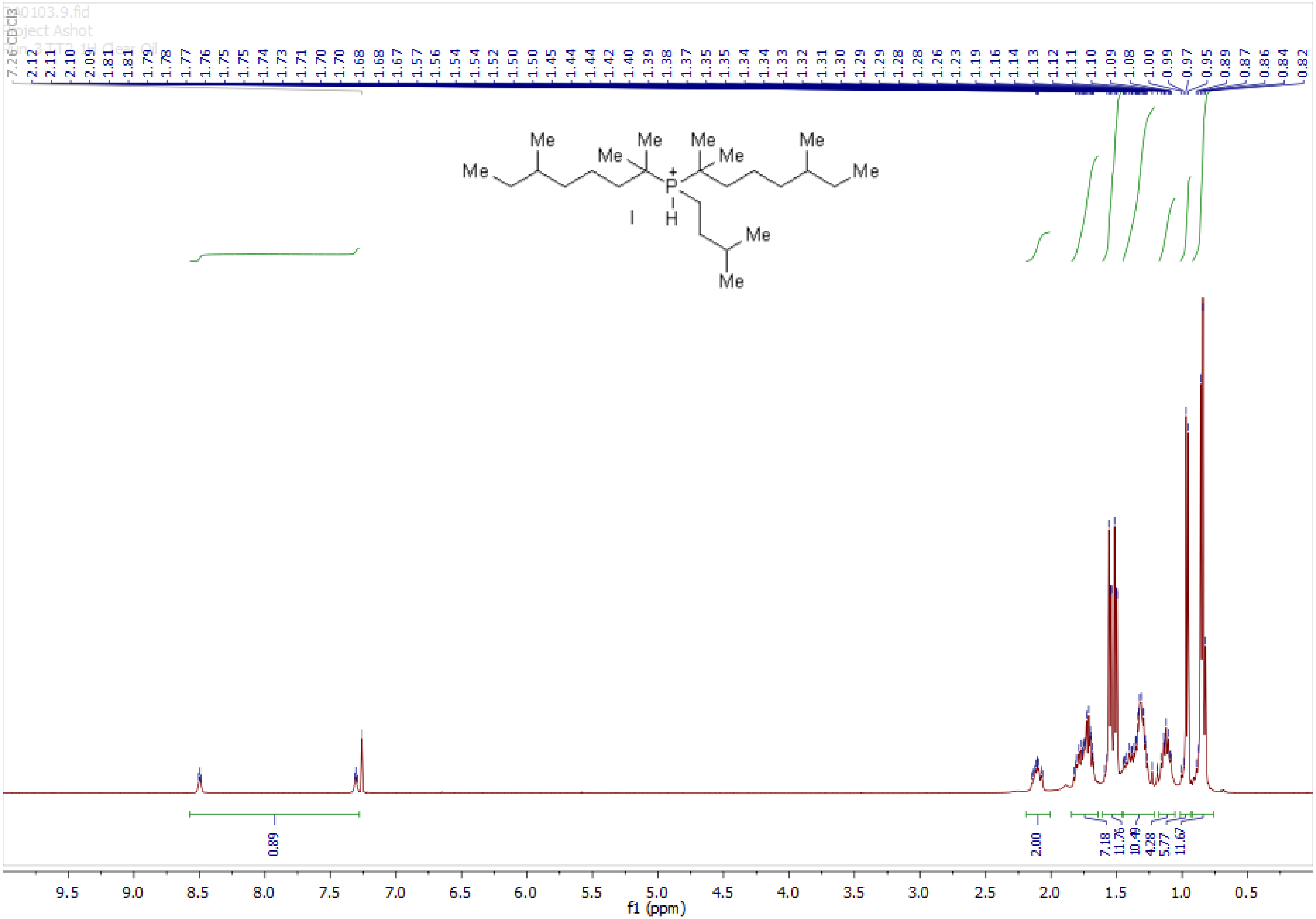

Spectrum S50. The ^{13}C NMR spectrum of *GreenPhos 9* in $CDCl_3$.

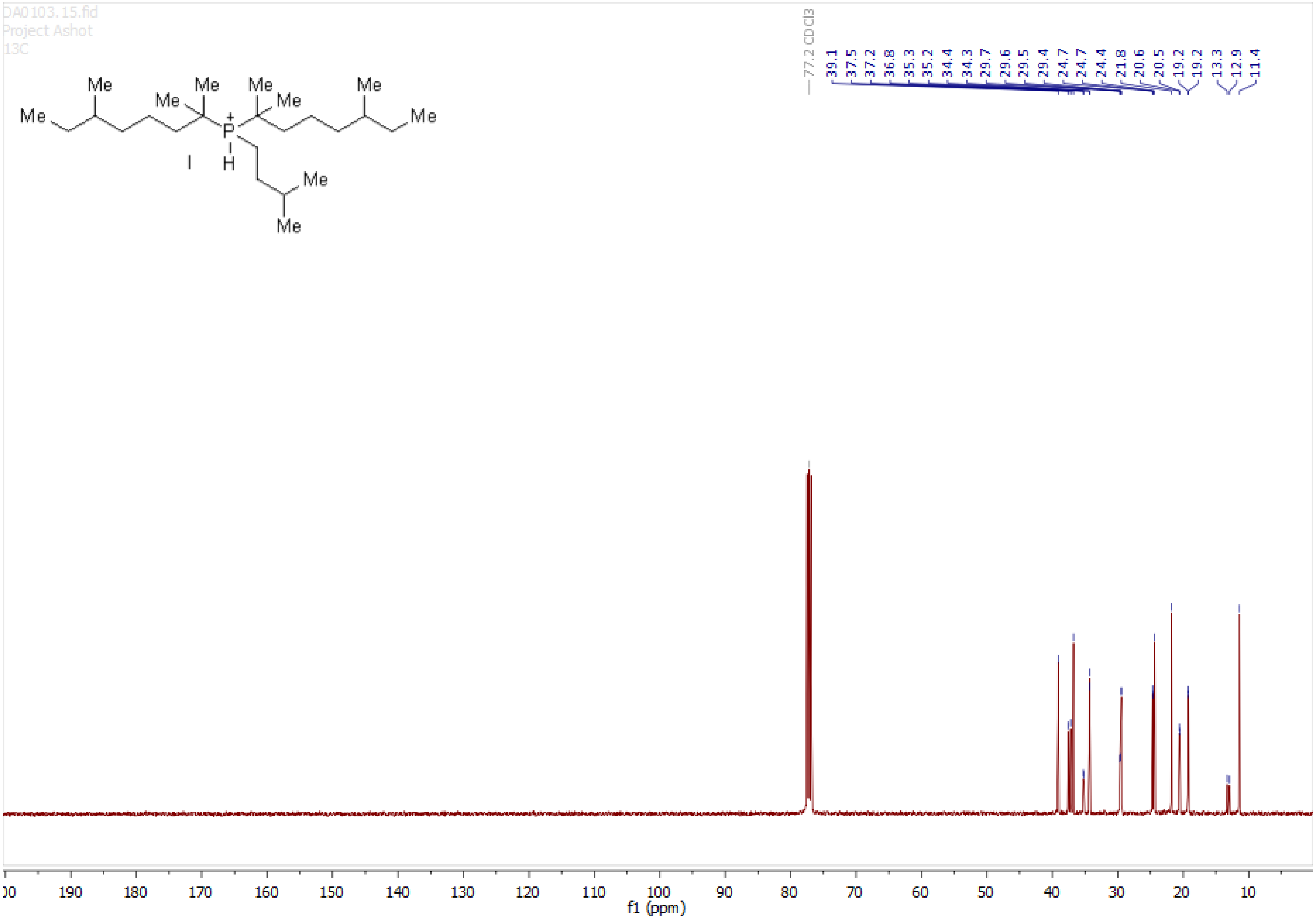

Spectrum S51. The ^{31}P NMR spectrum of *GreenPhos 9* with composite pulse decoupling in $CDCl_3$.

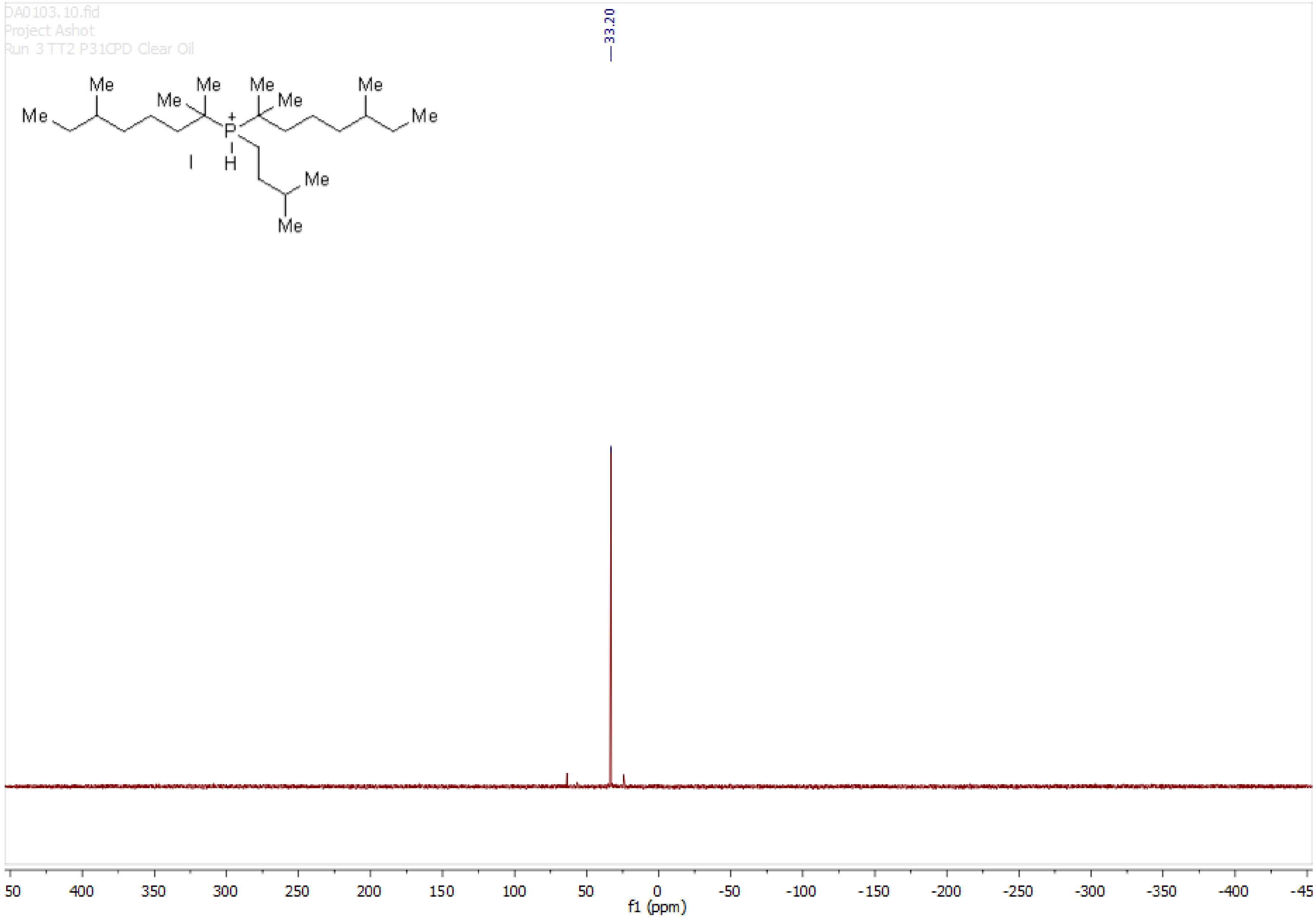

Spectrum S52. The ^{31}P NMR spectrum of *GreenPhos 9* with no decoupling in $CDCl_3$.

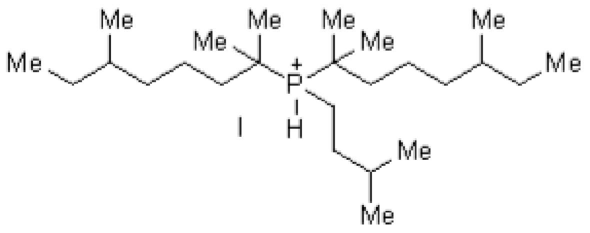

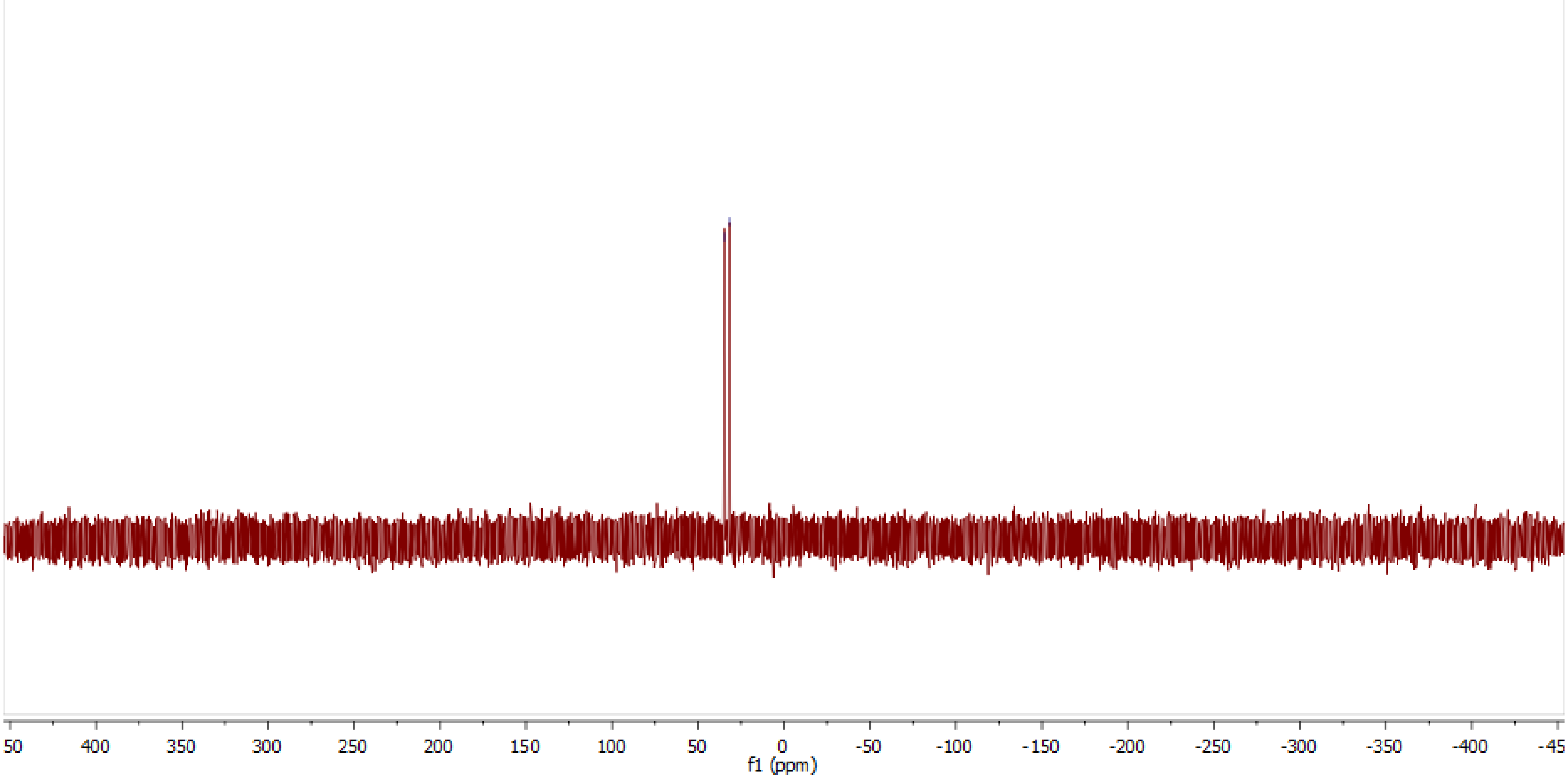

Spectrum S53. The ^1H NMR spectrum of *GreenPhos 10* in $CDCl_3$.

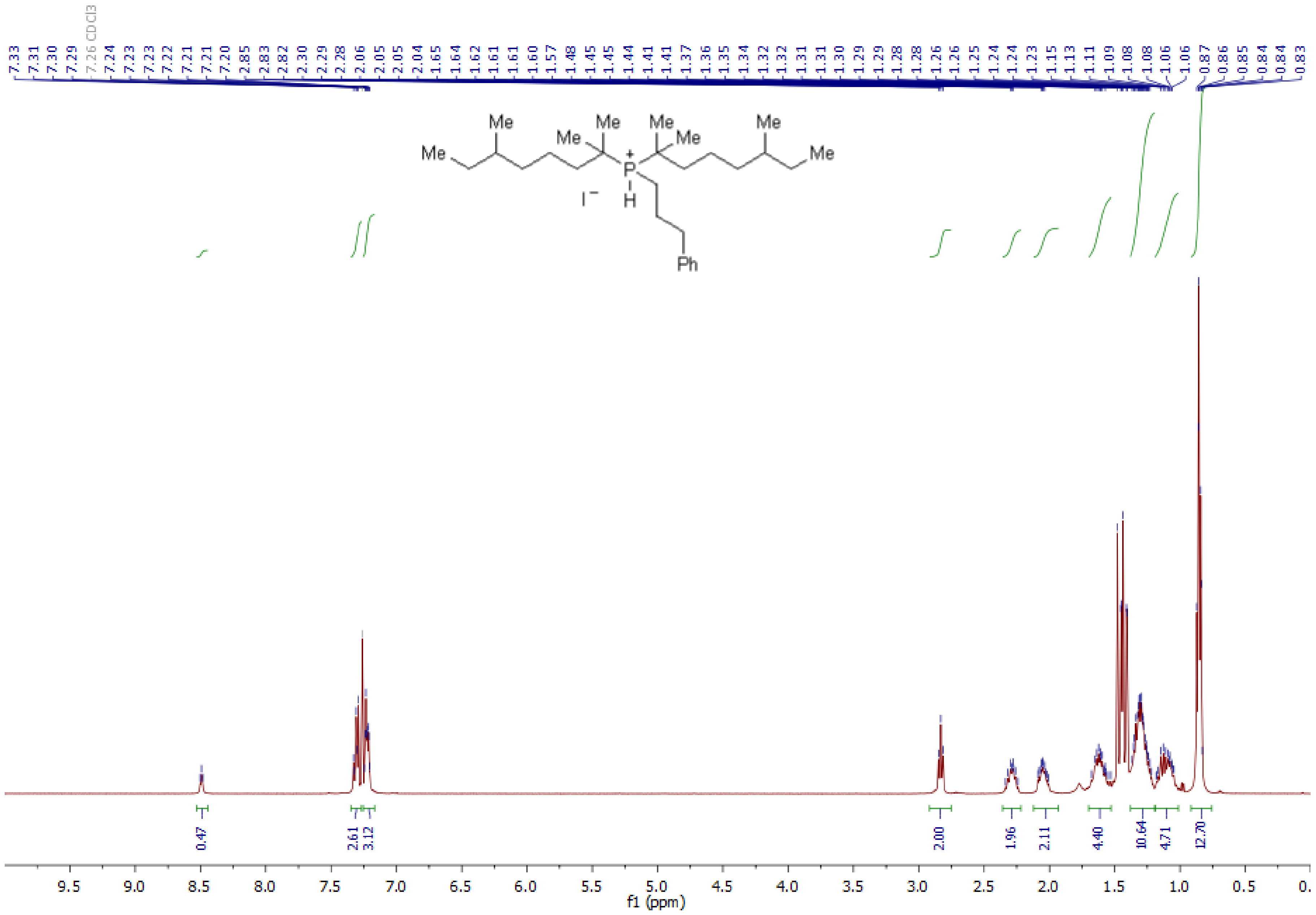

Spectrum S54. The ^{13}C NMR spectrum of *GreenPhos 10* in $CDCl_3$.

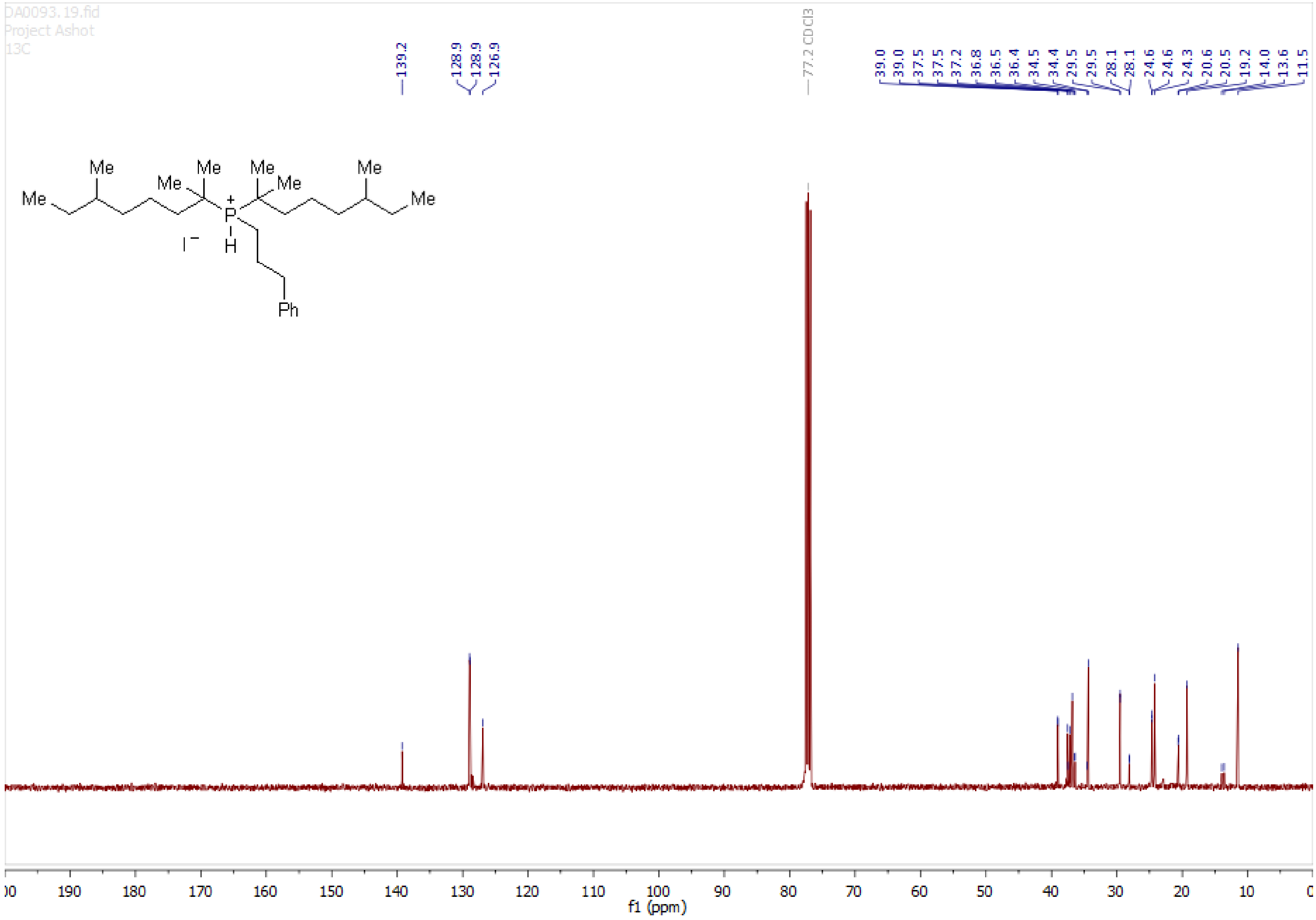

Spectrum S55. The ^{31}P NMR spectrum of *GreenPhos 10* with composite pulse decoupling in $CDCl_3$.

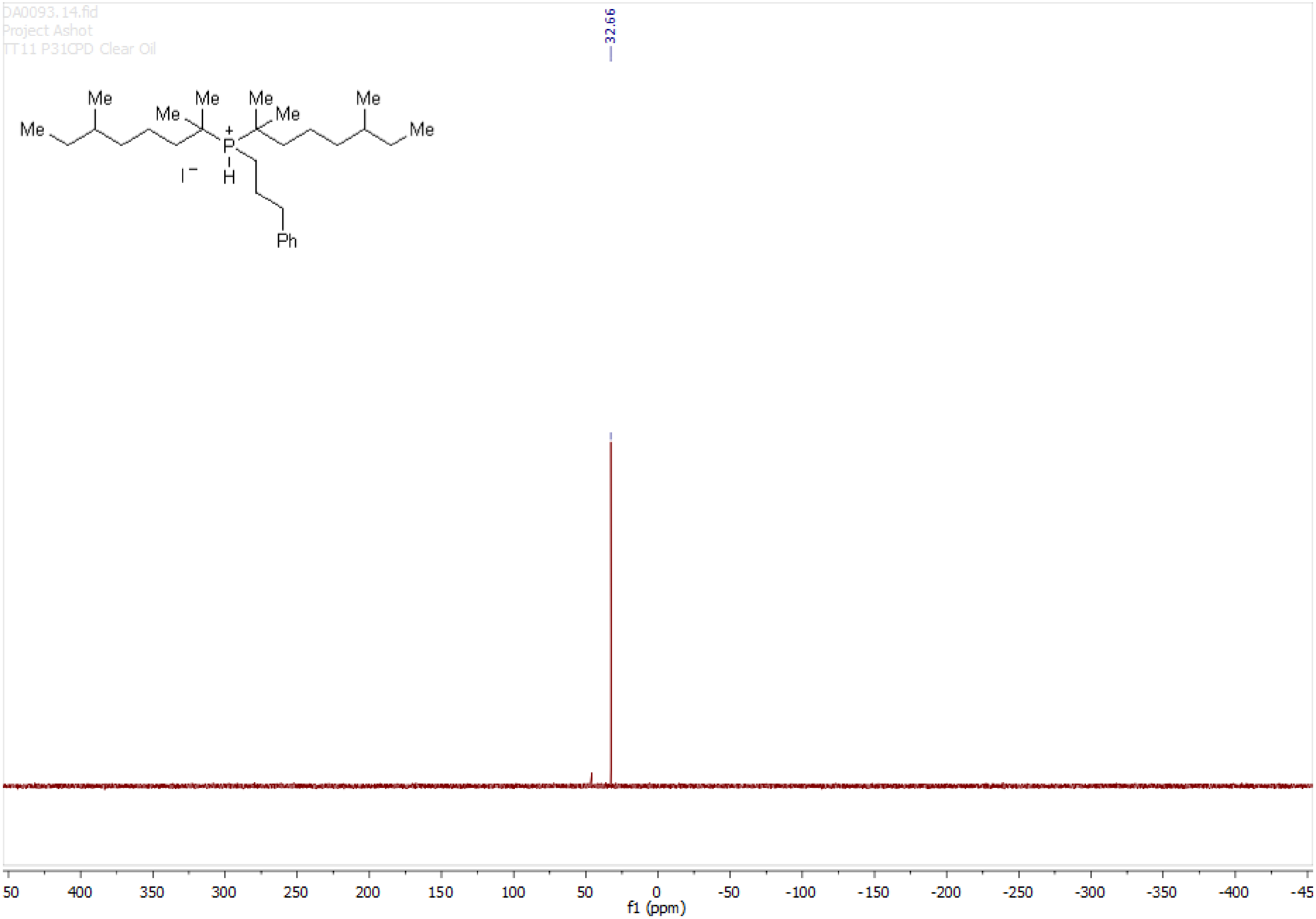

Spectrum S56. The ^{31}P NMR spectrum of *GreenPhos 10* with no decoupling in $CDCl_3$.

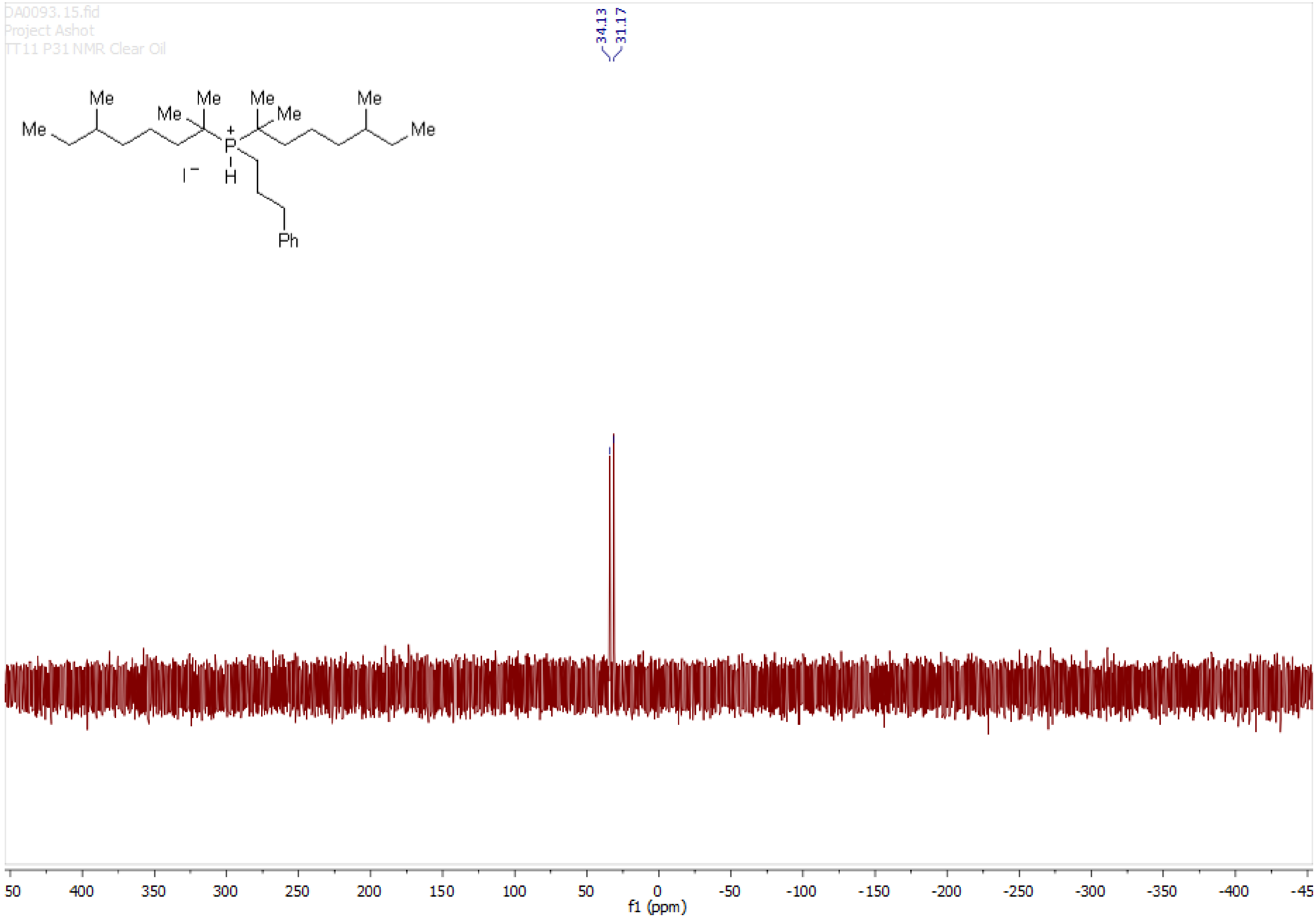

Spectrum S57. The ^{1}H NMR spectrum of *GreenPhos 11* in $CDCl_3$.

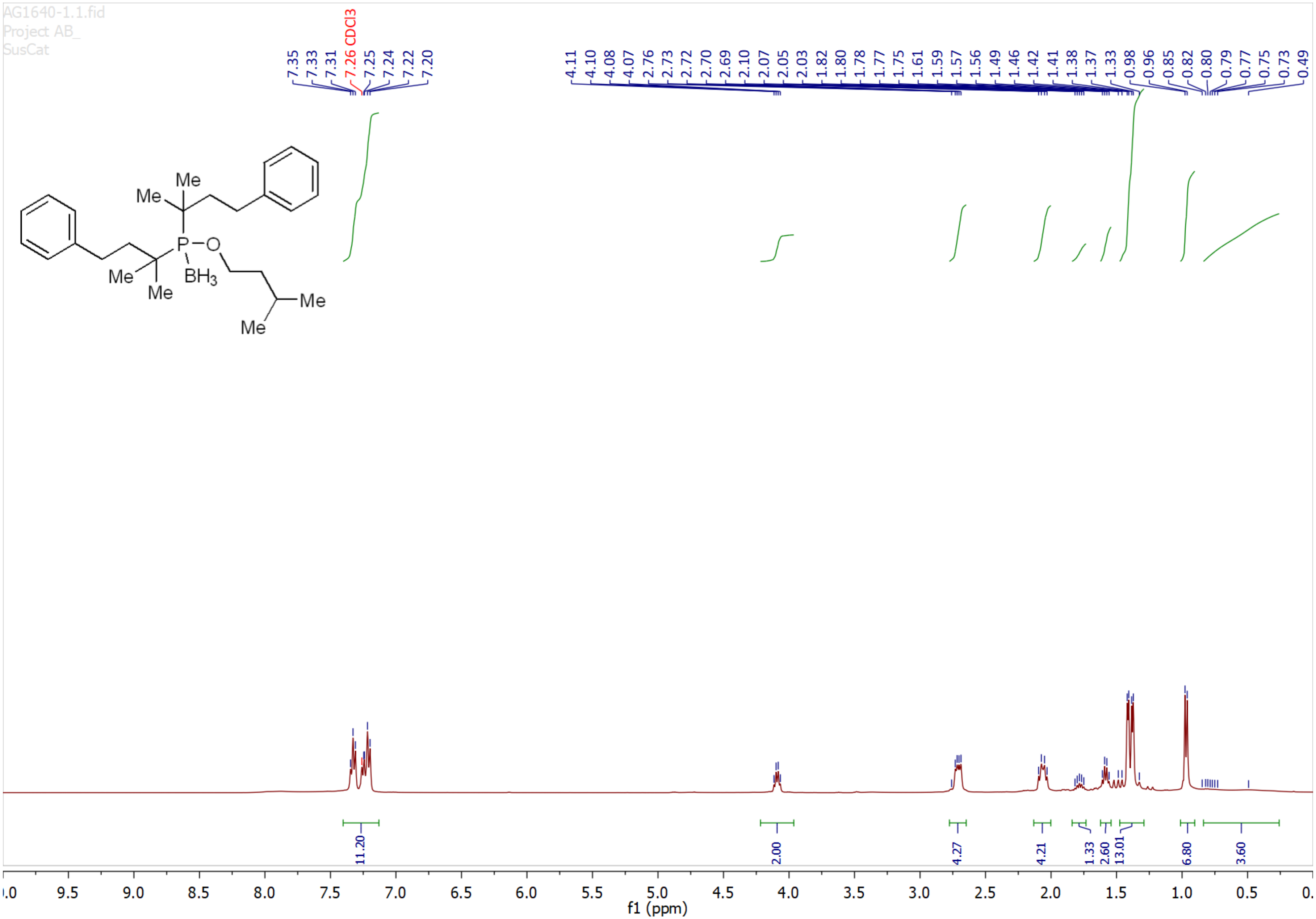

Spectrum S58. The ^{13}H NMR spectrum of *GreenPhos 11* in $CDCl_3$.

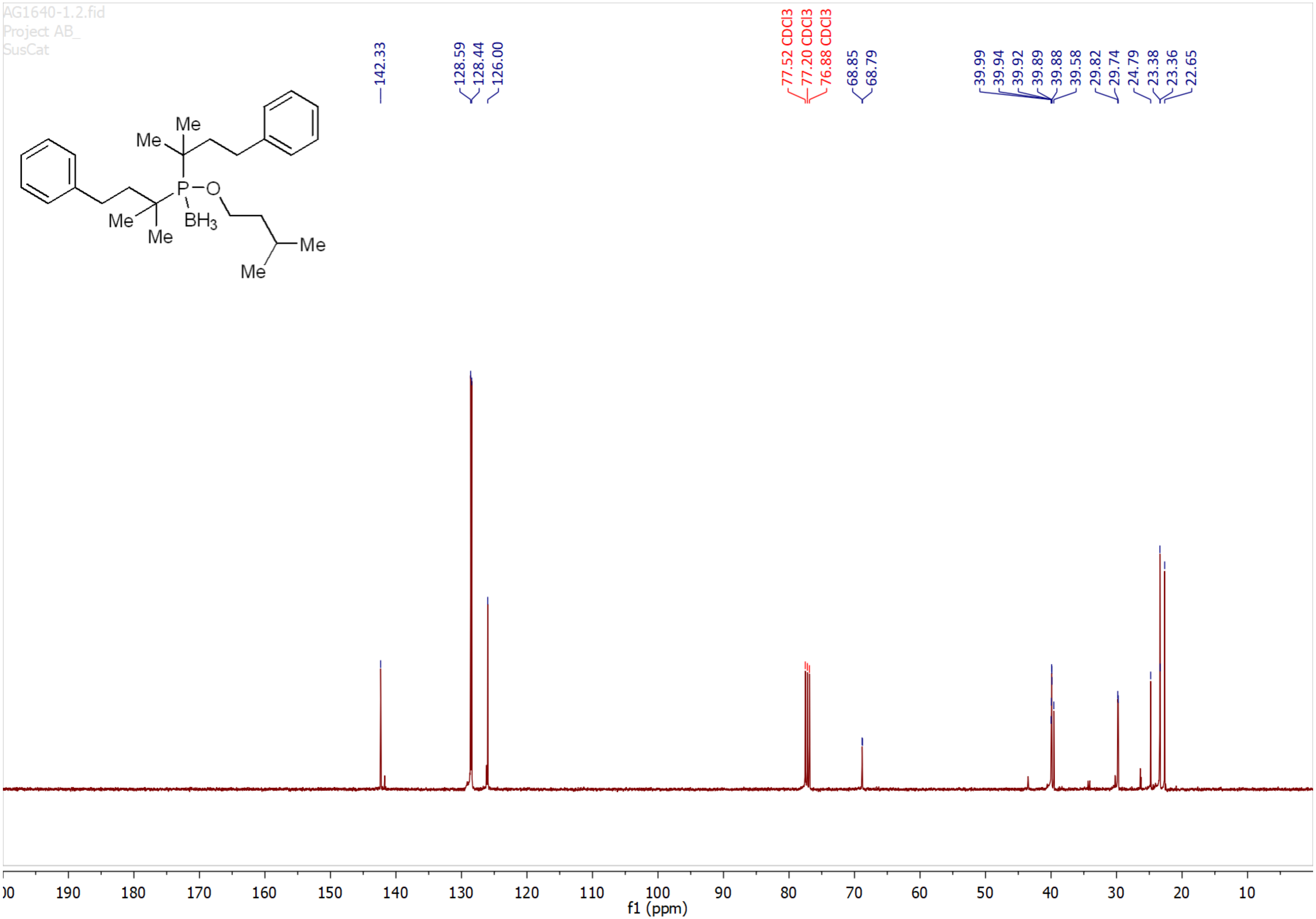

Spectrum S59. The ^{31}P NMR spectrum of *GreenPhos 11* with composite pulse decoupling in $CDCl_3$.

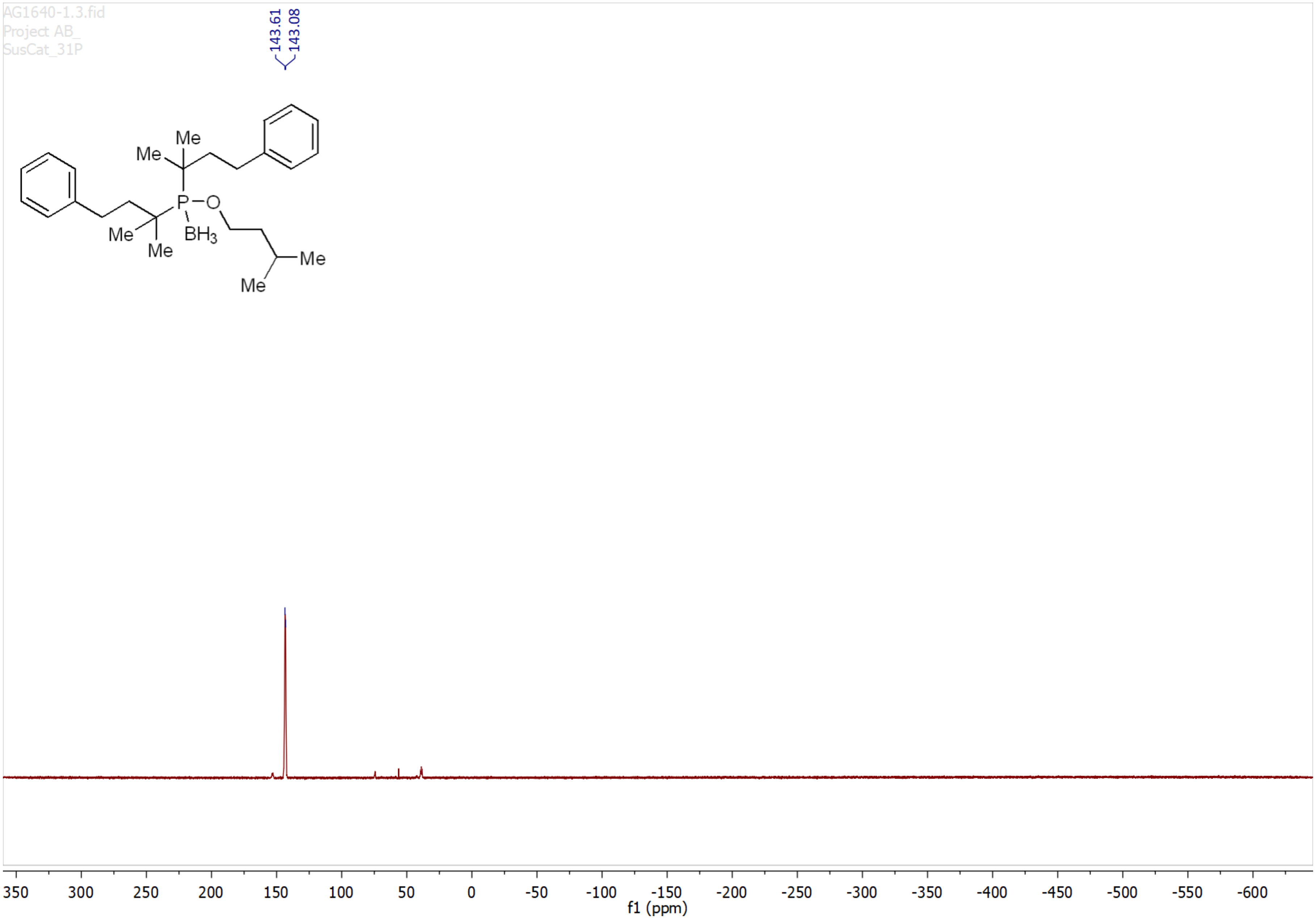

Spectrum S60. The ^{31}P NMR spectrum of *GreenPhos 11* with no decoupling in $CDCl_3$.

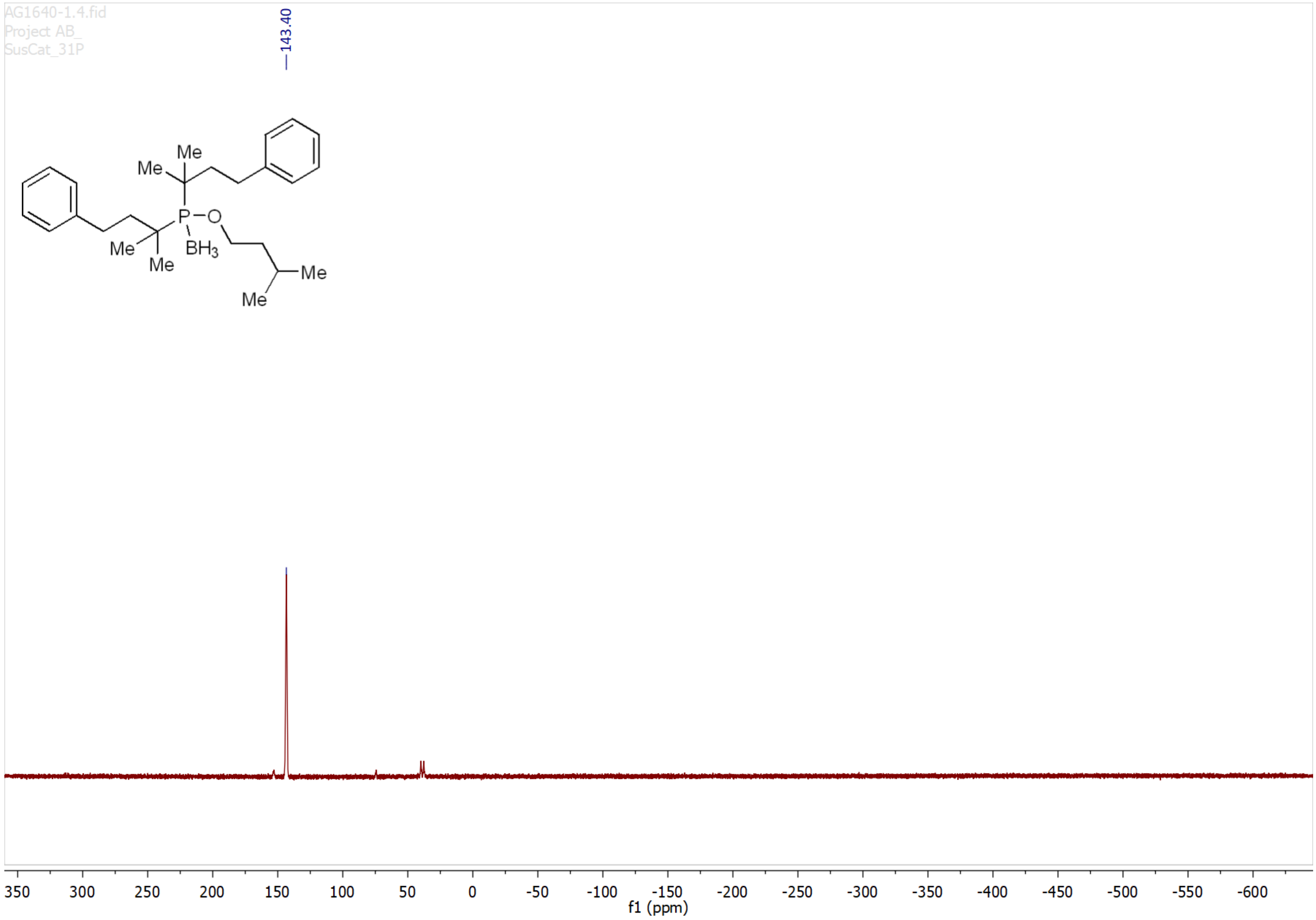

Spectrum S61. The ^{1}H NMR spectrum of *GreenPhos 12* in $CDCl_3$.

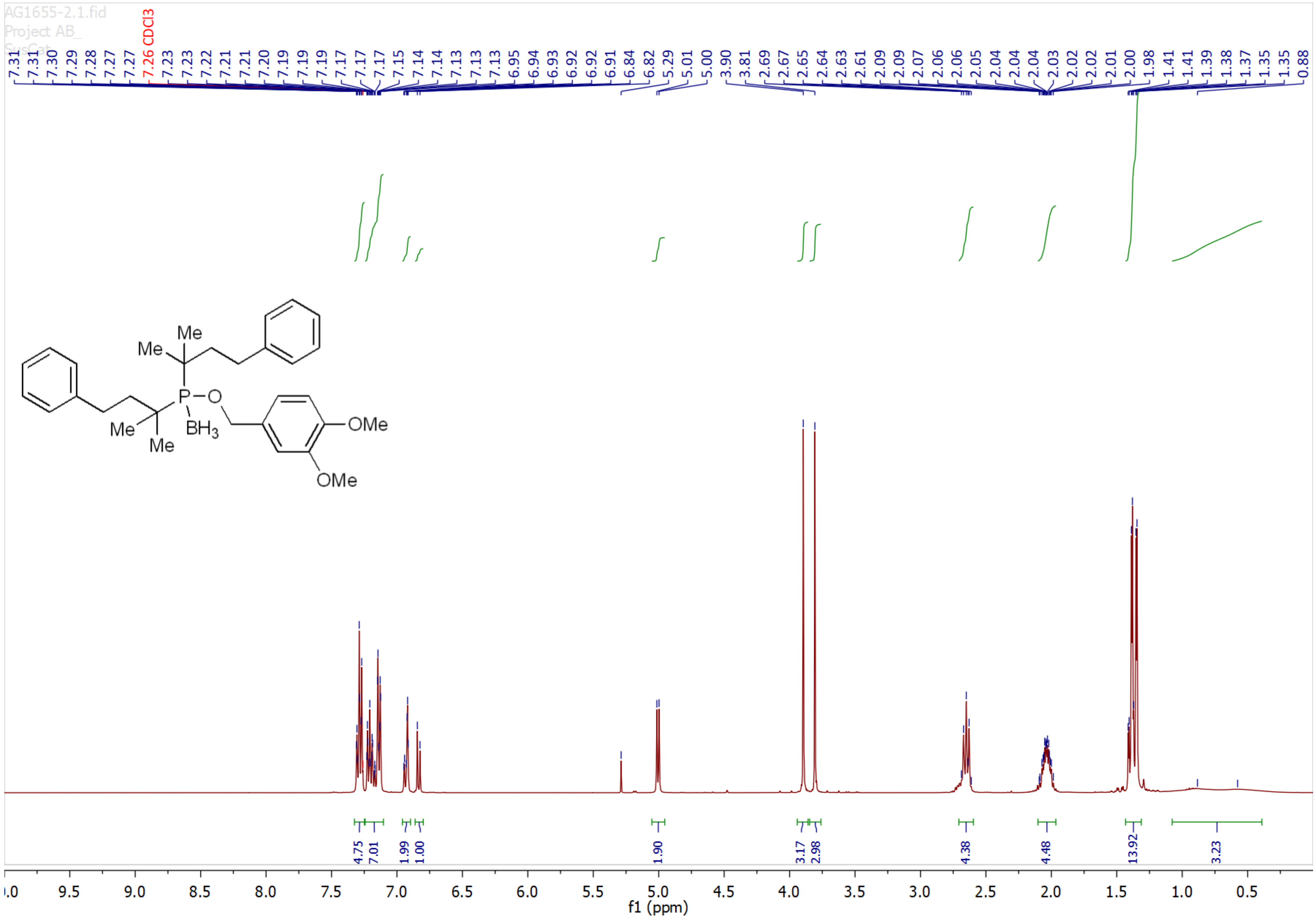

Spectrum S62. The ^{13}C NMR spectrum of *GreenPhos 12* in $CDCl_3$.

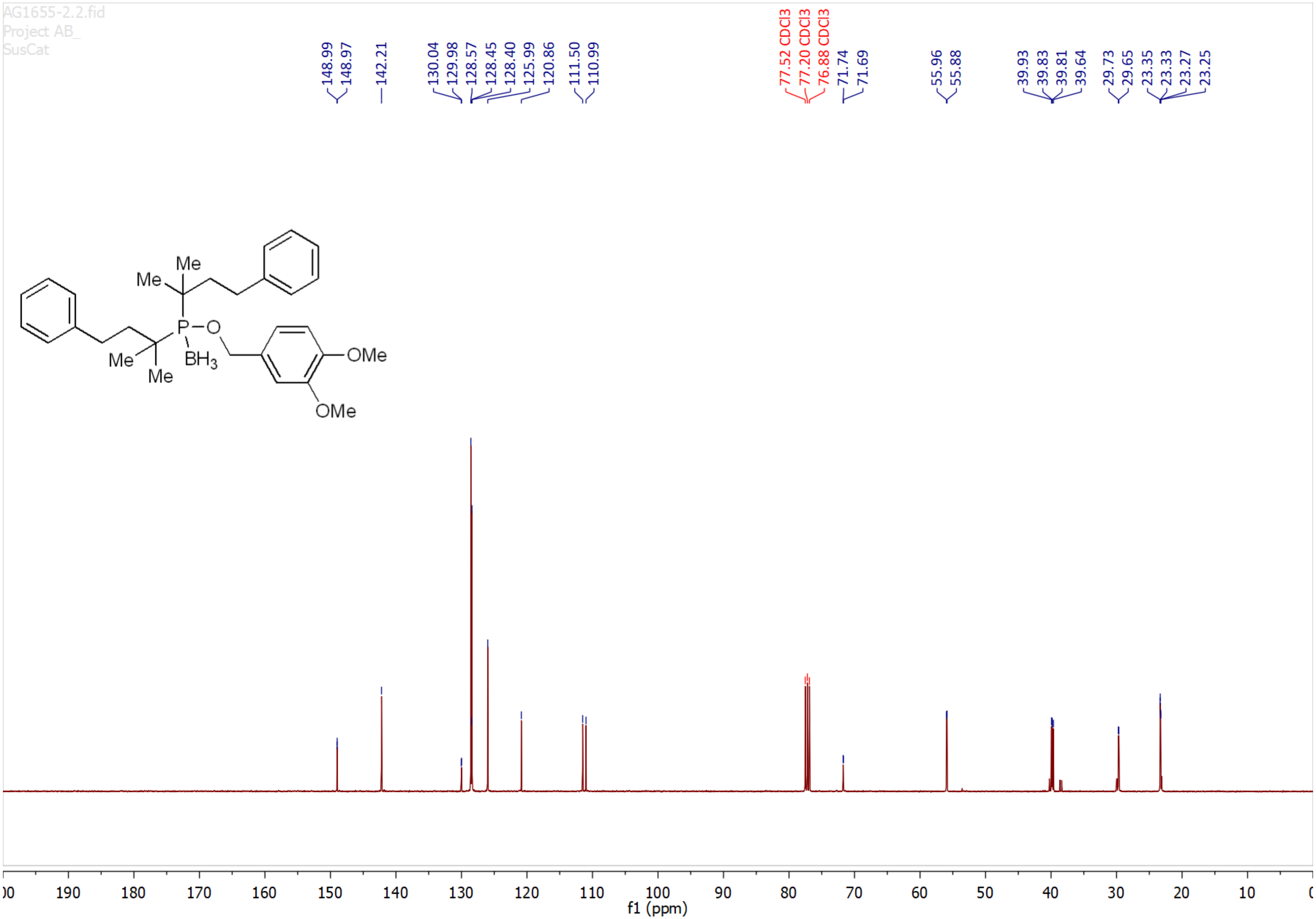

Spectrum S63. The ^{31}P NMR spectrum of *GreenPhos 12* with composite pulse decoupling in $CDCl_3$.

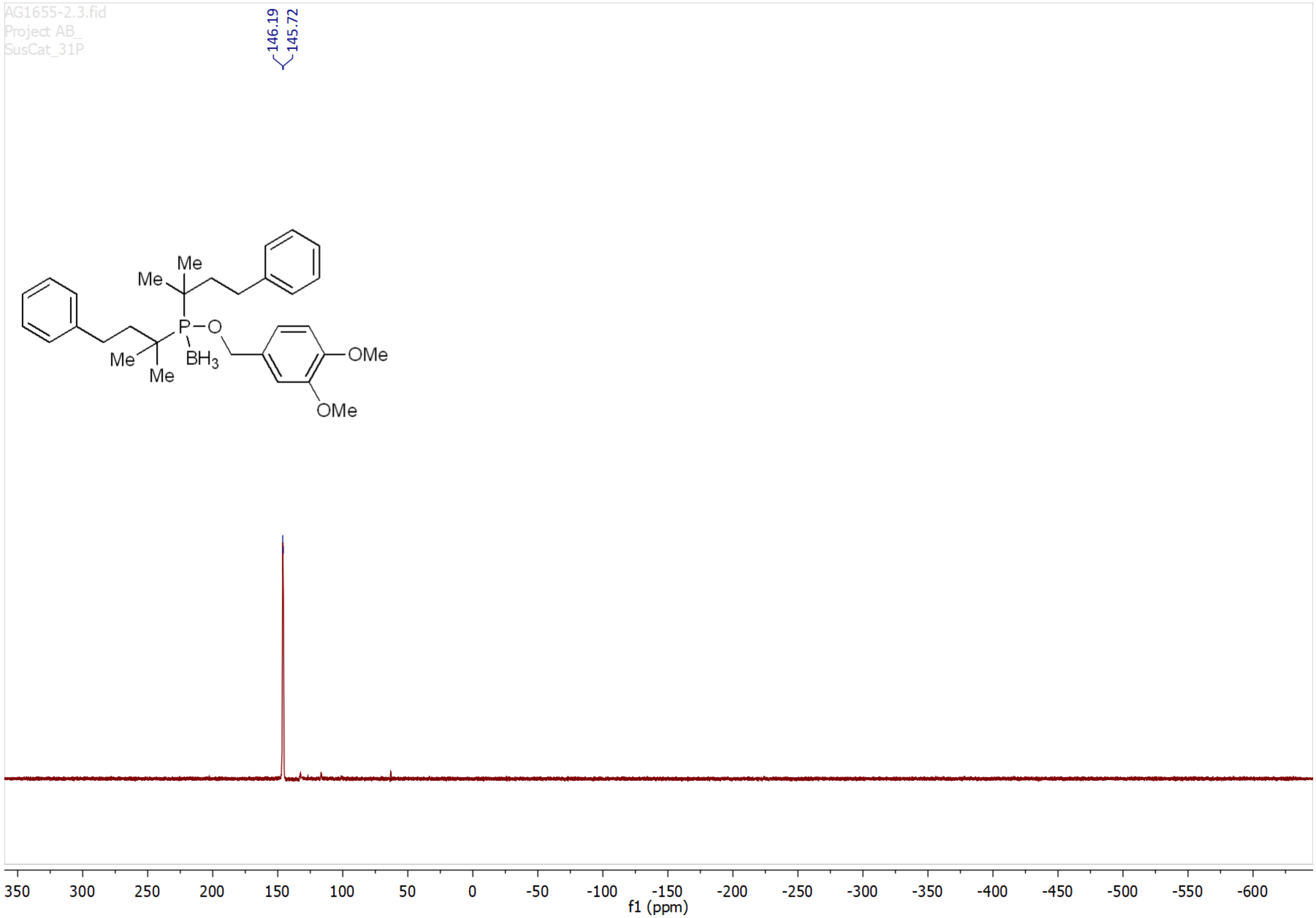

Spectrum S64. The ^{31}P NMR spectrum of *GreenPhos 12* with no decoupling in $CDCl_3$.

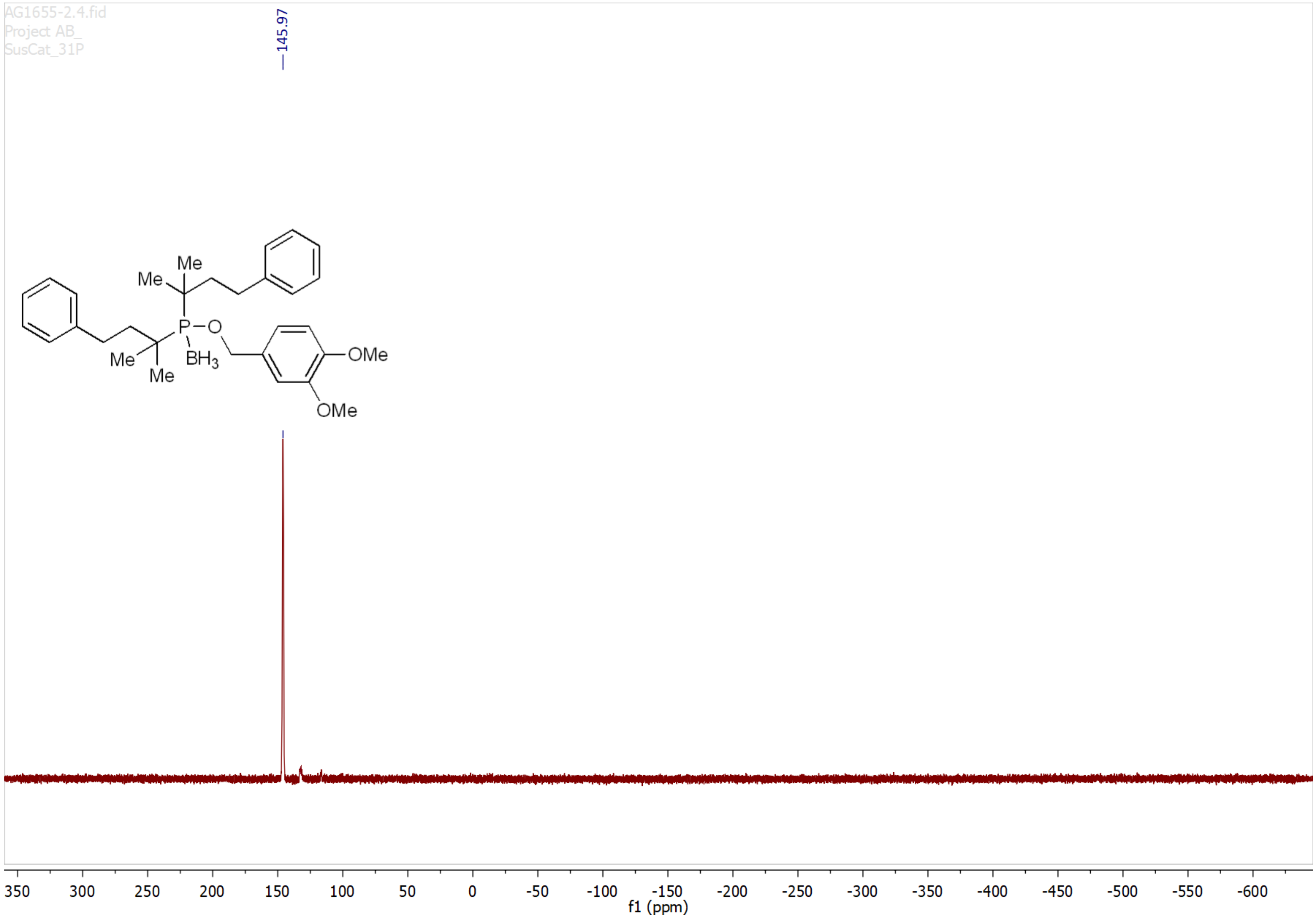

Spectrum S65. The ^{1}H NMR spectrum of *GreenPhos 13* in $CDCl_3$.

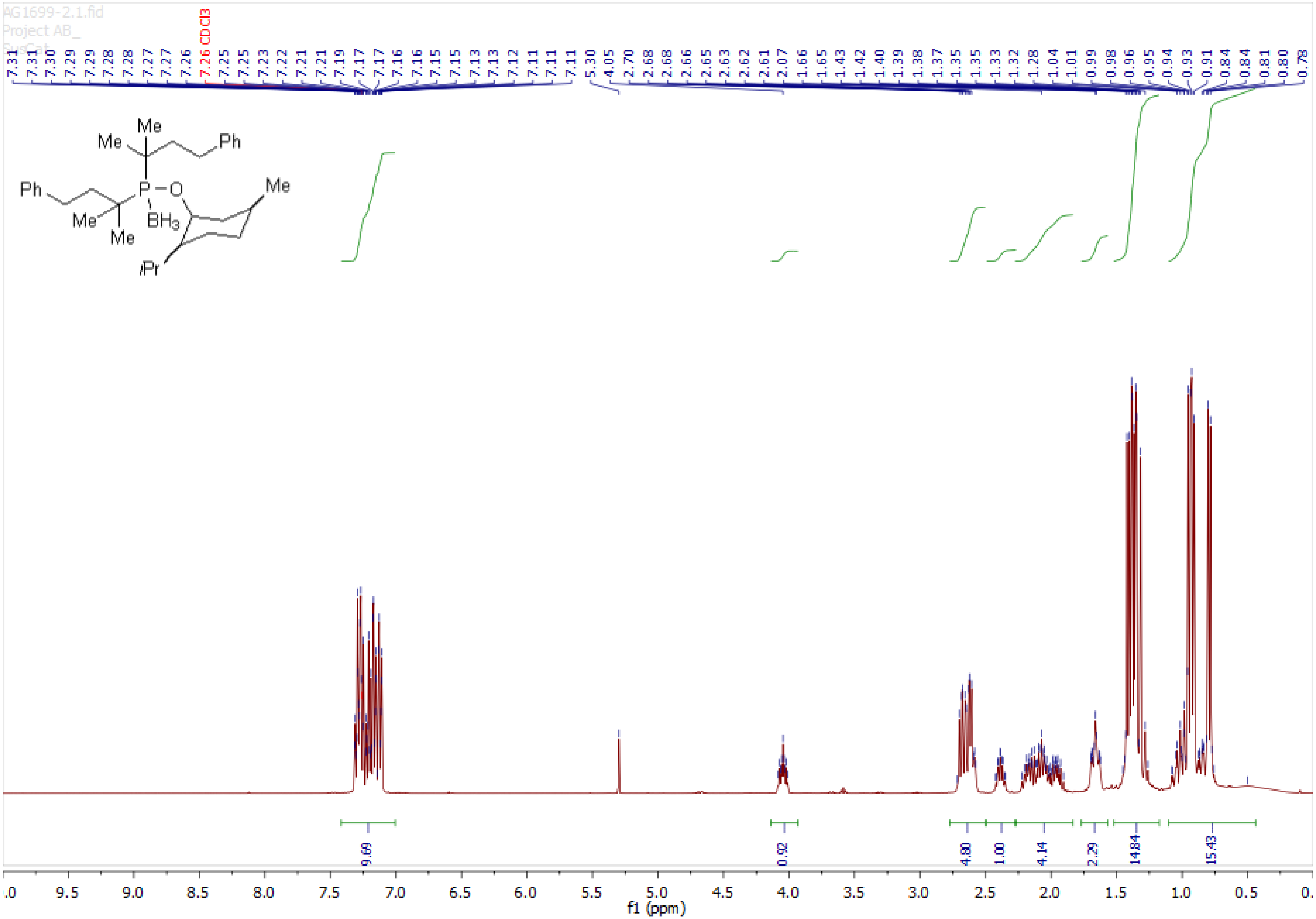

Spectrum S66. The ^{13}C NMR spectrum of *GreenPhos 13* in $CDCl_3$.

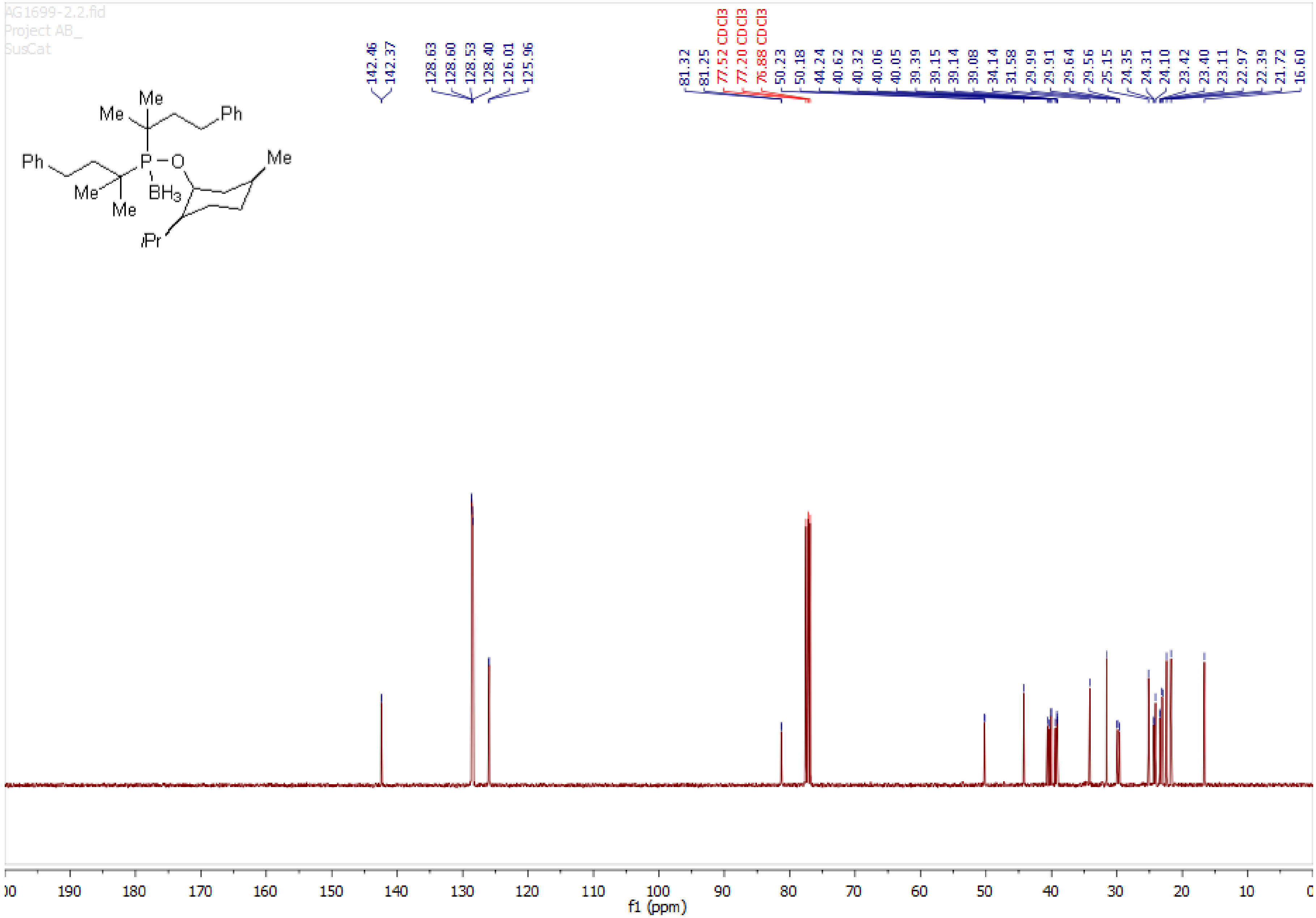

Spectrum S67. The ^{31}P NMR spectrum of *GreenPhos 13* with composite pulse decoupling in $CDCl_3$.

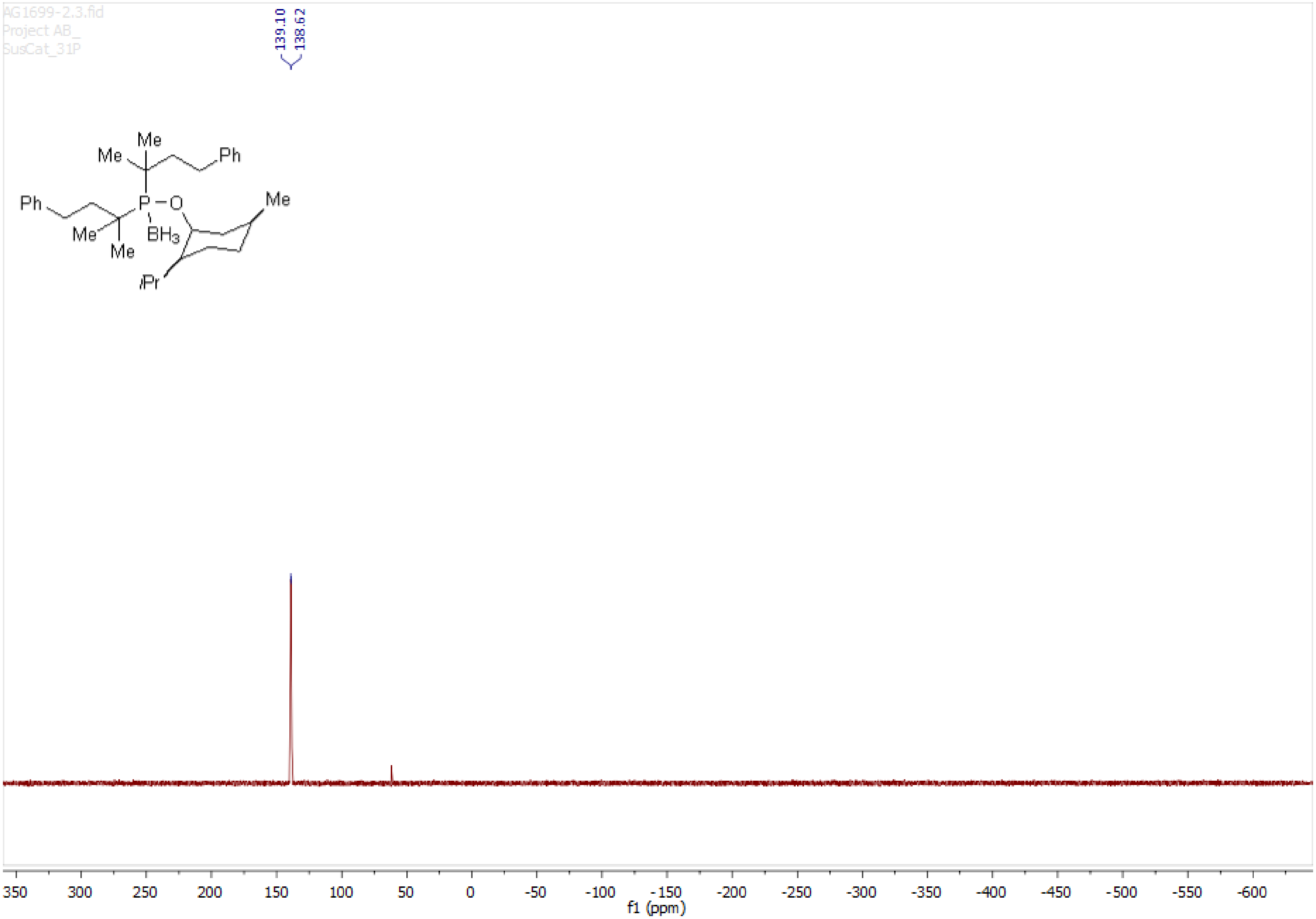

Spectrum S68. The ^{31}P NMR spectrum of *GreenPhos 13* with no decoupling in $CDCl_3$.

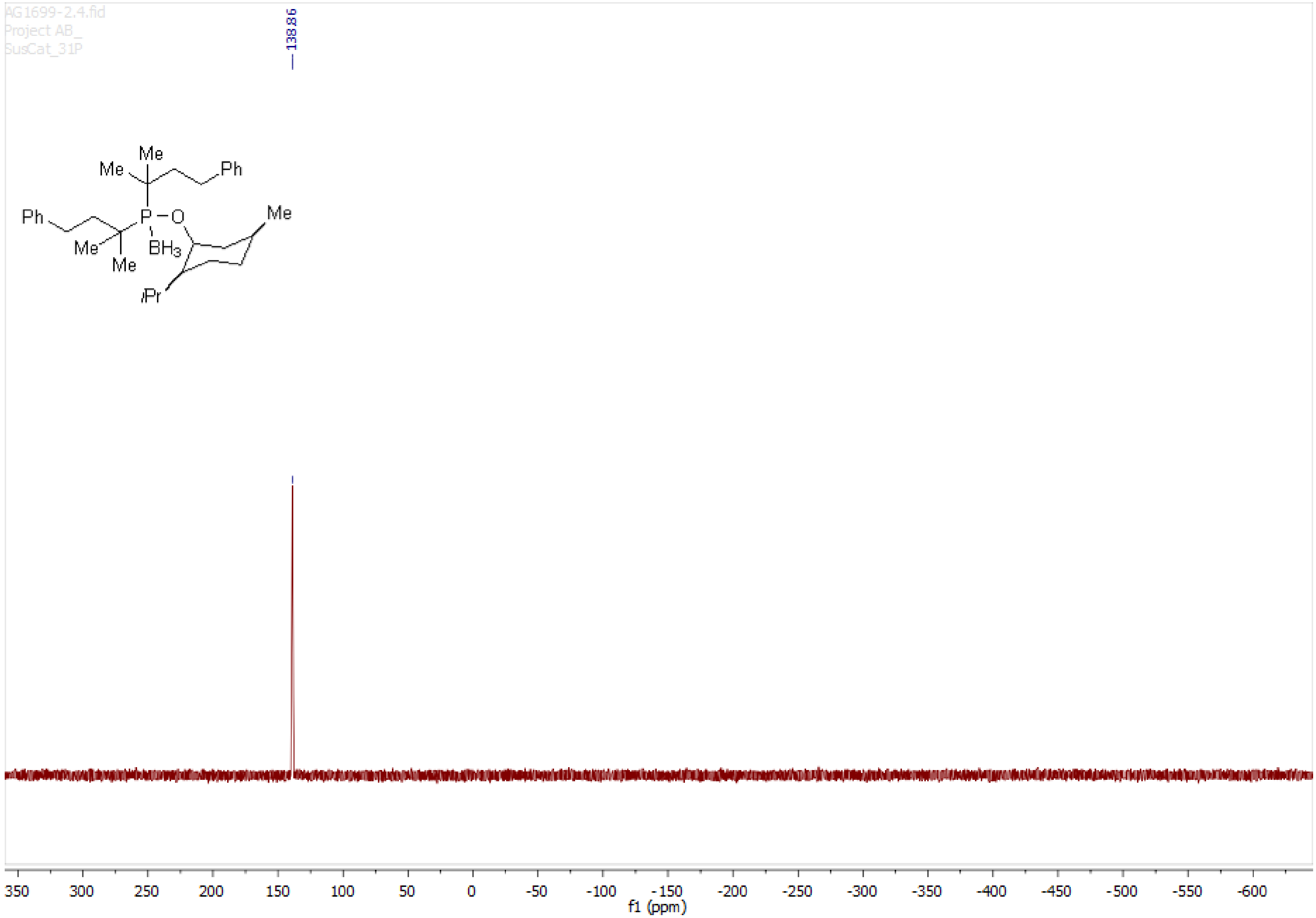

Spectrum S69. The ^{1}H NMR spectrum of *GreenPhos 14* in $CDCl_3$.

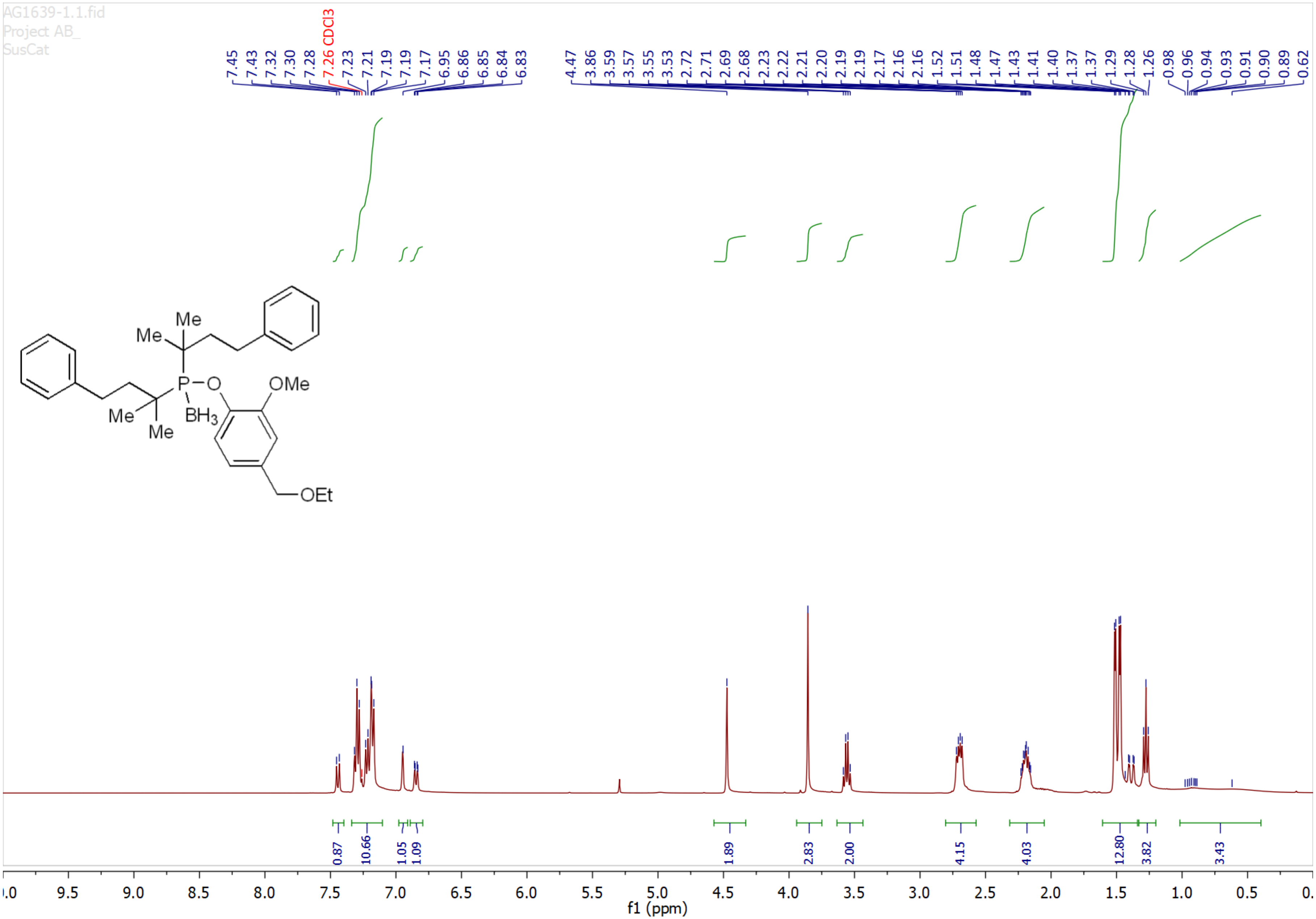

Spectrum S70. The ^{13}C NMR spectrum of *GreenPhos 14* in $CDCl_3$.

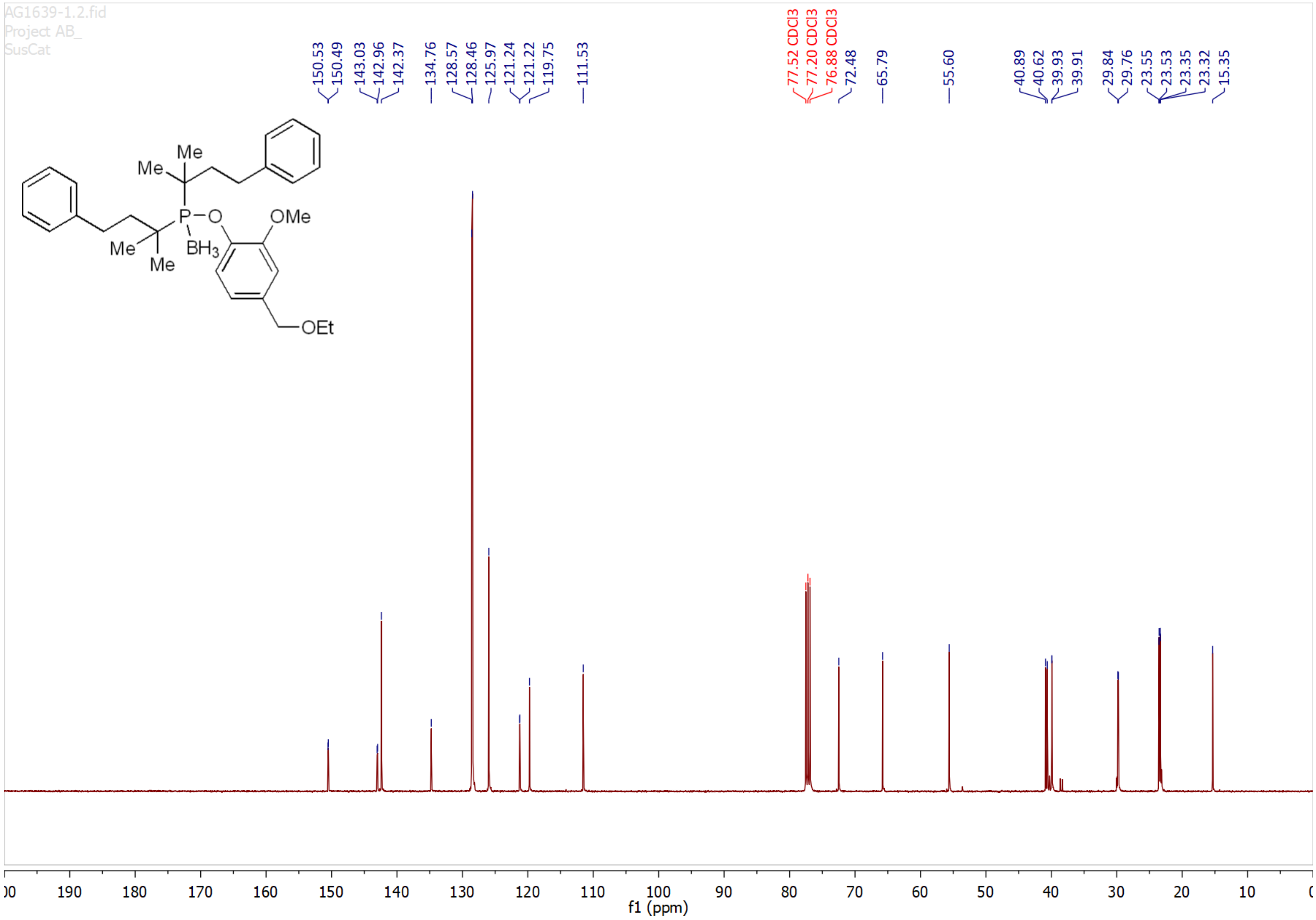

Spectrum S71. The ^{31}P NMR spectrum of *GreenPhos 14* with composite pulse decoupling in $CDCl_3$.

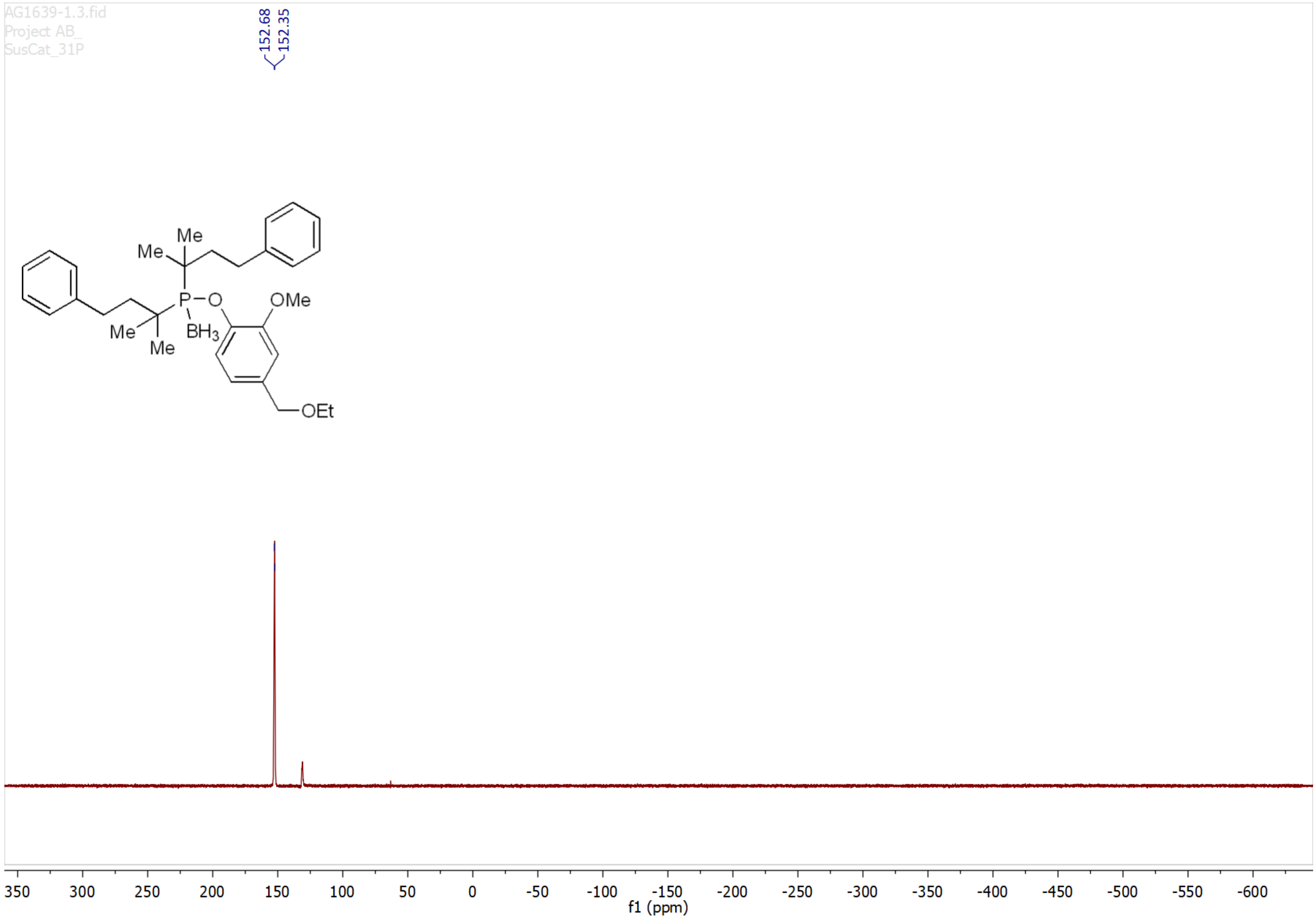

Spectrum S72. The ^{31}P NMR spectrum of *GreenPhos 14* with no decoupling in $CDCl_3$.

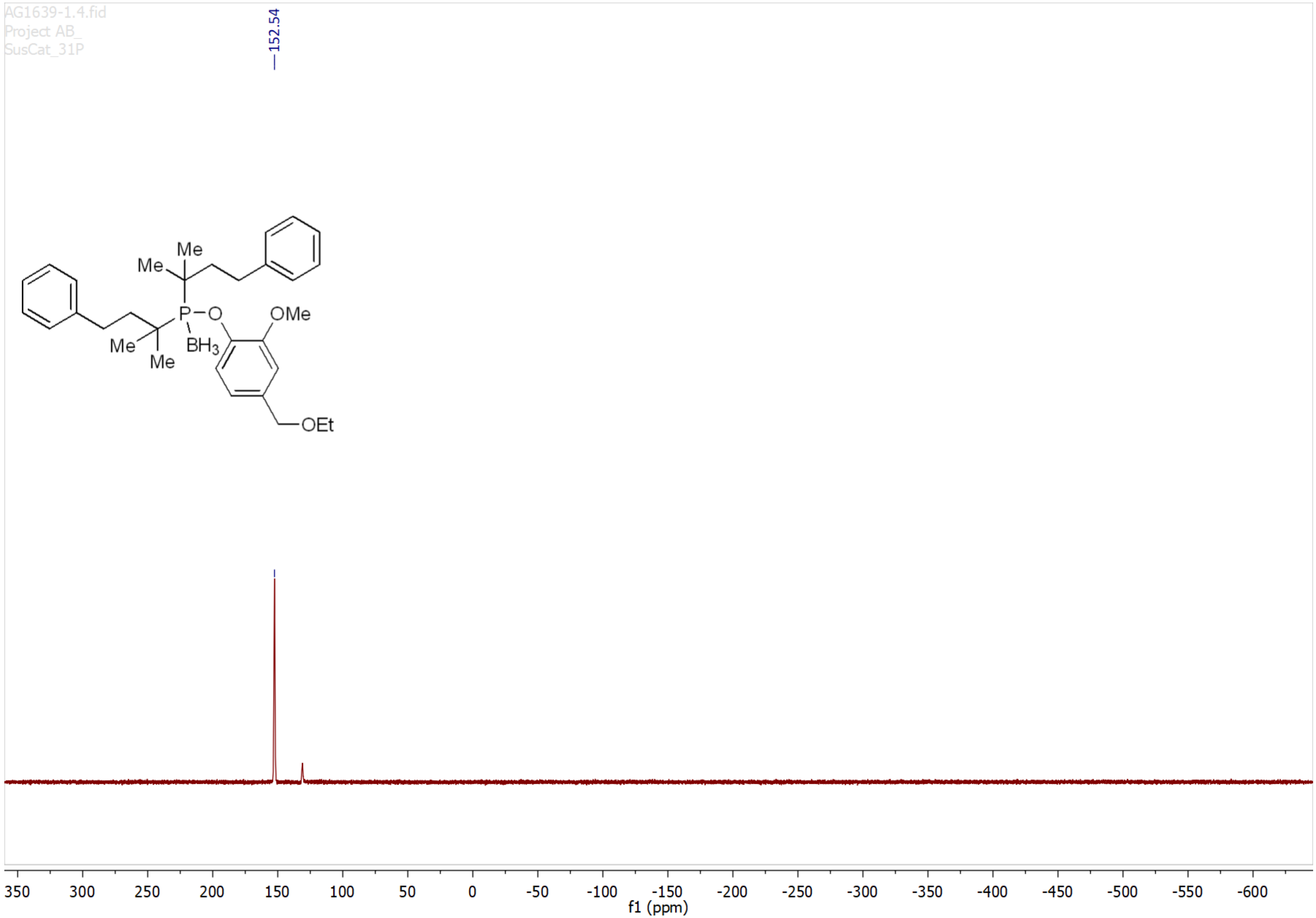

Spectrum S73. The ^{1}H NMR spectrum of *GreenPhos 15* in $CDCl_3$.

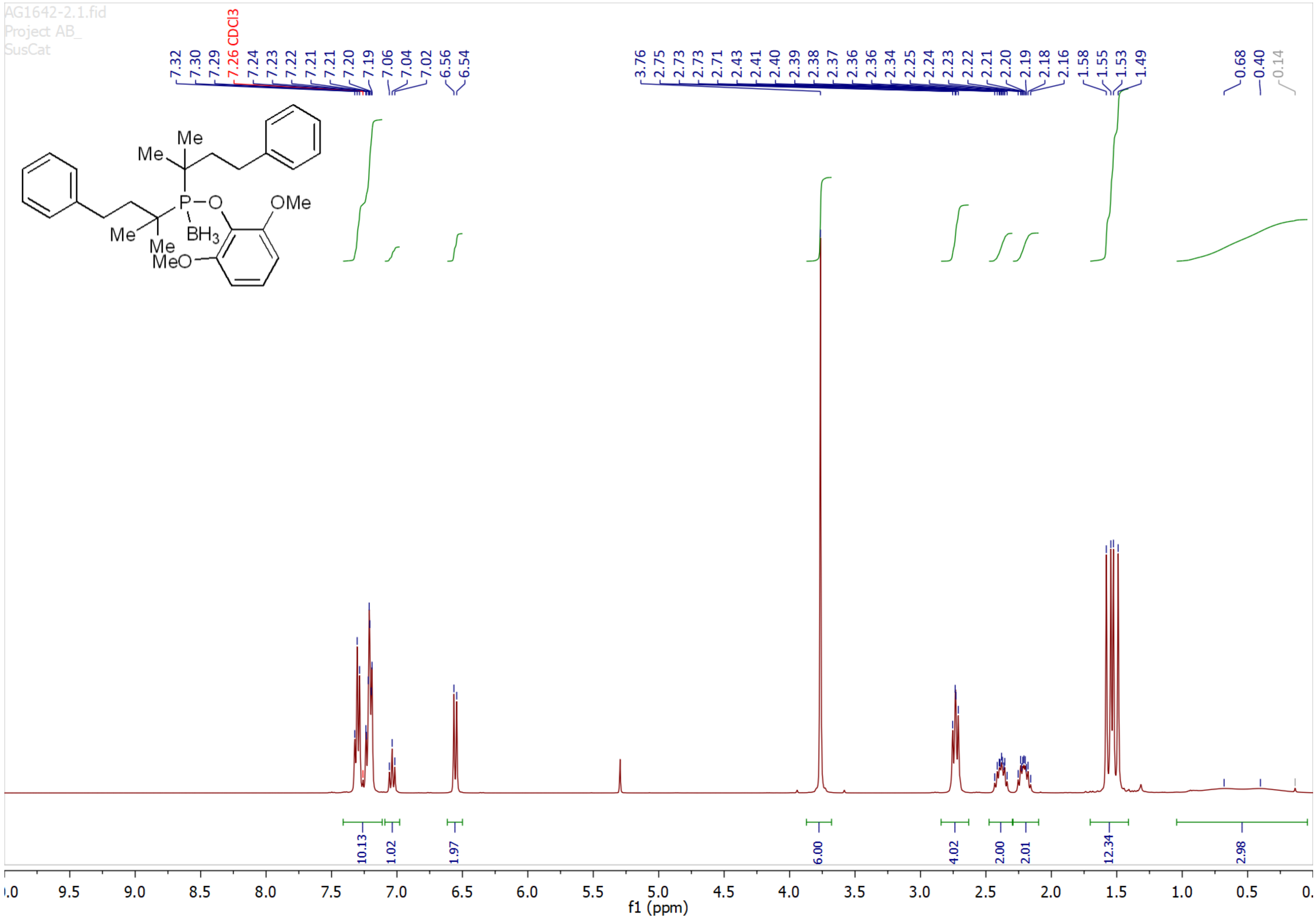

Spectrum S74. The ^{13}C NMR spectrum of *GreenPhos 15* in $CDCl_3$.

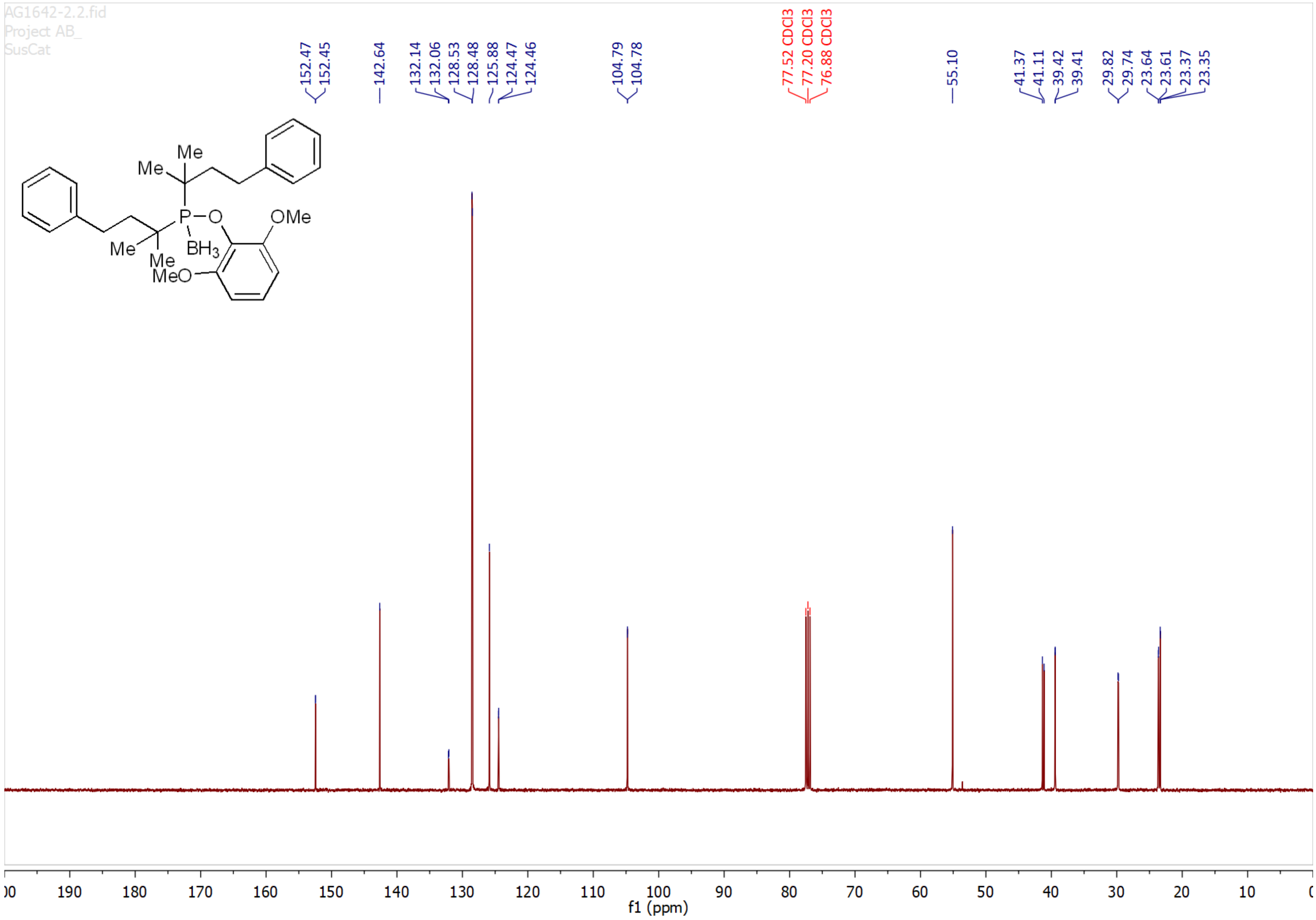

Spectrum S75. The ^{31}P NMR spectrum of *GreenPhos 15* with composite pulse decoupling in $CDCl_3$.

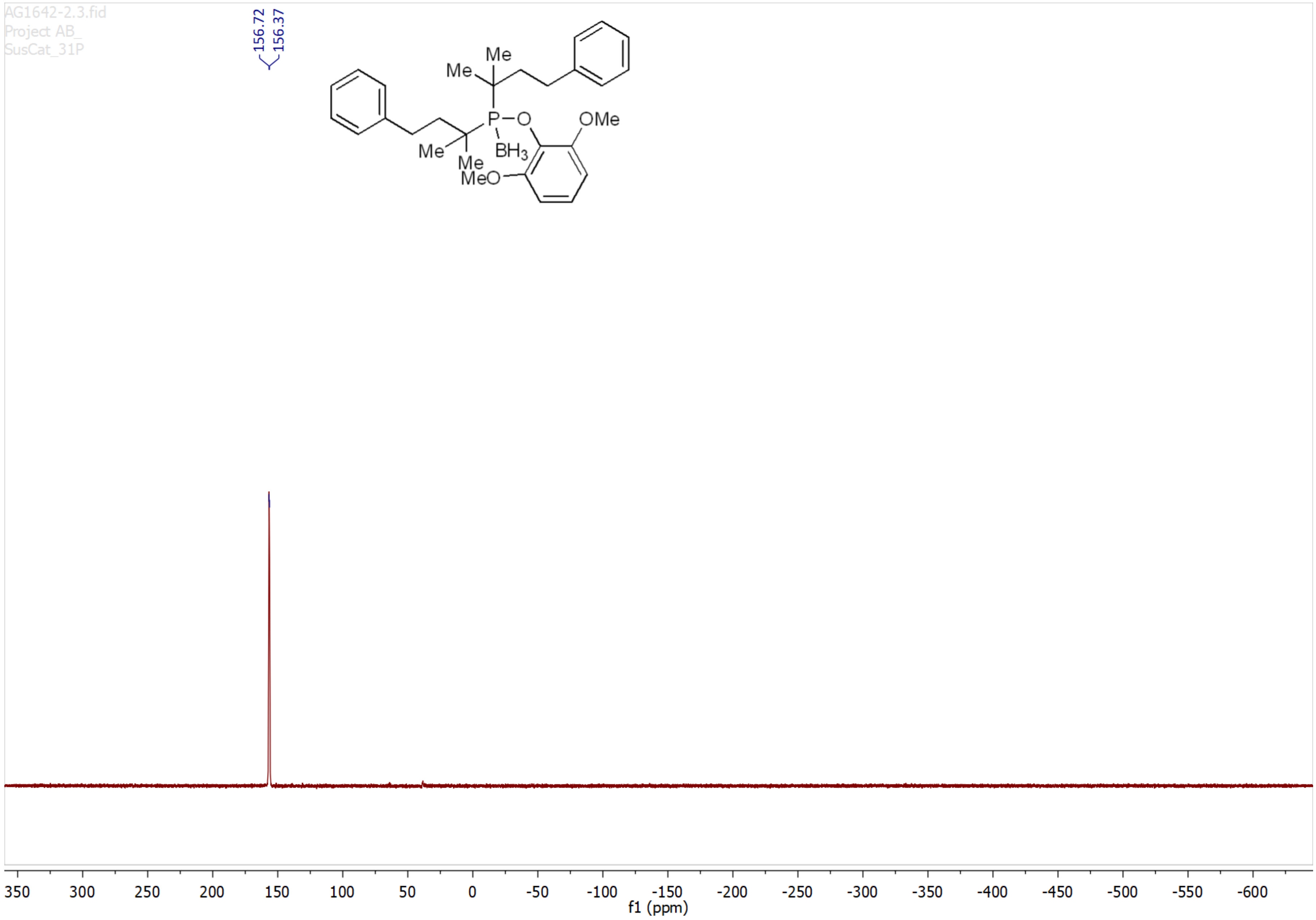

Spectrum S76. The ^{31}P NMR spectrum of *GreenPhos 15* with no decoupling in $CDCl_3$.

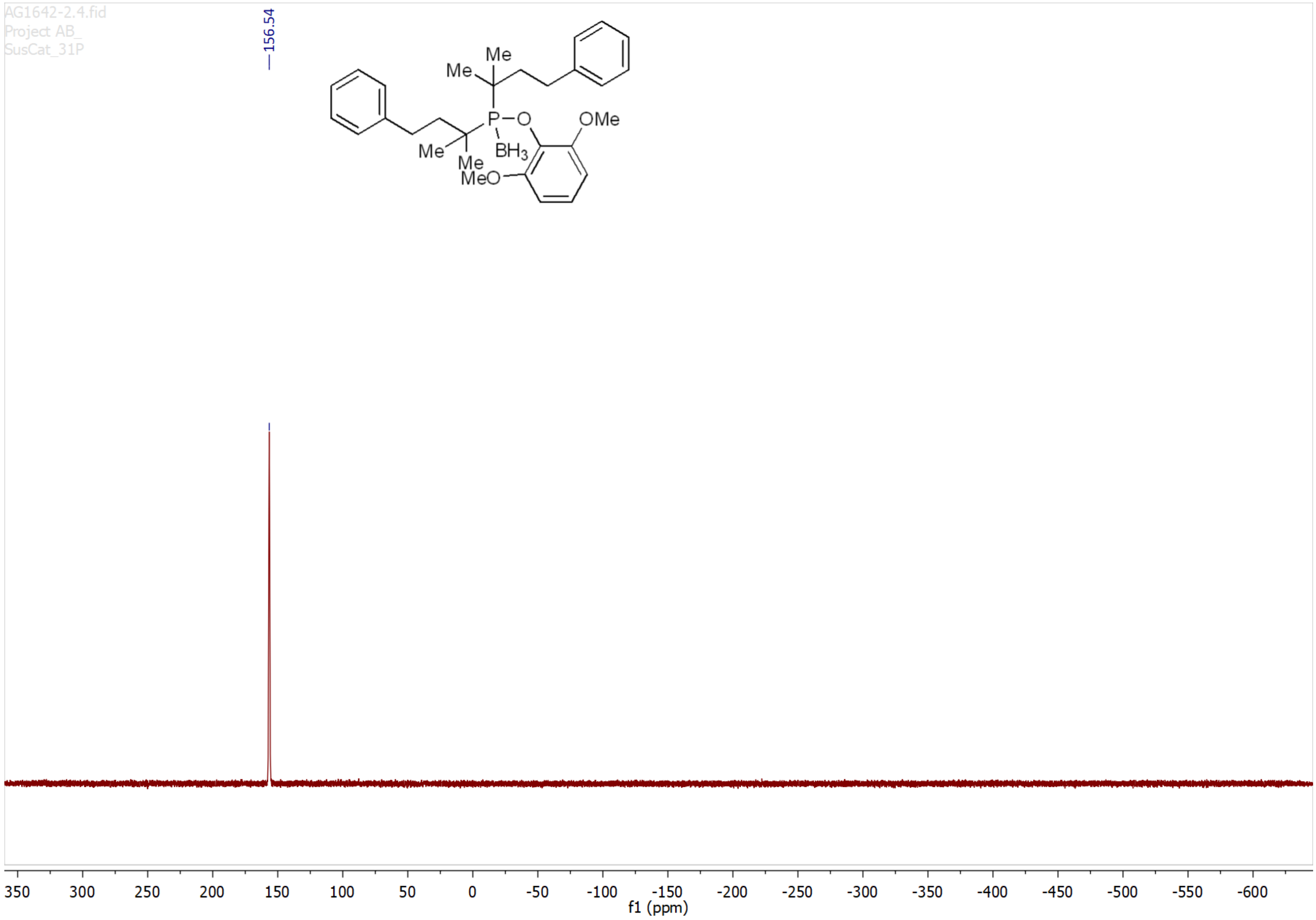

Spectrum S77. The ^{1}H NMR spectrum of *GreenPhos 16* in $CDCl_3$.

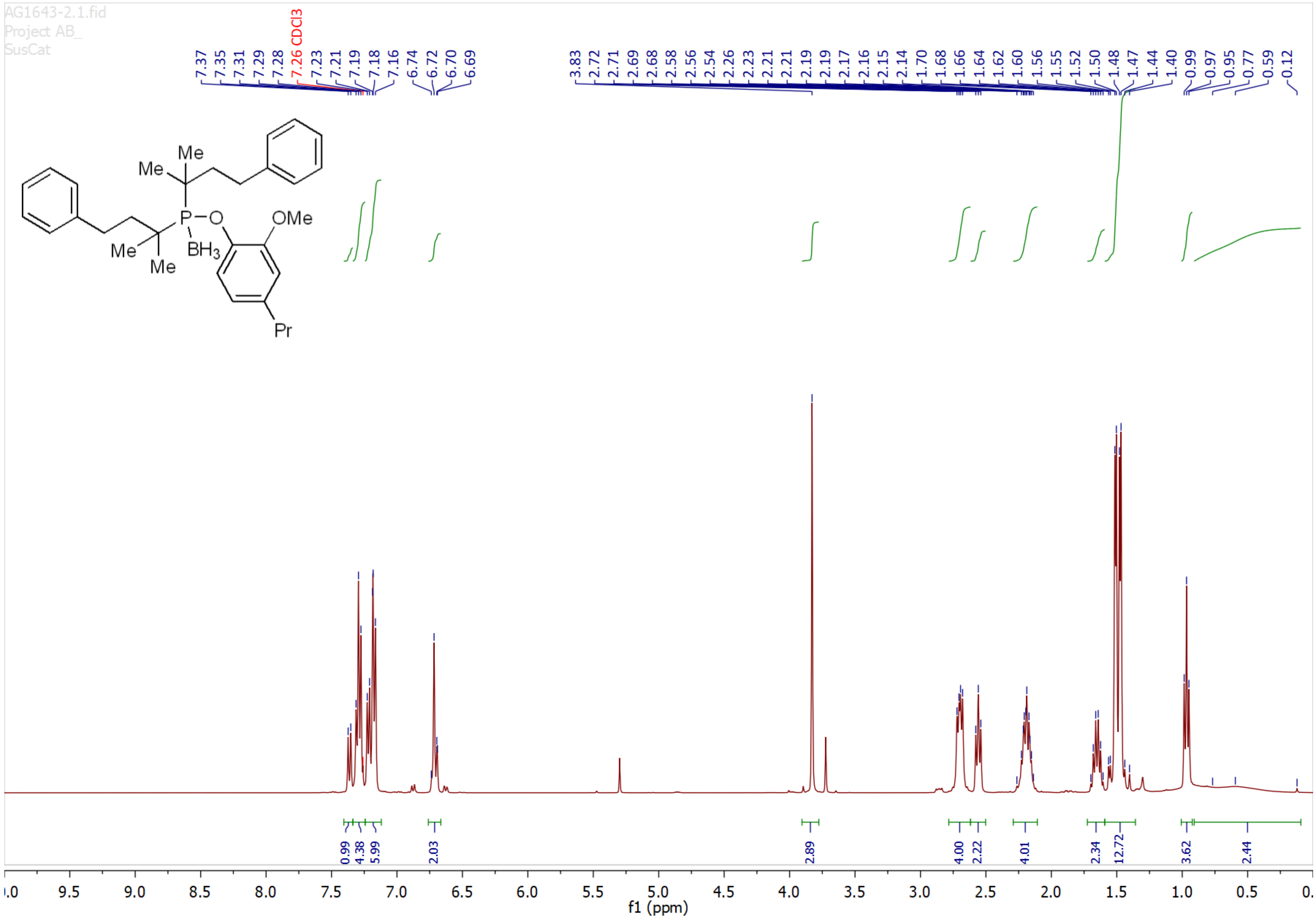

Spectrum S78. The ^{13}C NMR spectrum of *GreenPhos 16* in $CDCl_3$.

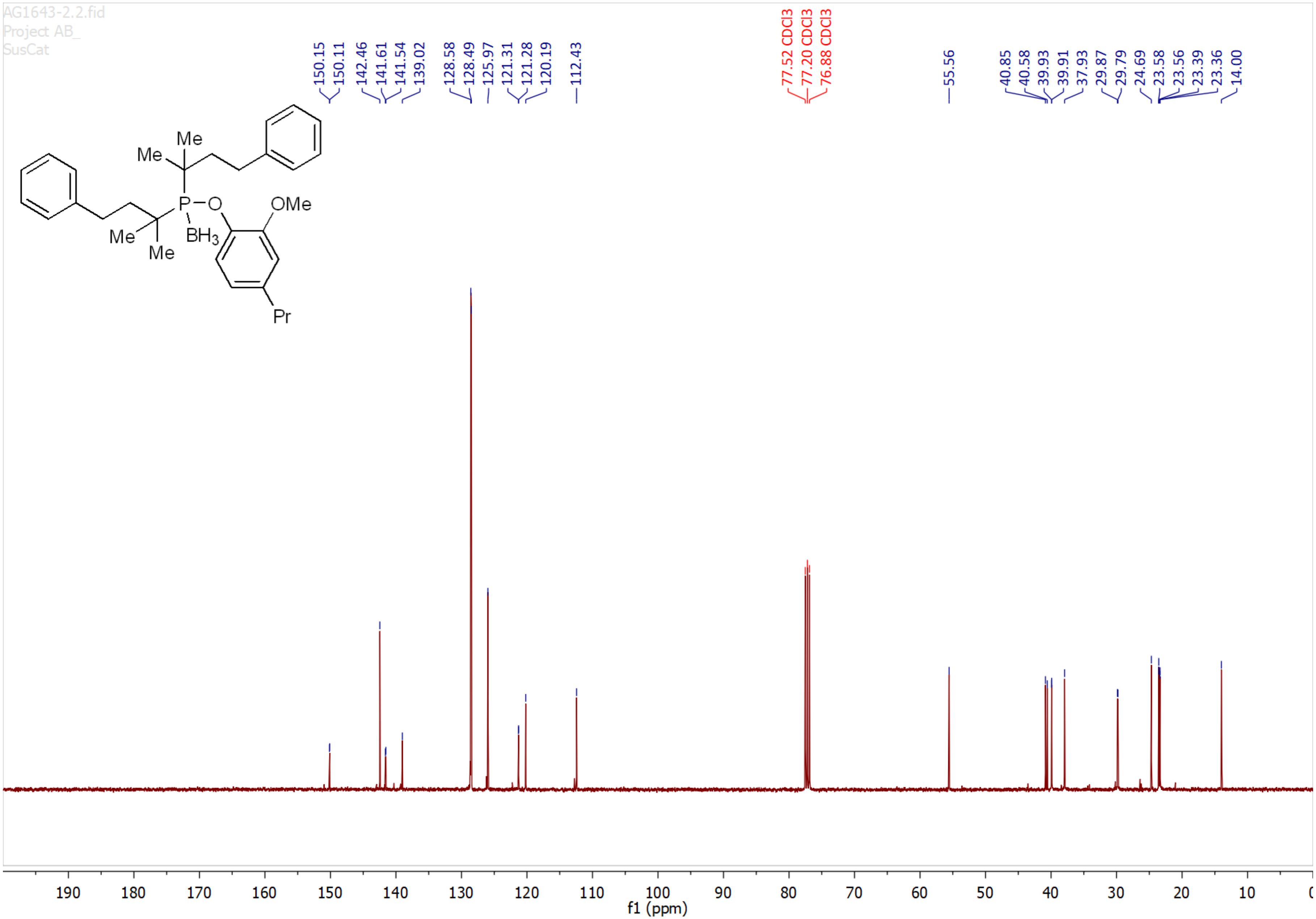

Spectrum S79. The ^{31}P NMR spectrum of *GreenPhos 16* with composite pulse decoupling in $CDCl_3$.

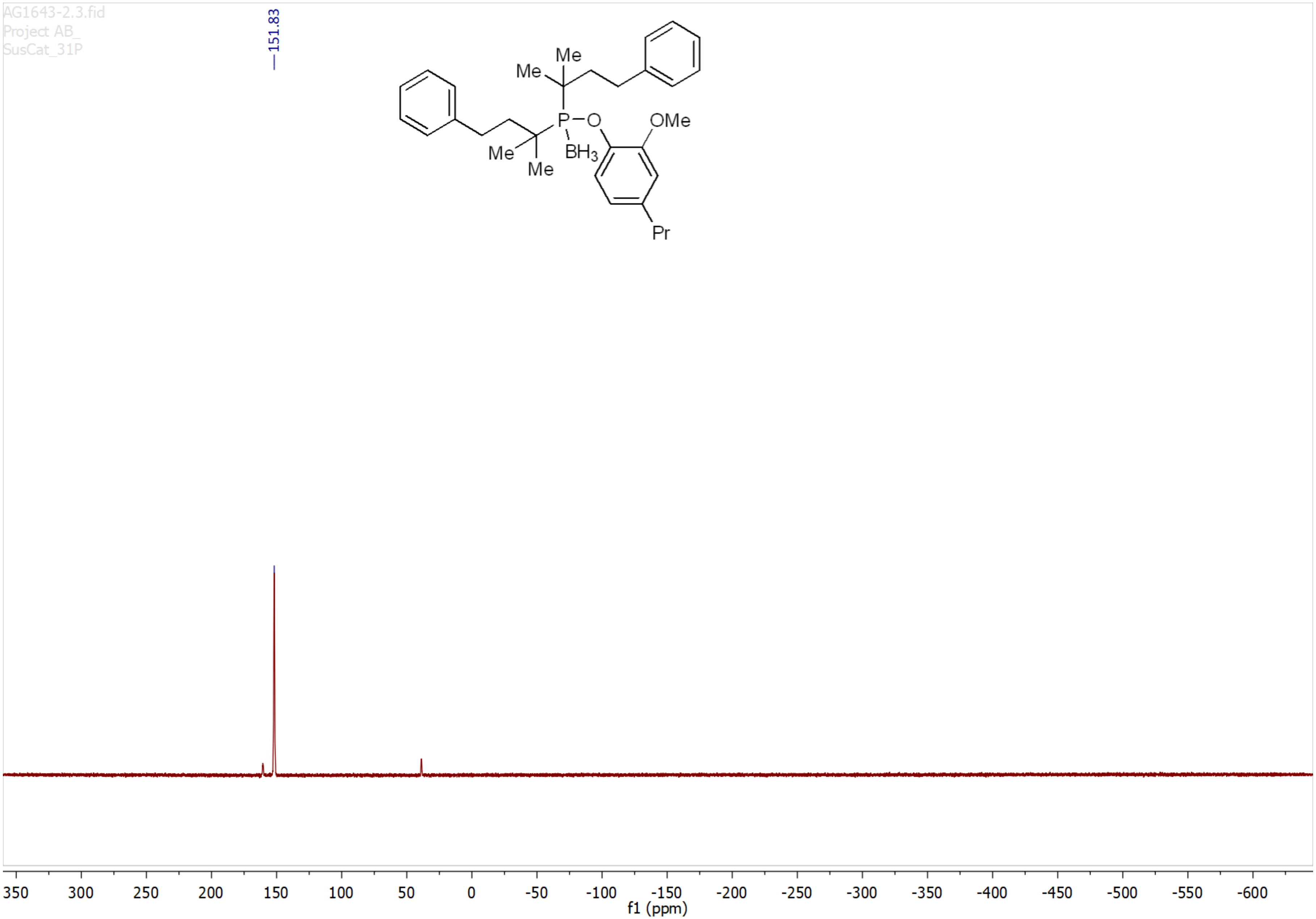

Spectrum S80. The ^{31}P NMR spectrum of *GreenPhos 16* with no decoupling in $CDCl_3$.

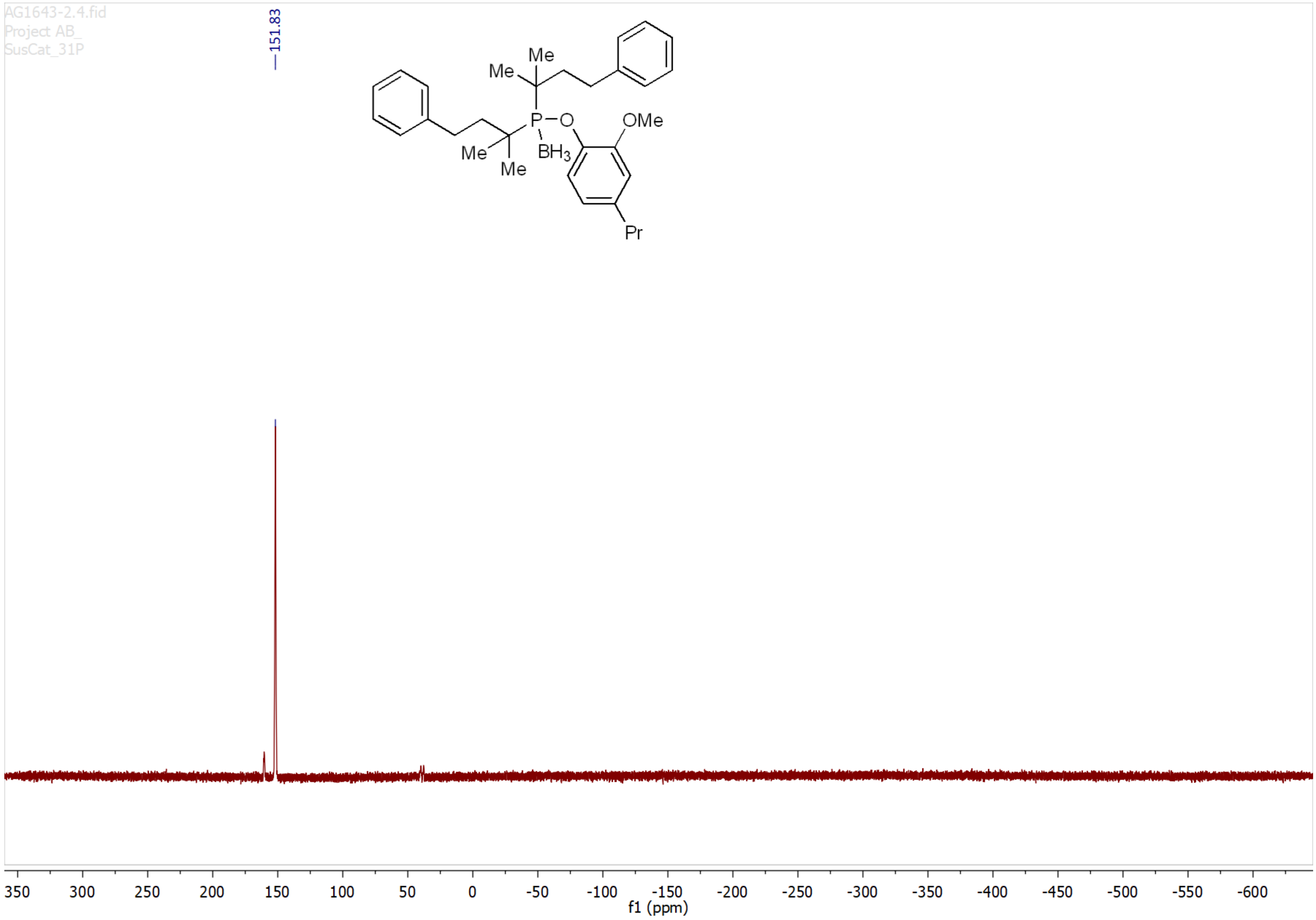

Spectrum S81. The ^{1}H NMR spectrum of *GreenPhos 17* in $CDCl_3$.

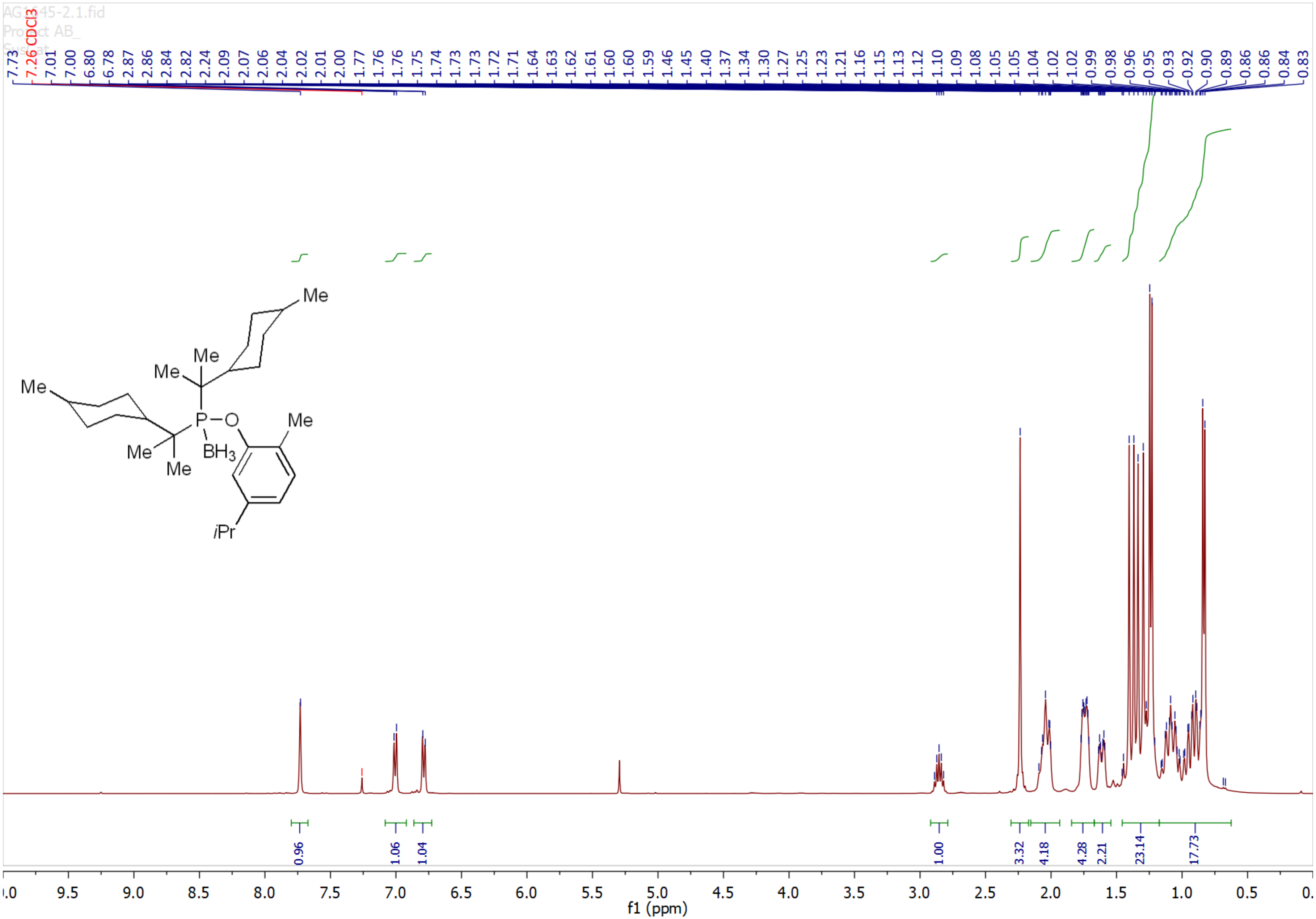

Spectrum S82. The ^{13}C NMR spectrum of *GreenPhos 17* in $CDCl_3$.

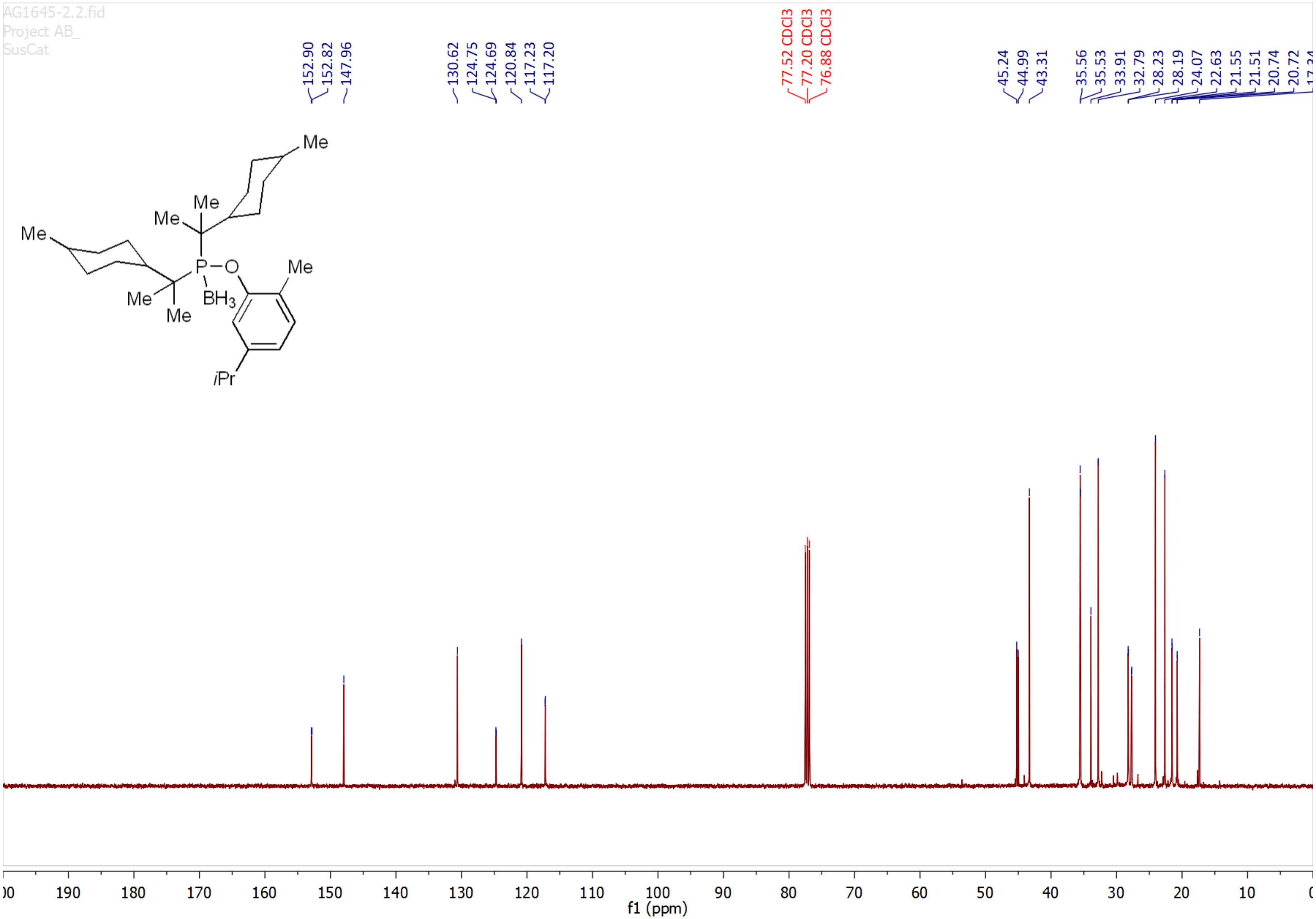

Spectrum S83. The ^{31}P NMR spectrum of *GreenPhos 17* with composite pulse decoupling in $CDCl_3$.

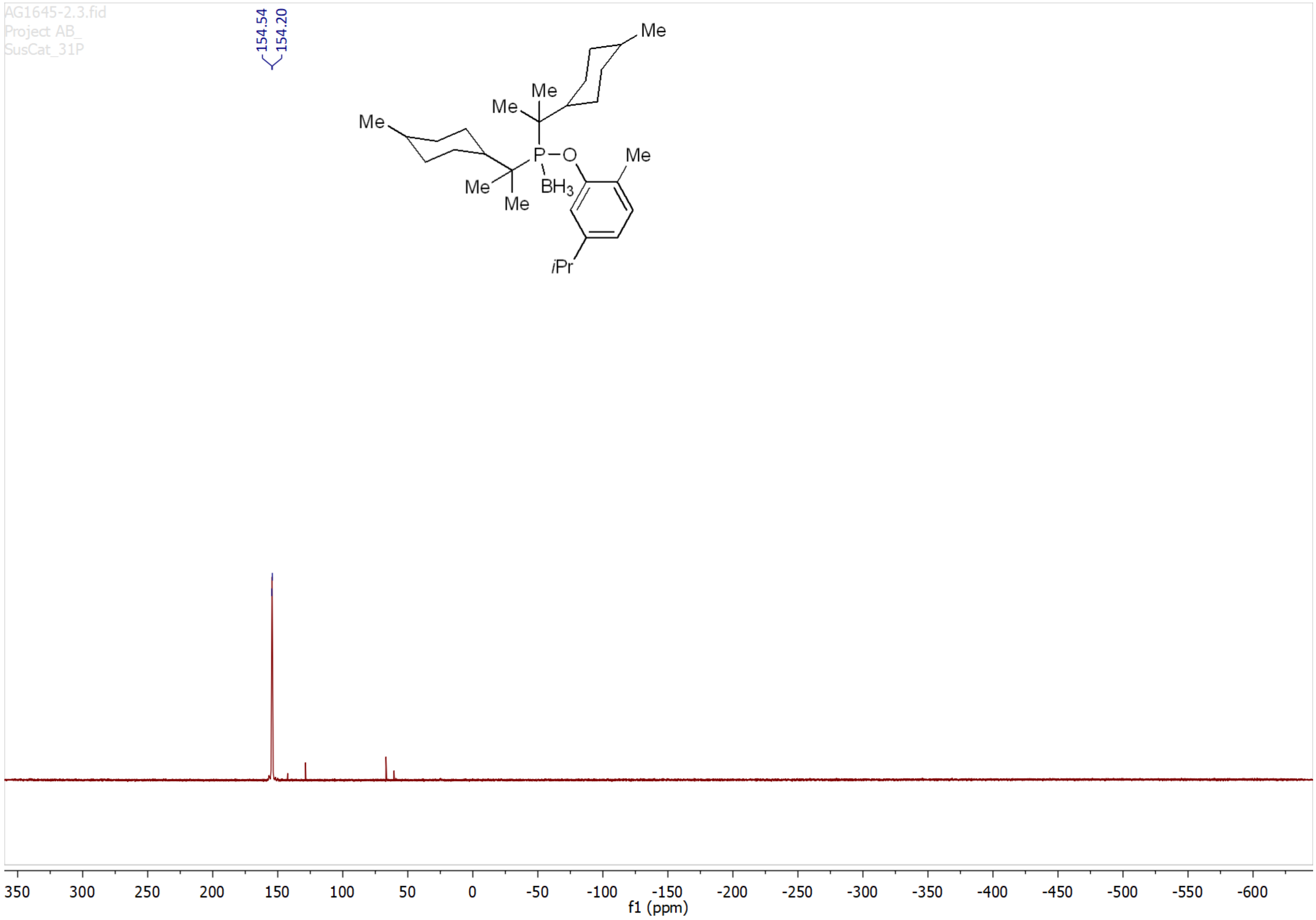

Spectrum S84. The ^{31}P NMR spectrum of *GreenPhos 17* with no decoupling in $CDCl_3$.

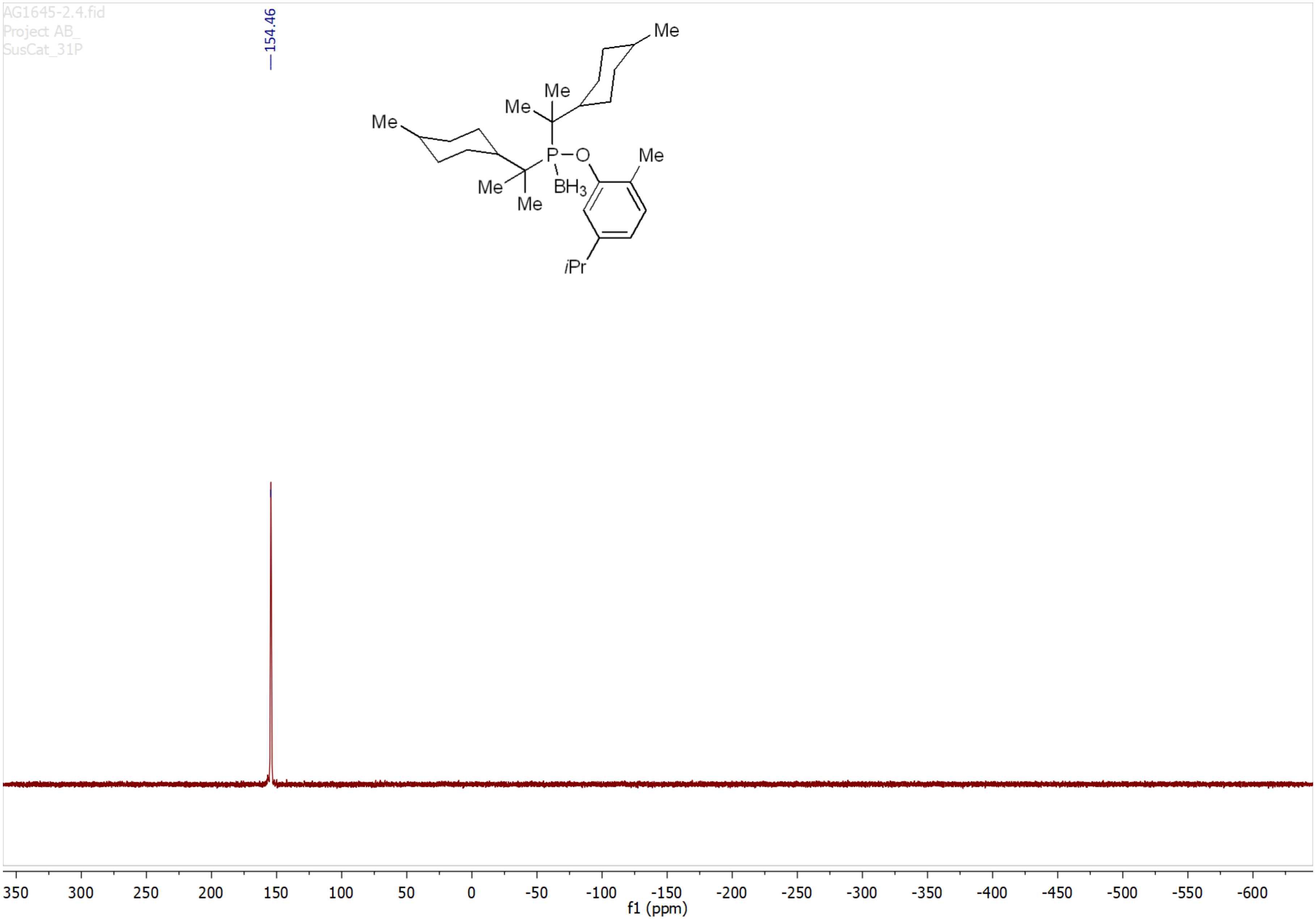

Spectrum S85. The ^{1}H NMR spectrum of *GreenPhos 18* in $CDCl_3$.

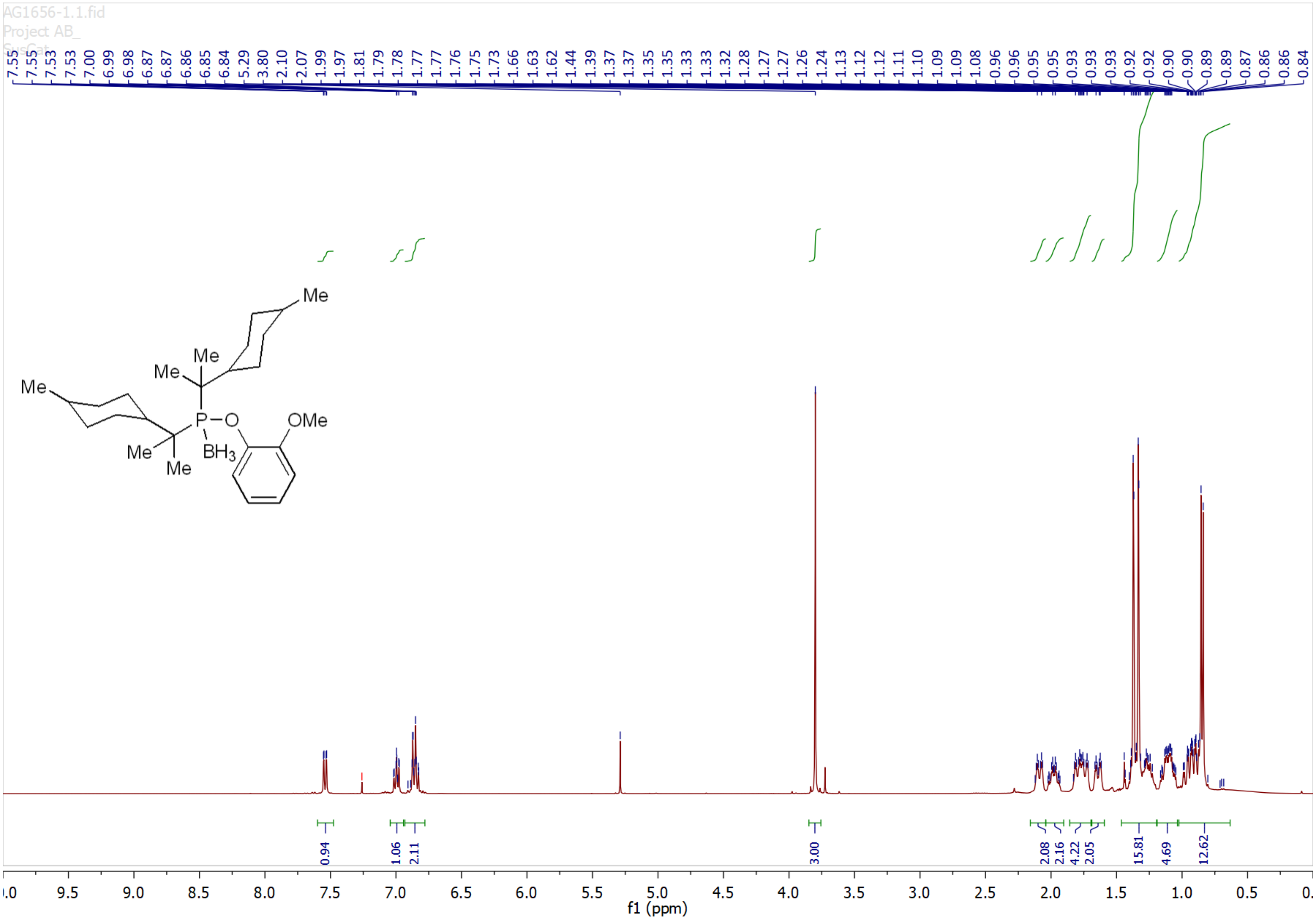

Spectrum S86. The ^{13}C NMR spectrum of *GreenPhos 18* in $CDCl_3$.

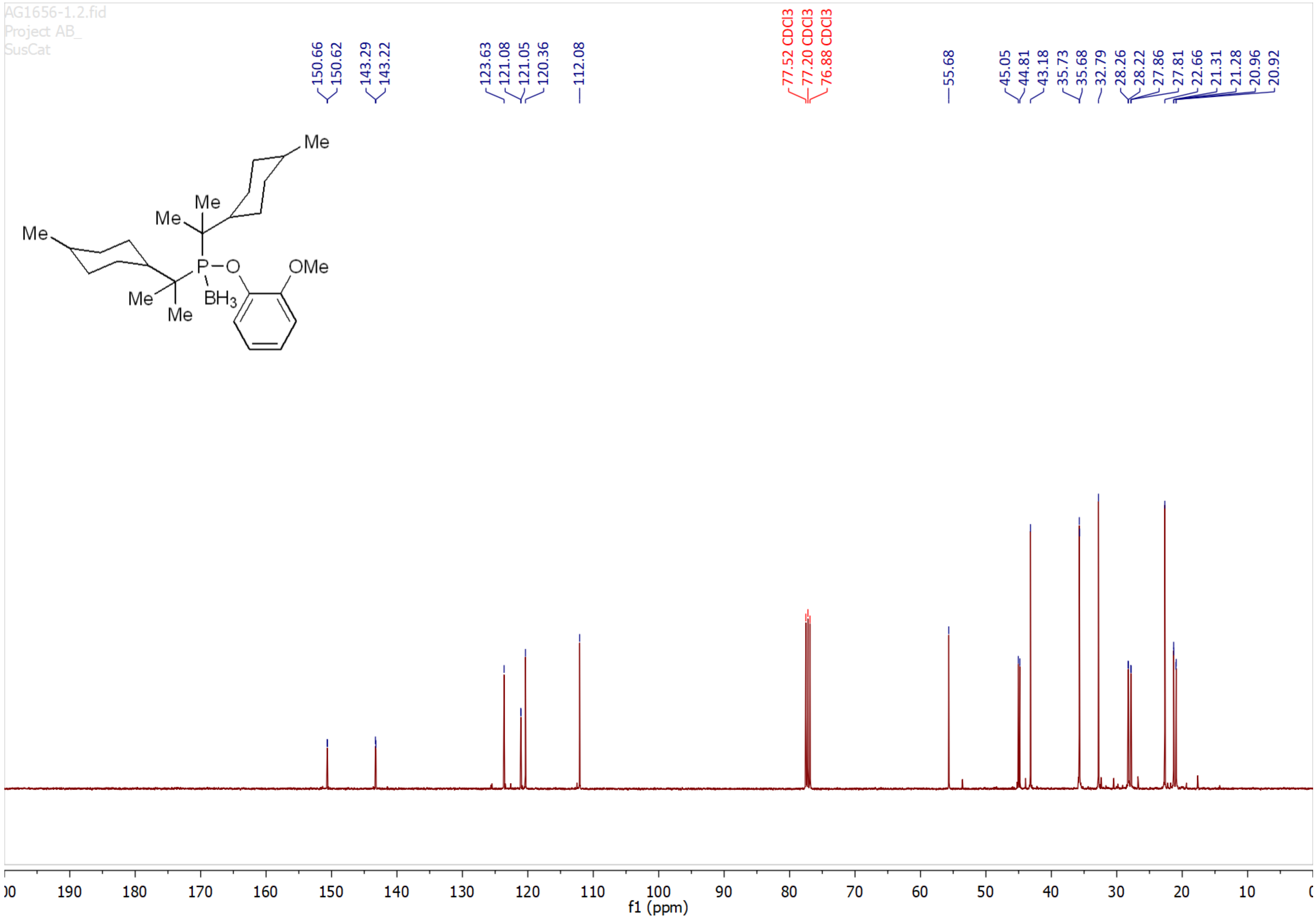

Spectrum S87. The ^{31}P NMR spectrum of *GreenPhos 18* with composite pulse decoupling in $CDCl_3$.

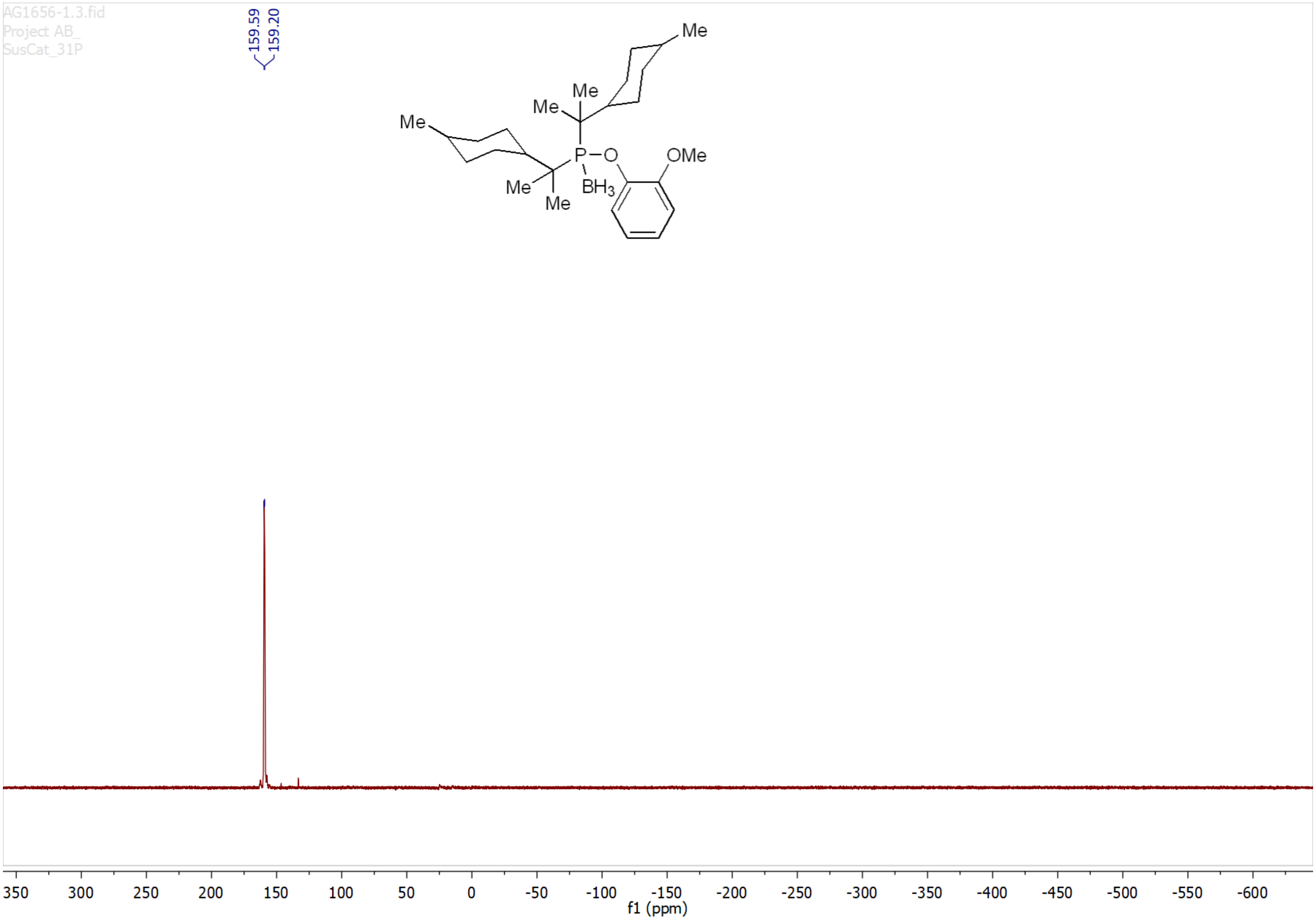

Spectrum S88. The ^{31}P NMR spectrum of *GreenPhos 18* with no decoupling in $CDCl_3$.

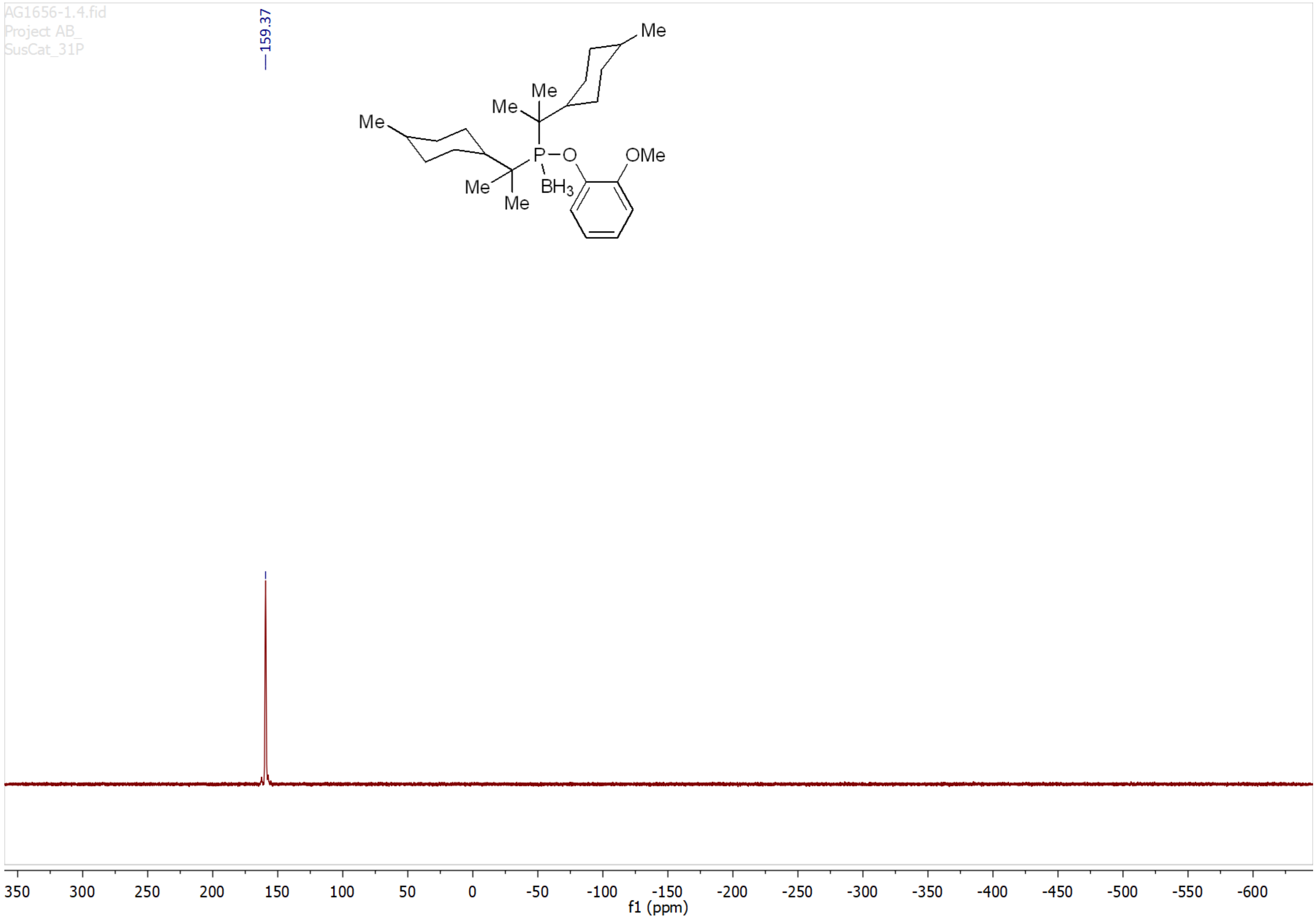

Spectrum S89. The ^{1}H NMR spectrum of *GreenPhos 19* in $CDCl_3$.

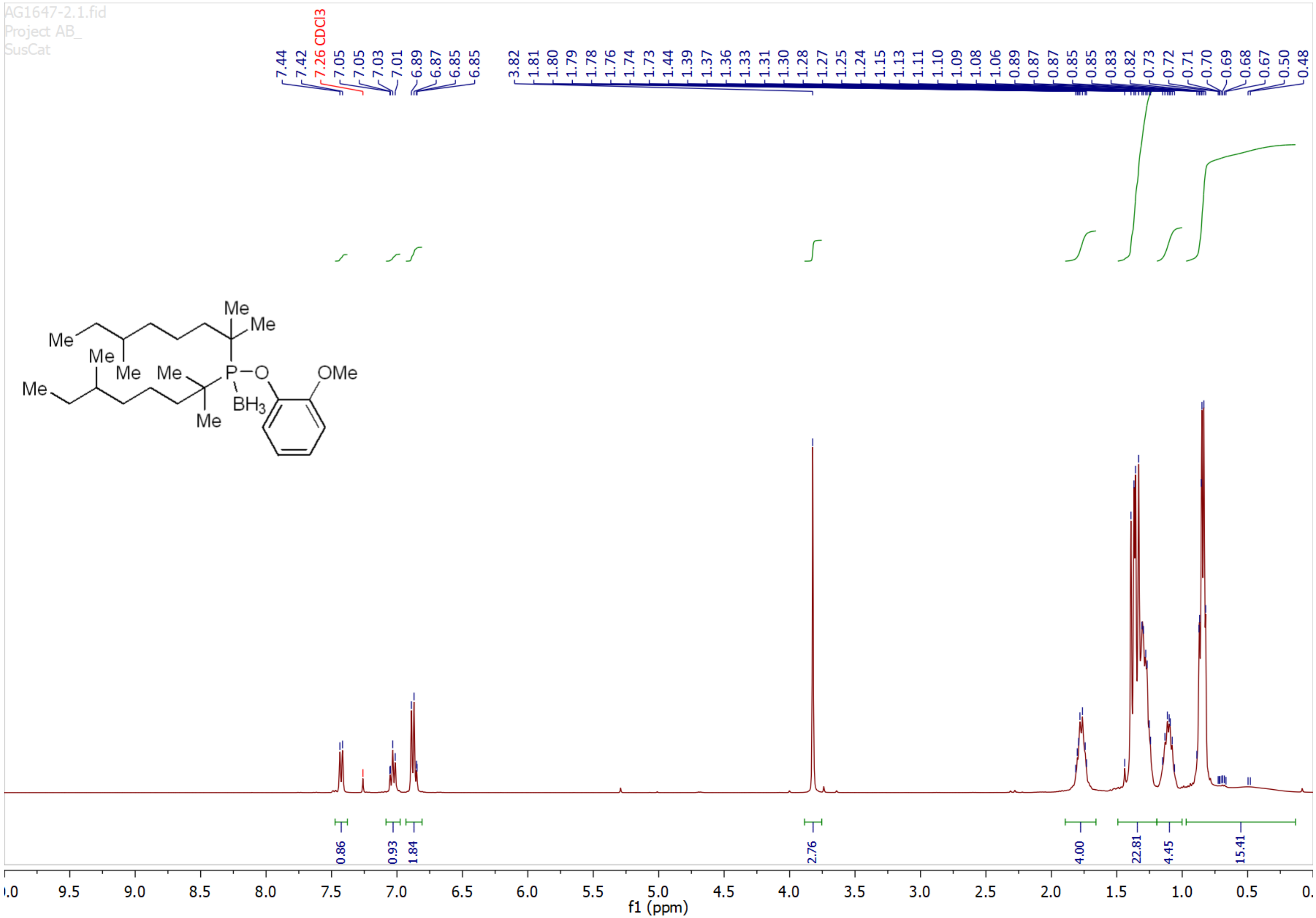

Spectrum S90. The ^{13}C NMR spectrum of *GreenPhos 19* in $CDCl_3$.

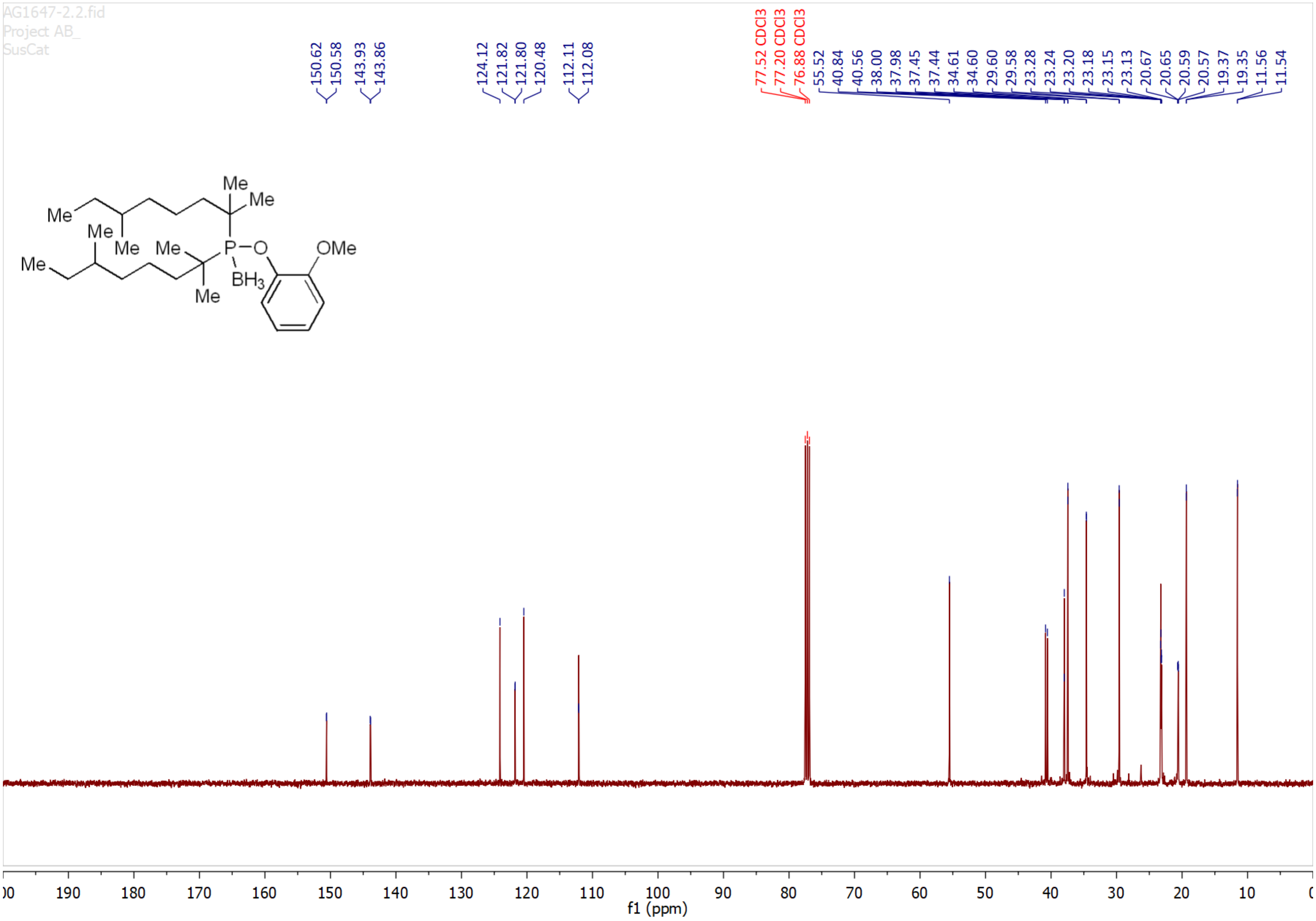

Spectrum S91. The ^{31}P NMR spectrum of *GreenPhos 19* with composite pulse decoupling in $CDCl_3$.

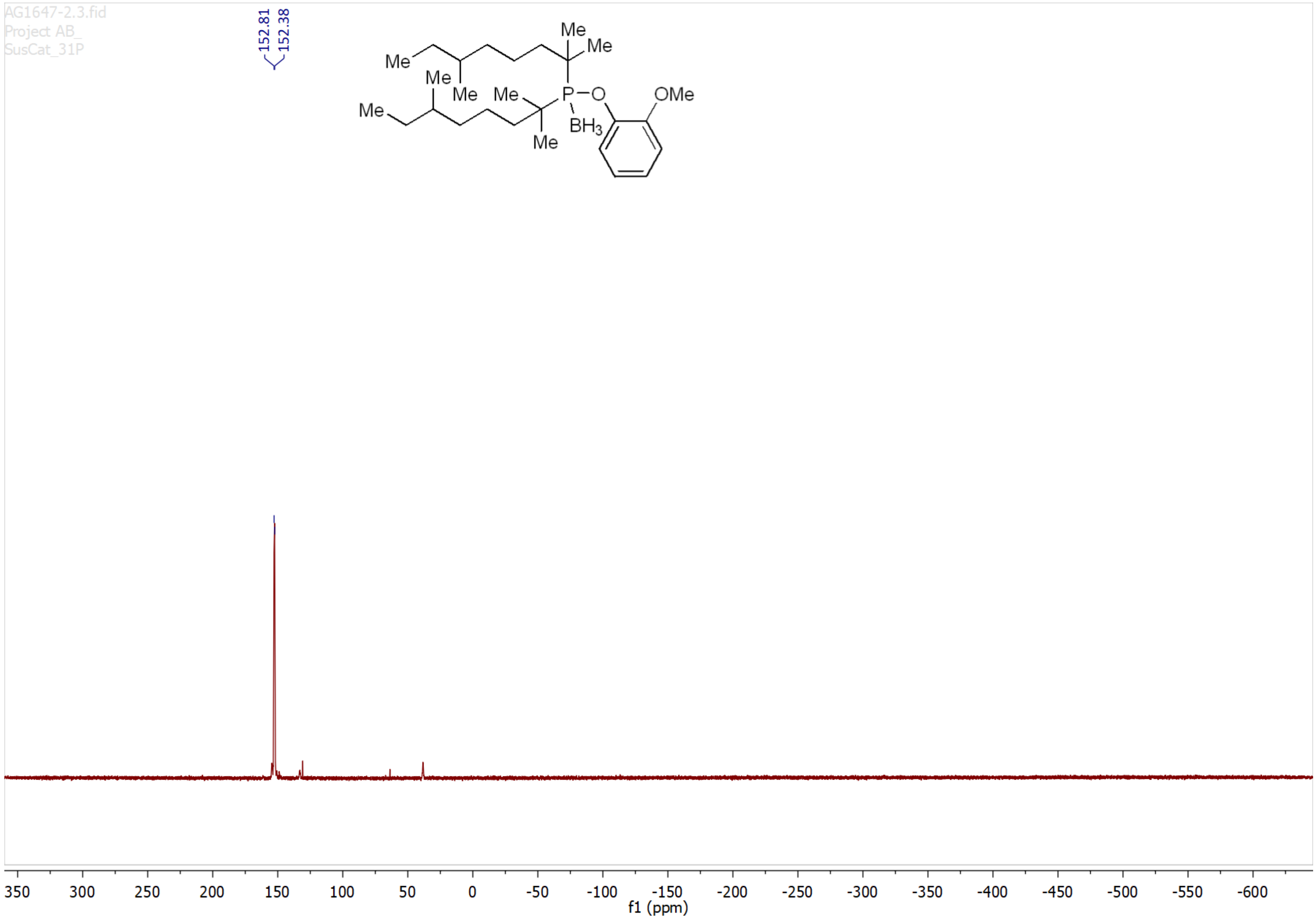

Spectrum S92. The ^{31}P NMR spectrum of *GreenPhos 19* with no decoupling in $CDCl_3$.

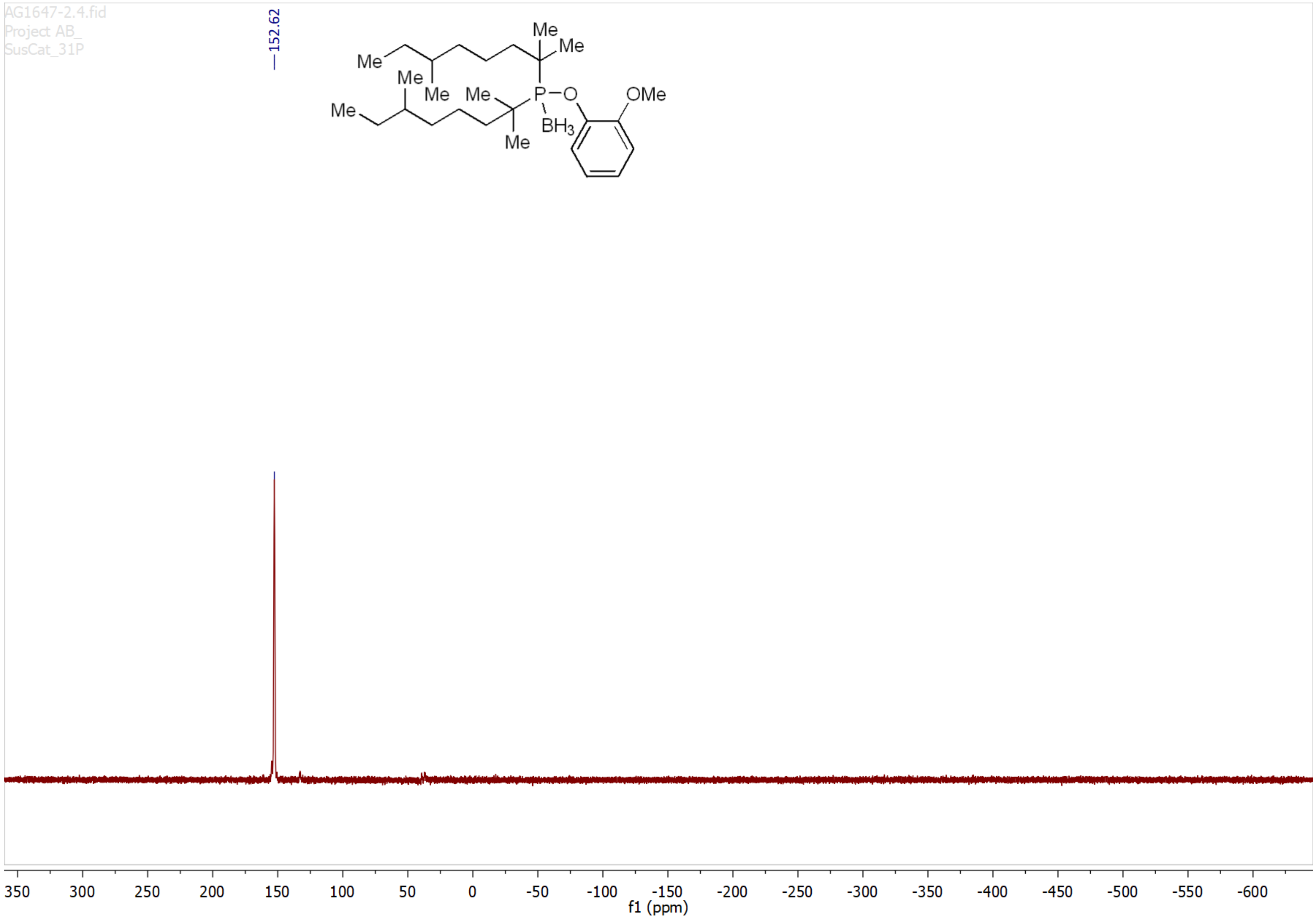

Spectrum S93. The ^{1}H NMR spectrum of *GreenPhos 20* in $CDCl_3$.

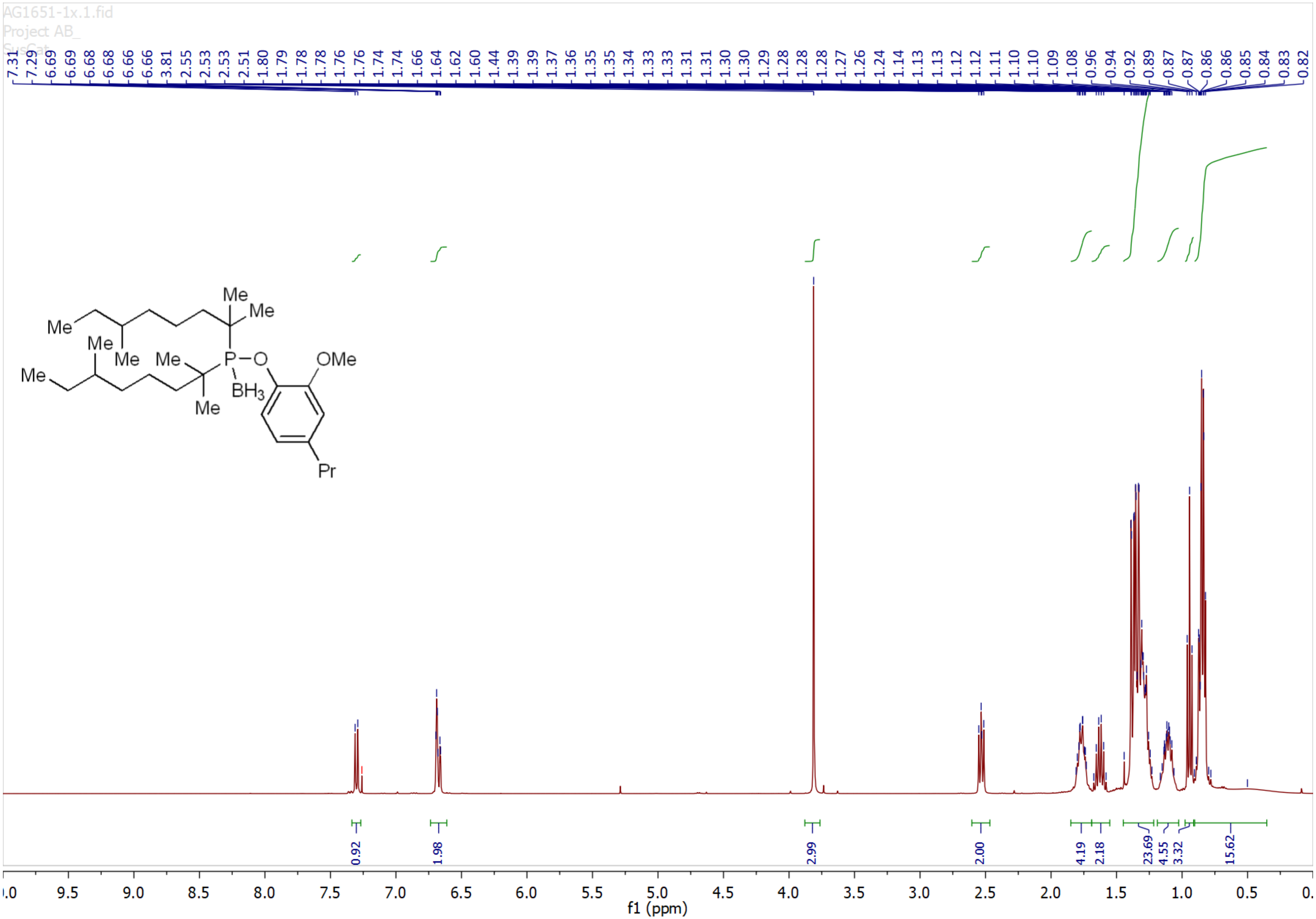

Spectrum S94. The ^{13}C NMR spectrum of *GreenPhos 20* in $CDCl_3$.

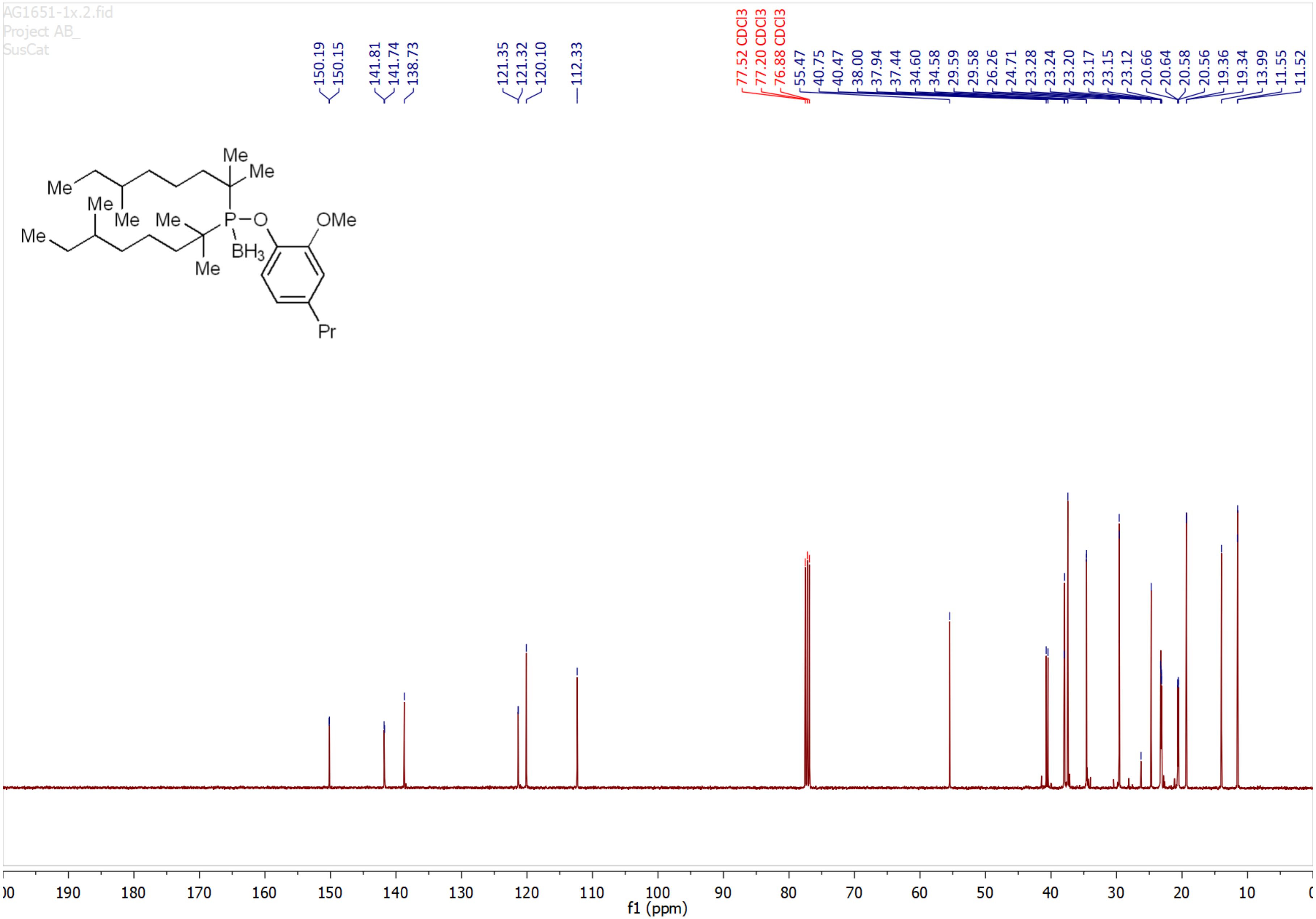

Spectrum S95. The ^{31}P NMR spectrum of *GreenPhos 20* with composite pulse decoupling in $CDCl_3$.

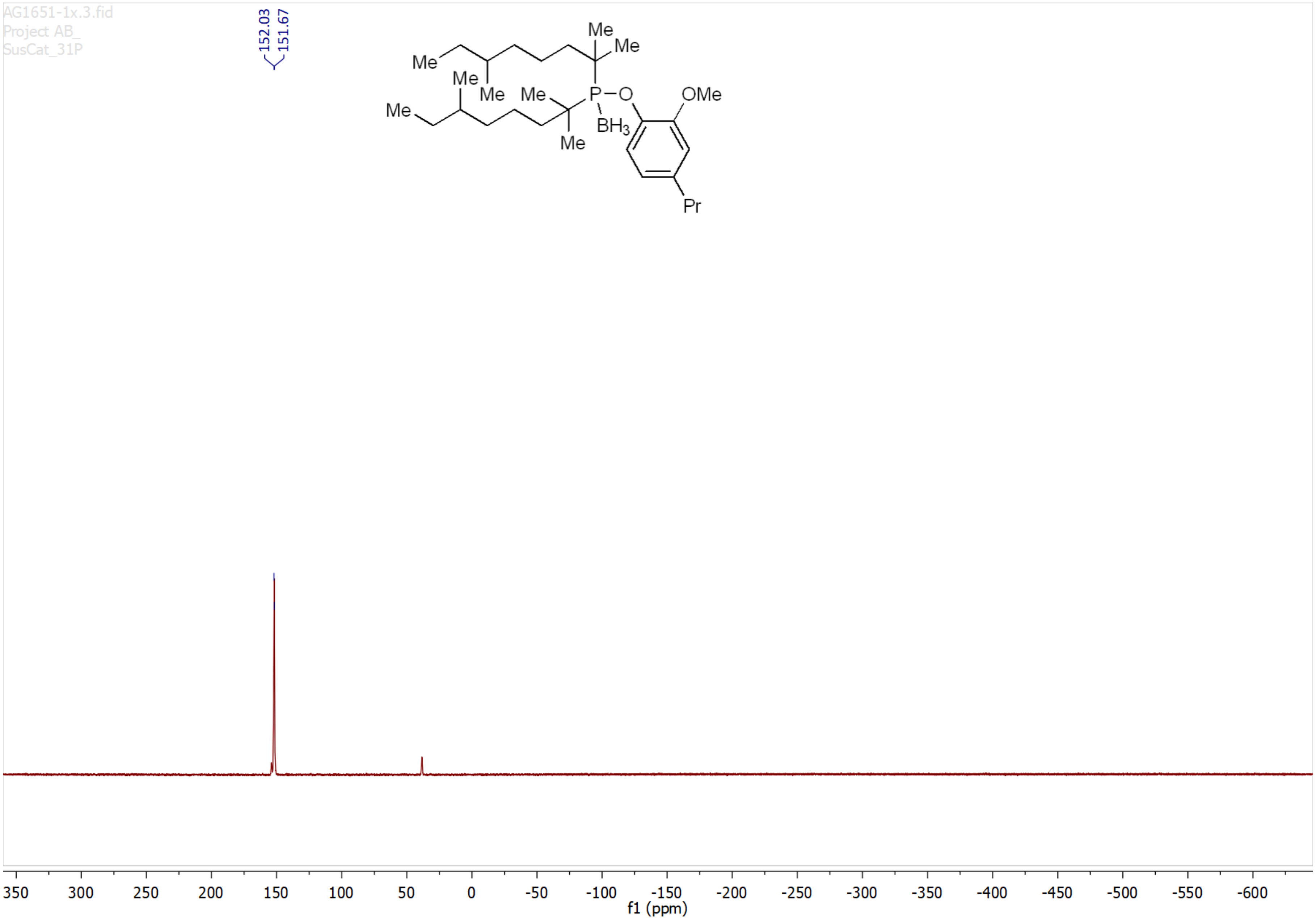

Spectrum S96. The ^{31}P NMR spectrum of *GreenPhos 20* with no decoupling in $CDCl_3$.

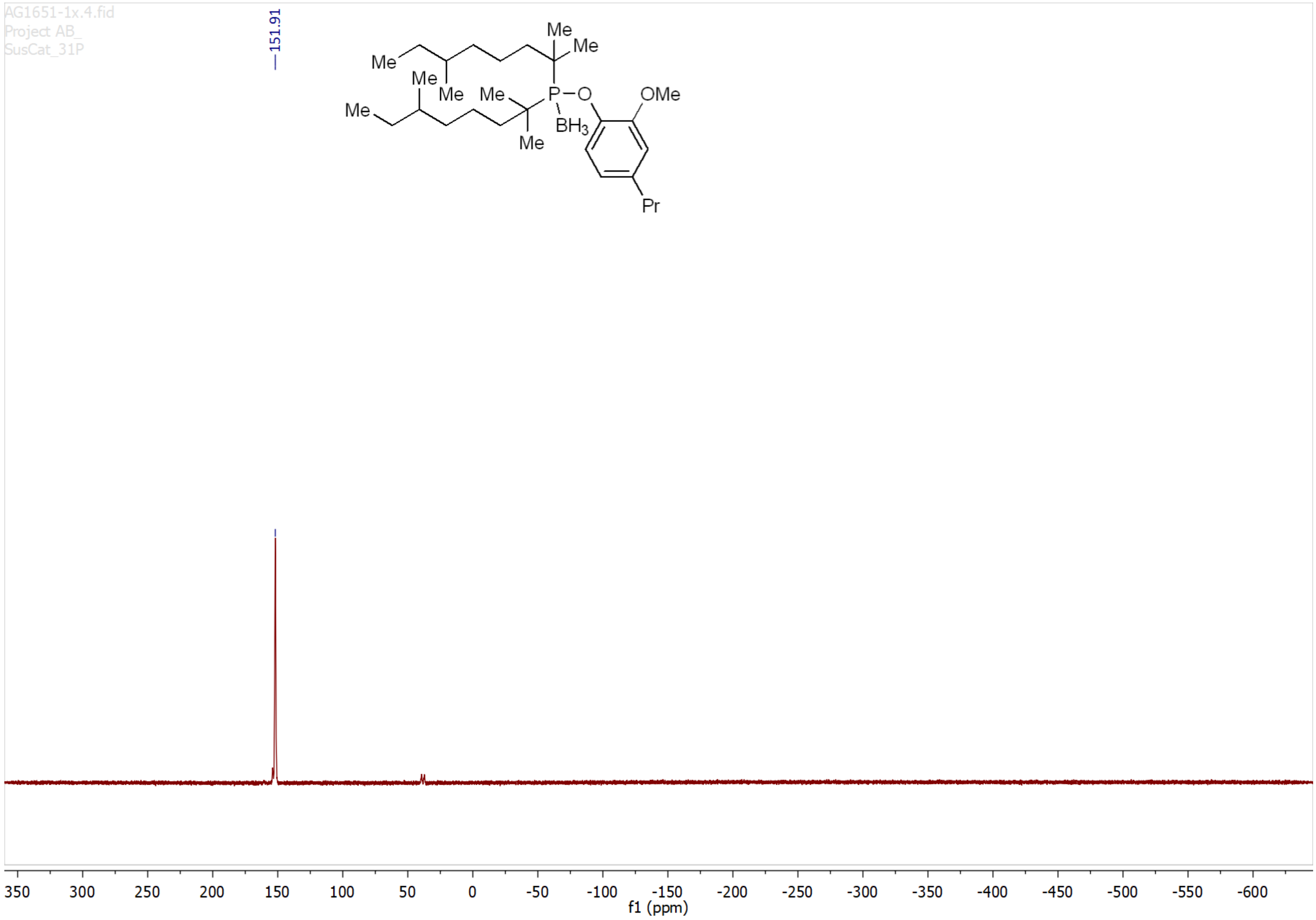

Spectrum S97. The crude ^{31}P NMR spectrum of phosphine selenide derived from *GreenPhos 4* in $CDCl_3$.

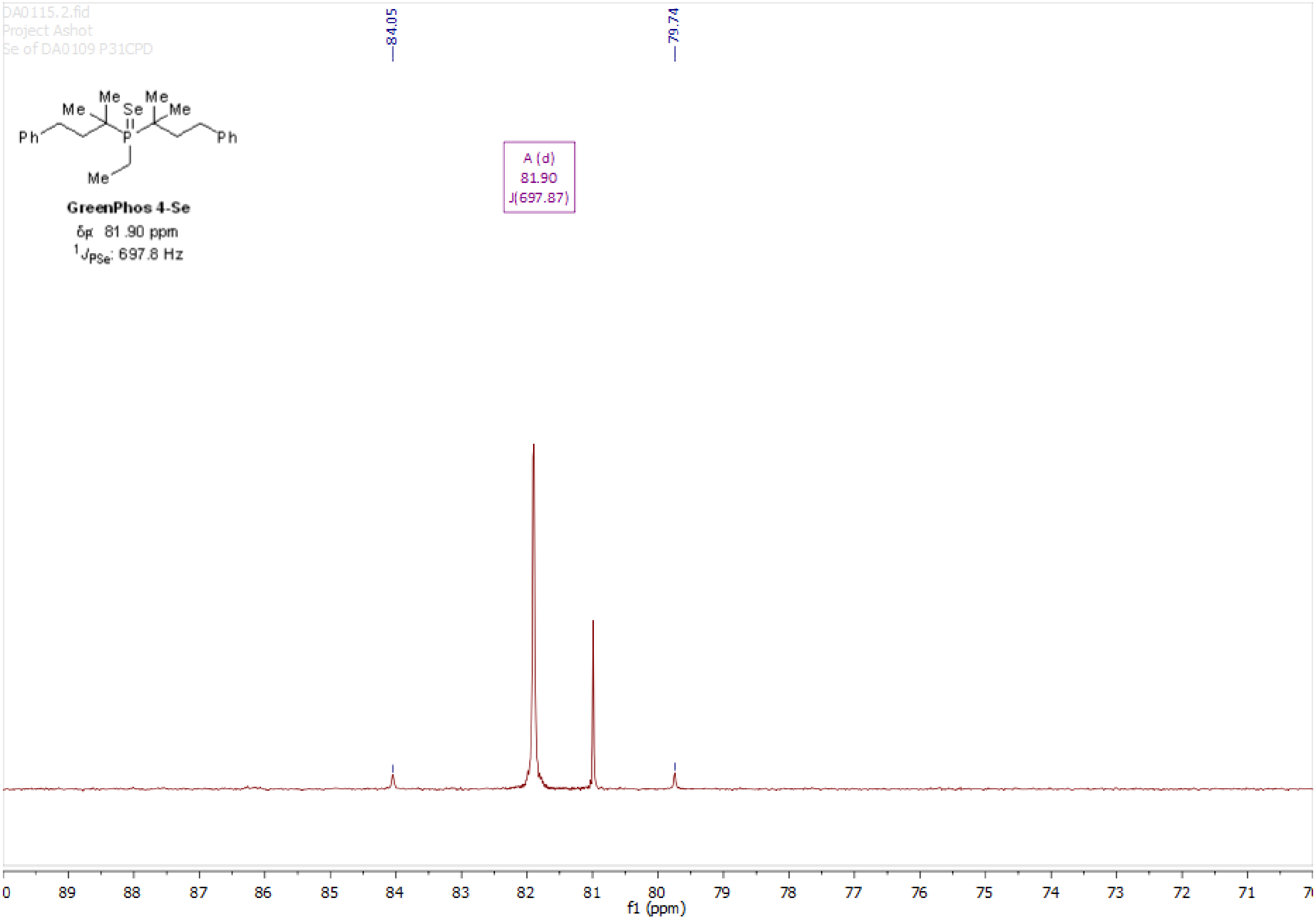

Spectrum S98. The crude ^{31}P NMR spectrum of phosphine selenide derived from *GreenPhos 11* in $CDCl_3$.

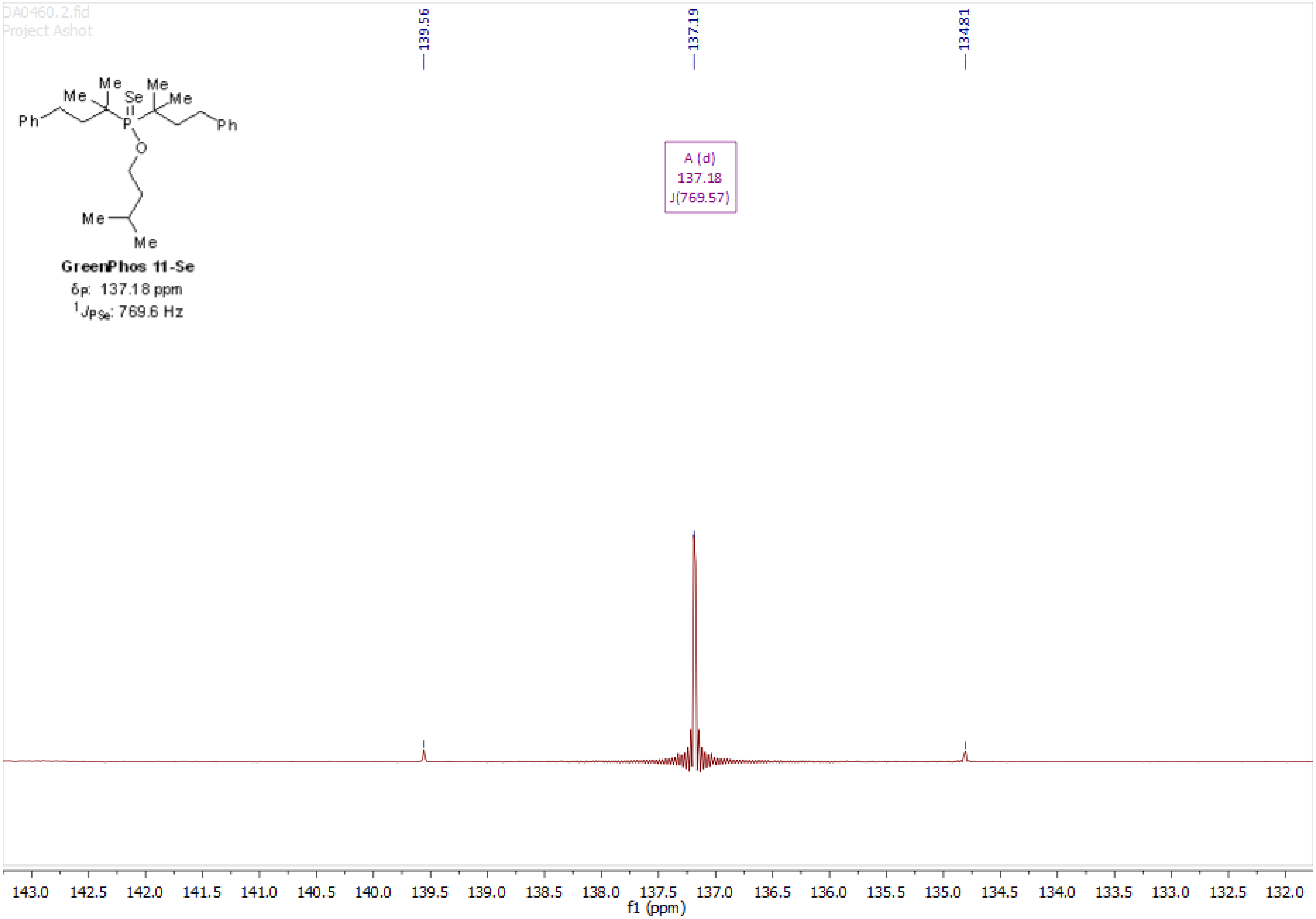

Spectrum S99. The ^{1}H NMR spectrum of *3a* in $CDCl_3$.

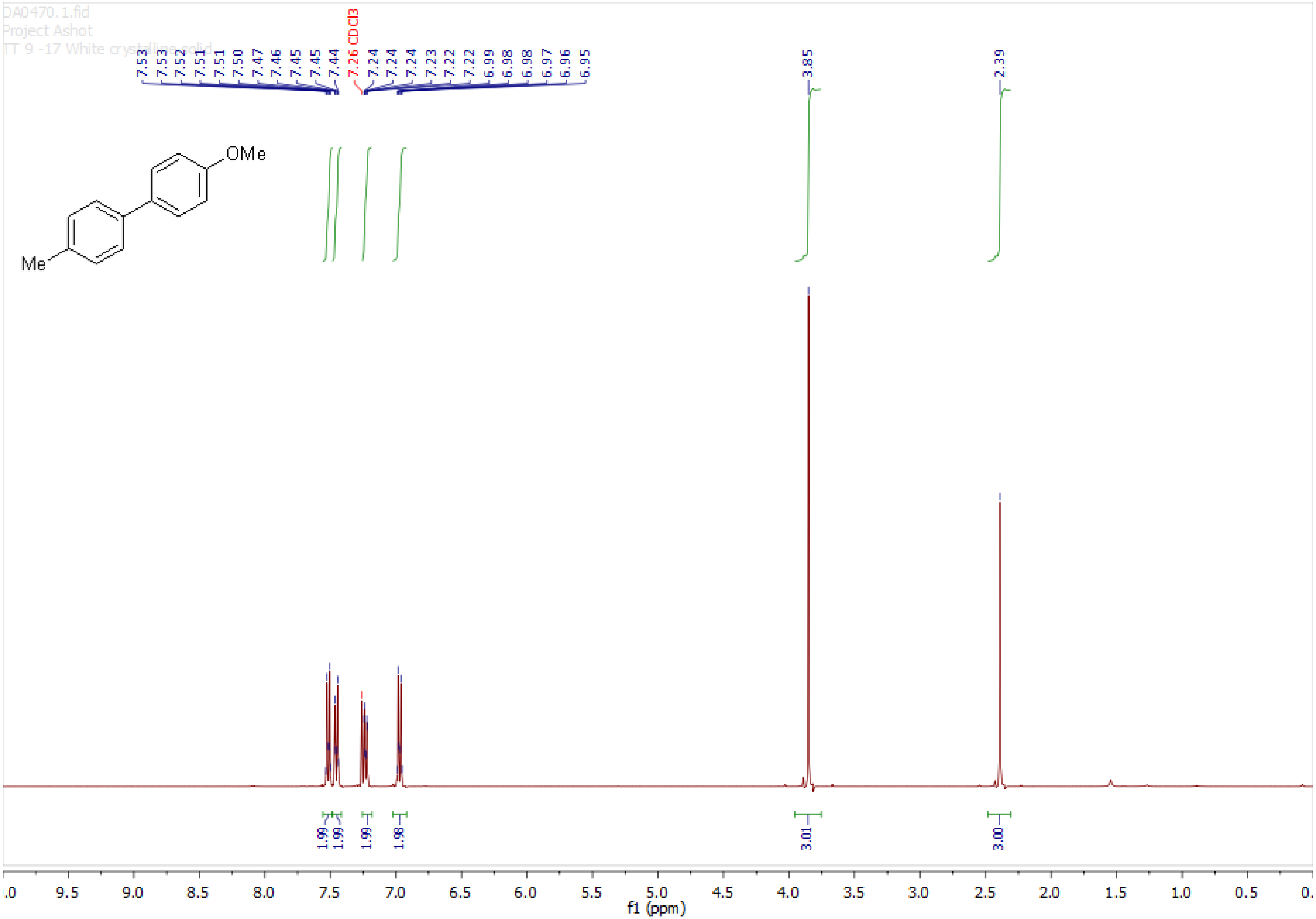

Spectrum S100. The ^{13}C NMR spectrum of *3a* in $CDCl_3$.

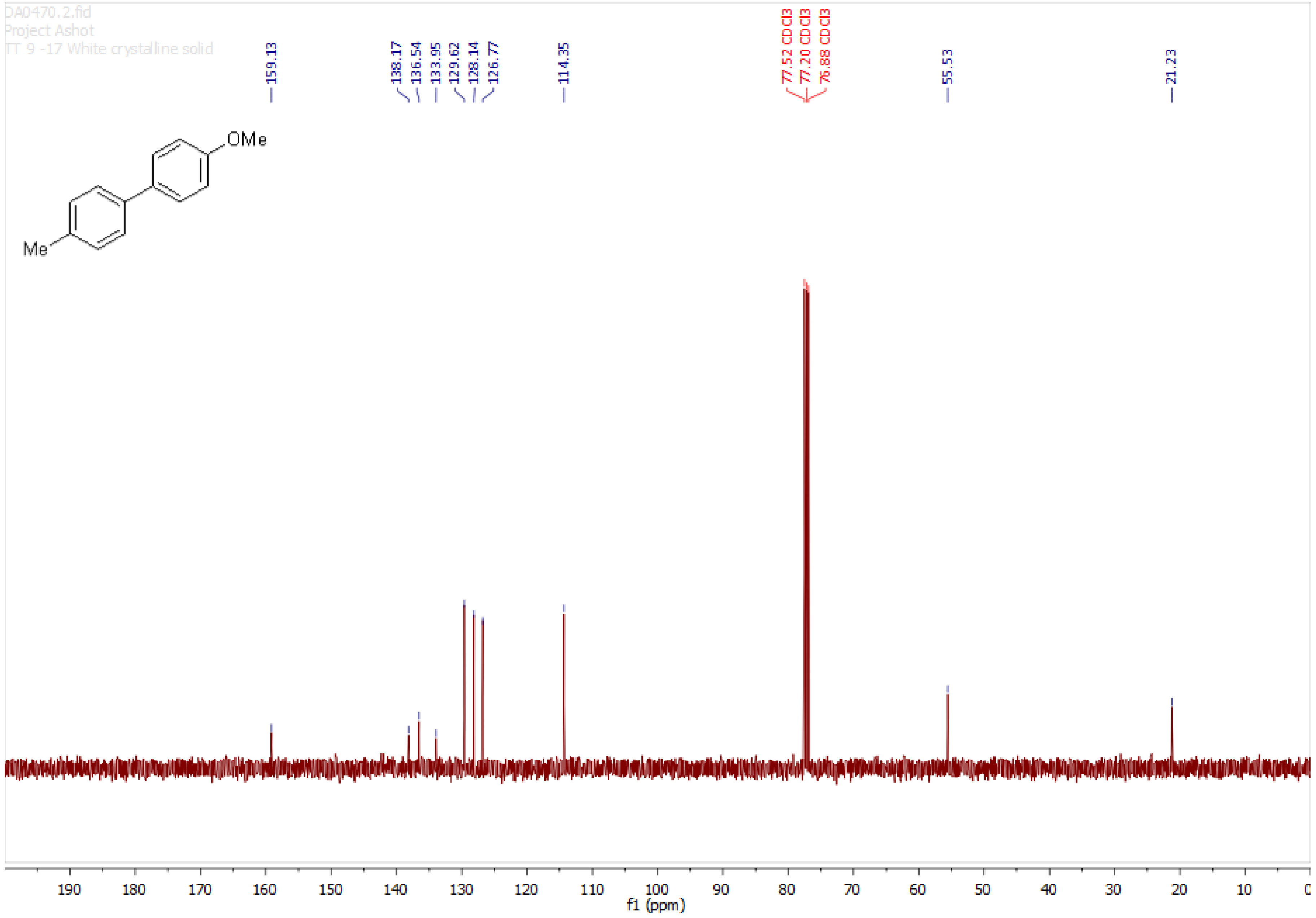

Spectrum S101. The ^{1}H NMR spectrum of *3b* in $CDCl_3$.

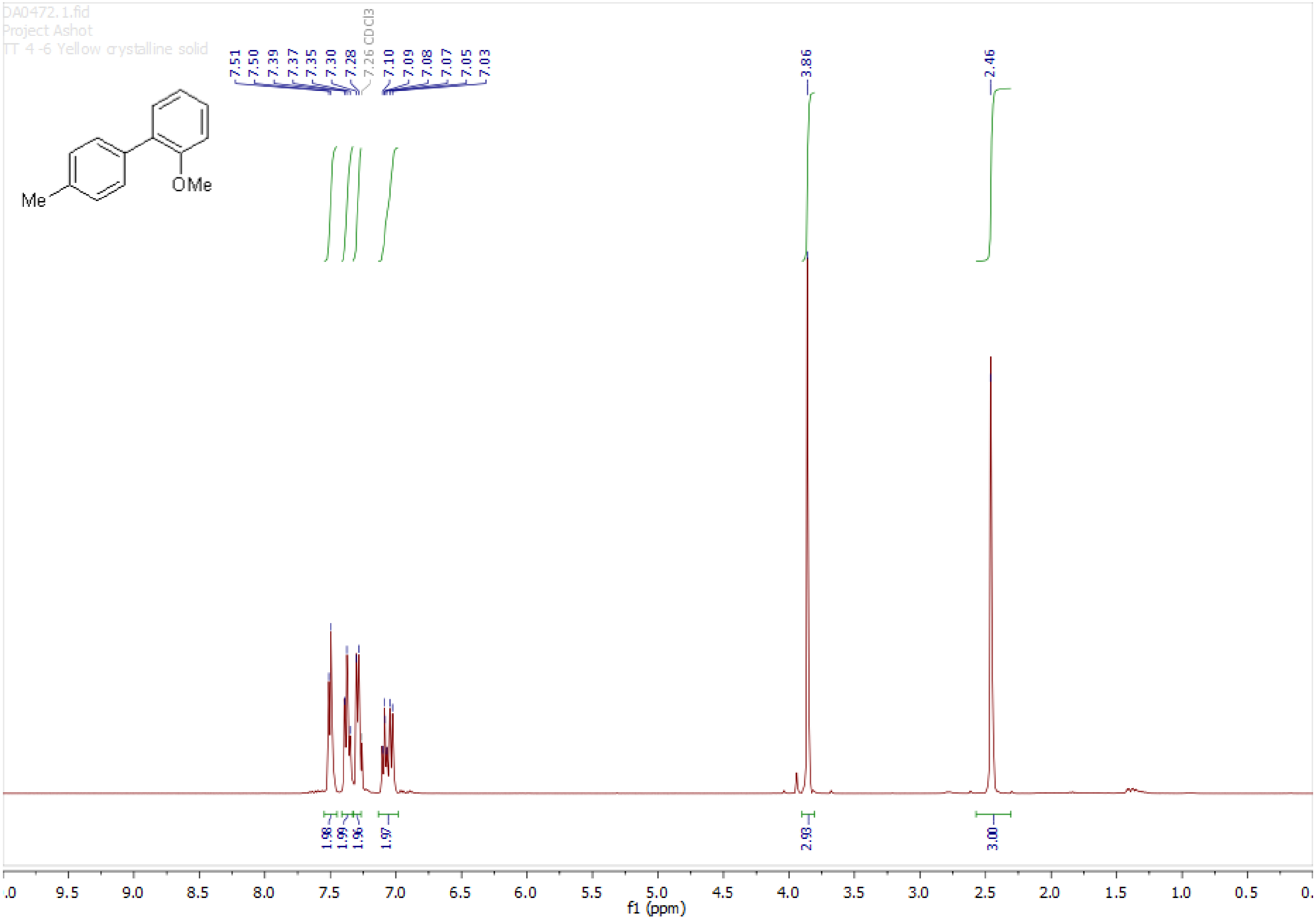

Spectrum S102. The ^{13}C NMR spectrum of *3b* in $CDCl_3$.

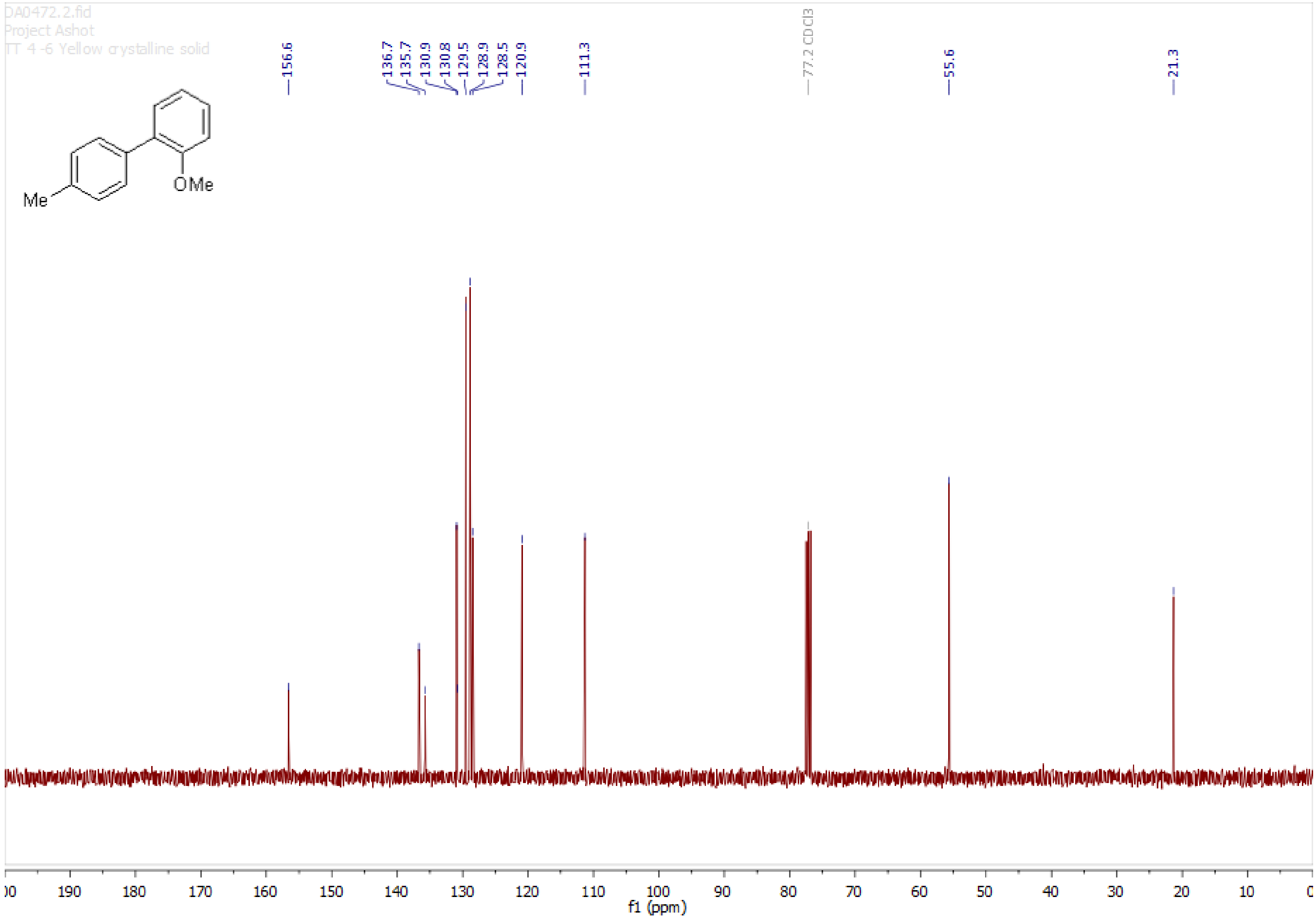

Spectrum S103. The ^{1}H NMR spectrum of *3c* in $CDCl_3$.

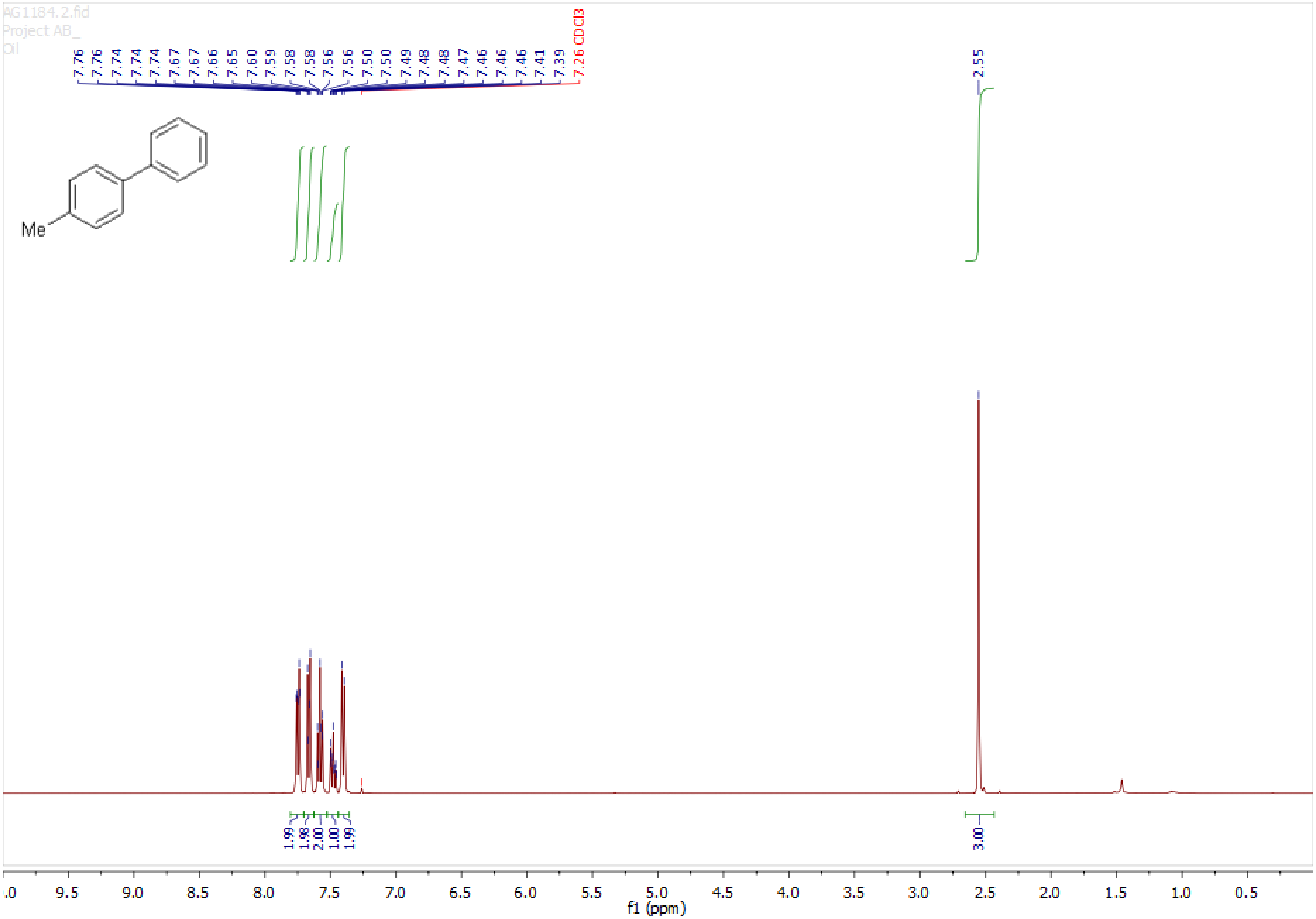

Spectrum S104. The ^{13}C NMR spectrum of *3c* in $CDCl_3$.

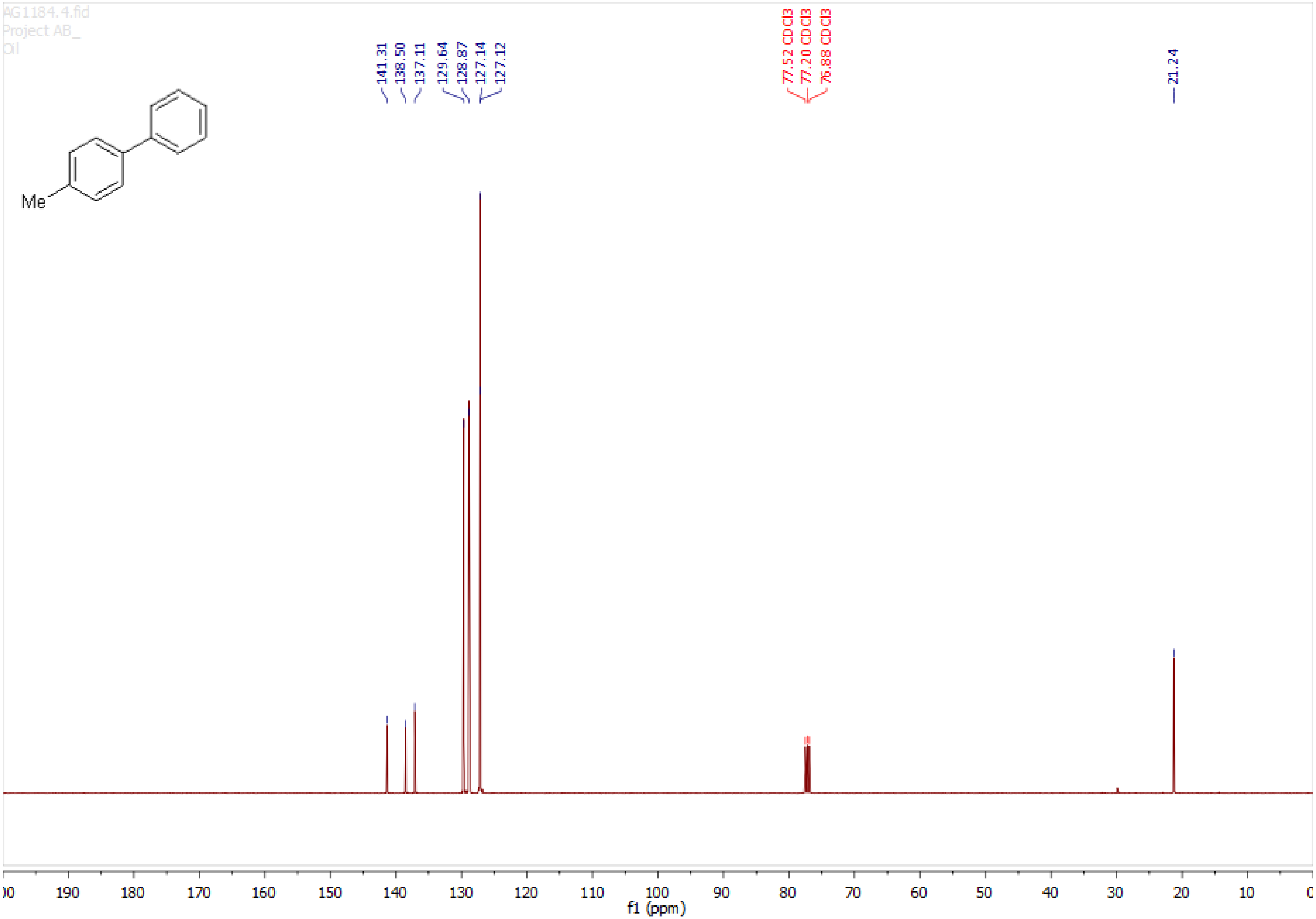

Spectrum S105. The ^{1}H NMR spectrum of *3d* in $CDCl_3$.

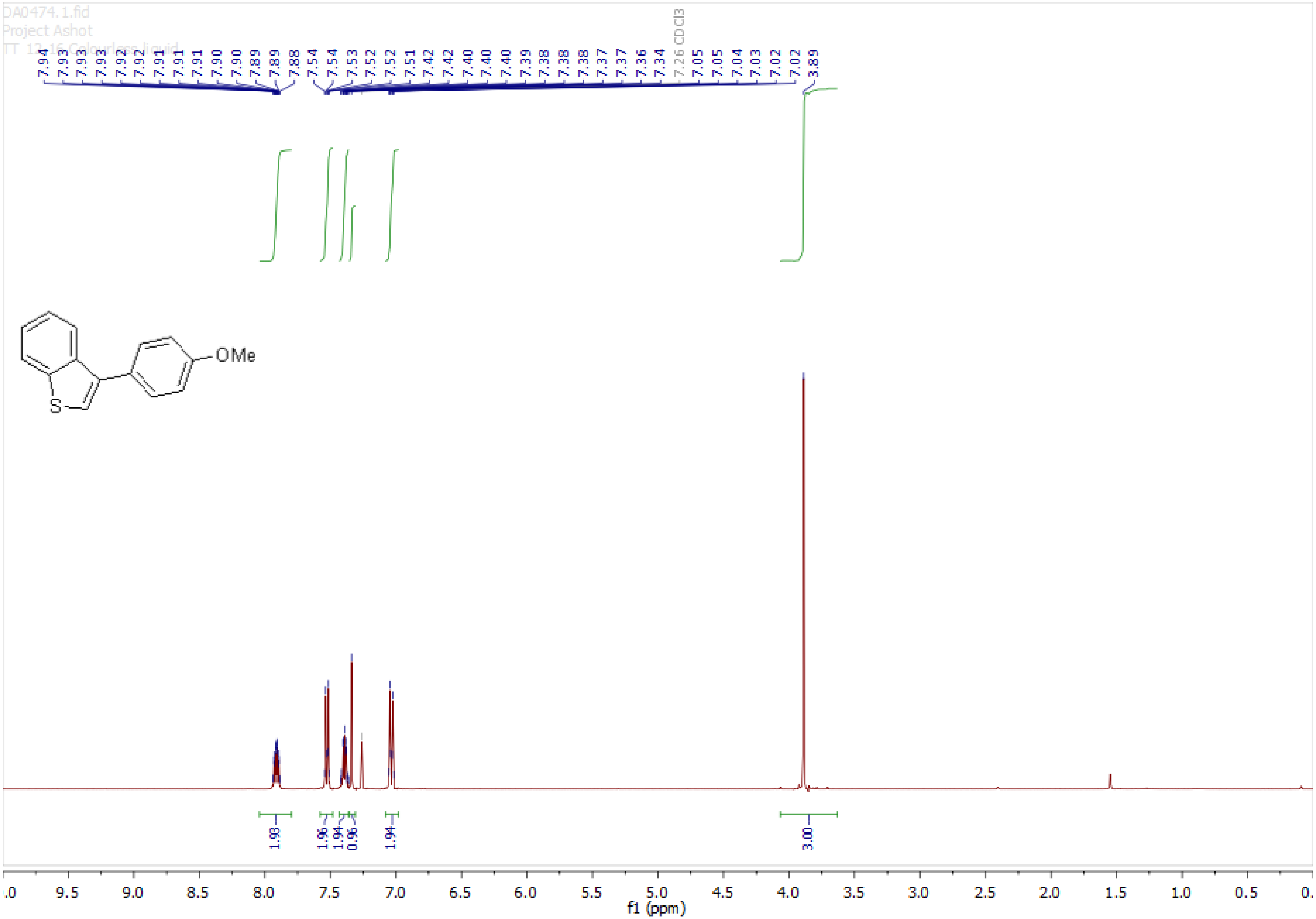

Spectrum S106. The ^{13}C NMR spectrum of *3d* in $CDCl_3$.

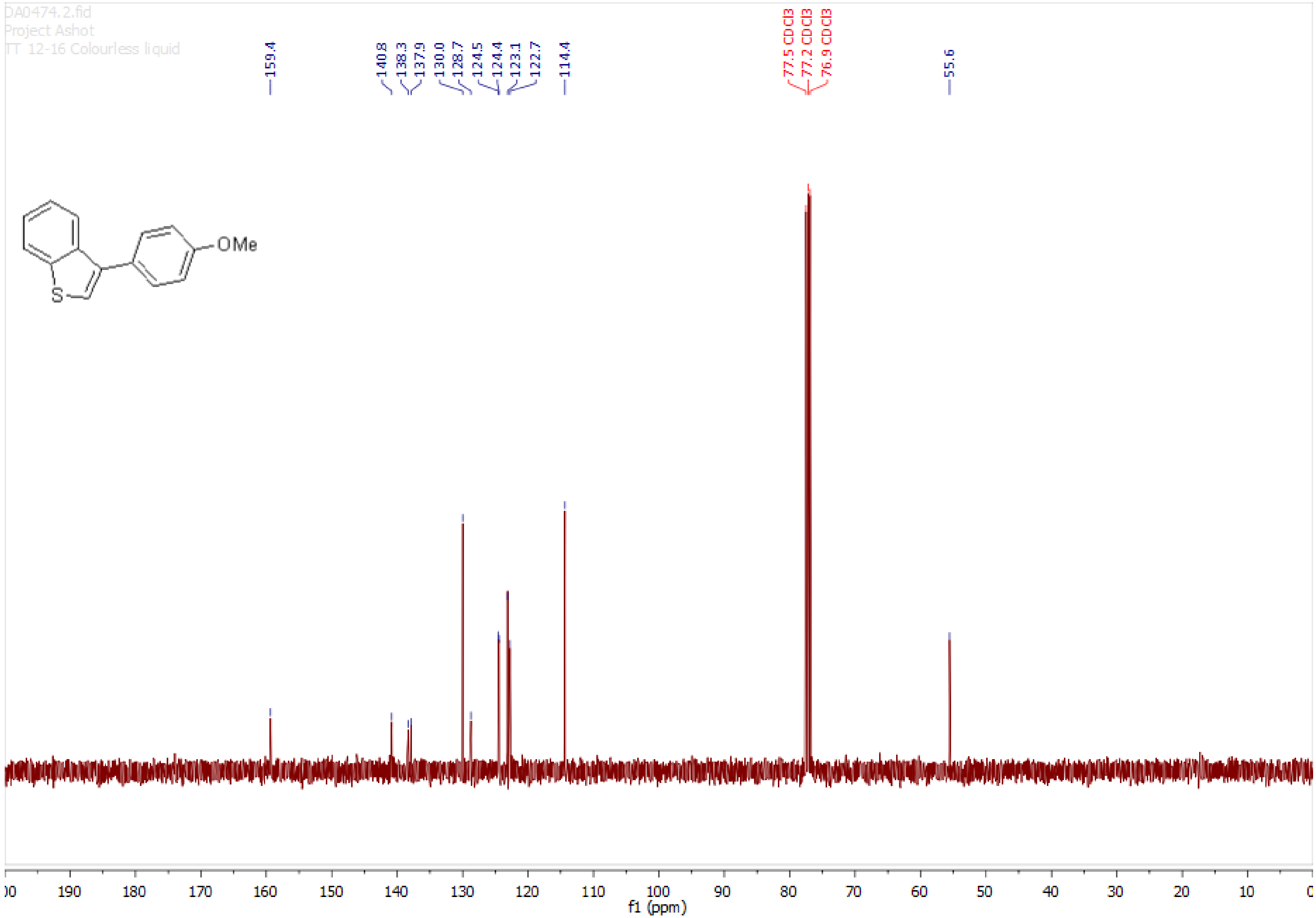

Spectrum S107. The ^{1}H NMR spectrum of *5a* in $CDCl_3$.

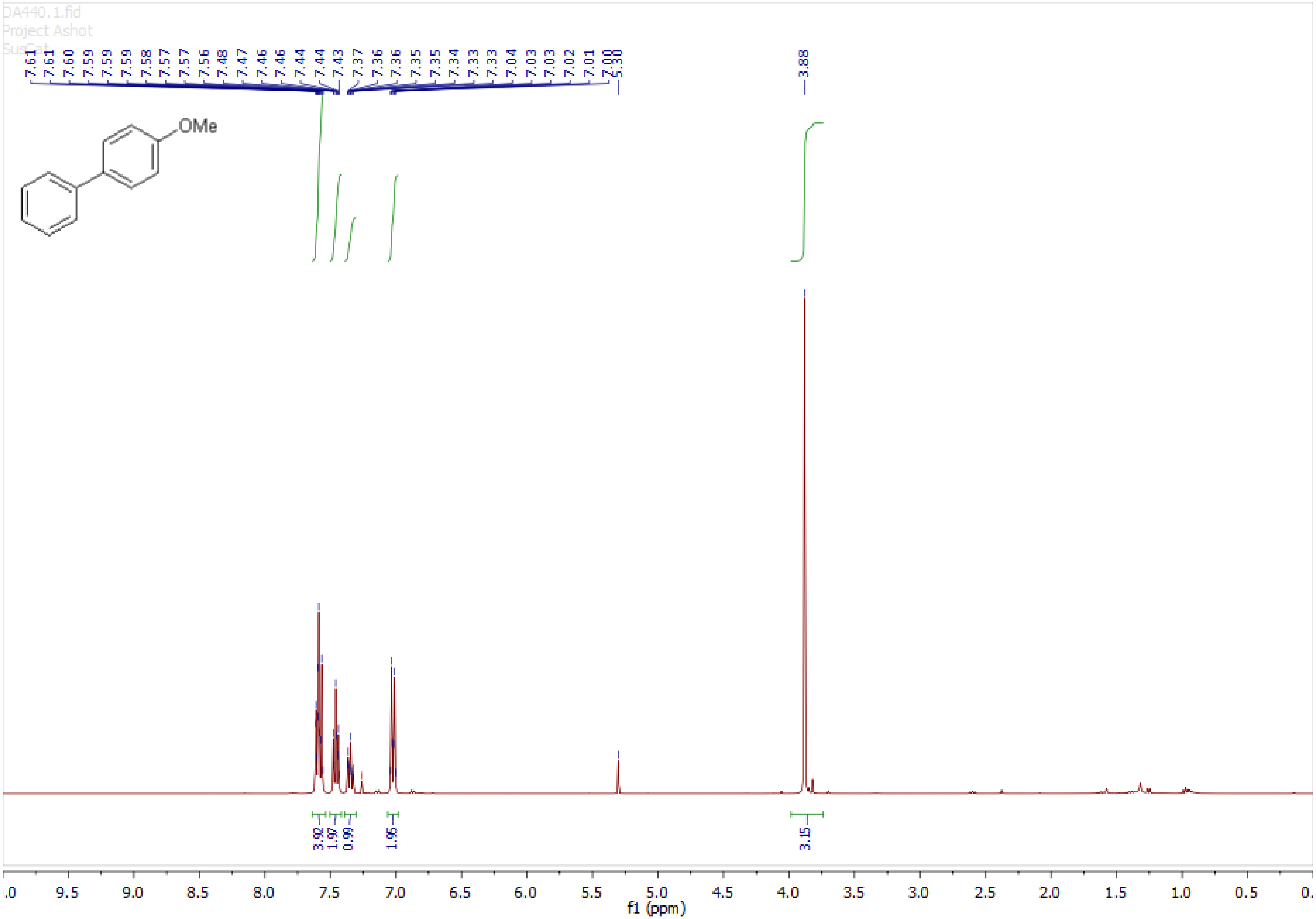

Spectrum S108. The ^{13}C NMR spectrum of *5a* in $CDCl_3$.

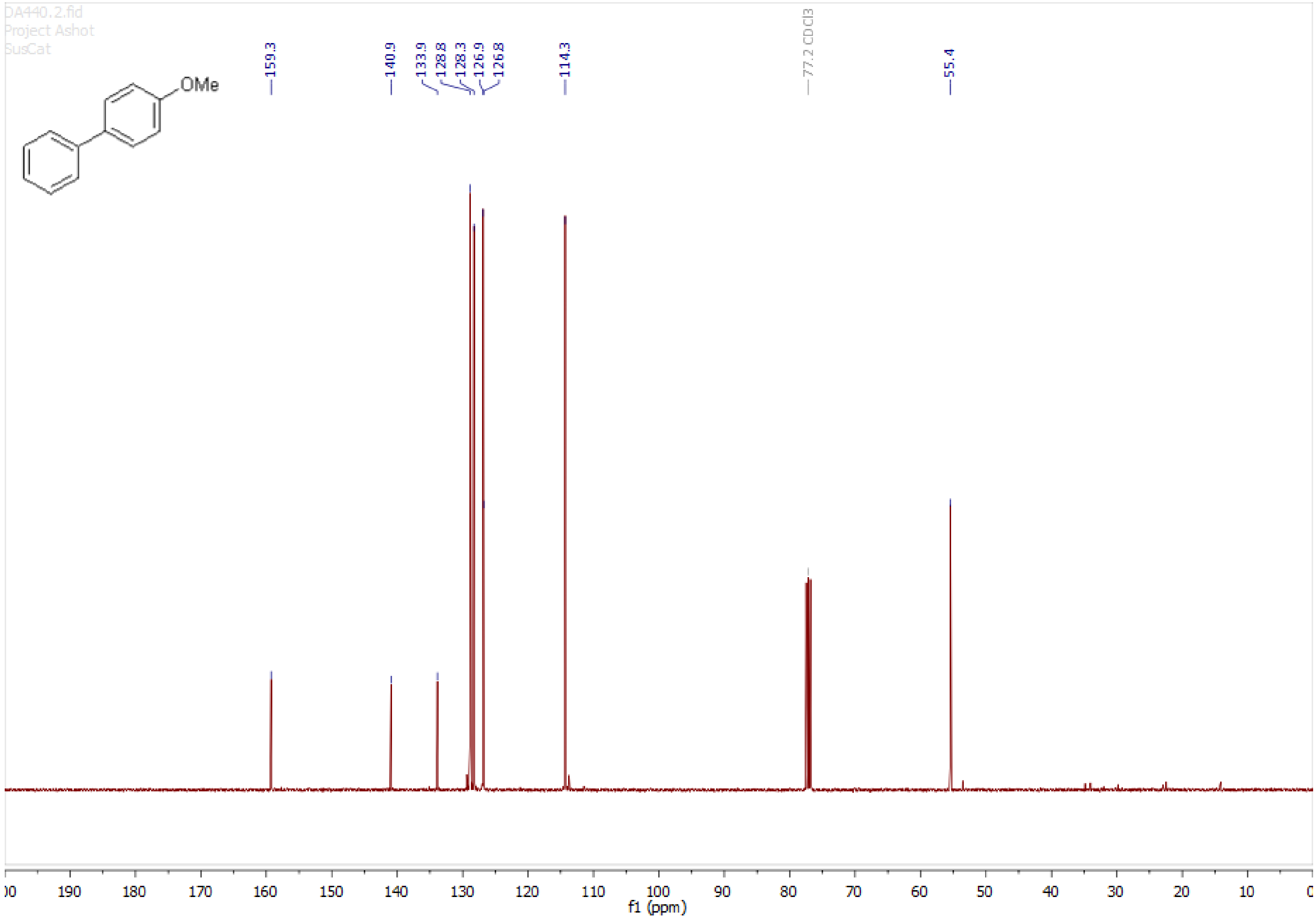

Spectrum S109. The ^{1}H NMR spectrum of *5b* in $CDCl_3$.

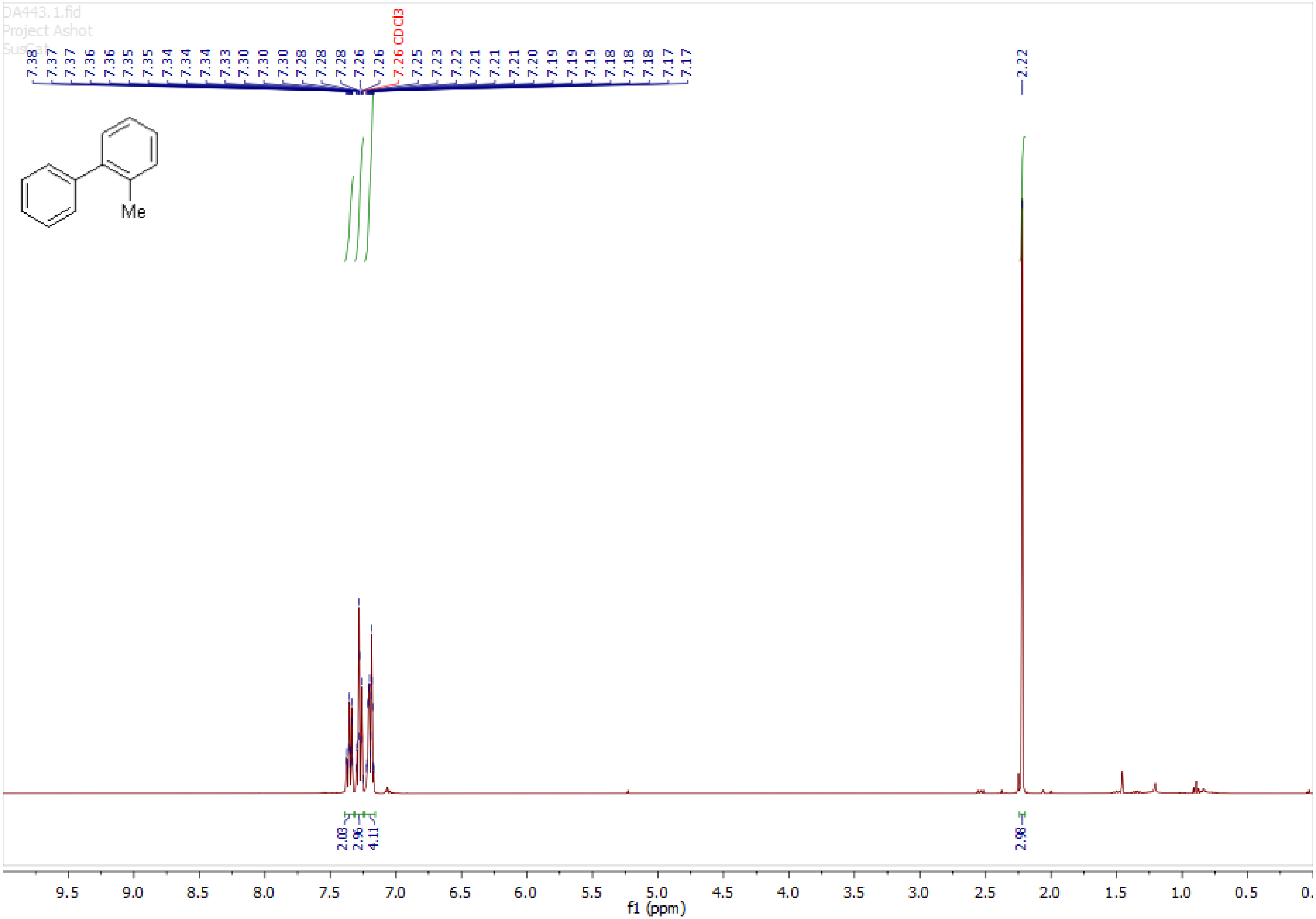

Spectrum S110. The ^{13}C NMR spectrum of *5b* in $CDCl_3$.

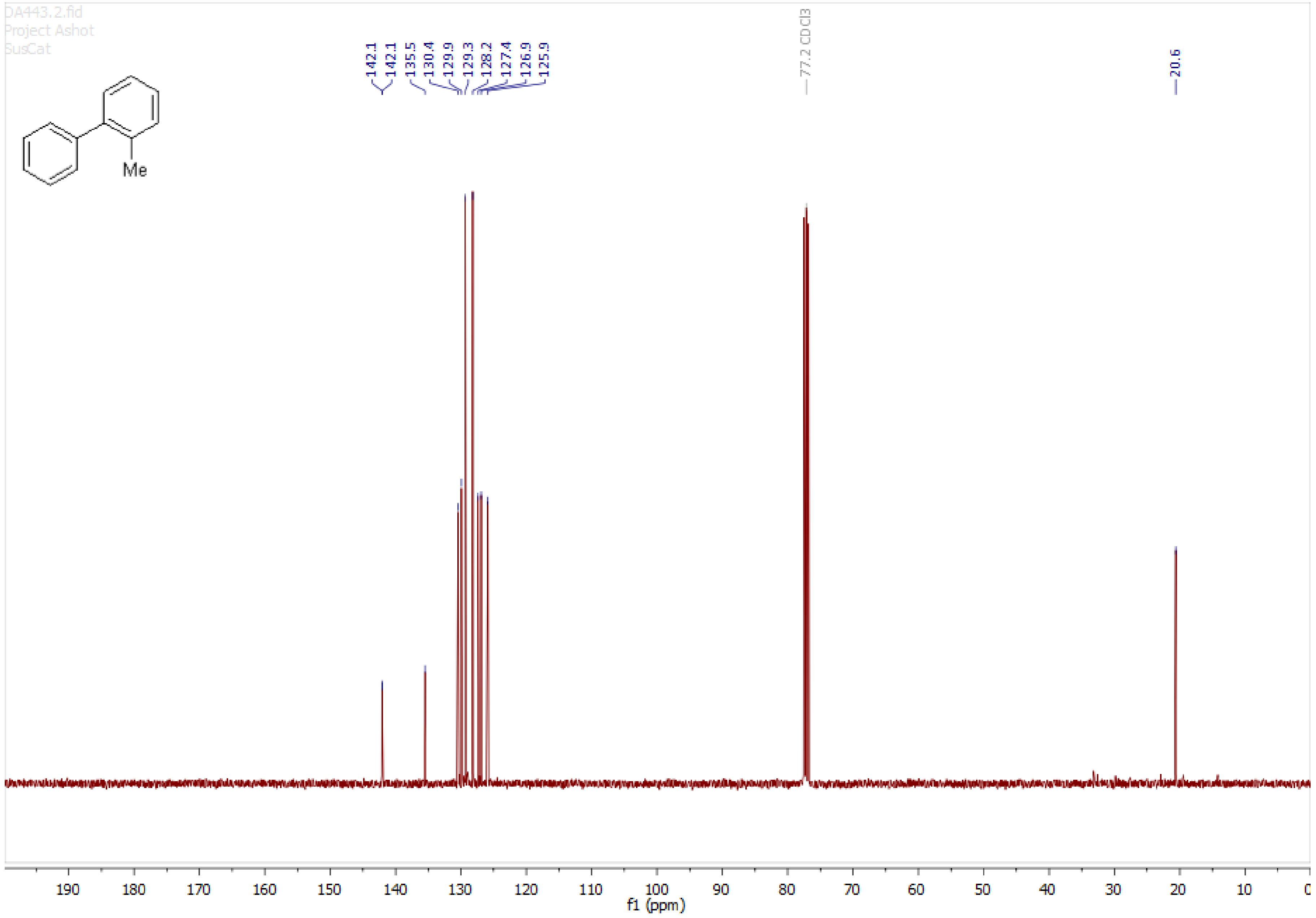

Spectrum S111. The ^{1}H NMR spectrum of *5c* in $CDCl_3$.

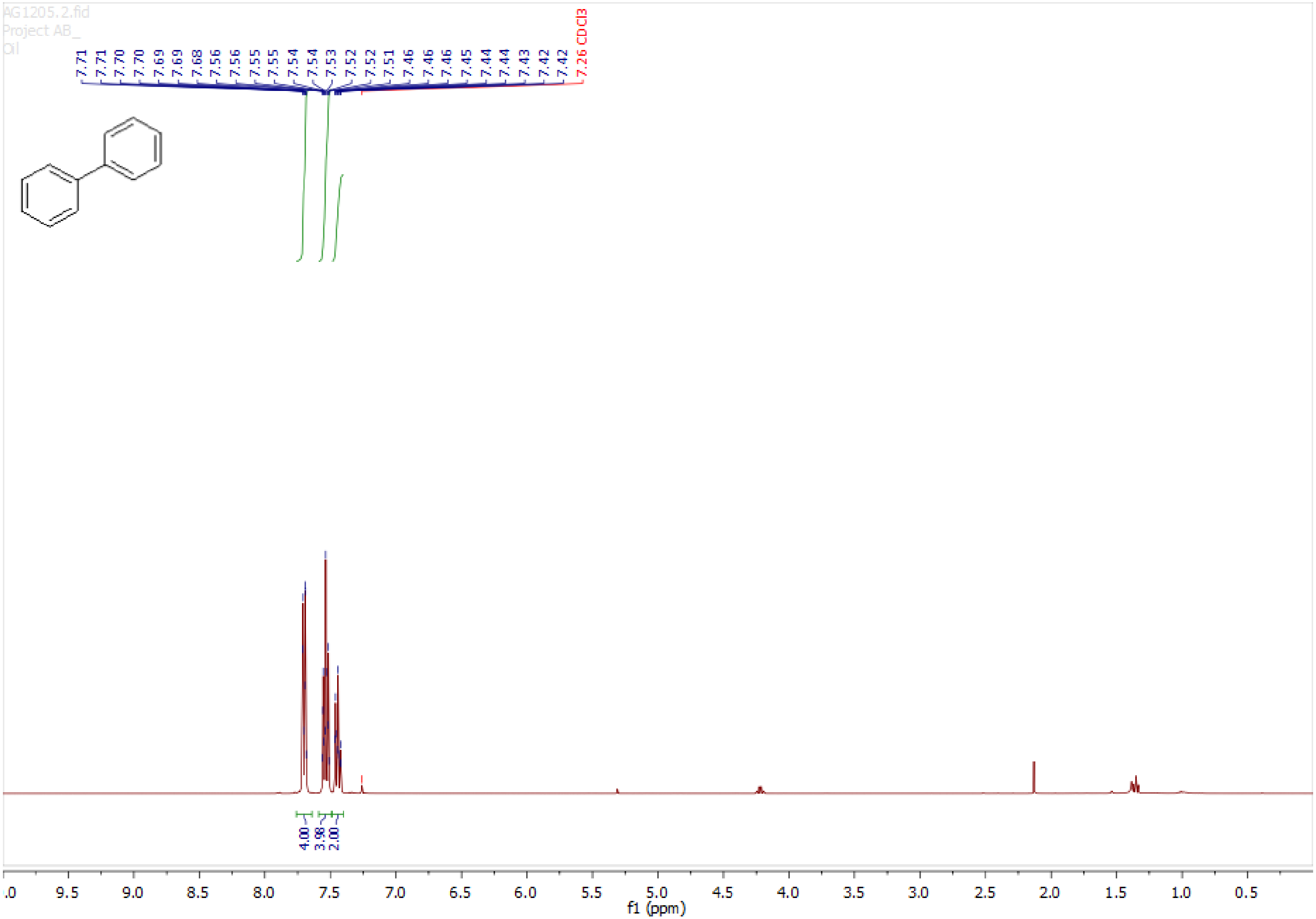

Spectrum S112. The ^{13}C NMR spectrum of *5c* in $CDCl_3$.

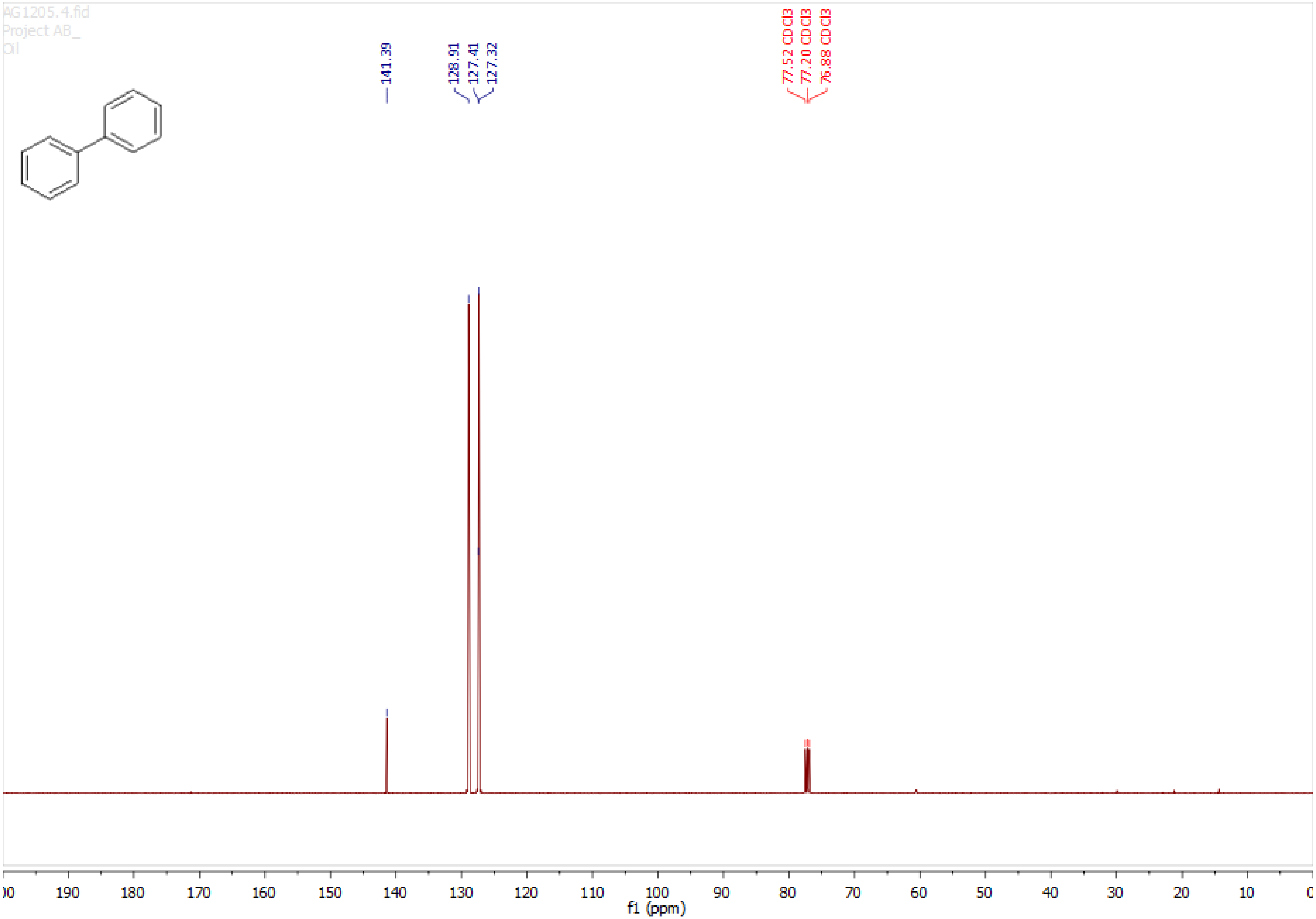

Spectrum S113. The ^{1}H NMR spectrum of *5d* in $CDCl_3$.

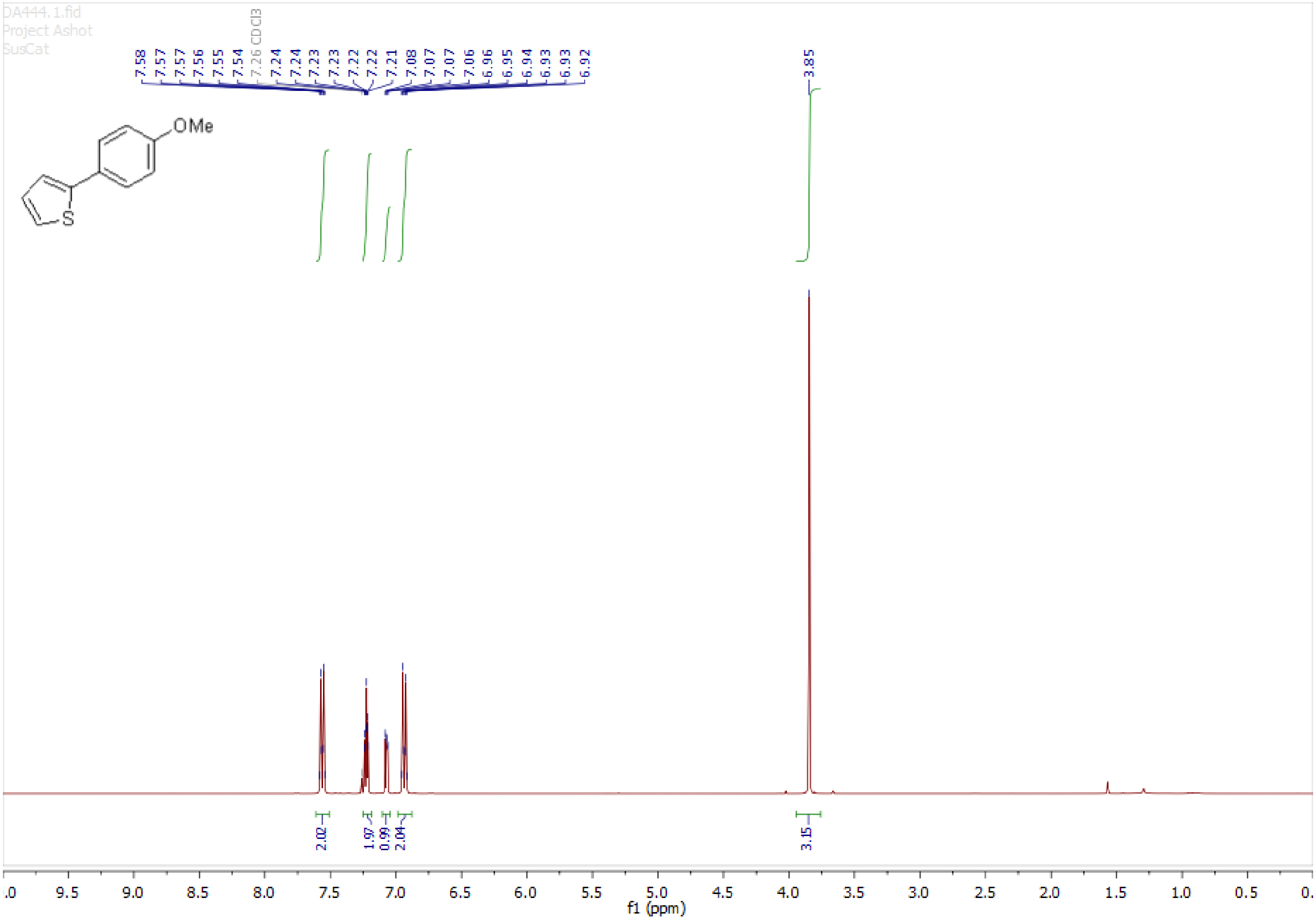

Spectrum S114. The ^{13}C NMR spectrum of *5d* in $CDCl_3$.

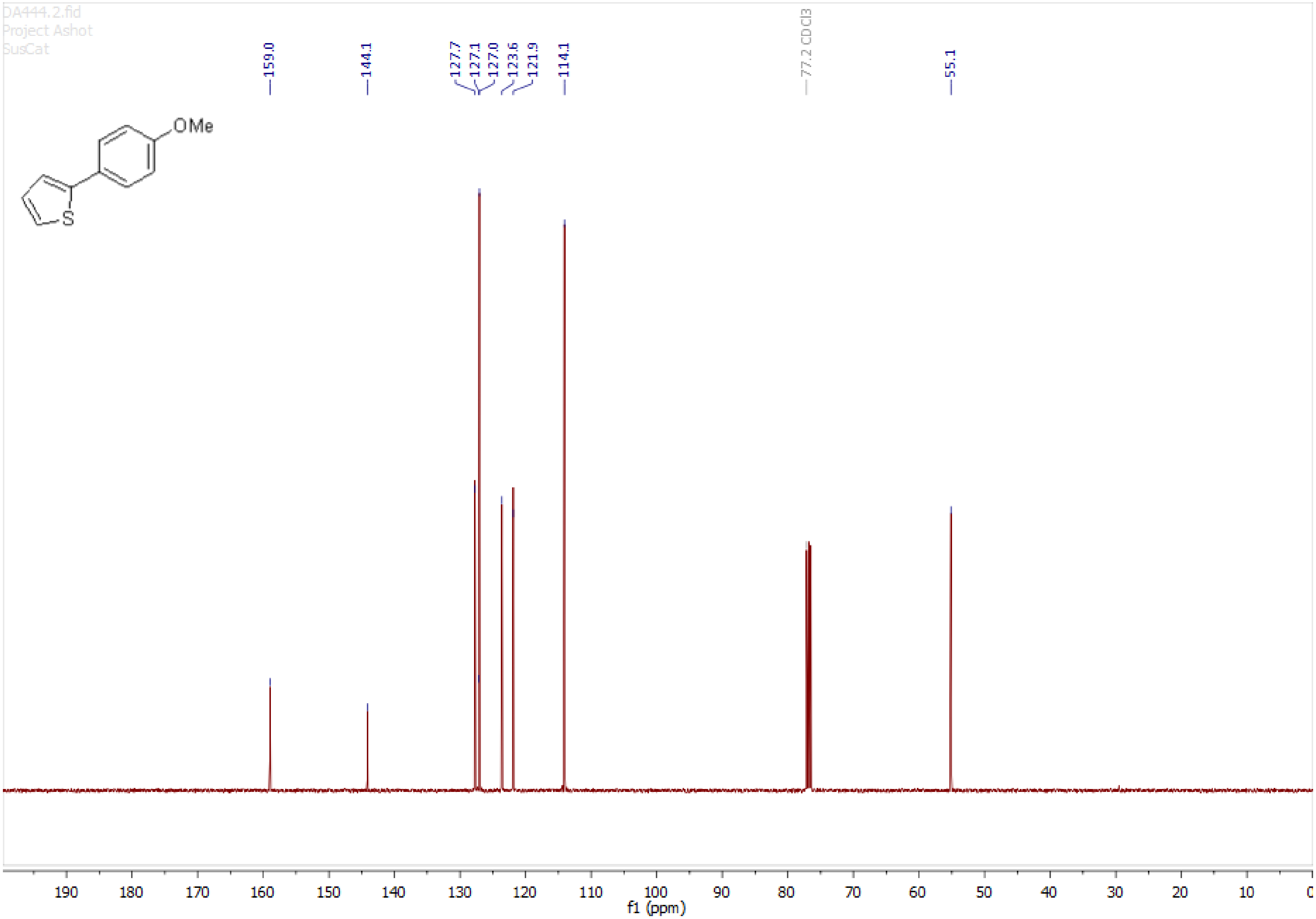

Spectrum S115. The ^{1}H NMR spectrum of *7a* in $CDCl_3$.

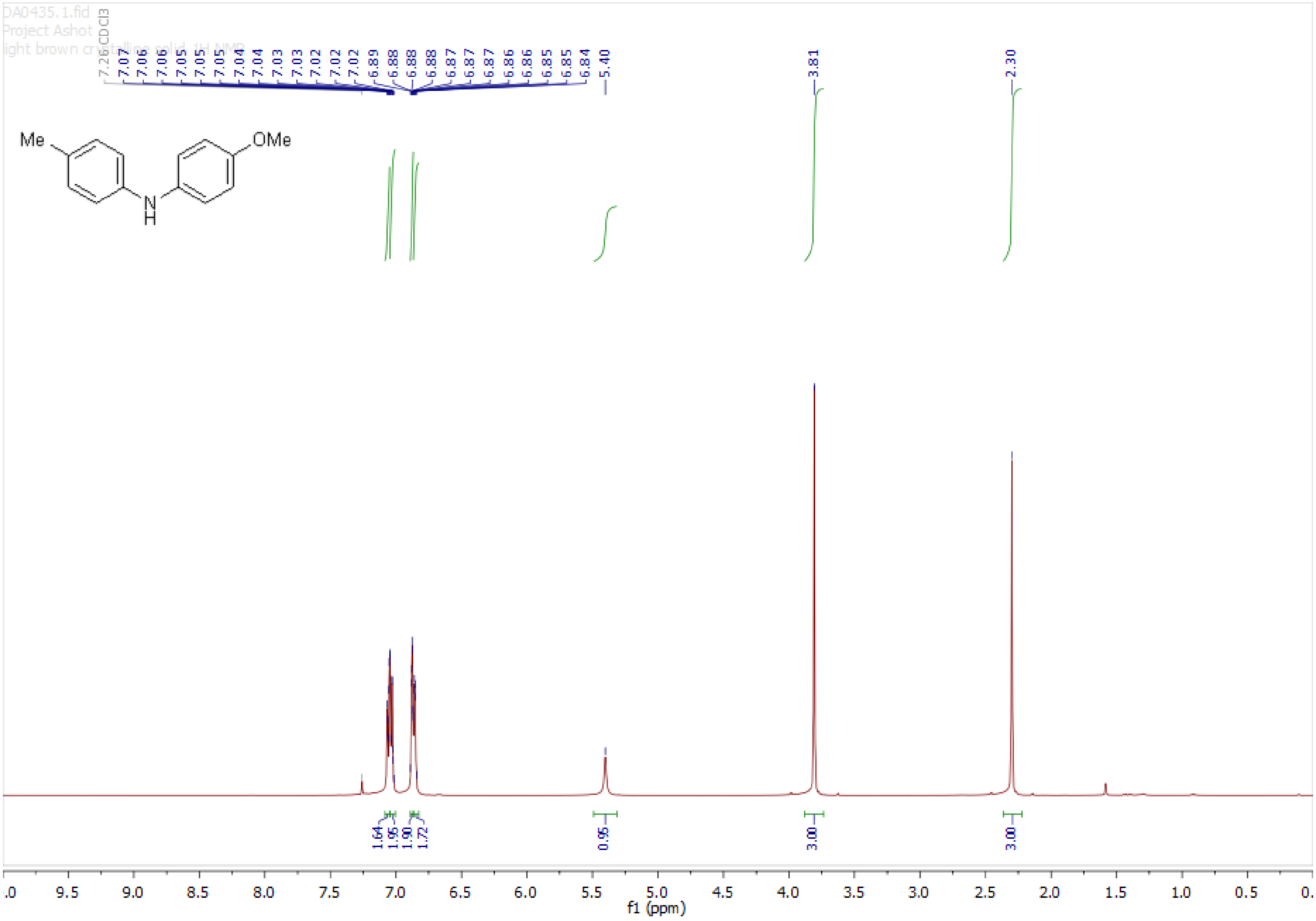

Spectrum S116. The ^{13}C NMR spectrum of *7a* in $CDCl_3$.

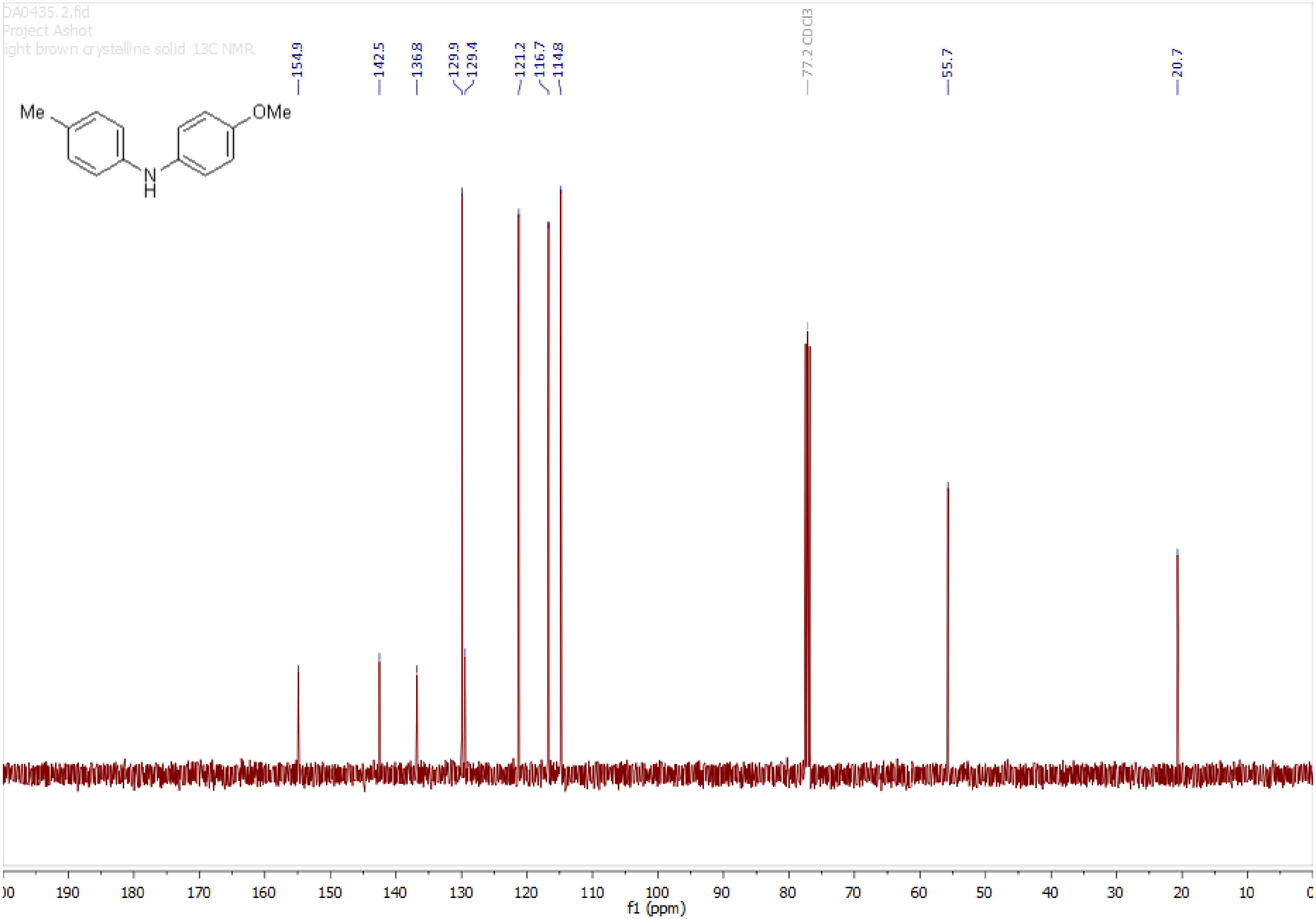

Spectrum S117. The ^1H NMR spectrum of *7b* in $CDCl_3$.

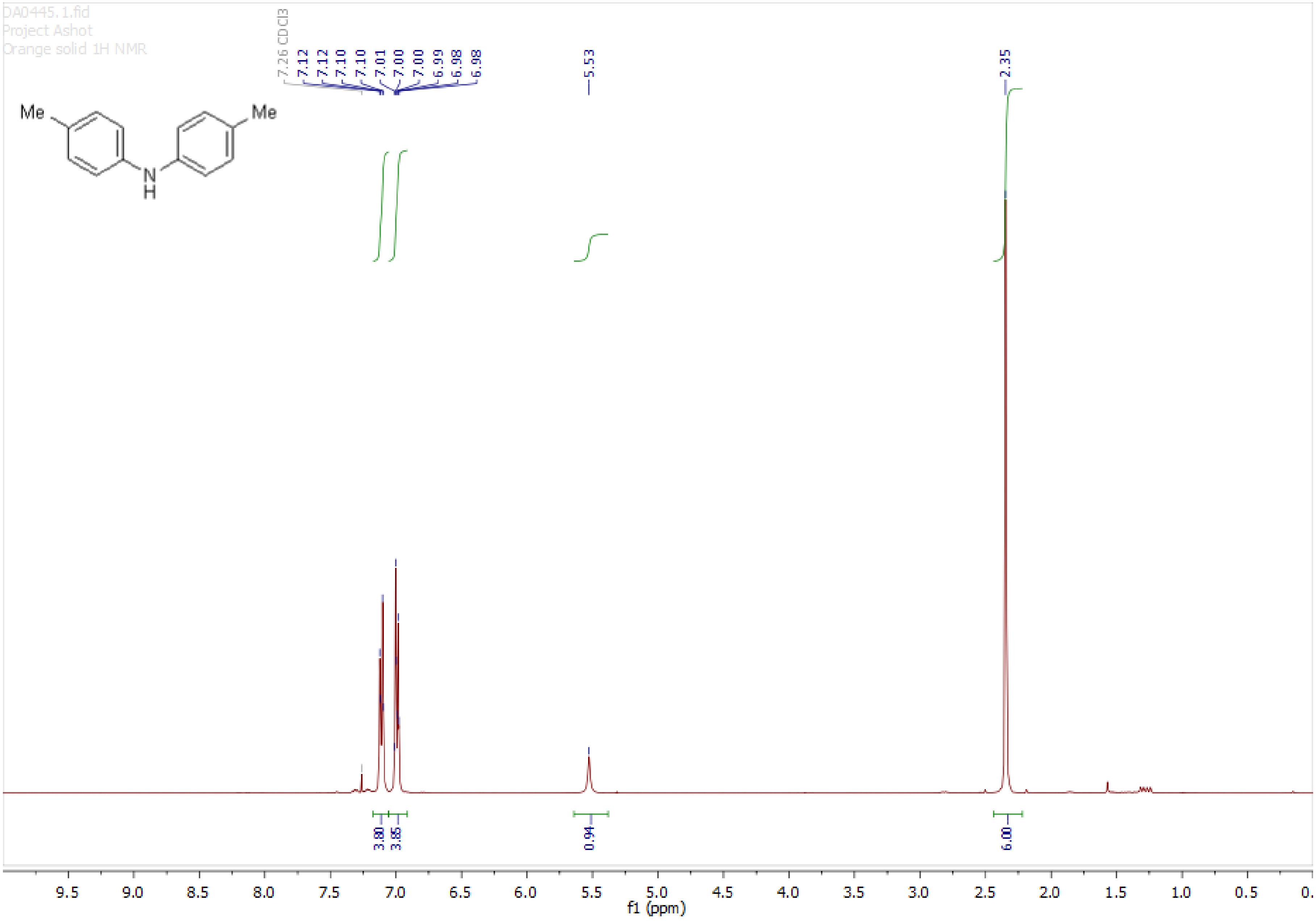

Spectrum S118. The ^{13}C NMR spectrum of *7b* in $CDCl_3$.

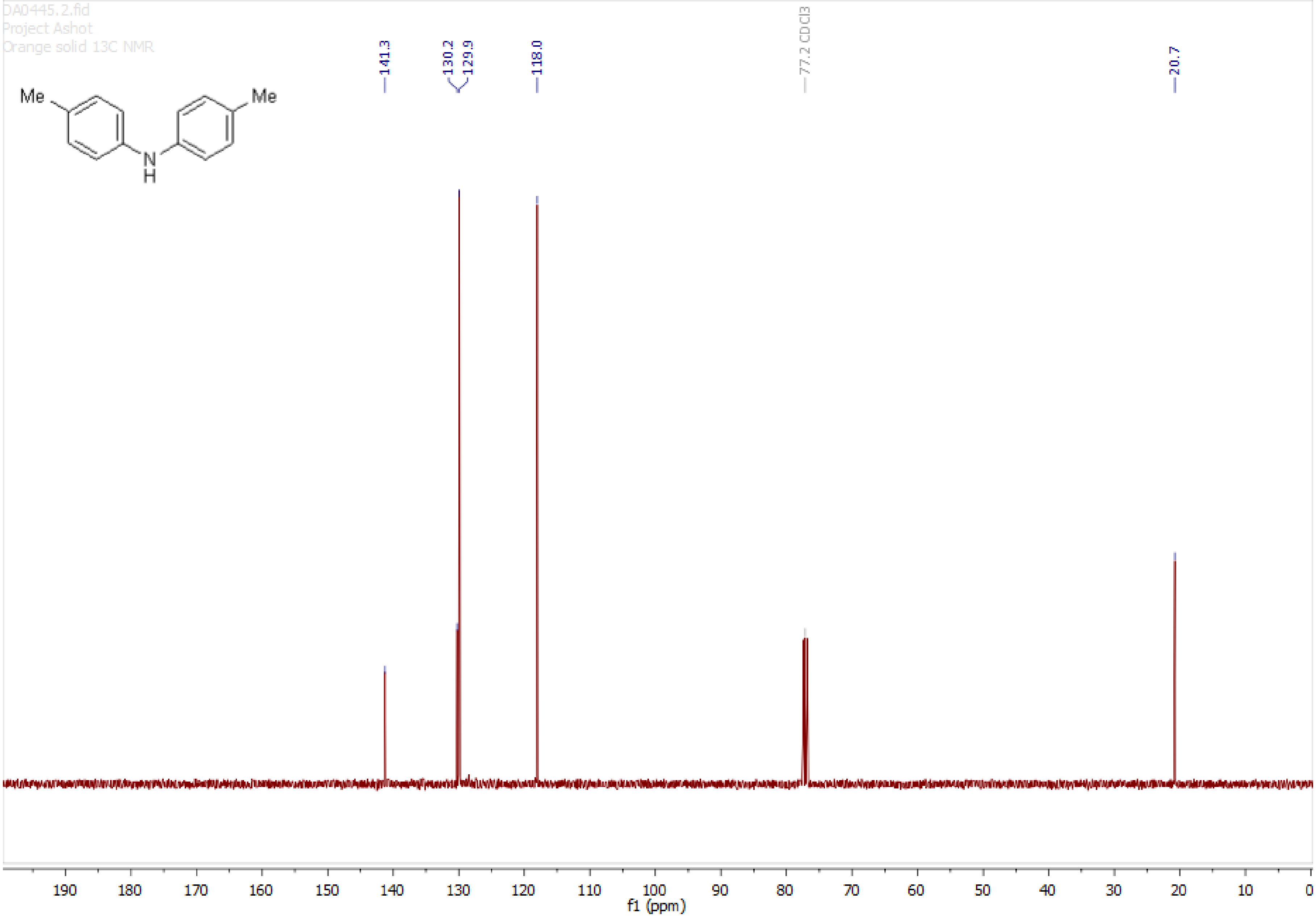

Spectrum S119. The ^1H NMR spectrum of *7c* in $CDCl_3$.

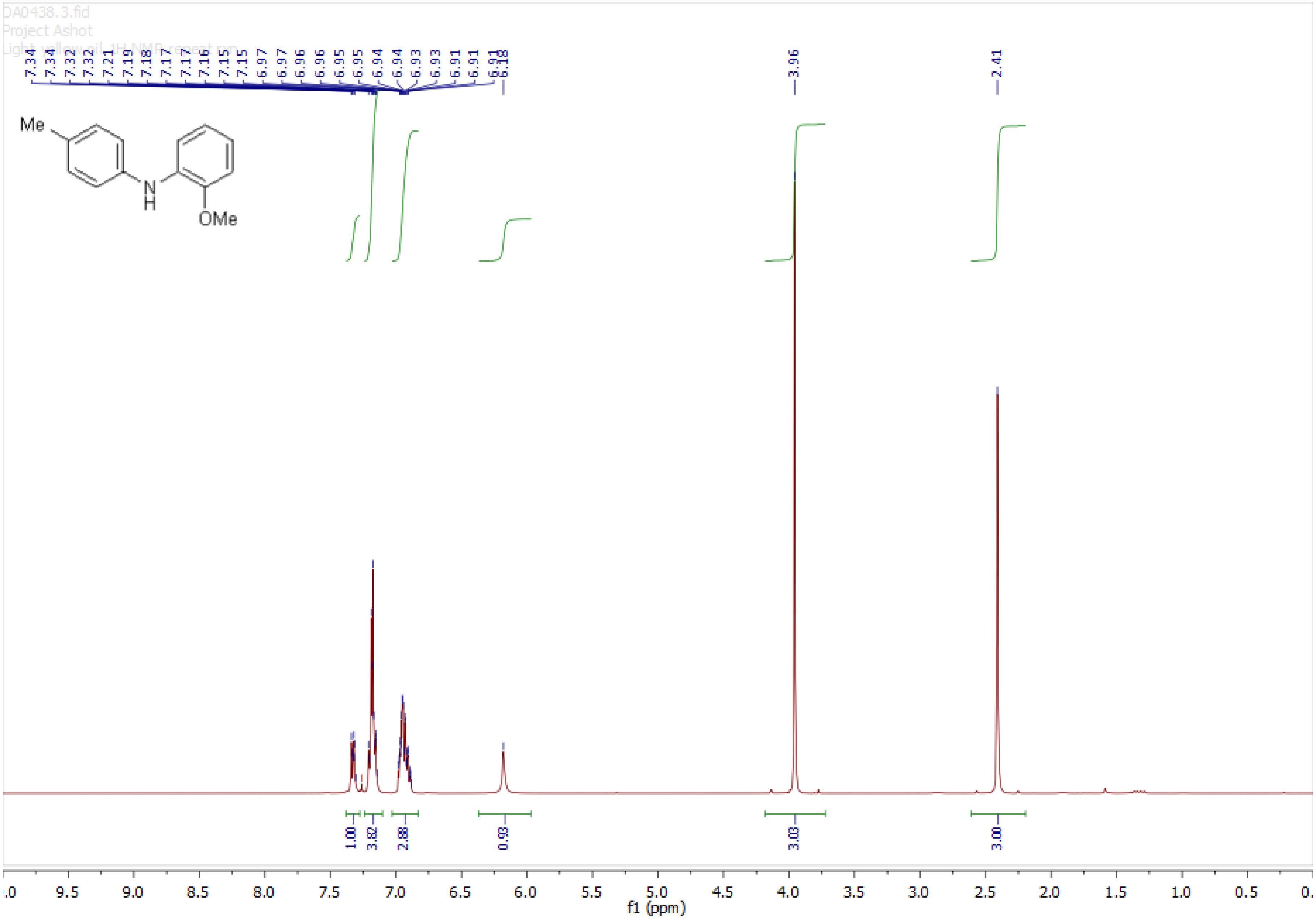

Spectrum S120. The ^{13}C NMR spectrum of *7c* in $CDCl_3$.

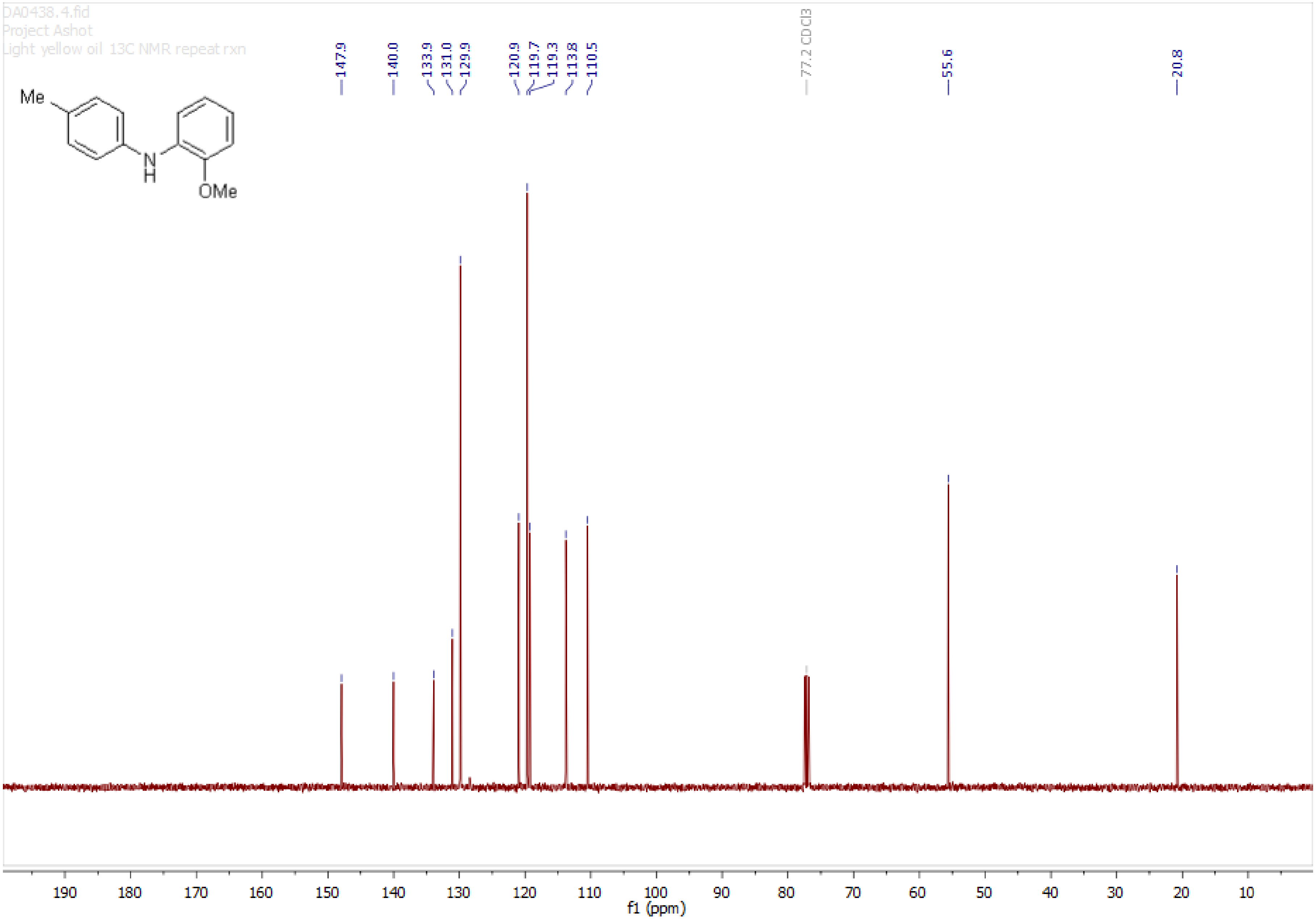

Spectrum S121. The ^{1}H NMR spectrum of *7d* in $CDCl_3$.

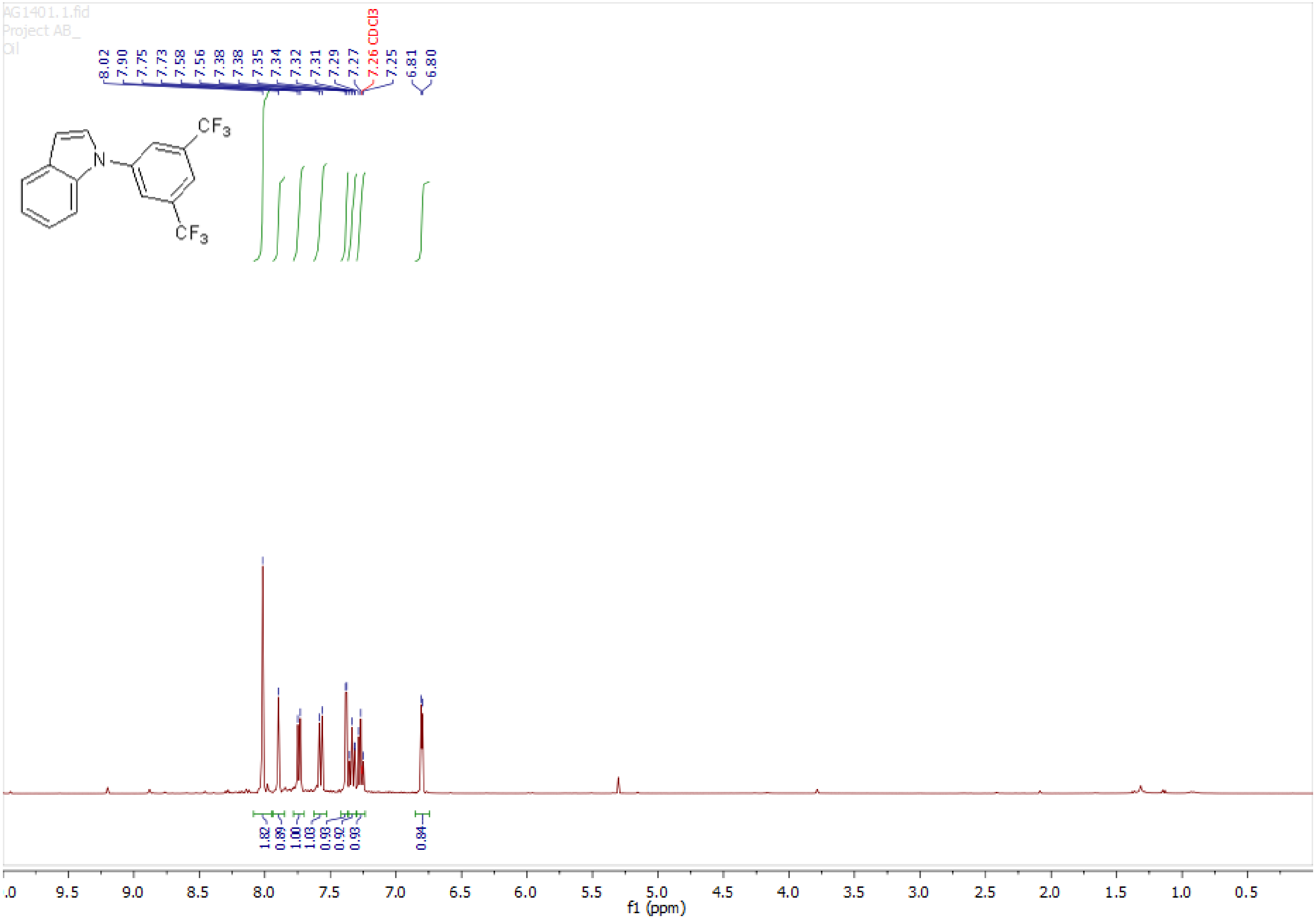

Spectrum S122. The ^{13}C NMR spectrum of *7d* in $CDCl_3$.

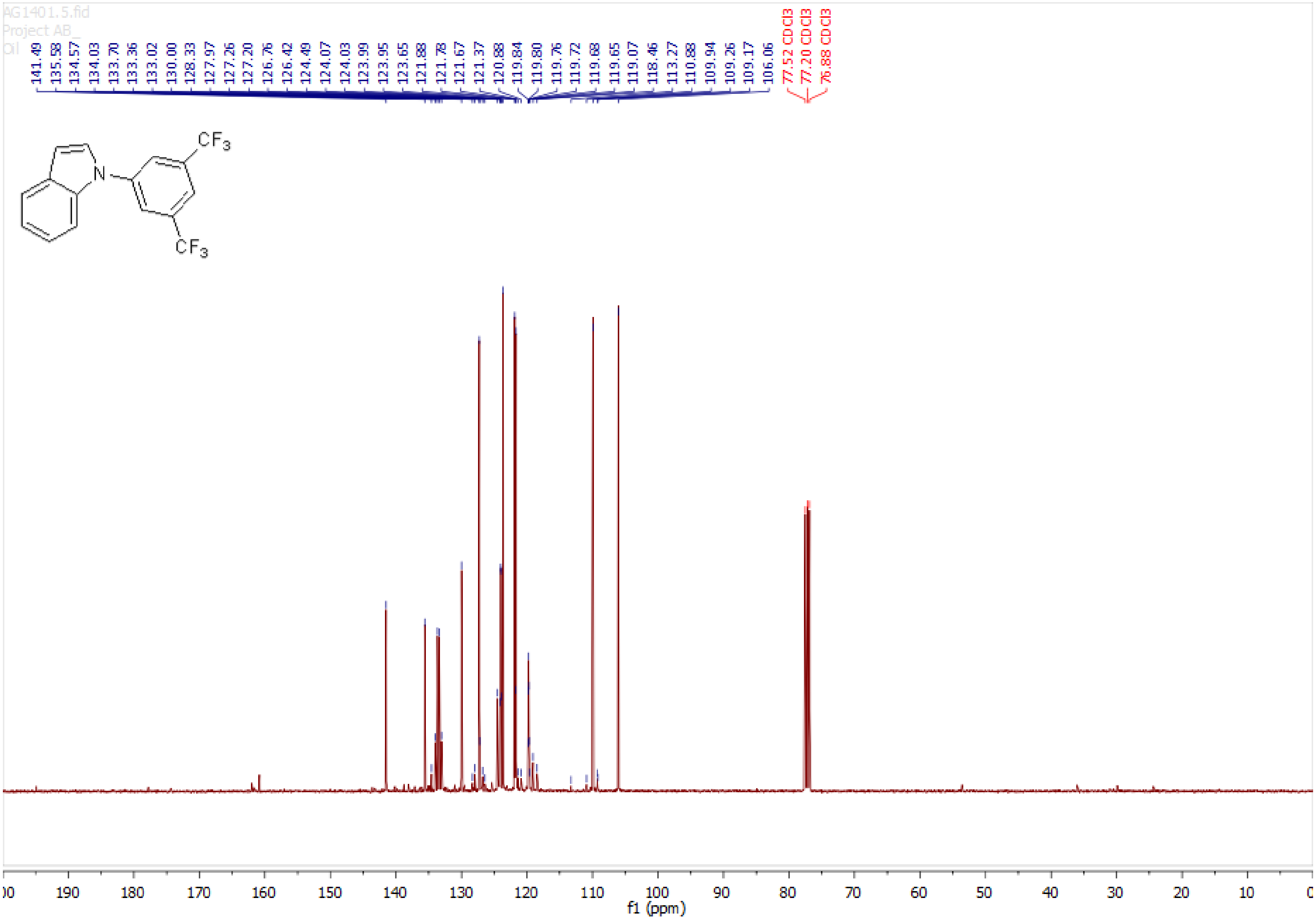

Spectrum S123. The ^{19}F NMR spectrum of *7d* in $CDCl_3$.

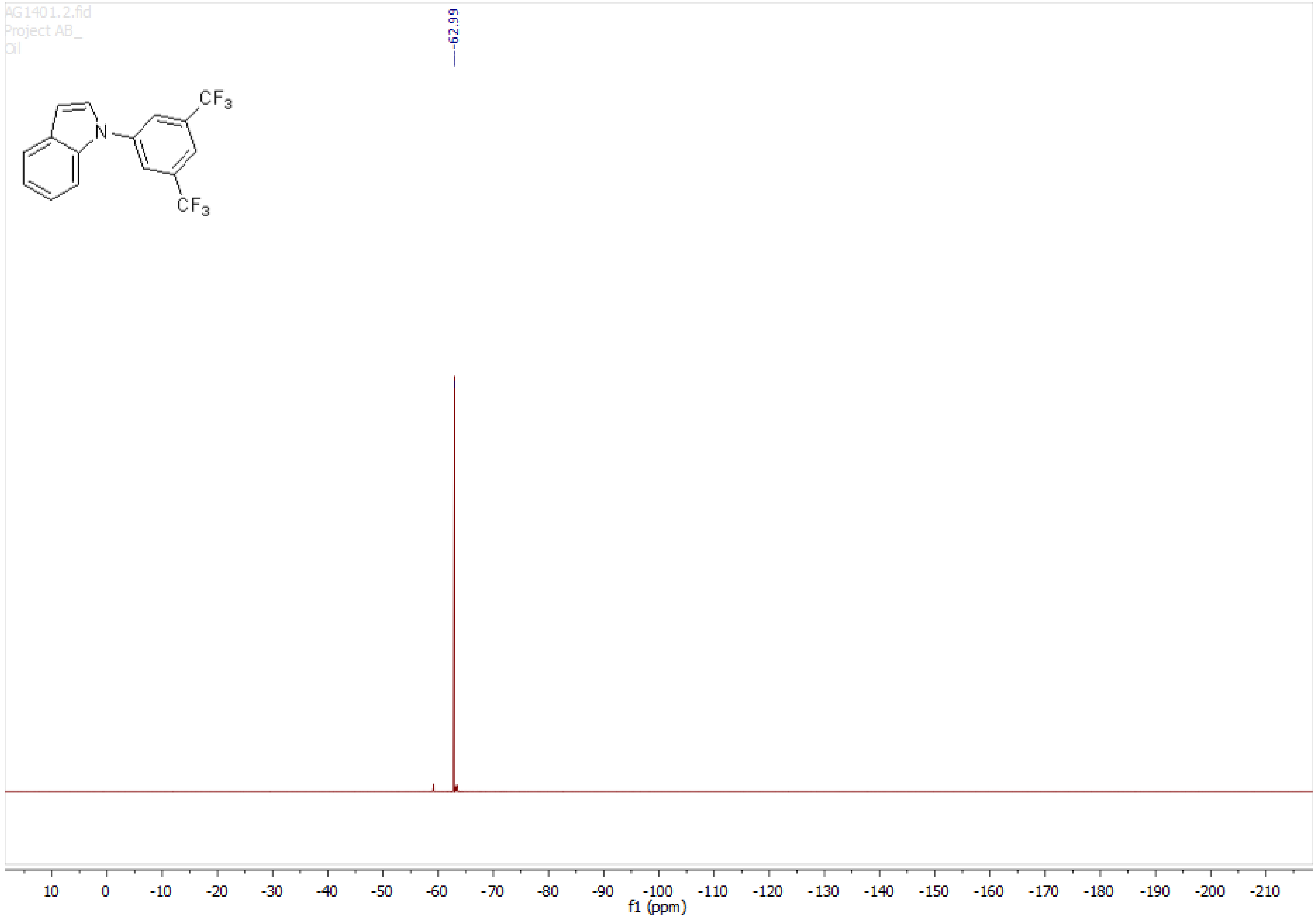

Spectrum S124. The ^{1}H NMR spectrum of *8a* in $CDCl_3$.

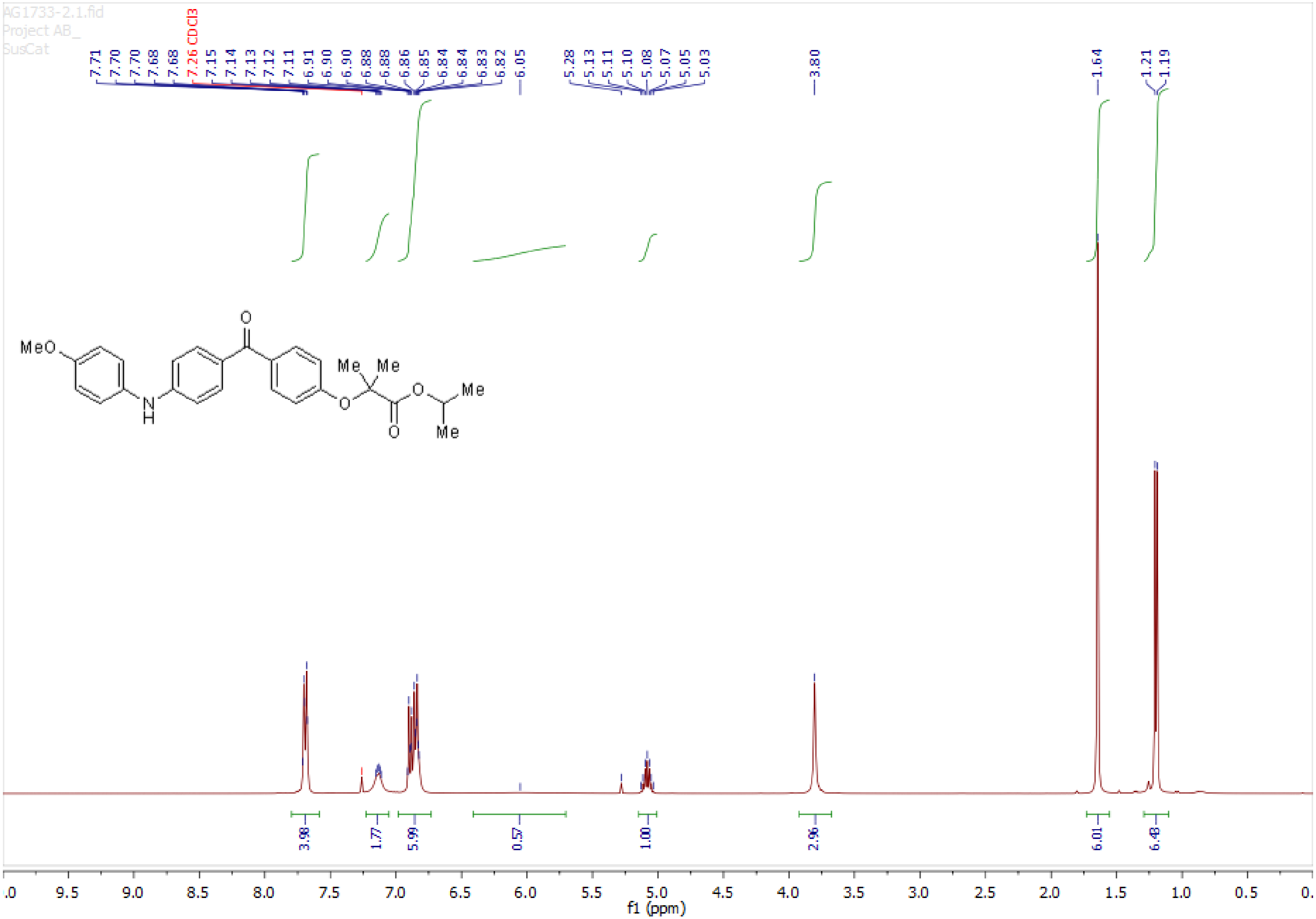

Spectrum S125. The ^{13}C NMR spectrum of *8a* in $CDCl_3$.

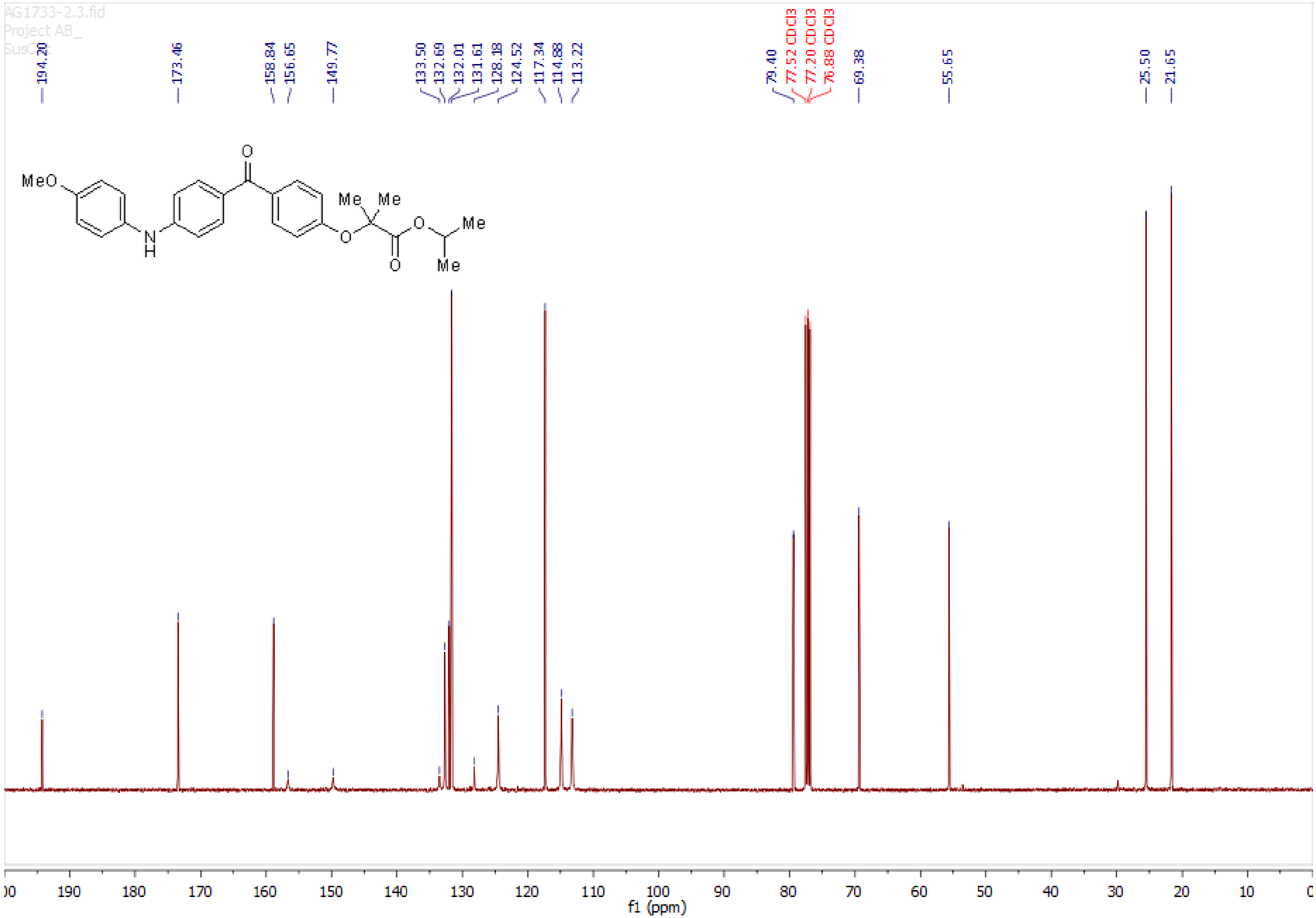

Spectrum S126. The ^{1}H NMR spectrum of *8b* in $CDCl_3$.

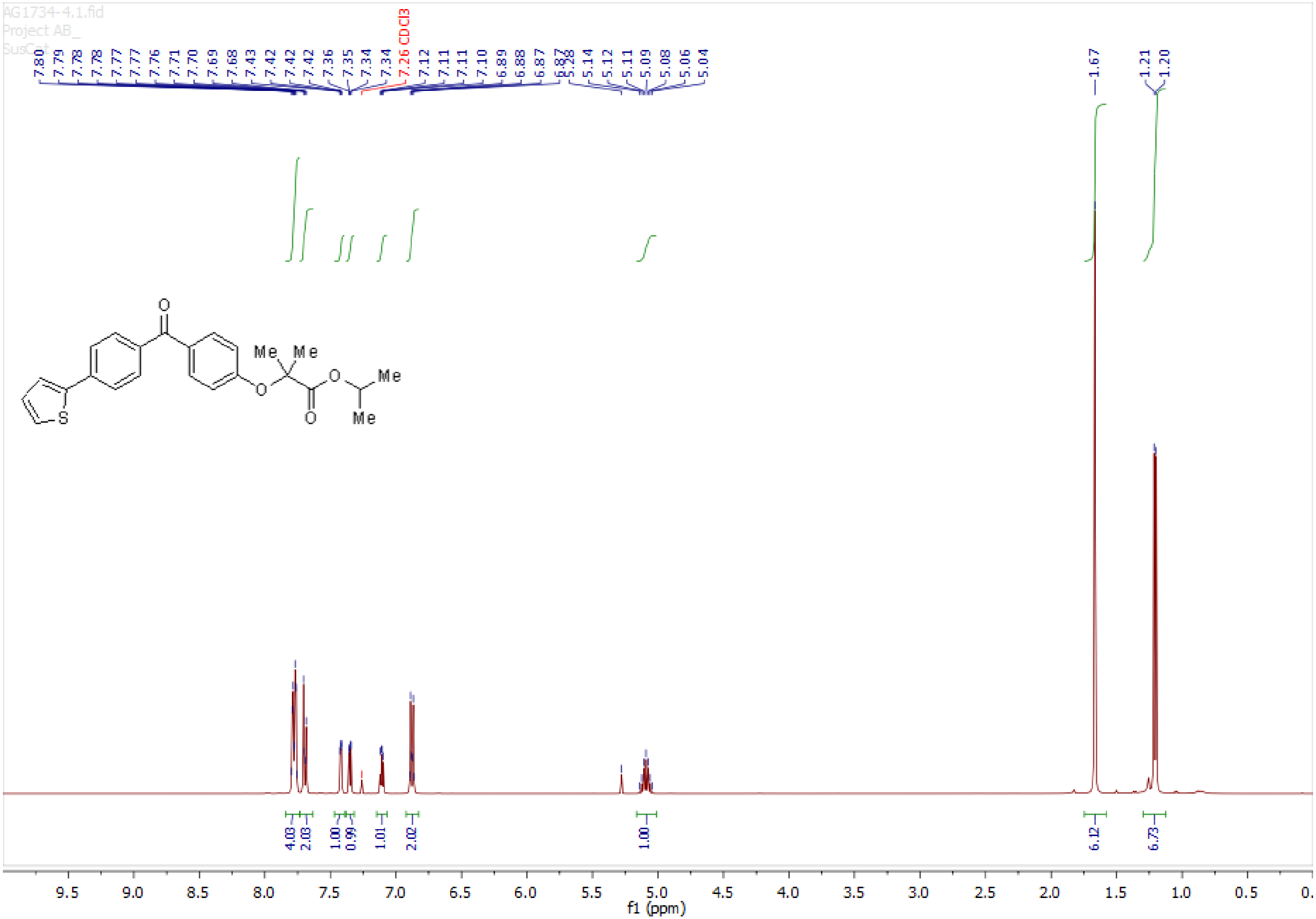

Spectrum S127. The ^{13}C NMR spectrum of *8b* in $CDCl_3$.

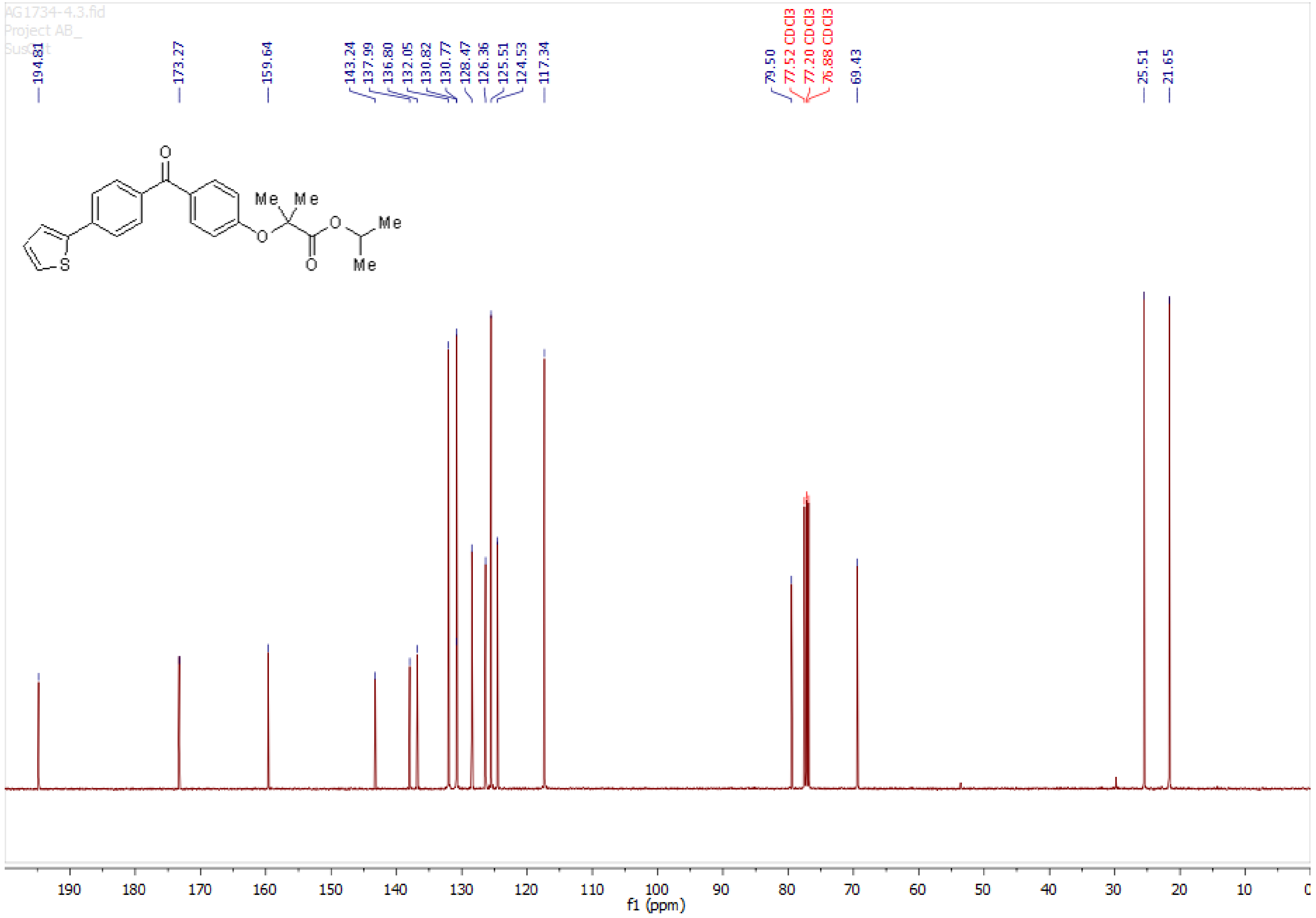

Spectrum S128. The ^{1}H NMR spectrum of *8c* in $CDCl_3$.

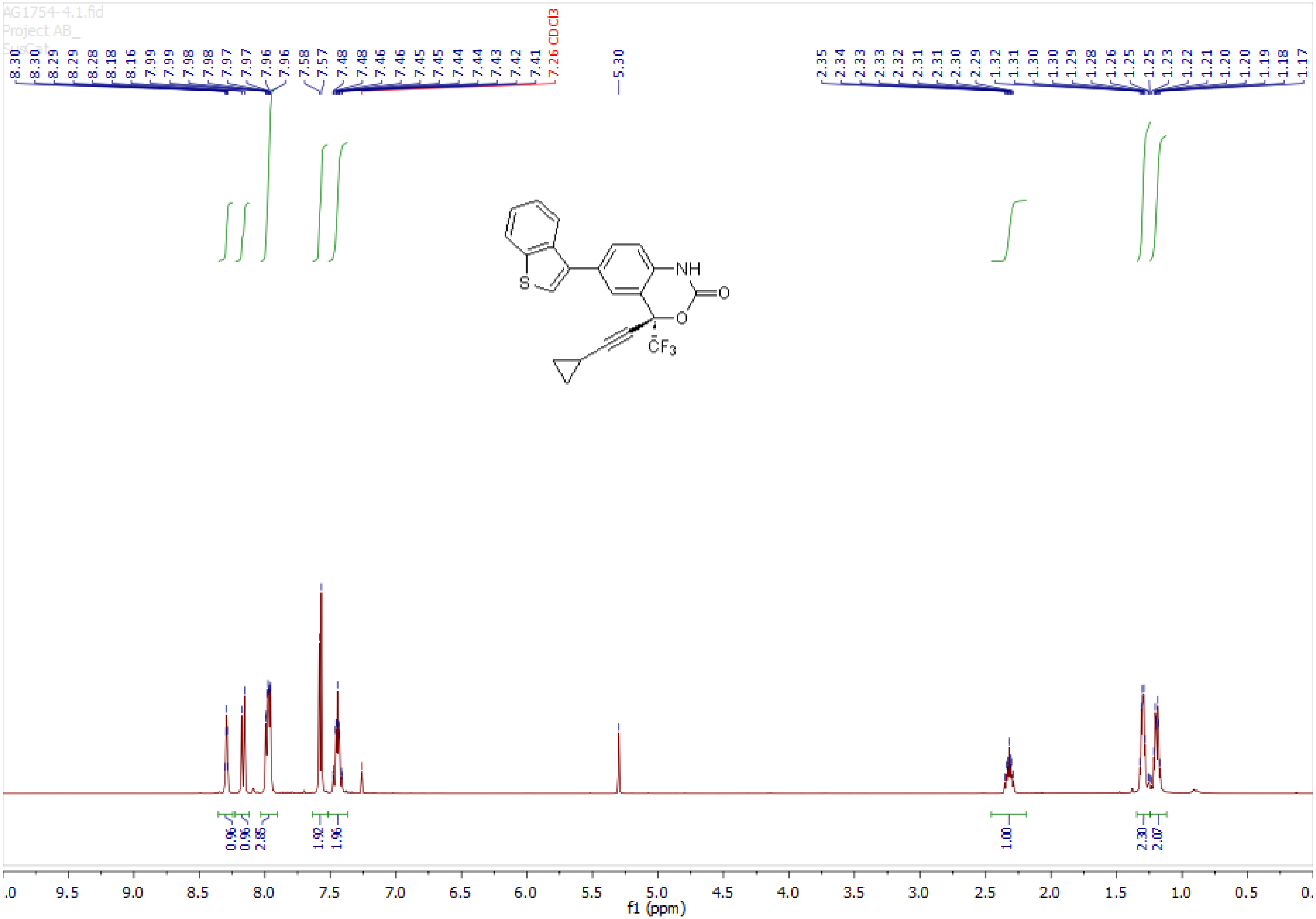

Spectrum S129. The ^{13}C NMR spectrum of *8c* in $CDCl_3$.

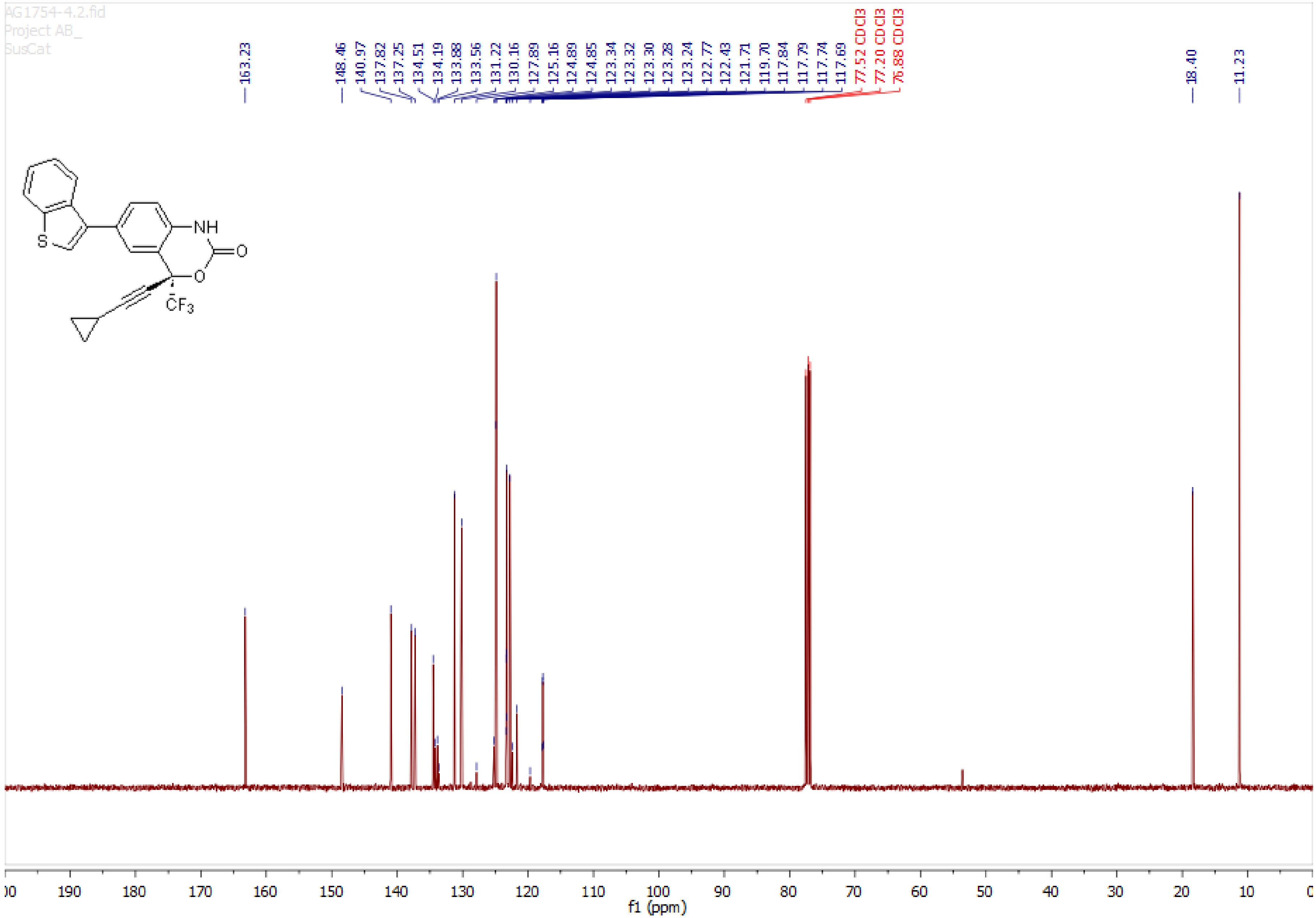

Spectrum S130. The ^{19}F NMR spectrum of *8c* in $CDCl_3$.

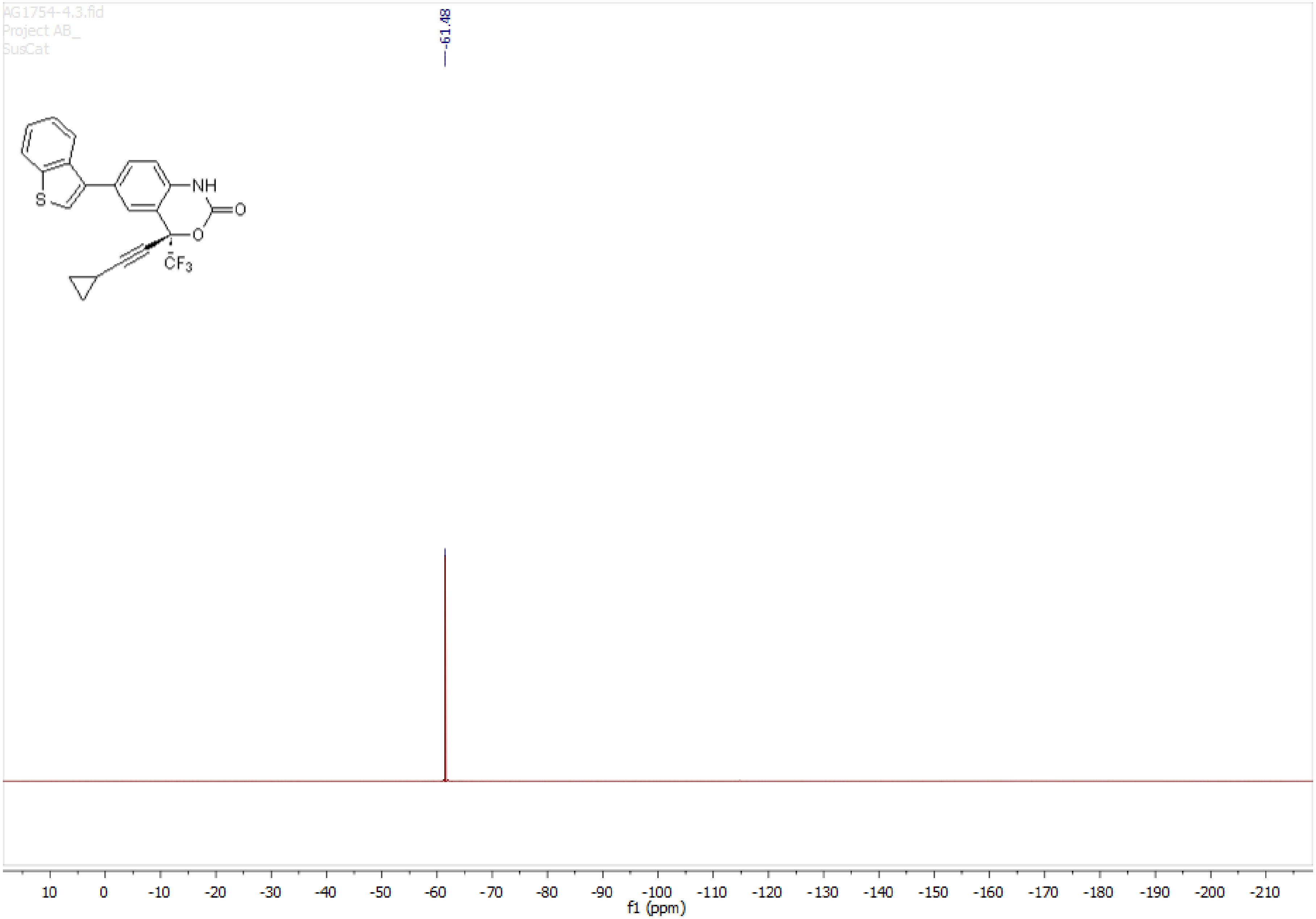

Spectrum S131. The crude ^{1}H NMR spectrum of *8d* in DMSO-d6. 1,3,5-Trimethoxybenzene is used as an internal standard.

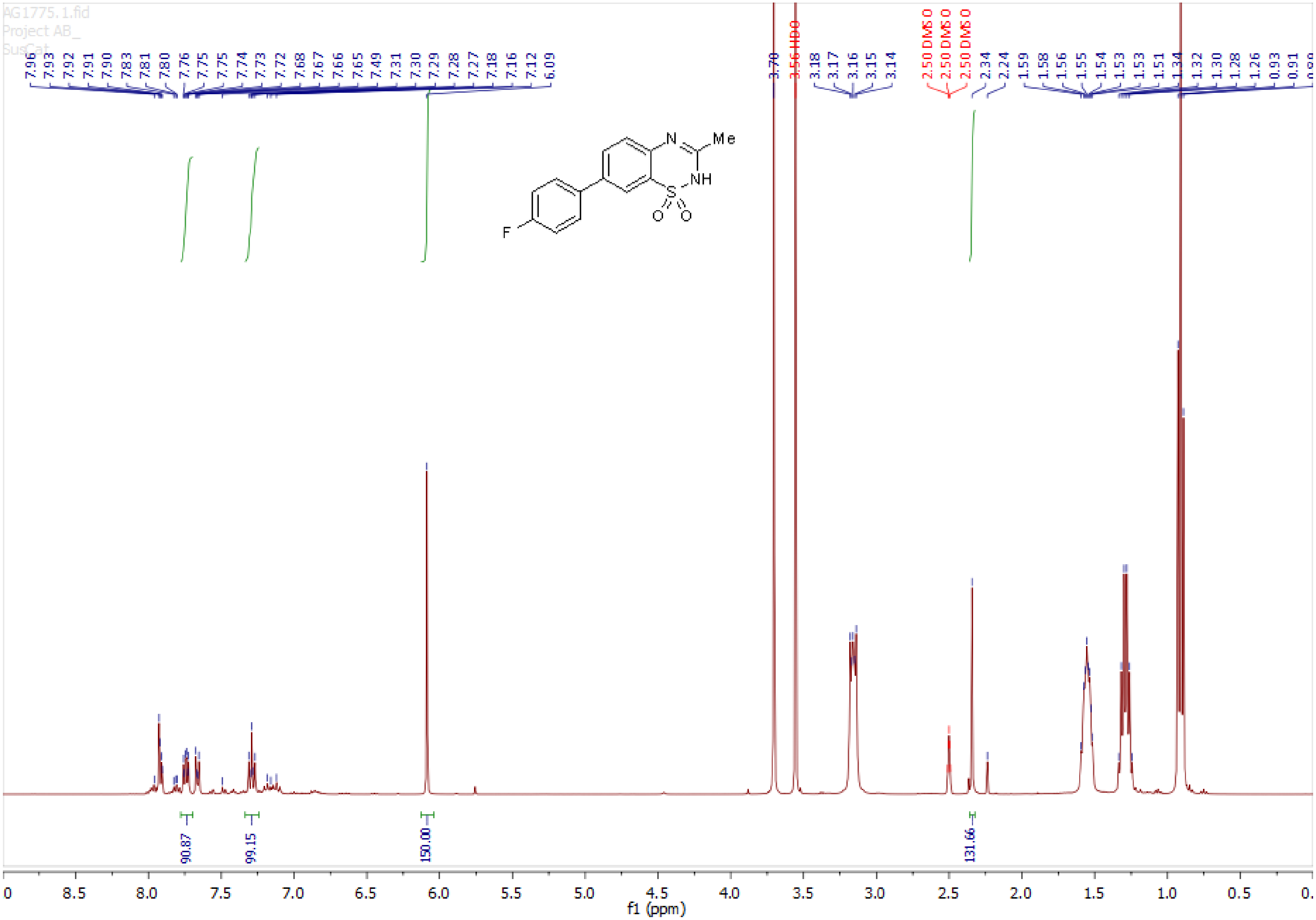

Spectrum S132. The crude ^{13}C NMR spectrum of *8d* in DMSO-d6. 1,3,5-Trimethoxybenzene is used as an internal standard.

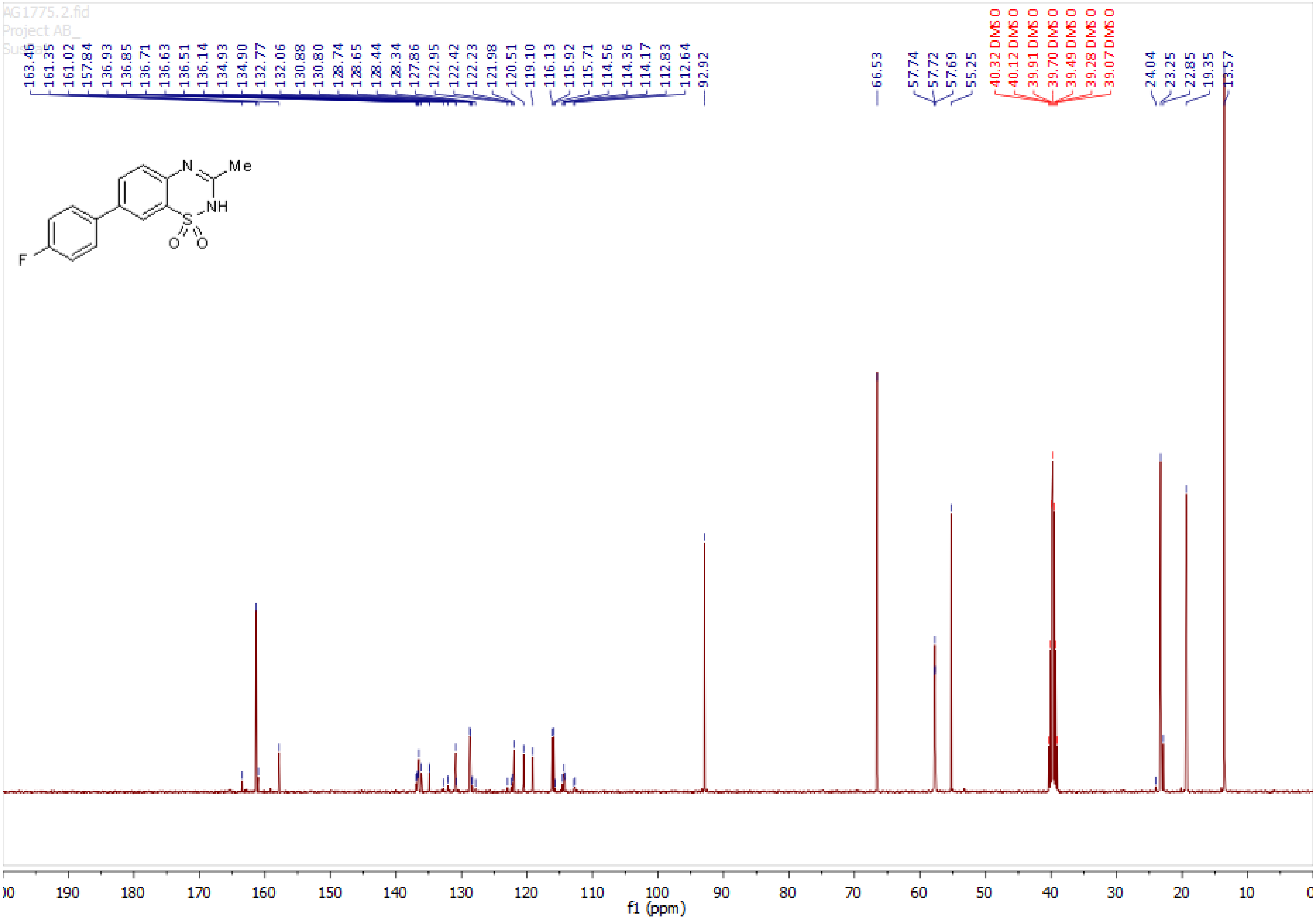

Spectrum S133. The crude ^{19}F NMR spectrum of *8d* in DMSO-d6. 1,3,5-Trimethoxybenzene is used as an internal standard.

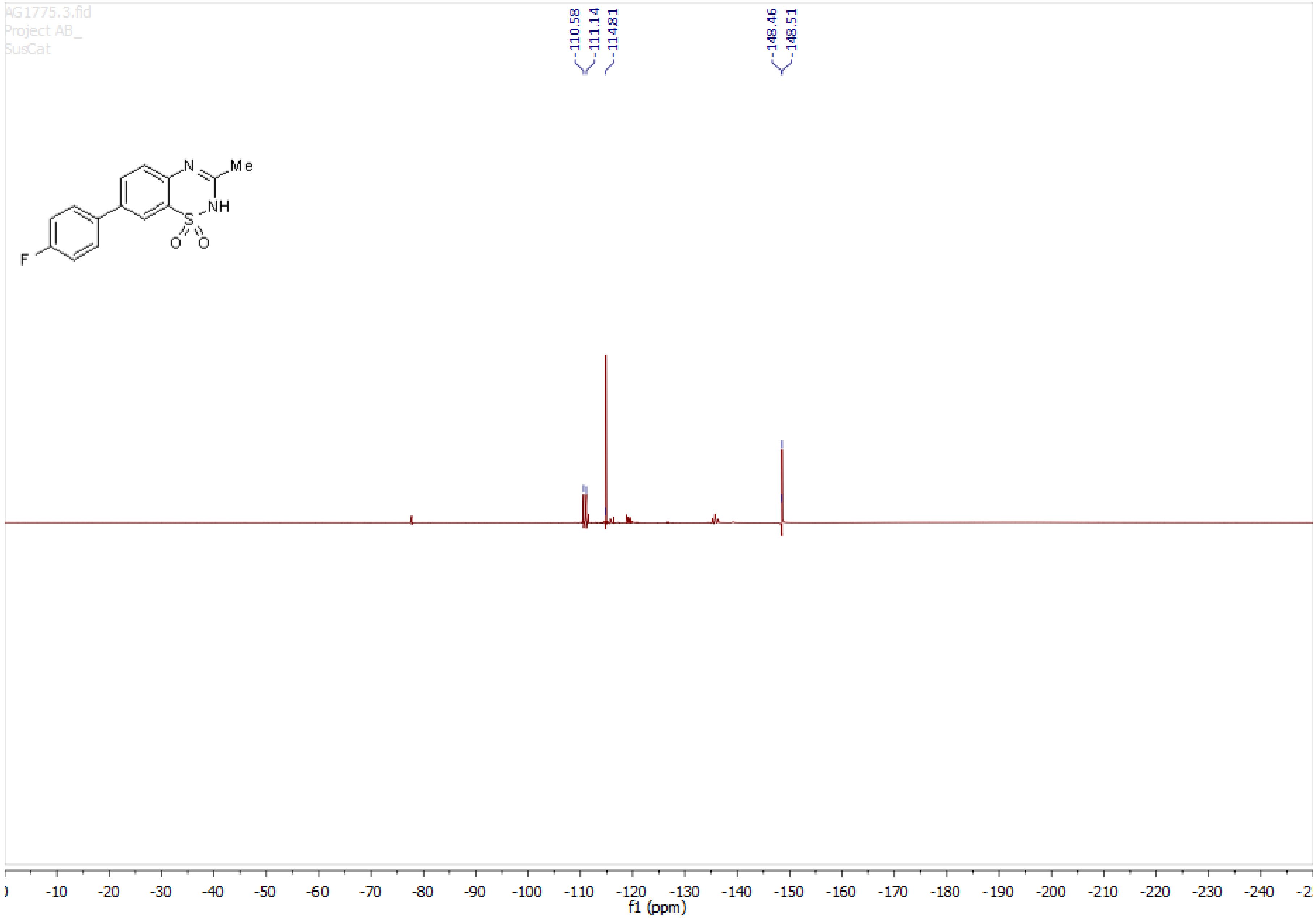

Spectrum S134. The ^{1}H NMR spectrum of *diazoxide* in DMSO-d6 for a reference.

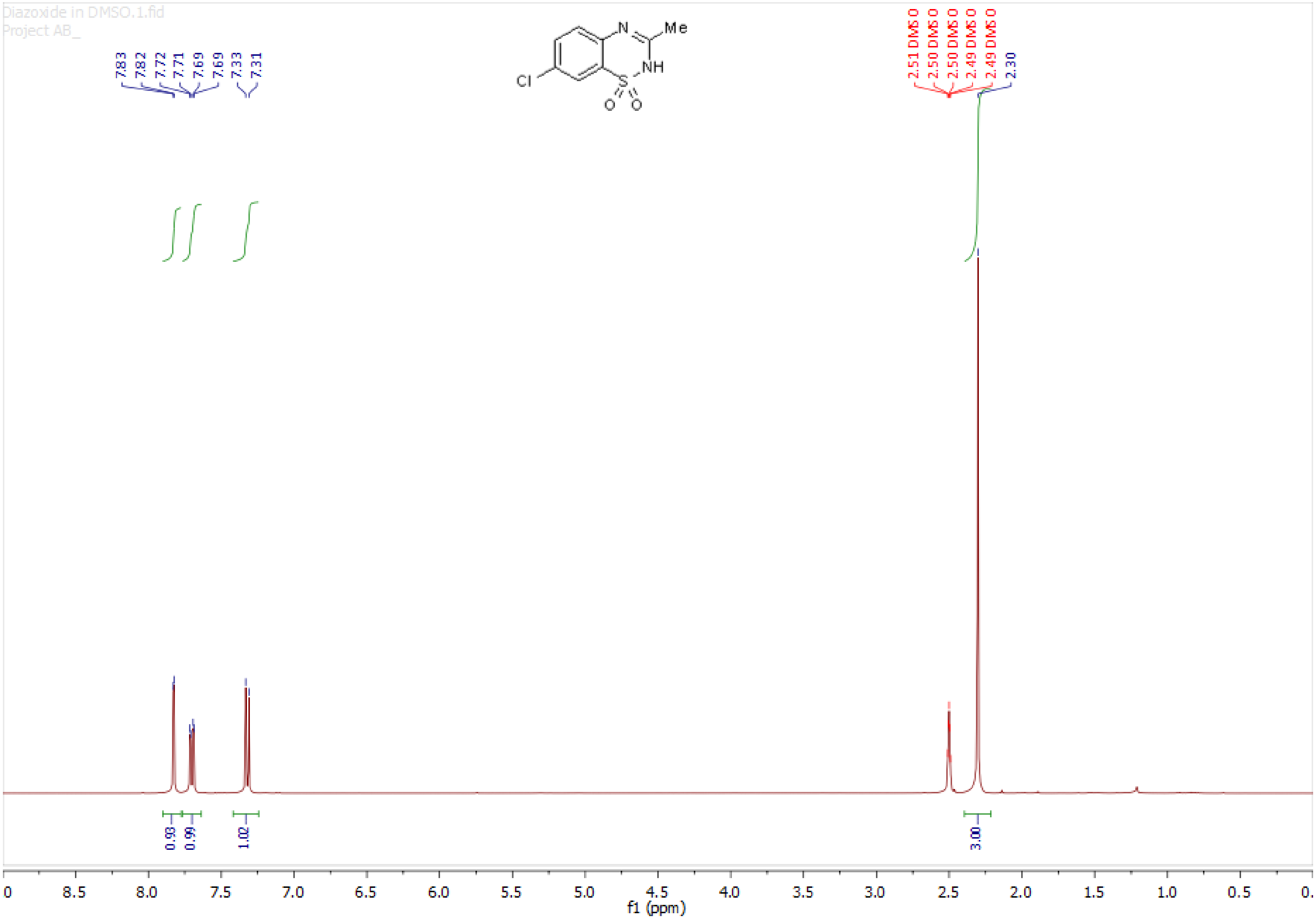

Spectrum S135. The ^{13}C NMR spectrum of *diazoxide* in DMSO-d6 for a reference.

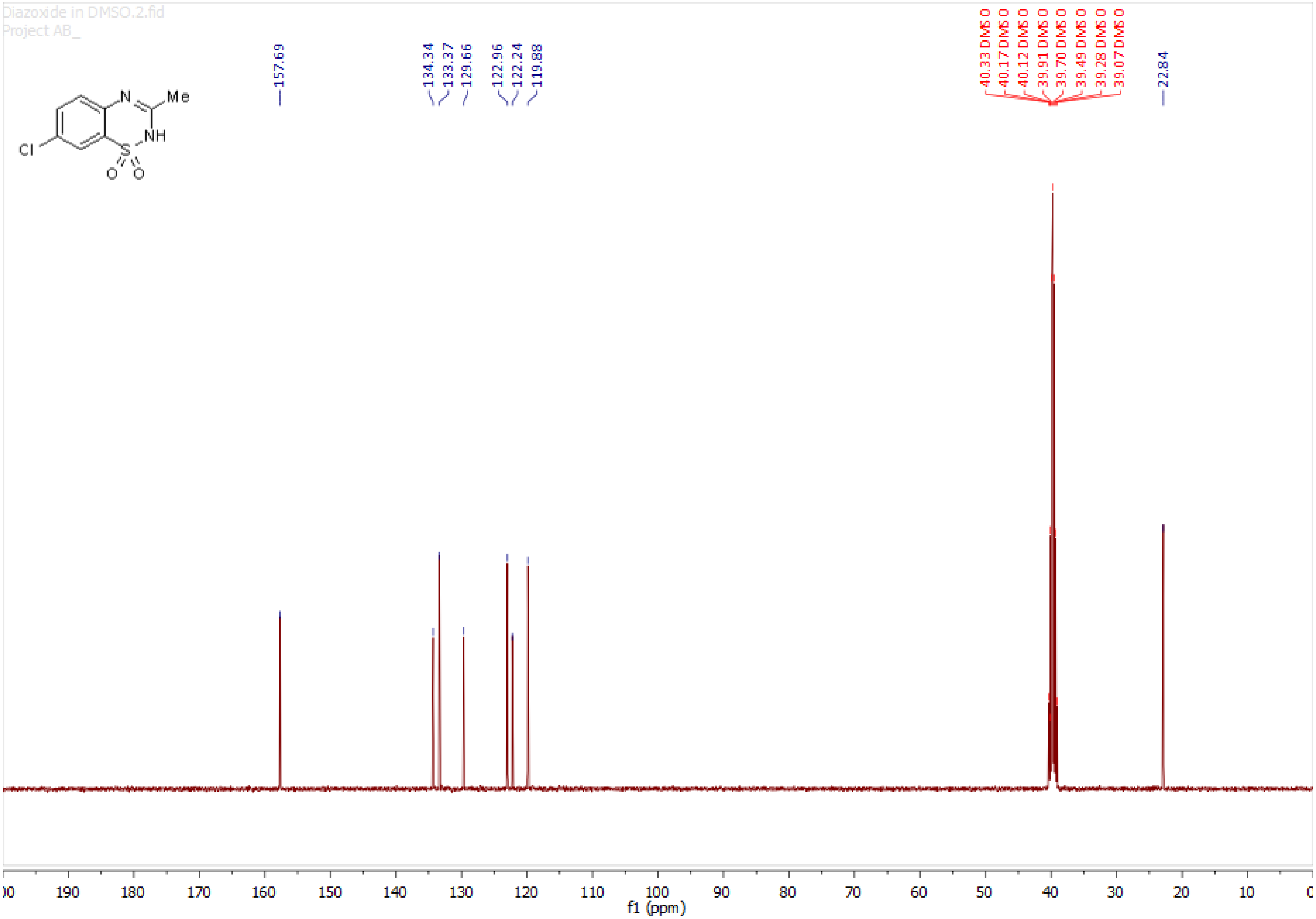

Spectrum S136. The ^{1}H NMR spectrum of *8e* in DMSO-d6.

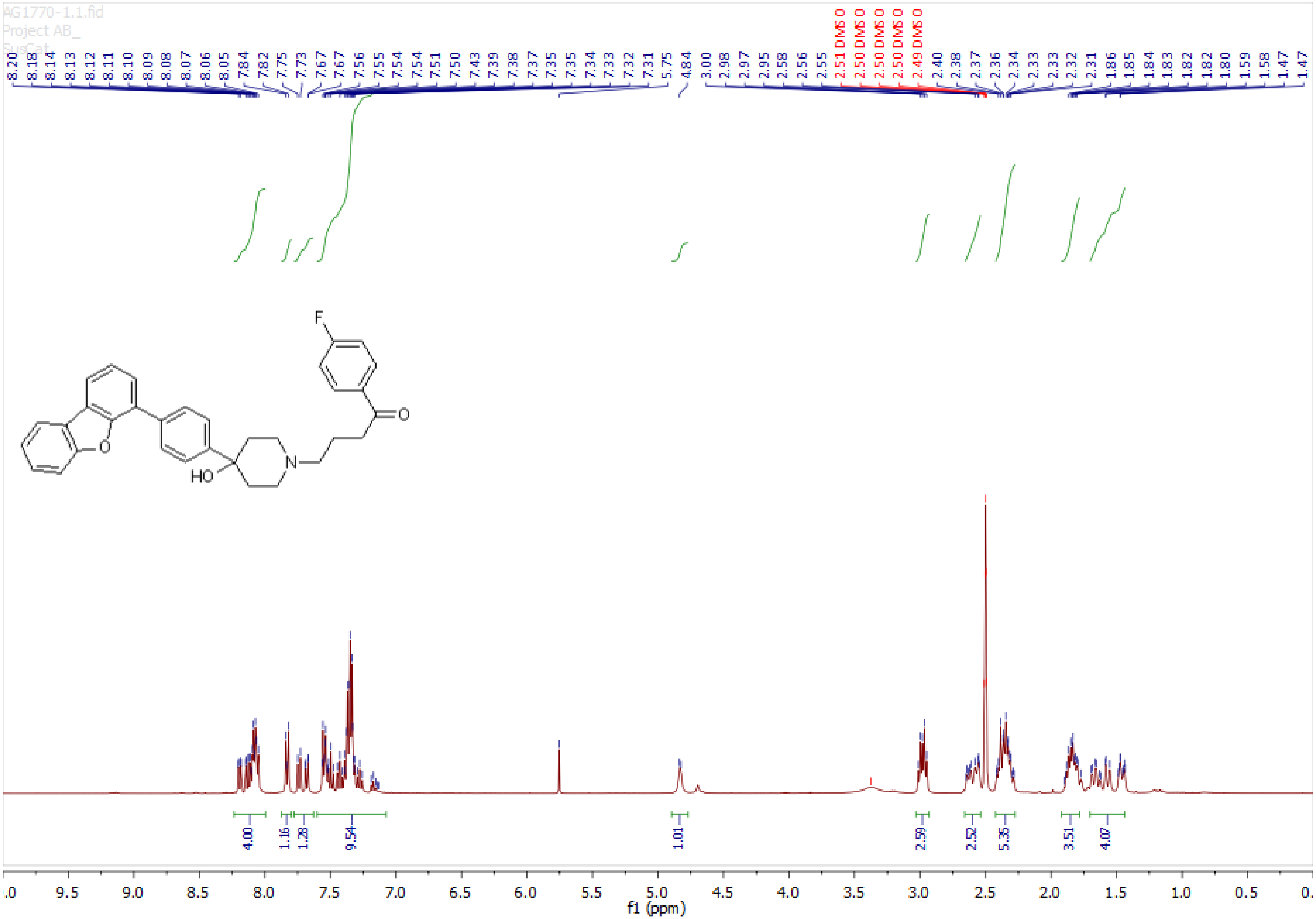

Spectrum S137. The ^{13}C NMR spectrum of *8e* in DMSO-d6.

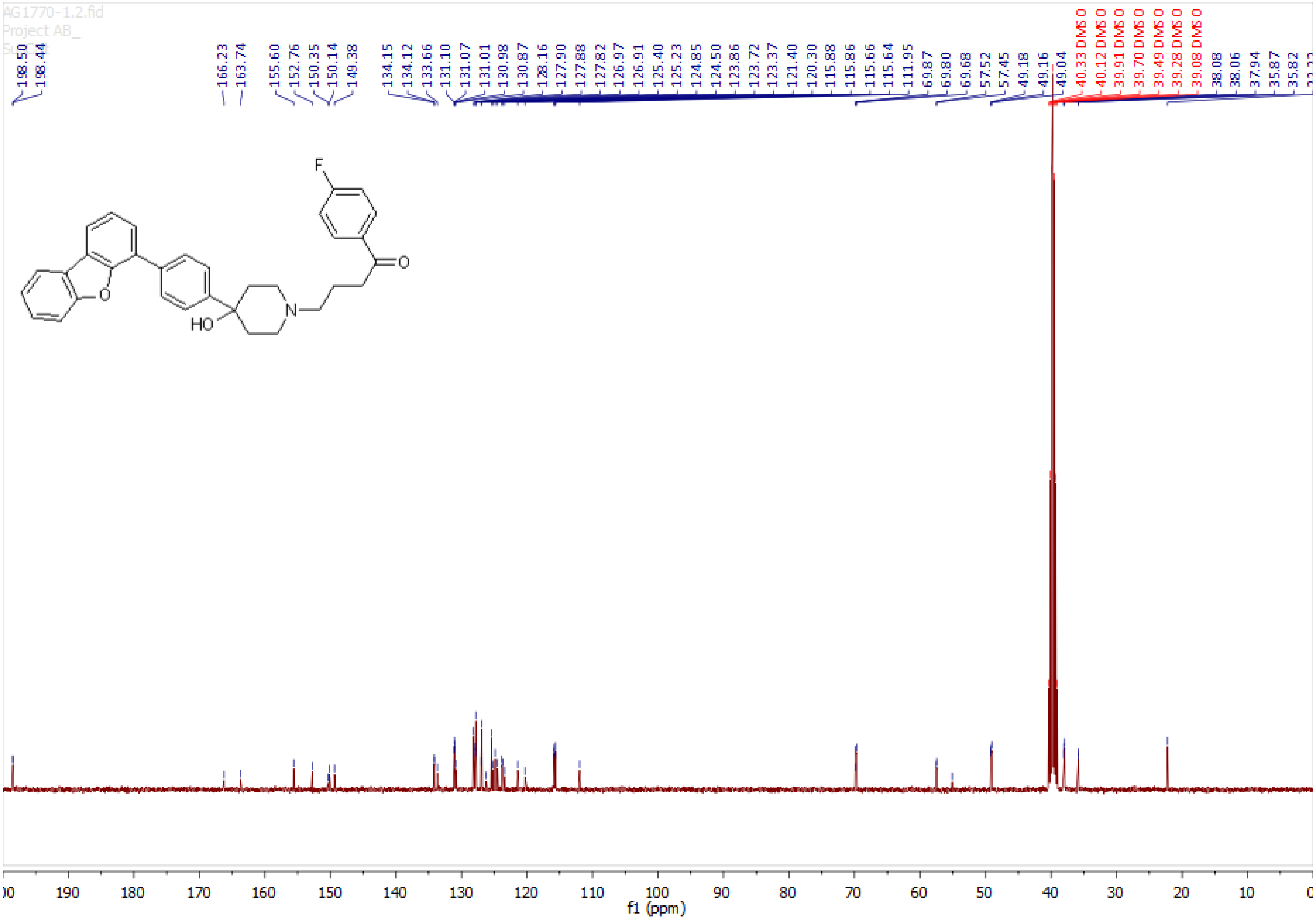

Spectrum S138. The ^{19}F NMR spectrum of *8e* in DMSO-d6.

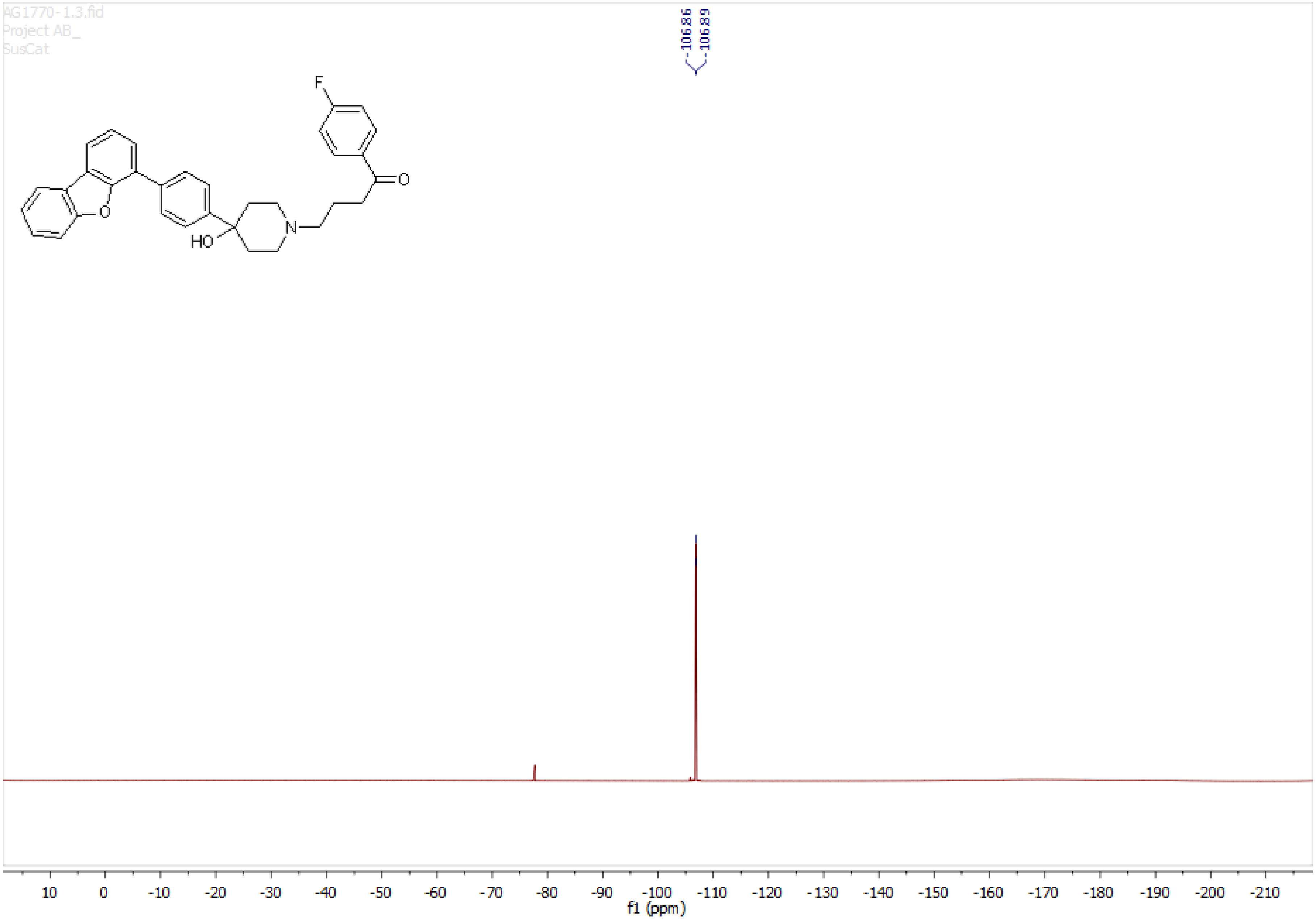

Supplement: CY-013-D3CY01375H-s001 [file CY-013-D3CY01375H-s001.pdf]
